# Analysis of the Role of FRMD5 in the Biology of Papillary Thyroid Carcinoma

**DOI:** 10.3390/ijms22136726

**Published:** 2021-06-23

**Authors:** Agata M. Gaweł, Maciej Ratajczak, Ewa Gajda, Małgorzata Grzanka, Agnieszka Paziewska, Marta Cieślicka, Maria Kulecka, Małgorzata Oczko-Wojciechowska, Marlena Godlewska

**Affiliations:** 1 Centre of Postgraduate Medical Education, Department of Biochemistry and Molecular Biology, Marymoncka 99/103, 01-813 Warsaw, Poland; agata.gawel@yahoo.com (A.M.G.); malgorzata.grzanka@cmkp.edu.pl (M.G.); 2 Faculty of Medicine, Medical University of Warsaw, Histology and Embryology Students’ Science Association HESA, Chałubinskiego 5, 02-004 Warsaw, Poland; 3 Centre of Postgraduate Medical Education, Department of Endocrinology, Marymoncka 99/103, 01-813 Warsaw, Poland; maciej.ratajczak@cmkp.edu.pl; 4 Centre of Postgraduate Medical Education, Department of Immunohematology, Marymoncka 99/103, 01-813 Warsaw, Poland; ewa.gajda@cmkp.edu.pl; 5 Centre of Postgraduate Medical Education, Department of Gastroenterology, Hepatology and Clinical Oncology, Marymoncka 99/103, 01-813 Warsaw, Poland; agnieszka.paziewska@cmkp.edu.pl (A.P.); maria.kulecka@cmkp.edu.pl (M.K.); 6 Centre of Postgraduate Medical Education, Department of Neuroendocrinology, Marymoncka 99/103, 01-813 Warsaw, Poland; 7 Department of Genetic and Molecular Diagnostics of Cancer, M. Sklodowska-Curie National Research Institute of Oncology Gliwice Branch, Wybrzeze Armii Krajowej 15, 44-102 Gliwice, Poland; marta.cieslicka@io.gliwice.pl (M.C.); malgorzata.oczko-wojciechowska@io.gliwice.pl (M.O.-W.)

**Keywords:** FRMD5, papillary thyroid carcinoma, *BRAF* V600E, migration, RNA sequencing

## Abstract

Background: Thyroid carcinoma (TC) is the most common endocrine system malignancy, and papillary thyroid carcinoma (PTC) accounts for >80% of all TC cases. Nevertheless, PTC pathogenesis is still not fully understood. The aim of the study was to elucidate the role of the FRMD5 protein in the regulation of biological pathways associated with the development of PTC. We imply that the presence of certain genetic aberrations (e.g., *BRAF* V600E mutation) is associated with the activity of FRMD5. Methods: The studies were conducted on TPC1 and BCPAP (*BRAF* V600E) model PTC-derived cells. Transfection with siRNA was used to deplete the expression of *FRMD5*. The mRNA expression and protein yield were evaluated using RT-qPCR and Western blot techniques. Proliferation, migration, invasiveness, adhesion, spheroid formation, and survival tests were performed. RNA sequencing and phospho-kinase proteome profiling were used to assess signaling pathways associated with the *FRMD5* expressional status. Results: The obtained data indicate that the expression of *FRMD5* is significantly enhanced in *BRAF* V600E tumor specimens and cells. It was observed that a drop in intracellular yield of FRMD5 results in significant alternations in the migration, invasiveness, adhesion, and spheroid formation potential of PTC-derived cells. Importantly, significant divergences in the effect of FRMD5 depletion in both *BRAF*-wt and *BRAF*-mutated PTC cells were observed. It was also found that knockdown of *FRMD5* significantly alters the expression of multidrug resistant genes. Conclusions: This is the first report highlighting the importance of the FRMD5 protein in the biology of PTCs. The results suggest that the FRMD5 protein can play an important role in controlling the metastatic potential and multidrug resistance of thyroid tumor cells.

## 1. Introduction

Tumors of the endocrine system are relatively rare. Nevertheless, thyroid cancers (TCs) are an exception. TCs belong to the five most frequently diagnosed cancers in women, where the highest number of thyroid carcinoma cases is recorded in middle-aged patients [1]. The most frequently diagnosed TC type is papillary thyroid carcinoma (PTC; up to 80% of all TC cases). The other, less frequent types of TCs are follicular thyroid carcinoma (FTC), anaplastic thyroid carcinoma (ATC), and medullary thyroid cancer (MTC) [2,3]. A large fraction of PTCs is accompanied by the presence of certain genetic alternations, such as single nucleotide mutations in the *BRAF* gene (especially *BRAF* V600E) and *RET* gene rearrangements (e.g., *RET/PTC*). Both genes are important regulators of the mitogen-activated protein kinase (MAPK) signaling pathway. *RET* encodes for receptor tyrosine kinase and RET/PTC fusion results in ligand-independent activation of the RET kinase. Similarly, single base mutations in the *BRAF* gene, which encodes serine-threonine kinase, lead to constitutive activation of MAPK signal transduction [4]. Mutations in these targets are mutually excluded, and *BRAF* mutant alleles are found in most (up to 80%) of all papillary tumors [5]. Moreover, it has been shown that the presence of the *BRAF* V600E allele in PTC is associated with an altered expressional profile of several other genes [6]. Therefore, such *BRAF* V600E-deregulated genes can be considered as potential drivers of malignant transformation and important prognostic or therapeutic targets.

Although the survival rate of the TCs is relatively high, the number of new cases is still growing [7]. Therefore, identification of new molecular players involved in carcinogenesis and understanding their factual biological functions might be a key factor for effective selection of groups of risk among patients and administration of more personalized treatments.

Here, we present a study on the FERM-domain containing protein 5 (FRMD5), which we found to be predominantly expressed in *BRAF*-mutated PTCs. FRMD5 is encoded by the *FRMD5* gene located on chromosome 15 (15q15.3). This 65 kDa protein belongs to the FERM-domain containing protein family, which occupies a unique position in cellular metabolism, performs structural functions, and participates in signal transduction. Although several members of this family have already been linked with tumor progression [8], the role of FRMD5 in cellular biology and tumorigenesis remains unclear. However, Brazdova et al. (2009) marked FRMD5 as a p53(R273H) target in U251 glioblastoma cells [9]. Additionally, the role of FRMD5 in p120-catenin-based cell–cell contact and regulation of lung tumor cell progression has been reported [10]. In other studies, Hu et al. (2014) proposed that FRMD5 may regulate lung cancer cell migration by interacting with integrin β5 cytoplasmic tail and ROCK1 [11]. Studies performed on the deregulation of the Wnt signaling pathway in colorectal cancer have highlighted FRMD5 as a novel target of the β-catenin/TCF7L2 complex, which plays an important role in tumorigenesis [12].

In the performed comprehensive analysis, we were particularly interested in studying the relationship between the expressional status of *FRMD5* and proliferative, invasiveness, and metastatic potential of PTC cells, as well as the expression of multidrug resistance proteins (MDRs). Our findings supported by NGS and phospho-kinase proteome profiling indicate significant discrepancies in the role of FRMD5 in both wild-type *BRAF* (*BRAF*-wt) and *BRAF*-mutated PTC cells. The data suggest that the mutational status of the cancer-driver *BRAF* gene might be crucial for the regulation of the expression of *FRMD5* and its activity in PTC cells.

## 2. Results

### 2.1. FRMD5 Expression Is Significantly Enhanced in BRAF-Mutated PTC Specimens and PTC-Derived Cell Lines

**Figure 1:**
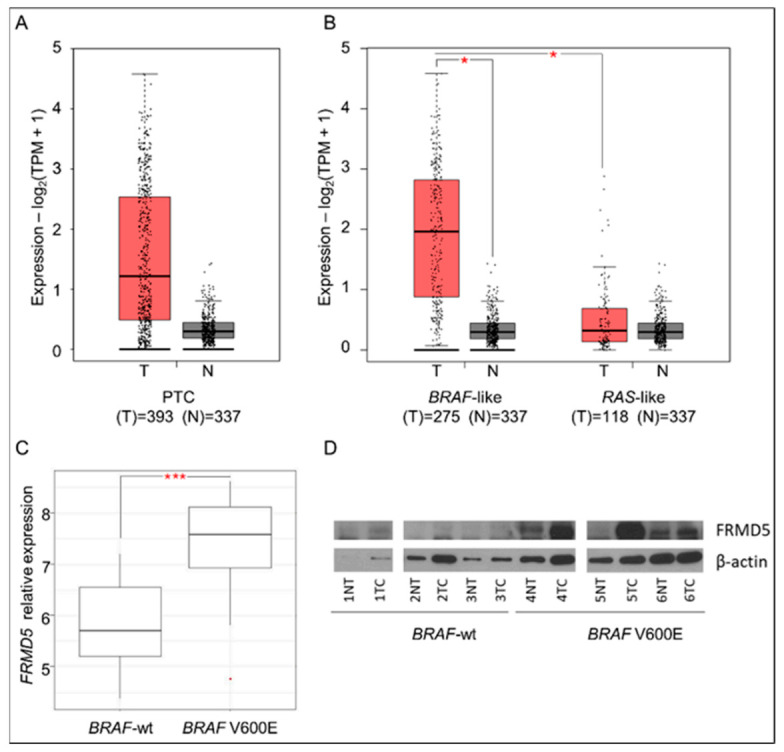
Analysis of *FRMD5* expression in human thyroid tissues. (**A**) Boxplot of *FRMD5* gene expression in papillary thyroid cancer (PTC) sections using The Cancer Genome Atlas (TCGA) databases and the Genotype Tissue Expression (GTEx) (tumor (T) vs. non-cancerous tissue (N)). (**B**) Boxplot of *FRMD5* gene expression in *BRAF*-like and *RAS*-like PTC subtypes vs. non-cancerous tissues. (**C**) Boxplot of *FRMD5* gene expression in PTC bearing the wild-type (*BRAF*-wt) and mutated *BRAF* (*BRAF* V600E) gene determined by microarray analysis. (**D**) Western blot analysis of FRMD5 protein expression in *BRAF*-wt and *BRAF* V600E PTCs and non-tumoral (NT) tissues. β-actin serves as a loading control. Data are presented as mean ± SD.* *p* < 0.05; *** *p* < 0.0001.

Primarily, the expressional status of *FRMD5* in TC specimens was analyzed using The Cancer Genome Atlas (TCGA) and Genotype Tissue Expression (GTEx) databases. The performed analysis using the GEPIA tool revealed that overall, the expression of *FRMD5* in PTC sections is enhanced (*n* = 393) comparing to control, non-cancerous tissues (*n* = 337). The average expression of *FRMD5* in tumor samples was ~3-fold higher than in non-tumor controls (Figure 1A).

As a particular focus was given to the expressional status of *FRMD5* in *BRAF*-mutated PTCs, an analysis of *FRMD5* activation status was performed on the *BRAF*-like molecular subtype of PTC and compared with other TC types and non-tumor (control) specimens. Altogether, *BRAF* mutations and *RET* rearrangements are termed as *BRAF*-like carcinomas and present similar expression patterns. As the predominantly harbored mutation is *BRAF* V600E (observed in up to 80% of PTCs cases), most *BRAF*-like samples correspond to *BRAF* V600E-mutated TCs [13].

The performed examination showed significant and strong upregulation of *FRMD5* in the *BRAF*-like cohort (*n* = 275; Figure 1B). Detailed analysis of PTC specimens also unfolded a significant disproportion in the *FRMD5* expressional status between *BRAF*-like and *RAS*-like samples (*n* = 118; Figure 1B). Accordingly, the same prevalence was found in a cohort of 54 samples of PTCs (30 *BRAF*-wt and 24 *BRAF* V600E) used for microarray data characterization and analysis of PTC expressional profiles in previous studies [6,14]. As shown in Figure 1C, the subset of *BRAF* V600E PTC samples presented significant upregulation of expression of the *FRMD5* gene when compared to *BRAF*-wt PTCs. An observed predominant activation of FRMD5 in *BRAF*-mutated specimens was tested in *BRAF* V600E tumor samples using Western blot analysis. In some cases, an increased yield of the FRMD5 protein in *BRAF* V600E specimens was observed (Figure 1D).

### 2.2. Depletion of FRMD5 Affects Migration, Invasive Potential, and Spheroid Formation of PTC Cells

**Figure 2:**
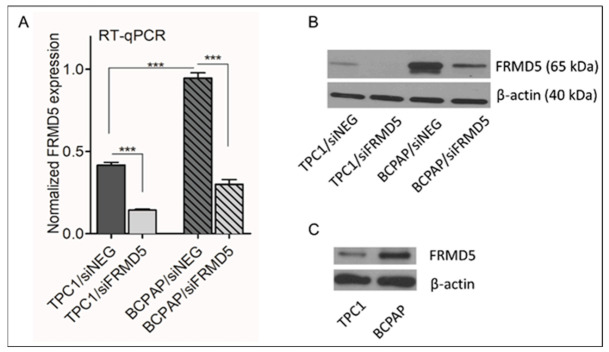
*FRMD5* expression at transcript (**A**; RT-qPCR) and protein level (**B**; Western blot) in TPC1 and BCPAP cell lines transfected with FRMD5-specific siRNA (siFRMD5) and a scrambled control (siNEG). (**C**) Native yield of FRMD5 in non-transfected TPC1 and BCPAP cells determined by Western blot. β-actin served as a loading control. Graphical data are presented as mean ± SD. *** *p* < 0.0001.

Two classic model PTC-derived cell lines, TPC1 and BCPAP, were selected to establish an in vitro cellular model for analysis of biological properties of FRMD5 in PTC. TPC1 and BCPAP cell lines harboring the *RET/PTC* alternation or *BRAF* V600E allele, respectively, presented significant imbalance in the *FRMD5* expressional status, as determined by RT-qPCR and Western blot analyses. As shown in Figure 2, *BRAF*-mutant BCPAP cells (BCPAP/siNEG) presented 2-fold greater expression (Figure 2A) and intracellular yield (Figure 2B) of *FRMD5* than TPC1 cells harboring the *BRAF*-wt allele (TPC1/siNEG). The observed discordance in *FRMD5* expression overlaps with the *FRMD5* transcript pattern detected in clinical specimens. The *FRMD5* gene was targeted with specific small interfering RNA (siFRMD5) to examine the role of FRMD5 in the biology of PTC. Cells treated with non-targeting siRNA (siNEG) served as controls. It was observed that the expression of *FRMD5* was decreased by 75% in both the TPC1/siFRMD5 and BCPAP/siFRMD5 cells (Figure 2A). Similarly, Western blot analysis showed a strong reduction in FRMD5 protein yield in siFRMD5-treated samples in comparison to the controls (Figure 2B).

**Figure 3:**
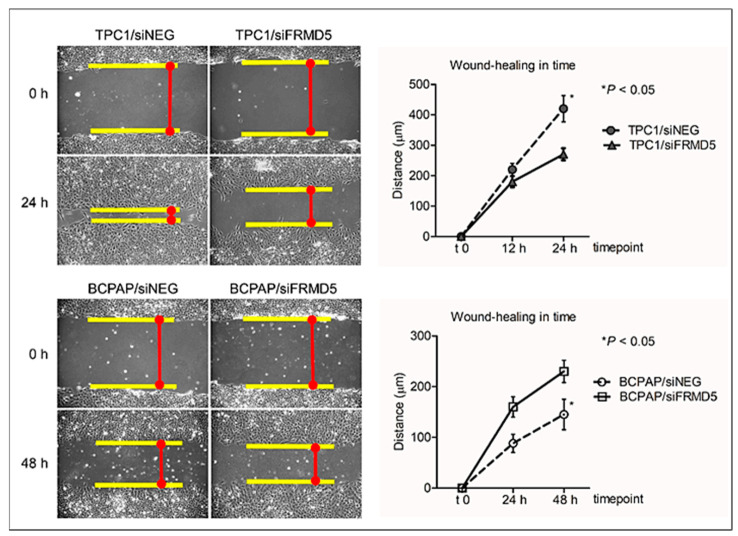
Silencing of *FRMD5* results in discrepant migratory and invasive potentials of TPC1 and BCPAP cells. The left panel shows representative images (10× lens) of wounds in monolayers of TPC1 and BCPAP cells transfected with siNEG (controls) or siFRMD5, at two time points: t0 (scratch area at time point 0) and t2 (scratch area at time point 24 h for TPC1 and 48 h for BCPAP) after scratch application. The right panel shows a graphical analysis of relative migration of transfected cells measured at t0, t1 (scratch area at time point 12 h for TPC1 and 24 h for BCPAP), and t2 time after scratch application. The cell migration distance was calculated by measuring the wound width, dividing it by two, and subtracting this value from the initial half-wound width. Data are presented as mean ± SD. * *p* < 0.05.

Biological analysis revealed significant alternations in the motility potential of both TPC1 and BCPAP cells treated with siFRMD5. The performed wound-healing assay showed that *FRMD5*-silencing in TPC1 triggers a strong anti-migratory phenotype, while BCPAP cells deficient in FRMD5 migrate faster than control cells. A graphical summary of the data showed a statistically significant (*p* < 0.05) ~3-fold reduction in distance traveled by TPC1/siFRMD5 cells and a nearly two-fold increase in distance traveled by siFRMD5-treated BCPAP cells, comparing to respective controls (cells treated with siNEG; Figure 3).

**Figure 4:**
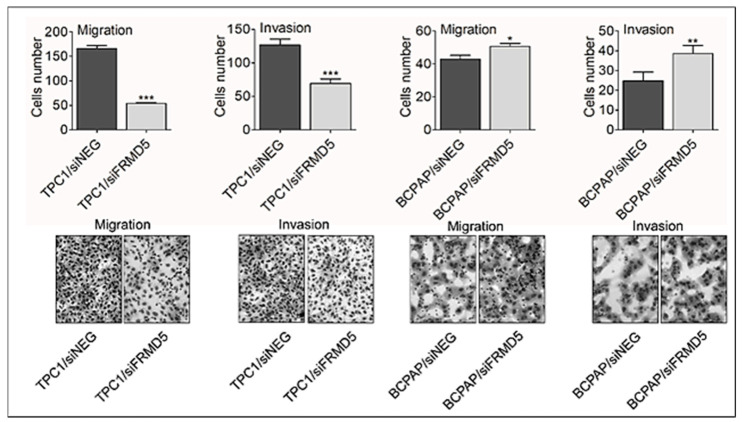
FRMD5 depletion alters the migration and invasion capacities of TPC1 and BCPAP cells. Cells were resuspended in null medium and seeded in migration and Matrigel invasion insert chambers (8-µm pore size), then placed in 24-well plates filled with medium containing a chemoattractant (10% fetal bovine serum; FBS). After 24 h of incubation, the migrated cells were stained and photographed using a light microscope equipped with a camera (40× lens). Representative images are shown on the bottom panel. The obtained data indicating the number of migrated cells are presented on the graphs (top panel). *FRMD5*-knockdown significantly reduced migration and invasiveness of TPC1 cells. Depletion of FRMD5 promoted motility of BCPAP cells. Data are presented as mean ± SD. * *p* < 0.05; ** *p* < 0.001; *** *p* < 0.0001.

Congruently, FRMD5 depletion resulted in the opposite migratory and invasive capabilities of the tested TPC1 and BCPAP cells, which was confirmed using the transwell migration and invasion assays. The obtained data confirmed a 3-fold and 2-fold reduction in migration and invasiveness potential, respectively, in *FRMD5*-deficient TPC1 cells, in comparison to the siNEG treated controls. Respective to scratch assay data, knockdown of *FRMD5* in the *BRAF*-mutated BCPAP cell line resulted in a statistically significant (up to two-fold) surge in the number of migrated and invasive cells (Figure 4).

**Figure 5:**
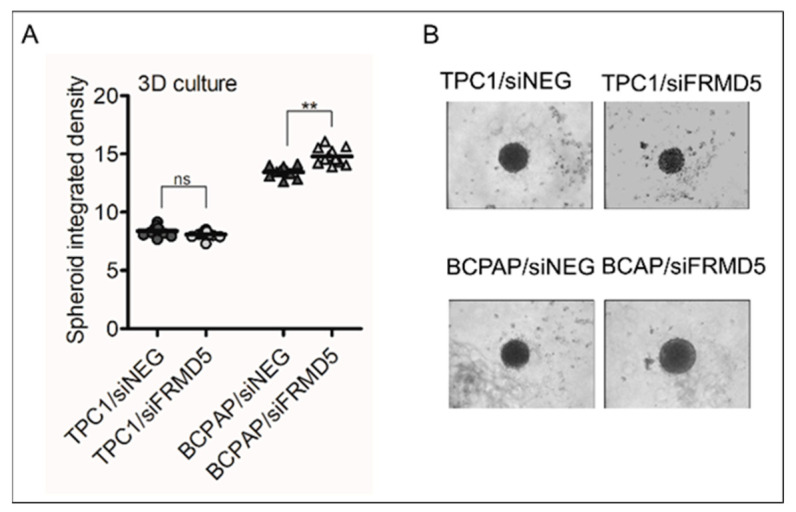
*FRMD5*-knockdown affects the growth of tumor spheroids. TPC1 and BCPAP cells deficient in FRMD5 and grown in a 3D model present significantly reduced or increased dimensions and shape of the formed spheroids, respectively. (**A**) Graphical plots summarizing the average integrated density of spheroids formed by cells treated with siNEG (controls) or siFRMD5. (**B**) Representative images of the formed spheroids on day 6. Magnification: 10× lens. Data are presented as mean ± SD. ** *p* < 0.001; ns, non-significant.

Additionally, the effect of FRMD5 depletion on the cells’ metastatic capability in vitro was investigated. A scaffold-free cell spheroid in a suspension model was used, which has been established to mimic the growth of naturally occurring tumors. It was found that deficiency of *FRMD5* expression promotes the expansion of BCPAP cells. In contrast, no significant effect on the size and integrated density of spheroids for TPC1/siFRMD5 cells was observed in comparison to the control (TPC1/siNEG; Figure 5).

### 2.3. FRMD5 Does Not Impact the Viability and Apoptosis of PTC Cells

The obtained data collectively indicated that FRMD5 may be an important factor controlling the migration, invasiveness, and 3D cellular aggregation of PTC cells. In order to examine whether the observed pro- and antimetastatic properties of FRMD5 are associated with alternations in viability or proliferation, trypan blue-based, MTS [3-(4,5-dimethylthiazol-2-yl)-5-(3-carboxymethoxyphenyl)-2-(4-sulfophenyl)-2H-tetrazolium]-based and BrdU (5-bromo-2′-deoxyuridine) incorporation analyses were performed.

**Figure 6:**
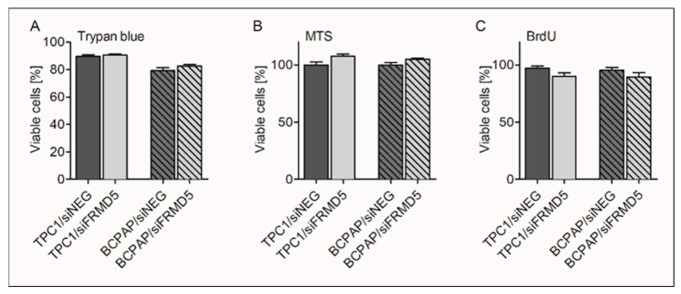
Effect of *FRMD5* silencing on the viability of TPC1 and BCPAP cells. (**A**) Trypan blue dye exclusion assay; (**B**) MTS-based cells viability analysis; (**C**) BrdU-based cell proliferation assay. Data showed non-significant changes in viability and proliferation between the control (siNEG) and siFRMD5-deficient cells. Data are expressed as mean ± SD.

Despite significantly altered migratory and invasive capacities of the tested *FRMD5*-deficient cell lines, only minor changes in the cell’s viability, growth, and division were observed (Figure 6).

Additionally, flow cytometry-based apoptosis analysis was performed. After 48 h post-transfection with designated siRNA, TPC1 and BCPAP cells were stained with Annexin V-fluorescein isothiocyanate (FITC) conjugate and propidium iodide. Flow cytometry revealed that the fraction of viable, apoptotic, and necrotic cells in all the tested samples was similar (Supplementary Figure S1).

### 2.4. FRMD5 Alters the Adhesion of PTC Cells

**Figure 7:**
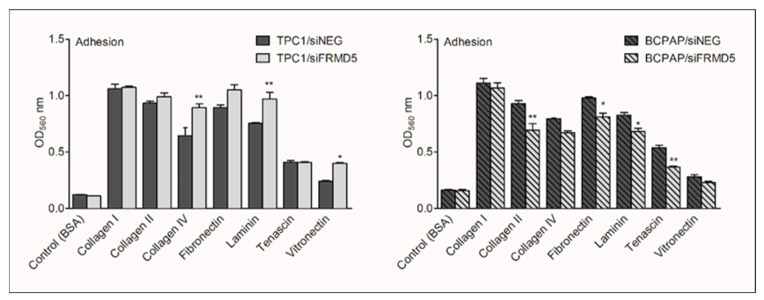
*FRMD5*-knockdown affects cell-extracellular matrix (ECM) interactions of TPC1 and BCPAP cells. The left and right panel shows adhesion assay performed on TPC1 and BCPAP cells, respectively. Data are expressed as optical density (OD_560_), which refers to the number of attached cells. The graphs present the fraction of attached cells to collagen I-, collagen II-, collagen IV-, fibronectin-, laminin-, tenascin-, and vitronectin-coated wells. Bovine serum albumin (BSA)-coated wells served as a negative control. Gray bars mark *FRMD5*-silenced cells, and dark-color bars indicate cells treated with control siRNA (siNEG). Data are presented as mean ± SD. * *p* < 0.05; ** *p* < 0.001.

To further study the properties of FRMD5 linked with metastasis, the effect of *FRMD5*-knockdown on the attachment capabilities of cells to extracellular matrix (ECM) proteins was investigated. A commercial ECM adhesion array assay was used for analysis, as described in the Materials and Methods section. After 72 h post-transfection, the tested cells were seeded on collagen I-, collagen II-, collagen IV-, fibronectin-, laminin-, tenascin-, and vitronectin-coated wells for 2 h. Cells seeded on wells coated with bovine serum albumin protein (BSA) served as a negative control. The cell adhesion characteristics showed that *FRMD5* silencing led to opposite effects in the tested cells. Overall, FRMD5 suppressed the adhesion of TPC1 cells, as it was found that depletion of FRMD5 significantly increased the binding of TPC1 cells to collagen IV, laminin, and vitronectin. In contrast, FRMD5 likely acted as a promotor of cell-matrix interaction in BCPAP cells, as cells deficient in FRMD5 exhibited reduced adherence to collagen II, fibronectin, laminin, and tenascin (Figure 7).

### 2.5. FRMD5 Depletion Is Associated with Activation of Multidrug Resistance Genes and Increased Chemoresistance

One of the major therapeutic problems leading to high cancer-related mortality is the phenomenon of multidrug resistance (MDR) observed in various tumors. MDR significantly reduces the effectiveness of administered drug therapies, and there are several mechanisms involved in its induction. High expression and activity of several ATP binding cassette (ABC) transporters, such as P-glycoprotein (*ABCB1*/P-gp), breast cancer resistance protein (*ABCG2*/BCRP), and multidrug resistance-associated proteins 1 and 6 (*ABCC1*/MRP1; *ABCC6*/MRP6, respectively), play a crucial role in the MDR phenomenon.

**Figure 8:**
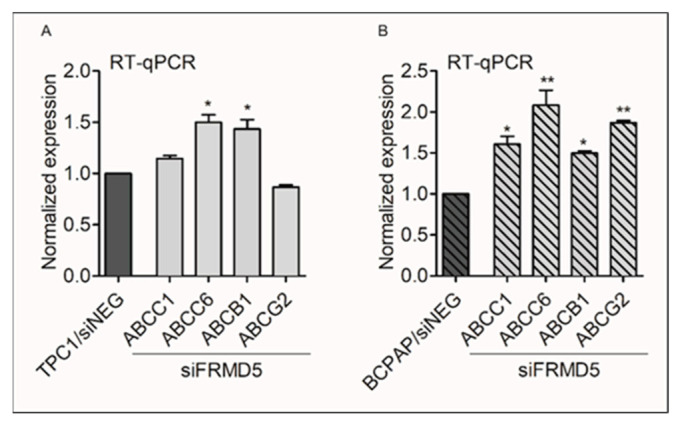
Knockdown of *FRMD5* significantly affects the expression patterns of the multidrug resistance (MDR) genes in TPC1 and BCPAP cells. Transcript-level expression analysis of MDR genes in TPC1 (**A**) and BCPAP (**B**) cell lines transfected with *FRMD5*-specific siRNA (siFRMD5) or siNEG (control) using RT-qPCR. Graphical data are presented as mean ± SD. * *p* < 0.05; ** *p* < 0.001.

Since depletion of FRMD5 displays mixed oncogenic activity depending on the cancer cell mutational status, the expressional profile of P-gp-, BCRP-, MRP1- and MRP6-encoding genes was evaluated in *FRMD5*-knocked-down TPC1 and BCPAP cells using RT-qPCR. It was observed that suppression of *FRMD5* results in upregulation of the tested MDR genes. BCPAP cells exhibited significant (up to two-fold) induction of all four MDR genes, while TPC1 siFRMD5-treated cells showed an increase in the expression of MRP6- and P-gp-encoding genes (Figure 8).

**Figure 9:**
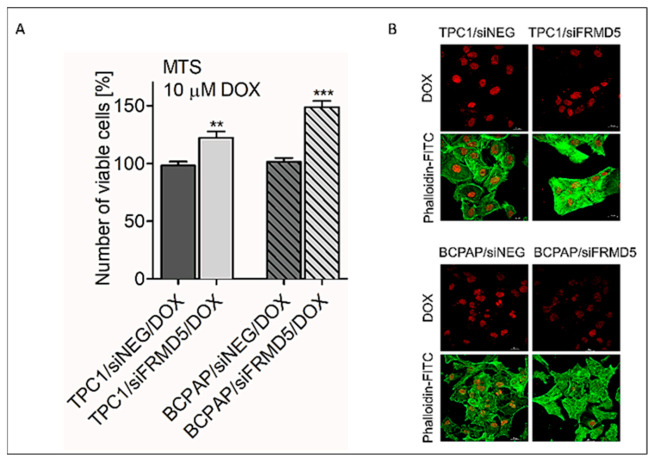
Knockdown of *FRMD5* affects chemoresistance of TPC1 and BCPAP cells. (**A**) MTS-based analysis of *FRMD5*-deficient TPC1 and BCPAP cells exposed to doxorubicin (DOX) for 24 h. (**B**) Representative confocal microscopy images display intracellular DOX accumulation (red signal) in fluorescently labeled (fluorescein isothiocyanate (FITC)-conjugated phalloidin; green signal) cells. Images were recorded using 63×/1.4 oil DIC M27 lens. Cells treated with non-targeting siRNA (siNEG) were used as a control. Graphical data are presented as mean ± SD. ** *p* < 0.001; *** *p* < 0.0001.

To further determine the linkage between the expression of *FRMD5* and MDR genes’ activity, a MTS-based cell viability analysis was performed. TPC1 and BCPAP cells treated with siNEG (control) or siFRMD5 were supplemented with 10 µM of doxorubicin (DOX) and further incubated for 24 h. It was considered that since FRMD5 depletion leads to activation of MDR-encoding genes, cells treated with the chemotherapeutic should present increased tolerance for the drug and enhanced viability. The obtained data showed that preincubation of cells with siFRMD5 resulted in a pro-survival effect in both the TPC1 and BCPAP cell lines. A 20% and 50% increase in the number of viable cells in siFRMD5/DOX treated TPC1 and BCPAP cells, respectively, was observed in comparison to the control (Figure 9A). These observations were further evaluated, visualized, and confirmed using confocal imaging (Figure 9B).

### 2.6. FRMD5 Expression Deficiency Interferes with the Activation Status of Signal Transduction Proteins

**Figure 10:**
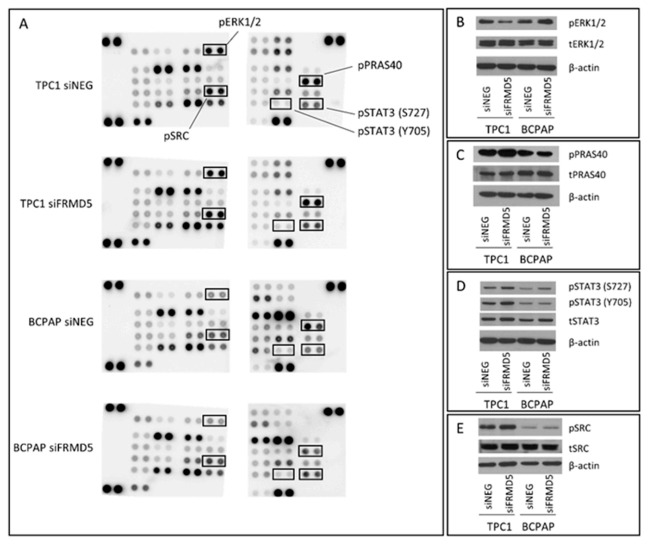
Analysis of the impact of FRMD5 depletion on signal transduction pathways in the TPC1 and BCPAP cell lines. (**A**) Human phospho-kinase array analysis of cells transfected with *FRMD5*-specific siRNA (siFRMD5) and non-targeting siRNA (siNEG; control). (**B**–**E**) Verification of human phospho-kinase array results using the Western blot technique. β-actin served as a loading control.

In order to gain insight into biological pathways and genes associated with biological alterations resulting from FRMD5 depletion, the Proteome Profiler Human Phospho-Kinase Array (R&D Systems, Minneapolis, MN, USA) analysis was performed. This membrane-based sandwich immunoassay allows for a fast study of the antibody array to detect specific changes in phosphorylation of specific kinases involved in signal transduction of major regulatory pathways and compare their activation between the tested samples. TPC1 and BCPAP cells treated with siNEG (control set) and siFRMD5 were used. Among 37 examined phospho-kinases and 2 related total proteins, 17 molecules in total exhibited an altered activation status. Ten of the kinases were deregulated in both *FRMD5*-deficient TPC1 and BCPAP cell lines. Among deregulated proteins, YES kinase was suppressed, while STAT5, Chk-2, SRC, PRAS40, STAT3 (S727), RSK1/2 were upregulated (Figure 10A and Figure S2). Activation of two of the phosphokinases, AKT and CREB, was enhanced in TPC1/siFRMD5, but downregulated in BCPAP/siFRMD5 cells. Only one protein, c-JUN, was uniquely upregulated in TPC1 *FRMD5*-deficient cells, while only six kinases (ERK, p53, PYK2, STAT1, STAT6, p70 S6) were activated in siFRMD5-treated BCPAP cells. Additionally, Western blot analysis was performed to confirm the obtained immunoassay data. As shown in Figure 10B–E, the activation status of five randomly selected kinases (ERK1/2, STAT3, PRAS40, and SRC) overlapped with the phospho-kinase profiler data.

### 2.7. FRMD5-Knockdown Significantly Alters the Transcriptome of PTC-Derived Cells

**Figure 11:**
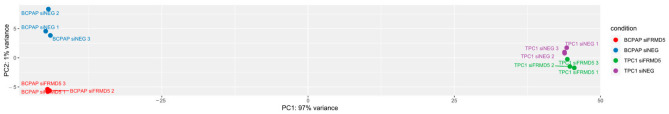
Principal component analysis (PCA) of RNA sequencing (RNA-Seq) datasets for *FRMD5*-knocked-down TPC1 and BCPAP cell lines. PCA was determined from the abundance of all transcripts detected in RNA-Seq analysis. Cells transfected with non-targeting small interfering RNA are marked as siNEG (control). Cells transfected with *FRMD5*-specific siRNA are marked as TPC1/siFRMD5 and BCPAP/siFRMD5.

As the obtained data imply that the presence of certain genetic aberrations in genes encoding for key trigger proteins of major signaling pathways is associated with FRMD5 depletion, RNA sequencing (RNA-Seq), which uses next-generation sequencing (NGS), was used for transcriptome profiling of TPC1 and BCPAP cells treated with non-targeting siRNA (siNEG; controls) or siRNA (siFRMD5) targeting the *FRMD5* gene. Principal component analysis (PCA) was completed on raw data by separating a group of siNEG from siFRMD5 samples (Figure 11). Comparison analysis of TPC1 cells deficient in FRMD5 and TPC1 cells treated with control siRNA showed a significantly altered expression of 1543 genes (749 were upregulated and 794 downregulated; adjusted *p*-value < 0.05; Supplementary Tables S1 and S2). Further analysis of genes expressed in *FRMD5*-suppressed and siNEG-treated BCPAP cells revealed deregulation of 1032 genes, where 517 of them presented increased, and 515 showed decreased expression (adjusted *p*-value < 0.05; Supplementary Tables S3 and S4).

The most upregulated genes in FRMD5-depleted TPC1 cells included: *CXCL6*, *ZNF718*, *CPA4*, *KCNN3,* and *NUAK2*, while the most often downregulated were: *SH2D1B*, *CSF3*, *NOX5*, *EGR1,* and *SERPINB2*. In the FRMD5-silenced BCPAP cell line, *CXCL5*, *WWC1*, *KRTAP2*-3, *LRRC38,* and *PDGFB* were found among the most upregulated genes, while *AMACR*, *CDH6*, *TNFRSF9*, *IDO1,* and *ARID5B* were utmostly downregulated. According to Gene Ontology (GO) enrichment analysis, the most commonly overexpressed genes in TPC1 were predominantly associated with regulation of the metabolic process, gene expression, cellular component, organelle organization, and ion binding. In BCPAP cells, upregulated genes are involved in integrin binding, chemokine activity, CXCR chemokine receptor binding, hepatocyte growth factor receptor binding, transcription coactivator activity, and protein binding, indicating significant discrepancies in the pattern of deregulated genes.

**Figure 12:**
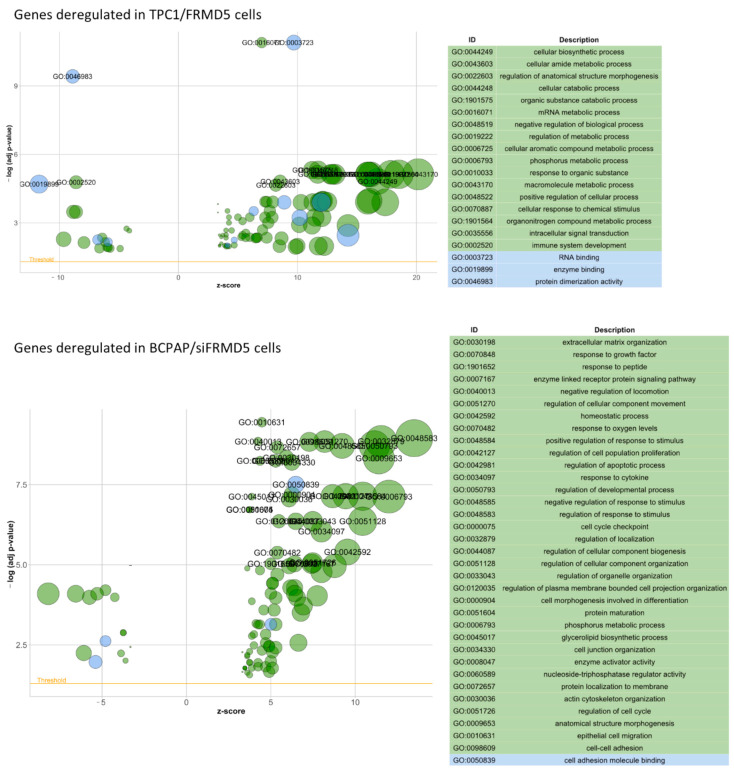
Bubble plots of significantly enriched Gene Ontology (GO) terms for the *FRMD5*-depleted TPC1 (top panel) and BCPAP (bottom panel) cells after term similarity reduction. Terms with adjusted *p*-value on the PHRED scale higher than 4 (TPC1) and 5 (BCPAP) are annotated. The area of the circles is proportional to the number of genes assigned to the term in the analysis. The z-score (horizontal axis) indicates whether the given term is more likely to be up- or downregulated. The threshold indicates the adjusted *p* < 0.05.

GO enrichment examination of the differentially expressed genes in *FRMD5*-deficient TPC1 and BCPAP cells resulted in the identification of multiple GO terms belonging to two ontology sources: biological processes and molecular functions. Out of 407 hits found in upregulated TPC1/siFRMD5 samples, 375 GO terms were linked with biological processes, while only 32 GO terms were associated with molecular functions (Supplementary Table S5). In contrast, GO enrichment analysis of downregulated genes in TPC1/siFRMD5 cells was manifested by 773 GO terms linked with biological processes, among which at least 5 are involved in cell migration, and 36 GO terms are linked with molecular functions (Supplementary Table S6). The top of the GO terms in TPC1/siFRMD5 cells was related to cellular biosynthesis, catabolic processes, and RNA binding (Figure 12, upper panel). A similar analysis in BCPAP/siFRMD5 samples showed 1500 upregulated GO terms (1473 associated with biological processes and 27 linked with molecular functions; Supplementary Table S7) and 111 downregulated GO terms (105 associated with biological processes and 6 linked with molecular functions; Supplementary Table S8). At least 13 GO terms belonging to the upregulated biological processes are related to cell migration. Top of the GO terms determined in BCPAP/siFRMD5 samples are related to intracellular transport, cilium movement, organization, regulation of organelle, and cytoskeleton organization (Figure 12, lower panel).

A RT-qPCR analysis was performed using the same RNA samples that were used for RNA-Seq analysis to confirm RNA-Seq-based gene expression data. The analyzed genes were selected randomly among those whose expression was found to be significantly altered (up- or downregulated) in RNA-Seq-based analysis (Supplementary Tables S1–S4) and was involved in GO terms associated with cell migration (Supplementary Tables S5–S8). For all of the screened genes (Table 1), changes in the expression profiles, which had originally been found in RNA-Seq data, were confirmed by RT-qPCR (Table 1).

## 3. Discussion

FRMD5 is a still poorly understood FERM-domain protein. Its biological function is unclear. However, relatively high expression of the *FRMD5* gene in various human tissues, including brain, kidney, thyroid, and testis, underlines its importance for biological processes. Former research concerning FRMD3, a close homolog of FRMD5, has emphasized the role of this FERM protein in tumorigenesis [15]. Likewise, the few studies performed on FRMD5 have also suggested its involvement in carcinogenesis, although its functions in this process still remain indistinct [9,10,11,12].

Here, we performed an analysis on the role of FRMD5 in thyroid cancer, as we found significant discrepancies in *FRMD5* expression between *BRAF*- and *RET*-mutated PTCs, as well other types of TCs (TCGA and microarray data analysis of clinical specimens). *BRAF* and *RET* tumorigenic genes are the most abundant among TCs and share a common property of signaling via activation of the MEK-ERK kinase pathway. Nevertheless, cells harboring these mutations present unique phenotypic features, signifying the different tumor biology resulting from unequal gene expressional profiles. Based on analyses of *FRMD5* expression in human thyroid cancer tissue, it was concluded that the high expression of the *FRMD5* gene may be associated with the presence of the *BRAF* V600E genetic aberration.

Here, we performed an analysis on the role of FRMD5 in thyroid cancer specimens and cell lines, as we found significant discrepancies in *FRMD5* expression between *BRAF*- and *RET*-mutated PTCs, as well other types of TCs (TCGA and microarray data analysis of clinical specimens). *BRAF* and *RET* tumorigenic genes are most abundant among TCs and share a common property of signaling via activation of the MEK-ERK kinase pathway. The nature of imbalance in FRMD5 intracellular yield and expression observed in cells harboring *BRAF*-wt and *BRAF* V600E is not clear and might result from overall unequal gene expressional profiles, which determines phenotypic features and unique tumor biology cells harboring these mutations. Based on analyses of *FRMD5* expression in human thyroid cancer tissue, it was concluded that high expression of the *FRMD5* gene may be associated with the presence of the *BRAF* V600E genetic aberration.

To search for biological processes and define genes associated with *FRMD5* expression, we knocked down *FRMD5* in PTC-derived, TPC1, and BCPAP cells, harboring *RET/PTC* and *BRAF* V600E mutations, respectively. We found that the depletion of FRMD5 significantly increases the migration and invasiveness, as well as promotes spheroid formation of BCPAP cells. In contrast, the depletion of FRMD5 in TPC1 cells strongly suppressed their metastatic potential. The observed diverse impact of *FRMD5* knockdown was not associated with alterations in the cells’ viability, proliferation, or apoptosis, as they remained unaffected. Together, these data strongly indicate that the observed disproportions in the motility of FRMD5-deficient cells do not result from altered viability of cells, but rather other processes, such as impaired transduction signal trafficking by major pathways involved in the control of migration processes, cell–cell or cell–matrix adhesion.

Partly, the effect of FRMD5 on the metastatic potential of PTC cells stays in accordance with previously published studies on glioblastoma, lung, and colorectal cancer (mentioned in the Introduction), suggesting its pro-migratory potential. Nevertheless, our findings also imply its antimetastatic character in *BRAF*-mutated cells, indicating that the expressional status and functions of FRMD5 may not be dependent on the type of cancer tissue but rather on genetic alternations in cancer cells. Interestingly, such an anti-migratory gene expression-dependent effect was previously reported for podoplanin, a membrane glycoprotein, which is also predominantly expressed in mutant *BRAF* V600E PTC cells [16].

Altered motility of FRMD5-deficient TPC1 and BCPAP cells was also associated with increased or decreased (respectively) adhesion to selected ECM proteins. Therefore, discrepancies in adherence of siFRMD5-treated TPC1 and BCPAP cells may at least partially explain the detected opposite migratory patterns.

Another novel finding concerned the ability of FRMD5 to modulate multidrug resistance genes. Intriguingly, depletion of FRMD5 in both cell lines resulted in the upregulation of key ABC transporter genes (P-gp and MRP6). This, in turn, led to increased survival of the cells treated with the chemotherapeutic agent (DOX). We cannot exclude that FRMD5 affects the expression of MDR genes indirectly. We consider that the mechanism of MDR regulation is rather independent of the *BRAF* mutational status, as a decrease in DOX sensitivity was observed in both tested cell lines. As induction of drug resistance by FRMD5 is unclear, the mechanism and critical molecular players involved in this phenomenon must be further elucidated.

Phospho-kinase array and RNA-Seq analyses were completed to identify signaling pathways and genes associated with the observed FRMD5 phenotype. The study on the profile of phospho-kinases revealed that lack of FRMD5 may significantly disturb the activity of various signaling pathways. The activation status of several kinases, including SRC and two STAT family kinases (3 and 5), was upregulated in both cell lines, while other kinases were deregulated uniquely in only one of the cell lines. For example, two STAT (1 and 6) and extracellular signal-regulated kinases 1/2 (ERK1/2) proteins were upregulated only in BCPAP cells, while c-JUN was repressed in TPC1 *FRMD5*-deficient cells. Upregulation of STAT family proteins is especially intriguing, as they are partners of JAK, another FERM-domain protein, and play a significant role in tumorigenic processes. Disrupted JAK-STAT signaling affects cellular growth and transformation, preventing apoptosis or tumor formation [17]. Therefore, it can be considered that an interaction between the FRMD5 protein and the JAK-STAT axis may exist.

Furthermore, a detailed analysis of ERK1/2 in FERM-depleted cells showed their increased activation in BCPAP and decreased phosphorylation in TPC1 cell lines. As ERK1/2 is one of the major regulators of the mitogen-activated protein kinase (MAPK) pathway, their deregulation may partially explain the observed variances in metastases of *FRMD5*-silenced TPC1 and BCPAP cells. In addition, other phosphokinases, AKT and cAMP response element-binding protein (CREB), were upregulated in TPC1 and downregulated in BCPAP *FRMD5*-depleted cells. AKT is a critical regulator of the PI3K/AKT/mTOR signaling pathway [18], while CREB is considered a key mediator in carcinogenesis, including invasion and metastasis [19]. As apoptosis was not significantly affected by *FRMD5* silencing in both cell lines, an alternative role of the PI3K/AKT/mTOR pathway must be considered.

The role of altered adhesion, hampering migration of siFRMD5-treated TPC1 cells, may be supported by the activation of the SRC kinase in FRMD5-depleted cells. Surprisingly, β-catenin, another important protein involved in the regulation of cell–cell adhesion and previously marked as one of the potential FRMD5 partners, was not affected in *FRMD5*-silenced cells.

The analysis using the RNA-Seq technique revealed hundreds of genes, which were up- or downregulated in *FRMD5*-suppressed TPC1 and BCPAP cells. Examination of the GO term enrichment of the differentially expressed genes in both studied cell lines revealed distinct differences. In siFRMD5-treated TPC1 cells, none of the GO terms of the upregulated genes were linked with cell migration. In contrast, a similar analysis of GO terms performed for siFRMD5-treated BCPAP cells revealed significant upregulation of genes associated with extracellular matrix organization and migration. GO terms representing downregulated genes in TPC1/siFRMD5 cells were associated with the suppression of the cells’ motility. In contrast, gene ontology enrichment analysis of the downregulated genes in BCPAP/siFRMD5 cells showed changes in processes related to cilium organization and movement, microtubule- and cytoskeleton-dependent transport.

Additional analysis of alternatively expressed genes in siFRMD5-treated cells showed significant upregulation of matrix metalloproteinases (MMPs)-encoding genes (e.g., MMP1 and MMP9) in BCPAP cells. MMPs are critical enzymes targeting extracellular matrix components, including collagen, elastin, fibronectin, and laminin proteins. MMP-driven degradation of ECM results in the remodeling of cells and promotes their detachment. Furthermore, MMPs affect cell adhesion receptors, such as E-cadherin or integrins, and therefore directly disrupt cell–cell adhesion [20]. As MMPs are the most significant enzymes in the degradation of the extracellular matrix, their observed upregulation may explain both observed increased motility and decreased adhesion of BCPAP/siFRMD5 cells. Overall, RNA-Seq and GO term profiles stay in accordance with the observed phenotype of FRMD5-deficient PTC-derived cells, which showed significant changes in migratory properties.

In conclusion, we have provided a novel insight into the role of FRMD5 in cancer cell biology. Moreover, it is the first trial reporting a genome-wide analysis of gene expression in FRMD5-deficient cells. Our findings indicate that the biological properties of FRMD5 are strongly dependent on the mutational status of cancer cells. It has been shown that lack of FRMD5 promotes the cells’ migration, likely via activation of the MAPK pathway of *BRAF*-mutated BCPAP cells but decreases motility of PTC-derived *BRAF*-wt TPC1 cells. It has also been presented that FRMD5 depletion affects the expression of various other contributors of metastases, mainly by regulating cellular matrix organization, cell–cell, and cell–matrix adhesion.

## 4. Materials and Methods

### 4.1. Retrieval of TCGA and GTEx Data

A visualization website GEPIA (Gene Expression Profiling Interactive Analysis), available at http://gepia2.cancer-pku.cn/#analysis (accessed on 24 May 2021), was used to analyze *FRMD5* expression in cancer and normal thyroid tissues in the Genotype Tissue Expression (GTEx) and the Cancer Genome Atlas (TCGA) projects data. The expression analyses were performed using the following terms: Gene, *FRMD5*; Dataset (cancer name), THCA; log 2-fold change cutoff, 1; *p*-value cut-off, 0.05; differential methods, ANOVA.

### 4.2. Microarray Data Analysis

*FRMD5* gene expression analysis was examined using previously published microarray data [6,14]. The dataset contained 54 samples of PTC, including 30 *BRAF*-wt and 24 *BRAF* V600E. The quality of the data was checked using dedicated software—Affymetrix Expression Console. Analysis was performed on gene and exome levels according to Affymetrix’s whitepaper. All data passed the quality test. Further analysis was performed in R Studio. Raw data were pre-processed; background subtraction, normalization, and summarization were performed with the fRMA algorithm implemented in the fRMA package. Arguments specifying methods for each step were selected as follows: for background correction, it was RMA, for normalization—quantile method, and for summarization: median polish. Due to the occurrence of batch effect between two series of sample processing, batch effect removal was necessary. It was performed with COMBAT (Empirical Bayes method), implemented in SVA package. Afterward, control probe sets were removed, and only those described as “main” in Affymetrix documentation were used for further analysis. Afterward, the *p*-value and false discovery rate (FDR) were calculated to detect the significance of expression deregulation in the *FRMD5* gene. The boxplots with the expression of *FRMD5* in both subsets were created using the ggplot2 package.

### 4.3. Cell Culture

The experiments were performed on cell lines derived from human papillary thyroid cancer, BCPAP bearing the *BRAF* V600E mutation, and TPC1 bearing *RET/PTC* rearrangement. BCPAP cells were purchased from the German Collection of Microorganisms. TPC1 cells were kindly provided by Prof. M. Santoro (The University of Naples Federico II, Naples, Italy) and were authenticated by short tandem repeat (STR) analysis at the American Type Culture Collection (ATCC), as already described [21]. Cells were propagated in Roswell Park Memorial Institute (RPMI) 1640 medium (Cat No. SH30027.FS, HyClone, Cytiva, Marlborough, MA, USA) supplemented with 10% fetal bovine serum (FBS; Cat No. SV30160.03, HyClone, Cytiva). Cells were cultured in a humidified incubator at 37 °C with 5% CO_2_.

### 4.4. Silencing of FRMD5 by Small Interfering RNA (siRNA)

The *FRMD5* gene was silenced by transfection with siRNA specifically targeting human FRMD5 (siFRMD5; siRNA ID: s39794, Silencer Select, ThermoFisher Scientific, Inc., Rockford, IL, USA) or MISSION siRNA Universal Negative Control (siNEG; Cat No. SIC001, Sigma-Aldrich, Steinheim, Germany), and Lipofectamine 2000 (Cat No. 11668019, ThermoFisher Scientific, Inc.) in Opti-MEM I Reduced Serum Medium (Cat No. 11058021, ThermoFisher Scientific, Inc.), as previously described [22]. The efficiency of *FRMD5*-knockdown was verified by RT-qPCR and Western blotting.

### 4.5. Total RNA Extraction, Reverse Transcription, and RT-qPCR

Forty-eight hours post-transfection, total RNA was isolated from cells using GeneMATRIX Universal RNA Purification Kit (Cat No. E3598, EURx, Gdansk, Poland) with the step of the on-column DNase I (Cat No. 1009-100, A&A Biotechnology, Gdynia, Poland) digestion according to the producer’s instructions. Five hundred nanograms of total RNA was reverse transcribed using a High-Capacity cDNA Reverse Transcription Kit with RNase Inhibitor (Cat No. 4374966, ThermoFisher Scientific, Inc.), following the recommended protocol. The expression of the genes was quantified by RT-qPCR using a 5x HOT FIREPol EvaGreen qPCR Mix Plus (Cat No. 08-25-00020, Solis BioDyne, Tartu, Estonia), 6-times diluted cDNA, and 0.5 µM of specific oligonucleotide primers (listed in Table 2). RT-qPCR was performed in the CFX96 Detection System (Bio-Rad, Hercules, CA, USA) with one cycle of 15 min at 95 °C, followed by 40 cycles of 15 s at 95 °C, 20 s at 58 °C, and 20 s at 72 °C. The expression of target genes was calculated using the 2^−ΔΔCt^ method and normalized to the *18S rRNA* reference gene.

### 4.6. RNA-Seq Library Preparation and Sequencing

The integrity of the extracted RNA was evaluated using the 2100 Bioanalyzer instrument (Agilent Technologies, Inc., St. Clara, CA, USA) and the Agilent RNA 6000 Nano Kit (Cat No. 5067-1511, Agilent Technologies, Inc.). Samples with RIN (RNA integrity number) values of 8 or greater were taken to construct a cDNA library. The Ion AmpliSeq Transcriptome Human Gene Expression Panel (Cat No. A31446, ThermoFisher Scientific, Inc.) was applied for the preparation of the library, following the manufacturer’s protocol [23]. In brief, cDNA libraries were prepared with the Ion AmpliSeq Transcriptome Human Gene Expression Panel (ThermoFisher Scientific, Inc.) according to the manufacturer’s protocol. RNA samples were reverse-transcribed, and cDNA libraries were generated and quantified on the Bioanalyzer 2100 with a High Sensitivity DNA Kit (Agilent Technologies, Inc.). Eight barcoded library templates (50 pM) were mixed and loaded onto Ion PI chips with Ion Chef Instrument and sequenced on an Ion Proton Sequencer (ThermoFisher Scientific, Inc.) with the Ion PI Hi-Q Chef Kit, according to the manufacturer’s instructions. Signal processing and base calling was conducted using the Ion Torrent Suite v5.10 (https://github.com/iontorrent/TS, accessed on 17 January 2020). The read-outs were mapped to the hg19 AmpliSeq Transcriptome v1 genome with tmap v5.10. Read counts per gene were obtained with HTSeq-count [24] v0.6, using default parameters. Normalization and differential gene expression estimations were performed using DESeq2 [25] v1.18.1, using default parameters and options. A gene was considered differentially expressed when the adjusted *p*-value was less than 0.05. Functional analysis of differentially expressed genes was conducted with the Cytoscape [26] (v3.7.2) plugin ClueGo [27] (v2.5.7), on a database consisting of Biological Process (BP) and Molecular Function (MF) categories in Gene Ontology [28] (GO, version 8 May 2020). Benjamini–Hochberg *p*-value adjustment was used, and terms with adjusted *p*-value less than 0.05 were considered significant. GO enrichment visualization was prepared with GOplot [29] R package v1.0.2, using a reduction of similar terms (90% for BCPAP and 70% for TPC1).

### 4.7. Total Protein Isolation and Western Blotting

Seventy-two hours after transfection, the cells were washed 3 times with chilled phosphate-buffered saline (PBS; pH 7.3) and lysed with RIPA Lysis and Extraction Buffer (Cat No. 89900, ThermoFisher Scientific, Inc.) supplemented with Pierce Phosphate Inhibitor Cocktail (Cat No. A32957, ThermoFisher Scientific, Inc.), Complete Protease Inhibitor Cocktail (Cat No. 4693116001, Roche, Basel, Switzerland), and Viscolase (Cat No. 1010-100, A&A Biotechnology) on ice for 30 min. Protein extraction from tissue samples was performed as described previously [30]. After one freeze-thaw cycle, the total protein concentration in the cell lysate was determined using the BCA Protein Assay Kit (Cat No. 23225, ThermoFisher Scientific, Inc.). The membrane was further processed as already described, with some minor modifications [31]. Twenty to thirty micrograms of the total protein lysate was resolved in 9% SDS-PAGE under reducing conditions and subsequently electro-transferred to a PVDF membrane (Cat No. IPVH00010, Merck Millipore, Tullagreen, Ireland). After one hour blocking in 5% skimmed milk in Tris-buffered saline (TBS) supplemented with 0.1% Tween 20 (TBST) at room temperature (RT) and extensive washing in TBST, the membrane was probed overnight at 4 °C with a primary antibody diluted in 5% skimmed milk-TBST or 5% BSA (Cat No. 7906, Sigma-Aldrich)-TBST (primary antibodies and blocking agents used in the study are listed in Table 3). After extensive washing, the membrane was incubated for 1 h at RT with secondary antibodies (1:5000 in 1% skimmed milk-TBST): goat anti-rabbit immunoglobulins/horseradish peroxidase (HRP; Cat No. P0448, Dako, Carpinteria, CA, USA), or goat anti-mouse immunoglobulins/HRP (Cat No. 115-035-146, Jackson ImmunoResearch Lab, Inc., West Grove, PA, USA). Then, the membrane was intensively washed, and signals from reactive bands were visualized using the SuperSignal West Dura Extended Duration Substrate (Cat No. 34076, ThermoFisher Scientific, Inc.) or SuperSignal West Pico PLUS Chemiluminescent Substrate (Cat No. 34577, ThermoFisher Scientific, Inc.).

### 4.8. Phospho-Kinase Proteome Profiling

The assay was performed using the Human Phospho-Kinase Array Kit (Cat No. ARY003C, R&D Systems), according to the manufacturer’s instructions. Seventy-two hours post-transfection, the cells were rinsed three times with chilled phosphate-buffered saline (PBS; pH 7.3) and lysed with Lysis Buffer 6 supplemented with Complete Protease Inhibitor Cocktail (Roche), Pierce Phosphate Inhibitor Cocktail (ThermoFisher Scientific, Inc.), and nuclease (Viscolase; A&A Biotechnology) on ice for 30 min. After a single freeze–thaw cycle, the total protein concentration in the cell lysate was determined using a BCA Protein Assay Kit (ThermoFisher Scientific, Inc.). The array membranes were blocked in Array Buffer 1 for 1 h and incubated overnight with 600 µg of total protein lysate in Array Buffer 1 at 4 °C. Then, they were probed with a Detection Antibody Cocktail for 2 h, followed by 30-min incubation with Streptavidin-HRP solution. After each step, the membranes were washed three times with Wash Buffer. The chemiluminescent signal was developed using the Chemi Reagent Mix and captured on the Mini HD9 acquisition system (Uvitec Ltd., Cambridge, UK). The results were confirmed by Western blot analysis.

### 4.9. Cell Migration and Matrigel Invasion Assays

The cell migration and invasion assays were determined using 8-μm pore non-coated (Cat No. 353097, Corning, Inc., Corning, New York, NY, USA) and Matrigel-coated (Cat No. 354480, Corning) inserts, respectively, as already described [16,22]. Briefly, 48-h after transfection, the harvested cells (2 × 10^5^) were added to the upper chambers in a serum-free medium and cultured for a further 24 h. Complete medium in the lower well was used as a chemoattractant. Cells that migrated or invaded through the membranes underwent fixation and staining using a RAL Diff-Quik kit (Cat No. 720555-0000, RAL Diagnostics, Martillac, France) and the cells were counted using Olympus BX41 microscope (Olympus Corporation, Tokyo, Japan) with a 40× objective lens.

### 4.10. In Vitro Wound Healing Motility Assay

A wound-healing assay was performed as already described [22]. Briefly, siRNA-treated cells were seeded on a 6-well plate and cultured until the formation of a nearly confluent monolayer. Then, a scratch wound was created using a sterile 200 μL pipette tip. Any cellular debris was removed by washing with Dulbecco’s phosphate-buffered saline without calcium and magnesium (D-PBS; Cat No. SH30028.FS, HyClone, Cytiva), and then growth medium was added. Images of the scratched areas were captured between 0 and 48 h using a light microscope (10× lens; AxioObserver D1, Carl Zeiss AG, Oberkochen, Germany) equipped with AxioVision LE software (Carl Zeiss AG). The cell migration distance was calculated by measuring the wound width, dividing it by two, and subtracting this value from the initial half-wound width. For the 10× lens, 1 pixel is equal to 1.026 µm.

### 4.11. Cell Adhesion Assay

The assay was performed using a colorimetric ECM Adhesion Array Kit (Cat No. ECM 540, Merck Millipore), following the manufacturer’s instructions. Seventy-two hours after *FRMD5* silencing, cells were detached with HyQTase Cell Detachment Solution (Cat No. SV30030.01, HyClone, Cytiva) and quenched with Dulbecco’s Modified Eagle Medium (DMEM; Cat No. 10-013-CV, Corning, Inc.) supplemented with 5% BSA. Then, 100 µL of cell suspension (1 × 10^6^/^mL^ in Assay Buffer) was added to each well. After a two-hour incubation, the plate was washed with Assay Buffer and stained with Cell Stain Solution for 5 min. The excess stain was removed by washing with deionized water, and the plate was air-dried. After the dye solubilization with Extraction Buffer for 10 min, the absorbance was determined using Synergy 2 Multi-Mode Microplate Reader (BioTek Instruments, Inc., Winooski, VT, USA) at 560 nm.

### 4.12. Cell Proliferation Assay

The assay was performed using BrdU-based colorimetric Cell Proliferation ELISA (Cat No. 11647229001, Roche) following the manufacturer’s protocol. Five thousand siRNA-transfected cells in a final volume of 100 µL were seeded in 96-well plates (8 replicates). Forty-eight hours later, 10 µL of BrdU labeling solution was added to each well, and the incubation was continued for an extra 7 h. The culture medium was removed, cells were fixed, and DNA was denatured by adding FixDenat. Then, cells were incubated with a BrdU-specific antibody for 90 min, and unbound antibody conjugates were removed in three washing cycles. After 15-min of incubation with tetramethylbenzidine (TMB) substrate, the absorbance was determined using the Synergy 2 Multi-Mode Microplate Reader (BioTek Instruments, Inc.) at 370 nm with a reference wavelength of 492 nm and finally expressed as the percentage of control (siNEG-treated cells).

### 4.13. Cell Viability Assay (MTS-Based Assay)

The assay was performed using the MTS CellTiter 96 AQueous Non-Radioactive Cell Proliferation Assay (MTS; Cat No. G3581, Promega Corporation, Madison, WI, USA) according to the manufacturer’s protocol. Five thousand siRNA-transfected cells in a final volume of 100 µL were seeded in 96-well plates (12 replicates). After 48 h, 20 µL of MTS reagent was added to each well, and the incubation was continued for an additional 3 h. In some experiments, 48 h post-transfection, the culture medium was supplemented with doxorubicin (Cat No. T1020, TargetMol, Boston, MA, USA), at a final concentration of 10 µM and MTS-based cell viability assay was performed 24 h later. The absorbance readings were recorded at 490 nm with a reference wavelength of 650 nm using the Synergy 2 Multi-Mode Microplate Reader (BioTek Instruments, Inc.). The data are expressed as the percent of control (siNEG-treated cells).

### 4.14. Cell Viability Assay (Trypan Blue-Based Assay)

Seventy-two hours after transfection, the trypan blue exclusion assay was performed, as described elsewhere [32,33]. Briefly, all cells (both attached and unattached) were harvested, centrifuged, resuspended in D-PBS (HyClone, Cytiva), and stained with trypan blue dye (NanoEnTek, Inc., Seoul, Korea) at a final concentration of 0.2%. The number of total and viable cells was determined using an automated cell counter (EVE; NanoEnTek, Inc.). Results are expressed as the percent of viable cells.

### 4.15. Cell Viability Assay (Annexin V/Propidium Iodide-Based Flow Cytometry)

The viability of siRNA-treated cells (for 72 h) using the FITC Annexin V Apoptosis Detection Kit I (Cat No. 556547, BD Biosciences, San Diego, CA, USA) was performed, as suggested by the manufacturer. Briefly, all cells were mixed, washed with D-PBS (HyClone, Cytiva), resuspended in Binding Buffer at a final concentration of 10^6^ cells/mL. Afterward, 100 µL (1 × 10^5^) of cell suspension was probed with 5 µL of FITC-conjugated Annexin V and 5 µL of propidium iodide for 15 min protected from light. Then, 400 µL of Binding Buffer was added to each tube, and cells were analyzed by flow cytometry using a BD Accuri C6 Plus flow cytometer and dedicated BD Biosciences software (v.1.0.23.1; BD Biosciences).

### 4.16. Confocal Microscopy

The transfected cells (1 × 10^5^) were seeded in 6-well plates containing uncoated cover glasses in a final volume of 2 mL. Forty-eight hours later, the culture medium was supplemented with doxorubicin (Cat No. T1020, TargetMol) at a final concentration of 10 µM, and the incubation was continued for a further 17 h. Next, the cells were fixed with 4% paraformaldehyde (Cat. No. 6148, Sigma-Aldrich) in PBS, as already described [31], followed by permeabilization with 0.25% Triton X-100 (Cat No. X-100, Sigma-Aldrich) in deionized water for 3 min. After washing with PBS, the cells were blocked with 2% BSA (Cat No. 7906, Sigma-Aldrich) in TBST for 1 h and stained with phalloidin conjugated with FITC (2 µg/mL in PBS; Cat No. P5282-.1MG, Sigma-Aldrich) and 4′,6-diamidino-2-phenylindole dihydrochloride (DAPI; 0.4 µg/mL in deionized water; Cat No. P5282-.1MG, Sigma-Aldrich) for 30 min and 2 min, respectively. After a final wash with PBS and mounting using Fluorescence Mounting Medium (Cat No. S3023, Dako), the cells were examined using the Zeiss LSM800 confocal unit supplied with a plan-apochromatic 63x/1.4 oil DIC M27 lens (Carl Zeiss AG), as previously described [31].

### 4.17. Spheroid Formation Assay

Forty-eight hours after transfection, 1 × 10^4^ of 0.05% trypsin-EDTA (HyClone, Cytiva)-detached cells in a final volume of 100 µL were seeded in Costar ultra-low attachment 96-well round-bottom plates (Cat No. 7007, Corning, Inc.) in complete medium (12 replicates). Spheroid formation was monitored and visualized using AxioObserver D1 microscope (10× lens; Carl Zeiss AG) and AxioVision LE software (Carl Zeiss AG) as previously described [32]. The area of spheroids was calculated using ImageJ (NIH) software. The results are presented as the percentage of spheroid area compared to controls (siNEG-treated cells).

### 4.18. Statistical Analysis

All experiments were performed at least three times. Quantitative data are expressed as mean ± standard deviation (SD). The data were analyzed using GraphPad Prism 6.0 for Windows (GraphPad, Inc., San Diego, CA, USA). For statistical purposes, nonparametric Mann–Whitney U test and one-way ANOVA followed by Bonferroni posthoc comparative test or *t*-student test were used. Statistical significance was considered at *p*-values < 0.05.

## Figures and Tables

**Table 1:** List of differentially expressed representative transcripts in RNA-Seq analysis of siFRMD5-treated TPC1 and BCPAP cells validated by RT-qPCR.

Gene	RNA-Seq				RT-qPCR			
	TPC1		BCPAP		TPC1		BCPAP	
	*p* Value	Fold Change	*p* Value	Fold Change	*p* Value	Fold Change	*p* Value	Fold Change
*VEGFA*	<0.001	0.379	0.016	0.634	<0.001	0.350	0.003	0.632
*STAT3*	0.105	1.418	0.001	1.905	0.605	1.077	0.036	2.036
*MTOR*	0.168	1.457	0.006	1.971	<0.001	1.313	0.041	1.186
*SOX9*	<0.001	0.350	0.409	1.233	<0.001	0.369	0.008	1.189
*MMP1*	0.672	1.138	<0.001	2.845	0.016	1.158	<0.001	4.737
*MMP9*	0.042	0.537	<0.001	3.212	<0.001	0.565	<0.001	2.999
*TIMP1*	0.703	0.921	0.037	1.420	0.189	0.885	0.001	1.593
*ITGA2*	0.124	0.702	0.004	1.815	0.002	0.585	0.002	2.149
*EPHA2*	0.019	0.711	<0.001	1.987	0.001	0.600	0.002	1.652
*HAS2*	0.017	0.585	0.014	1.589	<0.001	0.368	0.046	1.379
*ITGA6*	0.620	0.873	<0.001	2.419	0.002	0.585	0.002	2.149
*PDPN*	0.024	0.678	<0.001	1.976	0.007	0.703	<0.001	2.389

**Table 2:** List of primers used in RT-qPCR.

Gene Name	Nucleotide Sequences (5′→3′)
*ABCB1*	F: CAGGAACCTGTATTGTTTGCCACCAC R: TGCTTCTGCCCACCACTCAACTG
*ABCC1*	F: TGTGGGAAAACACATCTTTGA R: CTGTGCGTGACCAAGATCC
*ABCC6*	F: TGTCGCTCTTTGGAAAATCC R: AGGAACACTGCGAAGCTCAT
*ABCG2*	F: GGTGGAGGCAAATCTTCGTTATTAGA R: GAGTGCCCATCACAACATCATCTT
*FRMD5*	F: ATCAAAAGGGATCTCTACCATG R: ATCTCCGCTTGAAGGATGTA
*HAS2*	F: TGACAGGCATCTCACGAACC R: CAGCCATTCTCGGAAGTAGG
*ITGA2*	F: CTCACCAGGAACATGGGAAC R: GTCAGAACACACACCCGTTG
*ITGA6*	F: ATGCACGCGGATCGAGTTT R: TTCCTGCTTCGTATTAACATGCT
*MMP1*	F: CTGGCCACAACTGCCAAATG R: CTGTCCCTGAACAGCCCAGTACTTA
*MMP9*	F: GCACGACGTCTTCCAGTACC R: CAGGATGTCATAGGTCACGTAGC
*MTOR*	F: GACGAGAGATCATCCGCCAG R: ACAAGGGACCGCACCATAAG
*PDPN*	F: CGAAGATGATGTGGTGACTC R: CGATGCGAATGCCTGTTAC
*SOX9*	F: CGTGGACATCGGTGAACTGA R: GGTGGCAAGTATTGGTCAAACTC
*STAT3*	F: CCACCACCAAGCGAGGAC R: GCCAGACCCAGAAGGAGAAG
*TIMP1*	F: AAGGCTCTGAAAAGGGCTTC R: GAAAGATGGGAGTGGGAACA
*VEGFA*	F: CTTGCCTTGCTGCTCTACCT R: AAGATGTCCACCAGGGTCTC
*18S rRNA*	F: CCAGTAAGTGCGGGTCATAAG R: CCATCCAATCGGTAGTAGCG

F, forward; R, reverse.

**Table 3:** Primary antibodies used in the study for Western blotting.

Antigen	Catalog No.	Type/ Clone (Symbol)	Dilution/ Blocking Agent	Source
β-actin	3700	Mouse monoclonal (IgG2b)/ 8H10D10	1:5000/ 5% skimmed milk	Cell Signaling Technology, Inc. (Beverly, MA, USA)
pERK1/2 (T202/Y204)	4370	Rabbit monoclonal/ D13.14.4E	1:2000/ 5% BSA	Cell Signaling Technology, Inc.
tERK1/2	9102	Rabbit polyclonal	1:2000/ 5% BSA	Cell Signaling Technology, Inc.
P-gp	sc-13131	Mouse monoclonal (IgG2b)/ G-1	1:200/ 5% skimmed milk	Santa Cruz Biotechnology, Inc. (Santa Cruz, CA, USA)
pPRAS40 (T246)	13175	Rabbit monoclonal/ D4D2	1:2000/ 5% BSA	Cell Signaling Technology, Inc.
tPRAS40	sc-517549	Mouse monoclonal (IgG1)/ 73P21	1:200/ 5% skimmed milk	Santa Cruz Biotechnology, Inc.
pSRC (Y416)	2101	Rabbit polyclonal	1:1000/ 5% BSA	Cell Signaling Technology, Inc.
tSRC	2110	Mouse monoclonal (IgG1)/ L4A1	1:1000/ 5% skimmed milk	Cell Signaling Technology, Inc.
pSTAT3 (Y705)	4113	Mouse monoclonal (IgG1)/ M9C6	1:2000/ 5% skimmed milk	Cell Signaling Technology, Inc.
pSTAT3 (S727)	94994	Rabbit monoclonal/ D8C2Z	1:1000/ 5% BSA	Cell Signaling Technology, Inc.
tSTAT3	sc-8019	Mouse monoclonal (IgG1)/ F-2	1:200/ 5% skimmed milk	Santa Cruz Biotechnology, Inc.

BSA, bovine serum albumin; p, phosphorylated protein; t, total protein.

## Data Availability

The data presented in this study are openly available in the European Nucleotide Archive at https://www.ebi.ac.uk/ena/browser/home/, accession number: PRJEB45791.

## Supplementary Materials

The following are available online at https://www.mdpi.com/article/10.3390/ijms22136726/s1.
