# Supplementary material for: Analysis of the Role of FRMD5 in the Biology of Papillary Thyroid Carcinoma
Source: Int J Mol Sci. 2021 Jun 23;22(13):6726. doi: 10.3390/ijms22136726 (PMC8268710; doi:10.3390/ijms22136726)

Figure S1. Representative cytograms of flow cytometer-based apoptosis analysis of TPC1 and BCPAP cells treated with siFRMD5 or siNEG (control). Data show the percentage of viable, early apoptotic (E) and late apoptotic (L) or necrotic (N) cells.

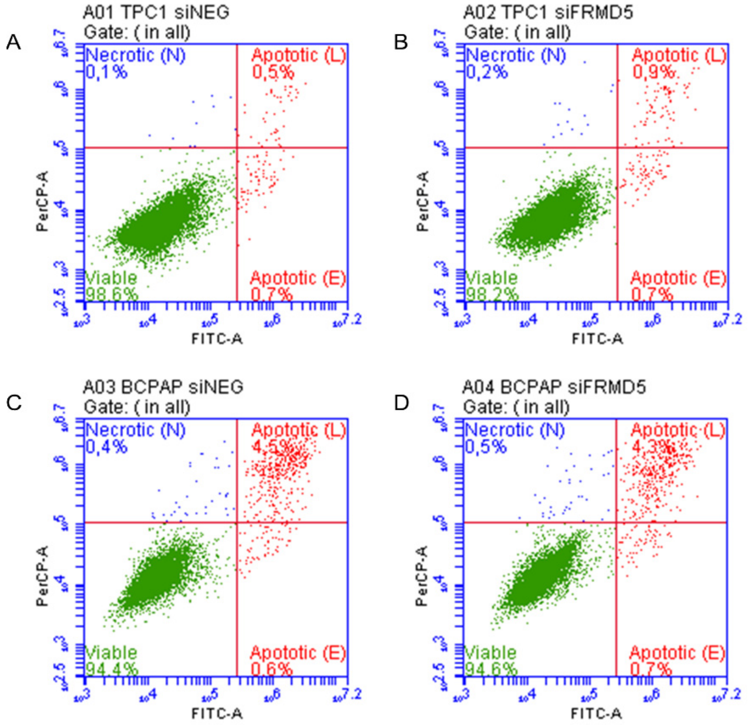

Supplement: Supplementary file 1 [file ijms-22-06726-s001.zip › Figure S1.pdf]
