# Supplementary material for: Analysis of the Role of FRMD5 in the Biology of Papillary Thyroid Carcinoma
Source: Int J Mol Sci. 2021 Jun 23;22(13):6726. doi: 10.3390/ijms22136726 (PMC8268710; doi:10.3390/ijms22136726)

Figure S2. Summary of human phospho-kinase array analysis performed on FRMD5-knocked-down TPC1 and BCPAP cell lines. Cells were transfected with FRMD5-specific siRNA (siFRMD5) or non-targeting siRNA (siNEG; control). Graphical data are presented as mean ± SD.

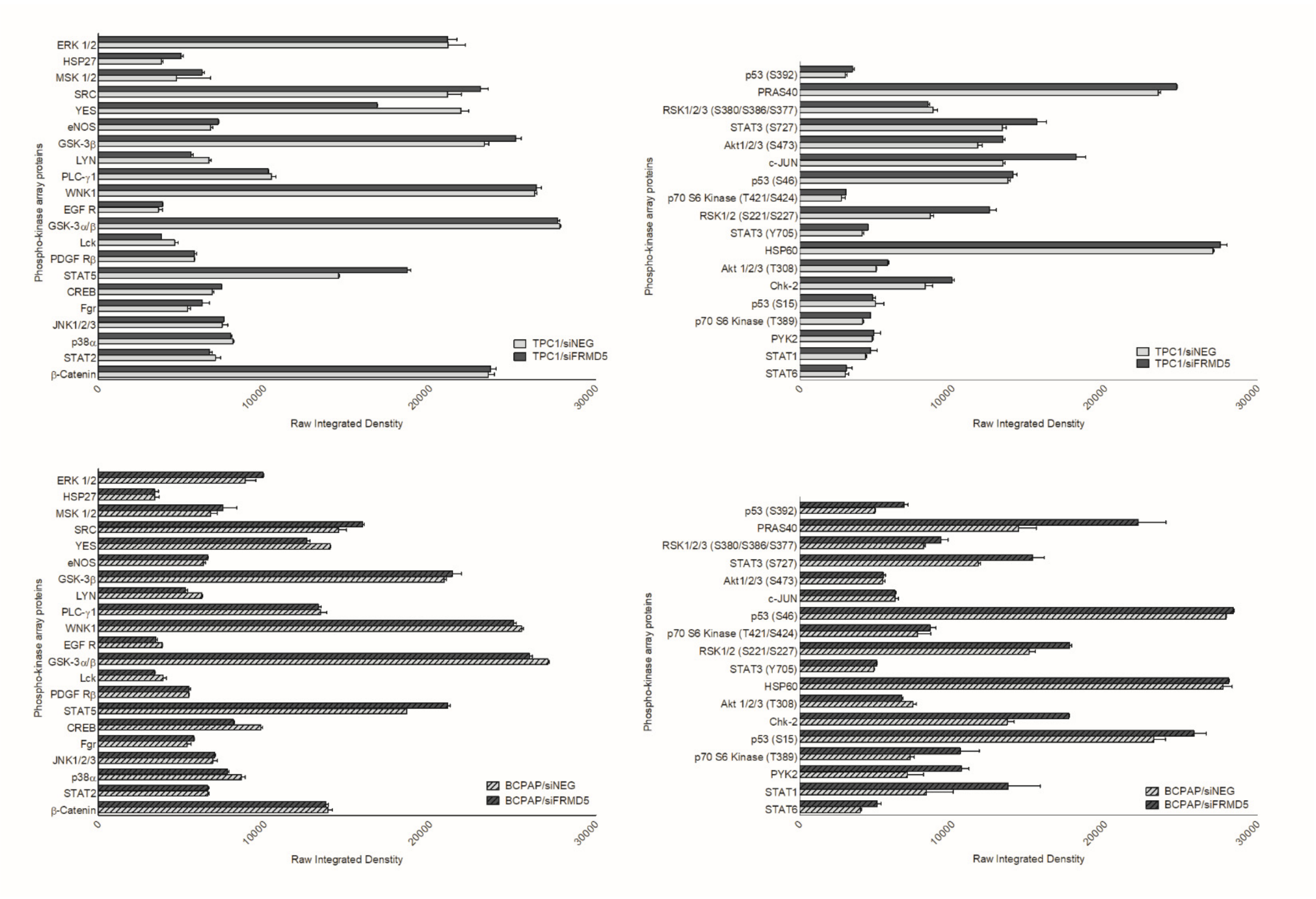

Supplement: Supplementary file 1 [file ijms-22-06726-s001.zip › Figure S2.pdf]
