# Supplementary material for: Analysis of the Role of FRMD5 in the Biology of Papillary Thyroid Carcinoma
Source: Int J Mol Sci. 2021 Jun 23;22(13):6726. doi: 10.3390/ijms22136726 (PMC8268710; doi:10.3390/ijms22136726)
Supplement: Supplementary file 1 [file ijms-22-06726-s001.zip › Supplementary Table S1.pdf]

Supplementary Table S1. Genes upregulated in TPC1 siFRMD5 (in comparison with TPC1 siNEG).

gene	TPC1 siNEG 1	TPC1 siNEG 2	TPC1 siNEG 3	TPC1 siFRMD5 1	TPC1 siFRMD5 2	TPC1 siFRMD5 3	baseMean	log2FoldChange	lfcSE	stat	pvalue	padj	Fold-change
ANXA2	6946,955331	7694,709845	8070,733485	29182,39744	33450,37831	26534,48526	14085,86744	1,972926093	0,150180308	13,13704922	2,01949E-39	2,2687E-35	3,925635146
GALNT7	708,025101	706,0935042	777,030009	1690,146684	1758,420035	1630,501492	767,8733723	1,212479562	0,126807172	9,561600852	1,15949E-21	3,25643E-18	2,317355789
CD2AP	1322,570115	1334,608765	1260,454654	3093,99094	3511,229285	3899,503942	1672,570136	1,423040004	0,155165371	9,171118498	4,68136E-20	8,76507E-17	2,681499536
CLDN12	95,2111994	92,04198379	84,66759324	380,1326349	374,8004414	311,9113881	228,1662748	1,974563893	0,220890625	8,939102276	3,92345E-19	5,5095E-16	3,930094199
SETD7	311,6002889	327,4064852	333,2079476	891,3869699	862,9387407	941,8500739	640,8412132	1,470453403	0,165643797	8,877201724	6,85634E-19	7,00219E-16	2,771089685
ZC3HAV1L	19,04223988	34,18702255	39,60258394	253,8227404	221,064931	194,4859243	67,82308783	2,827712671	0,318485559	8,878621297	6,76942E-19	7,00219E-16	7,099476615
TFPI	765,1518206	845,4713654	786,5892534	1746,685399	1949,186726	2206,620173	1128,357036	1,299320433	0,156997439	8,276061331	1,27316E-16	1,19189E-13	2,461129263
RBM8A	1718,994927	1857,933187	1928,236156	3985,37791	4674,906104	5659,662717	2818,5264	1,378865323	0,174774328	7,889404229	3,03633E-15	2,49807E-12	2,60063751
UBE2V2	971,1542339	1376,6851	1104,775531	2875,05379	3239,667288	2621,278842	1853,02205	1,338058467	0,169669033	7,886285709	3,11313E-15	2,49807E-12	2,52810865
ELL2	955,5742194	1268,864491	998,2582365	2408,308656	2383,461489	2681,214756	1441,608305	1,212180857	0,159131675	7,61747062	2,58695E-14	1,93745E-11	2,316876039
LCLAT1	128,102341	207,7519063	118,8077518	522,0808973	528,5359517	563,8868624	356,9871129	1,822939895	0,239748429	7,603553035	2,88109E-14	2,02289E-11	3,538014338
ITGB8	31,16002889	35,50190803	73,7427425	161,1954844	209,8433609	218,9495626	435,496939	2,055611686	0,273269254	7,522294052	5,38234E-14	3,55677E-11	4,157198643
PAWR	79,63118495	173,5648837	196,6473133	537,7192652	574,5443892	606,6982294	371,984813	1,923169504	0,257132885	7,479282564	7,47294E-14	4,66395E-11	3,792553426
ARSJ	58,85783236	77,57824348	83,3019869	246,6050322	219,942774	324,1432072	268,4491754	1,837469043	0,248701589	7,388248104	1,48776E-13	8,35674E-11	3,573825128
CAV2	1355,461257	1425,335863	1384,724831	3405,555347	4233,898399	5189,960862	1958,901565	1,622776265	0,225265871	7,203826546	5,85458E-13	3,0574E-10	3,07967106
HMGA2	2560,315707	1806,652653	1410,671352	4983,827552	6710,49892	6599,066427	4877,473187	1,663579024	0,231032625	7,200623828	5,99377E-13	3,0574E-10	3,168014687
PLAC8	62,32005779	85,46755638	106,5172947	334,4204826	309,7153348	286,2245679	100,2905725	1,861754148	0,26003188	7,159714974	8,0845E-13	3,78422E-10	3,634493054
CXADR	110,7912138	180,1393111	147,485485	433,0624955	526,2916377	599,3591379	166,5607224	1,82365635	0,256813979	7,101078995	1,23787E-12	5,34853E-10	3,539771784
EIF4EBP2	941,7253177	1033,499989	1051,516884	2411,91751	2622,480933	3647,528468	1541,989431	1,519835738	0,217653066	6,982836349	2,89279E-12	1,08325E-09	2,86758398
AKIRIN1	536,6449421	394,4656448	419,2411472	1065,814919	1336,488999	1283,117828	1007,416904	1,450996674	0,211981411	6,844924122	7,65164E-12	2,6048E-09	2,7339686
PLAGL1	320,2558525	287,9599207	241,7123226	637,5642295	879,7710959	917,3864356	285,1056414	1,520439463	0,226244213	6,720346318	1,81293E-11	5,81899E-09	2,868784231
APOL1	467,4004334	595,6431237	487,5214643	976,7965176	1158,066034	1051,936446	522,763663	1,037394835	0,15658831	6,62498262	3,47289E-11	1,08373E-08	2,052517944
MAMDC2	67,51339594	139,3778612	143,388666	421,0329817	356,8459292	512,513222	196,6026473	1,870401448	0,283428052	6,59921076	4,13352E-11	1,20075E-08	3,656343084
PON2	167,9179335	165,6755708	234,8842909	455,9185716	504,9706545	519,8523135	243,0489077	1,378774695	0,215024648	6,412170463	1,43462E-10	3,74804E-08	2,600474147
PIK3R1	72,70673409	142,0076321	195,281707	446,2949606	393,8771105	458,6932178	334,3870225	1,652107741	0,259168255	6,374653181	1,83377E-10	4,57792E-08	3,142924772
ZNF652	173,1112716	156,4713724	185,7224626	359,6824615	449,984961	371,8473019	262,1077073	1,198529255	0,190493025	6,291722519	3,13962E-10	7,34803E-08	2,29505584
DCP2	200,8090751	222,2156466	262,1964178	981,6083231	1037,995234	627,4923219	485,860211	1,948705863	0,312018443	6,245482944	4,22493E-10	9,68629E-08	3,860280983
RECK	19,04223988	32,87213707	20,48409514	134,7305542	112,215701	133,3268286	48,56974882	2,378575031	0,383187006	6,207347837	5,38862E-10	1,21072E-07	5,200228551
CPOX	76,16895952	61,79961769	75,10834885	203,2987826	164,9570805	232,4045637	140,3782916	1,498371029	0,243669241	6,149200543	7,78745E-10	1,65064E-07	2,825235302
UHMK1	292,5580491	311,6278594	397,3914457	773,497735	819,1746174	978,5455313	541,1150542	1,358467066	0,221680698	6,128034946	8,8971E-10	1,85093E-07	2,564125838
YOD1	228,5068786	152,526716	120,1733582	485,9923561	471,3059442	642,1705049	295,7553129	1,680667134	0,276516626	6,077996675	1,21693E-09	2,2785E-07	3,205761582
DENND1B	115,984552	111,765266	116,0765391	239,3873239	249,1188562	300,9027509	124,5372784	1,199265375	0,198901817	6,029433979	1,64535E-09	2,98127E-07	2,296227165
GFRA1	344,4914306	273,4961804	262,1964178	698,9147497	687,8822472	634,8314134	241,8177033	1,203290078	0,199841032	6,021236312	1,7309E-09	3,08649E-07	2,30264192
C12orf73	27,69780346	34,18702255	28,6777332	117,8892349	86,40608978	97,85455313	112,7878032	1,72985506	0,289088211	5,98383122	2,17949E-09	3,70976E-07	3,316944929
TMEM237	110,7912138	166,9904563	203,4753451	441,4831551	453,3514321	527,191405	363,4072375	1,556533748	0,261474856	5,952900287	2,63432E-09	4,417E-07	2,941462713
GALNT1	406,8114883	344,4999965	438,359636	868,5308937	717,0583294	980,9918951	516,2749932	1,109653601	0,186560544	5,947954361	2,71514E-09	4,48558E-07	2,157938279
DPY19L3	143,6823555	190,658395	191,184888	446,2949606	379,2890694	549,2086794	219,7371262	1,382356803	0,2325238	5,945012095	2,76436E-09	4,48571E-07	2,60693896
RASA2	76,16895952	69,68893058	91,49562496	242,996178	198,6217908	214,056835	152,1862722	1,464739826	0,247877477	5,909128345	3,43923E-09	5,36615E-07	2,760136901
MT1E	2742,082543	2435,167914	1996,516473	4667,45134	4438,130975	5050,518123	2098,755644	0,981112207	0,166662479	5,886821152	3,93694E-09	6,05858E-07	1,973986614
CRNKL1	3,462225433	36,81679352	46,43061565	159,9925331	125,6815851	121,0950095	97,86538189	2,186962833	0,371736607	5,883097849	4,02658E-09	6,11278E-07	4,553458807
EIF5A2	160,9934826	165,6755708	170,7007928	359,6824615	295,1272936	402,4268497	231,7421017	1,085858965	0,186204554	5,831538181	5,49187E-09	7,90971E-07	2,122638903
NUFIP2	493,3671242	704,7786187	736,0618187	1441,135749	1159,188191	1546,101939	771,4501338	1,09735936	0,188096318	5,834028935	5,41047E-09	7,90971E-07	2,139627067
GCC2	207,733526	282,7003788	288,1429383	467,9480854	507,2149686	514,9595858	432,9467502	0,929637201	0,159759471	5,818980211	5,92077E-09	8,41949E-07	1,90479693
ARL5B	259,6669075	376,0572481	375,5417442	720,5678745	738,3793126	622,5995943	434,1035868	1,036683207	0,180963612	5,728683208	1,01213E-08	1,33768E-06	2,051505763
PHTF2	126,3712283	149,896945	222,5938339	440,2802038	438,7633909	669,080507	230,1930142	1,629715292	0,285037971	5,717537505	1,08079E-08	1,39558E-06	3,094519239
FAM46A	131,5645664	153,8416015	137,9262406	295,9260386	366,9453423	270,323203	237,5979604	1,138380488	0,199317412	5,711395097	1,12054E-08	1,4144E-06	2,20133771
ANXA3	759,9584825	976,9599137	801,6109231	1692,552587	1500,323922	1604,814671	897,5267277	0,916945911	0,161567626	5,675307192	1,3844E-08	1,70905E-06	1,888114049
HIST1H2BI	704,5628756	749,4847251	651,3942254	1211,372036	1522,767063	1341,83056	738,6246163	0,953131866	0,167910718	5,676420629	1,37542E-08	1,70905E-06	1,936071007

RP2	377,3825722	355,0190803	344,1327983	624,3317643	647,4845948	775,4973336	418,5405529	0,928316769	0,163650398	5,672560395	1,40679E-08	1,71781E-06	1,903054355
C4orf29	77,90007224	90,72709831	80,57077422	190,0663175	206,4768899	161,4600127	140,7596026	1,159942039	0,206026552	5,63006092	1,80146E-08	2,15294E-06	2,234484503
SAA1	20,7733526	26,29770965	42,33379662	97,43906149	176,1786506	171,245468	142,9164855	2,304157252	0,413117482	5,577486676	2,44018E-08	2,88558E-06	4,93878872
JAK1	858,6319073	940,1431201	1003,720662	1599,925331	2025,493403	1681,875132	1943,063852	0,920689647	0,165681678	5,556979242	2,74483E-08	3,21203E-06	1,893019991
ZMYM2	154,0690318	206,4370208	281,3149066	476,368745	471,3059442	477,0409465	286,5131198	1,143737637	0,206372469	5,542103757	2,98859E-08	3,46122E-06	2,209527117
FOXE1	138,4890173	178,8244256	143,388666	288,7083303	454,4735891	340,0445721	365,0168686	1,23083353	0,222963524	5,520335834	3,38352E-08	3,87862E-06	2,347025521
MTMR9	53,66449421	77,57824348	57,35546639	155,1807276	171,6900225	173,6918318	126,2017616	1,399511862	0,254545141	5,498089092	3,83929E-08	4,35662E-06	2,638123058
OPTN	517,6027022	564,0858721	633,641343	970,7817607	1035,75092	1010,348261	532,922359	0,812953321	0,148340224	5,480329605	4,24534E-08	4,76922E-06	1,756804094
PGM2L1	69,24450865	60,4847322	64,1834981	192,4722202	161,5906095	187,1468329	76,85184834	1,483702635	0,271872769	5,457341829	4,83315E-08	5,37581E-06	2,796655667
OAT	685,5206357	821,8034267	690,9968094	1260,693042	1439,727444	1752,819683	857,4109048	1,017625095	0,18752953	5,4264792	5,74765E-08	6,20857E-06	2,024583429
RUFY2	167,9179335	170,9351128	210,3033768	387,3503432	331,036318	451,3541263	245,7170272	1,088050827	0,201491802	5,399975663	6,66499E-08	7,06364E-06	2,125866243
KRT7	112,5223266	61,79961769	69,64592348	205,7046854	209,8433609	272,7695669	3083,894417	1,51007438	0,280171218	5,389826947	7,05256E-08	7,40453E-06	2,848247233
NFIB	417,1981646	550,9370173	487,5214643	946,7227332	890,992666	1067,837811	369,0885854	0,994749541	0,185539627	5,361385907	8,25859E-08	8,51165E-06	1,992734547
UBE2B	341,0292051	370,7977061	447,9188804	694,1029442	839,3734435	851,3346122	736,6131915	1,038371024	0,194226229	5,34619361	8,98231E-08	9,17339E-06	2,053907236
MYLK	334,1047543	261,6622111	223,9594402	487,1953074	694,6151892	687,4282357	427,088995	1,19325259	0,22357921	5,337046265	9,44729E-08	9,47597E-06	2,286676992
NIPAL3	254,4735693	372,1125916	255,3683861	535,3133625	637,3851817	618,9300485	349,9593231	1,019011339	0,191103624	5,332244965	9,7006E-08	9,64394E-06	2,026529728
BRWD1	604,158338	525,9541931	501,1775278	855,2984286	912,3136492	954,081893	652,6718832	0,740983344	0,139044575	5,329106472	9,86971E-08	9,68591E-06	1,671314622
GNPNAT1	1088,869899	1150,524797	1103,409925	2549,053967	3016,358043	1932,627424	1579,318414	1,165451563	0,219509481	5,309344985	1,1002E-07	1,04743E-05	2,243034115
LEPREL1	36,35336704	31,55725159	58,72107273	121,498089	138,0253122	181,0309233	75,92420072	1,794076211	0,338678293	5,29728728	1,17536E-07	1,09124E-05	3,467933444
CCNT2	121,1778901	181,4541966	187,0880689	374,1178781	449,984961	514,9595858	242,4250094	1,446089598	0,273555361	5,286277672	1,2483E-07	1,13605E-05	2,724685279
KCNJ16	39,81559248	48,65076286	75,10834885	162,3984358	180,6672786	172,4686499	56,66724539	1,646553121	0,311612347	5,283979073	1,26408E-07	1,13605E-05	3,130847245
TULP3	406,8114883	495,711827	439,7252423	1011,682108	1026,773664	1283,117828	676,2442686	1,306207975	0,24715953	5,284878052	1,25789E-07	1,13605E-05	2,472906983
COL12A1	574,7294218	594,3282382	660,9534698	1004,464399	1071,659945	1049,490082	1507,889938	0,771409012	0,147033585	5,246481681	1,55031E-07	1,33971E-05	1,706936053
INPP5A	154,0690318	169,6202273	154,3135167	413,8152735	374,8004414	281,3318402	233,2916555	1,161614531	0,223380219	5,200167383	1,99109E-07	1,65688E-05	2,237076405
PLS3	3891,541386	3640,917902	4176,024196	5709,207232	6017,005888	5755,070906	3572,68714	0,578316458	0,111194967	5,200922967	1,98301E-07	1,65688E-05	1,493105863
TBC1D5	152,337919	173,5648837	222,5938339	319,9850661	394,9992675	421,9977604	265,9201753	1,0470565	0,202079377	5,181411968	2,20213E-07	1,81902E-05	2,066309697
TRAPPC11	20,7733526	27,61259514	54,62425371	108,2656239	100,9941309	117,4254638	60,59173348	1,645006942	0,318447832	5,165703074	2,39537E-07	1,9642E-05	3,127493622
TDG	302,9447254	326,0915997	345,4984047	556,9664873	644,1181238	626,26914	477,4781151	0,905602846	0,176188407	5,139968413	2,74785E-07	2,23691E-05	1,873327122
KRT80	406,8114883	326,0915997	181,6256436	661,623257	738,3793126	867,2359771	1147,395958	1,313564506	0,256290881	5,125287728	2,97084E-07	2,38388E-05	2,485548934
SSH2	261,3980202	330,0362562	344,1327983	607,4904451	737,2571556	968,760076	363,5880476	1,304091844	0,254866013	5,116774205	3,10805E-07	2,44167E-05	2,469282406
ARRDC3	38,08447976	53,91030479	62,81789176	105,8597211	159,3462954	144,3354659	93,65473635	1,390891768	0,272577041	5,102747329	3,34758E-07	2,57371E-05	2,622407286
SPP1	855,1696819	710,0381607	868,5256339	1648,043386	2088,334196	1791,961504	676,0097717	1,184144519	0,232111953	5,101609398	3,36777E-07	2,57371E-05	2,272286139
PLCB4	114,2534393	234,0496159	279,9493002	591,8520772	401,7322096	618,9300485	682,8397405	1,352396769	0,269493545	5,018290017	5,21334E-07	3,82789E-05	2,553359664
TOP2A	1919,804002	2049,906468	2184,970148	2962,86924	3153,261198	3141,131155	2641,827977	0,588386612	0,11764761	5,001262758	5,6956E-07	4,10156E-05	1,503564348
OSTM1	115,984552	265,6068675	234,8842909	513,6602377	504,9706545	570,002772	306,0912466	1,358450647	0,272981516	4,976346622	6,47956E-07	4,54306E-05	2,564096656
TNFRSF19	12,11778901	22,35305321	25,94652051	60,14756882	74,06236266	75,83727868	33,06520219	1,77035699	0,357852044	4,947175852	7,52979E-07	5,18955E-05	3,411383597
CYP51A1	761,6895952	816,5438848	970,9461096	1373,770472	1577,752756	1522,861483	920,1379684	0,810717007	0,163945316	4,945045254	7,61262E-07	5,21464E-05	1,75408299
AOX1	844,7830056	752,1144961	680,0719586	1254,678286	1273,648206	1641,510129	600,2844388	0,873849324	0,176915327	4,939364725	7,83775E-07	5,33632E-05	1,832545887
ATP6V1B2	1345,074581	1719,870211	1988,322835	3097,599794	2935,562738	3454,265726	1855,248974	0,907890209	0,183929548	4,93607589	7,97101E-07	5,39436E-05	1,876299599
CSNK1A1	2236,59763	2365,478983	2590,555232	3750,802392	4135,148582	4317,832157	4353,800378	0,762463898	0,154752185	4,926999231	8,35021E-07	5,5837E-05	1,696385313
ARHGEF28	268,322471	256,4026691	247,174748	449,9038148	494,8712414	640,947323	571,5414026	1,039231242	0,211105216	4,922811757	8,53095E-07	5,67081E-05	2,055132258
DKK1	27,69780346	42,07633545	20,48409514	87,81545048	85,28393277	101,5240989	200,8479562	1,596399459	0,327547621	4,873793482	1,09475E-06	7,02928E-05	3,023877003
EPDR1	458,7448698	487,8225141	427,4347853	917,8519002	1123,279167	1305,135102	394,7430064	1,284086147	0,263469899	4,873748958	1,095E-06	7,02928E-05	2,435277449
SAMD4A	96,94231212	103,8759531	95,59244399	223,748956	265,9512114	362,0618466	136,0112418	1,52196723	0,312247866	4,874227812	1,09235E-06	7,02928E-05	2,871823789
LIPA	316,7936271	297,1641191	387,8322013	696,5088469	684,5157761	764,4886963	531,9532656	1,09816605	0,225818991	4,863036734	1,15598E-06	7,30279E-05	2,140823786
SERINC2	2420,095577	2270,807229	1868,149477	3810,94996	3855,731487	3459,158453	1994,464184	0,762840933	0,157146728	4,854322715	1,20799E-06	7,58131E-05	1,696828706
PPP1CC	2392,397774	2537,728982	2429,413684	3827,79128	4672,66179	5652,323625	3604,543207	0,943303039	0,195084029	4,835367846	1,329E-06	8,20327E-05	1,922925727
TROVE2	212,9268641	380,0019045	296,3365764	803,5715194	766,4332379	611,5909571	422,884233	1,291268222	0,267784826	4,822036573	1,421E-06	8,72322E-05	2,44743106
ZNF92	41,54670519	73,63358703	58,72107273	139,5423597	139,1474692	156,567285	82,55688143	1,308148918	0,271495771	4,818303114	1,44784E-06	8,79193E-05	2,476236171
CBL	235,4313294	390,5209884	412,4131155	671,246868	651,9732229	670,3036889	448,8395984	0,935037785	0,194134472	4,816443844	1,46139E-06	8,8265E-05	1,911940709

FDFT1	758,2273698	811,2843428	798,8797105	1322,043563	1402,696263	1224,405096	904,4555628	0,737232848	0,15343435	4,804874833	1,54848E-06	9,20405E-05	1,666975439
KLHL28	32,89114161	31,55725159	50,52743468	86,6124991	102,1162879	116,2022818	68,39741516	1,400371503	0,292290338	4,791029059	1,65928E-06	9,65822E-05	2,639695472
PPWD1	230,2379913	327,4064852	349,5952237	540,125168	530,7802658	533,3073146	489,5334112	0,815922066	0,170399032	4,788302248	1,68198E-06	9,73989E-05	1,760422927
ATP10A	109,0601011	67,05915962	90,13001862	197,2840257	215,4541459	283,7782041	80,23047126	1,394640315	0,292551167	4,767167152	1,86834E-06	0,000106543	2,629229933
ARL6IP1	1165,038858	2247,13929	2288,75623	4047,931382	4472,917842	3833,452119	2242,510623	1,114813373	0,234400118	4,756027362	1,9744E-06	0,000111459	2,165669922
ANKRD13C	242,3557803	344,4999965	284,0461193	505,2395781	628,4079256	530,8609507	482,8766939	0,930999568	0,196266104	4,743557595	2,09997E-06	0,000117369	1,90659652
UCA1	254,4735693	245,8835853	184,3568563	394,5680515	350,1129871	418,3282146	713,401105	0,767342009	0,162266314	4,728905173	2,25734E-06	0,000125539	1,702130923
ATPIF1	503,7538005	583,8091543	640,4693747	882,9663103	990,8646399	995,6700781	1233,333587	0,729595558	0,154566557	4,720267903	2,35534E-06	0,000129705	1,658174177
SGTB	133,2956792	101,2461822	94,22683764	228,5607615	189,6445347	313,13457	173,4620429	1,15973871	0,245776122	4,718679344	2,37381E-06	0,000130085	2,234169604
C10orf12	271,7846965	347,1297674	249,9059607	558,1694386	572,3000751	455,0236721	381,2038481	0,866099031	0,184201354	4,701914567	2,57733E-06	0,000139201	1,822727674
KCNIP1	287,3647109	322,1469433	344,1327983	771,0918323	686,7600902	519,8523135	244,4974996	1,051040907	0,223754471	4,697295658	2,63629E-06	0,000141704	2,072024278
ARF4	4661,886545	5262,171702	4384,961966	7617,088115	9381,232604	10849,62358	6260,399059	0,960596939	0,205411262	4,676457031	2,91874E-06	0,000153939	1,946114966
HNRNPA3	2091,184161	2912,471344	2295,584262	5683,945253	4089,140145	4463,390805	2848,848447	0,963347593	0,206114361	4,673849945	2,95605E-06	0,000155179	1,949828984
PLK2	3467,418771	3318,770958	3033,011687	7227,331869	7136,918584	5004,037211	4636,916012	0,980145443	0,21051034	4,656044169	3,22343E-06	0,000167648	1,97266427
CREBRF	90,01786125	124,9141209	98,32365667	176,8338523	225,553559	249,5291105	195,2845298	1,050766294	0,225902747	4,651409995	3,29673E-06	0,000169888	2,071629911
MIB1	432,7781791	549,6221318	667,7815016	959,9551984	1048,094647	1012,794625	709,0685819	0,869808245	0,187303603	4,64384149	3,4199E-06	0,00017543	1,827419993
CNOT6	283,9024855	470,7290028	525,7584419	798,7597139	850,5950136	1082,515994	717,3400405	1,089709145	0,235177344	4,633563447	3,59425E-06	0,000181882	2,128311242
C9orf41	12,11778901	39,44656448	60,08667908	150,368922	112,215701	140,6659201	77,72284352	1,828267438	0,395157481	4,62668057	3,71573E-06	0,000187174	3,551103569
LINC00630	29,42891618	43,39122093	27,31212685	67,36527708	98,74981689	111,3095542	57,49827316	1,46094801	0,316211602	4,62015941	3,83445E-06	0,000189763	2,752891996
MAPK6	297,7513872	486,5076286	636,3725557	1166,862835	1092,980928	1751,596501	874,241773	1,495700266	0,325434157	4,596014996	4,30648E-06	0,000210343	2,820009976
PDCD4	694,1761993	574,6049559	452,0156994	1178,892349	1516,034121	1060,49872	877,5567032	1,127520419	0,24611778	4,581222935	4,62265E-06	0,00022384	2,184829076
CEP85L	48,47115606	86,78244186	68,28031713	141,9482624	163,8349235	151,6745574	92,76675951	1,153646508	0,252377894	4,571107599	4,85153E-06	0,000233915	2,224755061
LSM14A	1203,123338	1137,375943	1103,409925	1605,940087	1588,974326	1746,703773	1379,084924	0,521575534	0,114277517	4,564113306	5,0161E-06	0,000240816	1,435522093
CSDE1	51,93338149	101,2461822	103,786082	232,1696156	188,5223777	220,1727445	86,30133909	1,305281735	0,286917511	4,549327543	5,38176E-06	0,000251911	2,471319835
PCMTD1	12,11778901	26,29770965	20,48409514	63,75642295	80,79530473	62,38227762	49,94981369	1,788425725	0,393665353	4,543010225	5,54566E-06	0,000258431	3,454377437
B3GNT5	36,35336704	55,22519027	20,48409514	103,4538184	117,8264861	105,1936446	112,1169696	1,538461425	0,33885049	4,540236688	5,61911E-06	0,000259774	2,904845484
STARD3NL	476,055997	544,3625898	648,6630128	923,8666571	972,9101277	902,7082526	636,2542311	0,7440345	0,164067932	4,534917288	5,76261E-06	0,000265316	1,674853026
PIK3R3	79,63118495	107,8206096	208,9377704	329,6086771	348,9908301	308,2418424	163,8078665	1,308538541	0,288757113	4,53162358	5,85321E-06	0,000268388	2,476905008
DNAJB4	107,3289884	151,2118305	178,8944309	233,372567	328,792004	358,3923008	198,1605292	1,066233007	0,235384512	4,529750061	5,90535E-06	0,000269678	2,093958728
EPHB2	192,1535115	237,9942724	237,6155036	351,2618019	373,6782844	370,62412	1004,20505	0,708669743	0,157159136	4,509249431	6,50574E-06	0,000295893	1,634296496
FAM104B	150,6068063	185,3988531	204,8409514	324,7968716	298,4937647	382,8559391	226,9035824	0,889669109	0,197606848	4,502218014	6,72479E-06	0,000303399	1,852751135
PGRMC2	148,8756936	188,028624	342,767192	555,7635359	592,4989013	573,6723177	352,7606611	1,337469018	0,297725589	4,492287755	7,04621E-06	0,000316629	2,527075939
NNMT	1603,010375	1467,412199	1895,461604	2514,168377	3012,991572	2541,772018	1234,537103	0,700346043	0,156284649	4,481220961	7,42172E-06	0,000326963	1,624894491
BTAF1	173,1112716	142,0076321	166,6039738	257,4315945	337,76926	371,8473019	223,4821993	1,008009612	0,225327448	4,473532281	7,69379E-06	0,000337625	2,01113456
SDC2	292,5580491	327,4064852	292,2397573	525,6897515	534,1467368	546,7623156	272,719506	0,81546319	0,182886207	4,458855603	8,23984E-06	0,000358784	1,75986308
ATP8B1	242,3557803	365,5381642	385,1009886	561,7782928	574,5443892	746,1409676	511,5739162	0,917867163	0,206125006	4,452963672	8,46931E-06	0,000367352	1,889320114
GLT8D2	57,12671964	38,131679	58,72107273	110,6715266	124,5594281	118,6486457	51,31214368	1,208950172	0,272492937	4,436629383	9,13784E-06	0,000391811	2,31169357
KIAA1430	1075,020997	1075,576325	1061,076128	1489,253804	1661,914532	1694,106951	949,6407276	0,593328306	0,134044549	4,426351623	9,58403E-06	0,00040938	1,50872337
GCLC	200,8090751	278,7557223	255,3683861	443,8890579	509,4592826	362,0618466	255,5576968	0,835474701	0,18897072	4,421185995	9,81606E-06	0,00041301	1,784444091
FAM217B	166,1868208	119,6545789	117,4421455	226,1548588	337,76926	275,2159307	290,6763158	1,065041148	0,24111288	4,417188948	9,99928E-06	0,000418474	2,092229552
SOD2	4440,304117	3635,65836	3191,422023	5829,50237	7807,968476	9283,950728	5462,360106	1,024763988	0,232018585	4,416732336	1,00204E-05	0,000418474	2,034626511
NUAK1	114,2534393	172,2499982	152,9479104	291,1142331	250,2410132	254,4218381	262,6470023	0,846718886	0,191865895	4,413076564	1,01912E-05	0,000424029	1,798406165
IKBIP	488,173786	545,6774753	599,5011844	939,505025	839,3734435	916,1632537	613,0428885	0,72065869	0,163622045	4,404410714	1,06072E-05	0,000439709	1,647934259
OPN3	493,3671242	453,6354915	438,359636	680,870479	682,2714621	759,5959687	385,3053066	0,617212824	0,140250547	4,400787292	1,07859E-05	0,000445473	1,533908923
HAT1	995,3898119	1153,154568	1412,036958	1951,187132	1935,720842	2106,319256	1387,595859	0,750097123	0,170975823	4,387153165	1,14844E-05	0,000469148	1,681906054
INADL	100,4045375	118,3396934	120,1733582	214,125345	227,797873	292,3404775	90,97228606	1,111376081	0,253390572	4,386019861	1,15444E-05	0,000469889	2,160516249
PMM2	682,0584102	635,0896882	669,1471079	1032,132281	1192,852902	1229,297824	791,9608825	0,798719537	0,182206427	4,383596941	1,16736E-05	0,000473433	1,739556499
NCBP2	500,291575	585,1240398	536,6832927	784,3242974	840,4956005	825,647792	834,8138732	0,593304592	0,135416358	4,381336195	1,17954E-05	0,000476652	1,50869857
KLHL2	344,4914306	436,5419803	443,8220614	739,8150965	736,1349986	705,7759645	463,2305146	0,830287698	0,189731903	4,376110108	1,20816E-05	0,000486468	1,778039899
RPS13	11049,69247	11233,06668	13213,60697	18649,35519	19561,441	22754,85315	16885,97211	0,780281442	0,178433677	4,372949412	1,22579E-05	0,000488317	1,717465884

SEMA3C	758,2273698	796,8206025	867,1600276	1362,943909	1233,250554	1871,468329	983,8070586	0,88255916	0,202274254	4,363180898	1,28185E-05	0,000508844	1,843642793
SETD8	57,12671964	76,263358	116,0765391	218,9371505	176,1786506	256,868202	122,9424151	1,377485055	0,316436324	4,353119256	1,34214E-05	0,000529039	2,598150595
DSG2	569,5360837	1019,036249	1261,820261	1903,069077	2156,785773	2282,457452	1280,985642	1,152367961	0,264947952	4,349412601	1,36503E-05	0,000536179	2,222784309
MGLL	725,3362281	853,3606783	673,2439269	1119,947731	1293,847033	1378,526017	764,1803503	0,751355121	0,173013916	4,34274386	1,40714E-05	0,000548883	1,683373278
GANC	29,42891618	64,42938865	77,83956153	128,7157973	135,7809982	144,3354659	79,617298	1,229547329	0,283406607	4,338456827	1,43487E-05	0,000557761	2,344934019
SLC7A11	155,8001445	201,1774789	217,1314085	393,3651001	311,9596488	355,945937	223,2039818	0,880517659	0,203137915	4,334580564	1,46038E-05	0,000565722	1,841035772
TRIM55	1054,247644	1015,091593	1196,271156	2054,640951	1620,394723	1913,056514	745,4545699	0,774726753	0,178915902	4,33011681	1,4903E-05	0,000573358	1,710865982
ZNF323	140,22013	119,6545789	176,1632182	242,996178	320,9369049	267,8768392	137,8032524	0,932282206	0,215399914	4,328145672	1,5037E-05	0,000576538	1,908292346
SLC2A3	327,1803034	470,7290028	621,3508859	870,9367965	890,992666	903,9314345	635,1326307	0,906005167	0,20991216	4,316115693	1,58799E-05	0,000600655	1,873849605
PPP2CA	931,3386414	1192,601133	1417,499384	1947,578278	1948,064569	2533,209744	1877,37084	0,859049586	0,199087586	4,314932949	1,59651E-05	0,000601853	1,813842999
NADKD1	1104,449913	844,1564799	890,3753354	1489,253804	1969,385553	1669,643313	1203,061231	0,854403945	0,198187194	4,311095631	1,62448E-05	0,000608312	1,808011614
CENPQ	162,7245953	220,9007611	255,3683861	396,9739542	397,2435816	393,8645763	379,9529488	0,888845712	0,206425962	4,305881404	1,66322E-05	0,000614839	1,851694007
MAN2A1	424,1226155	412,8740416	486,155858	639,9701322	683,3936191	768,1582421	706,0560714	0,659779791	0,153362738	4,302086678	1,69197E-05	0,000620125	1,579841463
NUAK2	3,462225433	7,889312896	4,096819028	30,07378441	21,32098319	30,57954785	35,21754968	2,366851993	0,551917384	4,288417181	1,79951E-05	0,000651563	5,158143799
WDR75	695,907312	775,7824348	853,5039642	1194,530717	1246,716438	1236,636915	1124,308913	0,660251041	0,154113519	4,284186403	1,83409E-05	0,000658063	1,580357596
AMOTL2	699,3695374	636,4045736	574,9202703	945,5197818	1009,941309	1115,541906	1011,043133	0,685936136	0,160549994	4,272414592	1,93368E-05	0,00068482	1,608745525
CHRDL1	2728,233641	3100,499968	2322,896389	4121,311415	4268,685266	4601,610361	2198,669231	0,672348083	0,157453592	4,270134917	1,95355E-05	0,000687967	1,593664654
TENM2	195,6157369	176,1946547	146,1198787	315,1732606	291,7608226	335,1518445	145,0198498	0,866725774	0,203059812	4,268327468	1,96944E-05	0,000691397	1,823519685
LPP	289,0958236	310,3129739	278,5836939	501,630724	424,1753498	538,2000422	290,0399471	0,736479201	0,172663568	4,265400103	1,99544E-05	0,000698343	1,666104858
ZMYM6	107,3289884	142,0076321	124,2701772	202,0958312	231,1643441	209,1641073	187,7069134	0,774823461	0,181914298	4,259277416	2,05089E-05	0,000715518	1,71098067
BMI1	115,984552	213,0114482	188,4536753	325,999823	345,6243591	380,4095753	248,0725537	1,015361467	0,238498527	4,257307079	2,06904E-05	0,000719616	2,021409293
RHEB	1661,868208	1593,641205	1470,758031	2223,054144	2196,061269	2407,222007	1426,140716	0,530848014	0,124971133	4,247765064	2,15914E-05	0,00074633	1,444778184
SRSF5	1798,626112	1743,53815	1642,82443	2470,862127	2451,913067	2609,047023	2038,742988	0,538949614	0,12710707	4,240123013	2,23397E-05	0,000765136	1,452914306
MTF2	152,337919	228,790074	279,9493002	412,6123221	412,9537797	499,058221	365,405318	0,99661823	0,235301767	4,235489774	2,28054E-05	0,000778711	1,995317361
KIF5B	2655,526907	3000,568672	2485,403544	4336,639712	5248,328336	4725,151734	2893,042371	0,813613448	0,192148077	4,234304396	2,2926E-05	0,000780457	1,75760813
FLG	1055,978757	834,9522815	1160,765391	1987,275674	1686,601986	1734,471954	705,8378581	0,825923558	0,195312869	4,228720619	2,35024E-05	0,000797661	1,77266947
MT1L	995,3898119	861,2499912	711,4809045	1373,770472	1550,820988	1763,82832	604,7527105	0,869420504	0,2060557	4,219347022	2,45011E-05	0,000821628	1,826928919
FNTA	638,7805923	658,7576268	611,7916415	937,0991222	962,8107146	1058,052356	990,9372153	0,631409265	0,149820909	4,214426877	2,50413E-05	0,00083476	1,549077437
SLC11A2	361,8025577	493,082056	523,0272292	685,6822845	844,9842286	933,2878005	738,2080079	0,835607436	0,198315776	4,21351974	2,51422E-05	0,000835642	1,784608277
EHD2	100,4045375	95,98664024	98,32365667	175,630901	151,4911964	173,6918318	610,2586136	0,765310639	0,181664476	4,212769921	2,52258E-05	0,000835949	1,699735943
RCHY1	143,6823555	243,2538143	267,6588432	457,121523	421,9310358	470,9250369	241,3180145	1,037994223	0,247417027	4,195322511	2,72484E-05	0,000892443	2,053370868
NEDD4	2783,629248	2218,211809	1898,192816	3582,389199	4163,202507	4722,70537	2360,37581	0,854123756	0,203884769	4,189247491	2,79881E-05	0,000911358	1,80766051
DICER1	290,8269363	377,3721335	452,0156994	590,6491258	740,6236266	648,2864145	395,9156928	0,818085998	0,195389454	4,186950623	2,82727E-05	0,000917965	1,763065408
FUCA1	96,94231212	89,41221282	106,5172947	204,501734	199,7439478	157,7904669	260,022888	0,941320567	0,225740996	4,16991413	3,04714E-05	0,000967232	1,920285162
LOC152217	202,5401878	205,1221353	185,7224626	289,9112817	302,9823927	331,4822987	308,7893643	0,640044853	0,153486269	4,170046341	3,04538E-05	0,000967232	1,558377608
COL4A3BP	195,6157369	261,6622111	355,0576491	443,8890579	500,4820265	517,4059497	405,1234285	0,842019011	0,201994324	4,168528084	3,06573E-05	0,000970152	1,79255702
THRB	32,89114161	43,39122093	62,81789176	115,4833321	113,337858	122,3181914	47,6251035	1,323603433	0,317885808	4,163770139	3,13035E-05	0,00098782	2,502904826
CLDN16	3585,134436	4169,501866	3285,64886	5438,543173	7176,194079	6931,771907	2564,802115	0,824077202	0,198197447	4,15785983	3,21243E-05	0,001010881	1,770402265
ABCB10	257,9357947	285,3301497	292,2397573	464,3392313	452,2292751	456,246854	254,8201477	0,714266822	0,171895927	4,155228308	3,24963E-05	0,00101973	1,640649227
CYBRD1	931,3386414	781,0419767	865,7944212	1234,228112	1318,534487	1439,685113	1348,36886	0,632013501	0,152363303	4,148069059	3,35291E-05	0,001046295	1,549726365
WEE1	851,7074564	694,2595349	708,7496918	1156,036273	1009,941309	1296,572829	833,1818887	0,620555036	0,150020346	4,136472496	3,52686E-05	0,001092824	1,537466565
FIGN	282,1713728	502,2862544	419,2411472	868,5308937	815,8081463	742,4714219	347,804712	1,008361482	0,244440328	4,125184615	3,70437E-05	0,001130841	2,011625131
MYL12B	5577,645172	4081,404538	3861,934737	7490,778221	8392,612278	9226,461178	5420,878397	0,893338481	0,216531924	4,125666383	3,69663E-05	0,001130841	1,857469448
TMEM2	579,92276	1041,389302	1633,265186	2585,142508	2136,586947	3207,182979	1166,69927	1,283543144	0,311144436	4,125232511	3,7036E-05	0,001130841	2,434361029
EIF4B	2833,831517	2703,404552	3226,927788	6393,686565	6878,822472	4530,66581	3950,492614	1,022452024	0,248611434	4,112650848	3,91142E-05	0,001181206	2,031368569
TINAGL1	1071,558771	727,1316719	625,4477049	1353,320298	1435,238816	1569,342396	1896,871975	0,84849467	0,206462856	4,109672258	3,96221E-05	0,001190146	1,800621149
FZD2	60,58894507	56,54007576	35,50576491	109,4685753	125,6815851	94,18500739	86,53537342	1,120050162	0,272994297	4,102833557	4,08121E-05	0,001209718	2,173545297
TJP1	476,055997	624,5706043	663,6846825	945,5197818	951,5891445	1031,142354	497,6972888	0,728346397	0,177488761	4,103619807	4,06736E-05	0,001209718	1,656739065
HIST1H2BC	4173,712759	3609,36065	3912,462172	6224,070421	7320,952334	6267,584128	3347,630809	0,760635899	0,185558042	4,099180457	4,14616E-05	0,001216134	1,694237232
KDELR1	4715,551039	4904,522851	4216,992386	6197,605491	7149,262311	7642,440599	5241,02755	0,60114894	0,147057558	4,087847962	4,35393E-05	0,001267153	1,516924141

STC2	214,6579768	157,7862579	90,13001862	286,3024276	325,4255329	324,1432072	1364,568561	1,02577569	0,251193977	4,083599869	4,43434E-05	0,001287218	2,03605381
IL1RAP	25,96669075	28,92748062	73,7427425	95,03315873	107,727073	136,9963744	89,16373909	1,386652846	0,34038835	4,07373768	4,62646E-05	0,001332657	2,614713446
ATRX	53,66449421	99,93129669	131,0982089	224,9519074	178,4229646	207,9409254	169,458446	1,088023015	0,267195856	4,07200558	4,66101E-05	0,001339175	2,125825262
ACO1	931,3386414	1146,580141	781,126828	1500,080366	1678,746887	1727,132863	1096,215973	0,778663788	0,191886645	4,057936331	4,95083E-05	0,001415206	1,715541217
CHRNB1	24,23557803	24,98282417	20,48409514	68,56822845	60,59647854	45,25773082	90,92384507	1,326661123	0,326994018	4,057141869	4,96769E-05	0,001416422	2,50821518
NBN	463,938208	660,0725123	815,2669866	1323,246514	1131,134266	1635,394219	786,2019891	1,074878853	0,265408197	4,049908281	5,12377E-05	0,001451208	2,106545173
SLC24A6	19,04223988	32,87213707	25,94652051	58,94461744	78,55099071	106,4168265	57,67796266	1,63259249	0,40313963	4,049694872	5,12845E-05	0,001451208	3,100696866
NEXN	289,0958236	389,2061029	363,2512871	731,3944368	755,2116678	1059,275538	305,1912518	1,288073263	0,31825274	4,047328111	5,18056E-05	0,001462272	2,442017032
FAM49B	363,5336704	376,0572481	402,8538711	595,4609313	571,1779181	579,7882273	441,9696588	0,611077072	0,151133682	4,043288447	5,27067E-05	0,001483978	1,527399093
DPYSL3	2020,20854	1931,566774	2507,253245	3279,245452	3877,05247	4651,760819	3064,171643	0,870249965	0,215303526	4,041968013	5,30045E-05	0,00148863	1,827979593
CTGF	808,4296385	812,5992283	946,3651955	1329,261271	1256,815851	1341,83056	1418,614786	0,612796735	0,15180257	4,036800797	5,4185E-05	0,001516669	1,529220806
UBLCP1	27,69780346	69,68893058	142,0230596	271,8670111	200,8661048	285,001386	130,39554	1,651206115	0,409076795	4,036420875	5,42728E-05	0,001516669	3,140961186
CPM	1,731112716	13,14885483	8,193638056	32,47968716	20,19882618	33,02591168	59,58049939	1,80676899	0,448262509	4,030604733	5,56335E-05	0,001550836	3,498578809
PPP1R21	145,4134682	143,3225176	150,2166977	249,0109349	243,5080712	243,4132009	156,182491	0,745339896	0,185051826	4,027735963	5,63165E-05	0,001564129	1,676369172
TMEM64	297,7513872	426,0228964	544,8769307	994,8407883	822,5410884	730,2396027	440,2654088	1,003190583	0,249089252	4,027434236	5,63888E-05	0,001564129	2,004427981
ZNF564	24,23557803	22,35305321	36,87137125	64,95937432	74,06236266	75,83727868	39,46251609	1,358649397	0,337941712	4,020366087	5,81078E-05	0,001599958	2,564449918
LINC00152	200,8090751	169,6202273	196,6473133	346,4499964	329,914161	419,5513965	190,566832	0,951800741	0,236902966	4,017681832	5,87735E-05	0,001614331	1,934285485
GIGYF2	701,1006501	661,3873978	716,9433299	970,7817607	1080,637201	1317,366922	788,0271402	0,696110822	0,173298382	4,01683395	5,89853E-05	0,001616196	1,620131395
ZBTB4	479,5182224	507,5457963	420,6067535	743,4239506	814,6859893	1026,249626	753,5990815	0,876467893	0,218472445	4,011800625	6,02574E-05	0,001639092	1,835875077
CFL2	687,2517484	1033,499989	1487,145307	2316,884351	1830,238083	2530,76338	1279,361105	1,056544731	0,263477954	4,00999292	6,07206E-05	0,001647669	2,079944064
VPS33A	93,48008668	168,3053418	243,077929	372,9149267	411,8316227	298,456387	405,5950314	1,093710606	0,273229936	4,002894497	6,25722E-05	0,001693822	2,134222524
ZNF599	6,924450865	13,14885483	4,096819028	18,04427065	39,27549535	35,47227551	37,14757058	1,923307382	0,481651121	3,993154582	6,52E-05	0,001743946	3,792915897
ARMC1	1365,847933	1201,805331	1100,678712	1675,711267	2188,20617	2065,954253	1279,388524	0,693735182	0,174122381	3,984181583	6,77131E-05	0,00180258	1,617465771
THAP6	34,62225433	72,31870155	53,25864736	122,7010404	102,1162879	130,8804648	90,19604114	1,130886327	0,284224505	3,97884879	6,92498E-05	0,001834792	2,189932385
CSF1	585,1160981	403,6698432	540,7801117	787,9331515	848,3506996	1084,962358	530,1084201	0,832857954	0,209468888	3,976046099	7,00705E-05	0,001847823	1,781210416
GLIPR1	368,7270086	487,8225141	486,155858	665,2321111	759,7002958	1069,060993	581,1635603	0,89112972	0,224108477	3,976332051	6,99864E-05	0,001847823	1,854627844
GALNT2	1639,363742	1738,278608	1384,724831	2571,910043	2613,503676	2199,281082	2093,484993	0,633051659	0,159311489	3,97367235	7,07729E-05	0,001861974	1,550841943
MYBL1	81,36229767	98,6164112	129,7326026	193,6751716	197,4996338	216,5031988	99,15385969	0,964988805	0,242962712	3,971756811	7,13445E-05	0,001872627	1,952048374
CLIP4	406,8114883	491,7671705	449,2844867	664,0291598	806,8308902	1125,327361	507,057681	0,944557132	0,237905934	3,970296655	7,17832E-05	0,001875378	1,924597997
ACAT2	702,8317628	728,4465574	1025,570363	1408,656062	1421,772932	1990,116974	919,1024113	0,971671716	0,24483558	3,968670384	7,22748E-05	0,001883839	1,961111708
LMNB1	869,0185836	882,2881589	867,1600276	1431,512138	1649,570805	2014,580613	1311,500526	0,960479002	0,242467941	3,961261846	7,45547E-05	0,001929833	1,945955882
PIK3CB	758,2273698	750,7996106	738,7930314	1176,486446	1269,159578	1376,079653	777,6819639	0,765768713	0,193946363	3,94835304	7,86907E-05	0,002027549	1,700275717
UBE2J1	787,6562859	674,5362526	611,7916415	1067,017871	1020,040722	1301,465557	757,9292059	0,709559697	0,179737686	3,947751366	7,88887E-05	0,002027998	1,635304955
TEX15	15,58001445	11,83396934	2,731212685	22,85607615	43,76412339	50,15045848	24,57912771	1,990461461	0,504913387	3,942183968	8,0743E-05	0,002066212	3,973640789
CPA4	0	5,259541931	2,731212685	10,82656239	15,71019814	29,35636594	3048,740874	2,689361256	0,682616347	3,939784432	8,15549E-05	0,002073293	6,450277622
GINS1	251,0113439	357,6488513	417,8755409	670,0439166	542,0018359	557,7709528	445,1694661	0,780987869	0,198233857	3,939729966	8,15734E-05	0,002073293	1,718307061
EIF2C2	1035,205404	958,5515169	740,1586377	1442,3387	1652,937276	2465,934739	987,924475	1,024845699	0,261446918	3,919899716	8,85858E-05	0,00224644	2,03474175
HINT3	93,48008668	109,1354951	142,0230596	205,7046854	273,8063105	278,8854764	179,5191281	1,13388289	0,289490446	3,916823187	8,97235E-05	0,002261016	2,194485732
SEC11A	1410,856864	1600,215632	1746,610512	2173,733137	2840,179392	2659,197481	2038,933249	0,68907599	0,176077617	3,913478616	9,0976E-05	0,002281303	1,612250582
COX10	534,9138294	482,5629722	436,9940296	648,3907919	712,5697014	757,1496048	665,0483398	0,544765344	0,139390486	3,908196029	9,29878E-05	0,00232656	1,458783052
CLCN5	34,62225433	24,98282417	46,43061565	91,4243046	62,84079256	96,63137122	44,44532415	1,241698812	0,318609429	3,897244391	9,72934E-05	0,002418129	2,364768262
MGST3	1357,19237	1391,148841	1166,227817	1747,88835	2281,345201	2397,436552	1109,967754	0,715412035	0,183859963	3,891070265	9,9803E-05	0,002469575	1,641952094
RALGAPB	304,6758381	374,7423626	349,5952237	505,2395781	511,7035966	645,8400507	850,5593236	0,689312357	0,177429033	3,885003186	0,000102329	0,002520962	1,612514749
SHISA9	48,47115606	28,92748062	35,50576491	125,1069431	155,9798244	59,93591379	39,00514731	1,6070168	0,413917465	3,882457095	0,000103406	0,002536389	3,046212952
CYCS	938,2630923	1685,683189	1958,279495	2795,658999	2840,179392	2736,257942	1976,304407	0,868292937	0,224257367	3,87185915	0,000108008	0,0026073	1,825501604
DLEU1	119,4467774	84,15267089	83,3019869	172,0220468	186,2780637	228,7350179	100,0341498	1,040970285	0,268987854	3,869952752	0,000108856	0,0026073	2,057611035
EDIL3	444,8959681	314,2576304	337,3047666	564,1841955	668,805578	747,3641495	400,8582918	0,855988867	0,221003252	3,873195804	0,000107417	0,0026073	1,809998959
HLA-C	22,50446531	23,66793869	34,14015857	89,01840185	92,01687483	48,92727657	873,2822089	1,512544345	0,390899308	3,869396319	0,000109105	0,0026073	2,853127748
IL32	290,8269363	282,7003788	251,271567	405,3946138	445,496333	510,0668582	233,7412802	0,723597296	0,186976366	3,869993364	0,000108838	0,0026073	1,651294335
RASA1	1487,025823	1780,354944	1813,525223	2374,626017	2502,410132	2341,170184	1508,808122	0,505550947	0,130565289	3,872016455	0,000107939	0,0026073	1,419665405

TBC1D2B	12,11778901	11,83396934	32,77455222	74,58298534	66,20726359	45,25773082	38,24648169	1,698789144	0,439085108	3,868929079	0,000109314	0,0026073	3,24628383
TMEM87B	237,1624421	323,4618287	363,2512871	529,2986056	480,2832003	598,135956	495,5829439	0,79472105	0,205283652	3,871331405	0,000108243	0,0026073	1,734741924
CNN3	4069,845996	2866,450352	3707,62122	5278,55064	5244,961865	6192,970031	3410,94036	0,651630103	0,168613762	3,864631781	0,000111257	0,002648009	1,570942203
CXCL6	0	0	0	0	14,58804113	7,339091485	10336,20346	4,7944135	1,242803935	3,857739233	0,000114441	0,002712981	27,74995449
SFXN1	1125,223266	1275,438918	1170,324636	1987,275674	1922,254958	1601,145126	1773,915176	0,625503327	0,162433051	3,850837754	0,000117714	0,00277816	1,542748965
PLOD2	1348,536806	1638,347311	1793,041128	2504,544766	2814,369781	3532,549368	2591,744565	0,888449802	0,231579081	3,836485566	0,000124808	0,002914943	1,851185927
DLG1	195,6157369	270,8664094	267,6588432	350,0588505	390,5106395	490,4959476	311,4882465	0,740113209	0,193362658	3,827591203	0,000129403	0,003009769	1,670306904
ANKRD33B	145,4134682	136,7480902	135,1950279	212,9223936	214,3319889	324,1432072	147,6805428	0,848785109	0,221957183	3,824093902	0,000131254	0,003040219	1,800983681
DCUN1D5	500,291575	499,6564834	559,8986005	719,3649231	878,6489389	798,7377899	718,3648161	0,61939656	0,161950752	3,824598229	0,000130986	0,003040219	1,536232482
IPO8	782,4629478	976,9599137	950,4620145	1316,028806	1335,366842	1267,216463	1136,691192	0,530236354	0,13951236	3,800640718	0,000144322	0,003282021	1,444165771
TMTC3	256,204682	458,8950335	561,2642068	940,7079763	859,5722697	795,0682442	696,4159552	1,020760096	0,269155595	3,792453567	0,000149166	0,003371695	2,028987668
CNOT7	1374,503497	1103,18892	1365,606343	1883,821855	1964,896925	2486,728831	1510,551616	0,721498313	0,190363018	3,790118069	0,000150576	0,003392459	1,648893607
TENM4	358,3403323	316,8874013	400,1226584	526,8927029	565,5671331	655,625506	377,7857912	0,701107273	0,185216126	3,785346818	0,000153494	0,003448711	1,625752083
ABLIM1	154,0690318	168,3053418	204,8409514	268,2581569	319,8147479	434,2295795	141,1293622	0,952634695	0,25195302	3,781001293	0,000156199	0,003481623	1,935403928
PPP4R2	633,5872542	765,2633509	813,9013802	1057,39426	1101,958184	1367,51738	1002,881899	0,670905403	0,177504765	3,779647295	0,000157051	0,003493678	1,592071803
FNDC3A	782,4629478	746,8549542	839,8479007	1259,490091	1135,622894	1629,27831	881,4600113	0,764173254	0,202345154	3,776582921	0,000158995	0,003516035	1,698396442
UPF3A	152,337919	166,9904563	215,7658021	287,505379	267,0733684	319,2504796	188,6179942	0,702172912	0,186563186	3,76372706	0,0001674	0,003651586	1,626953381
MCAM	2169,084234	1237,307239	991,4302048	2345,755184	3074,710208	3422,462996	1876,886073	1,008887937	0,268191622	3,761817505	0,000168683	0,003672454	2,012359329
SORCS2	45,00893063	27,61259514	50,52743468	86,6124991	122,3151141	83,17637016	223,1330765	1,254381613	0,333770758	3,758213037	0,000171131	0,003716142	2,385648698
TGFB2	105,5978757	210,3816772	193,9161007	460,7303772	478,0388863	288,6709317	178,8223366	1,26268992	0,336165821	3,756151999	0,000172546	0,003727656	2,399426999
SEC22B	829,2029911	962,4961733	943,6339828	1324,449465	1257,938008	1469,041479	1130,13509	0,565429597	0,150697807	3,752075818	0,000175376	0,003781533	1,479828098
CA13	135,0267919	136,7480902	86,03319959	217,7341991	283,9057235	297,2332051	106,232781	1,161065328	0,309548249	3,750837975	0,000176245	0,003792972	2,23622496
FAM196B	195,6157369	252,4580127	232,1530783	435,4683983	390,5106395	373,0704838	175,8513406	0,814523414	0,217193266	3,750224072	0,000176677	0,003795	1,758717074
RNF138	993,6586992	907,2709831	633,641343	1320,840611	1574,386285	1568,119214	896,6391412	0,817407047	0,218039171	3,748899993	0,000177612	0,003807809	1,762235882
PDGFRB	48,47115606	28,92748062	46,43061565	70,97413121	96,50550287	84,39955207	63,54355246	1,039532419	0,277880059	3,740939247	0,000183334	0,003915537	2,055561333
ADAM17	311,6002889	366,8530497	484,7902516	671,246868	622,7971406	720,4541474	463,6010557	0,789560006	0,211321994	3,736288824	0,000186756	0,003966009	1,728547209
EIF2S3	1094,063237	1796,133569	2776,277695	3973,348396	3550,50478	4776,525375	2751,635478	1,117559916	0,299228415	3,734805459	0,00018786	0,003978095	2,169796762
PEG10	12,11778901	22,35305321	21,84970148	34,88558992	52,74137947	48,92727657	453,9072678	1,249391482	0,334879468	3,73086917	0,00019082	0,004029465	2,377411244
BRAF	273,5158092	295,8492336	267,6588432	440,2802038	460,0843741	511,2900401	375,2454088	0,753345358	0,202002791	3,729380931	0,000191951	0,004031001	1,685697139
RPS6KA3	476,055997	600,9026656	514,8335912	744,626902	836,0069725	895,3691611	1005,187511	0,635501531	0,170405152	3,729356313	0,00019197	0,004031001	1,553477699
ZNF100	67,51339594	57,85496124	101,0548694	132,3246514	158,2241384	182,2541052	99,32150536	1,059590085	0,284055513	3,730221865	0,000191311	0,004031001	2,084339211
ATG3	792,8496241	983,5343411	1091,119468	1396,626548	1399,329792	1334,491468	1152,903161	0,524383626	0,140750534	3,725624422	0,000194832	0,004083482	1,438318944
RFC5	223,3135404	232,7347304	251,271567	357,2765588	353,4794582	384,079121	278,9949333	0,628361613	0,168821054	3,722057161	0,000197606	0,004133909	1,54580851
C7orf41	53,66449421	47,33587738	43,69940296	74,58298534	99,8719739	92,96182547	113,6652731	0,895363203	0,240841011	3,717652569	0,000201083	0,004198813	1,860078107
SLC9A6	226,7757658	305,053432	180,2600372	422,2359331	399,4878956	573,6723177	358,4781059	0,968962679	0,260794562	3,715425167	0,000202862	0,004228117	1,957432663
RPL23AP82	29,42891618	24,98282417	36,87137125	68,56822845	66,20726359	92,96182547	32,45988991	1,317441965	0,354685483	3,71439494	0,00020369	0,004237516	2,492238203
IGFBP3	915,7586269	1029,555333	1138,91569	1488,050853	1732,610424	1926,511515	802,901828	0,738209111	0,198902053	3,711420273	0,0002061	0,004263946	1,668103853
SEC11C	460,4759825	443,1164077	400,1226584	660,4203056	613,8198845	722,9005113	588,5533602	0,616095123	0,165995776	3,711510851	0,000206026	0,004263946	1,532721016
CABIN1	264,8602456	284,0152643	301,7990017	372,9149267	478,0388863	519,8523135	293,7617501	0,686847389	0,185154586	3,709588864	0,000207596	0,004283873	1,609761983
LOX	38,08447976	47,33587738	36,87137125	95,03315873	96,50550287	73,39091485	133,339309	1,111498776	0,299651146	3,709309273	0,000207825	0,004283873	2,160699999
LSM11	24,23557803	22,35305321	32,77455222	57,74166607	66,20726359	74,61409676	46,55010332	1,319436593	0,355934813	3,706961342	0,000209761	0,004315851	2,495686281
MMD	425,8537282	515,4351092	352,3264364	588,2432231	742,8679407	842,7723388	687,8988756	0,748300508	0,202050918	3,703524419	0,000212625	0,004366775	1,679812848
FAM160B1	46,74004334	85,46755638	135,1950279	190,0663175	212,0876749	240,9668371	128,853023	1,255811602	0,339215349	3,702107247	0,000213816	0,004375248	2,388014509
PHKB	178,3046098	205,1221353	289,5085446	340,4352395	409,5873087	503,9509486	214,1602097	0,894429003	0,241577499	3,702451621	0,000213526	0,004375248	1,858874025
HIPK2	1440,28578	1922,362576	1760,266576	2788,44129	2426,103456	2465,934739	1419,56532	0,583210474	0,157679498	3,698708342	0,000216699	0,004426184	1,498179487
PRMT6	238,8935549	245,8835853	244,4435353	371,7119753	366,9453423	347,3836636	296,0846074	0,574105883	0,155351627	3,695525397	0,000219433	0,004470382	1,488754515
TRIM6	115,984552	76,263358	105,1516884	153,9777762	252,4853273	260,5377477	83,33121789	1,170142928	0,316660239	3,695263194	0,000219659	0,004470382	2,250339899
NAAA	39,81559248	36,81679352	53,25864736	81,80069359	89,77256081	117,4254638	118,6522984	1,150197461	0,311870835	3,688057148	0,000225973	0,004578927	2,219442697
RAP1A	290,8269363	389,2061029	421,9723599	688,0881873	705,8367593	604,2518656	500,1933789	0,856118142	0,232102334	3,68853741	0,000225547	0,004578927	1,810161154
HKDC1	685,5206357	478,6183157	576,2858766	885,372213	885,3818809	1136,335998	416,1925332	0,742035916	0,201260256	3,686947102	0,000226961	0,004585745	1,672534437

PTPLB	351,4158814	491,7671705	479,3278263	648,3907919	708,0810734	673,9732347	478,3486809	0,614555051	0,167034957	3,679200222	0,000233967	0,004701932	1,531085715
C1orf116	15,58001445	18,40839676	19,1184888	60,14756882	51,61922246	51,37364039	29,27867185	1,611939247	0,439428766	3,668260638	0,000244206	0,004881516	3,056624321
CAP2	474,3248843	541,7328189	573,5546639	696,5088469	834,8848155	817,0855186	470,3590801	0,561219525	0,153323282	3,660367291	0,000251854	0,004989996	1,475515959
PODXL	180,0357225	165,6755708	151,582304	295,9260386	278,2949385	305,7954785	1031,513989	0,825474021	0,225878255	3,654508576	0,000257675	0,005087384	1,7721172
E2F5	228,5068786	239,3091579	301,7990017	507,6454808	454,4735891	433,0063976	189,2168075	0,85654657	0,234568471	3,651584395	0,000260627	0,005136646	1,810698785
GADD45B	225,0446531	185,3988531	143,388666	271,8670111	318,6925909	321,6968434	239,9120165	0,726801434	0,199096474	3,650498773	0,000261732	0,005149374	1,654965835
LBR	1551,076994	1513,433191	1705,642322	2244,707268	2049,0587	2458,595647	1251,946396	0,501070446	0,13731358	3,649096069	0,000263165	0,005161756	1,415263266
UHRF1BP1L	107,3289884	143,3225176	167,9695801	245,4020808	256,9739553	266,6536573	210,1342175	0,87085248	0,238656175	3,648983643	0,00026328	0,005161756	1,828743175
USP37	84,8245231	122,2843499	147,485485	224,9519074	186,2780637	288,6709317	189,2804766	0,972560397	0,26728156	3,638711169	0,000274006	0,005325575	1,962320098
AP4E1	50,20226877	82,83778541	144,7542723	199,6899285	185,1559067	218,9495626	143,2088355	1,108459896	0,304943156	3,634972208	0,000278011	0,005347896	2,156153509
DANCR	839,5896674	703,4637333	662,3190762	999,6525938	1217,540356	1034,811899	1059,993702	0,562575515	0,154765487	3,635019186	0,00027796	0,005347896	1,47690345
PSMD1	2390,666661	2523,265241	2062,065577	3133,688335	3632,422242	3815,10439	2569,519638	0,601100258	0,165757421	3,626385198	0,000287417	0,005463345	1,516872955
REL	110,7912138	120,9694644	167,9695801	253,8227404	210,9655179	227,511836	150,4014686	0,786594532	0,217018218	3,624555292	0,000289459	0,005474383	1,724997811
TAF2	328,9114161	356,3339658	494,349496	629,1435698	675,5385201	792,6218804	461,4961968	0,828427697	0,228549549	3,624718142	0,000289277	0,005474383	1,775749029
SYNRG	574,7294218	798,135488	695,0936284	1086,265093	1106,446812	956,5282568	1137,408727	0,604634946	0,166998585	3,620599228	0,000293921	0,005532564	1,52059394
TMEM192	167,9179335	211,6965627	297,7021827	371,7119753	386,0220115	366,9545742	258,4958462	0,725653804	0,200437694	3,620345993	0,000294209	0,005532564	1,653649871
PATL1	1300,06565	1316,200368	1607,318665	2077,497027	2288,078144	2321,599273	1543,422994	0,662598328	0,183342468	3,613992633	0,000301518	0,005645415	1,582930955
KITLG	74,4378468	128,8587773	120,1733582	232,1696156	168,3235515	193,2627424	108,3663826	0,863730392	0,239156474	3,611570185	0,000304349	0,00567949	1,819737548
TCF4	180,0357225	199,8625934	240,3467163	325,999823	355,7237722	489,2727657	306,490245	0,914332834	0,253337246	3,609152809	0,000307199	0,005723166	1,884697303
GNG12	3050,220606	3619,879734	3758,148655	4302,957073	5409,918946	5181,398588	4275,868975	0,51386393	0,142426568	3,607921863	0,000308659	0,005739916	1,427869302
RPL6	10487,08084	11079,22508	10415,47958	14123,85211	16867,14202	17778,94912	17189,06471	0,608726022	0,168737373	3,607535251	0,00030912	0,005739916	1,524912035
ZNF33A	162,7245953	169,6202273	202,1097387	306,752601	300,7380787	244,6363828	186,6830841	0,670760941	0,186123814	3,603842656	0,000313547	0,005786845	1,59191239
YTHDF3	308,1380635	610,106864	654,1254381	968,375858	1063,804846	826,870974	656,6011342	0,859289462	0,238490541	3,603033727	0,000314525	0,005792411	1,814144611
TNS1	441,4337427	295,8492336	248,5403544	505,2395781	592,4989013	708,2223283	700,0650078	0,877580619	0,243856756	3,598754582	0,000319745	0,005878908	1,837291602
C11orf57	245,8180057	261,6622111	304,5302144	415,0182249	364,7010283	435,4527614	362,0430478	0,578187874	0,160780157	3,596139513	0,000322975	0,005918918	1,492972792
FAM73A	24,23557803	36,81679352	49,16182834	80,59774222	59,47432153	118,6486457	94,75174641	1,212298976	0,337336941	3,593733233	0,000325974	0,005964149	2,317065739
HBP1	225,0446531	169,6202273	254,0027797	359,6824615	371,4339703	335,1518445	381,6936056	0,719145622	0,200143373	3,593152297	0,000326702	0,005967748	1,646206846
NTN4	41,54670519	18,40839676	30,04333954	61,3505202	84,16177576	92,96182547	59,14699324	1,425415219	0,397098554	3,589575448	0,000331217	0,00602086	2,685917923
GPR126	46,74004334	76,263358	73,7427425	109,4685753	126,8037421	133,3268286	134,5098048	0,894290306	0,249296777	3,58725178	0,000334182	0,006055155	1,858695327
ADAMTS12	353,1469941	270,8664094	330,4767349	434,2654469	503,8484975	495,3886752	840,4301033	0,59040005	0,164803872	3,582440415	0,000340399	0,006151773	1,5056642
PROSER1	408,5426011	582,4942688	512,1023785	739,8150965	731,6463706	967,5368941	494,0620513	0,695466281	0,1941658	3,58181658	0,000341213	0,006152794	1,619407744
PRPF39	121,1778901	124,9141209	94,22683764	209,3135395	169,4457085	210,3872892	142,6080672	0,792810083	0,221422396	3,580532496	0,000342895	0,006163326	1,732445638
PSMA4	1319,10789	1672,534334	2236,863189	3140,906044	2738,063105	2607,823841	2496,895288	0,697951314	0,195054963	3,578228936	0,00034593	0,006207958	1,622199567
MGC12916	34,62225433	47,33587738	60,08667908	90,22135323	99,8719739	105,1936446	55,8182394	1,042455435	0,291405328	3,577338285	0,000347111	0,006219207	2,059730287
GPR180	121,1778901	272,1812949	258,0995988	439,0772524	432,0304489	511,2900401	232,0692335	1,079704253	0,30228309	3,571831466	0,000354494	0,006301234	2,113602756
TMEM14C	339,2980924	552,2519027	484,7902516	765,0770754	703,5924453	867,2359771	675,5246362	0,759676134	0,212676322	3,571982657	0,000354289	0,006301234	1,693110502
PTCD3	784,1940605	673,2213671	740,1586377	1021,305719	1024,52935	973,6528036	1094,976405	0,46004747	0,128923219	3,568383354	0,000359191	0,006364587	1,37558708
SLCO1B3	19,04223988	48,65076286	49,16182834	93,83020736	78,55099071	101,5240989	41,3547343	1,203358952	0,337734858	3,563028581	0,000366601	0,00647546	2,302751851
ZFP1	93,48008668	72,31870155	86,03319959	155,1807276	145,8804113	145,5586478	91,30272504	0,83264073	0,233811225	3,561166616	0,000369211	0,006511323	1,780942243
EIF4A2	1772,659422	1884,230897	1560,88805	2262,751539	2602,282106	2655,527936	2316,573178	0,527474585	0,148241494	3,558211465	0,000373389	0,006554138	1,441403831
TPR	1556,270332	1713,295784	1783,481884	2262,751539	2607,892891	2286,126998	1587,235561	0,501717178	0,141475876	3,546309045	0,000390668	0,006804282	1,415897843
PAPSS2	171,3801589	188,028624	236,2498973	324,7968716	302,9823927	338,8213902	181,919937	0,694349023	0,196049338	3,541705516	0,000397549	0,006913415	1,61815412
NTPCR	337,5669797	324,7767142	237,6155036	441,4831551	441,007705	503,9509486	413,8688372	0,625929289	0,176780941	3,540705713	0,000399058	0,006928937	1,543204536
GNAO1	13,84890173	9,204198379	9,559244399	32,47968716	30,29823927	24,46363828	50,53528559	1,448091126	0,409148764	3,539277767	0,000401223	0,006955778	2,728468004
RAB3B	301,2136126	424,7080109	385,1009886	600,2727368	552,101249	615,2605028	457,4020341	0,666091567	0,188437303	3,534817974	0,000408056	0,007052466	1,586768394
CAMSAP2	79,63118495	197,2328224	359,1544681	547,3428763	474,6724153	704,5527825	369,5612755	1,436490806	0,406875969	3,53053735	0,000414716	0,007134647	2,7066171
DDX17	1106,181026	1300,421742	2122,152256	2743,93209	2665,122899	3219,414798	1719,014364	0,929391809	0,263888753	3,521907612	0,000428453	0,007326097	1,904472966
NOP16	495,0982369	619,3110624	499,8119214	814,3980818	737,2571556	848,8882484	872,3148032	0,570601514	0,161999868	3,522234433	0,000427926	0,007326097	1,485142654
RAN	4377,98406	3857,874006	4750,944466	6272,188476	5620,884463	5637,645442	4786,38512	0,432918867	0,122982866	3,52015596	0,000431293	0,007363445	1,349962065
MGEA5	988,465361	1245,196552	1189,443124	1771,947377	1738,221209	1847,00469	1556,801137	0,64522079	0,183400552	3,518096228	0,000434655	0,007409577	1,563978619

DLC1	924,4141905	736,3358703	600,8667908	1352,117347	1209,685257	1631,724673	1035,697949	0,891839747	0,253587615	3,516889993	0,000436635	0,007432052	1,85554083
CREBBP	882,8674853	698,2041913	813,9013802	1077,844433	1250,082909	1289,233737	985,4073484	0,596156995	0,16958179	3,515454069	0,000439003	0,007461053	1,511684421
KAT7	1544,152543	1819,801508	1469,392425	2100,353103	2680,833097	3067,740241	2566,130781	0,699237986	0,199149035	3,511129176	0,000446208	0,007560626	1,623646976
BRWD3	38,08447976	46,02099189	68,28031713	99,84496424	97,62765988	97,85455313	69,33863159	0,941869919	0,268581088	3,506836342	0,000453468	0,007651569	1,921016511
TNRC6A	183,4979479	294,5343481	299,067789	398,1769056	387,1441685	461,1395816	386,6944648	0,673982423	0,192255328	3,50566317	0,000455471	0,007659826	1,595471041
INSR	15,58001445	21,03816772	10,92485074	32,47968716	50,49706545	42,81136699	37,8480451	1,400959327	0,399877275	3,503473229	0,000459233	0,007697391	2,640771231
SLC35D1	76,16895952	86,78244186	86,03319959	138,3394083	151,4911964	124,7645552	115,6862471	0,731942999	0,20893749	3,503167377	0,00045976	0,007697391	1,660874425
TMEM45A	22,50446531	31,55725159	21,84970148	49,32100643	62,84079256	77,06046059	32,65952366	1,309120766	0,374338144	3,497161019	0,000470238	0,00786109	2,477904809
B2M	8394,165562	7973,465567	8576,007832	10454,85041	11194,63833	11451,42908	9185,353037	0,408233754	0,116917336	3,491644329	0,000480057	0,007989571	1,327060141
SNX14	221,5824277	314,2576304	415,1443282	518,4720432	565,5671331	702,1064187	423,3285041	0,905935272	0,259522717	3,490774462	0,000481623	0,00800377	1,873758823
ABCA11P	122,9090029	186,7137385	211,6689831	333,2175313	262,5847404	299,679569	202,1287104	0,772172566	0,221386245	3,487897662	0,000486834	0,00807843	1,707839698
METTL14	39,81559248	67,05915962	68,28031713	109,4685753	134,6588412	112,5327361	95,62564864	1,012581766	0,290460544	3,486125004	0,000490072	0,00812016	2,017518309
TPM1	2241,790968	2504,856845	3934,311873	5527,561574	4671,539633	4383,88398	5789,920111	0,747981869	0,215032184	3,478464739	0,000504295	0,008292967	1,67944188
PRKAB2	57,12671964	78,89312896	43,69940296	139,5423597	118,9486431	99,07773504	67,01088974	0,988653556	0,284371513	3,476626563	0,000507765	0,008327338	1,984332181
TAF15	2802,671488	3201,74615	3842,816248	4711,960541	4908,314762	5297,60087	3902,034881	0,598887344	0,172520854	3,471391029	0,000517769	0,008454392	1,514548044
C21orf7	124,6401156	117,024808	92,8612313	188,8633661	172,8121796	248,3059286	152,771516	0,869167021	0,250416681	3,470883084	0,00051875	0,008458106	1,826607954
MAP3K2	244,086893	235,3645014	170,7007928	327,2027744	343,3800451	440,3454891	381,640529	0,774948055	0,223473758	3,46773626	0,000524862	0,008545364	1,71112844
COPS2	1223,89669	1112,393118	1403,84332	1970,434355	1687,724143	1855,566964	1881,172664	0,559818998	0,161465457	3,467113091	0,000526081	0,008552805	1,474084266
VCL	148,8756936	127,5438918	102,4204757	228,5607615	209,8433609	335,1518445	112,8628791	1,032863408	0,298305697	3,462432728	0,000535316	0,008652859	2,04608121
C20orf194	38,08447976	22,35305321	47,79622199	81,80069359	63,96294957	88,06909782	75,5208213	1,115763928	0,322296848	3,461913872	0,000536349	0,008657101	2,167097297
WASF3	1,731112716	6,574427414	12,29045708	22,85607615	20,19882618	25,6868202	73,90080875	1,670128827	0,483345043	3,455355236	0,000549568	0,008819785	3,1824301
TUBD1	57,12671964	53,91030479	60,08667908	110,6715266	89,77256081	146,7818297	141,303056	1,019469639	0,295362564	3,451587174	0,0005573	0,008880432	2,027173597
NUDT9	276,9780346	236,6793869	284,0461193	419,8300304	411,8316227	431,7832157	268,702543	0,664667299	0,19260381	3,450956135	0,000558604	0,008888614	1,585202667
CLDN1	2371,624421	3593,582024	2874,601351	4381,148913	4613,187468	4193,067602	4668,751729	0,576437953	0,16719645	3,447668613	0,000565447	0,008972084	1,491162984
ATP6V0A1	323,718078	295,8492336	229,4218656	445,0920093	637,3851817	390,1950306	498,1165325	0,797470986	0,231433055	3,445795532	0,000569381	0,009009051	1,738051686
ANXA4	391,2314739	557,5114447	600,8667908	830,0364497	901,0920791	1289,233737	559,3288587	0,961192447	0,2793895	3,44033132	0,000581002	0,009166926	1,946918438
DAAM1	36,35336704	49,96564834	53,25864736	84,20659635	77,42883369	84,39955207	217,2234769	0,799965259	0,232509994	3,440562898	0,000580505	0,009166926	1,741059201
UBE2V1	50,20226877	55,22519027	51,89304102	91,4243046	104,3606019	124,7645552	157,6505444	1,024158747	0,297757133	3,439577539	0,000582623	0,009166926	2,03377312
PSMC6	1810,743901	1776,410287	1887,267966	2691,002229	2619,114462	2867,138407	2246,800014	0,578854708	0,168380668	3,437774146	0,000586517	0,009202414	1,493663025
EGFR	2373,355534	2395,72135	1560,88805	2865,430179	4146,370152	4205,299421	4781,96567	0,825660195	0,240486247	3,433294861	0,000596293	0,009342759	1,7723459
CD109	337,5669797	535,1583915	555,8017815	773,497735	801,2201052	714,3382379	455,770515	0,676450067	0,197223767	3,429860804	0,000603891	0,009448623	1,598202335
KIF11	129,8334537	218,2709901	210,3033768	255,0256918	379,2890694	486,8264018	295,6652992	1,000708836	0,291868565	3,42862835	0,00060664	0,009464942	2,000982897
PAM	472,5937716	453,6354915	469,7685819	660,4203056	680,0271481	824,4246101	433,3724556	0,633056795	0,184736079	3,42681732	0,0006107	0,009476201	1,550847465
PTK2	489,9048987	827,0629686	867,1600276	1153,63037	1218,662513	1734,471954	1124,465762	0,909225682	0,265327084	3,426810663	0,000610715	0,009476201	1,878037255
PLEKHA3	299,4824999	284,0152643	309,9926398	423,4388845	574,5443892	473,3714008	383,2994122	0,720302494	0,210372501	3,423938438	0,000617206	0,009563714	1,64752744
DKK3	1030,012066	721,87213	893,1065481	1253,475334	1256,815851	1237,860097	3034,301065	0,505320838	0,147650249	3,422417797	0,000620669	0,009590117	1,419438988
NEAT1	251,0113439	311,6278594	206,2065577	404,1916625	506,0928115	554,1014071	428,0019279	0,928898271	0,271551863	3,420702992	0,000624595	0,00959877	1,903821568
ARHGAP18	936,5319795	999,3129669	807,0733485	1344,899639	1332,000371	1220,73555	744,67921	0,507093244	0,148983429	3,403688906	0,000664824	0,010051999	1,421183894
CRAT	152,337919	181,4541966	195,281707	239,3873239	305,2267067	330,2591168	203,1521337	0,721729269	0,212000119	3,404381438	0,000663141	0,010051999	1,649157594
MCTP1	173,1112716	194,6030514	199,378526	307,9555524	292,8829796	473,3714008	200,1798683	0,919576787	0,270142004	3,404049618	0,000663947	0,010051999	1,891560325
GTPBP8	86,55563582	140,6927467	122,9045708	184,0515606	212,0876749	190,8163786	148,7211343	0,734580384	0,216063385	3,399837429	0,000674259	0,010153658	1,66391344
RAB30	50,20226877	60,4847322	35,50576491	101,0479156	86,40608978	143,112284	75,46665616	1,175494697	0,345940427	3,397968573	0,000678882	0,010209588	2,258703182
ANKRD10	110,7912138	98,6164112	125,6357835	184,0515606	166,0792375	192,0395605	104,29572	0,694077913	0,204528216	3,393555801	0,000689915	0,010347804	1,617850066
FBXL17	50,20226877	51,28053383	73,7427425	107,0626725	108,84923	95,4081893	85,3043646	0,822962188	0,242491298	3,393780292	0,00068935	0,010347804	1,769034505
FXR1	1121,76104	1546,305328	1984,226016	2449,209002	2445,180125	2584,583385	2106,40238	0,683830274	0,201870611	3,387468198	0,000705409	0,01052399	1,606398997
TMEM56	126,3712283	168,3053418	200,7441324	267,0552056	270,4398394	297,2332051	122,1794482	0,745887222	0,220219425	3,387018299	0,000706567	0,010527282	1,67700527
NEK4	313,3314017	314,2576304	356,4232554	472,7598909	435,3969199	492,9423114	413,3558383	0,508201435	0,150131161	3,385049658	0,000711654	0,010589031	1,422275981
GCNT1	188,6912861	207,7519063	238,98111	298,3319413	340,0135741	346,1604817	171,8833574	0,628573481	0,185761061	3,383774174	0,000714968	0,010617051	1,546035537
SERTAD4	67,51339594	111,765266	121,5389645	200,8928799	172,8121796	207,9409254	90,06838424	0,94106819	0,278126488	3,383597867	0,000715427	0,010617051	1,919949268
CDC25B	893,2541616	963,8110588	701,9216601	1196,936619	1179,387018	1421,337384	1600,992013	0,569668183	0,168608911	3,378636263	0,000728463	0,010782023	1,484182172

PPARG	112,5223266	98,6164112	161,1415484	221,3430533	245,7523852	327,812753	100,6985807	1,093015576	0,32403922	3,373096551	0,000743279	0,010943636	2,133194592
RPL22L1	559,1494074	873,0839605	1341,025428	1885,024807	1588,974326	2261,663359	825,0649402	1,047265904	0,310556402	3,37222449	0,000745636	0,010963979	2,066609639
CDC37L1	83,09341038	84,15267089	133,8294216	173,2249982	160,4684524	232,4045637	150,7267181	0,905573433	0,268811883	3,368799855	0,000754962	0,011072123	1,87328893
SLC18B1	65,78228322	80,20801445	83,3019869	135,9335055	115,582172	146,7818297	107,9648297	0,788152271	0,234252228	3,364545463	0,000766699	0,011229584	1,726861371
LAPTM4B	2731,695866	2834,893101	2742,137536	3306,913334	4472,917842	4793,649921	2966,652578	0,597651001	0,178474864	3,34865644	0,000812044	0,011755805	1,513250682
PMAIP1	432,7781791	345,814882	342,767192	680,870479	599,2318434	570,002772	523,6752067	0,724595685	0,216474788	3,347252089	0,00081617	0,01177436	1,652437476
PNRC2	1402,2013	1793,503798	2313,337144	2579,127751	2934,440581	3629,180739	3137,013139	0,730150267	0,218206328	3,346146169	0,000819432	0,011786809	1,65881186
C12orf4	70,97562137	94,67175476	150,2166977	197,2840257	191,8888487	178,5845595	127,8840144	0,835910224	0,249882197	3,345217204	0,000822182	0,011811237	1,784982863
CCDC80	34,62225433	43,39122093	66,91471079	115,4833321	94,26118885	100,300917	41,17485833	1,085827771	0,324784582	3,343224501	0,000828109	0,011881193	2,122593008
RCN2	486,4426733	422,07824	639,1037684	775,9036378	746,2344117	903,9314345	854,797037	0,647995816	0,194207605	3,336614019	0,000848056	0,012136389	1,566989829
RAB3GAP1	557,4182947	518,0648802	538,048899	779,5124919	818,0524603	1067,837811	685,1927209	0,724349636	0,217157431	3,335596811	0,000851165	0,012149922	1,65215568
PTPN13	221,5824277	344,4999965	529,8552609	656,8114515	641,8738098	918,6096175	324,0971361	1,013770161	0,303992294	3,334854799	0,000853439	0,012158068	2,019180889
EIF2C4	79,63118495	90,72709831	111,9797201	159,9925331	152,6133534	176,1381956	133,43749	0,785089632	0,235504026	3,333656953	0,000857123	0,012188506	1,723199376
WRN	133,2956792	216,9561046	258,0995988	316,376212	369,1896563	523,5218592	339,1749515	0,986086714	0,296025115	3,331091396	0,000865062	0,012285848	1,980804798
NAP1L5	3,462225433	10,51908386	19,1184888	62,55347157	25,80961123	46,48091274	21,97287905	1,992399397	0,599128158	3,32549784	0,000882608	0,012487678	3,978982067
TMEM229B	31,16002889	17,09351128	24,58091417	55,33576331	51,61922246	47,70409465	80,34487798	1,109739797	0,333998757	3,322586604	0,00089187	0,012570275	2,158067212
TLR3	0	15,77862579	31,40894588	52,92986056	51,61922246	62,38227762	24,28812284	1,779901925	0,53619832	3,319484337	0,000901839	0,012648259	3,434028292
EPS8L2	266,5913583	414,1889271	409,6819028	594,2579799	607,0869424	522,2986773	434,4384111	0,656064028	0,197877947	3,315498454	0,000914798	0,012798065	1,575777704
TMEM123	2755,931444	4137,944614	4412,274093	5591,317997	6184,207283	6854,711447	4244,070441	0,720147524	0,217330664	3,313602929	0,000921022	0,012837173	1,647350477
FECH	896,7163871	591,6984672	453,3813058	929,8814139	1135,622894	1235,413733	690,0032794	0,76821056	0,231899306	3,312690205	0,000924033	0,012863176	1,703155971
STK38	238,8935549	220,9007611	344,1327983	452,3097175	387,1441685	401,2036678	288,2004587	0,623779334	0,18841868	3,310602402	0,000930954	0,012943484	1,540906511
SLAIN2	682,0584102	662,7022833	744,2554567	992,4348855	1245,594281	1427,453294	1146,38197	0,811084142	0,245166517	3,308298999	0,000938645	0,013034292	1,754529424
AHCYL1	2156,966445	1922,362576	1951,451464	2624,839903	3145,406099	3587,592554	2328,262747	0,634075116	0,191852806	3,305008293	0,000949736	0,013155774	1,551942511
SAT1	145,4134682	138,0629757	208,9377704	252,619789	309,7153348	321,6968434	172,7345224	0,842377168	0,25485465	3,305323914	0,000948667	0,013155774	1,793002087
ZNF227	157,5312572	143,3225176	139,291847	221,3430533	302,9823927	222,6191084	239,0340239	0,766251135	0,232007799	3,302695596	0,000957603	0,013248411	1,700844367
RSPRY1	386,0381357	403,6698432	443,8220614	624,3317643	553,223406	722,9005113	362,5836719	0,622219504	0,188487962	3,301110034	0,000963031	0,013290775	1,539241395
ADAMTS9	315,0625144	330,0362562	345,4984047	505,2395781	451,107118	585,9041369	336,400754	0,637348792	0,193117958	3,300308255	0,000965787	0,013296139	1,555468083
ADAMTSL3	72,70673409	73,63358703	98,32365667	122,7010404	187,4002207	146,7818297	125,8271373	0,898963351	0,272368585	3,300539786	0,00096499	0,013296139	1,864725602
TBCE	237,1624421	298,4790046	290,874151	470,3539882	372,5561273	401,2036678	338,7240323	0,585672188	0,177580968	3,298057183	0,000973563	0,013354099	1,500738058
BNIP3	4409,144089	3598,841566	2725,75026	5967,841778	7627,301197	4934,315842	3195,69817	0,787987127	0,239127507	3,29525924	0,000983309	0,013379283	1,72666371
SEC14L1	1171,963309	1167,618309	1054,248097	1619,172553	1448,7047	1992,563338	1842,523566	0,576368378	0,174925184	3,29494224	0,000984419	0,013379283	1,491091073
SCO1	131,5645664	195,9179369	208,9377704	292,3171845	325,4255329	256,868202	284,67842	0,698244075	0,212108849	3,291913936	0,000995081	0,013456416	1,622528788
MXI1	251,0113439	193,288166	193,9161007	281,4906221	372,5561273	360,8386647	332,8124178	0,674448817	0,204933835	3,291056443	0,000998119	0,013471128	1,595986908
ANXA5	5816,538727	7331,801452	7095,690556	9511,736533	8429,64346	9198,327994	7885,229487	0,42255569	0,128484068	3,288778872	0,00100623	0,013521523	1,340299752
AP4S1	70,97562137	98,6164112	88,76441227	174,4279496	199,7439478	138,2195563	110,0752939	0,983105152	0,299203829	3,285737205	0,001017159	0,013635751	1,976715372
AKAP13	10,3866763	67,05915962	91,49562496	168,4131927	130,1702132	173,6918318	106,34566	1,466097436	0,446284645	3,285117361	0,001019399	0,013649498	2,762735478
ABHD4	140,22013	215,6412192	221,2282275	303,1437468	300,7380787	407,3195774	260,016126	0,803058318	0,244728108	3,281430661	0,001032819	0,013769517	1,74479594
CHP1	714,9495519	855,9904492	737,427425	1181,298252	1137,867208	1628,055128	979,9125489	0,773333305	0,235709723	3,28087147	0,001034869	0,013774547	1,709214316
RABGAP1L	160,9934826	164,3606853	208,9377704	263,4463514	305,2267067	293,5636594	170,4699058	0,688315244	0,209849571	3,280041223	0,001037919	0,013788778	1,611400651
MEGF9	545,3005056	596,9580092	678,7063523	908,2282892	842,7399146	785,2827889	437,4458921	0,476244546	0,145251944	3,278748177	0,001042686	0,013814341	1,391117755
ZNF567	43,27781791	71,00381607	42,33379662	104,6567697	92,01687483	103,9704627	75,12960711	0,929160952	0,283391064	3,27872354	0,001042777	0,013814341	1,904168241
PLXDC2	110,7912138	211,6965627	218,4970148	270,6640597	341,1357311	415,8818508	141,9216194	0,91899166	0,280827462	3,272442283	0,001066226	0,01402962	1,890793304
LMAN1	74,4378468	151,2118305	259,4652051	532,9074597	408,4651517	256,868202	289,7668485	1,29975197	0,397527751	3,269588012	0,001077042	0,014085556	2,461865543
ABCF2	1085,407673	1455,578229	1386,090438	1916,301543	1761,786506	2391,320642	1371,594801	0,627047189	0,192264993	3,261369524	0,001108754	0,014399708	1,544400782
GGPS1	51,93338149	123,5992354	140,6574533	229,7637129	175,0564936	203,0481977	146,8643069	0,928818134	0,285159751	3,257185243	0,00112523	0,014596802	1,90371582
UTP23	254,4735693	347,1297674	447,9188804	544,9369735	636,2630247	686,2050538	449,1736209	0,828480167	0,254505621	3,255252923	0,001132914	0,014662625	1,775813614
GPR176	413,7359392	474,6736593	397,3914457	658,0144029	641,8738098	943,0732558	618,5169095	0,80203288	0,246579145	3,252638742	0,001143388	0,014724578	1,743556216
PLEKHA8	29,42891618	36,81679352	69,64592348	102,250867	80,79530473	96,63137122	78,05554332	1,025884594	0,315363321	3,253024455	0,001141837	0,014724578	2,03620751
API5	1724,188265	2379,942724	2899,182265	3802,529301	3773,814025	5419,919062	3085,960233	0,891590336	0,27426932	3,25078407	0,001150872	0,014775885	1,855220074
FKTN	195,6157369	265,6068675	307,2614271	411,4093707	396,1214246	436,6759433	322,3539834	0,690190289	0,212439326	3,248881934	0,001158596	0,014858062	1,613496322

MEMO1	1104,449913	1084,780523	1009,183087	1360,538007	1525,011377	1565,67285	1190,25826	0,477213227	0,147095877	3,244232525	0,001177676	0,014983976	1,392052119
NOL10	1036,936517	900,6965557	869,8912403	1336,478979	1447,582543	1733,248772	1432,492638	0,686752316	0,2115965	3,24557502	0,001172137	0,014983976	1,609655903
VTA1	337,5669797	506,2309108	510,7367722	590,6491258	781,021279	714,3382379	624,5796731	0,619385859	0,190952304	3,24366791	0,001180012	0,014983976	1,536221088
LAPTM4A	1609,934826	2398,35112	3053,495782	3421,193714	3802,990107	4898,843566	3495,984748	0,779052418	0,240481295	3,239555148	0,001197163	0,015128156	1,716003407
DDIT4	1147,727731	999,3129669	1175,787061	1378,582277	1749,442779	1714,901044	1009,659699	0,543931479	0,168039536	3,236925615	0,001208249	0,015249883	1,457940132
KCTD15	214,6579768	253,7728982	198,0129197	339,2322881	377,0447554	352,2763913	144,8069317	0,680065801	0,210320856	3,233468214	0,001222969	0,015297138	1,60221283
KPNA5	67,51339594	68,3740451	64,1834981	115,4833321	100,9941309	139,4427382	85,94657359	0,829910196	0,256606752	3,234171312	0,001219963	0,015297138	1,77757471
OPHN1	81,36229767	90,72709831	101,0548694	146,7600679	138,0253122	185,9236509	121,4527323	0,779830047	0,241110031	3,234332656	0,001219273	0,015297138	1,716928603
PDE12	330,6425288	419,448469	383,7353823	560,5753414	632,8965537	540,646406	405,3120596	0,610886605	0,188938001	3,23326489	0,00122384	0,015297138	1,527197456
CHIC1	6,924450865	17,09351128	62,81789176	86,6124991	62,84079256	119,8718276	51,08334127	1,611465837	0,498626647	3,231808503	0,001230095	0,015354314	3,055621478
TGFBR1	206,0024132	272,1812949	318,1862778	466,745134	411,8316227	429,3368519	386,8657567	0,711557568	0,220236281	3,230882595	0,001234086	0,015387041	1,637571124
DDX52	633,5872542	561,4561011	532,5864736	976,7965176	968,4214997	891,6996154	1213,05668	0,716446514	0,221830628	3,229700616	0,001239199	0,01541657	1,643129872
CRISPLD2	6,924450865	5,259541931	5,462425371	20,4501734	15,71019814	24,46363828	18,83666183	1,79334233	0,555548046	3,228059832	0,001246329	0,01548812	3,466169799
CDH4	605,8894507	879,6583879	871,2568466	1091,076898	1357,809982	1625,608764	536,1929639	0,788500457	0,244289744	3,227726401	0,001247783	0,015489049	1,72727819
ZBTB37	60,58894507	40,76144996	55,98986005	84,20659635	96,50550287	159,0136488	52,12101751	1,115869113	0,345912364	3,225872296	0,001255894	0,015572529	2,167255303
NAP1L3	5,193338149	2,629770965	6,828031713	19,24722202	22,4431402	11,00863723	44,13080151	1,866635584	0,579070295	3,223504294	0,001266324	0,015653917	3,646811383
UACA	2435,675592	2064,370208	1709,739141	2751,149798	3172,337867	3131,3457	4343,302554	0,544893704	0,169015604	3,223925428	0,001264463	0,015653917	1,458912849
AFG3L2	392,9625866	489,1373996	525,7584419	639,9701322	740,6236266	620,1532305	537,086701	0,50409856	0,156415134	3,222824717	0,001269332	0,015669974	1,418236922
GABARAPL1	88,28674853	86,78244186	92,8612313	115,4833321	178,4229646	172,4686499	182,0142704	0,800111675	0,248459729	3,220287164	0,001280622	0,015741167	1,741235906
LTN1	197,3468497	243,2538143	275,8524812	335,623434	455,5957461	475,8177646	377,3722417	0,819972026	0,254588678	3,220771771	0,001278459	0,015741167	1,765371762
PAFAH1B1	903,6408379	823,1183122	830,2886563	1115,135926	1251,205066	1612,153763	1141,385385	0,638090435	0,198502294	3,214524241	0,001306609	0,01598959	1,556267905
BTD	486,4426733	515,4351092	420,6067535	645,9848891	646,3624378	692,3209634	455,5205879	0,480638898	0,149591277	3,213014214	0,001313498	0,016038951	1,395361466
MORF4L1	240,6246676	256,4026691	284,0461193	346,4499964	347,8686731	428,1136699	279,7259402	0,520085602	0,161902181	3,212344626	0,001316563	0,016044245	1,434040334
AKR1B1	1284,485636	2007,830132	2753,062387	3202,256564	3162,238454	3988,796222	2057,999429	0,775406296	0,241558625	3,210012875	0,00132729	0,01615469	1,71167203
RFX3	36,35336704	35,50190803	38,23697759	67,36527708	62,84079256	78,2836425	44,83559488	0,919505124	0,28659936	3,208329302	0,001335085	0,016214431	1,891466368
SBDS	1319,10789	1342,498078	1249,529804	1842,921509	2004,17242	2134,45244	1253,885063	0,612960062	0,191173322	3,20630544	0,001344512	0,016293686	1,529393939
TBC1D2	365,2647831	273,4961804	267,6588432	424,6418359	460,0843741	566,3332262	293,6298466	0,682400761	0,212889309	3,205425222	0,001348631	0,01632599	1,604808064
SPEN	441,4337427	504,9160254	372,8105315	552,1546818	742,8679407	829,3173378	725,4105968	0,687205799	0,215004432	3,196240155	0,001392312	0,016800462	1,610161947
TMEM41B	31,16002889	115,7099225	177,5288245	245,4020808	262,5847404	271,5463849	184,3364567	1,254926194	0,392831452	3,194566497	0,00140041	0,016843906	2,386549391
HBS1L	212,9268641	214,3263337	346,864011	366,9001698	449,984961	589,5736826	321,873091	0,859389266	0,269100876	3,193558042	0,001405311	0,016884772	1,814270116
UGGT1	623,2005779	682,4255655	792,0516787	962,3611011	951,5891445	905,1546165	840,1500763	0,42454632	0,132998272	3,192119061	0,001412331	0,016932899	1,342150375
YWHAQ	5498,013987	6855,812907	5936,290772	9372,194173	8161,447934	8011,841538	6669,014725	0,481794802	0,151041049	3,189826913	0,00142358	0,017049574	1,396479894
RAB8B	640,511705	640,3492301	996,8926301	1372,56752	1224,273298	1592,582852	1195,757055	0,878497938	0,275558199	3,188066775	0,001432275	0,017135434	1,838460191
CENPE	6,924450865	53,91030479	105,1516884	262,2434001	127,9258991	168,7991041	79,88314271	1,739394637	0,545865961	3,186486723	0,001440121	0,017192688	3,338950341
TPCN1	316,7936271	370,7977061	432,8972106	475,1657937	658,7061649	688,6514177	866,0260623	0,699950917	0,219767104	3,184966737	0,001447706	0,01724659	1,624449526
NAP1L4	2672,838034	2629,770965	2400,73595	3369,466805	4178,912706	3706,2412	2922,213094	0,547137411	0,17186916	3,183453103	0,001455297	0,017318649	1,461183544
RAB1A	135,0267919	255,0877836	375,5417442	608,6933964	525,1694807	415,8818508	322,4294718	1,013046494	0,318305071	3,182627569	0,001459452	0,017349719	2,018168307
GLS	3720,161227	3176,763326	3338,907508	4776,919916	5455,927383	6970,913729	3268,997551	0,749206094	0,235898329	3,175970328	0,001493362	0,017659402	1,680867605
DHX57	102,1356503	148,5820595	114,7109328	162,3984358	210,9655179	243,4132009	191,2220706	0,748819367	0,236264963	3,169405047	0,001527514	0,018006388	1,680417095
SPAST	70,97562137	113,0801515	113,3453264	174,4279496	138,0253122	173,6918318	145,3110409	0,69429126	0,219166742	3,167867775	0,001535613	0,018038943	1,618089334
CRYZL1	817,0852021	696,8893058	714,2121172	1004,464399	1115,424068	1317,366922	728,2376703	0,626201125	0,197839702	3,165194433	0,001549794	0,018142045	1,543495337
IFI30	839,5896674	615,3664059	467,0373692	922,6637057	1087,370143	1421,337384	1104,618122	0,837797675	0,264699105	3,165094472	0,001550326	0,018142045	1,787319652
NREP	623,2005779	869,1393041	1199,002369	1341,290785	1510,423336	1787,068777	977,6344329	0,784071357	0,247717222	3,165187106	0,001549833	0,018142045	1,721983546
TFDP2	100,4045375	152,526716	187,0880689	215,3282964	256,9739553	380,4095753	307,4298814	0,948527589	0,299839352	3,163452642	0,001559097	0,018213041	1,929901999
WDR36	765,1518206	763,9484655	871,2568466	976,7965176	1279,258991	1144,898272	1143,209276	0,502498162	0,158996247	3,160440385	0,001575308	0,018357899	1,416664527
ATP8A1	10,3866763	30,2423661	55,98986005	66,1623257	80,79530473	107,6400084	38,1859127	1,37475617	0,43575496	3,154883581	0,001605622	0,018595416	2,593240788
ZBTB41	112,5223266	130,1736628	129,7326026	197,2840257	222,187088	269,1000211	174,292654	0,884082571	0,280446763	3,152407826	0,001619299	0,018696001	1,845590612
TRAPPC8	341,0292051	420,7633545	488,8870707	700,1177011	659,8283219	535,7536784	477,6912136	0,597375976	0,189545392	3,151624898	0,001623647	0,018726952	1,512962234
MFSD9	117,7156647	124,9141209	84,66759324	178,0368037	160,4684524	195,7091063	112,2063683	0,708216686	0,224971838	3,148023728	0,001643783	0,018939758	1,63378335
NID2	159,2623699	106,5057241	167,9695801	222,5460046	237,8972861	425,6673061	614,2646076	1,032200605	0,328324035	3,143847218	0,001667425	0,019104578	2,045141416

LAMTOR3	112,5223266	193,288166	319,5518842	356,0736074	363,5788713	532,0841326	376,3488005	0,995892991	0,317037409	3,141247572	0,001682298	0,019214339	1,994314573
LMCD1	136,7579046	111,765266	81,93638056	194,878123	181,7894356	229,9581999	169,320536	0,882032779	0,280911372	3,139897015	0,001690072	0,019275404	1,842970246
ERO1L	181,7668352	365,5381642	680,0719586	845,6748176	781,021279	1006,678715	607,5323172	1,098680902	0,35011757	3,138034185	0,00170085	0,019378654	2,141587914
HIGD1A	401,6181502	554,8816737	603,5980035	707,3354093	801,2201052	1054,38281	559,830825	0,71393585	0,228097818	3,129954759	0,001748332	0,019839156	1,640272885
NR1D2	479,5182224	393,1507593	521,6616229	713,3501662	691,2487182	666,6341432	644,6674555	0,571752602	0,182713811	3,129224877	0,001752681	0,019868436	1,486328083
INIP	275,2469219	336,6106836	300,4333954	395,7710028	465,6951592	440,3454891	324,6961493	0,510225947	0,16315288	3,127287406	0,001764274	0,019929333	1,42427324
HDLBP	6631,892816	7262,112521	6668,255771	8089,848006	10496,65667	11382,93089	8729,812902	0,543448224	0,174168267	3,12024821	0,001806987	0,020311944	1,457451852
PRTG	76,16895952	80,20801445	53,25864736	126,3098945	111,093544	121,0950095	82,67457728	0,776672889	0,249348155	3,114813056	0,001840615	0,020554147	1,713175427
RPL34	128,102341	109,1354951	147,485485	212,9223936	205,3547328	190,8163786	154,8091859	0,663811534	0,213412726	3,110459004	0,001867968	0,020776985	1,584262649
BNIP3L	936,5319795	1264,919834	983,2365667	1586,692865	1692,212771	1554,664213	1578,175986	0,601117217	0,193375386	3,108550826	0,001880073	0,020849695	1,516890785
PLCE1	93,48008668	94,67175476	122,9045708	162,3984358	157,1019814	211,6104711	143,5735436	0,768028561	0,247067311	3,108580241	0,001879886	0,020849695	1,702941129
ITGAV	645,7050432	786,3015187	1024,204757	1431,512138	1170,409762	1804,193323	874,252121	0,842118117	0,27104969	3,106877258	0,001890749	0,020906174	1,792680165
SCLT1	36,35336704	39,44656448	66,91471079	97,43906149	89,77256081	95,4081893	56,25444949	0,976248443	0,314647066	3,102677726	0,001917784	0,02114267	1,967342911
SCAMP2	569,5360837	577,2347269	719,6745426	828,8334983	928,0238473	810,9696091	699,134628	0,459279354	0,148046022	3,102274185	0,0019204	0,021150758	1,374854887
SCARNA9L	185,2290606	181,4541966	238,98111	388,5532946	398,3657386	251,9754743	184,7010073	0,777796554	0,250993403	3,098872501	0,001942586	0,021339168	1,714510279
TRIAP1	555,6871819	666,6469397	740,1586377	884,1692616	833,7626585	979,7687132	858,1413689	0,456257726	0,147303073	3,097408072	0,001952209	0,021417109	1,37197836
ROR1	287,3647109	369,4828206	413,7787218	560,5753414	506,0928115	607,9214113	419,4884983	0,642057117	0,207344857	3,0965664	0,00195776	0,021436137	1,560552742
WBP5	557,4182947	429,9675528	591,3075464	780,7154433	766,4332379	960,1978026	911,3833607	0,668084669	0,215855693	3,095052352	0,001967782	0,021524887	1,58896205
AMMECR1	256,204682	295,8492336	255,3683861	371,7119753	463,4508452	567,5564082	279,4862425	0,795903132	0,257390998	3,092194898	0,001986824	0,021678584	1,736163879
C9orf85	0	21,03816772	23,21530783	69,77117983	39,27549535	34,2490936	27,14184118	1,646579768	0,533497118	3,086389244	0,002026034	0,021990789	3,130905074
DTX3L	294,2891618	289,2748062	393,2946267	425,8447872	498,2377125	567,5564082	341,079725	0,609126641	0,197511077	3,084012546	0,00204229	0,022107493	1,525335542
TTC37	410,2737138	451,0057206	532,5864736	670,0439166	657,5840079	634,8314134	599,6157671	0,491411402	0,159359385	3,083667782	0,002044658	0,022107493	1,405819529
SLC17A5	100,4045375	122,2843499	150,2166977	209,3135395	218,820617	166,3527403	126,2813395	0,666724133	0,216256964	3,083018092	0,002049127	0,022134512	1,587464284
HNMT	70,97562137	71,00381607	73,7427425	111,874478	124,5594281	118,6486457	79,70166897	0,718909583	0,233335055	3,081018336	0,002062939	0,022262307	1,645937533
MSMO1	753,0340316	821,8034267	1326,003759	1586,692865	1517,156278	1821,31787	849,1524424	0,762833873	0,247753494	3,079003493	0,002076942	0,022370443	1,696820403
RCN1	6739,221805	5740,790018	5511,587199	8454,342273	8696,716828	11225,14043	6273,83907	0,657457345	0,213567462	3,078452764	0,002080785	0,022390364	1,577300284
SAP30L	103,866763	148,5820595	118,8077518	156,3836789	204,2325758	286,2245679	182,447332	0,795343059	0,258497661	3,07679016	0,002092425	0,022494074	1,735490008
GSTA4	22,50446531	63,11450317	106,5172947	139,5423597	157,1019814	218,9495626	63,9456435	1,413075321	0,459596364	3,074600742	0,002107845	0,022594974	2,663042256
HMGCS1	822,2785403	698,2041913	760,6427329	1093,482801	1179,387018	945,5196196	706,9694579	0,497949442	0,161946723	3,074773187	0,002106627	0,022594974	1,412204914
RASGRF2	76,16895952	139,3778612	139,291847	208,1105881	209,8433609	309,4650243	91,40830953	1,02863309	0,334735165	3,072975886	0,002119356	0,022696708	2,040090412
ZNF395	405,0803756	389,2061029	378,2729569	500,4277726	520,6808527	544,3159518	453,7114211	0,41796596	0,13610865	3,070825835	0,002134676	0,022828019	1,336042555
ATP5J2	2572,433496	2207,692725	1782,116277	2704,234694	3749,126571	3986,349858	3164,849106	0,670240429	0,218317062	3,070032287	0,002140356	0,022856238	1,591338146
PCNXL4	484,7115606	811,2843428	680,0719586	901,0105809	1156,943877	987,1078047	802,9567649	0,621433965	0,202818503	3,063990491	0,002184059	0,0232566	1,538403516
AGPS	569,5360837	536,4732769	749,7178821	974,3906149	905,5807071	906,3777984	795,6543954	0,585729316	0,191259271	3,062488498	0,002195049	0,023351498	1,500797486
VCAM1	109,0601011	164,3606853	251,271567	323,5939202	461,2065311	247,0827467	408,173517	0,971571808	0,317978583	3,055463035	0,002247133	0,023703561	1,960975904
VCAN	398,1559248	725,8167865	965,4836843	1550,604324	1707,922969	919,8327994	673,2898023	0,998516575	0,326773646	3,055682697	0,002245488	0,023703561	1,997944593
THEM4	202,5401878	132,8034338	120,1733582	233,372567	288,3943516	216,5031988	219,0158411	0,707286047	0,231731106	3,052184321	0,002271826	0,023874357	1,632729786
LRRFIP1	1881,719523	1466,097313	1436,617872	2194,183311	2400,293845	2747,266579	1569,330476	0,618452033	0,202688646	3,051241614	0,002278971	0,023927066	1,535227046
FRMD6	538,3760548	604,847322	790,6860724	1380,98818	1165,921133	818,3087006	939,2946041	0,798408497	0,261695767	3,050903375	0,00228154	0,02393167	1,739181498
AP1M2	228,5068786	181,4541966	200,7441324	335,623434	289,5165086	332,7054806	130,9121354	0,652286928	0,213974566	3,048432065	0,002300389	0,024095533	1,571657578
CCNJL	254,4735693	234,0496159	258,0995988	395,7710028	316,4482768	358,3923008	320,1586169	0,520315502	0,170887859	3,044777456	0,002328525	0,024333632	1,434268873
SLC38A6	117,7156647	144,6374031	155,6791231	209,3135395	203,1104188	366,9545742	301,2539612	0,894861114	0,294230002	3,041365968	0,002355074	0,024519833	1,859430873
C7orf23	20,7733526	63,11450317	133,8294216	192,4722202	158,2241384	217,7263807	104,4507829	1,373572092	0,452191523	3,037589211	0,002384788	0,024783264	2,591113285
APIP	290,8269363	483,8778576	491,6182834	585,8373203	629,5300827	751,0336953	604,8479986	0,630507142	0,207827792	3,03379608	0,002414976	0,024981438	1,548109095
ACTBL2	12,11778901	26,29770965	27,31212685	51,72690918	39,27549535	51,37364039	146,7734767	1,081705887	0,356630108	3,033131145	0,002420304	0,024990528	2,116537261
SLFN12	145,4134682	82,83778541	117,4421455	168,4131927	219,942774	214,056835	173,4506192	0,809884437	0,267001228	3,033261097	0,002419262	0,024990528	1,753071013
MTRF1	5,193338149	18,40839676	23,21530783	33,68263854	44,8862804	41,58818508	24,63533196	1,318588787	0,434958014	3,031531195	0,002433168	0,025054268	2,494220112
UBR4	1789,970549	1665,959907	1917,311305	2245,91022	2679,71094	3630,403921	3458,495494	0,67112326	0,221723009	3,026854378	0,00247113	0,02536608	1,592312234
CEP70	77,90007224	80,20801445	79,20516787	121,498089	139,1474692	125,9877372	78,93865629	0,704081945	0,233030563	3,021414601	0,002515966	0,025668025	1,629107657
MPC2	607,6205634	746,8549542	831,6542627	1024,914573	930,2681613	1438,461931	648,77869	0,632922649	0,209608674	3,019544171	0,002531554	0,025765085	1,550703269

NSD1	365,2647831	382,6316755	385,1009886	477,5716964	556,589877	680,0891443	525,174096	0,596702457	0,19768133	3,018506898	0,002540236	0,025802001	1,512256076
EPG5	278,7091473	358,9637368	509,3711658	680,870479	711,4475444	518,6291316	406,5994184	0,733879651	0,243446319	3,014544041	0,002573658	0,026070765	1,663105455
POGLUT1	83,09341038	163,0457999	120,1733582	214,125345	212,0876749	193,2627424	218,4462608	0,747387656	0,248102583	3,012413845	0,00259179	0,026183604	1,678750299
C11orf54	148,8756936	170,9351128	206,2065577	241,7932267	253,6074843	288,6709317	243,6786841	0,570414001	0,189410222	3,011527013	0,002599373	0,026236616	1,484949636
NIPA1	166,1868208	195,9179369	191,184888	270,6640597	240,1416002	321,6968434	217,320145	0,585040365	0,194829181	3,002837469	0,002674752	0,026876716	1,500080959
DHCR24	2638,21578	3324,0305	3073,979877	4601,289015	4312,44939	3619,395284	3107,141566	0,471538666	0,157068383	3,002123382	0,002681035	0,026915769	1,386587506
FAM129A	3012,136126	2341,811045	2213,647881	2997,75483	4013,955625	3834,675301	1955,471174	0,520006642	0,173331165	3,000075841	0,002699124	0,027073177	1,43396185
TTC39B	29,42891618	44,70610641	64,1834981	58,94461744	103,2384449	114,9790999	54,64700578	0,988393781	0,329802083	2,996930078	0,002727133	0,027281039	1,983974911
GPAM	91,74897397	93,35686927	102,4204757	155,1807276	130,1702132	162,6831946	318,7795021	0,63668702	0,212669421	2,993787336	0,002755379	0,027490169	1,554754744
UBP1	545,3005056	519,3797657	540,7801117	766,2800268	620,5528266	731,4627846	637,1147721	0,39989275	0,133868379	2,987208421	0,002815377	0,027939882	1,319409822
RRP1B	1081,945448	769,2080074	872,622453	1200,545474	1343,221941	1461,702387	1023,333672	0,557952514	0,186801664	2,986871214	0,002818484	0,02794603	1,472178405
LIMS1	1445,479118	1220,213728	923,1498876	1634,810921	1863,902794	1754,042865	1330,760768	0,550826767	0,18450662	2,985403818	0,002832042	0,028055695	1,464924962
LANCL1	574,7294218	383,946561	419,2411472	600,2727368	789,9985351	684,9818719	502,4483366	0,594639052	0,199350808	2,982877555	0,002855522	0,028263379	1,510094727
DLAT	432,7781791	507,5457963	640,4693747	684,4793332	777,654808	880,6909782	673,6058022	0,565381188	0,189698349	2,98042229	0,002878513	0,028465855	1,479778444
FAM160A1	72,70673409	123,5992354	135,1950279	221,3430533	185,1559067	160,2368308	112,9019391	0,762901823	0,256083708	2,97911112	0,002890859	0,028490116	1,696900324
MIS18BP1	432,7781791	516,7499947	472,4997946	621,9258616	686,7600902	835,4332474	476,0999546	0,591018215	0,198407101	2,978815845	0,002893646	0,028490116	1,506309485
NAA15	192,1535115	500,9713689	797,5141041	1216,183842	788,8763781	1473,934207	783,1310622	1,220857701	0,40982385	2,978981584	0,002892082	0,028490116	2,330852483
PIAS2	219,851315	419,448469	517,5648039	623,128813	622,7971406	724,1236932	444,7371961	0,763797624	0,256384099	2,97911464	0,002890826	0,028490116	1,697954294
NSMCE4A	245,8180057	287,9599207	304,5302144	389,7562459	386,0220115	518,6291316	331,4778208	0,623945536	0,209839491	2,97344191	0,002944801	0,028867277	1,541084037
CHST9	31,16002889	30,2423661	27,31212685	48,11805506	80,79530473	70,94455102	24,10616206	1,175138516	0,396316785	2,965149496	0,003025359	0,02942587	2,258145608
MYOF	1196,198887	1109,763347	1209,92722	1539,777762	1735,976895	2989,456598	1237,930674	0,833455809	0,281174579	2,964193318	0,003034776	0,029466439	1,781948706
TPRKB	415,4670519	543,0477044	503,9087404	670,0439166	781,021279	715,5614198	743,164025	0,565097366	0,190666569	2,963798896	0,003038668	0,029478753	1,479487355
PLS1	199,0779624	191,9732805	269,0244495	299,5348927	323,1812189	458,6932178	207,5625459	0,710348373	0,239818326	2,962027069	0,003056209	0,029618031	1,636199169
CHMP3	955,5742194	1049,278615	1062,441735	1163,253981	1604,684524	1390,757836	1200,092034	0,438717966	0,148159989	2,9611096	0,003065328	0,02966055	1,355399332
PSAT1	1270,636734	1551,56487	1511,726221	1957,201889	1934,598685	2330,161546	1253,168675	0,52090885	0,175977115	2,960094274	0,003075449	0,029732871	1,434858876
CLTCL1	57,12671964	38,131679	15,02166977	80,59774222	61,71863555	90,51546165	59,1137428	1,099701845	0,371614542	2,959254069	0,003083847	0,029775298	2,143103975
DDOST	2672,838034	2419,389288	2602,845689	3696,66958	3541,527524	3505,639366	4683,094536	0,481676567	0,1628314	2,958130714	0,003095108	0,029775298	1,396365451
FAM126B	60,58894507	113,0801515	105,1516884	143,1512138	160,4684524	163,9063765	126,8582843	0,731569403	0,247335324	2,95780397	0,003098391	0,029775298	1,660444386
IL13RA1	67,51339594	155,156487	255,3683861	315,1732606	295,1272936	386,5254849	351,5690899	1,053421517	0,356034797	2,958760005	0,003088796	0,029775298	2,075446175
MED6	602,4272253	767,8931219	655,4910445	931,0843653	888,748352	989,5541685	1042,490766	0,469849063	0,158934714	2,956239393	0,003114153	0,02990119	1,384964564
C12orf23	292,5580491	320,8320578	367,3481062	488,3982588	417,4424077	653,1791421	487,9063643	0,666717315	0,225849014	2,952048812	0,00315673	0,030232487	1,587456781
ZNF614	48,47115606	34,18702255	40,96819028	70,97413121	69,57373462	75,83727868	56,48181929	0,819736694	0,27768182	2,952071887	0,003156494	0,030232487	1,765083818
NAA60	379,1136849	499,6564834	650,0286191	756,6564157	759,7002958	810,9696091	550,522967	0,603426588	0,204678523	2,948167595	0,003196637	0,030505457	1,519320868
PPP2R5A	93,48008668	138,0629757	177,5288245	257,4315945	203,1104188	288,6709317	187,3866579	0,866326047	0,29387331	2,947957568	0,00319881	0,030505457	1,823014513
ABAT	12,11778901	7,889312896	20,48409514	30,07378441	30,29823927	34,2490936	72,17388386	1,221251067	0,415296943	2,940669534	0,003275037	0,031100396	2,331488102
NMT2	105,5978757	94,67175476	116,0765391	178,0368037	155,9798244	170,0222861	129,7467473	0,672188778	0,228739189	2,938669063	0,003296248	0,03124899	1,593488688
LMBRD1	36,35336704	38,131679	62,81789176	83,00364497	90,89471782	73,39091485	55,78589625	0,840503856	0,286147447	2,937310337	0,003310726	0,031306984	1,790675419
EIF1AX	3403,3676	3372,681263	2776,277695	4513,473564	4154,225251	5466,399974	3024,794874	0,565331214	0,192583397	2,935513765	0,003329958	0,031409532	1,479727186
SMURF2	1152,921069	995,3683104	1209,92722	1656,464045	1527,255691	2075,739708	1551,067799	0,647308598	0,220688392	2,933133875	0,003355592	0,031519526	1,566243582
TMED5	792,8496241	1091,354951	613,1572479	1432,715089	1853,803381	1497,174663	1090,769564	0,937520716	0,31963164	2,933128636	0,003355648	0,031519526	1,915234062
LMAN2L	458,7448698	441,8015222	449,2844867	504,0366267	715,9361724	640,947323	680,4370502	0,464216782	0,15850236	2,928768895	0,003403073	0,031858439	1,379568203
EYA4	95,2111994	181,4541966	154,3135167	218,9371505	226,675716	245,8595647	159,852307	0,670914041	0,229155341	2,927769595	0,003414029	0,031934393	1,592081335
CCNJ	50,20226877	77,57824348	69,64592348	104,6567697	106,604916	122,3181914	95,99247248	0,743368948	0,253925778	2,927504851	0,003416937	0,031935003	1,674080552
TMEM126B	36,35336704	219,5858756	319,5518842	581,0255148	456,7179031	440,3454891	252,4734107	1,356066758	0,464001155	2,922550393	0,003471774	0,032339895	2,559863279
FBXO21	240,6246676	343,185111	305,8958208	363,2913157	485,8939854	433,0063976	404,1617446	0,522710734	0,17891893	2,921494868	0,00348356	0,032422796	1,436652094
LCA5	62,32005779	74,94847251	84,66759324	99,84496424	151,4911964	116,2022818	89,14738544	0,722455835	0,247401283	2,920178207	0,003498313	0,032479377	1,649988347
ANKS3	103,866763	81,52289993	81,93638056	122,7010404	155,9798244	133,3268286	105,0346597	0,634358373	0,217346585	2,918648911	0,003515519	0,032584379	1,552247247
FAF2	159,2623699	194,6030514	267,6588432	336,8263854	280,5392525	329,0359349	309,0761878	0,600427476	0,205738563	2,918400267	0,003518324	0,032584379	1,516165746
ORC2	81,36229767	207,7519063	316,8206715	400,5828083	361,3345572	423,2209423	255,1050199	0,961287717	0,329644249	2,916136778	0,003543951	0,032794683	1,947047009
ARID4A	93,48008668	124,9141209	178,8944309	193,6751716	227,797873	335,1518445	257,8450438	0,924086726	0,317339567	2,911980802	0,003591447	0,033125055	1,897482691

CRYAB	1,731112716	5,259541931	5,462425371	8,420659635	28,05392525	7,339091485	66,16895957	1,777198588	0,610517228	2,910972051	0,003603062	0,033150535	3,427599598
IFIT1	22,50446531	64,42938865	103,786082	104,6567697	125,6815851	195,7091063	198,34868	1,144439936	0,393116798	2,91119571	0,003600484	0,033150535	2,210602969
ZNF280B	107,3289884	113,0801515	83,3019869	151,5718734	152,6133534	172,4686499	80,62821965	0,651564063	0,223802173	2,911339308	0,00359883	0,033150535	1,570870294
ANKRD12	410,2737138	677,1660236	690,9968094	748,2357561	961,6885576	1059,275538	944,634456	0,636118208	0,21860247	2,909931474	0,00361508	0,033233887	1,554141872
IDH1	798,0429622	1033,499989	1265,91708	1356,929153	1479,002939	1943,636062	912,7807414	0,624554508	0,214691956	2,909072699	0,003625026	0,033270863	1,541734677
SRFBP1	218,1202023	310,3129739	309,9926398	358,4795102	429,7861349	468,4786731	315,2537993	0,579064882	0,199089394	2,908567202	0,003630891	0,033297496	1,49388064
SUV39H2	207,733526	243,2538143	304,5302144	407,8005166	351,2351442	363,2850285	251,2565873	0,566699576	0,194863843	2,90818229	0,003635364	0,033311317	1,481131338
ATL3	1628,977066	1497,654565	1428,424234	1802,021162	2115,265964	2573,574747	1677,476008	0,511267578	0,17589164	2,906719035	0,003652411	0,033371801	1,425301942
SPCS2	3810,179089	3832,891182	3501,414663	4550,765057	5268,527162	5304,939962	3634,004678	0,440607026	0,151690338	2,904647935	0,003676664	0,033498495	1,35717525
RSL1D1	2155,235332	1706,721357	1745,244906	2642,884174	2527,097587	2269,002451	2210,311872	0,408940818	0,140818656	2,904024436	0,003683994	0,033538079	1,327710692
SLC25A23	441,4337427	523,3244221	456,1125184	613,505202	731,6463706	924,7255271	419,4924283	0,674952652	0,232476397	2,903316897	0,003692328	0,033586732	1,596544375
CCAR1	1225,627803	1247,826323	1062,441735	1521,733491	1738,221209	1970,546064	1264,468195	0,565014075	0,19463718	2,902909266	0,003697137	0,033603269	1,479401942
RNLS	39,81559248	13,14885483	20,48409514	50,52395781	51,61922246	45,25773082	75,56331184	1,04952006	0,361697757	2,901649346	0,003712038	0,033684196	2,069841161
ZFR	576,4605345	573,2900705	759,2771265	873,3426993	857,3279557	985,8846228	862,8279673	0,50782506	0,175134067	2,899636089	0,003735961	0,03381933	1,421904981
OTUD4	493,3671242	603,5324366	438,359636	834,8482552	655,3396939	697,2136911	542,5753524	0,509600343	0,175785727	2,898985891	0,003743717	0,033862255	1,423655758
ZNF451	46,74004334	77,57824348	84,66759324	111,874478	130,1702132	110,0863723	110,3228164	0,737909954	0,254728169	2,896852584	0,003769268	0,034065937	1,667757991
SSBP1	2778,43591	2941,398825	3572,426192	4270,477386	4228,287614	3766,177114	3370,500905	0,400084871	0,138150872	2,895999609	0,003779529	0,034115076	1,319585538
USP53	126,3712283	165,6755708	174,7976119	198,4869771	294,0051366	240,9668371	263,3940091	0,647105098	0,223455931	2,895895821	0,003780779	0,034115076	1,566022671
EVI5	53,66449421	84,15267089	117,4421455	192,4722202	127,9258991	133,3268286	146,2665315	0,817535429	0,282585456	2,893055569	0,003815138	0,034287408	1,762392705
TPI1	4163,326083	2945,343481	4256,59497	6013,553931	5597,319166	7896,862438	5060,918716	0,779507727	0,269617346	2,891163116	0,003838188	0,034466994	1,716545058
APOL3	41,54670519	36,81679352	28,6777332	58,94461744	52,74137947	68,49818719	168,1133273	0,760787986	0,263320515	2,889208941	0,003862124	0,034630646	1,694415846
CCBE1	53,66449421	42,07633545	53,25864736	97,43906149	69,57373462	70,94455102	493,4945798	0,68132968	0,235821782	2,889171957	0,003862578	0,034630646	1,603617071
RRP15	166,1868208	243,2538143	286,777332	372,9149267	390,5106395	404,8732136	309,8716183	0,742390102	0,256997624	2,888704142	0,003868328	0,034654547	1,672945099
NEGR1	6,924450865	30,2423661	47,79622199	70,97413121	54,98569349	59,93591379	97,66796254	1,09209311	0,378430689	2,885847109	0,003903617	0,034755659	2,131831053
TMED2	5473,778409	4871,650713	4296,197554	6025,583444	7399,503324	8479,097029	6817,042982	0,58127523	0,201417114	2,885927713	0,003902618	0,034755659	1,496171163
BTN3A1	25,96669075	28,92748062	45,06500931	50,52395781	65,08510658	75,83727868	57,12700805	0,925942795	0,321262412	2,882200843	0,003949079	0,035098067	1,899925429
LMBR1L	323,718078	262,9770965	238,98111	394,5680515	435,3969199	388,9718487	466,8198501	0,566225148	0,196511961	2,881377527	0,003959411	0,035162072	1,48064435
SRGN	709,7562137	704,7786187	820,7294119	1081,453287	1063,804846	1499,621027	2118,011051	0,705074573	0,244840167	2,879734086	0,003980107	0,035317949	1,63022893
FAM117B	41,54670519	84,15267089	60,08667908	92,62725598	92,01687483	138,2195563	104,8576519	0,778154768	0,270318825	2,87865548	0,003993744	0,035383057	1,714936037
RPL35A	102,1356503	272,1812949	424,7035726	584,6343689	622,7971406	433,0063976	353,055063	1,033654767	0,35936795	2,876313168	0,004023503	0,035618624	2,047203851
STAU1	195,6157369	198,5477079	223,9594402	274,2729138	280,5392525	347,3836636	423,5775432	0,543612211	0,189058391	2,875366743	0,004035585	0,03566936	1,457617526
AK4	652,6294941	760,003809	629,544524	1103,106412	1115,424068	894,1459792	740,0395438	0,60772466	0,211495407	2,873465042	0,00405996	0,035856597	1,523853973
ERMP1	429,3159537	403,6698432	480,6934326	548,5458276	671,049892	631,1618677	549,4295269	0,494605791	0,172272972	2,871058564	0,004090997	0,03607399	1,408935718
QSER1	1997,704075	2398,35112	2459,457023	2930,389553	3379,936914	4376,544889	2679,104946	0,640187169	0,223034659	2,870348365	0,004100198	0,036126764	1,558531343
SLC35B4	387,7692485	423,3931254	326,3799159	489,6012102	572,3000751	708,2223283	419,2554332	0,637852768	0,222361591	2,868538421	0,004123731	0,036248823	1,556011548
ZNF627	29,42891618	26,29770965	31,40894588	40,9003468	68,45157761	64,82864145	31,73831753	1,002208612	0,349763861	2,865386406	0,004165007	0,036468968	2,003064131
APH1B	117,7156647	105,1908386	87,39880593	140,745311	159,3462954	203,0481977	134,6530105	0,701454928	0,244878303	2,864504199	0,004176626	0,036485395	1,626143898
CASP1	6,924450865	10,51908386	8,193638056	19,24722202	20,19882618	25,6868202	62,40616703	1,322963515	0,461797909	2,864810534	0,004172588	0,036485395	2,501794891
DNAJC13	547,0316184	919,1049524	1164,86221	1591,504671	1084,003672	1784,622413	994,3393107	0,759815031	0,2652397	2,864635393	0,004174896	0,036485395	1,693273515
AHSA1	798,0429622	802,0801445	1005,086268	1201,748425	1130,012109	1116,765088	1187,935663	0,403601087	0,140952423	2,863385227	0,004191406	0,036557503	1,322805626
FAM102B	112,5223266	127,5438918	142,0230596	184,0515606	189,6445347	294,7868413	125,5053723	0,80360555	0,280681531	2,863051047	0,00419583	0,036557503	1,745457888
GDE1	403,3492629	426,0228964	596,7699717	606,2874937	746,2344117	729,0164208	561,81558	0,54380838	0,190086058	2,860853587	0,004225021	0,036651651	1,457815737
GSR	960,7675576	1034,814875	1066,538554	1267,910751	1385,863907	1542,432394	1077,859601	0,453938589	0,158665191	2,860984112	0,004223282	0,036651651	1,369774672
PRKCI	386,0381357	489,1373996	525,7584419	626,7376671	732,7685276	708,2223283	633,1774059	0,559417346	0,195780522	2,857369771	0,004271679	0,036885503	1,473673931
PSMB9	209,4646387	253,7728982	243,077929	301,9407955	356,8459292	307,0186604	564,7143451	0,447686559	0,156671136	2,857492271	0,00427003	0,036885503	1,363851491
RNF130	640,511705	568,0305285	577,6514829	730,1914855	1061,560532	890,4764335	815,5081345	0,587593321	0,205710671	2,856406608	0,00428466	0,036940907	1,502737814
RSPH3	38,08447976	44,70610641	76,47395519	104,6567697	107,727073	113,755918	71,85606796	1,025825002	0,3591314	2,856405769	0,004284672	0,036940907	2,036123405
YY1	1047,323193	1186,026705	1289,132387	1522,936442	1432,994502	1636,617401	1444,495027	0,381301357	0,133540009	2,855334225	0,004299156	0,036997841	1,302516237
KLHL15	60,58894507	60,4847322	51,89304102	99,84496424	92,01687483	103,9704627	79,85249971	0,775699113	0,27206516	2,851151954	0,004356115	0,037327683	1,712019475
C10orf118	223,3135404	214,3263337	189,8192816	268,2581569	326,5476899	463,5859455	299,1952186	0,754949755	0,264838808	2,850600942	0,00436367	0,037363922	1,687572818

STX3	368,7270086	378,687019	368,7137125	504,0366267	460,0843741	675,1964166	291,757007	0,553816559	0,19442261	2,84851931	0,004392319	0,037551986	1,46796397
ZNF718	0	0	0	3,608854129	4,48862804	2,446363828	24,00513056	3,720508019	1,30669205	2,847272253	0,004409563	0,037670749	13,18209729
C2orf69	93,48008668	110,4503805	155,6791231	186,4574633	166,0792375	222,6191084	170,1006713	0,669707415	0,235302375	2,846156631	0,004425042	0,037688344	1,590750323
TRIM5	278,7091473	218,2709901	218,4970148	310,3614551	399,4878956	441,568671	265,2145953	0,68944838	0,242199711	2,846611078	0,004418731	0,037688344	1,612666791
OSBPL1A	225,0446531	228,790074	263,5620241	381,3355863	364,7010283	381,6327572	218,8067888	0,651440573	0,229327141	2,840660591	0,00450202	0,038170333	1,570735838
ROBO1	257,9357947	403,6698432	465,6717628	532,9074597	570,0557611	603,0286837	408,4945207	0,593134396	0,208882689	2,839557446	0,004517616	0,038244837	1,508520598
N4BP2	88,28674853	77,57824348	90,13001862	159,9925331	125,6815851	143,112284	79,07582765	0,745193573	0,262567035	2,838107884	0,004538184	0,038361144	1,676199158
COL5A2	199,0779624	273,4961804	434,262817	470,3539882	456,7179031	587,1273188	2627,404329	0,735638126	0,259315022	2,836851181	0,004556084	0,038466874	1,665133821
DDX19A	604,158338	883,6030444	670,5127142	1150,021516	1086,247986	905,1546165	850,0197061	0,539897895	0,190799891	2,829655148	0,00465982	0,039007764	1,453869618
CDV3	1478,37026	1967,068682	1670,136557	2362,596503	2203,916368	2605,377477	1571,640879	0,486643867	0,172169654	2,826536816	0,004705433	0,039330982	1,401181521
SPATS2L	339,2980924	406,2996142	434,262817	638,7671809	566,6892901	479,4873103	472,6423926	0,511673475	0,181090684	2,825509644	0,004720547	0,039427971	1,425703002
NAP1L1	2880,57156	2790,186994	3982,108095	4432,875822	4516,681966	5921,423646	5006,749774	0,623305992	0,220639223	2,825000851	0,004728049	0,039461295	1,540401028
HMBOX1	244,086893	227,4751885	148,8510913	269,4611083	353,4794582	330,2591168	217,1053516	0,623677825	0,220971928	2,822430122	0,004766121	0,039631828	1,540798095
MYO1B	1822,86169	1631,772884	1518,554253	2549,053967	2487,822091	2051,27607	1622,662492	0,511748947	0,181301321	2,822643229	0,004762954	0,039631828	1,425777587
GOLT1B	292,5580491	477,3034302	412,4131155	590,6491258	625,0414546	523,5218592	523,1972379	0,552698504	0,195869664	2,821766743	0,00477599	0,039684521	1,466826773
PELI1	152,337919	160,4160289	166,6039738	232,1696156	239,0194431	227,511836	180,707886	0,542043379	0,192170451	2,820638533	0,004792817	0,039761225	1,456033329
C14orf105	384,307023	545,6774753	651,3942254	748,2357561	791,1206921	1066,614629	360,3289618	0,718575394	0,254818116	2,819954111	0,004803052	0,039791654	1,645556308
MFSD12	20,7733526	19,72328224	12,29045708	36,08854129	33,6647103	50,15045848	24,12189896	1,191318827	0,422854595	2,817322616	0,004842586	0,040001181	2,283614024
ZNF83	20,7733526	27,61259514	23,21530783	43,30624955	40,39765236	59,93591379	37,86030021	0,992017447	0,35221398	2,816519226	0,004854714	0,040071899	1,988964391
CCDC14	103,866763	172,2499982	223,9594402	281,4906221	318,6925909	259,3145658	188,1667749	0,774806138	0,275529349	2,812063906	0,004922472	0,040423284	1,710960126
SOGA2	1365,847933	1312,255712	1216,755251	1530,154151	1840,337497	2184,602899	1721,134014	0,512476149	0,182455975	2,808766057	0,004973177	0,040661333	1,426496442
ZNF367	604,158338	312,9427449	390,563414	633,9553754	639,6294957	757,1496048	412,8250918	0,639353078	0,22775957	2,807140349	0,004998346	0,040807717	1,557630542
PPAP2B	167,9179335	123,5992354	117,4421455	162,3984358	212,0876749	236,0741094	2333,217654	0,587271939	0,209253422	2,806510568	0,005008127	0,040857878	1,502403094
AKTIP	330,6425288	361,5935077	376,9073506	507,6454808	429,7861349	529,6377688	351,733664	0,454042977	0,161970093	2,803251943	0,005059013	0,0411833	1,369873787
CES2	166,1868208	182,7690821	195,281707	247,8079835	295,1272936	325,3663892	218,6734172	0,672068536	0,239937103	2,801019632	0,005094142	0,041277992	1,593355884
HAUS6	1019,62539	1091,354951	1209,92722	1432,715089	1504,812551	1990,116974	687,5332718	0,568805825	0,203068189	2,801058246	0,005093532	0,041277992	1,483295281
LOC100190986	8,655563582	17,09351128	60,08667908	92,62725598	51,61922246	85,62273399	50,46593566	1,401179173	0,500224047	2,801103188	0,005092823	0,041277992	2,641173677
NIPBL	619,7383525	995,3683104	920,4186749	1014,08801	1392,596849	1538,762848	1324,530859	0,636315077	0,22717573	2,800981759	0,00509474	0,041277992	1,554353963
OCRL	76,16895952	106,5057241	117,4421455	193,6751716	196,3774768	136,9963744	113,6722829	0,80646447	0,287918919	2,801012431	0,005094256	0,041277992	1,748920209
TMEM14B	657,8228322	894,1221282	921,7842813	1212,574987	1081,759358	1225,628278	924,5246518	0,506858997	0,180891187	2,802010455	0,005078523	0,041277992	1,420953158
PIGB	77,90007224	134,1183192	152,9479104	184,0515606	187,4002207	242,190019	147,6699988	0,739299079	0,263999603	2,800379507	0,005104256	0,041305838	1,669364595
TARSL2	169,6490462	140,6927467	103,786082	188,8633661	212,0876749	238,5204733	167,8232246	0,633752942	0,226324488	2,800196067	0,005107158	0,041305838	1,551595979
SGPL1	373,9203467	289,2748062	447,9188804	635,1583267	557,712034	464,8091274	353,603241	0,577879946	0,206737351	2,795237261	0,005186164	0,041794379	1,492654166
SYPL1	668,2095085	649,5534285	745,6210631	941,9109277	960,5664006	1292,903283	749,0998082	0,630681897	0,225761967	2,793570176	0,005212972	0,041940433	1,548296631
C5orf28	195,6157369	169,6202273	161,1415484	223,748956	259,2182693	259,3145658	230,8202346	0,500787598	0,179460638	2,790514978	0,005262427	0,042189592	1,414985823
TMEM30A	1191,005549	1610,734716	2047,043908	2338,537476	2501,287975	2261,663359	1679,03728	0,54952905	0,196972298	2,789879873	0,00527276	0,042189592	1,463607842
EGLN3	46,74004334	49,96564834	51,89304102	83,00364497	141,3917833	69,72136911	49,69919448	0,985475473	0,353415278	2,788434836	0,00529634	0,042348103	1,979965749
C3orf14	176,5734971	289,2748062	285,4117256	386,1473918	353,4794582	397,5341221	227,7431571	0,591365749	0,212260753	2,786034352	0,005335722	0,0424989	1,506672386
CORO2A	10,3866763	10,51908386	10,92485074	32,47968716	17,95451216	24,46363828	99,45622393	1,228299549	0,440946715	2,785596325	0,005342936	0,0424989	2,342906773
DCBLD2	9076,223972	7713,118242	7271,853775	11409,9938	10614,48316	14126,52793	9688,649976	0,58740454	0,210853736	2,785838899	0,00533894	0,0424989	1,502541189
ZNF280D	38,08447976	59,16984672	64,1834981	91,4243046	78,55099071	114,9790999	60,87444265	0,80390762	0,288610332	2,785442965	0,005345464	0,0424989	1,745823388
RPS6KA6	27,69780346	11,83396934	24,58091417	55,33576331	35,90902432	39,14182125	36,50322107	1,046168451	0,375655956	2,784911126	0,00535424	0,042511728	2,065038173
SEPT10	1374,503497	1049,278615	1203,099188	1644,434532	1564,286872	2056,168798	1150,832682	0,538375597	0,193336384	2,784657422	0,00535843	0,042511728	1,452336338
PCYOX1	389,5003612	544,3625898	482,059039	591,8520772	668,805578	752,2568772	576,9750818	0,504500208	0,181263556	2,783241267	0,005381877	0,042667611	1,418631816
BTRC	178,3046098	215,6412192	243,077929	294,7230872	332,158475	373,0704838	258,204842	0,647178673	0,232923121	2,778507652	0,005460923	0,043118021	1,566102537
FTL	15907,19475	17689,1544	20947,03569	30622,33024	24969,11563	33176,36306	20131,70061	0,702601587	0,25289521	2,778232082	0,005465557	0,043118021	1,627436877
IFNGR2	270,0535838	289,2748062	396,0258394	413,8152735	475,7945723	535,7536784	382,8045701	0,574781204	0,206883235	2,778287976	0,005464616	0,043118021	1,489451558
FAM63B	34,62225433	53,91030479	110,6141138	104,6567697	113,337858	212,8336531	121,728294	1,102816753	0,397019179	2,777741758	0,00547381	0,04312257	2,147736127
PSIMCT-1	15,58001445	10,51908386	20,48409514	26,46493028	24,68745422	47,70409465	223,9163225	1,084536331	0,390552004	2,776931929	0,005487467	0,043169611	2,120693801
SLC7A2	128,102341	159,1011434	154,3135167	202,0958312	218,820617	298,456387	219,2280151	0,700307024	0,252167429	2,777150985	0,00548377	0,043169611	1,624850545

MRPL3	5676,318597	4214,207972	3866,031556	6032,801153	6979,816603	7806,346976	5575,748214	0,598059166	0,215434491	2,776060432	0,005502198	0,043255208	1,513678869
SH3BGRL2	36,35336704	40,76144996	50,52743468	90,22135323	83,03961875	59,93591379	37,23624341	0,863627025	0,311170841	2,775411162	0,005513196	0,043310354	1,819607171
ERCC8	1,731112716	39,44656448	51,89304102	78,19183946	78,55099071	106,4168265	56,72167145	1,47502539	0,53159708	2,774705592	0,00552517	0,043344804	2,779885362
MICALCL	36,35336704	43,39122093	53,25864736	78,19183946	65,08510658	88,06909782	80,7566588	0,786780913	0,283675963	2,773519844	0,005545346	0,043442409	1,725220678
ARL6IP5	1206,585563	1243,881667	899,9345798	1380,98818	1620,394723	2246,985176	957,7365284	0,647707203	0,233634652	2,772307943	0,005566035	0,043543761	1,566676382
ZNF195	76,16895952	82,83778541	57,35546639	108,2656239	108,84923	149,2281935	80,75146055	0,759996494	0,274562715	2,768025125	0,005639711	0,043972332	1,693486509
EFR3A	1094,063237	1317,515254	1544,500774	1888,633661	1833,604554	2178,486989	1142,209368	0,57604428	0,20831923	2,765199739	0,005688795	0,044257565	1,490756142
KIAA1432	384,307023	385,2614464	335,9391603	514,8631891	495,9933985	655,625506	576,182277	0,592269208	0,214269468	2,764132533	0,005707435	0,044341167	1,507616205
CCNH	212,9268641	353,7041948	445,1876677	530,501557	500,4820265	622,5995943	551,7056734	0,704423689	0,254877504	2,763773485	0,005713719	0,044359307	1,629493603
EFHA2	6,924450865	14,46374031	30,04333954	28,87083303	43,76412339	58,71273188	27,13311884	1,324138115	0,479356317	2,762325371	0,005739125	0,044495055	2,503832608
LIPC	12,11778901	6,574427414	5,462425371	20,4501734	25,80961123	17,1245468	20,3724915	1,438846811	0,52103016	2,761542269	0,005752907	0,04450975	2,711040773
MPP5	278,7091473	362,9083932	375,5417442	538,9222166	396,1214246	740,025058	357,7588989	0,717190106	0,259676348	2,761861489	0,005747285	0,04450975	1,643976989
PPT2	169,6490462	199,8625934	173,4320055	218,9371505	268,1955254	289,8941136	283,4421297	0,514459383	0,186329704	2,761016477	0,005762177	0,044550788	1,428458757
HAVCR1	2364,699971	2687,625927	3430,403133	4455,731898	4184,523491	3783,30166	1742,616456	0,550150576	0,19943755	2,758510498	0,005806544	0,044789416	1,464238512
PDHA1	782,4629478	850,7309073	893,1065481	1098,294607	1107,568969	1174,254638	1092,139896	0,419111142	0,151923364	2,758701038	0,00580316	0,044789416	1,337103499
SLC25A16	100,4045375	76,263358	121,5389645	174,4279496	209,8433609	123,5413733	123,6280677	0,770608144	0,279584996	2,756257147	0,005846701	0,045018397	1,705988763
ACBD6	147,1445809	261,6622111	385,1009886	466,745134	445,496333	431,7832157	273,3533435	0,754312306	0,273871289	2,754258431	0,00588253	0,045232265	1,686827336
LCN2	5,193338149	6,574427414	17,75288245	26,46493028	22,4431402	19,57091063	277,6149116	1,17783857	0,427907871	2,752551772	0,00591328	0,045401145	2,262375763
METTL6	152,337919	138,0629757	234,8842909	259,8374973	311,9596488	259,3145658	230,3691422	0,660216741	0,239872349	2,752367014	0,005916617	0,045401145	1,580320023
KLF10	460,4759825	590,3835817	628,1789176	804,7744708	664,31695	995,6700781	569,9084092	0,551459076	0,200398967	2,751805979	0,005926762	0,045447951	1,465567155
ARPC1A	1033,474292	990,1087685	1080,194617	1361,740958	1202,952315	1336,937832	1259,046637	0,329890034	0,12003013	2,74839355	0,005988808	0,045689964	1,256917566
NDUFV2	431,0470664	549,6221318	680,0719586	781,9183946	837,1291295	943,0732558	723,7861266	0,623617627	0,226911867	2,748281244	0,00599086	0,045689964	1,540733805
PHC3	199,0779624	380,0019045	546,2425371	576,2137093	638,5073387	746,1409676	479,2855576	0,79755141	0,290143445	2,748817609	0,005981066	0,045689964	1,738148578
SNX27	225,0446531	494,3969415	442,456455	712,1472148	585,7659593	594,4664103	452,2884612	0,699329119	0,254421634	2,748701474	0,005983185	0,045689964	1,623749542
EPSTI1	0	11,83396934	2,731212685	14,43541652	26,93176824	13,45500106	29,46714003	1,829138824	0,66610192	2,746034457	0,006032043	0,045848423	3,553249081
ROCK1	212,9268641	202,4923643	266,2932368	318,7821147	317,5704339	388,9718487	296,6136081	0,587431932	0,214003385	2,744965615	0,006051724	0,045966914	1,502569718
C9orf72	12,11778901	28,92748062	46,43061565	62,55347157	46,00843741	69,72136911	54,70555583	0,995393972	0,362872864	2,743092887	0,006086347	0,046167467	1,993624872
IL15RA	51,93338149	32,87213707	47,79622199	105,8597211	80,79530473	58,71273188	61,13751449	0,897768726	0,327531106	2,741018211	0,006124911	0,046397339	1,863182154
KCNN3	0	1,314885483	2,731212685	4,811805506	7,855099071	13,45500106	21,98589238	2,588311831	0,945308516	2,738060418	0,006180272	0,046628057	6,013945653
SLC4A7	69,24450865	320,8320578	331,8423413	559,37239	478,0388863	462,3627635	386,3807652	1,049730255	0,383374905	2,738129805	0,006178968	0,046628057	2,070142751
IRAK1BP1	133,2956792	144,6374031	120,1733582	209,3135395	177,3008076	238,5204733	123,0475126	0,649833512	0,237439265	2,736840984	0,006203227	0,046738466	1,568987124
PPIP5K2	64,0511705	109,1354951	204,8409514	273,0699624	260,3404263	189,5931967	173,9653909	0,928312614	0,339248628	2,736378389	0,006211955	0,046741529	1,903048875
CDK6	261,3980202	420,7633545	579,0170893	608,6933964	630,6522397	740,025058	655,3511723	0,646355606	0,236235797	2,736061231	0,006217945	0,046755288	1,56520932
ZDHHC7	773,8073842	640,3492301	654,1254381	818,0069359	1127,767795	914,9400718	610,6016644	0,469861776	0,171844069	2,734233298	0,006252573	0,046921443	1,384976767
GPR137B	171,3801589	153,8416015	182,9912499	249,0109349	237,8972861	228,7350179	190,9008426	0,494870909	0,181134662	2,732060788	0,006293953	0,047168956	1,409194656
PRPF8	2266,026546	2827,003788	2399,370344	3087,976183	3844,509917	3426,132541	3423,325028	0,467025784	0,170964973	2,731704479	0,006300763	0,047188514	1,38225691
AASDH	10,3866763	18,40839676	28,6777332	44,50920093	35,90902432	39,14182125	34,18916544	1,02548792	0,376323036	2,725020317	0,006429753	0,04786736	2,035647724
AP2A1	1130,416604	807,3396864	606,3292161	1257,084188	1533,988633	1261,100553	1050,847935	0,673229532	0,247139311	2,724089213	0,006447909	0,04791129	1,594638639
PCBP2	16691,38881	13741,86818	13601,43917	17637,67308	20588,21466	24577,3942	20474,30623	0,512263378	0,188051322	2,724061567	0,006448448	0,04791129	1,426286076
NAPEPLD	72,70673409	51,28053383	75,10834885	92,62725598	124,5594281	103,9704627	75,86409179	0,697608213	0,256392427	2,720861227	0,006511209	0,048313686	1,621813822
ATF6B	886,3297108	683,740451	568,0922385	920,2578029	942,6118885	1152,237363	835,4829774	0,498159223	0,183131637	2,720224819	0,006523755	0,048374824	1,412410277
CANX	13331,29903	10878,0476	10815,60223	15450,70748	18990,26308	13238,49786	13483,55351	0,445132942	0,163717664	2,718906025	0,006549821	0,048504083	1,361439565
SSR1	1116,567702	1438,484718	2098,936949	2480,485738	2205,038525	2216,405628	1917,105127	0,567554494	0,209162731	2,713458996	0,006658481	0,049179075	1,482009293
CHD1	412,0048265	813,9141138	849,4071451	1169,268738	996,4754249	1705,115588	1027,5107	0,89783305	0,331019171	2,712329462	0,006681215	0,049314566	1,863265227
PLAG1	0	0	0	3,608854129	2,24431402	4,892727657	18,2130394	3,728330287	1,375800655	2,709934955	0,00672964	0,049519389	13,25376451
SPNS2	32,89114161	49,96564834	23,21530783	68,56822845	77,42883369	75,83727868	27,40003969	1,05950794	0,391006905	2,709691127	0,006734589	0,049519389	2,084220535
GLB1	164,4557081	351,0744239	376,9073506	454,7156203	415,1980937	675,1964166	504,0714171	0,787113066	0,290764701	2,707044778	0,00678851	0,04967931	1,725617923
SLC37A3	373,9203467	361,5935077	337,3047666	483,5864533	479,1610433	490,4959476	292,800621	0,438877963	0,162143162	2,706731243	0,006794924	0,04967931	1,355549656
IGF1R	109,0601011	148,5820595	127,0013899	175,630901	175,0564936	248,3059286	292,0688932	0,632599699	0,233851044	2,70513951	0,006827571	0,049870566	1,55035618
DUT	386,0381357	636,4045736	684,1687777	745,8298534	811,3195183	1050,713264	879,2252611	0,608801386	0,225181572	2,703602164	0,006859236	0,049945017	1,524991695

PUS7	31,16002889	105,1908386	110,6141138	166,0072899	138,0253122	205,4945616	104,9429081	1,031874895	0,381671871	2,703565483	0,006859993	0,049945017	2,044679747
