# Supplementary material for: Analysis of the Role of FRMD5 in the Biology of Papillary Thyroid Carcinoma
Source: Int J Mol Sci. 2021 Jun 23;22(13):6726. doi: 10.3390/ijms22136726 (PMC8268710; doi:10.3390/ijms22136726)
Supplement: Supplementary file 1 [file ijms-22-06726-s001.zip › Supplementary Table S2.pdf]

**Supplementary Table S2. Genes downregulated in TPC1 siFRMD5 (in comparison with TPC1 siNEG).**

| gene | TPC1 siNEG 1 | TPC1 siNEG 2 | TPC1 siNEG 3 | TPC1 siFRMD5 1 | TPC1 siFRMD5 2 | TPC1 siFRMD5 3 | baseMean | log2FoldChange | lfcSE | stat | pvalue | padj | Fold-change |
|---|---|---|---|---|---|---|---|---|---|---|---|---|---|
| HIST1H2AL | 3557,436632 | 4176,076293 | 3654,362573 | 1557,822032 | 1376,886651 | 1246,42237 | 2113,953145 | -1,445752951 | 0,142481854 | -10,14692687 | 3,41962E-24 | 1,9208E-20 | 2,724049559 |
| FZD8 | 351,4158814 | 385,2614464 | 368,7137125 | 113,0774294 | 88,6504038 | 122,3181914 | 225,5790616 | -1,774506656 | 0,177815246 | -9,979496672 | 1,87415E-23 | 7,01807E-20 | 3,421209986 |
| NOX5 | 2001,1663 | 2362,849212 | 3151,819439 | 210,5164909 | 332,158475 | 127,2109191 | 685,9321651 | -3,48709831 | 0,378239427 | -9,219288269 | 2,99079E-20 | 6,7197E-17 | 11,21298366 |
| HIST1H4F | 3751,321256 | 3565,969429 | 3179,131566 | 1587,895817 | 1328,6339 | 1467,818297 | 2021,420653 | -1,259385452 | 0,140649208 | -8,954088479 | 3,42554E-19 | 5,4975E-16 | 2,393937442 |
| SPSB1 | 2030,595216 | 1458,208 | 1226,314496 | 508,8484322 | 402,8543666 | 425,6673061 | 1134,655187 | -1,816981694 | 0,204279057 | -8,894605844 | 5,8627E-19 | 7,00219E-16 | 3,523432789 |
| PSMC3IP | 219,851315 | 136,7480902 | 139,291847 | 34,88558992 | 46,00843741 | 37,91863934 | 149,7956718 | -2,050265635 | 0,275270644 | -7,448181198 | 9,46358E-14 | 5,59547E-11 | 4,141822235 |
| EGR1 | 79,63118495 | 136,7480902 | 157,0447294 | 14,43541652 | 32,54255329 | 14,67818297 | 74,44058018 | -2,595109656 | 0,360697152 | -7,194705145 | 6,25959E-13 | 3,0574E-10 | 6,042349591 |
| SOX9 | 787,6562859 | 616,6812914 | 479,3278263 | 191,2692688 | 262,5847404 | 204,2713797 | 577,8615116 | -1,513662961 | 0,211823184 | -7,145879565 | 8,94214E-13 | 4,01824E-10 | 2,855340823 |
| TRIM16 | 472,5937716 | 493,082056 | 372,8105315 | 125,1069431 | 139,1474692 | 168,7991041 | 199,5791517 | -1,62802061 | 0,231343556 | -7,037242088 | 1,96082E-12 | 8,15846E-10 | 3,090886355 |
| CLEC11A | 1604,741488 | 1530,526702 | 1518,554253 | 656,8114515 | 631,7743967 | 681,3123262 | 683,3834245 | -1,240232208 | 0,177600417 | -6,983273055 | 2,88381E-12 | 1,08325E-09 | 2,362365525 |
| VEGFA | 1416,050202 | 1284,643117 | 1167,593423 | 436,6713496 | 437,6412339 | 590,7968645 | 1153,31473 | -1,40063356 | 0,200462254 | -6,987018928 | 2,80788E-12 | 1,08325E-09 | 2,640175001 |
| TMEM158 | 770,3451588 | 886,2328153 | 815,2669866 | 346,4499964 | 313,0818058 | 423,2209423 | 454,0296473 | -1,19251435 | 0,172425576 | -6,916110577 | 4,64212E-12 | 1,68224E-09 | 2,285507178 |
| H2AFX | 4918,091227 | 4076,144996 | 4577,512461 | 2369,814211 | 2090,57851 | 2026,812432 | 2774,769716 | -1,064727165 | 0,154624038 | -6,885909698 | 5,74194E-12 | 2,01578E-09 | 2,091774257 |
| BCL2A1 | 257,9357947 | 289,2748062 | 315,4550652 | 126,3098945 | 118,9486431 | 145,5586478 | 220,8987396 | -1,147062637 | 0,170666567 | -6,721074075 | 1,8039E-11 | 5,81899E-09 | 2,214625318 |
| PLAUR | 8778,472585 | 5063,623994 | 4296,197554 | 2267,563344 | 1973,874181 | 1898,378331 | 3482,253077 | -1,56243509 | 0,236158847 | -6,61603454 | 3,68962E-11 | 1,12025E-08 | 2,953519405 |
| FRMD5 | 48,47115606 | 55,22519027 | 55,98986005 | 6,014756882 | 13,46588412 | 8,562273399 | 94,86368746 | -2,502237352 | 0,3792839 | -6,597267506 | 4,18805E-11 | 1,20075E-08 | 5,665633784 |
| NOM1 | 550,4938438 | 614,0515204 | 546,2425371 | 303,1437468 | 242,3859142 | 223,8422903 | 288,8872845 | -1,153723681 | 0,174960246 | -6,594204715 | 4,27541E-11 | 1,20075E-08 | 2,224874071 |
| SLFN5 | 718,4117773 | 583,8091543 | 696,4592348 | 309,1585037 | 322,0590619 | 338,8213902 | 709,4246858 | -1,041604096 | 0,159149026 | -6,544834882 | 5,95612E-11 | 1,63198E-08 | 2,058515192 |
| COQ9 | 443,1648554 | 449,6908351 | 446,553274 | 211,7194422 | 208,7212039 | 223,8422903 | 317,5719935 | -1,056360128 | 0,162662313 | -6,494190977 | 8,34808E-11 | 2,23291E-08 | 2,079677939 |
| CHST1 | 221,5824277 | 353,7041948 | 450,6500931 | 104,6567697 | 120,0708001 | 91,73864356 | 151,2374111 | -1,699467007 | 0,266002282 | -6,388918906 | 1,67063E-10 | 4,26541E-08 | 3,247809485 |
| ZDHHC3 | 1024,818728 | 1103,18892 | 865,7944212 | 362,0883643 | 369,1896563 | 326,5895711 | 675,267729 | -1,500591273 | 0,236848134 | -6,335668543 | 2,36314E-10 | 5,77121E-08 | 2,829586563 |
| SRPR | 3552,243294 | 3149,150731 | 2238,228796 | 911,8371433 | 978,5209128 | 544,3159518 | 2247,912981 | -1,876100111 | 0,297901124 | -6,297727523 | 3,02041E-10 | 7,21942E-08 | 3,670814243 |
| WDR34 | 1403,932413 | 1214,954186 | 1190,808731 | 706,1324579 | 666,561264 | 551,6550433 | 894,3309677 | -0,984214898 | 0,159785133 | -6,159614982 | 7,2922E-10 | 1,60629E-07 | 1,97823648 |
| ANKRD39 | 147,1445809 | 164,3606853 | 144,7542723 | 51,72690918 | 50,49706545 | 58,71273188 | 98,21056911 | -1,505924865 | 0,244868295 | -6,149938133 | 7,75132E-10 | 1,65064E-07 | 2,840066803 |
| CDKN2D | 254,4735693 | 131,4885483 | 139,291847 | 51,72690918 | 51,61922246 | 59,93591379 | 129,7191758 | -1,676296928 | 0,274167059 | -6,114144181 | 9,70765E-10 | 1,93162E-07 | 3,196065395 |
| GFPT2 | 408,5426011 | 411,5591561 | 338,670373 | 123,9039918 | 145,8804113 | 103,9704627 | 631,2406793 | -1,630753514 | 0,266583651 | -6,117230034 | 9,52159E-10 | 1,93162E-07 | 3,096746984 |
| PSMD4 | 206,0024132 | 176,1946547 | 248,5403544 | 89,01840185 | 75,18451967 | 63,60545953 | 148,7846889 | -1,468808086 | 0,24029106 | -6,112620608 | 9,80082E-10 | 1,93162E-07 | 2,767931204 |
| TWF1 | 1360,654595 | 1232,047697 | 1824,450074 | 512,4572863 | 549,8569349 | 628,7155039 | 1345,875397 | -1,385484471 | 0,227123093 | -6,10014794 | 1,0597E-09 | 2,05254E-07 | 2,612596762 |
| DEAF1 | 877,6741472 | 769,2080074 | 686,8999904 | 407,8005166 | 446,61849 | 384,079121 | 490,6771956 | -0,911657613 | 0,149539244 | -6,096443892 | 1,08454E-09 | 2,06504E-07 | 1,881205706 |
| PCDHGC3 | 242,3557803 | 305,053432 | 322,2830969 | 110,6715266 | 100,9941309 | 143,112284 | 562,9640502 | -1,298721686 | 0,215059647 | -6,038890624 | 1,55177E-09 | 2,85781E-07 | 2,460108058 |
| GTF2A2 | 1431,630216 | 1430,595405 | 1596,393815 | 914,243046 | 710,3253874 | 727,7932389 | 1464,637914 | -0,922927925 | 0,153509166 | -6,012200765 | 1,83021E-09 | 3,2126E-07 | 1,895959209 |
| EID1 | 4189,292774 | 3576,488513 | 3741,761379 | 2371,017163 | 2174,740286 | 2213,959265 | 3716,013985 | -0,767193335 | 0,127894816 | -5,998627311 | 1,98992E-09 | 3,4392E-07 | 1,701955522 |
| SLCO4A1 | 671,6717339 | 599,5877801 | 721,0401489 | 350,0588505 | 269,3176824 | 292,3404775 | 531,045175 | -1,128014054 | 0,189799051 | -5,943201752 | 2,79508E-09 | 4,48571E-07 | 2,185576768 |
| C6orf120 | 1042,129855 | 873,0839605 | 816,6325929 | 295,9260386 | 359,0902432 | 461,1395816 | 611,9877946 | -1,290756164 | 0,217803225 | -5,926249103 | 3,09932E-09 | 4,90392E-07 | 2,446562543 |
| HIST1H2AI | 7095,831024 | 6553,389246 | 6056,46413 | 3744,787635 | 3268,84337 | 3663,429833 | 4002,518274 | -0,884024622 | 0,150949569 | -5,856423615 | 4,72941E-09 | 7,08402E-07 | 1,845516481 |
| PPP1R15A | 1042,129855 | 644,2938865 | 587,2107273 | 311,5644065 | 277,1727815 | 308,2418424 | 444,763925 | -1,339647906 | 0,229642608 | -5,833620847 | 5,42373E-09 | 7,90971E-07 | 2,530895439 |
| MYH16 | 765,1518206 | 624,5706043 | 577,6514829 | 275,4758652 | 331,036318 | 229,9581999 | 302,2939375 | -1,231447631 | 0,212045974 | -5,807455849 | 6,34293E-09 | 8,90706E-07 | 2,348024776 |
| DHRS3 | 86,55563582 | 126,2290063 | 114,7109328 | 21,65312477 | 46,00843741 | 44,03454891 | 508,8716925 | -1,556904686 | 0,268272174 | -5,803452001 | 6,49634E-09 | 9,00986E-07 | 2,942219102 |
| DOT1L | 1850,559494 | 1037,444646 | 1076,097798 | 581,0255148 | 552,101249 | 457,4700359 | 714,2013995 | -1,315732672 | 0,228134158 | -5,76736375 | 8,05211E-09 | 1,10314E-06 | 2,489287168 |
| G0S2 | 39893,49255 | 21912,56657 | 21258,39394 | 7994,814847 | 6479,334576 | 10574,40765 | 12311,20079 | -1,729478517 | 0,300251424 | -5,760100966 | 8,40636E-09 | 1,1378E-06 | 3,316079322 |
| HPCAL1 | 2910,000476 | 2400,980891 | 2231,400764 | 1169,268738 | 1235,494868 | 1494,728299 | 1437,653542 | -0,951284124 | 0,165976557 | -5,731436665 | 9,95835E-09 | 1,33181E-06 | 1,933592957 |
| PHLDA2 | 4265,461733 | 2009,145018 | 1966,473133 | 768,6859295 | 654,2175369 | 976,0991675 | 1230,573135 | -1,780017356 | 0,310910588 | -5,725174455 | 1,03328E-08 | 1,34975E-06 | 3,43430306 |
| STC1 | 2430,482254 | 1802,707997 | 1961,010708 | 950,3315873 | 1058,19406 | 1171,808274 | 2970,520927 | -0,960970092 | 0,168170742 | -5,71425254 | 1,10187E-08 | 1,40664E-06 | 1,946618394 |
| VGLL4 | 1327,763453 | 1033,499989 | 1003,720662 | 559,37239 | 493,7490844 | 647,0632326 | 654,7340257 | -0,984003469 | 0,174434965 | -5,641090764 | 1,68976E-08 | 2,04116E-06 | 1,977946589 |
| ANKH | 225,0446531 | 219,5858756 | 211,6689831 | 111,874478 | 113,337858 | 101,5240989 | 288,8682868 | -1,00447789 | 0,18453156 | -5,443393491 | 5,2275E-08 | 5,75742E-06 | 2,006217318 |
| AP1G1 | 1436,823555 | 1451,633573 | 1446,177117 | 725,37968 | 882,0154099 | 946,7428015 | 1019,221985 | -0,763101547 | 0,140420145 | -5,434416451 | 5,4976E-08 | 5,99613E-06 | 1,697135256 |
| RNF126 | 908,8341761 | 673,2213671 | 656,8566508 | 359,6824615 | 322,0590619 | 238,5204733 | 581,5462766 | -1,28097944 | 0,23662566 | -5,413527162 | 6,17952E-08 | 6,6115E-06 | 2,430038953 |
| CDC42EP3 | 1978,661835 | 1313,570597 | 1324,638152 | 774,7006864 | 604,8426284 | 757,1496048 | 933,1514093 | -1,110681717 | 0,206740333 | -5,37235141 | 7,77165E-08 | 8,08395E-06 | 2,15947665 |

| | | | | | | | | | | | | | |
|---|---|---|---|---|---|---|---|---|---|---|---|---|---|
| SERTAD2 | 216,3890895 | 340,55534 | 417,8755409 | 158,7895817 | 124,5594281 | 101,5240989 | 177,165294 | -1,344796285 | 0,251950068 | -5,337550798 | 9,42105E-08 | 9,47597E-06 | 2,539943288 |
| SIRPB1 | 183,4979479 | 256,4026691 | 221,2282275 | 74,58298534 | 126,8037421 | 101,5240989 | 379,3355776 | -1,128183353 | 0,211735389 | -5,328270164 | 9,91526E-08 | 9,68591E-06 | 2,18583326 |
| RGL2 | 353,1469941 | 269,551524 | 376,9073506 | 157,5866303 | 154,8576674 | 133,3268286 | 270,5569869 | -1,163170059 | 0,218438494 | -5,324931685 | 1,00991E-07 | 9,78045E-06 | 2,239489744 |
| CCND1 | 14871,98935 | 10555,90066 | 9114,056731 | 6569,317466 | 5552,432886 | 4974,680845 | 6805,532768 | -1,014476298 | 0,190985501 | -5,311797452 | 1,08549E-07 | 1,04226E-05 | 2,020169433 |
| CD68 | 1608,203713 | 1644,921739 | 1372,434374 | 938,3020736 | 921,2909053 | 1034,811899 | 1373,383838 | -0,676248964 | 0,127450618 | -5,305968518 | 1,12076E-07 | 1,05804E-05 | 1,597979571 |
| HMGA1 | 23304,23939 | 14370,38344 | 14091,69185 | 7701,294712 | 7086,421519 | 5561,808164 | 12300,14687 | -1,346925681 | 0,254261963 | -5,297393537 | 1,17467E-07 | 1,09124E-05 | 2,543694974 |
| SDC3 | 1334,687904 | 1080,835867 | 1014,645513 | 476,368745 | 503,8484975 | 294,7868413 | 907,6978585 | -1,426806442 | 0,269546349 | -5,293362146 | 1,20088E-07 | 1,10579E-05 | 2,688509263 |
| ITPKA | 110,7912138 | 136,7480902 | 140,6574533 | 43,30624955 | 26,93176824 | 47,70409465 | 68,96845307 | -1,725950203 | 0,327200647 | -5,274898501 | 1,3283E-07 | 1,18429E-05 | 3,307979291 |
| PFKP | 4358,94182 | 3441,055308 | 4034,001136 | 2319,290254 | 2285,83383 | 2353,402003 | 2913,824343 | -0,765744012 | 0,145468878 | -5,263971383 | 1,40976E-07 | 1,23729E-05 | 1,700246607 |
| RGCC | 117,7156647 | 61,79961769 | 79,20516787 | 21,65312477 | 29,17608226 | 23,24045637 | 76,40262205 | -1,789238296 | 0,339877634 | -5,264360215 | 1,40678E-07 | 1,23729E-05 | 3,456323599 |
| LRFN3 | 173,1112716 | 198,5477079 | 185,7224626 | 97,43906149 | 83,03961875 | 84,39955207 | 134,9693176 | -1,076611033 | 0,204844302 | -5,255752884 | 1,4742E-07 | 1,28381E-05 | 2,109075927 |
| ATAD3B | 275,2469219 | 270,8664094 | 238,98111 | 139,5423597 | 100,9941309 | 118,6486457 | 311,8250046 | -1,128576301 | 0,21598773 | -5,22518711 | 1,73979E-07 | 1,49197E-05 | 2,186428698 |
| TMEM209 | 294,2891618 | 251,1431272 | 277,2180876 | 126,3098945 | 133,5366842 | 106,4168265 | 241,5234523 | -1,164744181 | 0,223149085 | -5,21957857 | 1,79331E-07 | 1,51474E-05 | 2,24193458 |
| ZMIZ1 | 683,789523 | 688,9999929 | 538,048899 | 286,3024276 | 352,3573012 | 353,4995732 | 618,5176117 | -0,944590096 | 0,18094629 | -5,220278866 | 1,78654E-07 | 1,51474E-05 | 1,924641972 |
| DVL1 | 2037,519667 | 1354,332047 | 1088,388255 | 678,4645763 | 720,4248005 | 740,025058 | 1328,732131 | -1,065249874 | 0,207411703 | -5,135919807 | 2,80767E-07 | 2,26917E-05 | 2,092532274 |
| IDH2 | 2091,184161 | 1671,219449 | 1529,479104 | 997,246691 | 1024,52935 | 828,0941559 | 1445,569886 | -0,891795277 | 0,174182292 | -5,119896315 | 3,05704E-07 | 2,42455E-05 | 1,855483635 |
| SAMD4B | 1139,072167 | 892,8072428 | 895,8377608 | 543,7340221 | 590,2545873 | 605,4750475 | 914,0607238 | -0,748990617 | 0,146303656 | -5,119425169 | 3,06468E-07 | 2,42455E-05 | 1,680616574 |
| PFDN2 | 4833,266704 | 4636,286212 | 4154,174494 | 2884,677401 | 2616,870147 | 2704,455212 | 3131,279513 | -0,731247578 | 0,143112316 | -5,109606199 | 3,22831E-07 | 2,51853E-05 | 1,660074029 |
| ZNF335 | 429,3159537 | 277,4408369 | 213,0345895 | 111,874478 | 148,1247253 | 129,6572829 | 309,1426405 | -1,232234904 | 0,241490316 | -5,102626569 | 3,34972E-07 | 2,57371E-05 | 2,349306433 |
| RHBDD2 | 810,1607513 | 760,003809 | 876,719272 | 513,6602377 | 512,8257536 | 516,1827678 | 469,0079605 | -0,665483694 | 0,130657469 | -5,093345966 | 3,51799E-07 | 2,67034E-05 | 1,586099957 |
| SIX1 | 216,3890895 | 134,1183192 | 228,0562592 | 75,78593671 | 87,52824679 | 78,2836425 | 456,285883 | -1,255209607 | 0,24686411 | -5,084617632 | 3,68367E-07 | 2,77734E-05 | 2,387018267 |
| SLC25A22 | 1019,62539 | 892,8072428 | 817,9981992 | 487,1953074 | 437,6412339 | 609,1445932 | 761,4635475 | -0,831349529 | 0,163831304 | -5,074424168 | 3,88671E-07 | 2,91089E-05 | 1,779349026 |
| SOCS3 | 122,9090029 | 93,35686927 | 117,4421455 | 43,30624955 | 43,76412339 | 52,59682231 | 83,53582238 | -1,252949349 | 0,248081495 | -5,05055546 | 4,40527E-07 | 3,27741E-05 | 2,383281475 |
| TNFRSF12A | 8402,821125 | 5012,34346 | 5451,50052 | 3398,337638 | 2914,241755 | 3171,710703 | 4166,399585 | -0,991898917 | 0,197419416 | -5,024323023 | 5,05211E-07 | 3,73391E-05 | 1,988800987 |
| AGPAT2 | 659,5539449 | 679,7957946 | 729,233787 | 407,8005166 | 313,0818058 | 276,4391126 | 502,1561806 | -1,052909032 | 0,21019635 | -5,009168964 | 5,46656E-07 | 3,96202E-05 | 2,074709049 |
| TNPO2 | 598,9649999 | 784,9866332 | 733,330606 | 335,623434 | 389,3884825 | 347,3836636 | 515,1623023 | -0,982527009 | 0,196143978 | -5,009213231 | 5,4653E-07 | 3,96202E-05 | 1,975923385 |
| YWHAG | 1194,467774 | 1267,549605 | 1178,518274 | 645,9848891 | 533,0245798 | 426,890488 | 697,6977951 | -1,180765834 | 0,236428742 | -4,994172137 | 5,90887E-07 | 4,22804E-05 | 2,26697084 |
| PHC2 | 283,9024855 | 226,160303 | 199,378526 | 102,250867 | 95,38334586 | 68,49818719 | 329,2082094 | -1,410774833 | 0,282656005 | -4,991136963 | 6,00249E-07 | 4,26785E-05 | 2,658799215 |
| NR2F2 | 2953,278294 | 2088,038147 | 1666,039738 | 1123,556586 | 1142,355836 | 1187,709639 | 1181,764027 | -0,956674303 | 0,192280405 | -4,975412361 | 6,51089E-07 | 4,54306E-05 | 1,940830736 |
| NTMT1 | 221,5824277 | 235,3645014 | 200,7441324 | 128,7157973 | 116,704329 | 119,8718276 | 190,2211279 | -0,848924992 | 0,170594475 | -4,976274841 | 6,48196E-07 | 4,54306E-05 | 1,801158312 |
| C19orf10 | 1194,467774 | 890,1774718 | 938,1715574 | 623,128813 | 558,834191 | 552,8782252 | 889,51243 | -0,799271616 | 0,161206188 | -4,958070331 | 7,11968E-07 | 4,93719E-05 | 1,740222305 |
| PREX1 | 581,6538727 | 435,2270948 | 492,9838897 | 251,4168377 | 242,3859142 | 160,2368308 | 587,2591801 | -1,204798893 | 0,244292468 | -4,931788954 | 8,14799E-07 | 5,48111E-05 | 2,305051355 |
| RHOF | 2080,797485 | 2540,358753 | 2303,7779 | 1497,674464 | 1169,287604 | 1456,80966 | 1873,188028 | -0,748625765 | 0,152383619 | -4,912770597 | 8,97983E-07 | 5,93408E-05 | 1,680191606 |
| HIST2H2BE | 16731,2044 | 15464,36816 | 16104,5956 | 10881,89815 | 9639,328717 | 7928,665167 | 9813,458182 | -0,76356372 | 0,155892039 | -4,898028948 | 9,68028E-07 | 6,35955E-05 | 1,697679027 |
| CSF3 | 41,54670519 | 19,72328224 | 16,38727611 | 1,202951376 | 1,12215701 | 1,223181914 | 675,7076276 | -4,418397058 | 0,906561989 | -4,873794743 | 1,09475E-06 | 7,02928E-05 | 21,38306948 |
| GPRIN3 | 74,4378468 | 93,35686927 | 86,03319959 | 32,47968716 | 26,93176824 | 23,24045637 | 35,91687053 | -1,622559414 | 0,333084339 | -4,871317033 | 1,10857E-06 | 7,07594E-05 | 3,079208192 |
| IL1B | 17,31112716 | 46,02099189 | 72,37713616 | 13,23246514 | 13,46588412 | 11,00863723 | 1728,341485 | -1,874666552 | 0,38550825 | -4,862844181 | 1,15711E-06 | 7,30279E-05 | 3,667168487 |
| CCDC8 | 751,3029189 | 633,7748027 | 581,748302 | 319,9850661 | 309,7153348 | 242,190019 | 291,4334359 | -1,172348102 | 0,24236167 | -4,837184444 | 1,31691E-06 | 8,20327E-05 | 2,253782197 |
| RRM2 | 3000,018337 | 3116,278594 | 4039,463562 | 1957,201889 | 1839,21534 | 1893,485603 | 2712,542468 | -0,836098712 | 0,172883433 | -4,836199164 | 1,32345E-06 | 8,20327E-05 | 1,785216087 |
| TMCC3 | 2077,33526 | 2089,353032 | 1381,993619 | 904,619435 | 749,6008827 | 718,0077836 | 988,4529977 | -1,225717321 | 0,254360538 | -4,818818716 | 1,44411E-06 | 8,79193E-05 | 2,338717039 |
| HIVEP3 | 273,5158092 | 248,5133562 | 241,7123226 | 139,5423597 | 133,5366842 | 121,0950095 | 192,2619128 | -0,95191454 | 0,19772372 | -4,814366941 | 1,47667E-06 | 8,87109E-05 | 1,934438067 |
| ADIPOR1 | 1448,941344 | 1366,166017 | 1084,291436 | 874,5456506 | 769,7997089 | 747,3641495 | 1329,103691 | -0,704324484 | 0,146510979 | -4,807315383 | 1,52971E-06 | 9,14081E-05 | 1,629381558 |
| GDI1 | 2506,651213 | 2057,79578 | 1574,544113 | 1146,412662 | 1219,78467 | 1020,133716 | 1643,267986 | -0,857283586 | 0,178811083 | -4,794353758 | 1,632E-06 | 9,5989E-05 | 1,811624037 |
| ZFP36L2 | 901,9097252 | 465,4694609 | 512,1023785 | 312,7673579 | 272,6841534 | 250,7522924 | 448,1514377 | -1,165198538 | 0,243023406 | -4,794593883 | 1,63005E-06 | 9,5989E-05 | 2,242640758 |
| ATF5 | 2077,33526 | 1337,238536 | 1189,443124 | 738,6121451 | 875,2824679 | 692,3209634 | 1336,84642 | -0,995723426 | 0,207732625 | -4,793293432 | 1,64065E-06 | 9,59953E-05 | 1,994080188 |
| HIST1H4H | 25267,32121 | 18013,93111 | 13981,07774 | 10424,77663 | 9818,873838 | 8541,479306 | 9887,055531 | -0,992157362 | 0,207312794 | -4,785799003 | 1,70308E-06 | 9,81151E-05 | 1,989157292 |
| LENG1 | 574,7294218 | 391,8358738 | 329,1111286 | 239,3873239 | 205,3547328 | 178,5845595 | 342,8935413 | -1,051412207 | 0,220232039 | -4,774111036 | 1,80503E-06 | 0,000103458 | 2,072557613 |
| HIST1H3A | 5132,749204 | 5902,520932 | 4501,038505 | 3346,610729 | 3201,51395 | 3039,607057 | 3223,339699 | -0,696433874 | 0,146217395 | -4,763002882 | 1,90733E-06 | 0,000108217 | 1,620494219 |
| CDT1 | 1294,872312 | 936,1984637 | 838,4822944 | 543,7340221 | 449,984961 | 598,135956 | 781,0932209 | -0,946103995 | 0,199449874 | -4,743567767 | 2,09987E-06 | 0,000117369 | 1,926662664 |
| ANTXR2 | 1686,103786 | 1254,400751 | 969,5805033 | 651,999646 | 666,561264 | 483,1568561 | 1578,314936 | -1,116566391 | 0,23653718 | -4,720468848 | 2,35302E-06 | 0,000129705 | 2,168303026 |

| | | | | | | | | | | | | | |
|---|---|---|---|---|---|---|---|---|---|---|---|---|---|
| BEND3 | 266,5913583 | 144,6374031 | 154,3135167 | 81,80069359 | 48,25275143 | 85,62273399 | 103,8963689 | -1,386709852 | 0,294315982 | -4,711636256 | 2,45736E-06 | 0,000133639 | 2,614816764 |
| HIST1H1E | 10611,72095 | 8619,074339 | 8810,892123 | 5720,033795 | 4494,238825 | 3227,977071 | 4981,266639 | -1,060705943 | 0,225144943 | -4,711213712 | 2,46246E-06 | 0,000133639 | 2,085951975 |
| B3GALNT1 | 695,907312 | 452,3206061 | 345,4984047 | 221,3430533 | 230,0421871 | 251,9754743 | 355,3153513 | -1,082867251 | 0,230648251 | -4,694886032 | 2,66755E-06 | 0,000142701 | 2,118241751 |
| PKMYT1 | 455,2826444 | 385,2614464 | 348,2296174 | 216,5312477 | 186,2780637 | 238,5204733 | 229,1129621 | -0,888730857 | 0,189646282 | -4,686255111 | 2,78249E-06 | 0,000147446 | 1,851546596 |
| SUMO3 | 4104,46825 | 2717,868293 | 2145,367564 | 1514,515783 | 1565,409029 | 1351,616015 | 1778,017533 | -1,01619568 | 0,216822935 | -4,686753645 | 2,77573E-06 | 0,000147446 | 2,022578475 |
| TFDP1 | 3858,650245 | 2386,517151 | 2437,607322 | 1589,098768 | 1566,531186 | 1431,12284 | 1643,90837 | -0,919869922 | 0,197380173 | -4,66039678 | 3,156E-06 | 0,000164905 | 1,891944702 |
| JARID2 | 834,3963293 | 687,6851075 | 650,0286191 | 459,5274258 | 301,8602357 | 280,1086583 | 528,569923 | -1,059632959 | 0,227801158 | -4,651569684 | 3,29418E-06 | 0,000169888 | 2,084401154 |
| DDX39A | 1045,592081 | 1042,704188 | 1024,204757 | 577,4166607 | 582,3994882 | 675,1964166 | 746,6965631 | -0,762460543 | 0,16424623 | -4,642179861 | 3,44753E-06 | 0,000176043 | 1,696381369 |
| PIM3 | 711,4873264 | 569,345414 | 514,8335912 | 369,3060725 | 280,5392525 | 269,1000211 | 483,4265405 | -0,964810958 | 0,207927633 | -4,64012861 | 3,48192E-06 | 0,000176995 | 1,951807752 |
| C14orf80 | 382,5759103 | 281,3854933 | 304,5302144 | 158,7895817 | 162,7127665 | 135,7731925 | 232,8626728 | -1,079167294 | 0,23329477 | -4,625767193 | 3,73214E-06 | 0,000187174 | 2,112816237 |
| GRK6 | 375,6514594 | 265,6068675 | 275,8524812 | 190,0663175 | 154,8576674 | 136,9963744 | 268,3745629 | -0,923181422 | 0,199703986 | -4,622749099 | 3,78688E-06 | 0,000189075 | 1,896292378 |
| PGP | 410,2737138 | 293,2194626 | 427,4347853 | 166,0072899 | 127,9258991 | 200,6018339 | 220,7211026 | -1,19349215 | 0,258289635 | -4,620751235 | 3,82353E-06 | 0,000189763 | 2,287056727 |
| ELAVL1 | 1040,398743 | 1054,538157 | 1095,216287 | 728,9885341 | 634,0187107 | 706,9991464 | 811,3929381 | -0,624615169 | 0,135402501 | -4,613025345 | 3,9685E-06 | 0,00019539 | 1,541799504 |
| NCAPH2 | 436,2404045 | 330,0362562 | 341,4015857 | 187,6604147 | 173,9343366 | 215,2800169 | 257,122395 | -0,938811841 | 0,203546562 | -4,61227068 | 3,98294E-06 | 0,00019539 | 1,916948849 |
| KANSL3 | 131,5645664 | 145,9522886 | 181,6256436 | 70,97413121 | 72,94020565 | 79,50682442 | 127,5107019 | -1,044005611 | 0,227212822 | -4,594835802 | 4,3309E-06 | 0,000210621 | 2,061944657 |
| ARPC5 | 3462,225433 | 3844,725151 | 3397,628581 | 2194,183311 | 2173,618129 | 2092,864255 | 2321,278693 | -0,728565979 | 0,159772951 | -4,560008269 | 5,11516E-06 | 0,000244526 | 1,656991244 |
| ACSL4 | 2018,477427 | 1443,74426 | 1640,093218 | 1021,305719 | 1051,461118 | 1075,176903 | 1369,107973 | -0,695464396 | 0,152558014 | -4,558688064 | 5,14742E-06 | 0,000245026 | 1,619405627 |
| IBA57 | 147,1445809 | 118,3396934 | 113,3453264 | 61,3505202 | 68,45157761 | 57,48954996 | 121,1569677 | -1,006923763 | 0,221005762 | -4,556097337 | 5,21128E-06 | 0,000246402 | 2,009621443 |
| KIAA1549L | 2338,73328 | 2099,872116 | 1723,395204 | 1222,198598 | 1204,074472 | 1186,486457 | 1159,064297 | -0,769726595 | 0,168957612 | -4,555737893 | 5,2202E-06 | 0,000246402 | 1,704946648 |
| ATP6V0B | 1113,105477 | 678,4809091 | 613,1572479 | 370,5090239 | 301,8602357 | 321,6968434 | 787,7687762 | -1,273019081 | 0,279608085 | -4,55286935 | 5,29191E-06 | 0,000248742 | 2,41666765 |
| MRI1 | 391,2314739 | 351,0744239 | 352,3264364 | 233,372567 | 204,2325758 | 232,4045637 | 474,2136673 | -0,706943317 | 0,155639004 | -4,542198923 | 5,56705E-06 | 0,000258431 | 1,632341957 |
| RARG | 443,1648554 | 414,1889271 | 352,3264364 | 234,5755184 | 265,9512114 | 205,4945616 | 435,5063821 | -0,773641321 | 0,171761859 | -4,504150847 | 6,66389E-06 | 0,000301863 | 1,709579272 |
| DHX35 | 290,8269363 | 276,1259514 | 267,6588432 | 179,2397551 | 134,6588412 | 161,4600127 | 406,384055 | -0,812370058 | 0,18099183 | -4,48843495 | 7,17483E-06 | 0,000321124 | 1,756093983 |
| ALDH1A3 | 57,12671964 | 98,6164112 | 84,66759324 | 31,27673579 | 42,64196638 | 22,01727445 | 87,70346877 | -1,330904482 | 0,296754502 | -4,484867032 | 7,29594E-06 | 0,000324658 | 2,515603384 |
| HIST1H2BG | 1701,6838 | 2051,221353 | 1683,79262 | 1134,383148 | 1144,60015 | 1208,503731 | 1396,019694 | -0,641124156 | 0,142967336 | -4,484410031 | 7,31159E-06 | 0,000324658 | 1,559543891 |
| HIST1H2AG | 15103,95845 | 10195,62203 | 9071,722934 | 6171,140561 | 6230,21572 | 6580,718698 | 6774,780776 | -0,856378121 | 0,191093246 | -4,481467235 | 7,41316E-06 | 0,000326963 | 1,810487381 |
| INO80E | 806,6985258 | 670,5915962 | 610,4260352 | 381,3355863 | 354,6016152 | 285,001386 | 529,7388838 | -1,03069148 | 0,230700244 | -4,467665315 | 7,90779E-06 | 0,000345666 | 2,043003223 |
| FAM100B | 2714,384739 | 1805,337768 | 1495,338945 | 933,4902681 | 1193,975059 | 889,2532516 | 2694,967649 | -0,99446979 | 0,223613986 | -4,447261139 | 8,69721E-06 | 0,000375786 | 1,992348177 |
| SH2B1 | 415,4670519 | 406,2996142 | 382,3697759 | 238,1843725 | 207,5990469 | 196,9322882 | 354,9000261 | -0,905315151 | 0,203872107 | -4,440603305 | 8,9707E-06 | 0,000386118 | 1,87295359 |
| NR1D1 | 233,7002167 | 182,7690821 | 142,0230596 | 91,4243046 | 96,50550287 | 105,1936446 | 269,1628796 | -0,922234173 | 0,208406487 | -4,425170189 | 9,63663E-06 | 0,000410068 | 1,895047713 |
| SWI5 | 1847,097268 | 1153,154568 | 1088,388255 | 730,1914855 | 671,049892 | 753,4800591 | 1195,555398 | -0,92269213 | 0,208580408 | -4,423675919 | 9,70355E-06 | 0,000411357 | 1,895649357 |
| SLC25A25 | 181,7668352 | 163,0457999 | 120,1733582 | 85,40954772 | 71,81804864 | 62,38227762 | 95,82651683 | -1,077402602 | 0,24361987 | -4,422474254 | 9,7577E-06 | 0,000412098 | 2,110233438 |
| SRXN1 | 746,1095807 | 624,5706043 | 628,1789176 | 392,1621487 | 323,1812189 | 308,2418424 | 547,268164 | -0,964647916 | 0,219366902 | -4,397417774 | 1,09546E-05 | 0,000450785 | 1,951587186 |
| HIST2H3D | 304,6758381 | 227,4751885 | 184,3568563 | 133,5276028 | 112,215701 | 118,6486457 | 166,067383 | -0,969948771 | 0,220663831 | -4,395594719 | 1,1047E-05 | 0,000452927 | 1,958771039 |
| HIST1H4D | 7291,446761 | 4896,633538 | 4516,060175 | 3039,858128 | 2961,37235 | 2516,085197 | 3217,575199 | -0,971417342 | 0,222115772 | -4,373473042 | 1,22285E-05 | 0,000488317 | 1,960765958 |
| TONSL | 358,3403323 | 310,3129739 | 356,4232554 | 194,878123 | 217,69846 | 196,9322882 | 194,3715079 | -0,74777006 | 0,170942445 | -4,374396669 | 1,21769E-05 | 0,000488317 | 1,679195331 |
| KLC2 | 1066,365433 | 665,3320543 | 655,4910445 | 457,121523 | 371,4339703 | 399,9804859 | 510,2636108 | -0,956266396 | 0,219315003 | -4,360241585 | 1,29919E-05 | 0,000513912 | 1,940282063 |
| HIST1H2AK | 824,009653 | 614,0515204 | 606,3292161 | 452,3097175 | 335,524946 | 310,6882062 | 456,7243395 | -0,894357556 | 0,205940842 | -4,342788671 | 1,40685E-05 | 0,000548883 | 1,85878197 |
| DUSP23 | 1376,234609 | 741,5954122 | 688,2655967 | 453,5126689 | 384,8998545 | 401,2036678 | 546,028386 | -1,176991283 | 0,271701945 | -4,331920713 | 1,47814E-05 | 0,000570634 | 2,261047473 |
| C16orf59 | 110,7912138 | 113,0801515 | 117,4421455 | 64,95937432 | 60,59647854 | 42,81136699 | 99,36626572 | -1,018351649 | 0,235389217 | -4,326245961 | 1,51672E-05 | 0,000577588 | 2,025603285 |
| RANGAP1 | 1544,152543 | 1272,809147 | 1373,799981 | 756,6564157 | 673,294206 | 971,2064398 | 976,7183591 | -0,803294519 | 0,185679163 | -4,326250222 | 1,51669E-05 | 0,000577588 | 1,745081624 |
| MCM7 | 1409,125751 | 1212,324415 | 1074,732192 | 785,5272488 | 768,6775519 | 820,7550644 | 1125,63519 | -0,636916646 | 0,14733033 | -4,32305177 | 1,53886E-05 | 0,000584037 | 1,555002226 |
| NAB2 | 247,5491184 | 156,4713724 | 172,0663992 | 97,43906149 | 98,74981689 | 111,3095542 | 202,6414334 | -0,896634021 | 0,207899112 | -4,312832378 | 1,61176E-05 | 0,00060557 | 1,861717304 |
| HIST1H4B | 8070,447484 | 8669,039988 | 8074,830304 | 5058,410538 | 4909,436919 | 2988,233416 | 4469,729613 | -0,937506354 | 0,217528305 | -4,309813182 | 1,63393E-05 | 0,000609818 | 1,915214997 |
| FAM86A | 221,5824277 | 149,896945 | 157,0447294 | 97,43906149 | 81,91746174 | 90,51546165 | 126,7428105 | -0,962569893 | 0,22346087 | -4,307554578 | 1,65069E-05 | 0,000614036 | 1,948778192 |
| ARMC5 | 128,102341 | 123,5992354 | 105,1516884 | 61,3505202 | 61,71863555 | 41,58818508 | 89,8841739 | -1,111020172 | 0,258028456 | -4,305804824 | 1,6638E-05 | 0,000614839 | 2,15998332 |
| FAM83H | 664,7472831 | 410,2442706 | 345,4984047 | 235,7784698 | 200,8661048 | 144,3354659 | 201,8096791 | -1,287034336 | 0,298985736 | -4,304668024 | 1,67236E-05 | 0,000615978 | 2,440259098 |
| KIAA0247 | 334,1047543 | 255,0877836 | 249,9059607 | 147,9630193 | 135,7809982 | 165,1295584 | 247,473881 | -0,899215599 | 0,209035584 | -4,301734569 | 1,69466E-05 | 0,000620125 | 1,865051669 |
| EDC4 | 221,5824277 | 243,2538143 | 215,7658021 | 141,9482624 | 131,2923702 | 127,2109191 | 180,6382889 | -0,766423653 | 0,178374614 | -4,296708113 | 1,73353E-05 | 0,000632289 | 1,701047766 |
| HIST2H2AB | 3673,421184 | 3210,950349 | 2931,956818 | 1996,899285 | 2355,407564 | 2232,306993 | 2002,900033 | -0,575471896 | 0,134154061 | -4,289634568 | 1,78967E-05 | 0,000650653 | 1,490164807 |

| | | | | | | | | | | | | | | |
|---|---|---|---|---|---|---|---|---|---|---|---|---|---|---|
| C7orf50 | 2525,693453 | 1221,528613 | 1103,409925 | 731,3944368 | 770,9218659 | 692,3209634 | 1026,900586 | -1,142846836 | 0,266528879 | -4,287891208 | 1,80377E-05 | 0,000651563 | 2,208163252 |
| ALKBH6 | 51,93338149 | 72,31870155 | 90,13001862 | 38,49444404 | 23,56529721 | 19,57091063 | 58,13761522 | -1,403930813 | 0,327574491 | -4,285836812 | 1,82053E-05 | 0,000655506 | 2,646215973 |
| MAST4 | 751,3029189 | 854,6755638 | 542,145718 | 388,5532946 | 429,7861349 | 296,0100232 | 401,521548 | -0,946111947 | 0,22087097 | -4,283550461 | 1,83934E-05 | 0,000658063 | 1,926673284 |
| GMIP | 110,7912138 | 131,4885483 | 128,3669962 | 66,1623257 | 60,59647854 | 46,48091274 | 114,7873086 | -1,099600364 | 0,256782557 | -4,282223752 | 1,85035E-05 | 0,000659899 | 2,14295323 |
| ENO2 | 2141,38643 | 1205,749988 | 1074,732192 | 724,1767286 | 694,6151892 | 488,0495837 | 919,6433806 | -1,212323074 | 0,283633852 | -4,274253809 | 1,91778E-05 | 0,000681785 | 2,317104441 |
| FBXO46 | 90,01786125 | 89,41221282 | 96,95805033 | 39,69739542 | 32,54255329 | 45,25773082 | 65,19023845 | -1,237785832 | 0,28975354 | -4,271857503 | 1,93851E-05 | 0,00068482 | 2,358363059 |
| VEGFB | 1010,969826 | 975,6450282 | 797,5141041 | 599,0697854 | 562,200662 | 499,058221 | 726,275629 | -0,744923971 | 0,175206868 | -4,251682486 | 2,12171E-05 | 0,000735655 | 1,675885949 |
| CDCA4 | 427,5848409 | 257,7175546 | 218,4970148 | 92,62725598 | 98,74981689 | 134,5500106 | 167,65471 | -1,468985722 | 0,345942951 | -4,246323617 | 2,17307E-05 | 0,000748842 | 2,768272035 |
| GTF2F1 | 1244,670043 | 1354,332047 | 1507,629402 | 973,1876635 | 750,7230397 | 837,8796112 | 1281,101922 | -0,68170177 | 0,160739712 | -4,241028946 | 2,22497E-05 | 0,000764384 | 1,604030718 |
| SRPX | 1126,954378 | 841,5267089 | 1074,732192 | 445,0920093 | 420,8088788 | 540,646406 | 991,9017454 | -1,113197969 | 0,263676804 | -4,221827457 | 2,4233E-05 | 0,000819979 | 2,163246351 |
| SPTSSA | 1258,518945 | 1426,650749 | 1326,003759 | 910,6341919 | 950,4669875 | 813,4159729 | 1273,129714 | -0,585117739 | 0,13867267 | -4,219416402 | 2,44936E-05 | 0,000821628 | 1,500161413 |
| ZMYND19 | 429,3159537 | 381,31679 | 387,8322013 | 235,7784698 | 222,187088 | 203,0481977 | 359,0737423 | -0,857052316 | 0,203069361 | -4,220490543 | 2,43771E-05 | 0,000821628 | 1,811333649 |
| GDF15 | 60,58894507 | 64,42938865 | 73,7427425 | 18,04427065 | 34,78686731 | 26,91000211 | 59,98244902 | -1,3155641 | 0,312068559 | -4,215625265 | 2,49087E-05 | 0,000832811 | 2,488996324 |
| OBFC1 | 493,3671242 | 393,1507593 | 416,5099345 | 253,8227404 | 245,7523852 | 226,2886541 | 302,8361553 | -0,841801199 | 0,200383962 | -4,200940982 | 2,65808E-05 | 0,000878261 | 1,792286407 |
| RASSF8 | 1293,141199 | 1272,809147 | 1087,022649 | 625,5347157 | 628,4079256 | 489,2727657 | 1501,056431 | -1,066974339 | 0,254206486 | -4,197274246 | 2,70147E-05 | 0,000888497 | 2,09503499 |
| SHANK1 | 2283,337673 | 1520,007618 | 1160,765391 | 787,9331515 | 722,6691145 | 924,7255271 | 639,2781341 | -1,026609724 | 0,244606277 | -4,196988464 | 2,70488E-05 | 0,000888497 | 2,03723121 |
| TM4SF19 | 4538,977542 | 3367,421721 | 3119,044887 | 1419,482624 | 1519,400592 | 2189,495626 | 1573,357614 | -1,104107752 | 0,263405545 | -4,191664802 | 2,76915E-05 | 0,00090432 | 2,149658894 |
| ADCK3 | 297,7513872 | 182,7690821 | 177,5288245 | 123,9039918 | 108,84923 | 103,9704627 | 271,1064752 | -0,95831827 | 0,229106915 | -4,182843062 | 2,87886E-05 | 0,000932021 | 1,943043595 |
| MUS81 | 1163,307745 | 642,979001 | 589,94194 | 401,7857597 | 405,0986806 | 325,3663892 | 523,4483839 | -1,079101285 | 0,258168248 | -4,179837346 | 2,91718E-05 | 0,000941712 | 2,112719571 |
| NUBP2 | 382,5759103 | 214,3263337 | 199,378526 | 96,23611011 | 109,971387 | 129,6572829 | 213,8424399 | -1,2406451 | 0,297284227 | -4,173262449 | 3,00269E-05 | 0,000966538 | 2,36304172 |
| KLF2 | 90,01786125 | 149,896945 | 172,0663992 | 76,98888809 | 62,84079256 | 56,26636805 | 114,3226874 | -1,080975948 | 0,259098705 | -4,172062328 | 3,01855E-05 | 0,000967232 | 2,115466659 |
| MAP1B | 3148,894031 | 3890,746143 | 3408,553431 | 2404,699801 | 2280,223044 | 2155,246533 | 3147,661495 | -0,611500194 | 0,146591867 | -4,171446937 | 3,02672E-05 | 0,000967232 | 1,527847124 |
| MRPL4 | 574,7294218 | 628,5152607 | 454,7469121 | 287,505379 | 249,1188562 | 384,079121 | 466,1834355 | -0,849454682 | 0,203713085 | -4,169858229 | 3,04789E-05 | 0,000967232 | 1,801819734 |
| TNIP1 | 5968,876646 | 5226,669794 | 5196,132134 | 3446,455693 | 3409,112997 | 4024,268497 | 6113,199199 | -0,591153699 | 0,142361985 | -4,152468795 | 3,28908E-05 | 0,001029233 | 1,50645095 |
| SASH1 | 517,6027022 | 540,4179334 | 644,5661937 | 313,9703092 | 364,7010283 | 346,1604817 | 499,8817612 | -0,733141722 | 0,176945344 | -4,14332305 | 3,42309E-05 | 0,001065236 | 1,662255005 |
| H2AFV | 16751,97776 | 12028,5724 | 10466,00701 | 7950,305647 | 8085,141258 | 6561,147787 | 8835,439861 | -0,796293505 | 0,192518606 | -4,13618986 | 3,5312E-05 | 0,001092824 | 1,736633724 |
| UBE2Q2 | 789,3873987 | 573,2900705 | 628,1789176 | 368,1031212 | 408,4651517 | 410,9891231 | 550,3460293 | -0,742903849 | 0,179847986 | -4,130732104 | 3,6161E-05 | 0,001116023 | 1,673540945 |
| HIST1H3J | 437,9715172 | 323,4618287 | 222,5938339 | 172,0220468 | 177,3008076 | 148,0050116 | 2728,037715 | -0,979396711 | 0,237381894 | -4,125827344 | 3,69404E-05 | 0,001130841 | 1,971640759 |
| AMPD3 | 344,4914306 | 373,4274771 | 312,7238525 | 218,9371505 | 205,3547328 | 155,3441031 | 430,5807865 | -0,829755363 | 0,201451642 | -4,118881116 | 3,80716E-05 | 0,00115907 | 1,777383946 |
| TANC1 | 808,4296385 | 585,1240398 | 610,4260352 | 446,2949606 | 403,9765236 | 374,2936657 | 441,4310408 | -0,707712133 | 0,171861827 | -4,117913483 | 3,82318E-05 | 0,001160801 | 1,633212068 |
| BGLAP | 121,1778901 | 117,024808 | 114,7109328 | 58,94461744 | 56,1078505 | 57,48954996 | 54,41651173 | -1,031622135 | 0,250697938 | -4,115000483 | 3,87179E-05 | 0,001172389 | 2,044321551 |
| BMP2 | 27,69780346 | 23,66793869 | 53,25864736 | 14,43541652 | 6,73294206 | 11,00863723 | 223,9203576 | -1,718020483 | 0,417804893 | -4,112016191 | 3,92219E-05 | 0,001181283 | 3,289846983 |
| HMOX1 | 9701,155662 | 7146,402599 | 5664,535109 | 4446,108287 | 4389,878223 | 3727,035292 | 3240,866038 | -0,841261683 | 0,204998763 | -4,103740296 | 4,06524E-05 | 0,001209718 | 1,791616283 |
| MAP3K6 | 339,2980924 | 347,1297674 | 318,1862778 | 179,2397551 | 213,2098319 | 189,5931967 | 273,9654077 | -0,786514694 | 0,191686972 | -4,103120248 | 4,07615E-05 | 0,001209718 | 1,724902354 |
| MON1A | 192,1535115 | 148,5820595 | 118,8077518 | 78,19183946 | 67,3294206 | 89,29227973 | 132,1051712 | -0,962866657 | 0,234658958 | -4,103259749 | 4,07369E-05 | 0,001209718 | 1,949179098 |
| ZBTB7A | 981,5409102 | 800,765259 | 798,8797105 | 506,4425295 | 436,5190769 | 516,1827678 | 737,1952624 | -0,82216286 | 0,200420508 | -4,102189285 | 4,09259E-05 | 0,0012099 | 1,768054639 |
| C6orf1 | 1516,45474 | 794,1908316 | 731,9649997 | 495,6159671 | 481,4053573 | 538,2000422 | 604,3649586 | -1,003765456 | 0,244855707 | -4,099416227 | 4,14194E-05 | 0,001216134 | 2,005226849 |
| LSM10 | 624,9316906 | 356,3339658 | 359,1544681 | 206,9076367 | 187,4002207 | 239,7436552 | 339,5459286 | -1,077098313 | 0,262713432 | -4,099898141 | 4,13332E-05 | 0,001216134 | 2,109788401 |
| SGTA | 3680,345635 | 2237,935092 | 1984,226016 | 1277,534362 | 1462,170584 | 1422,560566 | 2191,14011 | -0,924142646 | 0,22568022 | -4,094920885 | 4,22313E-05 | 0,001235484 | 1,897556241 |
| TRNP1 | 1236,014479 | 1217,583957 | 1152,571753 | 739,8150965 | 645,2402808 | 890,4764335 | 538,3258305 | -0,664576189 | 0,162390526 | -4,092456664 | 4,26827E-05 | 0,001245448 | 1,58510256 |
| COMMD5 | 166,1868208 | 101,2461822 | 103,786082 | 40,9003468 | 53,86353648 | 59,93591379 | 67,69073415 | -1,254464125 | 0,307467278 | -4,079992297 | 4,50372E-05 | 0,001303989 | 2,385785145 |
| MAML3 | 24,23557803 | 24,98282417 | 21,84970148 | 9,623611011 | 4,48862804 | 2,446363828 | 36,93372256 | -2,102285995 | 0,515991409 | -4,074265497 | 4,61598E-05 | 0,001332657 | 4,293892268 |
| SDC4 | 2794,015924 | 1978,902651 | 2104,399374 | 1520,53054 | 1458,804113 | 1477,603752 | 4749,50107 | -0,624742401 | 0,153499156 | -4,070005438 | 4,7012E-05 | 0,001347279 | 1,541935482 |
| NOP9 | 302,9447254 | 278,7557223 | 245,8091417 | 153,9777762 | 161,5906095 | 160,2368308 | 181,8852152 | -0,796001814 | 0,196322016 | -4,054572322 | 5,02261E-05 | 0,001428456 | 1,736282638 |
| ISG20 | 45,00893063 | 63,11450317 | 35,50576491 | 21,65312477 | 14,58804113 | 22,01727445 | 190,316148 | -1,314284775 | 0,326462375 | -4,025838425 | 5,67727E-05 | 0,001570897 | 2,48679016 |
| INPP5E | 162,7245953 | 126,2290063 | 159,7759421 | 70,97413121 | 72,94020565 | 94,18500739 | 95,25253994 | -0,912176575 | 0,226806296 | -4,021830931 | 5,77475E-05 | 0,001593945 | 1,88188253 |
| NRIP3 | 1175,425534 | 1470,04197 | 1749,341725 | 904,619435 | 890,992666 | 842,7723388 | 727,4460832 | -0,737083604 | 0,183570061 | -4,015271329 | 5,93774E-05 | 0,001622983 | 1,666803003 |
| KCTD12 | 479,5182224 | 243,2538143 | 159,7759421 | 116,6862835 | 122,3151141 | 113,755918 | 145,280831 | -1,317609177 | 0,328433749 | -4,011795927 | 6,02586E-05 | 0,001639092 | 2,492527076 |
| TOB2 | 498,5604623 | 596,9580092 | 432,8972106 | 292,3171845 | 283,9057235 | 225,0654722 | 317,0386636 | -0,931678644 | 0,232893826 | -4,000443722 | 6,32238E-05 | 0,001707347 | 1,907494165 |
| EEFSEC | 185,2290606 | 213,0114482 | 208,9377704 | 104,6567697 | 134,6588412 | 121,0950095 | 151,14369 | -0,754228888 | 0,18856613 | -3,999811039 | 6,33931E-05 | 0,001707813 | 1,686729804 |

| | | | | | | | | | | | | | |
|---|---|---|---|---|---|---|---|---|---|---|---|---|---|
| MCFD2 | 2934,236054 | 3154,410273 | 2781,74012 | 1971,637306 | 1849,314753 | 1360,178289 | 2329,037752 | -0,775664829 | 0,194036546 | -3,997519267 | 6,40098E-05 | 0,001720301 | 1,711978792 |
| SIGLEC15 | 2376,81776 | 2202,433184 | 1929,601762 | 1041,755892 | 1299,457818 | 1528,977393 | 1161,735229 | -0,749745984 | 0,187745625 | -3,993413866 | 6,51287E-05 | 0,001743946 | 1,681496743 |
| FAM104A | 917,4897397 | 499,6564834 | 503,9087404 | 268,2581569 | 256,9739553 | 291,1172956 | 447,5488873 | -1,232915124 | 0,308951247 | -3,990646214 | 6,58935E-05 | 0,001758308 | 2,350414374 |
| SMG9 | 733,9917917 | 578,5496124 | 559,8986005 | 442,6861065 | 363,5788713 | 375,5168476 | 674,8666929 | -0,661622096 | 0,166192301 | -3,981063455 | 6,86076E-05 | 0,001822076 | 1,581860191 |
| RIN1 | 832,6652166 | 741,5954122 | 670,5127142 | 490,8041616 | 435,3969199 | 550,4318614 | 578,0053438 | -0,603325572 | 0,151934498 | -3,970958412 | 7,15841E-05 | 0,001874535 | 1,519214491 |
| NR2C2AP | 366,9958959 | 262,9770965 | 204,8409514 | 133,5276028 | 136,9031552 | 157,7904669 | 166,5557983 | -0,958482033 | 0,241746293 | -3,964826185 | 7,34494E-05 | 0,001910026 | 1,943264165 |
| B3GAT3 | 204,2713005 | 228,790074 | 228,0562592 | 131,1217 | 121,1929571 | 154,1209212 | 233,8856587 | -0,706339723 | 0,178296253 | -3,961607228 | 7,44469E-05 | 0,001929833 | 1,631659161 |
| PRR14 | 929,6075287 | 483,8778576 | 491,6182834 | 333,2175313 | 314,2039628 | 331,4822987 | 396,329927 | -0,957441625 | 0,242070857 | -3,95521227 | 7,64667E-05 | 0,001974775 | 1,941863275 |
| NPEPL1 | 846,5141183 | 552,2519027 | 576,2858766 | 384,9444404 | 340,0135741 | 244,6363828 | 455,0932253 | -1,024449787 | 0,259798333 | -3,943250038 | 8,03848E-05 | 0,002061741 | 2,034183442 |
| USB1 | 1081,945448 | 1250,456094 | 1442,080298 | 843,2689148 | 713,6918584 | 522,2986773 | 713,1523487 | -0,860628824 | 0,218399727 | -3,94061309 | 8,12736E-05 | 0,002073293 | 1,815829599 |
| ZNF384 | 370,4581213 | 485,1927431 | 482,059039 | 223,748956 | 232,2865011 | 311,9113881 | 352,4208316 | -0,803791612 | 0,205105357 | -3,918920618 | 8,89464E-05 | 0,002250504 | 1,745683011 |
| FADD | 2051,368569 | 1773,780516 | 1655,114887 | 1342,493736 | 1159,188191 | 1144,898272 | 1447,005381 | -0,587008375 | 0,149872694 | -3,916713318 | 8,97644E-05 | 0,002261016 | 1,502128647 |
| MIDN | 351,4158814 | 314,2576304 | 315,4550652 | 188,8633661 | 188,5223777 | 242,190019 | 299,2741102 | -0,662209699 | 0,169196671 | -3,91384591 | 9,08376E-05 | 0,002281303 | 1,582504606 |
| TMEM173 | 327,1803034 | 176,1946547 | 196,6473133 | 105,8597211 | 104,3606019 | 62,38227762 | 173,1094447 | -1,355598318 | 0,347070135 | -3,90583395 | 9,3901E-05 | 0,002344186 | 2,559032231 |
| TRUB2 | 675,1339594 | 648,238543 | 610,4260352 | 419,8300304 | 370,3118133 | 414,6586689 | 678,5615648 | -0,6824378 | 0,174793731 | -3,904246441 | 9,45194E-05 | 0,002354393 | 1,604849267 |
| SIX5 | 112,5223266 | 119,6545789 | 121,5389645 | 70,97413121 | 61,71863555 | 63,60545953 | 102,6990087 | -0,852504775 | 0,219009588 | -3,892545441 | 9,91979E-05 | 0,002460021 | 1,805633105 |
| HIST1H2AM | 11903,13104 | 9145,028532 | 8203,1973 | 5568,461921 | 5572,631712 | 6733,616437 | 6500,607768 | -0,710438143 | 0,182751048 | -3,887464126 | 0,000101297 | 0,002501033 | 1,636300982 |
| C1orf106 | 522,7960403 | 473,3587738 | 533,95208 | 317,5791634 | 335,524946 | 346,1604817 | 238,7242004 | -0,614029404 | 0,158146534 | -3,88266114 | 0,00010332 | 0,002536389 | 1,530527963 |
| MOSPD3 | 166,1868208 | 81,52289993 | 102,4204757 | 50,52395781 | 57,23000751 | 35,47227551 | 95,39795807 | -1,277362387 | 0,329267601 | -3,879405029 | 0,000104712 | 0,002562827 | 2,423954112 |
| BTG1 | 1080,214335 | 1015,091593 | 800,2453168 | 548,5458276 | 701,3481313 | 560,2173167 | 1397,170895 | -0,676304143 | 0,17454427 | -3,874685444 | 0,000106763 | 0,0026073 | 1,59804069 |
| CPEB4 | 110,7912138 | 131,4885483 | 129,7326026 | 83,00364497 | 51,61922246 | 40,36500317 | 97,93440064 | -1,091534275 | 0,282030458 | -3,870270905 | 0,000108714 | 0,0026073 | 2,131005439 |
| PRKCD | 533,1827166 | 554,8816737 | 587,2107273 | 364,494267 | 291,7608226 | 332,7054806 | 322,248817 | -0,761407416 | 0,196558242 | -3,873698752 | 0,000107196 | 0,0026073 | 1,695143509 |
| TRMT10C | 190,4223988 | 294,5343481 | 320,9174905 | 144,3541652 | 143,6360973 | 105,1936446 | 255,3572482 | -1,038470588 | 0,268189972 | -3,872145482 | 0,000107882 | 0,0026073 | 2,054048985 |
| GALE | 204,2713005 | 119,6545789 | 109,2485074 | 61,3505202 | 62,84079256 | 78,2836425 | 202,5963477 | -1,088370127 | 0,282130958 | -3,857677069 | 0,00011447 | 0,002712981 | 2,126336796 |
| BNIP2 | 635,3183669 | 694,2595349 | 1037,86082 | 396,9739542 | 346,7465161 | 434,2295795 | 733,393754 | -1,008144844 | 0,261404754 | -3,856643121 | 0,000114955 | 0,002718742 | 2,011323085 |
| LYPD6 | 57,12671964 | 55,22519027 | 57,35546639 | 24,05902753 | 17,95451216 | 31,80272977 | 132,912659 | -1,207074609 | 0,313773866 | -3,84695712 | 0,000119594 | 0,002816599 | 2,308690225 |
| HSD11B1 | 285,6335982 | 190,658395 | 245,8091417 | 120,2951376 | 108,84923 | 108,8631904 | 91,91745181 | -1,092493705 | 0,284104554 | -3,845393146 | 0,000120359 | 0,002828695 | 2,132423085 |
| DNAJB1 | 1196,198887 | 1196,545789 | 998,2582365 | 594,2579799 | 640,7516528 | 365,7313923 | 797,3520916 | -1,08248254 | 0,281894192 | -3,840031364 | 0,000123019 | 0,002885158 | 2,117676974 |
| TMEM8B | 105,5978757 | 64,42938865 | 76,47395519 | 40,9003468 | 38,15333834 | 37,91863934 | 66,05907923 | -1,062147356 | 0,27676918 | -3,837664863 | 0,00012421 | 0,002907028 | 2,088037115 |
| COX7A2L | 1938,846242 | 2005,200361 | 1468,026818 | 1157,239224 | 1173,776233 | 955,3050749 | 1434,996733 | -0,719336871 | 0,187673701 | -3,832912483 | 0,000126635 | 0,002951489 | 1,646425088 |
| UCK1 | 998,8520373 | 431,2824383 | 495,7151024 | 334,4204826 | 337,76926 | 262,9841115 | 511,8704877 | -1,038767096 | 0,271726539 | -3,822840054 | 0,000131923 | 0,003049438 | 2,054471185 |
| ARL2 | 1312,183439 | 1111,078233 | 1264,551473 | 903,4164837 | 737,2571556 | 861,1200675 | 916,6757697 | -0,559425798 | 0,146546465 | -3,817395374 | 0,000134868 | 0,003104727 | 1,473682566 |
| FAM109A | 245,8180057 | 201,1774789 | 225,3250465 | 122,7010404 | 113,337858 | 151,6745574 | 142,2194353 | -0,792823351 | 0,2076649 | -3,81780142 | 0,000134646 | 0,003104727 | 1,732461571 |
| TMED9 | 3680,345635 | 3251,711799 | 2630,157816 | 2075,091124 | 1908,789074 | 2273,895178 | 2932,123352 | -0,611350662 | 0,160350194 | -3,812596965 | 0,000137514 | 0,003159172 | 1,527688773 |
| NPHP4 | 128,102341 | 102,5610677 | 107,8829011 | 57,74166607 | 51,61922246 | 77,06046059 | 152,54255 | -0,857754102 | 0,225151474 | -3,809675704 | 0,000139149 | 0,003190208 | 1,81221497 |
| AGXT2 | 31,16002889 | 23,66793869 | 15,02166977 | 6,014756882 | 4,48862804 | 9,785455313 | 52,028214 | -1,771766023 | 0,465316595 | -3,807657071 | 0,00014029 | 0,003209804 | 3,414717011 |
| CRIP2 | 1407,394638 | 1325,404567 | 1380,628012 | 774,7006864 | 789,9985351 | 676,4195985 | 846,9080478 | -0,87590704 | 0,230170118 | -3,805476781 | 0,000141531 | 0,003231631 | 1,835161512 |
| SMIM3 | 128,102341 | 213,0114482 | 208,9377704 | 109,4685753 | 95,38334586 | 101,5240989 | 135,3812523 | -0,85364298 | 0,224563798 | -3,801338364 | 0,000143917 | 0,00327943 | 1,807058211 |
| EIF2C1 | 534,9138294 | 515,4351092 | 404,2194774 | 292,3171845 | 332,158475 | 238,5204733 | 527,6775698 | -0,751012274 | 0,197723544 | -3,798294612 | 0,000145695 | 0,003306543 | 1,682973282 |
| CEP85 | 460,4759825 | 308,9980884 | 299,067789 | 232,1696156 | 189,6445347 | 206,7177435 | 317,1856576 | -0,760761641 | 0,200532452 | -3,793708371 | 0,000148414 | 0,003361457 | 1,694384904 |
| PML | 732,260679 | 502,2862544 | 644,5661937 | 323,5939202 | 366,9453423 | 342,490936 | 458,722797 | -0,861469827 | 0,227304841 | -3,78993172 | 0,000150689 | 0,003392459 | 1,816888426 |
| ATP6V0E2 | 529,7204912 | 270,8664094 | 279,9493002 | 134,7305542 | 155,9798244 | 188,3700148 | 188,5129245 | -1,169984119 | 0,309295057 | -3,78274431 | 0,000155109 | 0,003471098 | 2,2500922 |
| CCDC94 | 870,7496963 | 578,5496124 | 514,8335912 | 374,1178781 | 320,9369049 | 379,1863934 | 420,6945719 | -0,868435417 | 0,229551645 | -3,783180983 | 0,000154837 | 0,003471098 | 1,825681899 |
| VASP | 1261,98117 | 938,8282347 | 766,1051582 | 501,630724 | 487,0161424 | 250,7522924 | 522,7056445 | -1,258363248 | 0,332749134 | -3,781717571 | 0,00015575 | 0,00347852 | 2,392241847 |
| CUEDC2 | 57,12671964 | 69,68893058 | 57,35546639 | 27,66788166 | 25,80961123 | 8,562273399 | 48,84468893 | -1,566847534 | 0,414612921 | -3,779061037 | 0,000157421 | 0,003494991 | 2,96256649 |
| TBC1D16 | 122,9090029 | 80,20801445 | 121,5389645 | 44,50920093 | 59,47432153 | 25,6868202 | 82,06326814 | -1,316073832 | 0,348474632 | -3,776670413 | 0,000158939 | 0,003516035 | 2,48987589 |
| H6PD | 154,0690318 | 119,6545789 | 102,4204757 | 63,75642295 | 70,69589163 | 69,72136911 | 124,7192113 | -0,87199967 | 0,230960219 | -3,77554054 | 0,000159661 | 0,003523836 | 1,830197918 |
| PTGES2 | 979,8097975 | 604,847322 | 610,4260352 | 427,0477386 | 406,2208376 | 387,7486668 | 518,9966145 | -0,843718775 | 0,223521417 | -3,774666359 | 0,000160222 | 0,003529281 | 1,794670232 |
| DCAF7 | 1637,63263 | 1289,902659 | 1580,006538 | 773,497735 | 748,4787257 | 544,3159518 | 1044,236568 | -1,124973839 | 0,29834258 | -3,770745163 | 0,000162761 | 0,00357819 | 2,180975917 |
| RASSF7 | 346,2225433 | 285,3301497 | 254,0027797 | 157,5866303 | 179,5451216 | 162,6831946 | 210,5680958 | -0,821483261 | 0,21790725 | -3,769875761 | 0,000163329 | 0,003583664 | 1,767221972 |

| | | | | | | | | | | | | | | |
|---|---|---|---|---|---|---|---|---|---|---|---|---|---|---|
| MMP14 | 10481,8875 | 8557,274721 | 7588,674446 | 5323,05984 | 6206,650423 | 5415,026334 | 6473,114653 | -0,651892618 | 0,173072158 | -3,766594372 | 0,00016549 | 0,003623995 | 1,57122808 |
| FOSL1 | 8690,185836 | 6811,1068 | 5665,900716 | 3879,518189 | 3468,587318 | 1995,009702 | 3496,613458 | -1,179735121 | 0,313291889 | -3,765610158 | 0,000166143 | 0,003631223 | 2,265351814 |
| PQBP1 | 687,2517484 | 452,3206061 | 632,2757366 | 383,7414891 | 357,9680862 | 357,1691189 | 607,2075475 | -0,686842334 | 0,182773344 | -3,757891159 | 0,000171351 | 0,003716142 | 1,609756342 |
| SDSL | 53,66449421 | 36,81679352 | 60,08667908 | 19,24722202 | 15,71019814 | 23,24045637 | 39,60985548 | -1,371071968 | 0,365001823 | -3,756342791 | 0,000172414 | 0,003727656 | 2,586626893 |
| ARG2 | 169,6490462 | 114,395037 | 124,2701772 | 69,77117983 | 53,86353648 | 28,13318402 | 149,8004138 | -1,423070718 | 0,380082943 | -3,744105709 | 0,000181037 | 0,003873856 | 2,681556624 |
| FAM221A | 79,63118495 | 72,31870155 | 61,45228542 | 32,47968716 | 39,27549535 | 24,46363828 | 44,36144762 | -1,139839911 | 0,304753672 | -3,74020075 | 0,000183873 | 0,003919607 | 2,203565699 |
| HIST1H1D | 8350,887744 | 7204,25756 | 5907,613038 | 3961,318882 | 4240,631341 | 4394,892617 | 5046,382132 | -0,768639738 | 0,205692019 | -3,736847644 | 0,000186342 | 0,003964703 | 1,703662707 |
| GAS5 | 1661,868208 | 1145,265255 | 1141,646902 | 840,8630121 | 830,3961875 | 620,1532305 | 757,0237671 | -0,783770083 | 0,209868694 | -3,734573605 | 0,000188033 | 0,003978095 | 1,721623986 |
| LZTS2 | 396,424812 | 412,8740416 | 419,2411472 | 275,4758652 | 247,9966992 | 304,5722966 | 297,8117165 | -0,571032504 | 0,153801345 | -3,712792663 | 0,000204985 | 0,004256561 | 1,485586391 |
| CNOT3 | 548,7627311 | 549,6221318 | 520,2960166 | 368,1031212 | 315,3261198 | 349,8300274 | 475,1976186 | -0,647848634 | 0,175674229 | -3,687784121 | 0,000226215 | 0,004578927 | 1,566829975 |
| SPRY2 | 1317,376777 | 838,896938 | 1016,011119 | 665,2321111 | 685,6379332 | 636,0545953 | 633,0124892 | -0,6729656 | 0,18258845 | -3,685696448 | 0,000228078 | 0,004600052 | 1,594346936 |
| NXT1 | 46,74004334 | 23,66793869 | 28,6777332 | 9,623611011 | 12,34372711 | 9,785455313 | 25,14172848 | -1,615163925 | 0,438664151 | -3,68200575 | 0,000231406 | 0,004658812 | 3,063464058 |
| DNASE1L1 | 806,6985258 | 565,4007576 | 544,8769307 | 381,3355863 | 419,6867218 | 371,8473019 | 359,546189 | -0,70556282 | 0,19194291 | -3,675899352 | 0,000237013 | 0,004754649 | 1,630780736 |
| SLC25A28 | 171,3801589 | 109,1354951 | 96,95805033 | 69,77117983 | 54,98569349 | 61,15909571 | 95,53493827 | -1,012648964 | 0,275989998 | -3,66915096 | 0,000243357 | 0,00487322 | 2,017612284 |
| TP53I11 | 86,55563582 | 136,7480902 | 188,4536753 | 50,52395781 | 67,3294206 | 42,81136699 | 47,79415675 | -1,360574541 | 0,371031409 | -3,66700637 | 0,000245407 | 0,004896801 | 2,567874226 |
| ITPR3 | 900,1786125 | 995,3683104 | 890,3753354 | 643,5789864 | 726,0355855 | 648,2864145 | 1185,46956 | -0,465524416 | 0,127049828 | -3,664108992 | 0,000248201 | 0,004943778 | 1,380819186 |
| SCAF1 | 1036,936517 | 1193,916018 | 972,311716 | 741,0180479 | 697,9816603 | 782,836425 | 961,5077548 | -0,528494763 | 0,144288479 | -3,662764818 | 0,000249508 | 0,004961006 | 1,442423456 |
| ARMC6 | 607,6205634 | 585,1240398 | 465,6717628 | 383,7414891 | 325,4255329 | 314,3577519 | 411,6202989 | -0,695258237 | 0,189855266 | -3,662043463 | 0,000250211 | 0,00496621 | 1,619174233 |
| MAD2L1 | 2290,262124 | 1942,085858 | 1696,083078 | 1319,63766 | 1352,199197 | 1137,55918 | 1585,813892 | -0,637304617 | 0,174256982 | -3,657268769 | 0,000254917 | 0,005041792 | 1,555420455 |
| ZNF710 | 479,5182224 | 247,1984707 | 226,6906529 | 166,0072899 | 152,6133534 | 166,3527403 | 240,9877044 | -0,969472357 | 0,265810166 | -3,647235821 | 0,000265077 | 0,005187928 | 1,958124311 |
| MED8 | 792,8496241 | 695,5744204 | 615,8884605 | 555,7635359 | 438,7633909 | 425,6673061 | 964,6828988 | -0,565586961 | 0,155166848 | -3,645024477 | 0,000267366 | 0,005223641 | 1,479989521 |
| HIST1H2BH | 5293,742687 | 8235,127778 | 6994,635687 | 4384,757767 | 3822,066776 | 2567,458838 | 4556,892237 | -0,929793348 | 0,25532636 | -3,641587756 | 0,000270962 | 0,005284695 | 1,905003103 |
| MICAL1 | 540,1071675 | 295,8492336 | 312,7238525 | 200,8928799 | 222,187088 | 151,6745574 | 228,6117708 | -0,994326036 | 0,273106788 | -3,640795755 | 0,000271797 | 0,005291792 | 1,992149663 |
| C10orf35 | 135,0267919 | 122,2843499 | 90,13001862 | 67,36527708 | 48,25275143 | 61,15909571 | 76,70286532 | -0,972260571 | 0,267327366 | -3,636966106 | 0,000275868 | 0,005341508 | 1,961912324 |
| CDC42EP4 | 240,6246676 | 157,7862579 | 154,3135167 | 102,250867 | 105,4827589 | 110,0863723 | 159,2529909 | -0,789000417 | 0,216923441 | -3,637229858 | 0,000275586 | 0,005341508 | 1,727876874 |
| SH2D1B | 1,731112716 | 5,259541931 | 9,559244399 | 0 | 0 | 0 | 22,15761772 | -4,720212408 | 1,297971274 | -3,63660776 | 0,000276252 | 0,005341508 | 26,35879307 |
| HES1 | 102,1356503 | 67,05915962 | 71,01152982 | 46,91510368 | 34,78686731 | 23,24045637 | 54,12536797 | -1,184280796 | 0,325753379 | -3,635513469 | 0,000277427 | 0,005347896 | 2,27250079 |
| PXN | 1950,964031 | 1994,681277 | 1709,739141 | 1265,504848 | 1447,582543 | 1367,51738 | 1893,432453 | -0,470488034 | 0,129469097 | -3,633979417 | 0,000279083 | 0,00535935 | 1,385578101 |
| HDAC5 | 31,16002889 | 43,39122093 | 34,14015857 | 13,23246514 | 21,32098319 | 12,23181914 | 153,1083781 | -1,217562172 | 0,335288418 | -3,631387505 | 0,000281902 | 0,005395029 | 2,325534224 |
| NME3 | 768,6140461 | 377,3721335 | 357,7888618 | 222,5460046 | 291,7608226 | 228,7350179 | 327,737312 | -1,01292442 | 0,278932051 | -3,6314379 | 0,000281846 | 0,005395029 | 2,017997546 |
| CCDC71 | 219,851315 | 106,5057241 | 169,3351865 | 73,38003396 | 54,98569349 | 78,2836425 | 105,2105068 | -1,258849665 | 0,346804519 | -3,629853697 | 0,000283582 | 0,005408975 | 2,393048548 |
| NUDT22 | 270,0535838 | 194,6030514 | 202,1097387 | 131,1217 | 120,0708001 | 140,6659201 | 213,679244 | -0,761814255 | 0,20987524 | -3,629843408 | 0,000283593 | 0,005408975 | 1,695621607 |
| NPAS2 | 986,7342483 | 887,5477008 | 742,8898504 | 547,3428763 | 629,5300827 | 486,8264018 | 1241,124778 | -0,652001423 | 0,17965312 | -3,629224036 | 0,000284274 | 0,005412778 | 1,571346583 |
| FASN | 3444,914306 | 3046,589663 | 2643,813879 | 2053,437999 | 1912,155545 | 1355,285561 | 1832,786354 | -0,779472674 | 0,215013185 | -3,625231977 | 0,000288702 | 0,005474383 | 1,716503351 |
| PLXNA2 | 869,0185836 | 637,7194591 | 473,8654009 | 301,9407955 | 379,2890694 | 325,3663892 | 468,4820724 | -0,974497768 | 0,269074467 | -3,621665707 | 0,000292712 | 0,005526603 | 1,964957036 |
| SCAF4 | 1083,67656 | 812,5992283 | 815,2669866 | 613,505202 | 647,4845948 | 518,6291316 | 710,2010161 | -0,605083449 | 0,167146163 | -3,620085782 | 0,000294505 | 0,005532564 | 1,521066733 |
| PPP6R1 | 2240,059855 | 2130,114482 | 1918,676911 | 1512,10988 | 1319,656644 | 1252,53828 | 1618,051578 | -0,622459573 | 0,172107234 | -3,616696172 | 0,000298387 | 0,00559613 | 1,539497552 |
| CORO1A | 55,39560692 | 72,31870155 | 79,20516787 | 34,88558992 | 38,15333834 | 26,91000211 | 55,07028142 | -1,055604046 | 0,292179326 | -3,612863579 | 0,000302834 | 0,005660628 | 2,078588315 |
| TCF19 | 474,3248843 | 465,4694609 | 419,2411472 | 273,0699624 | 286,1500376 | 248,3059286 | 320,4367867 | -0,750086767 | 0,208040835 | -3,605478538 | 0,000311578 | 0,005766507 | 1,68189398 |
| ZNF503 | 77,90007224 | 74,94847251 | 75,10834885 | 46,91510368 | 30,29823927 | 39,14182125 | 100,7066324 | -0,972827769 | 0,269798297 | -3,60575949 | 0,000311241 | 0,005766507 | 1,962683806 |
| SERPINB2 | 17,31112716 | 18,40839676 | 6,828031713 | 2,405902753 | 3,36647103 | 2,446363828 | 46,84776139 | -2,350582068 | 0,652267314 | -3,603709733 | 0,000313707 | 0,005786845 | 5,100299855 |
| LAMB3 | 11397,64612 | 7154,291911 | 7002,829325 | 4379,945961 | 4573,911973 | 5892,06728 | 4468,102422 | -0,783336172 | 0,21780841 | -3,596445939 | 0,000322595 | 0,005918918 | 1,721106261 |
| PPM1M | 183,4979479 | 114,395037 | 116,0765391 | 63,75642295 | 61,71863555 | 48,92727657 | 71,80744277 | -1,240849939 | 0,34556988 | -3,590735222 | 0,000329746 | 0,006013591 | 2,363377255 |
| PAGR1 | 1158,114407 | 800,765259 | 774,2987963 | 514,8631891 | 474,6724153 | 590,7968645 | 799,9934706 | -0,789016752 | 0,219764602 | -3,590281357 | 0,000330321 | 0,006014309 | 1,727896439 |
| TUBA4A | 2660,720245 | 2374,683182 | 2195,894999 | 1666,087656 | 1507,056865 | 1173,031456 | 2095,366804 | -0,734173888 | 0,204604755 | -3,588254275 | 0,0003329 | 0,00604167 | 1,66344468 |
| SIGMAR1 | 1002,314263 | 1171,562965 | 1119,797201 | 850,4866231 | 789,9985351 | 770,6046059 | 719,0787667 | -0,451383315 | 0,126004494 | -3,582279491 | 0,000340609 | 0,006151773 | 1,3673507 |
| C9orf89 | 476,055997 | 449,6908351 | 344,1327983 | 250,2138863 | 297,3716077 | 261,7609296 | 318,7970427 | -0,647150606 | 0,18073396 | -3,58068073 | 0,0003427 | 0,006163326 | 1,56607207 |
| COQ5 | 285,6335982 | 222,2156466 | 307,2614271 | 156,3836789 | 172,8121796 | 149,2281935 | 330,5408876 | -0,766450633 | 0,214371818 | -3,575332987 | 0,000349782 | 0,006257095 | 1,701079578 |
| SNORA43 | 2137,924205 | 1596,270976 | 1593,662602 | 1062,206065 | 934,7567894 | 1014,017807 | 1168,411451 | -0,822827592 | 0,230194394 | -3,574490135 | 0,000350911 | 0,006267305 | 1,768869471 |
| ZWINT | 2108,495289 | 1982,847308 | 1637,362005 | 1218,589744 | 1058,19406 | 1323,482831 | 1505,794122 | -0,669929179 | 0,187539135 | -3,572210038 | 0,000353981 | 0,006301234 | 1,590994865 |

| | | | | | | | | | | | | | |
|---|---|---|---|---|---|---|---|---|---|---|---|---|---|
| SLC39A4 | 1345,074581 | 945,4026621 | 811,1701675 | 672,4498194 | 594,7432153 | 639,7241411 | 457,6467945 | -0,700006762 | 0,19601768 | -3,571140944 | 0,00035543 | 0,006307892 | 1,624512407 |
| CCNE1 | 128,102341 | 90,72709831 | 99,68926301 | 57,74166607 | 49,37490844 | 57,48954996 | 95,38568903 | -0,945962582 | 0,265438738 | -3,563769894 | 0,000365566 | 0,00646736 | 1,926473822 |
| FURIN | 1526,841416 | 1180,767163 | 1158,034179 | 920,2578029 | 785,5099071 | 790,1755165 | 1144,152297 | -0,630021097 | 0,176957648 | -3,560293131 | 0,000370441 | 0,006522782 | 1,547587624 |
| ABCB8 | 150,6068063 | 103,8759531 | 91,49562496 | 37,29149267 | 51,61922246 | 69,72136911 | 72,07102633 | -1,118731068 | 0,314294699 | -3,55949709 | 0,000371566 | 0,006532345 | 2,171558876 |
| SH3BP5 | 1687,834898 | 1086,095409 | 908,1282179 | 642,376035 | 767,5553949 | 720,4541474 | 737,4477121 | -0,787686977 | 0,221514512 | -3,555915904 | 0,000376665 | 0,006601326 | 1,726304519 |
| CBX8 | 228,5068786 | 163,0457999 | 132,4638152 | 101,0479156 | 100,9941309 | 97,85455313 | 137,1309596 | -0,795846613 | 0,223844608 | -3,555353071 | 0,000377472 | 0,006605169 | 1,736095864 |
| DUS1L | 178,3046098 | 101,2461822 | 96,95805033 | 51,72690918 | 58,35216452 | 69,72136911 | 104,0256265 | -1,05651585 | 0,297426176 | -3,552195255 | 0,000382031 | 0,006674555 | 2,079902427 |
| PQLC1 | 155,8001445 | 132,8034338 | 148,8510913 | 66,1623257 | 77,42883369 | 89,29227973 | 94,88810743 | -0,907764572 | 0,255773845 | -3,549090694 | 0,000386564 | 0,006743259 | 1,876136209 |
| PCDH1 | 81,36229767 | 80,20801445 | 54,62425371 | 31,27673579 | 30,29823927 | 50,15045848 | 265,0898625 | -0,951737673 | 0,268973464 | -3,538407315 | 0,000402549 | 0,006967998 | 1,934200929 |
| THBD | 34,62225433 | 56,54007576 | 36,87137125 | 19,24722202 | 22,4431402 | 11,00863723 | 169,4223698 | -1,285124749 | 0,363607748 | -3,534371191 | 0,000408747 | 0,007053547 | 2,437031246 |
| CIT | 971,1542339 | 746,8549542 | 639,1037684 | 449,9038148 | 502,7263405 | 483,1568561 | 598,2550493 | -0,713188479 | 0,201972702 | -3,531113227 | 0,000413815 | 0,00713005 | 1,639423381 |
| CYB5D2 | 143,6823555 | 117,024808 | 99,68926301 | 73,38003396 | 65,08510658 | 53,82000422 | 91,28277295 | -0,899133773 | 0,254808284 | -3,528667753 | 0,000417657 | 0,007174251 | 1,864945891 |
| POLR3K | 1137,341055 | 800,765259 | 730,5993933 | 541,3281194 | 508,3371256 | 551,6550433 | 790,7799003 | -0,735155425 | 0,208676722 | -3,522939294 | 0,000426789 | 0,007319922 | 1,664576789 |
| ESYT2 | 1914,610664 | 1606,79006 | 1455,736361 | 1123,556586 | 1163,676819 | 897,815525 | 1027,863378 | -0,643101397 | 0,183085294 | -3,512578109 | 0,000443782 | 0,007530879 | 1,561682741 |
| HYAL2 | 479,5182224 | 382,6316755 | 329,1111286 | 277,8817679 | 191,8888487 | 190,8163786 | 348,8825447 | -0,848803102 | 0,242048483 | -3,506748281 | 0,000453618 | 0,007651569 | 1,801006143 |
| SRC | 948,6497686 | 664,0171688 | 523,0272292 | 461,9333285 | 373,6782844 | 418,3282146 | 535,1034452 | -0,766047926 | 0,21844083 | -3,506889836 | 0,000453377 | 0,007651569 | 1,700604813 |
| PANK3 | 1855,752832 | 1466,097313 | 1338,294216 | 1069,423774 | 1066,04916 | 736,3555123 | 1130,438782 | -0,697356651 | 0,198901065 | -3,506047851 | 0,000454813 | 0,007659826 | 1,621531052 |
| SUMO2 | 9919,275865 | 8459,973196 | 7624,180211 | 6418,948544 | 5773,497817 | 5485,970885 | 7517,25565 | -0,556549091 | 0,158833508 | -3,503977827 | 0,000458364 | 0,007696944 | 1,470746998 |
| RMND5B | 247,5491184 | 209,0667918 | 238,98111 | 144,3541652 | 169,4457085 | 130,8804648 | 232,8351485 | -0,641080354 | 0,183477741 | -3,494049746 | 0,000475752 | 0,007941459 | 1,559496542 |
| SDF2L1 | 266,5913583 | 332,6660271 | 278,5836939 | 206,9076367 | 171,6900225 | 110,0863723 | 173,8752846 | -0,84587923 | 0,242231225 | -3,492032175 | 0,000479361 | 0,007989571 | 1,797359787 |
| HIST1H2BE | 1132,147716 | 791,5610606 | 606,3292161 | 502,8336753 | 528,5359517 | 475,8177646 | 751,2884256 | -0,744914268 | 0,213823398 | -3,483782769 | 0,000494381 | 0,008179487 | 1,675874678 |
| RTN3 | 119,4467774 | 67,05915962 | 84,66759324 | 27,66788166 | 49,37490844 | 46,48091274 | 57,01354255 | -1,122435712 | 0,322409164 | -3,481401396 | 0,000498797 | 0,008240426 | 2,177142308 |
| ALAS1 | 335,835867 | 365,5381642 | 458,8437311 | 233,372567 | 199,7439478 | 277,6622945 | 288,0864806 | -0,709883994 | 0,203942375 | -3,480806734 | 0,000499906 | 0,008246615 | 1,635672588 |
| SEMA7A | 8556,890157 | 8216,719381 | 5299,918216 | 4566,403425 | 4603,088055 | 4807,104923 | 5900,882068 | -0,659174961 | 0,189429249 | -3,479795038 | 0,000501798 | 0,008265679 | 1,579179274 |
| SFN | 178,3046098 | 188,028624 | 187,0880689 | 110,6715266 | 97,62765988 | 129,6572829 | 251,4870833 | -0,714567534 | 0,20544607 | -3,478127045 | 0,000504931 | 0,008292967 | 1,640991236 |
| CAPN10 | 299,4824999 | 117,024808 | 127,0013899 | 76,98888809 | 87,52824679 | 74,61409676 | 117,4422105 | -1,175232907 | 0,338122885 | -3,475756767 | 0,000509414 | 0,008342212 | 2,258293357 |
| CYB5D1 | 124,6401156 | 166,9904563 | 146,1198787 | 93,83020736 | 75,18451967 | 83,17637016 | 123,4606674 | -0,802124145 | 0,231064709 | -3,471426461 | 0,000517701 | 0,008454392 | 1,743666516 |
| EMP1 | 7376,271284 | 7262,112521 | 6474,339671 | 4686,698562 | 4481,895098 | 3247,547982 | 5475,485053 | -0,765828287 | 0,221009698 | -3,465134307 | 0,000529967 | 0,00859112 | 1,700345929 |
| NRGN | 124,6401156 | 123,5992354 | 125,6357835 | 55,33576331 | 52,74137947 | 68,49818719 | 97,60928014 | -1,083532124 | 0,312694058 | -3,465150993 | 0,000529934 | 0,00859112 | 2,119218179 |
| UPRT | 275,2469219 | 166,9904563 | 188,4536753 | 119,0921863 | 126,8037421 | 122,3181914 | 163,4852483 | -0,76788286 | 0,22164788 | -3,464426822 | 0,000531363 | 0,008601338 | 1,702769153 |
| CYP1B1 | 34,62225433 | 31,55725159 | 45,06500931 | 18,04427065 | 10,09941309 | 20,79409254 | 358,9668833 | -1,198025304 | 0,3464447 | -3,458056381 | 0,000544088 | 0,00876941 | 2,294254288 |
| ZNF706 | 659,5539449 | 641,6641156 | 794,7828914 | 453,5126689 | 479,1610433 | 507,6204944 | 495,6897421 | -0,541976022 | 0,156761874 | -3,457320389 | 0,000545576 | 0,0087808 | 1,45596535 |
| IRF2BPL | 1351,999031 | 771,8377784 | 731,9649997 | 536,5163139 | 557,712034 | 395,0877583 | 687,7801736 | -0,937424688 | 0,271263458 | -3,455772094 | 0,000548719 | 0,008818755 | 1,915106585 |
| GPRC5C | 1438,554667 | 900,6965557 | 1102,044319 | 506,4425295 | 531,9024228 | 802,4073357 | 533,1404027 | -0,902035466 | 0,261154248 | -3,454033282 | 0,000552269 | 0,008850488 | 1,868700631 |
| CYTH2 | 901,9097252 | 887,5477008 | 830,2886563 | 584,6343689 | 572,3000751 | 489,2727657 | 728,7679657 | -0,669872763 | 0,193998239 | -3,452983735 | 0,000554422 | 0,008868346 | 1,590932651 |
| DHRS13 | 214,6579768 | 123,5992354 | 122,9045708 | 70,97413121 | 51,61922246 | 86,8459159 | 94,29534326 | -1,134447127 | 0,328566101 | -3,452721158 | 0,000554962 | 0,008868346 | 2,195344162 |
| MEAF6 | 1100,987688 | 681,11068 | 640,4693747 | 510,0513836 | 479,1610433 | 503,9509486 | 773,8659511 | -0,695386365 | 0,20145669 | -3,451790876 | 0,000556879 | 0,008880432 | 1,619318042 |
| C16orf52 | 458,7448698 | 351,0744239 | 378,2729569 | 265,8522542 | 300,7380787 | 217,7263807 | 574,3875621 | -0,594182224 | 0,172265147 | -3,449230643 | 0,000562186 | 0,008932957 | 1,509616633 |
| KIAA1217 | 160,9934826 | 170,9351128 | 178,8944309 | 99,84496424 | 74,06236266 | 92,96182547 | 79,70305178 | -0,93919047 | 0,272448113 | -3,447226927 | 0,000566373 | 0,008974091 | 1,91745201 |
| TMEM164 | 1092,332124 | 1055,853043 | 890,3753354 | 601,4756882 | 564,4449761 | 327,812753 | 594,9755004 | -1,024001349 | 0,297682289 | -3,43991358 | 0,0005819 | 0,009166926 | 2,033551248 |
| CD320 | 922,6830778 | 581,1793834 | 577,6514829 | 492,0071129 | 353,4794582 | 368,1777562 | 450,603462 | -0,776080906 | 0,225670916 | -3,438993908 | 0,00058388 | 0,009173863 | 1,712472601 |
| C21orf33 | 685,5206357 | 399,7251867 | 335,9391603 | 274,2729138 | 273,8063105 | 242,190019 | 439,5278981 | -0,84242918 | 0,245744257 | -3,428072709 | 0,000607883 | 0,009464942 | 1,793066731 |
| CENPM | 848,245231 | 503,6011399 | 434,262817 | 363,2913157 | 287,2721946 | 316,8041158 | 319,96571 | -0,882156573 | 0,257326217 | -3,428164379 | 0,000607677 | 0,009464942 | 1,843128392 |
| SETDB1 | 325,4491907 | 270,8664094 | 210,3033768 | 127,5128459 | 179,5451216 | 96,63137122 | 169,055611 | -0,994461952 | 0,290109523 | -3,427884555 | 0,000608304 | 0,009464942 | 1,992337352 |
| CCDC85B | 9766,937946 | 4218,152629 | 3609,297564 | 2668,146153 | 2991,670589 | 3076,302514 | 3780,203748 | -1,009711266 | 0,295091107 | -3,421693308 | 0,000622325 | 0,009590117 | 2,013508085 |
| COX4I1 | 4218,72169 | 3868,39309 | 3770,439112 | 2857,009519 | 2597,793478 | 3175,380249 | 3597,748188 | -0,458229992 | 0,133894572 | -3,422319415 | 0,000620893 | 0,009590117 | 1,373855234 |
| PRKCDBP | 6832,701891 | 3963,064845 | 2982,484252 | 2239,895463 | 2840,179392 | 2535,656108 | 2427,233002 | -0,854866067 | 0,249821827 | -3,421903034 | 0,000621845 | 0,009590117 | 1,808590846 |
| SLC39A13 | 498,5604623 | 507,5457963 | 565,3610259 | 304,3466982 | 315,3261198 | 415,8818508 | 394,3771881 | -0,603047498 | 0,176289594 | -3,420777624 | 0,000624424 | 0,00959877 | 1,518921697 |
| BBS1 | 244,086893 | 186,7137385 | 158,4103357 | 105,8597211 | 115,582172 | 136,9963744 | 226,4294264 | -0,71041359 | 0,207705353 | -3,420295048 | 0,000625533 | 0,009600044 | 1,636273134 |
| ARID3A | 160,9934826 | 89,41221282 | 88,76441227 | 56,53871469 | 63,96294957 | 62,38227762 | 165,2075908 | -0,874760856 | 0,256339274 | -3,412512028 | 0,000643671 | 0,009840031 | 1,833704102 |

| | | | | | | | | | | | | | |
|---|---|---|---|---|---|---|---|---|---|---|---|---|---|
| NUDT14 | 164,4557081 | 93,35686927 | 96,95805033 | 68,56822845 | 71,81804864 | 55,04318614 | 220,2765885 | -0,843472821 | 0,247174541 | -3,412458335 | 0,000643798 | 0,009840031 | 1,794364298 |
| PAX8 | 3418,947615 | 4170,816751 | 3323,885838 | 2421,541121 | 2675,222312 | 2494,067923 | 2769,790534 | -0,523978092 | 0,153540752 | -3,412632056 | 0,000643387 | 0,009840031 | 1,437914697 |
| NDUFS4 | 1639,363742 | 1220,213728 | 1127,990839 | 1032,132281 | 754,0895108 | 725,3468751 | 1368,560015 | -0,665849118 | 0,195167745 | -3,411676028 | 0,000645648 | 0,009854904 | 1,586501756 |
| HIST1H2AH | 16037,0282 | 9022,744182 | 7054,722366 | 5497,48779 | 6618,482045 | 5419,919062 | 6319,495333 | -0,872711909 | 0,255954099 | -3,409642238 | 0,000650481 | 0,009915209 | 1,831101684 |
| VPS28 | 815,3540894 | 865,1946476 | 916,3218559 | 656,8114515 | 441,007705 | 488,0495837 | 524,38767 | -0,712215533 | 0,209091243 | -3,406242766 | 0,000658636 | 0,010025901 | 1,638318135 |
| PSMB7 | 3022,522803 | 2863,820581 | 2672,491613 | 2134,035742 | 2032,226345 | 1821,31787 | 3111,255737 | -0,515174329 | 0,151283374 | -3,405359862 | 0,000660769 | 0,010044764 | 1,429166823 |
| CCDC109B | 519,3338149 | 447,0610641 | 562,6298132 | 291,1142331 | 223,309245 | 151,6745574 | 314,3069227 | -1,198500043 | 0,351998171 | -3,404847354 | 0,00066201 | 0,010050035 | 2,295009368 |
| PNKP | 765,1518206 | 568,0305285 | 553,0705688 | 451,1067661 | 344,5022021 | 326,5895711 | 385,8720395 | -0,747471656 | 0,21977786 | -3,40103255 | 0,000671318 | 0,010136546 | 1,678848046 |
| NDOR1 | 547,0316184 | 257,7175546 | 204,8409514 | 164,8043386 | 136,9031552 | 155,3441031 | 212,4912684 | -1,139333831 | 0,335047798 | -3,400511322 | 0,0006726 | 0,010142259 | 2,20279285 |
| ANKRD54 | 640,511705 | 482,5629722 | 491,6182834 | 368,1031212 | 356,8459292 | 354,7227551 | 336,629337 | -0,57748118 | 0,170261166 | -3,391737494 | 0,000694509 | 0,010388973 | 1,492241648 |
| GPR161 | 84,8245231 | 63,11450317 | 94,22683764 | 40,9003468 | 41,51980937 | 44,03454891 | 55,20243665 | -0,934218972 | 0,275433133 | -3,391817692 | 0,000694306 | 0,010388973 | 1,91085588 |
| SH3BP1 | 455,2826444 | 327,4064852 | 243,077929 | 194,878123 | 193,0110057 | 217,7263807 | 272,6817755 | -0,755654984 | 0,223035271 | -3,3880515 | 0,00070391 | 0,010515599 | 1,688397952 |
| SKP2 | 1386,621286 | 1510,80342 | 1470,758031 | 1119,947731 | 1045,850333 | 1113,095542 | 1584,404624 | -0,414567787 | 0,12257865 | -3,382055425 | 0,000719456 | 0,01066276 | 1,332899298 |
| GNB2 | 3065,800621 | 2694,200354 | 2260,078497 | 1887,43071 | 1643,96002 | 2012,134249 | 2205,175025 | -0,53243387 | 0,157715865 | -3,375905582 | 0,000735732 | 0,010860984 | 1,446367203 |
| RPS28 | 27472,75881 | 17144,79181 | 14164,06899 | 11856,28877 | 11566,0723 | 11342,56589 | 13766,5697 | -0,757628517 | 0,224415203 | -3,376012436 | 0,000735446 | 0,010860984 | 1,690709174 |
| HIST1H4I | 162,7245953 | 128,8587773 | 91,49562496 | 80,59774222 | 63,96294957 | 44,03454891 | 175,2287603 | -1,015514814 | 0,300916785 | -3,374736348 | 0,000738864 | 0,010892917 | 2,021624164 |
| ACACB | 147,1445809 | 107,8206096 | 118,8077518 | 86,6124991 | 69,57373462 | 56,26636805 | 93,68830315 | -0,807500023 | 0,239552825 | -3,370864115 | 0,000749328 | 0,011003858 | 1,750176017 |
| THEMIS2 | 24,23557803 | 35,50190803 | 24,58091417 | 4,811805506 | 11,2215701 | 12,23181914 | 485,1478976 | -1,582839496 | 0,470962585 | -3,360860391 | 0,000777001 | 0,011365661 | 2,995588583 |
| SELO | 401,6181502 | 262,9770965 | 228,0562592 | 181,6456578 | 166,0792375 | 102,7472808 | 186,9966748 | -0,982319987 | 0,292326862 | -3,360348008 | 0,000778443 | 0,011371956 | 1,975639868 |
| ALKBH5 | 5094,664724 | 3266,175539 | 2825,439523 | 2212,227581 | 2267,879317 | 2479,38974 | 2758,523942 | -0,684093259 | 0,203886615 | -3,355263217 | 0,000792895 | 0,011568029 | 1,60669185 |
| GM2A | 685,5206357 | 770,5228929 | 592,6731527 | 459,5274258 | 510,5814396 | 466,0323093 | 730,307911 | -0,512394719 | 0,152762371 | -3,354194595 | 0,000795964 | 0,011597738 | 1,426415929 |
| ST3GAL5 | 27,69780346 | 18,40839676 | 27,31212685 | 4,811805506 | 10,09941309 | 9,785455313 | 25,89106502 | -1,557068028 | 0,464504358 | -3,352106394 | 0,000801992 | 0,011670437 | 2,942552239 |
| MAML1 | 1154,652182 | 853,3606783 | 792,0516787 | 719,3649231 | 572,3000751 | 523,5218592 | 734,5025731 | -0,623508279 | 0,186044465 | -3,351393869 | 0,000804059 | 0,011685373 | 1,540617031 |
| PTRH1 | 154,0690318 | 126,2290063 | 137,9262406 | 70,97413121 | 75,18451967 | 91,73864356 | 95,2558922 | -0,811282835 | 0,242204224 | -3,349581687 | 0,000809337 | 0,011746887 | 1,75477108 |
| AP3D1 | 1055,978757 | 1437,169833 | 1605,953059 | 815,6010332 | 732,7685276 | 950,4123473 | 1108,682489 | -0,715220747 | 0,213565286 | -3,348956009 | 0,000811167 | 0,011755805 | 1,6417344 |
| ATP5D | 2825,175953 | 1698,832044 | 1816,256436 | 1099,497558 | 965,0550287 | 1473,934207 | 1548,433856 | -0,840919289 | 0,251183509 | -3,347828422 | 0,000814474 | 0,01177436 | 1,79119113 |
| URB2 | 527,9893785 | 356,3339658 | 331,8423413 | 246,6050322 | 228,9200301 | 160,2368308 | 278,199877 | -0,932818915 | 0,278690503 | -3,347149995 | 0,00081647 | 0,01177436 | 1,909002398 |
| SRF | 900,1786125 | 1001,942738 | 898,5689735 | 670,0439166 | 649,7289088 | 540,646406 | 778,6867257 | -0,590320518 | 0,176409728 | -3,346303663 | 0,000818967 | 0,011786809 | 1,5055812 |
| NRSN2 | 1540,690318 | 1179,452278 | 1048,785671 | 769,8888809 | 724,9134285 | 878,2446143 | 1031,243833 | -0,666453036 | 0,199372639 | -3,342750729 | 0,000829524 | 0,011886314 | 1,587166011 |
| UHRF1 | 2548,197918 | 1906,58395 | 1747,976119 | 1248,663529 | 1320,778801 | 807,3000633 | 1237,585148 | -0,876692458 | 0,262824286 | -3,335660005 | 0,000850972 | 0,012149922 | 1,836160865 |
| LONRF1 | 48,47115606 | 67,05915962 | 99,68926301 | 37,29149267 | 37,03118133 | 23,24045637 | 39,25396313 | -1,148291916 | 0,344345909 | -3,334704689 | 0,0008539 | 0,012158068 | 2,216513141 |
| HIST1H2AJ | 7620,358177 | 6899,204128 | 6326,854186 | 4331,827906 | 4470,673528 | 4799,765831 | 4471,564245 | -0,615862083 | 0,185036195 | -3,328333048 | 0,000873674 | 0,012376858 | 1,532473453 |
| TMEM189 | 5413,189464 | 2773,093483 | 2511,350064 | 1941,563521 | 1863,902794 | 2218,851992 | 4237,136258 | -0,827856207 | 0,248720375 | -3,328461557 | 0,000873271 | 0,012376858 | 1,775045747 |
| ZDHHC18 | 695,907312 | 503,6011399 | 467,0373692 | 405,3946138 | 353,4794582 | 303,3491147 | 471,8545944 | -0,646532605 | 0,194437627 | -3,325141408 | 0,000883737 | 0,012487926 | 1,565401361 |
| EXOC3 | 671,6717339 | 545,6774753 | 594,0387591 | 446,2949606 | 414,0759367 | 360,8386647 | 698,8814938 | -0,566824858 | 0,170495277 | -3,324578063 | 0,000885524 | 0,012497464 | 1,481259963 |
| COX7A2 | 34,62225433 | 30,2423661 | 31,40894588 | 10,82656239 | 14,58804113 | 9,785455313 | 21,67808943 | -1,441477908 | 0,433885021 | -3,322257832 | 0,000892921 | 0,012570275 | 2,71598951 |
| RGMB | 391,2314739 | 383,946561 | 364,6168935 | 297,12899 | 232,2865011 | 258,0913839 | 401,1843249 | -0,533587936 | 0,160678293 | -3,32084643 | 0,000897449 | 0,012618201 | 1,44752467 |
| EARS2 | 292,5580491 | 286,6450352 | 300,4333954 | 221,3430533 | 199,7439478 | 168,7991041 | 275,4532294 | -0,575987611 | 0,173464817 | -3,320486669 | 0,000898607 | 0,012618682 | 1,490697585 |
| TTYH3 | 1656,67487 | 1063,742356 | 1324,638152 | 881,7633589 | 791,1206921 | 885,5837058 | 852,3607512 | -0,659826996 | 0,198977464 | -3,316089085 | 0,000912867 | 0,012786971 | 1,579893156 |
| TCOF1 | 1258,518945 | 987,4789975 | 863,0632086 | 677,2616249 | 789,9985351 | 577,3418635 | 1001,306642 | -0,602611978 | 0,181779433 | -3,315072387 | 0,000916194 | 0,012801645 | 1,518463235 |
| CEP164 | 688,9828611 | 458,8950335 | 435,6284233 | 328,4057258 | 359,0902432 | 287,4477498 | 401,4566329 | -0,695814435 | 0,209981225 | -3,313698332 | 0,000920708 | 0,012837173 | 1,619798589 |
| DLG4 | 315,0625144 | 276,1259514 | 327,7455222 | 234,5755184 | 155,9798244 | 201,8250158 | 247,5785093 | -0,634080425 | 0,192013968 | -3,302261965 | 0,000959085 | 0,01325259 | 1,551948222 |
| TBL3 | 981,5409102 | 652,1831994 | 625,4477049 | 520,877946 | 481,4053573 | 450,1309444 | 637,6506119 | -0,634470244 | 0,19230532 | -3,299285965 | 0,000969311 | 0,013328325 | 1,552367618 |
| ALYREF | 4433,379667 | 2991,364473 | 2503,156426 | 2416,729315 | 1770,763762 | 1862,906055 | 2734,59939 | -0,713960783 | 0,216432066 | -3,298775433 | 0,000971076 | 0,013336263 | 1,640301234 |
| NAB1 | 372,189234 | 307,683203 | 356,4232554 | 198,4869771 | 246,8745422 | 240,9668371 | 447,8782311 | -0,591936961 | 0,179508293 | -3,297546597 | 0,000975335 | 0,013362088 | 1,507269047 |
| RXRA | 732,260679 | 571,975185 | 550,3393561 | 433,0624955 | 425,2975068 | 363,2850285 | 364,3635406 | -0,59964662 | 0,181874857 | -3,297028691 | 0,000977135 | 0,013370447 | 1,515345346 |
| CARKD | 183,4979479 | 206,4370208 | 208,9377704 | 113,0774294 | 105,4827589 | 138,2195563 | 113,5510136 | -0,750562363 | 0,227769499 | -3,295271612 | 0,000983266 | 0,013379283 | 1,682448522 |
| COX5B | 5343,944955 | 2815,169818 | 2862,310894 | 2357,784698 | 2013,149676 | 1847,00469 | 3095,50657 | -0,825286858 | 0,250481825 | -3,294797373 | 0,000984927 | 0,013379283 | 1,771887316 |
| FAM125A | 48,47115606 | 64,42938865 | 53,25864736 | 20,4501734 | 28,05392525 | 33,02591168 | 57,75782903 | -1,034754136 | 0,313910798 | -3,296331765 | 0,000979563 | 0,013379283 | 2,048764467 |
| HIST1H1B | 12209,53799 | 7131,938858 | 6852,612627 | 4533,923738 | 4851,084755 | 5841,916822 | 5060,081935 | -0,782421889 | 0,237385497 | -3,295997011 | 0,000980731 | 0,013379283 | 1,720015885 |

| | | | | | | | | | | | | | | |
|---|---|---|---|---|---|---|---|---|---|---|---|---|---|---|
| FICD | 218,1202023 | 106,5057241 | 107,8829011 | 55,33576331 | 88,6504038 | 74,61409676 | 192,2712066 | -0,971548031 | 0,295020923 | -3,293149588 | 0,000990717 | 0,013425477 | 1,960943586 |
| ZNF574 | 553,9560692 | 624,5706043 | 610,4260352 | 399,379857 | 378,1669124 | 451,3541263 | 410,503811 | -0,543402315 | 0,164993127 | -3,293484544 | 0,000989538 | 0,013425477 | 1,457405475 |
| ADAM8 | 81,36229767 | 89,41221282 | 69,64592348 | 42,10329817 | 21,32098319 | 42,81136699 | 47,75413904 | -1,18330557 | 0,359467975 | -3,291824731 | 0,000995396 | 0,013456416 | 2,270965155 |
| ZDBF2 | 141,9512427 | 84,15267089 | 140,6574533 | 72,17708258 | 58,35216452 | 51,37364039 | 68,2703492 | -1,00708679 | 0,306027161 | -3,290841194 | 0,000998883 | 0,013471128 | 2,009848546 |
| FAM195A | 86,55563582 | 98,6164112 | 68,28031713 | 60,14756882 | 33,6647103 | 36,69545742 | 75,68493956 | -0,960184994 | 0,291846832 | -3,290030554 | 0,001001765 | 0,013493799 | 1,945559353 |
| TBC1D9B | 4038,685967 | 2548,248065 | 2126,249076 | 2070,279319 | 1611,417466 | 1497,174663 | 2055,606291 | -0,749922357 | 0,22796624 | -3,289620241 | 0,001003227 | 0,013497306 | 1,681702322 |
| ZNF646 | 98,67342483 | 71,00381607 | 65,54910445 | 45,7121523 | 30,29823927 | 46,48091274 | 74,92522469 | -0,934629025 | 0,284275691 | -3,287755708 | 0,001009894 | 0,013554543 | 1,911399074 |
| POLR2C | 1045,592081 | 782,3568622 | 669,1471079 | 572,6048552 | 527,4137947 | 514,9595858 | 780,4990201 | -0,626626039 | 0,190817117 | -3,283908957 | 0,00102378 | 0,013691837 | 1,543950007 |
| RABIF | 1762,272745 | 1226,788155 | 1061,076128 | 888,9810671 | 856,2057987 | 821,9782463 | 825,2440254 | -0,656337035 | 0,199952049 | -3,282472164 | 0,001029011 | 0,013745439 | 1,576075924 |
| ACTN4 | 18043,38784 | 18759,47118 | 15774,11886 | 12611,74223 | 14191,91971 | 13080,70739 | 13224,31474 | -0,398569478 | 0,121466614 | -3,281308859 | 0,001033265 | 0,013769517 | 1,318200185 |
| PACS2 | 121,1778901 | 82,83778541 | 80,57077422 | 44,50920093 | 39,27549535 | 61,15909571 | 61,89737256 | -0,967177498 | 0,294879057 | -3,27991248 | 0,001038393 | 0,013788778 | 1,955012048 |
| PEF1 | 952,111994 | 1055,853043 | 1056,979309 | 777,1065891 | 673,294206 | 815,8623367 | 1333,98587 | -0,437031345 | 0,133357366 | -3,277144405 | 0,001048627 | 0,013875475 | 1,353815693 |
| GSTO1 | 1393,545737 | 1055,853043 | 740,1586377 | 629,1435698 | 525,1694807 | 725,3468751 | 651,5215081 | -0,76187539 | 0,232528336 | -3,276484073 | 0,001051082 | 0,0138916 | 1,695693461 |
| CLPP | 1028,280954 | 645,608772 | 533,95208 | 297,12899 | 333,280632 | 481,9336742 | 588,575934 | -0,987794711 | 0,301834942 | -3,272632073 | 0,001065511 | 0,01402962 | 1,983151248 |
| HIST1H3E | 434,5092918 | 307,683203 | 329,1111286 | 222,5460046 | 182,9115926 | 214,056835 | 183,7807874 | -0,787885541 | 0,240742247 | -3,272734851 | 0,001065123 | 0,01402962 | 1,726542133 |
| NOSIP | 1229,090029 | 698,2041913 | 592,6731527 | 531,7045084 | 430,9082919 | 477,0409465 | 581,1941748 | -0,805520514 | 0,246158576 | -3,272364212 | 0,001066521 | 0,01402962 | 1,747776263 |
| PIH1D2 | 64,0511705 | 61,79961769 | 64,1834981 | 32,47968716 | 28,05392525 | 30,57954785 | 61,83183249 | -1,061153352 | 0,32439111 | -3,27121589 | 0,001070861 | 0,014053803 | 2,086598972 |
| POLR3D | 1575,312572 | 732,3912139 | 609,0604288 | 479,9775992 | 559,956348 | 288,6709317 | 795,8425695 | -1,132723574 | 0,34625671 | -3,271340425 | 0,00107039 | 0,014053803 | 2,192723002 |
| PHLDA1 | 8910,037151 | 8558,589607 | 8682,525127 | 6603,000105 | 6756,507358 | 6651,663249 | 9670,938482 | -0,386011516 | 0,118014548 | -3,270880769 | 0,001072131 | 0,014054049 | 1,306775685 |
| CCDC159 | 55,39560692 | 44,70610641 | 39,60258394 | 20,4501734 | 22,4431402 | 17,1245468 | 34,21831633 | -1,207711189 | 0,369320212 | -3,270092322 | 0,001075124 | 0,014076855 | 2,309709146 |
| NRM | 46,74004334 | 39,44656448 | 54,62425371 | 20,4501734 | 16,83235515 | 18,34772871 | 31,51208056 | -1,340520245 | 0,410132538 | -3,268504988 | 0,001081173 | 0,014123133 | 2,532426232 |
| KCTD13 | 138,4890173 | 136,7480902 | 122,9045708 | 74,58298534 | 69,57373462 | 81,95318825 | 122,8197288 | -0,816162756 | 0,249839549 | -3,266747628 | 0,001087906 | 0,014194585 | 1,760716649 |
| RECQL4 | 673,4028467 | 289,2748062 | 308,6270334 | 174,4279496 | 206,4768899 | 253,1986562 | 205,0577814 | -0,999768812 | 0,306261269 | -3,264431098 | 0,001096841 | 0,014294566 | 1,999679531 |
| AURKA | 320,2558525 | 308,9980884 | 240,3467163 | 181,6456578 | 164,9570805 | 216,5031988 | 345,0071676 | -0,62598333 | 0,191833974 | -3,263151553 | 0,001101806 | 0,014342626 | 1,543262343 |
| CC2D1A | 358,3403323 | 248,5133562 | 273,1212685 | 212,9223936 | 194,1331627 | 151,6745574 | 218,8302706 | -0,649512811 | 0,199088173 | -3,262437951 | 0,001104584 | 0,014362143 | 1,568638387 |
| CEP104 | 531,4516039 | 544,3625898 | 532,5864736 | 429,4536414 | 354,6016152 | 351,0532094 | 590,3299597 | -0,503271052 | 0,15460109 | -3,255287861 | 0,001132775 | 0,014662625 | 1,417423675 |
| DYNLRB1 | 2655,526907 | 3313,511416 | 2936,053637 | 1720,220468 | 1536,232947 | 2089,194709 | 4166,261201 | -0,736567131 | 0,226467178 | -3,252423321 | 0,001144255 | 0,014724578 | 1,666206408 |
| PLCXD1 | 121,1778901 | 76,263358 | 65,54910445 | 48,11805506 | 44,8862804 | 45,25773082 | 71,49699006 | -0,912813411 | 0,280600052 | -3,253076412 | 0,001141628 | 0,014724578 | 1,882713415 |
| SPATA20 | 472,5937716 | 408,9293851 | 401,4882647 | 269,4611083 | 258,0961123 | 221,3959265 | 314,773746 | -0,775228183 | 0,238350076 | -3,252477183 | 0,001144038 | 0,014724578 | 1,711460722 |
| RAB35 | 3045,027268 | 2354,9599 | 1900,924029 | 1785,179843 | 1601,318053 | 1380,972381 | 2114,353551 | -0,614095151 | 0,188882095 | -3,251208913 | 0,001149154 | 0,014770703 | 1,530597715 |
| ADAMTS6 | 296,0202745 | 333,9809126 | 294,97097 | 230,9666643 | 232,2865011 | 198,1554701 | 980,6423389 | -0,484849129 | 0,149402945 | -3,245244782 | 0,001173497 | 0,014983976 | 1,39943951 |
| CHMP6 | 493,3671242 | 336,6106836 | 352,3264364 | 261,0404487 | 230,0421871 | 267,8768392 | 332,5147622 | -0,635655519 | 0,195963831 | -3,243738988 | 0,001179718 | 0,014983976 | 1,553643521 |
| NUP98 | 1841,90393 | 1781,669829 | 1672,86777 | 1366,552764 | 1174,89839 | 1354,062379 | 1341,181289 | -0,443135451 | 0,13661113 | -3,243772686 | 0,001179578 | 0,014983976 | 1,359555882 |
| SCP2 | 491,6360114 | 483,8778576 | 622,7164923 | 384,9444404 | 326,5476899 | 338,8213902 | 444,0387341 | -0,606752745 | 0,186980173 | -3,245011153 | 0,00117446 | 0,014983976 | 1,522827729 |
| SYVN1 | 278,7091473 | 226,160303 | 188,4536753 | 137,1364569 | 148,1247253 | 127,2109191 | 247,4802916 | -0,744270661 | 0,229298669 | -3,24585687 | 0,001170977 | 0,014983976 | 1,675127212 |
| ZGPAT | 219,851315 | 168,3053418 | 142,0230596 | 96,23611011 | 70,69589163 | 117,4254638 | 209,6771743 | -0,896386998 | 0,276358153 | -3,243569944 | 0,001180418 | 0,014983976 | 1,861398562 |
| DOK1 | 559,1494074 | 372,1125916 | 327,7455222 | 263,4463514 | 242,3859142 | 190,8163786 | 299,8532337 | -0,850512055 | 0,262249675 | -3,243138638 | 0,001182207 | 0,014989741 | 1,803140799 |
| C21orf119 | 24,23557803 | 59,16984672 | 55,98986005 | 24,05902753 | 14,58804113 | 12,23181914 | 36,8299308 | -1,46965764 | 0,453457725 | -3,241002541 | 0,001191101 | 0,015085492 | 2,769561624 |
| NFKBIL1 | 143,6823555 | 68,3740451 | 99,68926301 | 36,08854129 | 49,37490844 | 33,02591168 | 80,04562939 | -1,387723574 | 0,428246714 | -3,240476875 | 0,0011933 | 0,015096314 | 2,616654734 |
| CSNK1G2 | 1544,152543 | 1101,874035 | 969,5805033 | 884,1692616 | 747,3565687 | 776,7205155 | 1014,944189 | -0,584557106 | 0,180606841 | -3,2366277 | 0,001209511 | 0,015249883 | 1,499578562 |
| POLD4 | 555,6871819 | 512,8053383 | 494,349496 | 376,5237808 | 350,1129871 | 415,8818508 | 650,0617991 | -0,45105981 | 0,1394009 | -3,235702285 | 0,001213439 | 0,015282254 | 1,367044125 |
| CCDC107 | 723,6051154 | 349,7595384 | 337,3047666 | 244,1991294 | 283,9057235 | 211,6104711 | 254,4027384 | -0,927174332 | 0,286698302 | -3,23397218 | 0,001220813 | 0,015297138 | 1,901547968 |
| PYGB | 7395,313524 | 4842,723233 | 3973,914457 | 3684,640066 | 3373,203972 | 2941,752503 | 3707,332408 | -0,696729064 | 0,215492631 | -3,233192056 | 0,001224152 | 0,015297138 | 1,620825823 |
| ZNF576 | 228,5068786 | 255,0877836 | 236,2498973 | 157,5866303 | 172,8121796 | 135,7731925 | 213,0015395 | -0,627072631 | 0,193868353 | -3,234528074 | 0,001218439 | 0,015297138 | 1,544428016 |
| CLSPN | 579,92276 | 704,7786187 | 846,6759324 | 472,7598909 | 447,740647 | 529,6377688 | 688,7740624 | -0,558051206 | 0,17274163 | -3,230554242 | 0,001235505 | 0,015387648 | 1,472279117 |
| MAN2C1 | 43,27781791 | 74,94847251 | 87,39880593 | 30,07378441 | 34,78686731 | 29,35636594 | 43,22390211 | -1,135961718 | 0,35240739 | -3,223433309 | 0,001266638 | 0,015653917 | 2,19765012 |
| BCL7C | 881,1363726 | 550,9370173 | 506,6399531 | 393,3651001 | 406,2208376 | 382,8559391 | 457,8762234 | -0,710223448 | 0,220547866 | -3,220268955 | 0,001280704 | 0,015741167 | 1,636057493 |
| MCRS1 | 626,6628033 | 678,4809091 | 677,340746 | 507,6454808 | 446,61849 | 473,3714008 | 687,2034118 | -0,474930694 | 0,147440299 | -3,221172902 | 0,001276671 | 0,015741167 | 1,389851451 |
| DNAJC30 | 545,3005056 | 298,4790046 | 267,6588432 | 200,8928799 | 203,1104188 | 123,5413733 | 227,1855837 | -1,071426781 | 0,332851438 | -3,218933914 | 0,001286681 | 0,015788501 | 2,101510669 |
| PPP2R1A | 3145,431806 | 2841,467528 | 2438,972928 | 1853,748071 | 1767,397291 | 1209,726913 | 1874,87799 | -0,802290821 | 0,249252968 | -3,218781419 | 0,001287366 | 0,015788501 | 1,743867975 |

| | | | | | | | | | | | | | |
|---|---|---|---|---|---|---|---|---|---|---|---|---|---|
| ZNF611 | 121,1778901 | 110,4503805 | 84,66759324 | 61,3505202 | 54,98569349 | 58,71273188 | 91,78684411 | -0,84936377 | 0,263987266 | -3,217442202 | 0,001293391 | 0,015845096 | 1,801706196 |
| BCL7B | 242,3557803 | 310,3129739 | 372,8105315 | 194,878123 | 171,6900225 | 209,1641073 | 196,259411 | -0,689276433 | 0,214497535 | -3,213446865 | 0,001311521 | 0,016032234 | 1,612474597 |
| TRAF3 | 860,36302 | 724,501901 | 811,1701675 | 594,2579799 | 494,8712414 | 577,3418635 | 874,7633641 | -0,523246586 | 0,162888675 | -3,212295673 | 0,001316788 | 0,016044245 | 1,4371858 |
| PDXP | 450,0893063 | 218,2709901 | 219,8626212 | 163,6013872 | 130,1702132 | 152,8977393 | 253,037178 | -0,987265463 | 0,30759576 | -3,209619866 | 0,001329106 | 0,016159284 | 1,982423868 |
| TRIM65 | 283,9024855 | 259,0324401 | 187,0880689 | 158,7895817 | 155,9798244 | 152,8977393 | 185,3385264 | -0,638406942 | 0,19904292 | -3,207383318 | 0,001339484 | 0,016250283 | 1,556609365 |
| RNF123 | 126,3712283 | 143,3225176 | 124,2701772 | 69,77117983 | 70,69589163 | 42,81136699 | 138,6115225 | -1,103201629 | 0,344245895 | -3,204690728 | 0,001352077 | 0,016350086 | 2,148309167 |
| TSR3 | 2449,524494 | 915,160296 | 904,0313988 | 648,3907919 | 778,776965 | 491,7191295 | 964,1546982 | -1,152331138 | 0,360192071 | -3,199212952 | 0,001378033 | 0,016646051 | 2,222727575 |
| BRD1 | 462,2070953 | 306,3683175 | 327,7455222 | 193,6751716 | 233,4086581 | 178,5845595 | 207,5319555 | -0,852657794 | 0,266872146 | -3,195004824 | 0,001398285 | 0,016836572 | 1,80582463 |
| CCDC97 | 488,173786 | 539,1030479 | 436,9940296 | 316,376212 | 389,3884825 | 294,7868413 | 490,7737577 | -0,548572178 | 0,171697008 | -3,195001377 | 0,001398302 | 0,016836572 | 1,46263742 |
| RASSF1 | 57,12671964 | 55,22519027 | 36,87137125 | 19,24722202 | 24,68745422 | 13,45500106 | 33,81176168 | -1,369712522 | 0,429081544 | -3,192196313 | 0,001411953 | 0,016932899 | 2,584190671 |
| MAPK13 | 1930,190679 | 1138,690828 | 946,3651955 | 741,0180479 | 850,5950136 | 673,9732347 | 764,5657092 | -0,824174626 | 0,258572214 | -3,187406 | 0,001435551 | 0,017156363 | 1,770521823 |
| CEBPG | 133,2956792 | 93,35686927 | 128,3669962 | 48,11805506 | 60,59647854 | 84,39955207 | 74,48156721 | -0,875420709 | 0,274848979 | -3,185097191 | 0,001447054 | 0,01724659 | 1,834542986 |
| HAS2 | 83,09341038 | 90,72709831 | 117,4421455 | 66,1623257 | 57,23000751 | 47,70409465 | 166,8349667 | -0,772690705 | 0,242860239 | -3,181627048 | 0,001464503 | 0,017391357 | 1,708453173 |
| KRT79 | 29,42891618 | 15,77862579 | 17,75288245 | 4,811805506 | 5,61078505 | 7,339091485 | 22,69322765 | -1,806682338 | 0,56802714 | -3,180626789 | 0,001469568 | 0,017433082 | 3,498368681 |
| MGC2752 | 408,5426011 | 299,7938901 | 251,271567 | 185,254512 | 158,2241384 | 228,7350179 | 237,6597221 | -0,742967058 | 0,233632274 | -3,180070305 | 0,001472393 | 0,017448171 | 1,67361427 |
| BRF1 | 223,3135404 | 148,5820595 | 176,1632182 | 89,01840185 | 117,8264861 | 119,8718276 | 131,0013391 | -0,739494714 | 0,232663406 | -3,178388579 | 0,001480961 | 0,017531211 | 1,669590983 |
| RUVBL2 | 2719,578077 | 1823,746165 | 1212,658432 | 1169,268738 | 1068,293474 | 683,75869 | 1780,964475 | -0,977771376 | 0,307992388 | -3,174660846 | 0,001500117 | 0,017720628 | 1,969420767 |
| MARCKSL1 | 373,9203467 | 177,5095402 | 206,2065577 | 113,0774294 | 95,38334586 | 162,6831946 | 177,0875222 | -1,02584958 | 0,323423889 | -3,171842323 | 0,001514752 | 0,017874713 | 2,036158092 |
| FABP5 | 3848,263568 | 2813,854933 | 2731,212685 | 1892,242515 | 1780,863175 | 922,2791633 | 1293,26057 | -1,031185145 | 0,325477549 | -3,168222042 | 0,001533743 | 0,018038943 | 2,043702423 |
| NGEF | 88,28674853 | 86,78244186 | 68,28031713 | 36,08854129 | 53,86353648 | 42,81136699 | 55,26189951 | -0,867486546 | 0,273857003 | -3,167662456 | 0,001536698 | 0,018038943 | 1,82448153 |
| PITPNA-AS1 | 216,3890895 | 98,6164112 | 84,66759324 | 62,55347157 | 70,69589163 | 51,37364039 | 99,38719827 | -1,102469489 | 0,348009039 | -3,167933489 | 0,001535266 | 0,018038943 | 2,147219217 |
| C1orf122 | 339,2980924 | 209,0667918 | 249,9059607 | 156,3836789 | 147,0025683 | 183,4772871 | 321,2606137 | -0,70851319 | 0,223975441 | -3,163352143 | 0,001559636 | 0,018213041 | 1,634119162 |
| RAB3IL1 | 160,9934826 | 106,5057241 | 77,83956153 | 62,55347157 | 49,37490844 | 58,71273188 | 60,78063714 | -1,009386311 | 0,319228214 | -3,161958333 | 0,00156712 | 0,018281438 | 2,013054612 |
| PHB | 2132,730867 | 2257,658374 | 2307,874719 | 1365,349812 | 1329,756057 | 683,75869 | 1953,651641 | -0,987164203 | 0,312481415 | -3,159113331 | 0,001582499 | 0,01840757 | 1,982284731 |
| RAD54L | 308,1380635 | 366,8530497 | 211,6689831 | 159,9925331 | 162,7127665 | 101,5240989 | 274,7292611 | -1,062743495 | 0,336444573 | -3,158747624 | 0,001584486 | 0,01840757 | 2,088900095 |
| SDHB | 884,5985981 | 460,2099189 | 449,2844867 | 362,0883643 | 350,1129871 | 260,5377477 | 628,3995496 | -0,880145412 | 0,278620856 | -3,15893585 | 0,001583463 | 0,01840757 | 1,840560805 |
| LOC100506714 | 90,01786125 | 55,22519027 | 66,91471079 | 38,49444404 | 32,54255329 | 28,13318402 | 31,82028519 | -1,087344063 | 0,344363695 | -3,157545578 | 0,001591034 | 0,018464537 | 2,124825055 |
| ELL | 299,4824999 | 236,6793869 | 226,6906529 | 104,6567697 | 154,8576674 | 185,9236509 | 263,4349462 | -0,773253077 | 0,245062271 | -3,155333025 | 0,00160315 | 0,018585952 | 1,709119269 |
| MED29 | 1038,66763 | 777,0973203 | 772,9331899 | 639,9701322 | 591,3767443 | 523,5218592 | 959,2983901 | -0,558793392 | 0,177219698 | -3,153111081 | 0,001615403 | 0,018689434 | 1,473036718 |
| DMWD | 74,4378468 | 82,83778541 | 40,96819028 | 28,87083303 | 21,32098319 | 40,36500317 | 43,60642641 | -1,132507384 | 0,359239955 | -3,152509533 | 0,001618735 | 0,018696001 | 2,192394444 |
| MCM3 | 3268,340808 | 2368,108754 | 1969,204346 | 1649,246337 | 1730,36611 | 1669,643313 | 1841,126133 | -0,590157144 | 0,18752001 | -3,1471689 | 0,001648597 | 0,018974297 | 1,505410714 |
| SIRT7 | 347,953656 | 239,3091579 | 198,0129197 | 162,3984358 | 158,2241384 | 173,6918318 | 240,3934825 | -0,660844243 | 0,209999024 | -3,14689197 | 0,001650159 | 0,018974297 | 1,581007534 |
| MLX | 2658,989132 | 1688,31296 | 1360,143917 | 1073,032628 | 1061,560532 | 556,5477709 | 1625,392969 | -1,084039934 | 0,344746099 | -3,144458885 | 0,001663943 | 0,019093702 | 2,119964247 |
| PHF12 | 505,4849132 | 528,5839641 | 517,5648039 | 298,3319413 | 276,0506245 | 425,6673061 | 414,7692681 | -0,634962364 | 0,201924715 | -3,144550015 | 0,001663425 | 0,019093702 | 1,552897239 |
| FAU | 10137,39607 | 7497,477022 | 5982,721387 | 5384,410361 | 4864,550639 | 5013,822666 | 6336,717953 | -0,629627138 | 0,200282511 | -3,143695051 | 0,001668292 | 0,019104578 | 1,54716508 |
| CAMTA2 | 249,2802312 | 241,9389288 | 274,4868749 | 178,0368037 | 173,9343366 | 181,0309233 | 187,9835365 | -0,523453923 | 0,166645349 | -3,141125307 | 0,001683 | 0,019214339 | 1,43739236 |
| HIST1H2BD | 24794,72744 | 21975,68107 | 19137,60729 | 16301,1941 | 14084,19263 | 10153,63307 | 13093,30187 | -0,701105694 | 0,223202173 | -3,141123966 | 0,001683008 | 0,019214339 | 1,625750304 |
| GLUL | 503,7538005 | 499,6564834 | 561,2642068 | 374,1178781 | 331,036318 | 407,3195774 | 380,1273081 | -0,493386854 | 0,157346258 | -3,135675802 | 0,001714586 | 0,019515362 | 1,407745807 |
| THSD1 | 91,74897397 | 48,65076286 | 39,60258394 | 14,43541652 | 30,29823927 | 22,01727445 | 23,48650079 | -1,414985667 | 0,4515339 | -3,133730747 | 0,001725992 | 0,019625292 | 2,666570861 |
| MRTO4 | 2208,899826 | 1608,104945 | 1584,103357 | 1358,132104 | 1206,318786 | 1125,327361 | 1843,542666 | -0,548650839 | 0,175232144 | -3,130994271 | 0,001742156 | 0,019789055 | 1,462717171 |
| UBE2S | 5380,298322 | 4214,207972 | 3054,861389 | 2760,773409 | 2267,879317 | 1577,904669 | 3109,300541 | -0,936888916 | 0,299494035 | -3,128238982 | 0,001758571 | 0,019915111 | 1,914395507 |
| C11orf84 | 728,7984536 | 518,0648802 | 435,6284233 | 334,4204826 | 386,0220115 | 347,3836636 | 415,7925569 | -0,652494908 | 0,208689982 | -3,126623056 | 0,001768265 | 0,019929333 | 1,571884167 |
| EPHA2 | 3098,691762 | 3584,377826 | 2773,546482 | 2386,655531 | 2307,154813 | 2029,258796 | 3747,76597 | -0,492265208 | 0,157446714 | -3,126551164 | 0,001768697 | 0,019929333 | 1,406651758 |
| RFC4 | 690,7139738 | 607,477093 | 583,1139083 | 485,9923561 | 419,6867218 | 440,3454891 | 479,4997125 | -0,481602564 | 0,154009306 | -3,127100411 | 0,001765396 | 0,019929333 | 1,396293826 |
| SH3BP2 | 131,5645664 | 84,15267089 | 72,37713616 | 55,33576331 | 52,74137947 | 31,80272977 | 76,02115345 | -1,03067734 | 0,32957367 | -3,127304863 | 0,001764169 | 0,019929333 | 2,042983199 |
| ELOF1 | 1158,114407 | 909,900754 | 772,9331899 | 703,7265552 | 570,0557611 | 677,6427804 | 890,7904759 | -0,540244509 | 0,172842074 | -3,125653931 | 0,001774102 | 0,019970202 | 1,454218958 |
| AIDA | 1012,700939 | 1445,059146 | 2388,445493 | 798,7597139 | 646,3624378 | 1009,125079 | 1213,986016 | -0,982299306 | 0,314933869 | -3,119065315 | 0,001814257 | 0,020311944 | 1,975611547 |
| PPIA | 386,0381357 | 343,185111 | 464,3061565 | 191,2692688 | 188,5223777 | 300,9027509 | 296,3254278 | -0,811116891 | 0,260063816 | -3,118914823 | 0,001815184 | 0,020311944 | 1,754569252 |
| SIPA1 | 280,44026 | 188,028624 | 178,8944309 | 107,0626725 | 117,8264861 | 84,39955207 | 171,1487681 | -1,061622076 | 0,340384101 | -3,118894424 | 0,00181531 | 0,020311944 | 2,087277006 |
| TTC26 | 368,7270086 | 248,5133562 | 277,2180876 | 190,0663175 | 196,3774768 | 163,9063765 | 227,7765808 | -0,695769506 | 0,22305684 | -3,119247574 | 0,001813135 | 0,020311944 | 1,619748146 |

| | | | | | | | | | | | | | |
|---|---|---|---|---|---|---|---|---|---|---|---|---|---|
| WDR37 | 394,6936993 | 360,2786223 | 182,9912499 | 202,0958312 | 157,1019814 | 170,0222861 | 200,0429179 | -0,823369644 | 0,263988835 | -3,118956316 | 0,001814929 | 0,020311944 | 1,769534199 |
| TRAPPC5 | 2221,017615 | 996,6831959 | 1143,012509 | 638,7671809 | 720,4248005 | 962,6441664 | 1179,46577 | -0,908270939 | 0,291489158 | -3,115968177 | 0,001833421 | 0,020494176 | 1,876794823 |
| IQCE | 803,2363004 | 448,3759496 | 393,2946267 | 301,9407955 | 328,792004 | 232,4045637 | 358,4071444 | -0,927462944 | 0,2978828 | -3,113516271 | 0,001848723 | 0,020624187 | 1,901928411 |
| CIDECP | 126,3712283 | 88,09732734 | 96,95805033 | 50,52395781 | 49,37490844 | 72,16773293 | 66,55162723 | -0,850506152 | 0,273379449 | -3,111082988 | 0,001864025 | 0,020769729 | 1,803133422 |
| NET1 | 346,2225433 | 376,0572481 | 402,8538711 | 210,5164909 | 209,8433609 | 124,7645552 | 232,626907 | -1,045285003 | 0,336012158 | -3,110854704 | 0,001865467 | 0,020769729 | 2,063774015 |
| ARHGAP22 | 1450,672456 | 1007,20228 | 1039,226427 | 523,2838487 | 424,1753498 | 765,7118782 | 703,489826 | -1,029279141 | 0,331053584 | -3,109101341 | 0,001876573 | 0,020849695 | 2,041004186 |
| LEPREL4 | 450,0893063 | 395,7805303 | 299,067789 | 227,3578101 | 230,0421871 | 287,4477498 | 415,6195059 | -0,618039295 | 0,19886516 | -3,107830927 | 0,001884659 | 0,020872302 | 1,534787899 |
| SAYSD1 | 152,337919 | 120,9694644 | 120,1733582 | 73,38003396 | 65,08510658 | 88,06909782 | 81,70631851 | -0,793385664 | 0,255301035 | -3,107647659 | 0,001885828 | 0,020872302 | 1,733136956 |
| FBXW9 | 93,48008668 | 115,7099225 | 99,68926301 | 68,56822845 | 60,59647854 | 39,14182125 | 125,933122 | -0,877567746 | 0,282545335 | -3,105936071 | 0,001896777 | 0,020952207 | 1,837275209 |
| PRKAG1 | 387,7692485 | 456,2652625 | 514,8335912 | 332,0145799 | 317,5704339 | 329,0359349 | 630,2079484 | -0,477046111 | 0,153695458 | -3,103840006 | 0,001910266 | 0,02108048 | 1,391890878 |
| CPSF4 | 578,1916473 | 411,5591561 | 337,3047666 | 270,6640597 | 296,2494507 | 278,8854764 | 469,1513222 | -0,645546201 | 0,20812829 | -3,101674454 | 0,001924294 | 0,021172894 | 1,564331426 |
| CDCP1 | 1860,94617 | 2315,513335 | 2148,098777 | 1530,154151 | 1639,471392 | 1602,368308 | 2022,535212 | -0,407190169 | 0,131403464 | -3,098778049 | 0,001943205 | 0,021339168 | 1,326100549 |
| PPP1R37 | 813,6229767 | 406,2996142 | 353,6920427 | 279,0847193 | 283,9057235 | 304,5722966 | 364,3628588 | -0,855532489 | 0,276279358 | -3,096621098 | 0,001957399 | 0,021436137 | 1,809426479 |
| ARHGAP27 | 365,2647831 | 203,8072498 | 233,5186846 | 135,9335055 | 93,13903184 | 177,3613775 | 144,5796094 | -0,979469224 | 0,316767545 | -3,092075689 | 0,001987622 | 0,021678584 | 1,97173986 |
| ESM1 | 5,193338149 | 15,77862579 | 5,462425371 | 3,608854129 | 1,12215701 | 0 | 388,1158562 | -2,534060435 | 0,819450564 | -3,09238964 | 0,00198552 | 0,021678584 | 5,791995312 |
| PCK2 | 334,1047543 | 214,3263337 | 178,8944309 | 133,5276028 | 144,7582543 | 151,6745574 | 173,2521133 | -0,751587148 | 0,243102163 | -3,09165143 | 0,001990464 | 0,021688531 | 1,683644035 |
| KCNK5 | 12,11778901 | 11,83396934 | 9,559244399 | 3,608854129 | 3,36647103 | 1,223181914 | 67,81883938 | -2,012945978 | 0,651814246 | -3,088220289 | 0,002013591 | 0,021887442 | 4,036055402 |
| PLXNA4 | 519,3338149 | 468,0992318 | 409,6819028 | 275,4758652 | 319,8147479 | 308,2418424 | 196,9994885 | -0,627103617 | 0,203072548 | -3,088076766 | 0,002014564 | 0,021887442 | 1,544461188 |
| TAF6L | 458,7448698 | 340,55534 | 305,8958208 | 258,6345459 | 228,9200301 | 209,1641073 | 292,5792135 | -0,661884714 | 0,214327104 | -3,088198839 | 0,002013737 | 0,021887442 | 1,582148168 |
| SIRT6 | 396,424812 | 262,9770965 | 223,9594402 | 169,6161441 | 168,3235515 | 91,73864356 | 260,6553092 | -1,036180218 | 0,335788599 | -3,085811191 | 0,002029977 | 0,022012316 | 2,050790639 |
| AHCY | 1945,770693 | 2006,515247 | 1623,705941 | 1171,674641 | 1389,230378 | 1061,721901 | 1975,525442 | -0,621878996 | 0,201660089 | -3,083798081 | 0,002043763 | 0,022107493 | 1,538878144 |
| QTRT1 | 678,5961848 | 493,082056 | 426,0691789 | 354,870656 | 341,1357311 | 255,6450201 | 444,4325796 | -0,744934268 | 0,241845217 | -3,080210875 | 0,002068541 | 0,022301332 | 1,67589791 |
| SH3PXD2B | 744,378468 | 544,3625898 | 510,7367722 | 406,5975652 | 372,5561273 | 399,9804859 | 332,8285511 | -0,60738941 | 0,197472491 | -3,075817831 | 0,002099261 | 0,022545979 | 1,523499905 |
| BTBD11 | 90,01786125 | 84,15267089 | 57,35546639 | 26,46493028 | 33,6647103 | 58,71273188 | 276,4889192 | -0,960423802 | 0,312771818 | -3,070685231 | 0,002135682 | 0,022828019 | 1,945881427 |
| FBXO36 | 221,5824277 | 209,0667918 | 102,4204757 | 78,19183946 | 111,093544 | 101,5240989 | 240,9243378 | -0,869478675 | 0,283551674 | -3,066385259 | 0,002166639 | 0,023114936 | 1,827002584 |
| C11orf83 | 1007,507601 | 685,0553365 | 595,4043654 | 478,7746478 | 457,8400601 | 512,513222 | 482,1555698 | -0,656697344 | 0,214208528 | -3,065691875 | 0,00217167 | 0,023146622 | 1,576469594 |
| SUFU | 69,24450865 | 52,59541931 | 64,1834981 | 26,46493028 | 25,80961123 | 29,35636594 | 46,95681432 | -1,185228487 | 0,387089228 | -3,061899946 | 0,00219937 | 0,023375324 | 2,273994061 |
| LTBP3 | 2375,086647 | 1211,00953 | 1418,86499 | 762,6711726 | 949,3448305 | 1130,220089 | 1739,536358 | -0,815415126 | 0,266369712 | -3,06121563 | 0,002204403 | 0,023406674 | 1,759804452 |
| METRN | 31,16002889 | 53,91030479 | 77,83956153 | 25,2619789 | 28,05392525 | 19,57091063 | 32,99712477 | -1,171415705 | 0,383079373 | -3,057892922 | 0,002228992 | 0,02362311 | 2,252326075 |
| YDJC | 508,9471386 | 351,0744239 | 335,9391603 | 193,6751716 | 176,1786506 | 292,3404775 | 227,8849615 | -0,851382914 | 0,278407297 | -3,05804813 | 0,002227838 | 0,02362311 | 1,804229564 |
| ADCK2 | 207,733526 | 202,4923643 | 162,5071548 | 129,9187486 | 103,2384449 | 97,85455313 | 113,8684838 | -0,789700776 | 0,258446073 | -3,055572739 | 0,002246311 | 0,023703561 | 1,728715879 |
| CDK19 | 645,7050432 | 452,3206061 | 365,9824998 | 291,1142331 | 322,0590619 | 326,5895711 | 383,0369511 | -0,635609932 | 0,208004149 | -3,055756022 | 0,002244938 | 0,023703561 | 1,553594429 |
| DOHH | 424,1226155 | 264,291982 | 247,174748 | 182,8486092 | 177,3008076 | 122,3181914 | 235,9224116 | -0,951770469 | 0,311460157 | -3,055833785 | 0,002244356 | 0,023703561 | 1,934244899 |
| PDPN | 1010,969826 | 1017,721364 | 970,9461096 | 678,4645763 | 705,8367593 | 649,5095964 | 1937,106608 | -0,560418871 | 0,183435956 | -3,055120076 | 0,002249704 | 0,023708422 | 1,474697318 |
| DOLPP1 | 626,6628033 | 378,687019 | 260,8308114 | 252,619789 | 231,1643441 | 174,9150137 | 344,1467022 | -0,939442876 | 0,30766645 | -3,053445947 | 0,002262295 | 0,023818762 | 1,917787505 |
| RPL13 | 3794,599074 | 3291,158363 | 3842,816248 | 2676,566812 | 2589,938379 | 2743,597033 | 3464,511311 | -0,44807593 | 0,14676804 | -3,052953003 | 0,002266014 | 0,023835584 | 1,364219633 |
| FAM219A | 51,93338149 | 53,91030479 | 60,08667908 | 28,87083303 | 29,17608226 | 23,24045637 | 28,98247299 | -1,030061669 | 0,337914222 | -3,048293331 | 0,002301452 | 0,024095533 | 2,042111541 |
| CSTF2 | 1350,267919 | 788,9312896 | 626,8133113 | 576,2137093 | 491,5047704 | 566,3332262 | 1198,778687 | -0,757442619 | 0,2487225 | -3,04533212 | 0,002324235 | 0,024311411 | 1,690491332 |
| SGK2 | 1258,518945 | 1300,421742 | 1257,723442 | 934,6932194 | 939,2454174 | 697,2136911 | 533,5016675 | -0,569718114 | 0,187150423 | -3,044172198 | 0,002333215 | 0,024359984 | 1,48423354 |
| PKIG | 206,0024132 | 95,98664024 | 88,76441227 | 45,7121523 | 87,52824679 | 63,60545953 | 204,6650709 | -0,974780458 | 0,320317671 | -3,04316791 | 0,002341017 | 0,024418739 | 1,965342099 |
| AP5Z1 | 373,9203467 | 232,7347304 | 234,8842909 | 166,0072899 | 168,3235515 | 155,3441031 | 207,4149282 | -0,776149842 | 0,255163787 | -3,041771132 | 0,002351906 | 0,024509571 | 1,712554431 |
| SNAP29 | 1126,954378 | 950,662204 | 783,8580407 | 704,9295066 | 724,9134285 | 578,5650454 | 652,5784865 | -0,508714741 | 0,167453667 | -3,037943269 | 0,002381988 | 0,024777086 | 1,422782112 |
| LBX2-AS1 | 90,01786125 | 85,46755638 | 58,72107273 | 52,92986056 | 29,17608226 | 20,79409254 | 66,13425406 | -1,184027004 | 0,389861168 | -3,037047802 | 0,002389076 | 0,024804876 | 2,272101057 |
| PLEKHO2 | 342,7603178 | 320,8320578 | 293,6053637 | 221,3430533 | 209,8433609 | 234,8509275 | 381,6903871 | -0,521886038 | 0,171940724 | -3,035267184 | 0,002403227 | 0,024895281 | 1,435831087 |
| TCTA | 223,3135404 | 281,3854933 | 240,3467163 | 168,4131927 | 180,6672786 | 135,7731925 | 248,274356 | -0,621366702 | 0,204725844 | -3,035116093 | 0,002404431 | 0,024895281 | 1,538331793 |
| TMEM98 | 422,3915028 | 374,7423626 | 350,9608301 | 251,4168377 | 191,8888487 | 114,9790999 | 471,2071503 | -1,039274373 | 0,342376893 | -3,035468791 | 0,002401621 | 0,024895281 | 2,055193699 |
| VSIG10 | 325,4491907 | 168,3053418 | 233,5186846 | 178,0368037 | 93,13903184 | 132,1036467 | 195,1849191 | -0,847177238 | 0,279334936 | -3,032836671 | 0,002422667 | 0,024991955 | 1,798977619 |
| NUDC | 6181,80351 | 5104,385444 | 3490,489812 | 2855,806568 | 3043,289811 | 2333,831092 | 4898,054132 | -0,843647458 | 0,278243045 | -3,032052282 | 0,002428971 | 0,025034003 | 1,794581518 |
| HIST1H2AB | 1961,350708 | 2506,17173 | 2070,259215 | 1560,227935 | 1519,400592 | 1568,119214 | 1415,804604 | -0,492809197 | 0,162724512 | -3,028487788 | 0,00245781 | 0,025284834 | 1,407182257 |
| GOT1 | 581,6538727 | 528,5839641 | 512,1023785 | 412,6123221 | 397,2435816 | 404,8732136 | 486,6741365 | -0,415712144 | 0,137348811 | -3,026689051 | 0,002472482 | 0,02536608 | 1,333956983 |

| | | | | | | | | | | | | | |
|---|---|---|---|---|---|---|---|---|---|---|---|---|---|
| RPL23AP53 | 62,32005779 | 42,07633545 | 46,43061565 | 25,2619789 | 19,07666917 | 29,35636594 | 34,44256342 | -1,025751377 | 0,33885087 | -3,027146953 | 0,002468739 | 0,02536608 | 2,036019498 |
| C19orf6 | 1149,458844 | 1225,47327 | 869,8912403 | 771,0918323 | 692,3708752 | 818,3087006 | 1032,95992 | -0,507907516 | 0,167895367 | -3,025143123 | 0,002485155 | 0,02544044 | 1,421986252 |
| R3HDM2 | 160,9934826 | 228,790074 | 259,4652051 | 147,9630193 | 132,4145272 | 80,73000633 | 265,4829069 | -0,849153239 | 0,280684851 | -3,025290591 | 0,002483943 | 0,02544044 | 1,801443294 |
| SPSB2 | 51,93338149 | 57,85496124 | 50,52743468 | 34,88558992 | 17,95451216 | 29,35636594 | 42,96678386 | -0,972082051 | 0,321351921 | -3,024976633 | 0,002486523 | 0,02544044 | 1,961669572 |
| FAM64A | 231,969104 | 290,5896917 | 279,9493002 | 198,4869771 | 184,0337497 | 159,0136488 | 242,9921414 | -0,570759665 | 0,188818578 | -3,022793994 | 0,002504527 | 0,025601323 | 1,485305466 |
| MED30 | 124,6401156 | 237,9942724 | 217,1314085 | 101,0479156 | 111,093544 | 135,7731925 | 165,137065 | -0,745449473 | 0,24674107 | -3,021181157 | 0,002517907 | 0,025668025 | 1,676496502 |
| TMBIM1 | 2124,075303 | 1162,358767 | 1137,550083 | 904,619435 | 989,7424829 | 863,5664314 | 940,128121 | -0,680117362 | 0,225114214 | -3,021210205 | 0,002517665 | 0,025668025 | 1,602270093 |
| IER3 | 3195,634074 | 2247,13929 | 1902,289635 | 1287,157973 | 1392,596849 | 1849,451054 | 2171,320842 | -0,697013569 | 0,230859222 | -3,01921475 | 0,002534308 | 0,025765085 | 1,621145487 |
| KRT15 | 1121,76104 | 671,9064817 | 603,5980035 | 430,6565927 | 516,1922246 | 315,5809338 | 343,7305628 | -0,923394581 | 0,305819284 | -3,01941254 | 0,002532654 | 0,025765085 | 1,896572577 |
| HAX1 | 1161,576633 | 1124,227088 | 1177,152667 | 842,0659635 | 903,3363931 | 768,1582421 | 921,4602414 | -0,461911107 | 0,153081649 | -3,017416592 | 0,002549392 | 0,025869056 | 1,377365176 |
| ZNF609 | 723,6051154 | 802,0801445 | 975,0429287 | 655,6085001 | 563,3228191 | 551,6550433 | 826,4390174 | -0,499562581 | 0,165573077 | -3,017172784 | 0,002551443 | 0,025869056 | 1,413784843 |
| ARPC5L | 796,3118495 | 707,4083897 | 518,9304102 | 368,1031212 | 430,9082919 | 514,9595858 | 647,1210141 | -0,620999966 | 0,206101573 | -3,013077273 | 0,002586131 | 0,026173505 | 1,537940795 |
| HOMER3 | 1249,863381 | 1009,832051 | 841,2135071 | 773,497735 | 756,3338248 | 636,0545953 | 675,2737141 | -0,515828457 | 0,17122488 | -3,012578887 | 0,002590381 | 0,026183604 | 1,429814965 |
| SLC16A14 | 96,94231212 | 51,28053383 | 38,23697759 | 25,2619789 | 29,17608226 | 22,01727445 | 27,01724238 | -1,270164888 | 0,421806324 | -3,011251408 | 0,002601733 | 0,02623687 | 2,411891299 |
| EIF2D | 247,5491184 | 172,2499982 | 148,8510913 | 117,8892349 | 114,460015 | 125,9877372 | 162,7207574 | -0,657997415 | 0,218537094 | -3,010918666 | 0,002604586 | 0,026242081 | 1,577890854 |
| SETBP1 | 77,90007224 | 113,0801515 | 77,83956153 | 50,52395781 | 49,37490844 | 31,80272977 | 76,86372653 | -1,031533112 | 0,342763678 | -3,009458637 | 0,002617137 | 0,026344909 | 2,044195407 |
| FYN | 117,7156647 | 93,35686927 | 105,1516884 | 70,97413121 | 68,45157761 | 51,37364039 | 138,3446143 | -0,721627804 | 0,239873679 | -3,008365934 | 0,002626567 | 0,026416159 | 1,649041612 |
| GNB1 | 6701,137325 | 5054,419796 | 4309,853617 | 2735,51143 | 2862,622533 | 4243,21806 | 5547,45645 | -0,706876284 | 0,235769927 | -2,998161359 | 0,002716138 | 0,027219533 | 1,632266113 |
| DDX21 | 2113,688627 | 2039,387384 | 2768,084057 | 1835,7038 | 1564,286872 | 1536,316484 | 2549,420554 | -0,487891235 | 0,162788573 | -2,997085276 | 0,002725745 | 0,027281039 | 1,40239352 |
| ARL8A | 737,4540172 | 510,1755673 | 491,6182834 | 394,5680515 | 366,9453423 | 294,7868413 | 354,6383808 | -0,717090717 | 0,23936481 | -2,995806769 | 0,002737198 | 0,027357371 | 1,643863738 |
| CCM2 | 637,0494796 | 448,3759496 | 539,4145053 | 268,2581569 | 249,1188562 | 368,1777562 | 309,1625859 | -0,875042123 | 0,292278236 | -2,993866852 | 0,002754661 | 0,027490169 | 1,834061636 |
| RPPH1 | 93589,14678 | 48068,26859 | 39471,48573 | 39264,33293 | 32172,24148 | 23937,67006 | 42519,06773 | -0,925319351 | 0,309268143 | -2,991964646 | 0,002771884 | 0,027630293 | 1,899104576 |
| TOLLIP | 633,5872542 | 686,370222 | 462,9405502 | 419,8300304 | 338,891417 | 340,0445721 | 433,700392 | -0,698158543 | 0,23340276 | -2,991218026 | 0,00277867 | 0,027648877 | 1,622432597 |
| UNG | 1090,601011 | 923,0496089 | 822,0950183 | 631,5494726 | 735,0128416 | 711,891874 | 931,466088 | -0,446213162 | 0,149161719 | -2,991472377 | 0,002776357 | 0,027648877 | 1,362459327 |
| SCO2 | 136,7579046 | 113,0801515 | 155,6791231 | 63,75642295 | 71,81804864 | 72,16773293 | 57,74807569 | -0,964143882 | 0,322430703 | -2,990235956 | 0,00278762 | 0,027713385 | 1,95090548 |
| SF3A2 | 1246,401156 | 1525,26716 | 1675,598982 | 1106,715266 | 1061,560532 | 864,7896133 | 1609,412629 | -0,552792129 | 0,185036917 | -2,987469407 | 0,002812974 | 0,027939882 | 1,466921967 |
| NOTCH1 | 141,9512427 | 151,2118305 | 132,4638152 | 89,01840185 | 87,52824679 | 92,96182547 | 111,5806902 | -0,659997314 | 0,221503271 | -2,979627839 | 0,002885988 | 0,028490116 | 1,580079682 |
| ARHGEF2 | 559,1494074 | 412,8740416 | 344,1327983 | 246,6050322 | 243,5080712 | 122,3181914 | 235,8602113 | -1,101992795 | 0,370031662 | -2,978104065 | 0,002900375 | 0,028490366 | 2,146509854 |
| MXD3 | 155,8001445 | 132,8034338 | 169,3351865 | 91,4243046 | 97,62765988 | 57,48954996 | 94,5216114 | -0,890741533 | 0,29910645 | -2,978008439 | 0,00290128 | 0,028490366 | 1,854128885 |
| TK1 | 3086,573973 | 2398,35112 | 2182,238936 | 1809,23887 | 1618,150409 | 1851,897418 | 2062,095396 | -0,537804627 | 0,180562708 | -2,978492256 | 0,002896703 | 0,028490366 | 1,451761666 |
| CDC45 | 678,5961848 | 411,5591561 | 348,2296174 | 281,4906221 | 324,3033759 | 280,1086583 | 379,8824502 | -0,694770858 | 0,233590579 | -2,974310275 | 0,00293648 | 0,028810842 | 1,618627327 |
| TLN1 | 3439,720967 | 2883,543864 | 2688,878889 | 1834,500849 | 2193,816955 | 1489,835571 | 1646,452146 | -0,707344423 | 0,23794807 | -2,97268401 | 0,002952082 | 0,028913417 | 1,632795853 |
| NIT1 | 211,1957514 | 184,0839676 | 211,6689831 | 151,5718734 | 120,0708001 | 99,07773504 | 142,1549882 | -0,709873483 | 0,238908664 | -2,971317452 | 0,002965251 | 0,029017099 | 1,635660672 |
| PNP | 1649,750419 | 1686,998074 | 1115,700382 | 1021,305719 | 813,5638323 | 1039,704627 | 1078,026448 | -0,631128065 | 0,212478258 | -2,970318331 | 0,002974913 | 0,029086312 | 1,548775531 |
| DPCD | 477,7871097 | 411,5591561 | 435,6284233 | 360,8854129 | 304,1045497 | 297,2332051 | 454,4559579 | -0,459659929 | 0,15478121 | -2,969739866 | 0,00298052 | 0,029115795 | 1,375217614 |
| WHSC1 | 3578,209985 | 3118,908365 | 2436,241715 | 2058,249805 | 2221,87088 | 2281,23427 | 2211,109113 | -0,476613557 | 0,160611819 | -2,967487457 | 0,003002445 | 0,029304492 | 1,391473619 |
| RGP1 | 505,4849132 | 541,7328189 | 527,1240483 | 431,8595441 | 372,5561273 | 385,302303 | 436,9361229 | -0,40553507 | 0,136690793 | -2,966806045 | 0,003009107 | 0,029344019 | 1,324580083 |
| FLCN | 547,0316184 | 433,9122093 | 404,2194774 | 315,1732606 | 302,9823927 | 322,9200253 | 308,0386084 | -0,554953455 | 0,187083849 | -2,966335463 | 0,003013716 | 0,029363471 | 1,469121235 |
| PIAS4 | 1199,661112 | 552,2519027 | 517,5648039 | 436,6713496 | 398,3657386 | 457,4700359 | 586,0924646 | -0,809624835 | 0,273020636 | -2,965434582 | 0,003022556 | 0,029424087 | 1,752755589 |
| HIST1H2BN | 4447,228568 | 4414,070565 | 3160,013077 | 2861,821324 | 2918,730383 | 2540,548836 | 2788,029485 | -0,530521912 | 0,178971354 | -2,96428394 | 0,003033882 | 0,029466439 | 1,444451648 |
| FBXO8 | 271,7846965 | 272,1812949 | 288,1429383 | 175,630901 | 184,0337497 | 155,3441031 | 208,9289854 | -0,6919165 | 0,233612193 | -2,961816724 | 0,003058298 | 0,029618031 | 1,615428055 |
| AEN | 1519,916965 | 1122,912202 | 1033,764001 | 974,3906149 | 765,3110809 | 768,1582421 | 807,5778086 | -0,550583889 | 0,186138635 | -2,957923756 | 0,003097187 | 0,029775298 | 1,464678362 |
| MPP4 | 12,11778901 | 17,09351128 | 13,65606343 | 4,811805506 | 2,24431402 | 6,115909571 | 24,48141458 | -1,731459843 | 0,585295565 | -2,958265782 | 0,003093752 | 0,029775298 | 3,320636589 |
| RPS2 | 32891,14161 | 34286,95385 | 28937,1984 | 24102,33378 | 21415,24438 | 27034,76667 | 25151,91604 | -0,405754011 | 0,137120837 | -2,959098131 | 0,003085408 | 0,029775298 | 1,324781114 |
| HEMK1 | 126,3712283 | 88,09732734 | 62,81789176 | 51,72690918 | 56,1078505 | 41,58818508 | 74,59162494 | -0,878360744 | 0,297511453 | -2,952359428 | 0,003153556 | 0,030232487 | 1,83828537 |
| MSL3 | 219,851315 | 273,4961804 | 296,3365764 | 191,2692688 | 191,8888487 | 168,7991041 | 214,2911731 | -0,521133762 | 0,176615509 | -2,950668172 | 0,003170874 | 0,030335717 | 1,435082585 |
| TMEM86A | 60,58894507 | 43,39122093 | 49,16182834 | 22,85607615 | 29,17608226 | 18,34772871 | 44,03015283 | -1,109528496 | 0,376051455 | -2,950469893 | 0,00317291 | 0,030335717 | 2,157751158 |
| ARRB2 | 853,4385692 | 729,7614429 | 665,0502889 | 552,1546818 | 568,9336041 | 514,9595858 | 601,9003298 | -0,456424726 | 0,154794394 | -2,948586927 | 0,003192303 | 0,030495185 | 1,372137183 |
| SLC12A4 | 432,7781791 | 298,4790046 | 300,4333954 | 215,3282964 | 203,1104188 | 209,1641073 | 204,0438858 | -0,713834659 | 0,242358216 | -2,945370166 | 0,003225685 | 0,030735661 | 1,64015784 |
| GORASP1 | 290,8269363 | 369,4828206 | 309,9926398 | 220,1401019 | 236,7751291 | 240,9668371 | 295,4953495 | -0,478799448 | 0,162586142 | -2,944897032 | 0,003230622 | 0,030756612 | 1,393583501 |

| | | | | | | | | | | | | | |
|---|---|---|---|---|---|---|---|---|---|---|---|---|---|
| GADD45GIP1 | 5788,840923 | 3892,061029 | 2714,825409 | 2493,718203 | 2348,674622 | 2753,382489 | 3377,217762 | -0,706188555 | 0,23986163 | -2,944149735 | 0,003238433 | 0,030804872 | 1,631488202 |
| SAMD10 | 174,8423844 | 97,30152572 | 101,0548694 | 75,78593671 | 42,64196638 | 73,39091485 | 87,31764169 | -0,954627634 | 0,324528122 | -2,941586781 | 0,003265353 | 0,03103467 | 1,938079342 |
| BAX | 3481,267673 | 2173,505703 | 1644,190037 | 1456,774117 | 1472,269997 | 1584,020579 | 1607,373783 | -0,692800389 | 0,235627173 | -2,940239785 | 0,003279583 | 0,031117263 | 1,616418075 |
| BLOC1S3 | 83,09341038 | 59,16984672 | 36,87137125 | 19,24722202 | 23,56529721 | 39,14182125 | 39,45125259 | -1,119276688 | 0,380921728 | -2,938337739 | 0,003299773 | 0,031256032 | 2,172380304 |
| HIST1H3C | 30824,19303 | 27957,09513 | 19481,74008 | 17474,07169 | 17778,33351 | 18969,10512 | 17369,62605 | -0,529418856 | 0,180196423 | -2,938009792 | 0,003303266 | 0,031262754 | 1,443347671 |
| MRPL52 | 789,3873987 | 740,2805268 | 602,2323971 | 585,8373203 | 495,9933985 | 375,5168476 | 522,5991458 | -0,547514362 | 0,186484382 | -2,935979716 | 0,003324961 | 0,031408602 | 1,461565376 |
| SNORD17 | 467,4004334 | 402,3549577 | 365,9824998 | 298,3319413 | 196,3774768 | 278,8854764 | 435,2103722 | -0,675184001 | 0,229984259 | -2,935783542 | 0,003327064 | 0,031408602 | 1,596800415 |
| OSCP1 | 62,32005779 | 55,22519027 | 47,79622199 | 31,27673579 | 25,80961123 | 31,80272977 | 69,078271 | -0,891390747 | 0,303767997 | -2,934445879 | 0,003341438 | 0,031464976 | 1,854963433 |
| QRICH1 | 588,5783236 | 883,6030444 | 975,0429287 | 507,6454808 | 506,0928115 | 605,4750475 | 752,3519106 | -0,59815937 | 0,203831781 | -2,934573639 | 0,003340063 | 0,031464976 | 1,513784007 |
| C2CD2L | 107,3289884 | 95,98664024 | 76,47395519 | 55,33576331 | 61,71863555 | 34,2490936 | 74,87340783 | -0,879047913 | 0,299598832 | -2,934083248 | 0,003345345 | 0,031475379 | 1,839161171 |
| CYSTM1 | 366,9958959 | 326,0915997 | 292,2397573 | 267,0552056 | 198,6217908 | 226,2886541 | 276,0376552 | -0,508024243 | 0,173238191 | -2,932518742 | 0,003362246 | 0,031555117 | 1,422101308 |
| NMRAL1 | 321,9869652 | 244,5686998 | 232,1530783 | 164,8043386 | 193,0110057 | 174,9150137 | 222,5301579 | -0,578340386 | 0,197358724 | -2,930401933 | 0,003385238 | 0,031744379 | 1,493130627 |
| FDX1L | 867,2874709 | 742,9102977 | 506,6399531 | 413,8152735 | 381,5333834 | 278,8854764 | 458,3112301 | -0,977747902 | 0,333703085 | -2,929993591 | 0,00338969 | 0,031759614 | 1,969388722 |
| FOXA1 | 65,78228322 | 63,11450317 | 47,79622199 | 20,4501734 | 22,4431402 | 34,2490936 | 21,55179705 | -1,195255943 | 0,40874741 | -2,924192089 | 0,003453516 | 0,032250037 | 2,28985452 |
| CSRP1 | 2406,246676 | 2817,799589 | 2455,360204 | 1999,305188 | 1953,675355 | 1613,376945 | 1733,654269 | -0,464442055 | 0,158869557 | -2,923417578 | 0,003462119 | 0,032276715 | 1,379783636 |
| STOML2 | 1855,752832 | 1409,557237 | 1439,349085 | 1012,885059 | 956,0777726 | 596,9127741 | 1074,41023 | -0,874158777 | 0,299012019 | -2,923490429 | 0,003461309 | 0,032276715 | 1,832939004 |
| CCDC34 | 370,4581213 | 269,551524 | 472,4997946 | 218,9371505 | 223,309245 | 266,6536573 | 347,0529128 | -0,650051759 | 0,222581381 | -2,920512741 | 0,003494559 | 0,032471362 | 1,569224493 |
| SPICE1 | 145,4134682 | 164,3606853 | 140,6574533 | 98,64201286 | 103,2384449 | 75,83727868 | 156,7949304 | -0,697415977 | 0,238788118 | -2,920647736 | 0,003493045 | 0,032471362 | 1,621597732 |
| TMEM115 | 884,5985981 | 725,8167865 | 618,6196732 | 498,0218698 | 577,9108602 | 497,8350391 | 690,0062578 | -0,499626895 | 0,171129078 | -2,919590874 | 0,003504912 | 0,032513774 | 1,41384787 |
| IL17RA | 810,1607513 | 570,6602995 | 555,8017815 | 454,7156203 | 436,5190769 | 382,8559391 | 307,9669566 | -0,601505565 | 0,20637988 | -2,914555265 | 0,003561957 | 0,032924868 | 1,517299161 |
| NHLRC2 | 185,2290606 | 198,5477079 | 322,2830969 | 167,2102413 | 127,9258991 | 102,7472808 | 163,8570488 | -0,829803333 | 0,284726568 | -2,914386739 | 0,003563881 | 0,032924868 | 1,777443046 |
| COMMD7 | 69,24450865 | 90,72709831 | 94,22683764 | 48,11805506 | 53,86353648 | 37,91863934 | 71,54271027 | -0,865546362 | 0,297143941 | -2,912885785 | 0,003581056 | 0,03305635 | 1,822029556 |
| CSGALNACT2 | 436,2404045 | 387,8912174 | 472,4997946 | 294,7230872 | 264,8290544 | 123,5413733 | 220,2253381 | -0,923835968 | 0,317527263 | -2,90946975 | 0,003620424 | 0,033255802 | 1,897152914 |
| PTPN18 | 469,1315461 | 347,1297674 | 337,3047666 | 241,7932267 | 253,6074843 | 260,5377477 | 264,9388052 | -0,60634301 | 0,208567897 | -2,90717324 | 0,003647112 | 0,033371801 | 1,522395297 |
| SLC12A7 | 136,7579046 | 165,6755708 | 147,485485 | 87,81545048 | 90,89471782 | 113,755918 | 136,7896314 | -0,626294791 | 0,215473625 | -2,906596073 | 0,003653847 | 0,033371801 | 1,543595551 |
| SLC35B2 | 659,5539449 | 627,2003753 | 598,1355781 | 446,2949606 | 391,6327965 | 533,3073146 | 590,8916522 | -0,459102124 | 0,157940476 | -2,906804747 | 0,00365141 | 0,033371801 | 1,374686002 |
| GTPBP4 | 1358,923482 | 1316,200368 | 1418,86499 | 1154,833321 | 984,1316978 | 877,0214324 | 1234,827132 | -0,440846044 | 0,151686626 | -2,906294746 | 0,003657368 | 0,033376822 | 1,357400119 |
| ZFPL1 | 495,0982369 | 416,818698 | 352,3264364 | 275,4758652 | 300,7380787 | 247,0827467 | 360,6402556 | -0,61598809 | 0,211993139 | -2,905698232 | 0,003664347 | 0,033413368 | 1,532607307 |
| SUMF1 | 225,0446531 | 266,921753 | 230,7874719 | 187,6604147 | 140,2696263 | 111,3095542 | 164,1189937 | -0,719744616 | 0,247988235 | -2,902333719 | 0,003703937 | 0,033637859 | 1,646890479 |
| CUL7 | 275,2469219 | 332,6660271 | 308,6270334 | 206,9076367 | 219,942774 | 185,9236509 | 261,4055097 | -0,582413271 | 0,200754809 | -2,901117404 | 0,003718345 | 0,033714198 | 1,497351853 |
| HIST1H4A | 4724,206603 | 3020,291954 | 2310,605932 | 2075,091124 | 1911,033388 | 1327,152377 | 1698,768248 | -0,919904961 | 0,317218804 | -2,899906781 | 0,003732737 | 0,033817389 | 1,891990652 |
| SEC24D | 2861,52932 | 1719,870211 | 1414,768171 | 1234,228112 | 1275,89252 | 1291,680101 | 1634,391279 | -0,656283306 | 0,226714533 | -2,894756227 | 0,003794531 | 0,034211685 | 1,576017228 |
| IL11 | 50,20226877 | 48,65076286 | 46,43061565 | 26,46493028 | 24,68745422 | 12,23181914 | 49,37175694 | -1,191800447 | 0,411803738 | -2,894098172 | 0,003802493 | 0,034255975 | 2,284376497 |
| MOB3A | 335,835867 | 466,7843464 | 589,94194 | 294,7230872 | 271,5619964 | 148,0050116 | 281,4880902 | -0,964005334 | 0,333166651 | -2,893462874 | 0,003810193 | 0,034270386 | 1,950718136 |
| PPP1R11 | 687,2517484 | 712,6679316 | 628,1789176 | 543,7340221 | 469,0616302 | 475,8177646 | 635,2940133 | -0,446229334 | 0,154213238 | -2,893586448 | 0,003808694 | 0,034270386 | 1,3624746 |
| CDK2AP1 | 2039,25078 | 2212,952267 | 2246,422434 | 1723,829322 | 1517,156278 | 1166,915546 | 1656,774826 | -0,560145292 | 0,193942412 | -2,888204223 | 0,003874482 | 0,034682019 | 1,474417697 |
| S100A6 | 957,3053321 | 987,4789975 | 1264,551473 | 773,497735 | 628,4079256 | 780,3900612 | 782,192 | -0,55731086 | 0,192988567 | -2,887792108 | 0,003879562 | 0,03469984 | 1,471523784 |
| FAM100A | 1514,723627 | 1161,043881 | 813,9013802 | 773,497735 | 815,8081463 | 629,9386858 | 1082,862483 | -0,651575601 | 0,225787851 | -2,885786802 | 0,003904365 | 0,034755659 | 1,570882857 |
| NEU1 | 1268,905621 | 930,9389218 | 820,7294119 | 650,7966946 | 717,0583294 | 644,6168687 | 910,4939315 | -0,584094908 | 0,202318369 | -2,887008784 | 0,003889234 | 0,034755659 | 1,499098217 |
| RPUSD1 | 545,3005056 | 495,711827 | 408,3162965 | 351,2618019 | 259,2182693 | 344,9372998 | 420,0720078 | -0,600524632 | 0,208058062 | -2,886331947 | 0,003897608 | 0,034755659 | 1,516267853 |
| ZBTB17 | 581,6538727 | 328,7213707 | 264,9276305 | 238,1843725 | 216,5763029 | 242,190019 | 411,1197755 | -0,749435652 | 0,259644465 | -2,886391786 | 0,003896867 | 0,034755659 | 1,681135082 |
| TECR | 2658,989132 | 3068,942717 | 2261,444103 | 1603,534185 | 1912,155545 | 1066,614629 | 2300,014429 | -0,801887184 | 0,277921404 | -2,885302007 | 0,003910383 | 0,034781668 | 1,743380144 |
| COX15 | 1750,154956 | 1051,908386 | 837,116688 | 784,3242974 | 751,8451967 | 725,3468751 | 1053,563617 | -0,684669571 | 0,237819604 | -2,878945044 | 0,003990079 | 0,035378487 | 1,607333802 |
| ZNF592 | 462,2070953 | 419,448469 | 383,7353823 | 307,9555524 | 265,9512114 | 298,456387 | 430,1715143 | -0,535462621 | 0,186182336 | -2,876011938 | 0,004027345 | 0,03562456 | 1,449406853 |
| C17orf58 | 214,6579768 | 178,8244256 | 185,7224626 | 115,4833321 | 123,4372711 | 152,8977393 | 205,5382959 | -0,560846751 | 0,195259563 | -2,872313877 | 0,00407478 | 0,035959216 | 1,475134754 |
| CHMP1A | 1558,001445 | 1112,393118 | 1114,334776 | 754,250513 | 797,8536342 | 461,1395816 | 888,885278 | -0,909970827 | 0,317118008 | -2,869502216 | 0,004111185 | 0,036166834 | 1,879007502 |
| TMEM208 | 718,4117773 | 444,4312932 | 334,573554 | 303,1437468 | 279,4170955 | 320,4736615 | 349,1558312 | -0,726372402 | 0,253133995 | -2,869517393 | 0,004110987 | 0,036166834 | 1,654473751 |
| NDEL1 | 249,2802312 | 358,9637368 | 281,3149066 | 188,8633661 | 187,4002207 | 223,8422903 | 233,7283496 | -0,572393822 | 0,199581289 | -2,867973372 | 0,004131103 | 0,036285232 | 1,486988843 |
| MAP4K4 | 410,2737138 | 640,3492301 | 685,534384 | 329,6086771 | 382,6555404 | 404,8732136 | 424,6479275 | -0,638670201 | 0,222773926 | -2,866898358 | 0,004145161 | 0,036380266 | 1,556893437 |
| ZNF673 | 292,5580491 | 194,6030514 | 178,8944309 | 115,4833321 | 140,2696263 | 89,29227973 | 173,2275809 | -0,94423765 | 0,329433263 | -2,866248663 | 0,004153678 | 0,03642656 | 1,924171845 |

| | | | | | | | | | | | | | |
|---|---|---|---|---|---|---|---|---|---|---|---|---|---|
| LRRC14 | 863,8252455 | 477,3034302 | 390,563414 | 352,4647533 | 299,6159217 | 357,1691189 | 320,8725995 | -0,77614773 | 0,270814625 | -2,865974208 | 0,004157281 | 0,036429716 | 1,712551924 |
| LHFP | 292,5580491 | 234,0496159 | 244,4435353 | 205,7046854 | 136,9031552 | 176,1381956 | 175,3220899 | -0,569822814 | 0,198961896 | -2,863979613 | 0,004183549 | 0,036517478 | 1,484341259 |
| GEMIN4 | 460,4759825 | 327,4064852 | 360,5200745 | 283,8965248 | 250,2410132 | 271,5463849 | 277,7283995 | -0,507255784 | 0,177209773 | -2,86245941 | 0,004203671 | 0,036557503 | 1,421344019 |
| HIST1H3H | 45,00893063 | 47,33587738 | 40,96819028 | 24,05902753 | 24,68745422 | 14,67818297 | 27,75305565 | -1,067563772 | 0,372960547 | -2,862404025 | 0,004204406 | 0,036557503 | 2,09589112 |
| NTAN1 | 1611,665939 | 1003,257623 | 807,0733485 | 707,3354093 | 721,5469575 | 763,2655144 | 727,2534848 | -0,640688708 | 0,223821979 | -2,862492378 | 0,004203234 | 0,036557503 | 1,559073246 |
| C9orf114 | 673,4028467 | 395,7805303 | 402,8538711 | 297,12899 | 332,158475 | 185,9236509 | 427,1928431 | -0,84962616 | 0,296912513 | -2,861537061 | 0,004215922 | 0,036629288 | 1,80203391 |
| PSMA7 | 10819,45448 | 5605,356813 | 5377,757777 | 4545,953251 | 4034,154451 | 4804,658559 | 8283,80389 | -0,703640337 | 0,246005626 | -2,86026116 | 0,004232923 | 0,036691862 | 1,628609064 |
| C19orf55 | 363,5336704 | 224,8454175 | 162,5071548 | 147,9630193 | 157,1019814 | 69,72136911 | 167,6424266 | -0,997447342 | 0,348957876 | -2,858360309 | 0,004258366 | 0,036874759 | 1,996464394 |
| TRABD | 1230,821141 | 746,8549542 | 678,7063523 | 629,1435698 | 564,4449761 | 506,3973124 | 546,1648797 | -0,641638501 | 0,224510016 | -2,85795045 | 0,00426387 | 0,036874759 | 1,560099994 |
| VARS2 | 230,2379913 | 169,6202273 | 178,8944309 | 122,7010404 | 127,9258991 | 134,5500106 | 157,8615522 | -0,581079065 | 0,20331796 | -2,857981976 | 0,004263446 | 0,036874759 | 1,495967741 |
| C7orf26 | 1185,812211 | 808,6545719 | 577,6514829 | 543,7340221 | 500,4820265 | 462,3627635 | 571,9351426 | -0,77012057 | 0,269662377 | -2,855869544 | 0,004291914 | 0,036974974 | 1,705412304 |
| ZNF32 | 410,2737138 | 261,6622111 | 319,5518842 | 224,9519074 | 251,3631703 | 163,9063765 | 219,7252345 | -0,62560397 | 0,219111415 | -2,855186569 | 0,004301155 | 0,036997841 | 1,542856592 |
| NCKIPSD | 219,851315 | 228,790074 | 204,8409514 | 145,5571165 | 136,9031552 | 110,0863723 | 166,0387734 | -0,734728148 | 0,257362378 | -2,854838976 | 0,004305865 | 0,037010017 | 1,664083871 |
| FLI1 | 72,70673409 | 114,395037 | 140,6574533 | 70,97413121 | 69,57373462 | 67,27500528 | 348,2047301 | -0,670879118 | 0,23504276 | -2,8542854 | 0,004313376 | 0,03704623 | 1,592042797 |
| GPAA1 | 8217,592064 | 4018,290035 | 3808,67609 | 2872,647887 | 2760,506245 | 3786,971206 | 2877,514499 | -0,767946768 | 0,269083622 | -2,853933517 | 0,004318157 | 0,037058955 | 1,702844583 |
| TIMM8B | 2016,746315 | 1556,824412 | 1422,961809 | 1253,475334 | 1088,4923 | 895,3691611 | 1299,627618 | -0,62539538 | 0,219198759 | -2,853097269 | 0,004329537 | 0,037128256 | 1,542633536 |
| SPOCD1 | 472,5937716 | 422,07824 | 299,067789 | 267,0552056 | 242,3859142 | 178,5845595 | 451,5316889 | -0,793191918 | 0,278448187 | -2,848615845 | 0,004390986 | 0,037551986 | 1,732904221 |
| HDHD2 | 798,0429622 | 365,5381642 | 431,5316043 | 281,4906221 | 313,0818058 | 353,4995732 | 368,4690109 | -0,747028084 | 0,262445776 | -2,846409251 | 0,004421533 | 0,037688344 | 1,678331945 |
| TRAPPC10 | 619,7383525 | 553,5667882 | 446,553274 | 348,8558992 | 418,5645648 | 402,4268497 | 467,2721256 | -0,466803364 | 0,164011371 | -2,846164632 | 0,004424931 | 0,037688344 | 1,382043824 |
| LGALS1 | 24438,11822 | 21193,32421 | 21538,34324 | 12092,06724 | 11984,63687 | 17179,58998 | 12590,50615 | -0,703192784 | 0,247191326 | -2,844730818 | 0,004444897 | 0,037828766 | 1,628103915 |
| MRPS26 | 1871,332846 | 1059,797699 | 757,9115202 | 779,5124919 | 636,2630247 | 753,4800591 | 1438,485534 | -0,764692237 | 0,269000827 | -2,842713327 | 0,004473128 | 0,038040216 | 1,699007518 |
| STAT5A | 249,2802312 | 202,4923643 | 114,7109328 | 98,64201286 | 120,0708001 | 124,7645552 | 318,8504905 | -0,715213515 | 0,251708597 | -2,841434598 | 0,004491106 | 0,038164212 | 1,641726171 |
| FKBP2 | 1431,630216 | 1418,761436 | 1444,811511 | 943,1138791 | 923,5352193 | 1033,588717 | 1098,301754 | -0,566636693 | 0,199470868 | -2,840698986 | 0,004501478 | 0,038170333 | 1,481066781 |
| PDLIM4 | 1322,570115 | 1187,341591 | 1092,485074 | 784,3242974 | 868,5495258 | 1018,910534 | 940,3196391 | -0,430405304 | 0,15149931 | -2,840972034 | 0,004497626 | 0,038170333 | 1,347612115 |
| BTBD19 | 50,20226877 | 48,65076286 | 30,04333954 | 19,24722202 | 17,95451216 | 25,6868202 | 50,44312347 | -1,031924742 | 0,363305014 | -2,840381232 | 0,004505965 | 0,038174969 | 2,044750395 |
| ASH2L | 657,8228322 | 723,1870155 | 770,2019773 | 596,6638827 | 531,9024228 | 430,5600338 | 652,5649882 | -0,465212598 | 0,163851322 | -2,839236151 | 0,004522168 | 0,038254541 | 1,380520774 |
| GTDC2 | 309,8691762 | 188,028624 | 136,5606343 | 107,0626725 | 130,1702132 | 133,3268286 | 210,3172986 | -0,767909907 | 0,270756931 | -2,836159735 | 0,00456596 | 0,038466874 | 1,702801076 |
| NAA10 | 2087,721936 | 1305,681284 | 1033,764001 | 1069,423774 | 877,5267819 | 765,7118782 | 920,4332764 | -0,705512744 | 0,248767713 | -2,836030185 | 0,004567813 | 0,038466874 | 1,630724133 |
| POP7 | 394,6936993 | 399,7251867 | 404,2194774 | 288,7083303 | 212,0876749 | 192,0395605 | 320,6236748 | -0,791026214 | 0,278876357 | -2,836476432 | 0,004561434 | 0,038466874 | 1,730304821 |
| SMARCA4 | 3290,845274 | 2473,299593 | 1915,945699 | 1648,043386 | 1725,877481 | 1261,100553 | 1699,86859 | -0,728041066 | 0,256702293 | -2,836129965 | 0,004566386 | 0,038466874 | 1,65638847 |
| OTUB2 | 1033,474292 | 1192,601133 | 1189,443124 | 856,50138 | 792,2428491 | 875,7982505 | 603,4284756 | -0,437154609 | 0,154193006 | -2,835113087 | 0,004580947 | 0,03854858 | 1,353931368 |
| MARCKS | 560,8805201 | 607,477093 | 527,1240483 | 406,5975652 | 391,6327965 | 282,5550222 | 355,7337709 | -0,649366283 | 0,229159035 | -2,833692686 | 0,004601356 | 0,038662402 | 1,568479075 |
| RBFA | 458,7448698 | 337,9255691 | 344,1327983 | 300,7378441 | 237,8972861 | 240,9668371 | 262,0476588 | -0,545759048 | 0,192582568 | -2,833896418 | 0,004598423 | 0,038662402 | 1,459788184 |
| B4GALT6 | 512,409364 | 628,5152607 | 617,2540669 | 405,3946138 | 471,3059442 | 430,5600338 | 580,8794103 | -0,429514277 | 0,151647808 | -2,832314437 | 0,004621238 | 0,038786685 | 1,34678007 |
| WBP2 | 924,4141905 | 1005,887394 | 919,0530686 | 706,1324579 | 671,049892 | 807,3000633 | 1346,108972 | -0,384254096 | 0,135673893 | -2,832188928 | 0,004623052 | 0,038786685 | 1,305184805 |
| PYCR2 | 1357,19237 | 974,3301427 | 736,0618187 | 698,9147497 | 700,2259743 | 595,6895922 | 1000,05133 | -0,619035415 | 0,218750904 | -2,829864487 | 0,004656772 | 0,039007764 | 1,535847971 |
| WDPCP | 84,8245231 | 57,85496124 | 57,35546639 | 21,65312477 | 44,8862804 | 36,69545742 | 64,27925865 | -0,940237247 | 0,33225607 | -2,829857248 | 0,004656878 | 0,039007764 | 1,918843761 |
| SMURF1 | 268,322471 | 286,6450352 | 293,6053637 | 210,5164909 | 191,8888487 | 211,6104711 | 322,7102469 | -0,469082887 | 0,165928982 | -2,827009969 | 0,004698487 | 0,039302157 | 1,384229242 |
| SRSF2 | 2362,968858 | 2361,534327 | 3128,604131 | 1636,013872 | 1340,977627 | 1974,215609 | 1950,629944 | -0,665762567 | 0,235713372 | -2,824458203 | 0,004736062 | 0,039498832 | 1,58640658 |
| PTPLAD1 | 952,111994 | 1016,406478 | 1366,971949 | 797,5567625 | 810,1973613 | 784,059607 | 989,9077525 | -0,480738227 | 0,170224806 | -2,824137324 | 0,004740807 | 0,039509068 | 1,39545754 |
| TJP2 | 1346,805693 | 1249,141209 | 1259,089048 | 1029,726378 | 820,2967744 | 933,2878005 | 714,2624996 | -0,469832529 | 0,166438937 | -2,822852254 | 0,00475985 | 0,039631828 | 1,384948691 |
| CBX2 | 384,307023 | 286,6450352 | 300,4333954 | 186,4574633 | 243,5080712 | 232,4045637 | 223,8664208 | -0,547757138 | 0,19415273 | -2,821269308 | 0,004783403 | 0,039716738 | 1,461811348 |
| SUCLG1 | 225,0446531 | 247,1984707 | 262,1964178 | 175,630901 | 181,7894356 | 165,1295584 | 279,6761038 | -0,493150027 | 0,174848844 | -2,820436304 | 0,004795839 | 0,039761225 | 1,407514736 |
| TAF1C | 278,7091473 | 177,5095402 | 192,5504943 | 132,3246514 | 127,9258991 | 80,73000633 | 157,2522411 | -0,923801226 | 0,327641655 | -2,819547555 | 0,00480914 | 0,039812737 | 1,89710723 |
| CXCL3 | 32,89114161 | 65,74427414 | 72,37713616 | 33,68263854 | 35,90902432 | 26,91000211 | 182,8656751 | -0,844535107 | 0,299707999 | -2,817859748 | 0,004834492 | 0,039976629 | 1,795686013 |
| HAGH | 403,3492629 | 270,8664094 | 285,4117256 | 163,6013872 | 222,187088 | 242,190019 | 279,6051 | -0,606957114 | 0,215404427 | -2,817755985 | 0,004836055 | 0,039976629 | 1,523043464 |
| DNPEP | 406,8114883 | 399,7251867 | 528,4896546 | 298,3319413 | 314,2039628 | 183,4772871 | 354,6650513 | -0,745815928 | 0,26505016 | -2,813867106 | 0,004894946 | 0,040265925 | 1,676922398 |
| FAM50B | 102,1356503 | 55,22519027 | 73,7427425 | 50,52395781 | 30,29823927 | 40,36500317 | 43,46515531 | -0,922855741 | 0,327976217 | -2,8137886 | 0,004896141 | 0,040265925 | 1,895864349 |
| FBXW2 | 1511,261401 | 1254,400751 | 1164,86221 | 907,0253378 | 789,9985351 | 1064,168265 | 1090,950461 | -0,508846445 | 0,18083634 | -2,813850609 | 0,004895197 | 0,040265925 | 1,422912004 |
| LRP12 | 332,3736415 | 302,423661 | 441,0908487 | 227,3578101 | 252,4853273 | 264,2072935 | 237,9808009 | -0,532996072 | 0,189386635 | -2,814327801 | 0,004887936 | 0,040265925 | 1,446930947 |

| | | | | | | | | | | | | | |
|---|---|---|---|---|---|---|---|---|---|---|---|---|---|
| RAP1GAP2 | 406,8114883 | 310,3129739 | 221,2282275 | 235,7784698 | 178,4229646 | 167,5759222 | 330,2313363 | -0,685881252 | 0,243682463 | -2,814651669 | 0,004883013 | 0,040265925 | 1,608684326 |
| BRAT1 | 1317,376777 | 787,6164041 | 535,3176863 | 535,3133625 | 571,1779181 | 398,757304 | 653,7860063 | -0,808737926 | 0,287508922 | -2,812914185 | 0,004909475 | 0,040346045 | 1,751678399 |
| RHOBTB2 | 472,5937716 | 391,8358738 | 289,5085446 | 282,6935734 | 231,1643441 | 181,0309233 | 427,4725306 | -0,729388667 | 0,259410578 | -2,811715209 | 0,004927811 | 0,040437569 | 1,657936402 |
| THRAP3 | 2929,042716 | 2600,843485 | 2866,407713 | 1929,534008 | 1815,650042 | 2283,680634 | 2471,964354 | -0,477817461 | 0,169957913 | -2,811386969 | 0,004932842 | 0,040449304 | 1,392635264 |
| LPIN1 | 472,5937716 | 454,950377 | 421,9723599 | 340,4352395 | 280,5392525 | 369,4009381 | 323,7939273 | -0,446514593 | 0,158847879 | -2,810957232 | 0,004939435 | 0,04045441 | 1,362744023 |
| PPTC7 | 1196,198887 | 902,0114411 | 650,0286191 | 536,5163139 | 594,7432153 | 673,9732347 | 1022,317406 | -0,60462709 | 0,215102649 | -2,810877009 | 0,004940667 | 0,04045441 | 1,520585659 |
| OGFR | 458,7448698 | 554,8816737 | 473,8654009 | 384,9444404 | 332,158475 | 384,079121 | 639,941577 | -0,436333811 | 0,155257498 | -2,810388003 | 0,004948181 | 0,040486426 | 1,353161289 |
| KCTD5 | 2601,862413 | 1608,104945 | 1183,980699 | 1006,870302 | 1023,407193 | 1176,701001 | 1613,634176 | -0,749292999 | 0,266798702 | -2,80845819 | 0,004977935 | 0,040670631 | 1,68096886 |
| CYFIP1 | 4616,877614 | 3007,143099 | 2533,199766 | 2237,48956 | 2125,365377 | 1502,067391 | 2110,978649 | -0,791928742 | 0,282398682 | -2,804293339 | 0,0050427 | 0,041110084 | 1,731387611 |
| RRAS | 136,7579046 | 166,9904563 | 155,6791231 | 102,250867 | 94,26118885 | 100,300917 | 184,3528754 | -0,634558368 | 0,226304427 | -2,804003329 | 0,005047238 | 0,041117242 | 1,552462443 |
| FAM32A | 1435,092442 | 1387,204184 | 1102,044319 | 946,7227332 | 977,3987558 | 655,625506 | 1217,029938 | -0,604603181 | 0,21586196 | -2,800878774 | 0,005096366 | 0,041277992 | 1,520560459 |
| AKNA | 164,4557081 | 222,2156466 | 180,2600372 | 113,0774294 | 121,1929571 | 114,9790999 | 166,9008345 | -0,701801875 | 0,250772054 | -2,79856493 | 0,005133025 | 0,041455356 | 1,626535009 |
| NHP2L1 | 3716,699002 | 3036,07058 | 2768,084057 | 2385,452579 | 2247,680491 | 1772,390594 | 2064,39116 | -0,571390125 | 0,204171576 | -2,798578221 | 0,005132814 | 0,041455356 | 1,48595469 |
| ORAI2 | 190,4223988 | 219,5858756 | 271,7556622 | 178,0368037 | 150,3690393 | 144,3354659 | 226,1210948 | -0,533046944 | 0,190529421 | -2,797714606 | 0,005146556 | 0,041534781 | 1,446981969 |
| PFAS | 541,8382802 | 907,2709831 | 928,612313 | 561,7782928 | 501,6041835 | 426,890488 | 691,7921568 | -0,675695029 | 0,241583231 | -2,796945078 | 0,00515883 | 0,041603947 | 1,59736613 |
| NFKBID | 62,32005779 | 30,2423661 | 36,87137125 | 16,84131927 | 5,61078505 | 31,80272977 | 52,52231554 | -1,251121313 | 0,447656282 | -2,794825768 | 0,005192769 | 0,041817613 | 2,380263534 |
| TIMM13 | 1564,925896 | 1270,179376 | 1234,508134 | 1014,08801 | 896,603451 | 766,9350602 | 1047,962045 | -0,603258642 | 0,215957468 | -2,793414132 | 0,005215487 | 0,041940433 | 1,519144013 |
| GMEB2 | 261,3980202 | 168,3053418 | 185,7224626 | 133,5276028 | 148,1247253 | 95,4081893 | 197,3552943 | -0,699427993 | 0,25049754 | -2,792155138 | 0,005235825 | 0,04205763 | 1,623860829 |
| TUBGCP2 | 843,0518929 | 731,0763284 | 629,544524 | 525,6897515 | 478,0388863 | 239,7436552 | 492,3089326 | -0,824585265 | 0,295333421 | -2,792048602 | 0,005237549 | 0,04205763 | 1,771025844 |
| COMT | 2252,177644 | 1354,332047 | 1435,252266 | 1184,907106 | 1105,324655 | 1004,232352 | 1101,032599 | -0,612749217 | 0,219608296 | -2,790191577 | 0,005267686 | 0,042189592 | 1,52917044 |
| MMP9 | 79,63118495 | 76,263358 | 118,8077518 | 42,10329817 | 65,08510658 | 40,36500317 | 50,06276006 | -0,895710122 | 0,321036141 | -2,790060085 | 0,005269826 | 0,042189592 | 1,860525446 |
| SH3TC1 | 55,39560692 | 42,07633545 | 40,96819028 | 16,84131927 | 24,68745422 | 22,01727445 | 20,23660441 | -1,113388824 | 0,399061371 | -2,79001904 | 0,005270494 | 0,042189592 | 2,163532546 |
| TTI2 | 180,0357225 | 163,0457999 | 163,8727611 | 123,9039918 | 94,26118885 | 117,4254638 | 177,1116757 | -0,59467409 | 0,213350329 | -2,787312737 | 0,005314716 | 0,042464809 | 1,510131403 |
| ACOT7 | 2371,624421 | 1477,931283 | 1623,705941 | 1006,870302 | 932,5124754 | 1384,641927 | 1723,461411 | -0,718974545 | 0,258095137 | -2,785695824 | 0,005341297 | 0,0424989 | 1,646011648 |
| PLD6 | 69,24450865 | 72,31870155 | 105,1516884 | 37,29149267 | 40,39765236 | 46,48091274 | 49,87342837 | -0,994435424 | 0,356904526 | -2,786278543 | 0,005331704 | 0,0424989 | 1,992300717 |
| PPP1R14B | 8946,390518 | 5618,505668 | 4574,781248 | 4348,669226 | 4136,270739 | 4116,007141 | 4044,267058 | -0,602694662 | 0,216276474 | -2,786686183 | 0,005325002 | 0,0424989 | 1,518550264 |
| CCDC59 | 496,8293496 | 548,3072463 | 517,5648039 | 413,8152735 | 398,3657386 | 407,3195774 | 648,0651873 | -0,359399163 | 0,129063995 | -2,784658588 | 0,005358411 | 0,042511728 | 1,282891503 |
| THAP11 | 773,8073842 | 439,1717512 | 398,7570521 | 318,7821147 | 331,036318 | 370,62412 | 425,7796676 | -0,655732553 | 0,235838159 | -2,780434497 | 0,005428621 | 0,043007848 | 1,575415694 |
| PASK | 86,55563582 | 76,263358 | 90,13001862 | 37,29149267 | 28,05392525 | 61,15909571 | 59,4922972 | -1,002912121 | 0,360743202 | -2,780127568 | 0,005433755 | 0,043018183 | 2,004041134 |
| TMEM156 | 122,9090029 | 114,395037 | 162,5071548 | 90,22135323 | 60,59647854 | 81,95318825 | 63,92365065 | -0,78372105 | 0,281956476 | -2,779581666 | 0,005442897 | 0,043060211 | 1,721565474 |
| SPRED1 | 491,6360114 | 696,8893058 | 932,709132 | 481,1805506 | 478,0388863 | 448,9077625 | 702,3970514 | -0,593336719 | 0,213550962 | -2,778431494 | 0,005462203 | 0,043118021 | 1,508732167 |
| SPRED2 | 1016,163164 | 828,3778541 | 856,2351768 | 747,0328047 | 603,7204714 | 693,5441453 | 696,3218894 | -0,400404339 | 0,144140137 | -2,777882335 | 0,005471443 | 0,04312257 | 1,319877777 |
| OGG1 | 829,2029911 | 546,9923608 | 387,8322013 | 338,0293368 | 305,2267067 | 245,8595647 | 436,2325315 | -0,986940166 | 0,355629588 | -2,775191373 | 0,005516923 | 0,043310354 | 1,981976924 |
| PHF1 | 214,6579768 | 145,9522886 | 131,0982089 | 108,2656239 | 99,8719739 | 86,8459159 | 157,7072945 | -0,729691337 | 0,263014408 | -2,774339785 | 0,005531387 | 0,043363296 | 1,658284266 |
| RNASEH2C | 1381,427948 | 863,8797621 | 737,427425 | 674,8557222 | 629,5300827 | 594,4664103 | 719,3108396 | -0,649665225 | 0,234273232 | -2,773109066 | 0,005552351 | 0,043466975 | 1,568804115 |
| MFSD6 | 69,24450865 | 84,15267089 | 114,7109328 | 48,11805506 | 47,13059442 | 66,05182336 | 80,05457734 | -0,74374975 | 0,268388044 | -2,771173179 | 0,005585471 | 0,043665402 | 1,674522487 |
| POLE3 | 3493,385462 | 2778,353025 | 1995,150867 | 1966,8255 | 1926,743586 | 1835,996053 | 2142,228587 | -0,5282558 | 0,190716481 | -2,769848713 | 0,005608234 | 0,043812862 | 1,442184558 |
| APBA2 | 257,9357947 | 249,8282417 | 223,9594402 | 178,0368037 | 142,5139403 | 154,1209212 | 101,1991166 | -0,62391845 | 0,225423623 | -2,767759836 | 0,005644303 | 0,043972332 | 1,541055105 |
| NOTCH2 | 4372,790721 | 4389,087741 | 3603,835138 | 3114,441113 | 3220,590619 | 3258,556619 | 3636,781198 | -0,366025895 | 0,132236453 | -2,767965163 | 0,005640748 | 0,043972332 | 1,288797763 |
| SYS1 | 875,9430345 | 756,0591526 | 715,5777236 | 581,0255148 | 636,2630247 | 603,0286837 | 1292,709023 | -0,364518161 | 0,131687539 | -2,768053567 | 0,005639219 | 0,043972332 | 1,287451568 |
| SLC45A3 | 65,78228322 | 47,33587738 | 62,81789176 | 26,46493028 | 31,42039628 | 11,00863723 | 42,83919801 | -1,34519898 | 0,486213986 | -2,766680965 | 0,005663014 | 0,044087529 | 2,540652354 |
| TSPAN18 | 219,851315 | 202,4923643 | 177,5288245 | 103,4538184 | 116,704329 | 170,0222861 | 220,2033727 | -0,619582063 | 0,224148553 | -2,764158211 | 0,005706986 | 0,044341167 | 1,536430025 |
| NEDD8 | 2172,546459 | 2038,072498 | 1973,301165 | 1697,364392 | 1347,710569 | 1190,156002 | 2090,10804 | -0,54591874 | 0,197573797 | -2,763113069 | 0,005725293 | 0,044418467 | 1,459949776 |
| ADAM19 | 5842,505418 | 3817,112556 | 2667,029187 | 2207,415776 | 2418,248357 | 1154,683727 | 2280,023515 | -1,092324665 | 0,395540313 | -2,761601361 | 0,005751866 | 0,04450975 | 2,132173244 |
| SAMD1 | 688,9828611 | 481,2480867 | 379,6385633 | 323,5939202 | 325,4255329 | 194,4859243 | 430,9256455 | -0,875613544 | 0,317236998 | -2,760124294 | 0,005777937 | 0,044641918 | 1,834788212 |
| REEP4 | 590,3094363 | 466,7843464 | 309,9926398 | 309,1585037 | 235,6529721 | 325,3663892 | 287,9468762 | -0,649616327 | 0,235507071 | -2,758372918 | 0,005808989 | 0,044789416 | 1,568750944 |
| DIS3L2 | 527,9893785 | 514,1202237 | 510,7367722 | 350,0588505 | 252,4853273 | 409,7659412 | 468,2093515 | -0,6180321 | 0,22409267 | -2,757930908 | 0,005816849 | 0,044819261 | 1,534780245 |
| TOX2 | 140,22013 | 144,6374031 | 118,8077518 | 99,84496424 | 83,03961875 | 88,06909782 | 936,2893233 | -0,574844346 | 0,208620006 | -2,755461266 | 0,005860944 | 0,045097157 | 1,489516748 |
| TMEM233 | 107,3289884 | 68,3740451 | 90,13001862 | 51,72690918 | 37,03118133 | 59,93591379 | 60,93787549 | -0,834009886 | 0,302854424 | -2,753830949 | 0,005890218 | 0,045260406 | 1,782633207 |
| RAP2B | 934,8008668 | 691,6297639 | 748,3522758 | 556,9664873 | 453,3514321 | 621,3764124 | 619,4181556 | -0,54035465 | 0,19642603 | -2,750931986 | 0,005942598 | 0,045538299 | 1,454329984 |

| | | | | | | | | | | | | | |
|---|---|---|---|---|---|---|---|---|---|---|---|---|---|
| CISD3 | 716,6806646 | 511,4904528 | 430,1659979 | 396,9739542 | 370,3118133 | 360,8386647 | 578,5447984 | -0,552464228 | 0,200865423 | -2,750419754 | 0,005951897 | 0,045578465 | 1,466588598 |
| TLE3 | 600,6961126 | 439,1717512 | 427,4347853 | 292,3171845 | 346,7465161 | 188,3700148 | 505,9762731 | -0,82437347 | 0,299921152 | -2,748633976 | 0,005984417 | 0,045689964 | 1,770765867 |
| UFSP1 | 62,32005779 | 40,76144996 | 42,33379662 | 31,27673579 | 17,95451216 | 17,1245468 | 30,32297097 | -1,123589146 | 0,408820231 | -2,748369727 | 0,005989243 | 0,045689964 | 2,178883628 |
| SOX7 | 166,1868208 | 139,3778612 | 211,6689831 | 103,4538184 | 89,77256081 | 121,0950095 | 157,1039209 | -0,720079542 | 0,262042129 | -2,747953335 | 0,005996854 | 0,045704655 | 1,647272854 |
| B9D2 | 159,2623699 | 95,98664024 | 111,9797201 | 74,58298534 | 74,06236266 | 62,38227762 | 94,2706096 | -0,790296245 | 0,287673773 | -2,747196027 | 0,00601072 | 0,045776952 | 1,729429549 |
| TMEM54 | 728,7984536 | 570,6602995 | 595,4043654 | 416,2211762 | 397,2435816 | 510,0668582 | 547,7044111 | -0,516348188 | 0,187968692 | -2,746990369 | 0,00601449 | 0,045776952 | 1,430330148 |
| SAR1A | 2144,848656 | 1974,957995 | 1767,094607 | 1378,582277 | 1453,193328 | 1263,546917 | 1557,231783 | -0,523125542 | 0,190475464 | -2,746419567 | 0,006024966 | 0,045825637 | 1,437065223 |
| MEX3D | 50,20226877 | 43,39122093 | 69,64592348 | 26,46493028 | 19,07666917 | 28,13318402 | 31,02535677 | -1,152041086 | 0,419949987 | -2,743281629 | 0,006082849 | 0,046167467 | 2,222280744 |
| PWP2 | 604,158338 | 624,5706043 | 532,5864736 | 454,7156203 | 391,6327965 | 348,6068455 | 455,9981741 | -0,55945158 | 0,204055237 | -2,741667353 | 0,006112821 | 0,046337 | 1,473708902 |
| MAP6D1 | 91,74897397 | 71,00381607 | 42,33379662 | 27,66788166 | 43,76412339 | 36,69545742 | 40,88600634 | -0,90932124 | 0,331785334 | -2,740691488 | 0,006131005 | 0,0464122 | 1,878161652 |
| FAM109B | 275,2469219 | 102,5610677 | 99,68926301 | 70,97413121 | 56,1078505 | 81,95318825 | 65,01302364 | -1,186150168 | 0,432858249 | -2,740273912 | 0,0061388 | 0,046439919 | 2,275447291 |
| SALL1 | 2196,782037 | 1463,467542 | 1177,152667 | 911,8371433 | 983,0095408 | 1072,730539 | 650,4149558 | -0,704108896 | 0,257064751 | -2,739033229 | 0,006162015 | 0,046584166 | 1,62913809 |
| OXLD1 | 126,3712283 | 76,263358 | 66,91471079 | 69,77117983 | 32,54255329 | 35,47227551 | 90,00321322 | -0,960406838 | 0,350759175 | -2,738080448 | 0,006179896 | 0,046628057 | 1,945858546 |
| ZFAND2B | 309,8691762 | 195,9179369 | 211,6689831 | 178,0368037 | 152,6133534 | 139,4427382 | 183,0488382 | -0,603119068 | 0,220316692 | -2,737509637 | 0,006190631 | 0,046674862 | 1,51899705 |
| USF2 | 1788,239436 | 1058,482814 | 1041,957639 | 940,7079763 | 802,3422622 | 848,8882484 | 1067,550932 | -0,583661706 | 0,213289039 | -2,736482414 | 0,006209991 | 0,046741529 | 1,498648146 |
| SRA1 | 1521,648078 | 1182,082049 | 991,4302048 | 820,4128387 | 756,3338248 | 912,4937079 | 1212,743818 | -0,568997103 | 0,207984101 | -2,735772111 | 0,006223411 | 0,046765082 | 1,483491954 |
| ITGA5 | 3950,399219 | 3128,112563 | 2615,136146 | 2440,788343 | 2363,262663 | 2097,756983 | 3841,165686 | -0,489494304 | 0,178938809 | -2,735540193 | 0,006227798 | 0,046766767 | 1,403952673 |
| KLHL25 | 183,4979479 | 114,395037 | 79,20516787 | 64,95937432 | 76,30667668 | 73,39091485 | 119,1526689 | -0,801409736 | 0,293286084 | -2,73251879 | 0,006285209 | 0,047134869 | 1,742803283 |
| RPL15 | 49407,68804 | 23499,63335 | 21023,50965 | 20448,97045 | 18578,43146 | 15330,13893 | 22501,72749 | -0,789059105 | 0,288938051 | -2,730893711 | 0,006316284 | 0,04727324 | 1,727947165 |
| NUDT16L1 | 455,2826444 | 272,1812949 | 225,3250465 | 209,3135395 | 187,4002207 | 190,8163786 | 290,7253678 | -0,692172668 | 0,253551881 | -2,729905477 | 0,006335249 | 0,047383611 | 1,615714919 |
| LIG4 | 399,8870375 | 330,0362562 | 292,2397573 | 218,9371505 | 179,5451216 | 211,6104711 | 163,0615262 | -0,743244678 | 0,272441745 | -2,728086611 | 0,006370288 | 0,047613982 | 1,673936357 |
| CCDC103 | 93,48008668 | 94,67175476 | 102,4204757 | 56,53871469 | 57,23000751 | 61,15909571 | 97,57061848 | -0,733624917 | 0,268962243 | -2,727613018 | 0,00637944 | 0,047650684 | 1,662811829 |
| PMPCA | 2125,806416 | 1397,723268 | 1117,065988 | 1039,349989 | 1059,316218 | 929,6182547 | 1398,38626 | -0,614530739 | 0,225330294 | -2,727244201 | 0,006386575 | 0,047672285 | 1,531059913 |
| SPG11 | 192,1535115 | 228,790074 | 221,2282275 | 139,5423597 | 152,6133534 | 96,63137122 | 198,4982497 | -0,724409579 | 0,26571587 | -2,726256351 | 0,006405723 | 0,047783458 | 1,652224327 |
| ANKRD9 | 131,5645664 | 105,1908386 | 92,8612313 | 69,77117983 | 60,59647854 | 72,16773293 | 65,88688492 | -0,696971674 | 0,255725558 | -2,725467411 | 0,006421051 | 0,04786602 | 1,621098411 |
| PLEKHA4 | 102,1356503 | 93,35686927 | 102,4204757 | 57,74166607 | 65,08510658 | 39,14182125 | 40,98171107 | -0,875730034 | 0,321348166 | -2,725175141 | 0,006426739 | 0,04786736 | 1,834936368 |
| ADRBK1 | 1005,776488 | 604,847322 | 525,7584419 | 365,6972184 | 392,7549535 | 154,1209212 | 546,1844355 | -1,226059967 | 0,449982224 | -2,724685337 | 0,00643628 | 0,047884215 | 2,33927256 |
| TMEM150A | 77,90007224 | 77,57824348 | 64,1834981 | 34,88558992 | 51,61922246 | 44,03454891 | 73,60821921 | -0,745429151 | 0,273946063 | -2,721079987 | 0,006506901 | 0,048313636 | 1,676472886 |
| BCS1L | 884,5985981 | 579,8644979 | 432,8972106 | 412,6123221 | 384,8998545 | 282,5550222 | 543,2970608 | -0,810877392 | 0,298169664 | -2,719516737 | 0,006537739 | 0,048446541 | 1,754278003 |
| PER2 | 70,97562137 | 56,54007576 | 60,08667908 | 32,47968716 | 38,15333834 | 36,69545742 | 49,27961499 | -0,797589716 | 0,293748329 | -2,715214476 | 0,006623286 | 0,049015808 | 1,738194729 |
| DNAJB12 | 500,291575 | 435,2270948 | 467,0373692 | 341,6381909 | 342,2578881 | 364,5082104 | 381,4314463 | -0,418323822 | 0,154105392 | -2,714530731 | 0,006636974 | 0,049084772 | 1,336374003 |
| TGFB1I1 | 1985,586286 | 1399,038154 | 1041,957639 | 998,4496424 | 957,1999296 | 966,3137122 | 913,0839334 | -0,598069455 | 0,220355514 | -2,714111591 | 0,006645378 | 0,049114587 | 1,513689664 |
| DECR2 | 472,5937716 | 327,4064852 | 325,0143096 | 276,6788166 | 269,3176824 | 216,5031988 | 247,5513037 | -0,556344437 | 0,205259221 | -2,710447957 | 0,006719239 | 0,049519389 | 1,470538379 |
| FXYD5 | 11704,05308 | 9761,709824 | 8465,393718 | 8216,157901 | 7115,597601 | 7454,070585 | 6645,547341 | -0,393326882 | 0,145161746 | -2,709576686 | 0,006736913 | 0,049519389 | 1,313418682 |
| KIAA0226 | 1125,223266 | 753,4293816 | 614,5228542 | 532,9074597 | 567,8114471 | 593,2432284 | 600,9106707 | -0,555026529 | 0,204849656 | -2,709433536 | 0,006739821 | 0,049519389 | 1,469195649 |
| PLEKHB2 | 3500,309912 | 2849,356841 | 2281,928199 | 1927,128105 | 2127,609691 | 1738,1415 | 2149,440929 | -0,574857433 | 0,212156629 | -2,709589769 | 0,006736647 | 0,049519389 | 1,48953026 |
| RANBP10 | 202,5401878 | 293,2194626 | 192,5504943 | 150,368922 | 133,5366842 | 95,4081893 | 165,8652401 | -0,860848478 | 0,317572013 | -2,710718965 | 0,00671375 | 0,049519389 | 1,816106085 |
| C17orf62 | 167,9179335 | 209,0667918 | 217,1314085 | 138,3394083 | 140,2696263 | 129,6572829 | 182,6757622 | -0,545644557 | 0,201429303 | -2,708863844 | 0,006751404 | 0,049558183 | 1,459672341 |
| SF3B5 | 3611,101126 | 1863,192729 | 1768,460214 | 1581,88106 | 1689,968457 | 1004,232352 | 1809,988584 | -0,759445351 | 0,280390916 | -2,708523378 | 0,006758335 | 0,049558183 | 1,692839682 |
| XAB2 | 476,055997 | 397,0954158 | 338,670373 | 297,12899 | 300,7380787 | 220,1727445 | 695,047756 | -0,563726772 | 0,208116996 | -2,708701277 | 0,006754713 | 0,049558183 | 1,478082475 |
| RPL36AL | 3600,71445 | 4077,459882 | 3730,836528 | 2970,086948 | 2398,049531 | 3254,887073 | 2890,015192 | -0,404257011 | 0,149295134 | -2,707770853 | 0,006773677 | 0,049638285 | 1,32340718 |
| TM2D1 | 434,5092918 | 389,2061029 | 435,6284233 | 287,505379 | 301,8602357 | 299,679569 | 368,4776134 | -0,501491595 | 0,185266005 | -2,706873263 | 0,006792018 | 0,04967931 | 1,415676467 |
| YRDC | 268,322471 | 353,7041948 | 247,174748 | 199,6899285 | 179,5451216 | 135,7731925 | 459,0433231 | -0,755849555 | 0,279258384 | -2,706631558 | 0,006796965 | 0,04967931 | 1,688625675 |
| CLIC3 | 69,24450865 | 69,68893058 | 132,4638152 | 25,2619789 | 31,42039628 | 69,72136911 | 38,03527126 | -1,108747212 | 0,40995515 | -2,704557347 | 0,006839546 | 0,049893158 | 2,156582955 |
| HCCS | 415,4670519 | 502,2862544 | 512,1023785 | 386,1473918 | 329,914161 | 344,9372998 | 579,7222502 | -0,433484026 | 0,16027633 | -2,704604149 | 0,006838583 | 0,049893158 | 1,350491001 |
| TOMM34 | 1909,417326 | 1425,335863 | 1409,305746 | 1083,85919 | 1059,316218 | 1250,091916 | 1552,812723 | -0,482510012 | 0,178461436 | -2,703721445 | 0,006856774 | 0,049945017 | 1,397172364 |
