# Supplementary material for: Analysis of the Role of FRMD5 in the Biology of Papillary Thyroid Carcinoma
Source: Int J Mol Sci. 2021 Jun 23;22(13):6726. doi: 10.3390/ijms22136726 (PMC8268710; doi:10.3390/ijms22136726)
Supplement: Supplementary file 1 [file ijms-22-06726-s001.zip › Supplementary Table S3.pdf]

Supplementary Table S3. Genes upregulated in BCPAP siFRMD5 (in comparison with BCPAP siNEG).

gene	BCPAP siNEG 1	BCPAP siNEG 2	BCPAP siNEG 3	BCPAP siFRMD5 1	BCPAP siFRMD5 2	BCPAP siFRMD5 3	baseMean	log2FoldChange	lfcSE	stat	pvalue	padj	Fold-change
CXCL5	1341,291084	1175,972462	1045,282048	5155,835281	4958,072347	5102,272896	1564,995775	2,093973747	0,167222623	12,52207215	5,65395E-36	7,28116E-32	4,269223664
WWC1	108,0328593	76,75803521	96,48757365	467,9286856	443,5508402	421,9857731	553,6319914	2,255069318	0,186197241	12,11118546	9,21526E-34	5,93371E-30	4,773572324
CLDN12	91,19656951	64,45706803	105,5332837	391,5824264	404,0442876	342,4164218	228,1662748	2,143539872	0,206263465	10,39224215	2,68955E-25	8,659E-22	4,418448511
CTGF	828,4857584	1006,219115	891,5049774	2471,402295	2349,742001	2980,721541	1418,614786	1,515825129	0,148318501	10,22006777	1,61228E-24	4,15259E-21	2,859623333
ESM1	332,5167227	251,9238079	343,7369811	1321,282841	1142,09852	1234,666001	388,1158562	1,998138281	0,206926749	9,656258973	4,62444E-22	9,92558E-19	3,994841548
KRTAP2-3	118,5555404	227,8139122	161,8177016	757,3056359	728,1775939	775,1306468	388,8171107	2,147195154	0,232842017	9,221682506	2,92474E-20	5,38069E-17	4,429657499
LRRC38	58,22550207	55,60037166	57,28949685	224,1132126	280,1373727	246,7543927	76,84336239	2,133552491	0,235909789	9,043933705	1,51133E-19	2,16254E-16	4,387966438
HIPK2	342,3378917	388,7105629	444,2448703	1043,604108	1048,719395	963,772592	1419,56532	1,381486993	0,16056573	8,603872023	7,70709E-18	9,92519E-15	2,605367694
PDGFB	14,03024146	14,26912193	16,08126228	125,6019104	120,3154101	89,40376549	32,14058703	2,929371145	0,34461508	8,500414852	1,88914E-17	2,21167E-14	7,617782754
RASA2	65,24062281	76,26599652	44,22347126	223,4975169	226,2648011	297,7145391	152,1862722	1,984347684	0,234549452	8,460252908	2,66798E-17	2,86319E-14	3,956837133
EIF5A2	105,226811	111,692782	101,5129681	331,8599494	266,6692298	309,3370286	231,7421017	1,509300655	0,180906416	8,342991311	7,24345E-17	7,17547E-14	2,846720111
ZNF460	199,2294288	185,4985851	225,1376719	547,353423	522,5639453	487,2505219	302,5544539	1,358966702	0,164300435	8,271230102	1,32584E-16	1,21958E-13	2,565014004
LPP	88,39052122	115,1370528	98,49773143	274,600255	302,5842776	259,2709199	290,0399471	1,46176849	0,177634178	8,229094803	1,88633E-16	1,59274E-13	2,754458047
CALB2	324,0985778	301,6197153	350,7725334	796,0944612	826,9439753	747,4154795	514,0301461	1,282686298	0,156647241	8,188374672	2,64777E-16	2,00577E-13	2,432915643
SETD7	415,9966594	470,388985	319,6150877	913,6923282	928,4039853	973,6070062	640,8412132	1,216864195	0,158843019	7,660797431	1,84782E-14	1,13315E-11	2,324409402
ITGB8	394,2497851	389,6946403	302,5287465	1097,785324	1098,102586	1213,209098	435,496939	1,647312526	0,225788847	7,295809981	2,96868E-13	1,62932E-10	3,132495693
OTUD4	280,6048293	257,3362334	278,4068531	626,7781605	740,7478606	604,3694547	542,5753524	1,273313501	0,174599438	7,29276975	3,03647E-13	1,62932E-10	2,417160885
NTSR1	135,3918301	145,1514127	140,7110449	344,1738622	395,0655257	373,7077397	127,8501179	1,399755221	0,194192548	7,208078966	5,67467E-13	2,92314E-10	2,638568103
MARCH4	258,1564429	327,205727	266,3459064	666,1826814	595,2919171	626,7203961	501,8227758	1,144695225	0,158955401	7,201361002	5,96145E-13	2,95275E-10	2,210994176
ZBED2	54,71794171	27,55416649	36,18284012	222,8818213	162,5155912	199,370397	78,79548469	2,311659441	0,323925573	7,13639068	9,58134E-13	4,56995E-10	4,964537911
MYC	204,8415254	247,9874984	204,0310151	522,7255975	606,9643076	531,058367	966,3998161	1,333637813	0,189647554	7,032190941	2,03315E-12	9,0286E-10	2,520373967
PTPRJ	560,5081465	590,9384634	591,9914675	1263,407451	1105,285596	1148,838387	1255,810119	1,013341804	0,144077822	7,03329485	2,01712E-12	9,0286E-10	2,018581455
NGFR	96,10715402	62,48891328	98,49773143	382,9626874	263,077725	358,5090996	106,1805349	1,976671292	0,285454305	6,924650476	4,37053E-12	1,87612E-09	3,935839233
TRNP1	49,10584512	60,02871984	54,27426018	137,9158231	146,3538197	130,5294976	538,3258305	1,336455823	0,193398121	6,910386793	4,83334E-12	2,00786E-09	2,525301813
HECA	197,1248926	205,1801326	176,893885	366,9546008	423,7975639	381,7540786	291,1535873	1,010270504	0,146639112	6,88950233	5,59879E-12	2,25316E-09	2,014288742
GALNT1	226,5883996	280,9540904	207,0462518	607,0759	589,0067837	528,376254	516,2749932	1,265435632	0,185333956	6,827867149	8,61863E-12	3,26443E-09	2,403997894
NAPG	586,4640932	458,5800565	516,6105506	1137,189845	1189,685958	1046,024056	699,2217904	1,112064811	0,16475217	6,749925138	1,47921E-11	5,44266E-09	2,161547907
C12orf73	88,39052122	79,21822864	81,41139027	224,1132126	292,7076395	194,9002088	112,7878032	1,514794207	0,224637606	6,743279691	1,54851E-11	5,53935E-09	2,857580632
FGF2	656,6153005	309,984373	647,2708066	2082,282651	1796,650266	2181,451878	1068,024927	1,911088362	0,292749341	6,528070581	6,66223E-11	2,25779E-08	3,760927151
SEMA7A	3360,944343	3338,974532	3680,598903	8338,981734	9024,553633	7016,407516	5900,882068	1,23197511	0,189166636	6,512644827	7,3839E-11	2,4382E-08	2,348883419
INPP5A	129,0782215	83,15453814	131,6653349	301,0751675	307,0736586	299,5026144	233,2916555	1,412299499	0,217873321	6,482204844	9,03918E-11	2,83918E-08	2,661610566
NAMPT	2866,378331	3836,425644	2680,545405	7542,271577	5977,161828	7050,380946	2960,156158	1,131812723	0,174992617	6,46777414	9,9457E-11	3,04954E-08	2,191339055
CD40	1100,672443	1003,266883	1359,871741	2505,265555	2129,762334	2266,385455	1338,374151	0,99679448	0,154399471	6,455944922	1,07546E-10	3,22087E-08	1,995561139
NABP1	288,3214621	186,4826625	244,2341708	636,0135951	633,0027173	532,8464423	367,023685	1,329196475	0,206854565	6,425753646	1,31218E-10	3,84051E-08	2,512626924
ARMC10	140,3024146	158,4364573	155,7872283	432,2183386	321,4396777	435,3963379	205,9872543	1,388180235	0,217248987	6,389812242	1,6609E-10	4,64389E-08	2,617483123
UAP1	1159,599457	960,9515562	1180,967698	2085,361129	2099,234543	2208,273008	2048,584767	0,954566087	0,149461157	6,386716846	1,69485E-10	4,64389E-08	1,937996663
SLCO4A1	297,441119	401,0115301	367,8588745	831,1891126	674,3050222	896,7197678	531,045175	1,171077593	0,184127752	6,360136271	2,01575E-10	5,29772E-08	2,251798278
EPHA2	3451,4394	2847,919922	3342,892395	7004,769285	5667,394541	6479,090885	3747,76597	0,990422244	0,156029117	6,34767575	2,18592E-10	5,63006E-08	1,986766387
TSPAN4	403,3694421	370,0130928	472,3870793	788,7061135	771,2756512	759,9320067	740,5934129	0,902193579	0,143175057	6,301332079	2,95098E-10	7,45152E-08	1,868905445
MMP1	10209,1052	17243,49579	11563,43265	37349,32885	39920,47349	33755,2857	13652,48851	1,508656795	0,241233399	6,253930018	4,0025E-10	9,72533E-08	2,845449933
REL	77,16632805	61,99687459	62,31489132	191,4813437	153,5368293	166,2910038	150,4014686	1,346454037	0,215822792	6,238701786	4,41217E-10	1,05222E-07	2,542863528
RP2	229,3944479	214,0368289	183,9294373	391,5824264	409,4315448	470,2638065	418,5405529	1,013014328	0,162611724	6,22965122	4,67475E-10	1,09457E-07	2,01812331
ABCB10	82,07691256	108,2485112	75,38091691	207,4894303	193,9412581	182,3836816	254,8201477	1,118482263	0,180192722	6,20714451	5,3956E-10	1,21903E-07	2,171184404
PODXL	1302,006408	820,2284916	1103,576624	2095,212259	3139,873053	2539,960978	1031,513989	1,269697942	0,206804157	6,139615178	8,27216E-10	1,77548E-07	2,411110788
C9orf41	23,85141049	27,55416649	18,09142006	153,9239097	80,80885752	113,5427822	77,72284352	2,311438841	0,386265851	5,984062102	2,1764E-09	4,59471E-07	4,963778851
GGCT	675,5561265	608,6518561	716,6212501	1193,833844	1266,903311	1129,169558	838,0244294	0,845328035	0,141931216	5,955899332	2,58646E-09	5,2025E-07	1,796673221
MSANTD3	306,560776	226,8298348	307,554141	569,5184661	606,9643076	484,5684089	958,9325728	0,988283164	0,165938661	5,955713741	2,58939E-09	5,2025E-07	1,983822796

UHMK1	249,738298	373,4573636	217,0970407	649,5588991	691,3646699	739,3691406	541,1150542	1,300742895	0,218486513	5,953424205	2,62589E-09	5,2025E-07	2,463557073
LRRC57	273,5897085	262,748659	264,3357486	585,5265527	483,0573927	633,8726973	415,9301431	1,088710167	0,184093194	5,913907738	3,34085E-09	6,51871E-07	2,126838028
ZNF652	179,5870907	142,6912193	143,7262816	341,7110796	355,5589731	285,1980119	262,1077073	1,076456197	0,182226455	5,90724434	3,47878E-09	6,68652E-07	2,108849584
ROR1	182,393139	288,3346707	221,1173563	570,1341617	491,1382785	535,5285553	419,4884983	1,202835804	0,204690961	5,876350354	4,1941E-09	7,94289E-07	2,301916982
CEP97	119,9585645	90,04307976	107,5434415	242,5840818	241,5286964	219,9332631	169,4705713	1,155361729	0,196908972	5,867491551	4,42438E-09	8,25755E-07	2,227401646
ITPR3	1250,796026	1104,134814	1229,211485	1886,491438	1981,612762	1969,564954	1185,46956	0,704504983	0,120566372	5,843295861	5,1178E-09	9,41529E-07	1,629585426
CORO2B	14,03024146	18,20543143	20,10157784	48,02425984	73,62584796	47,38399571	419,374627	1,693492294	0,290610089	5,82736924	5,63079E-09	1,02131E-06	3,234386965
KIAA0317	697,3030007	431,5179287	678,4282522	1358,22458	1493,168112	1330,32803	1081,650897	1,212594461	0,213216808	5,687142913	1,29182E-08	2,25462E-06	2,317540356
LRRC15	101,0177385	103,3281243	99,50281033	246,8939512	252,3032107	210,9928866	85,25164572	1,224335779	0,215300024	5,686649515	1,29556E-08	2,25462E-06	2,336478528
CCNJ	50,50886927	69,86949359	56,28441796	124,3705191	163,4134674	156,4565896	95,99247248	1,31557796	0,232283338	5,663677702	1,48163E-08	2,50216E-06	2,489020236
ITGA6	1163,808529	866,9721669	1293,536534	2924,554285	2380,269792	2729,49696	2268,901301	1,274382883	0,225527854	5,650667362	1,59826E-08	2,63877E-06	2,418953243
MAPKAPK2	770,2602563	739,0421082	726,6720391	1456,120186	1164,545425	1332,116106	1133,345396	0,822448495	0,14592846	5,635970473	1,74075E-08	2,83765E-06	1,768404725
PMAIP1	378,8165195	231,7502217	400,0213991	709,8970717	806,2928228	785,8590986	523,6752067	1,192639293	0,212211817	5,620041841	1,90911E-08	3,07319E-06	2,285705121
ZFP36L1	1444,413359	780,8653966	1450,328841	3199,770236	3331,120682	2893,999889	2836,51501	1,359720701	0,243286832	5,588961352	2,28432E-08	3,63178E-06	2,566354913
CNOT6	408,9815387	536,3221691	384,9452157	1183,367018	1043,332138	1038,871755	717,3400405	1,293597955	0,231684418	5,58344823	2,35796E-08	3,66619E-06	2,451386481
STK35	202,0354771	201,2438231	210,0614885	373,1115572	353,7632207	354,932949	215,2284476	0,821198955	0,147086963	5,583084579	2,3629E-08	3,66619E-06	1,766873747
NIPA1	127,6751973	147,1195675	123,6247037	304,7693413	283,7288775	235,1319032	217,320145	1,044035863	0,187807932	5,559061614	2,71229E-08	4,10928E-06	2,061987895
PTPRF	675,5561265	742,486379	717,626329	1392,703535	1210,33711	1295,460562	1127,119943	0,868405252	0,156395075	5,552638106	2,8139E-08	4,21366E-06	1,825643727
XPOT	2524,741951	2964,533091	2634,311776	4442,859731	4210,141477	4069,659405	2827,663743	0,646859401	0,117004471	5,528501537	3,22978E-08	4,78081E-06	1,565755993
PDPN	2115,760413	1500,717996	2507,671836	3982,935089	4042,238628	4062,507104	1937,106608	0,982218483	0,178343085	5,507466026	3,64036E-08	5,26747E-06	1,975500872
LAMB3	1153,285848	1555,33429	1294,541613	3194,228975	2831,901518	3187,24424	4468,102422	1,202183921	0,218441239	5,503465944	3,72396E-08	5,32858E-06	2,300877094
CARD10	37,88165195	34,93474679	50,25394461	103,4368673	79,91098133	118,0129704	146,841399	1,317425233	0,241769956	5,449085798	5,06294E-08	7,16489E-06	2,4922093
SKP2	1393,904489	1429,864425	1608,126228	2394,44034	2397,32944	2142,114221	1584,404624	0,646999234	0,118991989	5,437334396	5,40836E-08	7,57053E-06	1,565907761
DHCR24	1945,994491	1658,170376	2001,112074	3297,050147	3683,986027	3130,02583	3107,141566	0,851678913	0,156756209	5,433143087	5,537E-08	7,66725E-06	1,804599777
EPHB2	1258,512659	1170,560037	1436,257737	2213,425822	2124,375077	2084,001774	1004,20505	0,734040132	0,136252583	5,387348382	7,15048E-08	9,55424E-06	1,663290464
GNB4	127,6751973	119,0733623	116,5891515	282,6042983	211,8987819	223,5094137	221,6597377	0,986182719	0,183094492	5,386195432	7,19647E-08	9,55424E-06	1,980936615
TAF9B	336,024283	231,7502217	359,8182434	785,6276353	757,8075083	637,4488479	375,8365842	1,237861491	0,230188099	5,377608551	7,54817E-08	9,86661E-06	2,358486742
NUFIP2	330,4121865	393,1389111	329,6658766	690,8105069	742,543613	690,1970696	771,4501338	1,009183585	0,188231771	5,361388135	8,25849E-08	1,053E-05	2,012771759
FST	415,9966594	520,0848924	445,2499492	946,9398927	852,982385	889,5674666	339,4508361	0,95997668	0,179139719	5,358815366	8,37694E-08	1,05763E-05	1,945278451
ARL5B	272,1866844	218,9572158	229,1579874	475,9327289	472,2828784	447,9128651	434,1035868	0,955566695	0,17845818	5,354569311	8,57604E-08	1,07226E-05	1,939341263
IRAK2	198,5279167	140,2310259	206,0411729	413,1317738	365,4356112	400,5288694	192,0950177	1,121744217	0,209575351	5,352462555	8,67653E-08	1,07439E-05	2,176099037
EIF4EBP2	611,7185278	926,5088481	558,8238641	1547,243141	1538,959798	1611,949892	1541,989431	1,16111305	0,217160417	5,346798779	8,95234E-08	1,09798E-05	2,236298932
PLCB4	527,537079	601,2712758	518,6207084	1417,331361	1443,784921	1444,76485	682,8397405	1,385410992	0,259947589	5,329578148	9,84412E-08	1,18479E-05	2,612463702
IL15RA	22,44838634	23,1258183	41,20823458	84,35030254	87,99186708	96,55606673	61,13751449	1,665527101	0,312858907	5,323572582	1,01749E-07	1,21326E-05	3,17229536
ROBO4	161,3477768	112,1848207	156,7923072	296,765298	338,4993254	245,8603551	383,0304852	1,042769584	0,197229138	5,287097	1,24273E-07	1,45489E-05	2,060178845
CDK6	470,013089	497,4511128	429,168687	1096,553933	1042,434262	1088,043826	655,3511723	1,20731823	0,231843721	5,207465734	1,91437E-07	2,22102E-05	2,309080117
ANXA2	8072,299426	6906,255014	6073,691745	11717,9194	12170,71182	12209,87225	14085,86744	0,777788785	0,150034899	5,184052448	2,17116E-07	2,47435E-05	1,714501047
PSMD4	87,68900915	103,820163	95,48249476	249,3567338	168,8007246	221,7213384	148,7846889	1,157375344	0,223422094	5,180218859	2,21626E-07	2,50359E-05	2,230512671
TDG	401,9664179	276,0337035	353,7877701	589,8364221	635,6963458	670,5282412	477,4781151	0,880544108	0,17005007	5,178146095	2,24102E-07	2,50955E-05	1,841069525
ABHD2	1814,811733	829,085188	1676,471592	3875,188352	3456,82335	3545,753339	2697,305658	1,33315084	0,260952253	5,108792215	3,24225E-07	3,56869E-05	2,519523374
LCLAT1	264,4700516	234,2104151	180,9142006	450,6892077	610,5558124	473,8399571	356,9871129	1,171059995	0,229304522	5,107007854	3,273E-07	3,57201E-05	2,251770811
PCGF5	136,0933422	154,9921865	131,6653349	266,5962117	287,3203823	244,9663174	190,5002472	0,91240903	0,178977853	5,097887886	3,43464E-07	3,71692E-05	1,882185773
ELMOD2	125,5706611	86,10677027	135,6856504	277,0630375	253,2010869	271,7874471	185,3663138	1,218121675	0,239154925	5,093441727	3,51621E-07	3,77348E-05	2,326436283
CDH5	17,53780183	15,74523799	17,08634117	67,72652029	63,74920982	54,53629695	19,69845067	1,892042599	0,371814559	5,088672713	3,60578E-07	3,83763E-05	3,711603498
RC3H2	357,7711573	366,0767833	304,5389043	819,4908955	622,2282029	784,965061	574,9157954	1,113176321	0,219248786	5,077229121	3,82979E-07	4,04263E-05	2,163213891
INPP4B	75,06179183	53,63221691	93,47233697	229,0387777	149,9453245	186,8538699	157,1825897	1,368194883	0,269574765	5,075381894	3,86719E-07	4,04891E-05	2,58147367
RAB3B	312,1728726	253,3999239	332,6811133	570,1341617	649,1644888	492,6147478	457,4020341	0,934637038	0,184494084	5,065945844	4,06377E-07	4,18666E-05	1,91140969
UBE2V2	1256,408123	1151,370528	1172,927067	1900,652438	2403,614573	2162,677087	1853,02205	0,852516972	0,168238405	5,06731488	4,03467E-07	4,18666E-05	1,805648371
PARP2	182,393139	174,673734	177,8989639	399,5864697	359,1504779	382,6481163	243,801534	1,09409209	0,218895711	4,998234484	5,78576E-07	5,77589E-05	2,134786939
TTL	357,7711573	352,7917387	404,0417147	666,798377	639,2878506	573,0781368	779,2758363	0,757221363	0,151552058	4,996443959	5,83971E-07	5,78491E-05	1,690232093

ATF4	1447,920919	1245,349917	1515,658969	2124,149954	2405,410326	2085,789849	2385,591142	0,653500155	0,130872291	4,993418761	5,93197E-07	5,78727E-05	1,572979812
GNPNAT1	1014,386458	626,3652488	952,8147898	1578,027923	1941,208333	1998,174159	1579,318414	1,090179408	0,218703835	4,984729264	6,20486E-07	5,96315E-05	2,129005103
YAP1	1578,402165	978,6649489	1272,429878	2340,259124	2879,488956	2563,205957	3257,776176	1,023588048	0,205542431	4,979935481	6,36055E-07	6,06749E-05	2,032968762
LAMA5	298,1426311	230,2741056	282,4271687	475,9327289	474,976507	477,4161077	415,7754234	0,820841893	0,165551792	4,958218118	7,11427E-07	6,68741E-05	1,766436506
OSTM1	166,9598734	110,7087046	138,7008871	333,7070363	417,5124305	300,396652	306,0912466	1,339091104	0,270678569	4,947163376	7,53028E-07	7,02717E-05	2,529918841
MMP3	162,750801	130,3902521	186,9446739	329,3971669	318,7460491	364,7673632	127,8206156	1,084384114	0,220206803	4,924389697	8,4624E-07	7,8402E-05	2,120470062
ARNTL2	253,9473705	224,3696414	297,5033521	530,1139452	490,2404023	482,7803336	865,7601154	0,959589946	0,19568172	4,903830299	9,39857E-07	8,58403E-05	1,944757063
CLDN11	239,917129	174,673734	287,4525632	511,643076	553,0917359	495,2968608	188,6200588	1,157963852	0,236795937	4,890133947	1,00767E-06	9,0747E-05	2,231422734
SHANK1	28,06048293	24,60193436	20,10157784	66,49512901	75,42160035	57,21840991	639,2781341	1,439210097	0,294298572	4,890306082	1,00679E-06	9,0747E-05	2,711723527
HHEX	192,214308	208,1323647	174,8837272	431,6026429	318,7460491	472,0518818	171,6555146	1,085079859	0,223226151	4,860899381	1,16854E-06	0,000103071	2,121492913
CD274	378,1150074	410,8523038	468,3667638	670,4925508	701,241308	698,2434085	357,4965766	0,721631224	0,148805733	4,849485365	1,23782E-06	0,000107707	1,64904552
DENND1B	37,18013988	44,28348185	35,17776123	78,80904179	85,2982385	80,46338894	124,5372784	1,047556365	0,216265064	4,843853867	1,27345E-06	0,00011001	2,067025755
MMP9	16,13477768	9,348735057	18,09142006	48,02425984	41,30230496	45,5959204	50,06276006	1,683319926	0,349885865	4,811054394	1,50136E-06	0,000125466	3,211661681
DOCK9	587,1656052	613,572243	573,9000474	944,4771102	953,5445188	858,2761487	620,2851985	0,634459689	0,132056518	4,804455694	1,55173E-06	0,000127281	1,55235626
PDLIM2	4,209072439	10,33281243	7,035552245	28,93769503	22,44690487	30,39728027	88,89578814	1,874288049	0,391373745	4,788997915	1,67616E-06	0,000136618	3,666206502
ANXA3	420,2057318	384,2822147	462,3362904	685,8849418	829,6376039	651,7534504	897,5267277	0,776946322	0,16234334	4,78582197	1,70289E-06	0,000137923	1,713500155
NAV2	136,7948543	136,2947164	129,6551771	326,3186887	247,8138297	261,9530329	103,2358582	1,054946573	0,220673561	4,780575299	1,74794E-06	0,000139953	2,077641264
PPIP5K2	92,59959366	61,5048359	91,46217919	275,2159506	203,8178962	261,9530329	173,9653909	1,600405232	0,334786509	4,780375523	1,74968E-06	0,000139953	3,032284738
GHITM	1398,113562	1272,412045	1420,176475	2084,129738	2111,80481	1969,564954	1633,30233	0,592793259	0,124065617	4,778062406	1,76992E-06	0,000140698	1,508163939
KRT18	3882,167813	3320,277061	4162,031693	7143,916499	7642,722169	6659,686491	4602,870874	0,916459743	0,193640359	4,732793036	2,21451E-06	0,000171798	1,887477888
CORO1A	37,88165195	30,01435992	39,1980768	77,57765051	77,21735274	92,08587845	55,07028142	1,21908753	0,258634362	4,713555934	2,43431E-06	0,000187719	2,327994307
C10orf12	261,6640033	246,5113823	250,2646442	400,2021653	511,789431	449,7009404	381,2038481	0,842911385	0,179130661	4,705567314	2,53161E-06	0,000192165	1,793666143
ETHE1	96,8086661	55,60037166	94,47741587	203,1795609	242,4265726	175,2313804	270,2320778	1,341197487	0,285048539	4,705154747	2,53673E-06	0,000192165	2,533615304
DGKH	166,2583613	116,6131689	180,9142006	278,2944288	360,0483541	362,0852502	229,6618496	1,115082925	0,238447765	4,676424315	2,9192E-06	0,000216055	2,166074592
PTAR1	148,0190474	145,6434514	124,6297826	269,6746899	246,9159535	262,8470705	213,492377	0,893695581	0,19134028	4,670713251	3,00156E-06	0,00022088	1,857929271
DDX52	1346,90318	812,8479113	1148,805174	1953,602262	2839,084528	1890,88964	1213,05668	1,01506078	0,21744167	4,668198045	3,03853E-06	0,000222331	2,020988035
AMPD3	451,7737751	330,6499978	474,3972371	724,6737671	813,4758324	761,720082	430,5807865	0,875160129	0,18791605	4,657186698	3,2056E-06	0,00023192	1,83421166
ZCCHC24	213,9611823	259,7964269	155,7872283	419,2887301	448,9380973	451,4890157	309,189786	1,059911196	0,228370374	4,64119394	3,46402E-06	0,000245108	2,084803189
PIK3CD	737,990701	449,7233601	761,8498003	1324,977015	1102,591967	1438,506587	835,4660406	0,990427211	0,213933501	4,629603162	3,66367E-06	0,000253932	1,986773227
HMGA2	3365,854927	4079,492756	3704,720797	6387,226559	9178,090462	7743,260129	4877,473187	1,063653558	0,230043518	4,623705851	3,76944E-06	0,000254151	2,090218206
SOX7	105,928323	138,2628711	81,41139027	264,1334291	197,5327628	266,4232212	157,1039209	1,149628165	0,248607172	4,624275936	3,75909E-06	0,000254151	2,218567065
PER2	26,65745878	34,44270811	27,13713009	80,04043307	70,03434319	58,11244757	49,27961499	1,228044222	0,267439053	4,591865727	4,39301E-06	0,000294652	2,342492165
HS2ST1	540,1642963	669,1726146	559,8289429	1171,668801	1192,379587	945,8918389	726,9577375	0,902736436	0,196881503	4,585176484	4,53603E-06	0,000302654	1,869608808
TGFBR1	269,3806361	355,2519322	228,1529085	609,5386826	477,6701356	598,1111911	386,8657567	0,977763523	0,213444106	4,580887903	4,63006E-06	0,000304214	1,969410046
PLAGL1	14,73175354	14,26912193	6,030473353	30,78478195	40,40442876	30,39728027	285,1056414	1,456917564	0,3196576	4,557744175	5,1706E-06	0,000336298	2,745211995
PLAU	1977,562534	1681,788233	1658,380172	2540,360206	2873,203823	2789,397483	4044,41712	0,624904727	0,137290684	4,551690691	5,32165E-06	0,000344383	1,542108984
LAMB1	749,2148941	272,5894327	587,9711519	1516,458359	1336,937654	1422,413909	948,5286577	1,411132201	0,310345944	4,546965181	5,4425E-06	0,000350443	2,659457904
EMP1	3997,215793	2407,545297	4314,803684	7007,232067	7488,287464	6961,871219	5475,485053	1,001719869	0,220731005	4,538192846	5,67384E-06	0,000359939	2,002385667
ADAMTS6	1302,006408	1394,929678	1400,074897	2099,522129	1898,110276	2086,683886	980,6423389	0,570822659	0,126328555	4,518556085	6,22628E-06	0,000388429	1,485370323
NXPE3	178,8855787	101,8520083	149,7567549	328,7814712	288,2182585	285,1980119	380,9151235	1,074636978	0,237817134	4,518753384	6,22048E-06	0,000388429	2,106192028
THY1	2670,656463	2688,991426	2714,718088	4175,647824	4321,478125	4021,381372	1716,259801	0,632677035	0,139975506	4,51991246	6,18652E-06	0,000388429	1,550439289
HLA-C	1088,045225	461,5322886	1156,845805	2866,678895	2035,485333	2560,523844	873,2822089	1,464736907	0,324365301	4,515701591	6,31075E-06	0,00039072	2,760131318
LAMC2	929,503497	1536,144782	930,7030542	2563,140945	2265,341639	2290,524472	2296,186238	1,066458389	0,237117374	4,497596986	6,87258E-06	0,000423469	2,094285878
NLRP3	47,0013089	36,90290154	61,30981242	97,27991096	111,3366481	103,708368	147,2352672	1,129172177	0,251945855	4,481804938	7,40144E-06	0,000447492	2,187331945
FBXL17	68,74818317	57,56852641	55,27933907	114,5193888	115,8260291	125,1652717	85,3043646	0,96529723	0,21560488	4,477158551	7,56431E-06	0,000453382	1,952465735
SDF2L1	89,09203329	61,5048359	79,40123248	155,7709967	171,4943532	162,7148532	173,8752846	1,097726454	0,245191409	4,477018428	7,56927E-06	0,000453382	2,140171566
DCP2	310,7698484	124,9778266	259,3103542	488,2466417	736,2584796	578,4423627	485,860211	1,378536249	0,308362953	4,470498924	7,80373E-06	0,000465261	2,600044381
SERTAD4	16,83628976	23,1258183	13,0660256	54,79691187	54,77044788	35,7615062	90,06838424	1,416687266	0,318215413	4,451975632	8,50838E-06	0,00049805	2,669717826
SLC9A6	183,7961632	310,9684503	189,9599106	522,1099019	511,789431	475,6280324	358,4781059	1,13512995	0,255490147	4,442950008	8,87338E-06	0,000517065	2,196383456
CAB39	371,8013988	396,0911432	399,0163202	623,0839866	633,0027173	614,2038689	612,4225173	0,680815364	0,153726827	4,428734911	9,47874E-06	0,000544943	1,603045488

SCO1	252,5443463	202,2279005	251,269723	444,5322513	379,8016304	474,7339947	284,67842	0,883479378	0,199881919	4,420006492	9,86979E-06	0,000564903	1,844819129
LFNG	63,83759866	25,09397305	51,2590235	127,4489973	115,8260291	108,1785562	435,6591728	1,34695544	0,305100415	4,41479386	1,01106E-05	0,000573589	2,543747444
CENPQ	312,1728726	364,1086286	292,4779576	586,7579439	485,7510213	691,0911072	379,9529488	0,861385289	0,195683803	4,401924311	1,07295E-05	0,000603382	1,816781964
VPS36	98,91320232	64,94910671	108,5485204	172,3947789	184,0646199	204,734623	235,4119934	1,056081882	0,239902283	4,402133526	1,07192E-05	0,000603382	2,079276879
TJP2	220,976303	215,0209063	285,4424054	377,4214267	372,6186208	461,3234299	714,2624996	0,753800993	0,171757611	4,388748697	1,14005E-05	0,000637745	1,686229604
TMPPE	27,35897085	13,77708324	14,07110449	54,18121623	56,56620027	50,06610867	39,74134987	1,54227254	0,351474763	4,388003642	1,14396E-05	0,000637745	2,912529253
PHF20L1	304,4562398	261,7645816	225,1376719	403,8963392	457,0189831	405,8930953	557,5858583	0,673718662	0,153581963	4,386704334	1,15081E-05	0,000638799	1,595179375
SERINC2	787,0965461	678,5213497	923,6675019	1244,936582	1322,571635	1291,884411	1994,464184	0,694238644	0,158356283	4,384029686	1,16504E-05	0,00064117	1,618030321
ATP13A3	1779,73613	1823,987414	1558,877362	2329,176602	2524,827859	2444,298948	1968,085943	0,49798923	0,113799072	4,376039453	1,20855E-05	0,00065948	1,412243861
UBXN2A	129,7797335	129,4061747	133,6754927	310,310602	231,6520582	278,9397483	231,4580827	1,065527135	0,243559125	4,374819203	1,21533E-05	0,000660381	2,092934461
TMBIM6	12026,72298	11275,06652	13481,12318	22350,98309	20548,79459	20432,33656	15189,60168	0,783996671	0,179644602	4,36415378	1,27616E-05	0,000690519	1,721894403
ATP6AP2	898,6369657	915,6839969	778,9361414	1588,494749	1412,359254	1320,493616	1019,468787	0,736053236	0,168951507	4,356594676	1,32102E-05	0,000706294	1,665613002
IGFBP7	423,7132922	228,7979896	383,9401368	757,3056359	763,1947655	718,8062745	387,7708307	1,114516506	0,256576218	4,343802854	1,40037E-05	0,000745206	2,165224333
FMNL1	117,1525162	71,34560965	137,6958082	248,7410381	235,243563	213,6749995	256,7525589	1,111636553	0,256618563	4,331863363	1,47853E-05	0,000780347	2,160906355
CDK17	828,4857584	690,3302782	816,1240605	1344,06358	1134,017634	1278,473846	829,3364395	0,687759297	0,159436402	4,313690524	1,60552E-05	0,000843912	1,610779813
TMPRSS2	51,91189341	49,20386872	60,30473353	121,2920409	120,3154101	109,9666316	44,56267993	1,13288336	0,262861772	4,309806448	1,63397E-05	0,000855379	2,192965871
IL32	86,285985	74,29784177	57,28949685	124,3705191	149,0474483	127,8473846	233,7412802	0,865173318	0,200875393	4,307014933	1,65472E-05	0,000862734	1,821558487
NRIP3	206,9460616	175,1657727	235,1884608	403,2806435	344,7844588	330,7939323	727,4460832	0,813815086	0,189913502	4,285188127	1,82585E-05	0,000936783	1,757853799
PRSS23	297,441119	511,228196	372,884269	744,3760275	672,5092698	873,4747888	900,6738552	0,952095763	0,222750363	4,274272559	1,91762E-05	0,000976093	1,934681075
EPHX4	77,86784012	70,85357096	96,48757365	147,1512577	156,2304579	156,4565896	103,8886302	0,919600046	0,215454663	4,268183529	1,97071E-05	0,000993379	1,891590821
STAT3	730,2740682	705,0914388	642,2454121	1305,274755	1382,72934	1270,427508	1186,189885	0,929451301	0,217785894	4,26772957	1,97473E-05	0,000993379	1,904551502
PPL	54,01642963	51,66406216	37,18791901	113,9036932	96,97062903	137,6817989	41,84381353	1,274603931	0,299002189	4,262858197	2,01829E-05	0,001011342	2,4193239
REXO2	584,359557	429,0577353	605,0574931	1002,3525	805,3949466	972,7129685	850,4274352	0,784085422	0,184118534	4,258590398	2,0572E-05	0,001022881	1,722000334
NGF	16,13477768	14,76116062	15,07618338	43,71439037	55,66832407	50,96014633	16,64012112	1,706974874	0,401460438	4,25191305	2,11952E-05	0,001045793	3,264755324
ADAMTS12	972,2957334	1063,295603	964,8757365	1588,494749	1379,137835	1729,068825	840,4301033	0,645857129	0,151993717	4,249235701	2,14501E-05	0,001054021	1,564668605
PIK3R1	274,2912206	234,2104151	242,224013	536,2709015	480,3637642	536,4225929	334,3870225	1,049156356	0,246950508	4,248447849	2,15257E-05	0,001054021	2,069319419
SDC4	5696,979546	6404,375553	5748,046184	9663,958749	8809,961222	9336,43523	4749,50107	0,639654618	0,150698409	4,244600996	2,18983E-05	0,001065911	1,557956139
FAM46A	202,0354771	185,9906238	158,802465	277,6787332	327,7248111	342,4164218	237,5979604	0,787077172	0,18552323	4,242472353	2,21071E-05	0,001070282	1,725574991
KLF2	81,37540049	86,10677027	86,43678473	153,3082141	173,2901056	183,2777193	114,3226874	1,004030605	0,237510694	4,227306941	2,36505E-05	0,001132234	2,005595417
FRMD6	717,6468509	416,7567681	772,9056681	1433,955143	1252,537292	1378,606064	939,2946041	1,094010736	0,259581815	4,214512235	2,50319E-05	0,001176497	2,134666561
MGLL	402,66793	346,8872745	427,1585292	576,2911181	756,0117559	616,8859819	764,1803503	0,729855947	0,173418742	4,208633612	2,5692E-05	0,001198772	1,658473485
CDA	1022,804603	794,6424799	1073,424257	1680,233399	1749,062827	1576,188386	658,3102018	0,79340608	0,188802318	4,202311117	2,64204E-05	0,001228309	1,733161483
MBP	35,07560366	33,95066942	32,16252455	67,11082465	52,07681929	70,62897474	165,5144344	0,90735392	0,215991858	4,200870937	2,6589E-05	0,001231703	1,875602257
C16orf74	242,7231773	265,2088524	294,4881154	603,3817262	590,8025361	556,985459	367,7822416	1,127131796	0,268445307	4,198739056	2,68405E-05	0,001238898	2,184240622
ARHGDIB	1740,451454	1508,098576	2065,437123	2824,811592	2603,840965	2688,371228	2069,51917	0,61273442	0,146047054	4,195458942	2,7232E-05	0,001252476	1,529154756
ZC3HAV1L	4,209072439	3,444270811	2,010157784	16,00808661	17,0596477	8,940376549	67,82308783	2,078017351	0,496106995	4,188647551	2,80622E-05	0,001281506	4,22226565
USB1	310,7698484	252,4158466	373,8893479	615,0799433	514,4830596	637,4488479	713,1523487	0,919750242	0,219709849	4,186203962	2,83658E-05	0,001290796	1,891787761
EFHD2	1551,744706	527,9575114	1476,460893	3409,106753	4621,368774	3437,574783	3556,551908	1,689850239	0,403862813	4,184218462	2,86149E-05	0,001292042	3,226232117
SFMBT1	143,1084629	112,6768594	131,6653349	202,5638652	205,6136486	225,297489	191,5389921	0,713877543	0,170986908	4,175042123	2,97931E-05	0,001336848	1,640206595
RPS2	16477,11557	18819,98775	18287,41044	28217,94683	25575,10553	25777,7877	25151,91604	0,570439585	0,137061298	4,161930417	3,15569E-05	0,001401342	1,484975969
GMFG	53,31491756	38,3790176	30,15236677	108,3624325	106,8472672	92,08587845	36,46018386	1,326769998	0,320300381	4,142267942	3,43888E-05	0,001516641	2,508404474
FGD6	126,2721732	127,43802	98,49773143	235,1957341	187,6561247	229,7676773	197,5794046	0,883700988	0,213562542	4,137902545	3,50495E-05	0,001540504	1,845102531
TNRC6A	325,501602	339,9987329	307,554141	526,4197713	569,2535074	548,0450824	386,6944648	0,754417162	0,183438108	4,112652317	3,91139E-05	0,00169599	1,686949939
SPP1	14,03024146	9,840773745	20,10157784	22,78073864	34,1192954	49,17207102	676,0097717	1,316021202	0,320468167	4,106558274	4,01598E-05	0,001726892	2,489785061
SEPT11	1249,393002	1306,362715	1351,83111	2290,387777	2079,481267	1962,412652	2281,384469	0,696898885	0,169974774	4,1000136	4,13126E-05	0,001761667	1,621016622
HIST1H2AL	1204,49623	1331,456688	1391,029187	2090,90239	1802,935399	1977,611293	2113,953145	0,581007457	0,141828277	4,096555843	4,19342E-05	0,001776412	1,495893491
VPS33A	380,2195437	312,4445664	365,8487168	754,2271577	653,6538697	812,6802283	405,5950314	1,069928916	0,261834301	4,086282475	4,3834E-05	0,001838744	2,099329927
SRGN	1715,898531	3146,095366	1692,552854	4266,770778	4296,337592	4418,33409	2118,011051	0,98503211	0,241379859	4,080838044	4,48736E-05	0,001876242	1,979357367
ANKRD10	39,2846761	47,72775266	45,22855015	86,19738946	78,11522894	77,78127597	104,29572	0,872289627	0,214731648	4,062231318	4,86059E-05	0,002006239	1,830565793
RIN2	1505,444909	1141,037716	1942,817499	2597,619901	2823,820632	2521,186187	1539,030304	0,792849854	0,195142757	4,062922276	4,84622E-05	0,002006239	1,732493397

COL17A1	120,6600766	127,43802	103,5231259	206,2580391	186,7582485	233,3438279	373,1958131	0,825817486	0,203704763	4,053992035	5,03509E-05	0,00206503	1,772539142
COMMD8	119,2570524	88,07492501	98,49773143	267,827603	215,4902867	294,1383885	234,7533311	1,34737182	0,335410472	4,017083347	5,89229E-05	0,002338242	2,544481709
DPP4	77,16632805	133,3424842	68,34536467	202,5638652	200,2263914	210,0988489	238,8325291	1,120894159	0,279055989	4,016735715	5,90098E-05	0,002338242	2,174817225
L1CAM	80,67388841	58,06056509	68,34536467	145,9198664	127,4984196	158,2446649	53,44006971	1,064328705	0,265919623	4,002445146	6,26912E-05	0,002455122	2,091196606
PAWR	244,1262015	298,6674832	225,1376719	487,0152504	488,4446499	551,6212331	371,984813	0,988462557	0,247016172	4,001610709	6,29127E-05	0,002455122	1,984069493
PITHD1	531,0446394	361,6484351	463,3413693	850,2756774	699,4455557	794,7994752	497,6240691	0,792324494	0,197978055	4,002082416	6,27874E-05	0,002455122	1,731862622
PABPC1	12823,6407	9881,612956	13775,6113	21680,49054	19915,79187	19701,9078	21615,21398	0,748844742	0,187231865	3,999558202	6,34608E-05	0,002469029	1,680446651
ATG2B	347,2484762	331,1420365	324,6404822	509,7959891	441,7550878	526,5881787	434,1590102	0,559507328	0,140331005	3,987054225	6,68987E-05	0,002564053	1,473765849
FRS2	246,9322498	120,5494784	213,0767251	503,6390327	387,8825161	476,5220701	315,9052396	1,240943218	0,31166814	3,981617172	6,8448E-05	0,002607908	2,363530067
METRN	14,03024146	12,79300587	15,07618338	41,25160781	30,52779062	46,48995805	32,99712477	1,510948378	0,379415838	3,982301808	6,8251E-05	0,002607908	2,84997325
PM20D2	35,77711573	23,1258183	32,16252455	74,49917232	61,05558124	107,2845186	125,7110106	1,424939087	0,358370581	3,976160886	7,00368E-05	0,00266057	2,685031636
LGALS3	589,9716535	681,9656205	539,7273651	1119,334672	870,9399088	1108,606692	500,3612977	0,773130689	0,194726897	3,970333324	7,17722E-05	0,002718476	1,708974285
PARD6B	108,0328593	128,9141361	79,40123248	272,1374724	211,8987819	239,6020915	172,6691595	1,186658595	0,298957568	3,969321139	7,20777E-05	0,002722042	2,276249334
FLI1	491,7599633	416,7567681	450,2753437	805,9455914	695,8540509	782,282948	348,2047301	0,750321738	0,189883084	3,951493315	7,7665E-05	0,002907472	1,682167932
AJUBA	351,4575487	257,8282721	432,1839236	560,2830315	600,6791742	683,0447683	644,1412818	0,829146261	0,209988896	3,948524315	7,86344E-05	0,002928564	1,776633699
DDIT4	426,5193405	582,081767	517,6156295	814,5653304	862,8590231	746,5214418	1009,659699	0,666283181	0,168788932	3,947434075	7,89933E-05	0,002931629	1,586979157
PDHX	425,8178284	177,6259661	360,8233223	778,8549833	832,3312325	738,4751029	505,6616949	1,28747552	0,32688295	3,938643843	8,19435E-05	0,003015051	2,441005456
DUSP22	43,49374854	48,21979135	53,26918129	92,97004149	94,27700044	97,45010438	111,9505657	0,978755575	0,248632124	3,936561216	8,26575E-05	0,00303266	1,970764754
IL6ST	53,31491756	14,26912193	61,30981242	153,9239097	124,8047911	154,6685143	112,0975716	1,77031915	0,450072071	3,933412586	8,37483E-05	0,003063951	3,411294124
GAS6	277,798781	145,6434514	289,462721	465,4659031	529,7469549	437,1844132	249,6056196	1,012688971	0,257721054	3,92939946	8,51583E-05	0,003106709	2,017668233
KLHL15	52,61340549	72,82172571	37,18791901	112,6723019	107,7451434	106,3904809	79,85249971	0,978046457	0,249308503	3,923036897	8,74398E-05	0,003166519	1,969796316
SIK1	79,97237634	83,15453814	65,33012799	146,5355621	129,294172	131,4235353	86,11855185	0,825499878	0,210487901	3,921840034	8,78753E-05	0,003169912	1,772148962
CYR61	3380,586681	1828,415762	3911,767048	6622,422293	8548,67925	7189,850821	4170,754602	1,294070268	0,330226953	3,918729999	8,90168E-05	0,003202117	2,452189154
RGCC	67,34515902	46,2516366	71,36060135	162,5436487	120,3154101	116,2248951	76,40262205	1,1261957	0,287829486	3,912718313	9,1263E-05	0,003273773	2,182823832
KIAA1033	65,94213488	123,0096718	80,40631138	195,1755176	167,0049722	174,3373427	134,3979557	0,983245953	0,252547509	3,893310836	9,88853E-05	0,003517804	1,976908301
CDKN1C	113,6449559	72,32968702	136,6907293	257,3607771	198,430639	242,2842045	97,42464328	1,125357343	0,289755561	3,883816203	0,00010283	0,003628055	2,181555751
FAM63B	65,24062281	96,4395827	56,28441796	251,8195163	159,8219627	201,1584723	121,728294	1,484532895	0,382504268	3,88108845	0,00010399	0,003635795	2,798265583
MTMR9	112,2419317	78,72618996	99,50281033	171,7790833	216,3881629	146,6221754	126,2017616	0,886114191	0,22838052	3,879990258	0,000104461	0,003635795	1,848191426
RAB43	267,2760999	206,6562486	273,3814587	440,8380775	349,2738397	440,7605639	362,1667684	0,725870844	0,187012919	3,881394119	0,000103859	0,003635795	1,653898666
ZCCHC10	51,91189341	58,06056509	44,22347126	107,1310412	91,58337186	110,8606692	89,82757004	0,994189434	0,256194367	3,880606146	0,000104196	0,003635795	1,991961045
RHOF	1353,918301	1600,109811	1618,177016	2380,895036	2301,256687	2175,193614	1873,188028	0,585062398	0,150859002	3,878206738	0,000105229	0,003652676	1,500103869
RBMS2	515,6113738	682,4576592	563,8492585	1041,757021	895,1825661	849,3357721	955,0344664	0,660241227	0,17043999	3,873745975	0,000107175	0,003690378	1,580346844
NAA30	319,1879933	263,7327364	320,6201666	613,2328564	597,9855457	582,0185133	462,6572315	0,990564401	0,255794817	3,87249598	0,000107726	0,00369947	1,986962164
SLC7A6	128,3767094	85,12269289	120,6094671	182,2459091	188,5540009	227,0855643	261,3860765	0,847115343	0,218790978	3,871801982	0,000108034	0,003700154	1,798900441
YOD1	140,3024146	215,0209063	114,5789937	316,4675584	309,7672872	352,250836	295,7553129	1,048995101	0,271495378	3,863767806	0,000111651	0,003803826	2,069088137
SFXN1	1786,75125	1335,392997	1694,563012	2370,42821	2707,096727	2311,087338	1773,915176	0,617986252	0,160075224	3,860599017	0,000113109	0,003833218	1,534731471
MINK1	399,8618817	397,0752206	371,8791901	684,0378549	540,5214692	582,912551	526,7745971	0,62943058	0,163146423	3,858071599	0,000114285	0,003852787	1,546954303
MARS2	68,74818317	53,14017822	62,31489132	113,2879976	125,7026673	99,23817969	89,89405736	0,883714777	0,229531273	3,850084408	0,000118077	0,003970228	1,845120166
ITGA2	2108,04378	1533,192549	1858,390872	3293,355973	3189,256244	3494,793193	1523,919285	0,859601096	0,223321311	3,849167331	0,00011852	0,003974742	1,814536523
ETS1	30,86653122	20,17358618	25,1269723	64,03234645	51,1789431	89,40376549	24,57325984	1,432228586	0,372288505	3,847093226	0,000119527	0,003998117	2,698632616
BCAP31	119,2570524	98,40773745	130,660256	203,1795609	210,1030296	172,5492674	227,1666936	0,760993961	0,198153494	3,84042665	0,000122821	0,004066027	1,694657776
COL13A1	75,06179183	88,07492501	53,26918129	157,6180836	121,2132863	151,9864013	71,62691267	0,978918025	0,255075946	3,837751227	0,000124166	0,004096713	1,970986677
AXL	1008,072849	1343,265616	1047,292206	1948,061002	1887,335761	2353,107108	2636,311925	0,863823839	0,225303444	3,834046314	0,000126052	0,004124007	1,81985542
DUSP5	346,5469641	536,8142078	358,8131645	668,6454639	788,3352989	692,8791825	502,2727831	0,787066402	0,205434889	3,831220709	0,000127509	0,004157118	1,725562108
MCFD2	1846,379777	1323,584069	2044,330467	2875,298634	2851,654794	2955,688487	2329,037752	0,736713621	0,193170525	3,813799336	0,000136847	0,004427916	1,666375601
STK17B	120,6600766	106,7723951	114,5789937	205,0266478	204,7157724	194,9002088	239,7593244	0,824269512	0,216862098	3,80089245	0,000144176	0,004630167	1,770638273
WASF3	69,44969524	103,3281243	95,48249476	173,6261702	152,6389531	202,9465477	73,90080875	0,978342838	0,257505769	3,799304548	0,000145103	0,00464834	1,970201023
FAM20B	260,9624912	168,277231	238,2036974	419,2887301	325,9290587	429,1380743	371,5447636	0,820579482	0,216130402	3,796686971	0,000146643	0,004662878	1,766115239
C15orf52	185,1991873	256,352156	193,9802262	352,1779055	359,1504779	300,396652	271,1821219	0,664572994	0,175594711	3,784698233	0,000153895	0,004857506	1,58509905
SERPINE1	2841,123896	980,141065	2753,916165	4971,126589	5878,395447	5003,034717	2243,600755	1,270114183	0,336284769	3,776900713	0,000158792	0,004975483	2,411806531

LAPTM5	4060,35188	2795,763821	3472,547573	5482,769665	5578,504798	5824,655322	3749,089978	0,709373316	0,188427273	3,764706169	0,000166745	0,005211997	1,635093704
ZNF121	449,6692389	358,2041643	416,1026614	600,9189436	564,7641265	614,2038689	481,4320874	0,542935414	0,144380327	3,760452858	0,000169606	0,005253789	1,456933889
C21orf7	75,7633039	132,3584069	101,5129681	192,0970394	185,8603723	201,1584723	152,771516	0,895899766	0,239268478	3,744328445	0,000180877	0,005532857	1,860770031
NOTCH2	3243,090314	2694,403851	3074,536331	4256,303952	4379,840078	4033,897899	3636,781198	0,491885554	0,131465812	3,741547302	0,000182891	0,005570938	1,406281638
TENM2	41,38921232	28,53824386	28,14220898	58,4910857	66,44283841	57,21840991	145,0198498	0,889587891	0,238198035	3,734656713	0,000187971	0,005695755	1,852646836
PPP2R5C	148,7205595	62,98095197	120,6094671	268,4432986	251,4053345	239,6020915	132,113663	1,199698911	0,321896948	3,72696578	0,000193799	0,005844825	2,296917296
SLC35G1	61,73306244	72,32968702	57,28949685	128,0646929	140,0686864	118,9070081	92,98871322	1,008097047	0,270447926	3,727508881	0,000193382	0,005844825	2,011256449
SMAD5	834,097855	567,3206064	837,2307172	1371,769884	1203,154101	1242,71234	1080,529801	0,772129079	0,207498447	3,721131854	0,000198332	0,005939808	1,707788219
EHF	16,83628976	28,53824386	17,08634117	50,4870424	56,56620027	68,84089943	20,28005142	1,46568379	0,394279082	3,717376488	0,000201302	0,005959056	2,761943467
FAM82A2	163,452313	136,7867551	221,1173563	328,7814712	333,1120682	272,6814847	173,1907662	0,851788901	0,229103943	3,717914626	0,000200874	0,005959056	1,804737361
NFIA	150,1235837	173,1976179	143,7262816	239,5056036	240,6308202	233,3438279	200,1008386	0,604192219	0,16261874	3,71539109	0,00020289	0,005959056	1,520127379
NBN	320,5910174	532,3858596	288,4576421	818,8751998	696,7519271	748,3095171	786,2019891	0,984415538	0,265197835	3,712004426	0,000205624	0,006004605	1,97851162
AHNAK	1226,243104	1019,50416	1299,567008	1573,718053	1797,548142	1740,691314	2140,004212	0,529175678	0,142778873	3,70626038	0,000210342	0,006087157	1,443104404
SLC35D1	97,51017817	93,48735057	85,43170584	137,3001275	162,5155912	148,4102507	115,6862471	0,689514556	0,186126819	3,704541662	0,000211773	0,006114835	1,612740766
KLHL28	42,79223646	56,09241034	35,17776123	83,11891126	94,27700044	89,40376549	68,39741516	0,967194142	0,261191349	3,703009865	0,000213057	0,006122543	1,955034602
MTOR	955,4594437	446,2790893	898,5405296	1587,263357	1352,201549	1588,704913	875,021062	0,978948332	0,264616719	3,699495385	0,000216029	0,00616855	1,971028083
C4orf29	123,4661249	100,3758922	116,5891515	180,3988222	184,0646199	177,0194557	140,7596026	0,67532935	0,182641012	3,697577783	0,000217667	0,00618788	1,596961298
SFN	238,5141049	334,0942686	247,2494075	430,3712516	437,2657068	438,9724885	251,4870833	0,666619054	0,180352559	3,696199593	0,000218851	0,006207847	1,587348664
FAM73A	72,25574354	104,3122017	68,34536467	171,1633876	152,6389531	199,370397	94,75174641	1,084979828	0,293738778	3,693689461	0,000221024	0,006255699	2,121345822
ADRB2	25,25443463	64,94910671	33,16760344	105,2839543	87,99186708	101,9202927	74,90783279	1,235287452	0,335166933	3,685588673	0,000228175	0,006429831	2,35428251
TM4SF1	1290,782215	724,2809476	1347,810794	2039,183956	2347,048373	2065,226983	5580,810857	0,941091607	0,255885597	3,677782644	0,00023527	0,006615308	1,919980432
CAPRIN2	61,03155037	52,64813953	68,34536467	124,3705191	105,949391	118,9070081	87,16244203	0,952028633	0,259752436	3,665138421	0,000247205	0,006920661	1,934591055
RGS17	45,59828476	29,52232123	39,1980768	108,9781281	64,64708602	72,41705005	70,4186451	1,124935016	0,307123557	3,662809281	0,000249464	0,006968766	2,180917228
RASSF7	156,4371923	115,1370528	150,7618338	264,1334291	188,5540009	266,4232212	210,5680958	0,775959637	0,212131803	3,657912806	0,000254278	0,00707254	1,712328662
ARHGEF10	98,21169024	81,67842208	97,49265254	161,9279531	146,3538197	133,2116106	130,740848	0,682756663	0,186777875	3,655447214	0,000256734	0,007106658	1,605204007
DDX1	522,6264945	397,0752206	540,732444	1178,441453	797,3140609	1029,037341	728,6560581	1,042491818	0,285264672	3,654472222	0,000257712	0,007106658	2,059782232
PITRM1	147,3175354	115,1370528	126,6399404	338,6326014	224,4690487	313,8072169	288,7444026	1,174154095	0,322186803	3,644327087	0,000268092	0,007345729	2,256605291
GABBR2	52,61340549	101,8520083	53,26918129	179,1674309	143,6601911	163,6088908	57,94783828	1,214605739	0,333739826	3,639379075	0,000273296	0,007456587	2,320773518
SLC25A40	28,06048293	14,76116062	26,1320512	70,18930284	46,68956212	63,4766735	55,04289238	1,412721384	0,388894054	3,632663878	0,00028051	0,0075891	2,662389011
DONSON	129,7797335	108,2485112	134,6805716	214,2620824	172,3922294	246,7543927	211,2949183	0,771532607	0,212860798	3,624587597	0,000289423	0,007797465	1,707082292
PLAG1	18,2393139	3,936309498	19,09649895	76,96195487	40,40442876	49,17207102	18,2130394	2,056156507	0,567538596	3,622936873	0,000291277	0,00779847	4,158768869
SIGLEC15	424,4148043	525,497318	413,0874247	750,5329839	814,3737086	633,8726973	1161,735229	0,687639108	0,189769661	3,62354607	0,000290591	0,00779847	1,610645627
IL1RAP	58,22550207	72,82172571	57,28949685	148,9983446	115,8260291	148,4102507	89,16373909	1,128541672	0,31172469	3,620315329	0,000294244	0,00786157	2,186376218
SRPRB	445,4601665	355,2519322	505,5546828	699,4302459	668,917765	664,2699776	633,425532	0,642382854	0,177772052	3,61351993	0,000302068	0,008053899	1,56090513
TNKS2	447,5647027	278,9859357	482,4378683	736,9876799	874,5314136	635,6607726	647,5039813	0,89754506	0,249117507	3,602898364	0,000314689	0,0083386	1,862893321
EVI5	119,2570524	128,9141361	108,5485204	232,7329515	187,6561247	269,1053341	146,2665315	0,947482638	0,26310899	3,601103253	0,00031687	0,008347628	1,928504668
RUFY2	133,988806	183,0383917	149,7567549	276,4473419	237,0393154	249,4365057	245,7170272	0,705117165	0,195810521	3,601017768	0,000316974	0,008347628	1,630277058
ARRB1	170,4674338	135,310639	204,0310151	300,4594718	307,9715348	280,7278236	246,816823	0,810511995	0,225382751	3,596158054	0,000322952	0,008480902	1,753833746
SH3RF3	66,64364695	90,53511845	79,40123248	138,5315188	167,0049722	137,6817989	114,6858277	0,900304911	0,250721766	3,590852626	0,000329598	0,008619913	1,866460414
TMTC3	621,5396968	360,6643577	546,7629174	948,171284	1076,553557	931,5872364	696,4159552	0,952680899	0,265832886	3,583758623	0,000338685	0,008811286	1,935465912
LCN2	364,786278	474,3252945	340,7217445	636,6292907	805,3949466	611,5217559	277,6149116	0,796286914	0,222300795	3,582024588	0,000340942	0,008852111	1,73662579
USP46	169,0644096	120,0574397	166,8430961	225,9602995	268,4649822	227,979602	192,4404336	0,671591467	0,187611982	3,579683243	0,000344011	0,008891644	1,592829082
RECK	11,92570524	12,79300587	11,05586781	50,4870424	22,44690487	21,45690372	48,56974882	1,415546839	0,39592381	3,575301113	0,000349825	0,008998744	2,667608292
WNT7A	164,8553372	130,3902521	211,0665674	306,0007326	290,9118871	325,4297064	120,3807629	0,873275211	0,244441769	3,572528601	0,000353551	0,009051745	1,83181678
GLIPR1	366,1893022	471,8651011	309,5642988	689,5791157	659,0411269	641,0249986	581,1635603	0,790357928	0,221636467	3,566010318	0,000362457	0,009261354	1,729503494
MCL1	955,4594437	1501,210035	954,8249476	2159,244606	2024,710819	1676,320603	1826,74432	0,779360388	0,218842453	3,561285193	0,000369044	0,009373862	1,71636976
MLPH	35,77711573	27,55416649	41,20823458	64,03234645	64,64708602	57,21840991	231,2114238	0,862403864	0,242329999	3,558799437	0,000372554	0,009425833	1,818065105
DENR	763,9466477	744,9465725	738,7329858	1405,017448	1107,979224	1426,884097	788,74647	0,809895378	0,228000327	3,552167624	0,000382071	0,009591258	1,753084307
FAM13B	144,5114871	148,5956835	127,6450193	296,1496023	235,243563	304,8668403	198,4663316	0,989193626	0,278724581	3,549000317	0,000386697	0,009688481	1,98507515
BAG4	446,8631906	428,5656966	346,7522178	570,1341617	630,3090887	582,0185133	550,1638628	0,540028613	0,152245072	3,547100771	0,000389496	0,009720783	1,454001354

DKC1	1085,239177	1160,227224	1157,850884	1690,084529	1619,768655	1642,347172	1381,568866	0,541278912	0,152822266	3,54188514	0,000397278	0,009895844	1,455261997
IMPAD1	1701,166777	1113,483549	1549,831652	2433,844861	2728,645756	2669,596437	1905,585189	0,844054678	0,238958624	3,532221035	0,000412085	0,010146898	1,795088134
ITGAV	343,0394038	586,5101152	301,5236677	881,0604594	692,2625461	824,3027178	874,252121	0,958292798	0,271280402	3,532480752	0,00041168	0,010146898	1,943009289
SPSB1	866,3674104	949,6346664	1058,348073	1738,724484	1458,15094	1492,148846	1134,655187	0,706966128	0,200313477	3,52929887	0,000416662	0,010220528	1,632367766
PLEKHA6	24,55292256	34,93474679	27,13713009	49,87134676	59,25982885	63,4766735	100,0862047	0,970025025	0,275117006	3,525863551	0,000422104	0,010334335	1,958874573
TMEM2	286,2169259	358,696203	249,2595653	741,2975493	513,5851834	667,8461282	1166,69927	1,10292141	0,313253183	3,520862575	0,000430145	0,010451724	2,147891935
CHPT1	296,0380949	222,8935253	308,5592199	466,6972943	472,2828784	474,7339947	369,5085335	0,776354601	0,220732016	3,517181678	0,000436155	0,010527187	1,712797507
IPO7	2737,30011	2743,115681	2496,615968	4135,011911	3663,334874	3776,415054	3057,863363	0,536938819	0,152676349	3,516843455	0,000436711	0,010527187	1,450890678
LOC441666	1,403024146	0,492038687	2,010157784	12,31391278	8,080885752	8,940376549	4,759536794	3,074370392	0,874932077	3,513838929	0,000441681	0,01057242	8,4232115
PFKP	1939,680882	2442,480043	2293,590032	3418,957883	2950,421176	3128,237754	2913,824343	0,508700582	0,144750307	3,514331621	0,000440862	0,01057242	1,422768149
RFC4	315,6804329	338,5226168	379,9198212	446,9950339	516,2788119	529,2702917	479,4997125	0,530465079	0,151144147	3,509663402	0,000448674	0,010660567	1,444394748
FXYD5	3492,1271	3906,787177	3755,97982	5302,986538	5369,299644	5202,405114	6645,547341	0,508990275	0,145270169	3,503749459	0,000458757	0,010860054	1,423053869
NRGN	42,79223646	87,58288633	73,37075913	152,0768228	94,27700044	170,7611921	97,60928014	1,030790561	0,294987058	3,494358588	0,000475202	0,011187669	2,043143536
NXT1	20,34385012	16,23727668	14,07110449	44,94578165	38,60867637	36,65554385	25,14172848	1,242627153	0,355906477	3,491442928	0,000480419	0,011248795	2,366290425
KRT8	4857,971107	7710,738268	5907,853728	10284,57995	9203,230996	10050,77132	7648,139186	0,676738912	0,193907652	3,490006214	0,000483009	0,011268467	1,598522346
LAYN	340,9348676	495,9749967	319,6150877	741,2975493	742,543613	618,6740572	617,7880165	0,859416676	0,246623853	3,484726501	0,00049264	0,011410471	1,814304586
UBL3	216,7672306	189,4348946	223,1275141	371,2644703	264,8734774	424,6678861	371,4562251	0,756519247	0,217272436	3,481892413	0,000497884	0,011511214	1,689409708
C9orf72	42,79223646	44,28348185	36,18284012	87,42878073	74,52372416	105,4964433	54,70555583	1,109573637	0,319557576	3,472218221	0,000516177	0,011786032	2,157818673
SH3RF3-AS1	35,77711573	22,63377961	42,21331347	47,4085642	82,60460991	90,29780314	56,33973095	1,147015031	0,330731567	3,468114762	0,000524123	0,011904164	2,214552242
B3GNT5	98,21169024	101,3599696	98,49773143	184,7086917	282,8310013	141,2579495	112,1169696	1,028775255	0,296711001	3,467263606	0,000525786	0,01191992	2,040291454
LGR5	49,10584512	47,23571397	40,20315569	111,4409107	75,42160035	124,271234	52,59066571	1,184460606	0,341656915	3,466812912	0,000526668	0,01191992	2,27278404
CNIH	1383,381808	1038,20163	1361,881899	1751,038397	2002,263914	1879,267151	1691,838763	0,575012663	0,166185285	3,460069657	0,000540036	0,012137137	1,489690539
MPP5	145,2129991	244,0511889	168,8532539	374,3429485	342,9887064	325,4297064	357,7588989	0,897460459	0,259356435	3,460336195	0,000539501	0,012137137	1,862784082
OGFRL1	58,92701415	65,93318409	65,33012799	416,2102519	226,2648011	362,9792879	461,1596991	2,40262967	0,695475325	3,454658398	0,00055099	0,012361768	5,287660941
RPL5	12469,3771	9782,221141	12495,14079	17609,51097	16093,53291	15827,1486	12788,38993	0,511612384	0,148422862	3,446991772	0,000566866	0,012673782	1,425642631
MYBBP1A	1894,78411	1680,312117	1869,446739	2756,469376	2628,083622	2366,517672	1816,986757	0,51021712	0,148149966	3,443923306	0,000573338	0,012774136	1,424264526
MST1R	102,4207627	109,7246273	118,5993093	196,4069088	177,7794865	178,807531	75,40888988	0,745854134	0,216769998	3,440762747	0,000580077	0,01286645	1,676966808
SLC25A36	244,1262015	157,9444186	236,1935397	406,3591217	369,9249922	364,7673632	295,576458	0,843260387	0,245092845	3,440575291	0,000580479	0,01286645	1,794100101
FOXN2	51,21038134	17,71339274	45,22855015	125,6019104	96,07275283	126,953347	76,98737944	1,624487825	0,473085588	3,433813809	0,000595153	0,013123936	3,083326837
MYCBP2	148,7205595	158,4364573	166,8430961	306,6164282	228,0605534	264,6351458	294,1591891	0,757438564	0,221196121	3,424285015	0,000616419	0,013409203	1,69048658
APMAP	559,8066344	482,1979135	515,6054717	777,0078964	659,0411269	678,5745801	721,7201871	0,443742865	0,129647417	3,422689592	0,000620048	0,013431377	1,360128411
EPG5	263,0670274	182,546353	210,0614885	403,2806435	332,214192	430,032112	406,5994184	0,831484047	0,243028737	3,42134044	0,000623133	0,013431377	1,779514942
MAGI1	57,52399	37,88697892	59,29965464	96,66421532	96,97062903	97,45010438	68,24841587	0,929077135	0,271573067	3,421094531	0,000623696	0,013431377	1,904057616
SLC35A5	108,0328593	32,96659204	88,44694251	235,1957341	182,2688675	241,3901668	158,4079532	1,529379795	0,44712612	3,420466233	0,000625139	0,013439966	2,886617187
RTN2	14,73175354	3,444270811	15,07618338	36,3260427	27,83416204	32,18535558	24,84447066	1,605731723	0,469536506	3,419822955	0,000626619	0,013449333	3,043500753
DOCK4	419,5042198	201,2438231	417,1077403	668,0297683	587,2110313	615,0979066	591,4175358	0,854668952	0,250021446	3,418382567	0,000629945	0,013475799	1,808343756
HIST1H2AD	86,98749707	49,69590741	80,40631138	145,9198664	137,3750578	143,9400624	121,4826322	0,988068285	0,289029973	3,418566849	0,000629519	0,013475799	1,983527342
GDPD5	90,49505744	43,29940448	91,46217919	165,6221269	140,9665626	147,5162131	123,9633251	1,027893037	0,300759555	3,417657125	0,000631626	0,013489358	2,039044183
KCNAB2	474,9236735	321,3012628	556,8137063	775,1608095	762,2968893	750,0975924	328,2371629	0,761578532	0,223025257	3,414763603	0,000638374	0,013597651	1,69534458
DLC1	568,2247793	988,013684	563,8492585	1163,664758	1513,819264	1175,659516	1035,697949	0,859549596	0,252024992	3,410572853	0,000648266	0,013730863	1,81447175
ARPC1A	1106,986051	1204,510706	1184,988014	1453,657404	1611,687769	1541,320917	1259,046637	0,397238625	0,116568716	3,407763571	0,000654976	0,013827514	1,316984736
TYW5	16,13477768	4,920386872	16,08126228	51,10273803	30,52779062	42,01976978	25,71203665	1,781145343	0,52286743	3,406495109	0,000658027	0,013846529	3,43698926
HAS2	218,1702548	162,8648055	215,0868829	312,7733846	318,7460491	312,0191416	166,8349667	0,66784418	0,196241065	3,403182611	0,000666057	0,013965924	1,588697201
SLC25A24	644,6895952	246,0193436	490,4784994	990,6542831	878,1229184	1046,024056	1056,419581	1,079222048	0,317197066	3,402370836	0,000668039	0,013965924	2,112896426
C11orf82	115,7494921	97,42366007	104,5282048	184,0929961	167,0049722	162,7148532	135,0428811	0,697698189	0,205384115	3,397040661	0,000681188	0,014171801	1,621914972
ADAMTSL1	103,1222748	106,2803564	91,46217919	181,6302135	140,9665626	156,4565896	152,2030983	0,669225712	0,197224139	3,393224145	0,000690751	0,014324461	1,590219275
C20orf197	22,44838634	34,44270811	19,09649895	51,10273803	57,46407646	86,72165252	36,02700504	1,334075382	0,393327187	3,391770074	0,000694427	0,014377539	2,521138512
LRP10	598,3897984	411,8363812	583,9508364	793,015983	848,493004	736,6870276	1040,034794	0,580344216	0,171606473	3,381831724	0,000720042	0,014860105	1,495205951
CPNE7	4,209072439	1,476116062	1,005078892	6,772652029	5,387257168	15,19864013	91,93150411	1,988005546	0,589061489	3,374869321	0,000738507	0,015168261	3,966882173

AGPAT5	368,9953505	304,5719474	346,7522178	539,9650754	496,5255357	438,9724885	381,9776015	0,535838727	0,158798004	3,374341703	0,000739925	0,015173167	1,449784759
C7orf23	39,2846761	44,28348185	37,18791901	165,6221269	69,13646699	111,7547069	104,4507829	1,520980522	0,451109572	3,371643205	0,000747212	0,01525301	2,869860322
PION	14,03024146	16,23727668	13,0660256	25,2435212	31,42566681	40,23169447	55,27790342	1,127950374	0,334537365	3,371672321	0,000747133	0,01525301	2,185480301
SCML2	30,86653122	46,2516366	46,23362904	88,66017201	73,62584796	74,20512536	59,01350558	0,944102262	0,280017391	3,371584378	0,000747371	0,01525301	1,923991283
ITGBL1	17,53780183	15,2531993	21,10665674	46,79286856	46,68956212	35,7615062	23,34598859	1,284128738	0,381094894	3,369577394	0,000752835	0,015340214	2,435349345
PLCD3	390,7422248	367,0608607	393,9909257	648,3275078	659,0411269	512,2835762	615,00182	0,661095032	0,196306757	3,367663146	0,000758082	0,015422709	1,581282391
KIAA1549L	560,5081465	457,5959791	646,2657277	806,5612871	876,327166	786,7531363	1159,064297	0,572737869	0,170171296	3,365654972	0,000763622	0,015510913	1,487343495
LOXL4	2,806048293	0,984077374	0	10,46682586	7,183009557	8,046338894	8,760668449	2,691193405	0,801379563	3,358200695	0,000784516	0,015860284	6,45847435
ZNF367	208,3490857	199,767707	188,9548317	297,9966893	380,6995065	339,7343089	412,8250918	0,768062855	0,229105499	3,352441824	0,000801021	0,016143263	1,702981609
EDC4	155,7356802	129,4061747	156,7923072	217,3405606	204,7157724	222,6153761	180,6382889	0,55257582	0,164851865	3,351953714	0,000802434	0,016146483	1,466702042
PTBP3	867,0689224	461,0402499	762,8548792	1116,871889	1213,928615	1188,176043	960,7244232	0,752727414	0,224648938	3,35068316	0,000806125	0,016195437	1,684975266
PLXNA2	323,3970657	203,7040165	397,0061624	525,8040757	644,6751078	539,9987435	468,4820724	0,892709202	0,266525867	3,349428004	0,000809786	0,016225658	1,856659428
GNG5	2007,026041	2143,320522	2093,579332	2861,137634	2818,433375	2673,172588	2218,648918	0,419821408	0,125415124	3,347454386	0,000815574	0,016276094	1,337761943
LINC00641	74,36027976	84,6306542	54,27426018	139,1472144	120,3154101	106,3904809	137,0682422	0,764597172	0,228551061	3,345410725	0,000821608	0,016328192	1,698895568
VTA1	561,2096585	483,1819909	552,7933907	810,2554609	872,7356612	774,2366091	624,5796731	0,622542359	0,186183104	3,343710282	0,00082666	0,016378046	1,539585896
VEPH1	115,04798	97,42366007	135,6856504	207,4894303	165,2092198	170,7611921	387,5798935	0,658610282	0,197395821	3,33649557	0,000848418	0,016757553	1,578561296
GLRX3	1188,361452	1172,036153	1332,734611	1954,833654	1531,776788	1971,353029	1424,818928	0,564452114	0,169282083	3,334387811	0,000854874	0,016823823	1,478825795
TGFA	61,73306244	17,71339274	67,34028578	120,6763452	122,1111625	105,4964433	218,414797	1,270086209	0,38098426	3,333697328	0,000856999	0,016823823	2,411759767
UACA	4575,963253	5754,392447	4577,129275	6861,312201	7789,973865	7296,241302	4343,302554	0,557690394	0,167271894	3,334035274	0,000855958	0,016823823	1,471910953
CD9	206,2445495	188,9428559	246,2443286	303,53795	313,3587919	310,2310662	804,4027961	0,539192209	0,161804713	3,332364053	0,000861115	0,016878908	1,45315864
PCGF3	284,8139017	235,6865312	331,6760344	466,0815987	359,1504779	508,7074256	324,8668212	0,652264677	0,195932869	3,329021216	0,000871518	0,017023611	1,571633339
YTHDF2	1071,910448	731,6615279	961,8604998	1676,539225	1292,94172	1814,002402	1112,702035	0,791391545	0,237693854	3,329457337	0,000870154	0,017023611	1,730743038
ZNF185	126,2721732	86,10677027	119,6043882	189,0185612	229,8563058	174,3373427	98,71869013	0,843367451	0,253734564	3,323817767	0,000887942	0,017169545	1,794233248
SH3BGRL2	7,716632805	8,364657683	9,04571003	17,85517353	23,34478106	19,66882841	37,23624341	1,274920821	0,384257007	3,317885676	0,000907016	0,01743366	2,419855366
ALOX5AP	62,43457451	136,2947164	90,4571003	216,1091693	192,1455057	148,4102507	738,2960957	0,93787641	0,282942305	3,314726694	0,000917328	0,017579383	1,915706319
G3BP1	6461,627706	3121,985471	6953,135776	11793,64996	10416,26173	9795,076547	7782,869191	0,952890756	0,288148556	3,306942676	0,000943202	0,017968276	1,93574747
OSBPL8	1295,692799	701,1551293	1188,003251	2045,956608	1810,118408	1859,598322	1305,788624	0,844840526	0,25564303	3,304766514	0,000950556	0,018081616	1,796066199
AGPAT2	291,1275104	393,1389111	454,2956593	664,9512901	527,0533263	629,402509	502,1561806	0,680638469	0,20612385	3,302084974	0,00095969	0,018201601	1,602848944
LUZP1	1160,300969	1398,373949	1198,054039	1729,48905	1863,093104	1693,307318	1292,581707	0,491675134	0,148973128	3,300428336	0,000965374	0,018228859	1,406076544
RASA3	371,0998867	204,6880939	350,7725334	571,9812486	458,8147355	558,7735343	743,4421121	0,783479213	0,237455407	3,299479347	0,000968644	0,018263824	1,721276914
APLN	10,5226811	3,936309498	3,015236677	20,31795609	11,67239053	26,82112965	17,82816957	1,738839293	0,528384034	3,29086267	0,000998807	0,018777564	3,33766531
SPOCD1	519,1189341	250,4476918	471,3820004	808,408374	738,054232	749,2035548	451,5316889	0,890409785	0,27082863	3,287723989	0,001010008	0,01890845	1,853702577
SLC4A7	260,9624912	205,6721713	249,2595653	670,4925508	499,2191642	529,2702917	386,3807652	1,248097961	0,379767259	3,286481212	0,001014475	0,018961414	2,375280611
NEK4	376,0104712	365,0927059	339,7166656	520,2628149	527,9512025	446,1247898	413,3558383	0,46645091	0,142106531	3,282403044	0,001029264	0,01918214	1,381706229
ALCAM	963,8775885	1480,54441	1062,368389	2154,934736	1503,04475	1950,790163	2771,70523	0,676778891	0,206335086	3,279999075	0,001038074	0,019318382	1,598566644
UBE2W	110,1373955	76,26599652	143,7262816	355,2563837	228,9584296	326,323744	192,8926657	1,468917295	0,448237253	3,277097752	0,001048801	0,019435043	2,768140739
LIN28B	15,43326561	9,840773745	25,1269723	58,4910857	40,40442876	45,5959204	16,24103721	1,562404595	0,477431448	3,272521329	0,001065928	0,019666224	2,953456975
NRARP	7,015120732	4,428348185	8,040631138	22,78073864	23,34478106	19,66882841	11,2251753	1,798718624	0,550347465	3,268332714	0,001081831	0,019817668	3,479110797
HSPA5	6728,903806	3947,626388	6092,788244	9776,015356	9132,298776	8572,033035	7969,300384	0,712827261	0,218232173	3,266371094	0,001089354	0,01992713	1,639012958
RPL32	11222,79015	9917,53178	11869,98172	18447,47273	16475,1303	16814,16618	13707,58914	0,648361006	0,198557845	3,26535074	0,001093286	0,019970692	1,567386532
SGK196	326,203114	425,1214258	315,5947721	522,1099019	529,7469549	573,9721744	502,404826	0,603289084	0,185117548	3,258951354	0,001118248	0,020254294	1,519176069
LSM6	117,1525162	78,72618996	117,5942304	165,6221269	170,596477	169,8671544	150,8518123	0,702790517	0,215963193	3,254214327	0,001137065	0,020537332	1,627650014
SLC17A9	108,0328593	108,7405499	108,5485204	161,9279531	186,7582485	187,7479075	71,81300321	0,719370602	0,221053385	3,254284481	0,001136784	0,020537332	1,646463582
PANX1	138,1978784	92,99531189	124,6297826	255,5136902	221,7754201	201,1584723	177,3270146	0,936523027	0,287988529	3,251945594	0,00114618	0,02067297	1,91391005
C10orf116	70,15120732	115,1370528	86,43678473	168,0849094	139,1708102	158,2446649	193,9259463	0,769251843	0,236783753	3,248752645	0,001159122	0,020848012	1,704385689
ZNF483	16,83628976	6,888541621	21,10665674	32,01617323	47,58743832	29,50324261	31,25312149	1,338194966	0,413146195	3,239034955	0,001199349	0,02148152	2,528347855
CAPN2	1940,382394	1681,788233	1901,609264	2588,384466	3039,310919	3288,270495	3161,06042	0,690780327	0,213540841	3,234886238	0,001216912	0,021735636	1,61415635
SLC35F5	537,3582481	738,5500695	467,3616849	910,61385	817,9652134	923,5408975	879,3349225	0,601727153	0,186080055	3,233700421	0,001221976	0,021765701	1,517532226
SIRPB1	457,3858717	467,4367529	505,5546828	763,4625923	707,5264414	686,620919	379,3355776	0,594419578	0,184286661	3,225516023	0,001257458	0,022274476	1,509865018
ENC1	1286,573142	861,0677027	1189,008329	1643,907356	1705,066894	1603,903553	1095,125135	0,571157203	0,177160691	3,223949951	0,001264355	0,022365883	1,485714802

C1orf106	49,10584512	38,87105629	43,21839236	65,26373773	66,44283841	72,41705005	238,7242004	0,643400038	0,199608955	3,223302478	0,001267217	0,022385755	1,562006046
SGMS2	260,9624912	193,8632428	279,411932	384,8097744	338,4993254	376,3898527	466,9772159	0,590522324	0,183647322	3,215523748	0,001302067	0,022690151	1,505791816
NUP155	1072,61196	773,976855	1277,455272	1702,398442	1814,607789	1724,598636	1070,802854	0,747980952	0,233442353	3,204135593	0,001354687	0,023543393	1,679440812
INIP	231,4989841	253,8919626	207,0462518	319,5460366	357,3547255	312,9131792	324,6961493	0,50894244	0,158867665	3,203562154	0,001357387	0,023558537	1,423006686
ANKRD1	16,83628976	3,444270811	12,06094671	52,33412931	45,79168593	28,60920496	13,98194457	2,00130731	0,62629349	3,195478387	0,001395992	0,024098646	4,003626275
TNFAIP3	565,418731	692,2984329	601,0371775	1027,596021	861,9611469	1080,891525	467,1057955	0,675701619	0,211582849	3,193555719	0,001405322	0,024194838	1,597373426
CFL1	20223,19005	19126,52785	19784,97799	28125,59248	24139,40149	26412,55444	23533,49161	0,411993208	0,12912058	3,190763285	0,001418975	0,024379676	1,330522777
HSPA8	16211,2425	14353,75258	16362,68436	25094,52285	19483,01555	23314,71396	21301,48108	0,532887895	0,16710669	3,188908203	0,001428112	0,024433504	1,446822455
SSR1	1495,62374	1550,413903	1383,993635	2467,708121	2104,6218	2446,981061	1917,105127	0,663808726	0,208127717	3,189429721	0,001425538	0,024433504	1,584259565
TMEM170A	93,30110573	55,60037166	88,44694251	129,2960842	146,3538197	131,4235353	113,2156481	0,790564521	0,248196031	3,185242402	0,001446328	0,024702669	1,729751175
GINM1	293,9335587	148,1036449	241,2189341	435,9125124	369,027116	425,5619237	309,8437365	0,852895313	0,26819411	3,180141849	0,00147203	0,02497602	1,806121957
HEXB	939,324666	1308,822908	981,9620777	2042,87813	1392,605978	1786,287234	1315,228127	0,692418807	0,218107836	3,174662677	0,001500108	0,02538553	1,615990601
ATP6V1B2	933,7125694	1229,112641	926,6827386	1679,617703	1501,248998	1451,917152	1855,248974	0,583462393	0,183896597	3,172774283	0,001509899	0,025494877	1,498441118
SAMD3	50,50886927	49,20386872	68,34536467	96,04851968	118,5196577	122,4831587	43,71139728	1,011701931	0,319978033	3,161785582	0,00156805	0,026259226	2,01628829
RAPGEF3	50,50886927	26,07805042	37,18791901	72,6520854	66,44283841	80,46338894	52,35365985	0,960324591	0,304032985	3,158619752	0,001585182	0,026511649	1,945747618
BUB3	2615,237009	2673,246188	2797,134557	3699,099399	3639,990093	3174,727712	2922,46073	0,379283563	0,120154707	3,156626768	0,001596055	0,026624347	1,300695774
POLQ	117,1525162	78,23415127	121,614546	179,7831266	163,4134674	223,5094137	173,0630911	0,846163276	0,26813497	3,155736372	0,001600935	0,026636743	1,797713697
TULP4	110,1373955	125,9619039	116,5891515	214,2620824	180,4731151	206,5226983	187,8397255	0,76904002	0,243678702	3,155959114	0,001599713	0,026636743	1,704135462
TBL1X	144,5114871	140,7230645	132,6704138	253,6666033	223,5711725	206,5226983	170,5055722	0,710718492	0,225339051	3,153996111	0,001610512	0,026692635	1,636618984
SOWAHC	613,823064	237,6546859	603,0473353	849,0442861	1187,890206	1002,216211	833,9431219	1,065033304	0,337806026	3,152795455	0,001617151	0,026699574	2,092218177
ZW10	467,9085528	214,0368289	400,0213991	709,8970717	564,7641265	700,0314838	501,3889282	0,871144161	0,276307862	3,152802651	0,001617111	0,026699574	1,829112944
DOCK2	136,7948543	136,2947164	142,7212027	216,1091693	243,3244488	204,734623	92,8321583	0,676392536	0,215053184	3,145233772	0,001659542	0,027247393	1,598138604
SNRK	110,8389076	113,1688981	119,6043882	254,8979945	157,1283341	216,3571125	150,8238474	0,874607728	0,278095322	3,144992591	0,00166091	0,027247393	1,833509484
PDE2A	266,5745878	396,0911432	266,3459064	526,4197713	526,1554501	479,204183	265,3731663	0,717542855	0,228825495	3,135764459	0,001714068	0,028012397	1,644379003
ARHGAP12	82,07691256	60,02871984	91,46217919	116,9821714	122,1111625	125,1652717	149,2695114	0,658863295	0,210320087	3,132669367	0,001732245	0,028273568	1,578838161
AP2S1	3151,893745	2812,985175	3241,379427	4558,610511	4465,138316	3978,467564	4192,029789	0,498453622	0,159225351	3,130491581	0,00174514	0,028447991	1,412698525
AP1S3	44,89677268	26,07805042	54,27426018	137,3001275	93,37912425	93,87395376	76,27964963	1,389531578	0,444499021	3,126062176	0,001771641	0,028698354	2,619936014
GSTO1	295,3365828	353,2837774	386,9553735	617,5427259	588,1089075	507,813388	651,5215081	0,728072608	0,232896353	3,126165777	0,001771017	0,028698354	1,656424686
GALNT14	240,6186411	306,5401021	290,4677998	442,0694688	380,6995065	366,5554385	752,1933413	0,50699409	0,162350128	3,122843793	0,001791128	0,028796684	1,421086222
RBPJ	350,7560366	489,5784938	352,7826912	644,0176384	596,1897933	718,8062745	510,0263077	0,71267653	0,228205947	3,122953358	0,001790461	0,028796684	1,638841725
PSMA2	1095,060346	876,8129407	1049,302363	1688,853138	1503,942626	1583,340687	1288,370181	0,66148092	0,212118231	3,11845388	0,001818026	0,029192691	1,581705404
INSR	38,58316402	22,63377961	33,16760344	52,94982495	70,03434319	63,4766735	37,8480451	0,993235324	0,318910798	3,114461256	0,001842812	0,029553832	1,990644119
BCAR3	1050,865086	512,7043121	1035,231259	1463,508534	1469,823331	1430,460248	882,7841331	0,74966861	0,241005923	3,110581682	0,001867192	0,02972275	1,681406563
BMPER	4,209072439	3,936309498	6,030473353	6,772652029	20,65115248	22,35094137	12,50643756	1,825004894	0,586470164	3,111846101	0,001859214	0,02972275	3,543082095
DKK3	4075,785145	4273,355999	4378,123654	5761,67979	5441,12974	6088,39643	3034,301065	0,442179853	0,142137201	3,11093683	0,001864948	0,02972275	1,35865565
SLC16A6	32,26955537	15,74523799	22,11173563	39,40452089	56,56620027	49,17207102	61,24826912	1,056815589	0,339694683	3,111074859	0,001864077	0,02972275	2,0803346
POU2F2	336,024283	118,5813236	328,6607977	558,4359446	665,3262603	523,9060658	232,6429667	1,161283813	0,373584217	3,1084927	0,001880443	0,029859857	2,236563644
GADD45B	163,452313	210,1005194	189,9599106	287,5298634	292,7076395	269,1053341	239,9120165	0,589975426	0,18984953	3,10759488	0,001886164	0,029913822	1,505221108
AKTIP	243,4246894	255,3680787	198,0005418	325,702993	327,7248111	334,3700829	351,733664	0,496178209	0,159811403	3,104773508	0,001904248	0,030073035	1,410472178
GALNT6	133,2872939	191,895088	169,8583328	274,600255	247,8138297	270,8934094	238,99246	0,678678694	0,218585025	3,104872772	0,001903609	0,030073035	1,600673092
ORC6	295,3365828	236,6706086	306,5490621	389,7353395	375,3122494	387,1183046	484,0262136	0,465211486	0,149847211	3,104572208	0,001905544	0,030073035	1,38051971
ERO1L	363,3832539	436,4383156	301,5236677	914,9237195	602,4749266	810,892153	607,5323172	1,078970006	0,348220969	3,098521056	0,001944891	0,030581578	2,11252733
GID8	188,7067477	86,59880895	147,7465972	253,6666033	337,6014492	258,3768823	156,6704644	1,009616005	0,326296042	3,094171777	0,001973632	0,030822887	2,013375139
NAP1L5	9,821169024	8,85669637	2,010157784	33,86326014	12,57026673	28,60920496	21,97287905	1,808140375	0,58439719	3,094026469	0,001974599	0,030822887	3,50190604
RANBP17	156,4371923	192,8791654	158,802465	382,3469918	231,6520582	301,2906897	161,056482	0,848689474	0,27425994	3,094471159	0,001971641	0,030822887	1,800864299
TBC1D2	148,0190474	184,5145077	116,5891515	225,9602995	248,7117059	242,2842045	293,6298466	0,662987217	0,21425081	3,09444439	0,001971819	0,030822887	1,583357702
GALC	173,2734821	154,5001478	163,8278594	303,53795	211,8987819	288,7741625	197,3084064	0,712825821	0,230584847	3,091381893	0,001992272	0,031061116	1,639011323
TULP3	335,322771	619,4767072	325,6455611	632,3194212	767,6841465	770,6604585	676,2442686	0,756642812	0,244997152	3,088373906	0,002012551	0,031339334	1,689554411
ANKRD18A	37,18013988	13,28504456	15,07618338	46,17717292	52,07681929	46,48995805	84,67632777	1,143114344	0,37056561	3,084782597	0,00203701	0,031643683	2,208572733
PAR5	23,85141049	15,74523799	22,11173563	45,56147728	36,81292398	38,44361916	36,50997658	0,997828935	0,323510038	3,084383222	0,002039747	0,031648022	1,996992528

GLYR1	408,9815387	395,5991045	374,8944268	738,8347668	536,0320882	684,8328436	672,9911468	0,732712446	0,2380114	3,078476272	0,002080621	0,032112245	1,661760473
CA13	6,313608659	18,69747011	10,05078892	20,31795609	35,01717159	27,7151673	106,232781	1,169158466	0,379999441	3,076737334	0,002092796	0,032238075	2,248804844
STC1	3412,856236	4218,247666	3218,262613	5242,648366	5063,123862	5116,577499	2970,520927	0,506976644	0,165111937	3,07050267	0,002136988	0,032684238	1,421069037
SPTY2D1	490,3569391	255,8601174	419,117898	616,9270303	777,5607846	629,402509	487,9192845	0,798454578	0,260240794	3,06813765	0,002153974	0,032865964	1,739237049
ATP6V1C1	379,5180316	417,7408455	419,117898	725,2894627	617,7388219	718,8062745	629,5332951	0,761683634	0,249091254	3,057849769	0,002229313	0,033690808	1,695468093
CTSB	3296,405232	5335,667524	3206,201666	6555,311468	5650,334893	6172,435969	3405,732139	0,634064042	0,207398307	3,057228631	0,002233938	0,033690808	1,551930599
SPTLC1	1128,732926	885,669637	950,804632	1517,074054	1367,465445	1582,446649	1236,306413	0,591425326	0,193430745	3,057555954	0,0022315	0,033690808	1,506734607
SSR3	3473,186274	1396,897833	3113,734408	4610,328945	4905,097652	5317,735971	5835,565725	0,894088112	0,292523219	3,056468871	0,002239607	0,033693532	1,858434848
GBP3	244,1262015	258,3203108	220,1122774	388,5039482	337,6014492	374,6017774	407,4874368	0,605193974	0,198270083	3,05237161	0,002270408	0,034077294	1,521183266
OSBPL3	394,2497851	150,0717996	328,6607977	527,035467	574,6407646	616,8859819	511,3217892	0,980058929	0,321052729	3,05264164	0,002268367	0,034077294	1,972545978
MYCT1	75,7633039	98,89977613	82,41646916	136,6844319	136,4771816	179,7015686	59,16189427	0,810345698	0,265644528	3,05048895	0,002284691	0,034203386	1,753631596
RPS9	5326,581172	2478,890906	6379,235729	9545,745187	8743,518384	8300,245588	8078,224663	0,90686632	0,297456722	3,04873366	0,002298081	0,034332587	1,874968451
CLOCK	250,4398101	86,10677027	242,224013	434,0654255	420,2060591	397,8467564	379,5243485	1,1184063	0,366965437	3,047715638	0,00230588	0,034337157	2,171070087
TIAL1	512,8053255	438,898509	434,1940814	684,6535505	677,896527	627,6144337	541,1041005	0,522030118	0,171341901	3,046716046	0,002313562	0,034364527	1,435974488
FAM103A1	337,4273072	241,5909954	384,9452157	440,8380775	571,0492598	566,8198732	321,1873519	0,714832579	0,234692544	3,045825686	0,002320423	0,034387123	1,641292739
INF2	290,4259983	239,130802	321,6252455	408,2062086	388,7803923	378,177928	452,4155374	0,47324696	0,155475819	3,043862153	0,002335621	0,034572565	1,388230335
AK4	552,0900016	357,2200869	548,7730751	708,0499848	903,2634519	656,2236387	740,0395438	0,639622352	0,21029312	3,041575271	0,002353437	0,03475638	1,557921296
LAMC1	1425,472533	1318,663682	1132,723911	2000,395131	1859,501599	2012,478761	2640,999606	0,598559964	0,196983922	3,038623437	0,002376617	0,034917587	1,514204398
TSR1	997,5501681	945,2063182	1192,023566	1451,194621	1606,300512	1326,75188	1117,786614	0,485477492	0,159995562	3,034318494	0,002410798	0,035279833	1,400049167
IRAK1	308,6653122	114,1529754	381,929979	566,4399879	531,5427073	580,230438	764,2733248	1,065364865	0,351170456	3,033754252	0,002415311	0,035305759	2,092699067
GPR110	264,4700516	374,9334797	320,6201666	496,8663807	495,6276595	490,8266725	225,457737	0,62597791	0,206629155	3,029475247	0,00244979	0,035632387	1,543256546
ATP5G1	975,1017817	974,2366007	1216,14546	1528,772272	1458,15094	1451,023114	1268,543546	0,489116175	0,162156985	3,01631271	0,002558692	0,036940403	1,403584747
RNF152	67,34515902	47,72775266	82,41646916	161,9279531	94,27700044	133,2116106	50,44954769	0,995158711	0,330044305	3,015227639	0,002567864	0,037031309	1,993299796
TENM3	162,0492889	101,3599696	112,5688359	196,4069088	175,9837342	207,4167359	269,5734982	0,626974897	0,207997689	3,014335876	0,002575425	0,037057346	1,544323395
RAB19	0	0	0	2,462782556	6,285133363	0	6,374702397	4,484588638	1,488262525	3,013304819	0,002584192	0,037141993	22,38698964
PHB	1765,705888	797,1026733	2023,22381	2994,127892	2793,292842	2993,238069	1953,651641	0,937990286	0,311419496	3,011983186	0,00259547	0,037221001	1,915857537
EIF2C3	518,4174221	351,3156227	529,6765762	757,3056359	791,0289275	677,6805424	438,6246356	0,672401354	0,223429981	3,009449988	0,002617212	0,037449391	1,5937235
TIMP1	4720,47474	3051,623938	4267,564976	5775,225094	6021,157762	5293,596955	4587,184827	0,505779605	0,168055386	3,009600689	0,002615914	0,037449391	1,41989043
PDIA3	6250,472572	3206,616125	5526,928828	7978,18409	8261,358867	7800,478539	5974,844968	0,682288918	0,227243153	3,002461947	0,002678055	0,038019261	1,604683659
P2RY6	7,716632805	4,428348185	6,030473353	13,54530406	18,85540009	16,09267779	16,49653661	1,432338654	0,478053752	2,996187455	0,002733783	0,038527857	2,698838511
PLAT	1531,400856	1093,309963	1412,135844	2088,439607	2178,247648	1930,227297	909,5702122	0,618974496	0,20653217	2,996988299	0,002726612	0,038527857	1,535783119
LY75	99,61471439	82,17046077	87,44186362	154,5396054	137,3750578	143,0460248	60,42060686	0,695289243	0,232222294	2,99406758	0,00275285	0,038636992	1,619209032
RNF144B	11,22419317	15,74523799	11,05586781	39,40452089	26,03840965	22,35094137	40,72250246	1,196944967	0,400152446	2,991222422	0,00277863	0,038684541	2,292536919
TRHDE	59,62852622	50,1879461	65,33012799	111,4409107	107,7451434	92,08587845	68,07796658	0,840528207	0,280978676	2,991430595	0,002776736	0,038684541	1,790705644
FKBP1A	10265,22617	5605,796764	10079,93621	13162,95707	15170,51619	13921,06032	9283,887763	0,70347283	0,236616754	2,973047424	0,002948589	0,040394696	1,628419983
NHP2	735,1846527	698,6949359	1094,530914	1255,403408	1450,967931	1145,262236	820,6051817	0,609524108	0,20511838	2,971572352	0,002962791	0,040461099	1,525755836
PRKCZ	197,1248926	166,801115	218,1021196	292,4554285	272,056487	277,151673	149,9419847	0,539901487	0,181901122	2,968104211	0,002996427	0,040737818	1,453873238
SIRPA	291,8290224	278,0018583	258,3052753	485,1681635	423,7975639	518,5418398	637,5620568	0,784971148	0,264759734	2,964843399	0,00302837	0,041008785	1,723057861
EFR3A	469,311577	654,411454	390,9756891	794,2473743	705,730689	835,0311697	1142,209368	0,62062962	0,209575968	2,961358716	0,00306285	0,041432122	1,537546049
GXYLT1	150,8250957	56,58444903	105,5332837	222,8818213	234,3456868	197,5823217	140,0016095	1,070220055	0,362085125	2,955713949	0,003119463	0,04202138	2,099753619
BAG1	117,8540283	105,7883178	116,5891515	193,9441263	148,1495721	167,1850415	207,5723221	0,589399387	0,199605519	2,952821094	0,003148844	0,042252606	1,504620224
VAT1L	39,2846761	11,31688981	36,18284012	81,27182434	67,3407146	78,67531363	26,17268822	1,410288261	0,477630328	2,952677371	0,00315031	0,042252606	2,657902644
VKORC1	1610,67172	1533,684588	1904,624501	2239,285039	2056,136486	2239,564325	1919,883741	0,373782059	0,12677842	2,948309808	0,003195167	0,042772721	1,295745208
TMEM159	408,2800266	278,9859357	430,1737659	642,7862471	590,8025361	522,1179905	305,5808353	0,656079165	0,222712536	2,94585647	0,003220618	0,043023983	1,575794238
ENTPD7	388,6376885	309,984373	387,9604524	636,6292907	482,1595165	736,6870276	703,8822928	0,773511043	0,262804748	2,943291739	0,003247422	0,043193781	1,709424901
FAM35A	20,34385012	9,348735057	13,0660256	38,78882526	29,62991442	42,91380743	36,00600584	1,39280004	0,473454505	2,941782207	0,003263293	0,043193781	2,625878274
GLRX	222,3793272	113,6609368	188,9548317	385,42547	280,1373727	294,1383885	505,6472185	0,875880306	0,29752331	2,943904822	0,003240997	0,043193781	1,835127505
C14orf105	13,32872939	19,1895088	13,0660256	38,17312962	21,54902867	31,29131792	360,3289618	0,980348553	0,333673974	2,938043212	0,00330291	0,043580812	1,972942011
ELOVL6	77,86784012	64,45706803	83,42154805	129,2960842	123,9069149	130,5294976	313,099166	0,773485056	0,263555692	2,934806875	0,003337553	0,043902976	1,70939411
WDR62	113,6449559	136,2947164	102,518047	171,1633876	191,2476295	166,2910038	169,4627171	0,573864574	0,195716622	2,932119764	0,003366569	0,044194368	1,488505522

C10orf55	11,92570524	11,31688981	9,04571003	24,62782556	27,83416204	16,98671544	32,09158759	1,089554682	0,372103898	2,92809263	0,003410484	0,044589052	2,128083386
ILVBL	124,869149	102,3440469	130,660256	176,7046484	165,2092198	182,3836816	173,0968942	0,560123939	0,191404661	2,926386096	0,003429251	0,044698268	1,474395874
FIGN	77,16632805	53,14017822	71,36060135	100,3583892	146,3538197	94,76799142	347,804712	0,765843643	0,261847802	2,924766359	0,003447149	0,044886136	1,700364027
PPP2R5A	131,1827577	174,1816953	84,42662694	241,9683861	198,430639	260,1649576	187,3866579	0,835423121	0,286142888	2,919601207	0,003504796	0,045498749	1,784380294
MTX3	213,2596702	272,5894327	189,9599106	397,1236871	370,8228684	362,9792879	216,0609737	0,739071039	0,253207158	2,918839441	0,003513371	0,045564145	1,669100747
AMOTL1	484,7448426	346,3952358	477,4124738	733,9092017	672,5092698	684,8328436	589,8131194	0,678683464	0,232855984	2,91460607	0,003561378	0,045915523	1,600678384
QPCT	57,52399	64,45706803	73,37075913	112,6723019	105,949391	108,1785562	67,38246332	0,748946659	0,257316938	2,910599919	0,003607356	0,046362805	1,680565368
TJP1	193,6173322	172,2135405	155,7872283	251,8195163	289,1161347	217,2511501	497,6972888	0,536383825	0,18440742	2,908688961	0,003629478	0,046600611	1,450332639
APP	2261,674924	1522,367698	2040,310151	2830,968548	2845,369661	2435,358572	3548,064487	0,478793602	0,164743354	2,906299948	0,003657307	0,046911152	1,393577853
SOD2	3639,444636	5180,675338	3266,5064	6353,978994	7334,750634	5584,159192	5462,360106	0,672792128	0,231660009	2,904222147	0,003681668	0,047082944	1,594155241
EEF1A2	3,507560366	3,444270811	1,005078892	11,0825215	6,285133363	7,152301239	240,9059023	1,546891047	0,532840155	2,903105243	0,003694824	0,047204315	2,921868088
RAD51AP1	166,9598734	131,8663682	224,132593	263,5177335	288,2182585	322,7475934	225,8946718	0,748592823	0,258395594	2,897080448	0,003766532	0,048025142	1,680153242
ERO1LB	16,83628976	19,68154749	25,1269723	37,55743398	29,62991442	45,5959204	61,02431949	0,892837553	0,308467426	2,894430587	0,003798469	0,048384452	1,856824615
E2F7	387,9361765	501,3874223	429,168687	610,7700739	563,8662503	624,9323208	577,9908302	0,446654609	0,154347067	2,893832832	0,003805707	0,048428752	1,362876287
SNHG16	1756,586231	876,8129407	1675,466513	2606,855335	2257,260753	2373,669974	1901,795236	0,749247568	0,259178747	2,890852649	0,003841982	0,048721302	1,680915927
CCDC85C	211,155134	42,3153271	223,1275141	436,528208	342,9887064	376,3898527	262,1102077	1,284369022	0,444997488	2,886238814	0,003898762	0,049254098	2,435754991
HUWE1	2262,376436	1663,582802	2131,77233	2874,067243	2781,620451	2853,768194	1873,397193	0,490933609	0,170300831	2,882743467	0,003942284	0,049578835	1,405354026
PFAS	595,5837501	430,0418126	674,4079367	1031,290195	822,4545943	879,7330524	691,7921568	0,687886414	0,238606088	2,882937389	0,003939857	0,049578835	1,610921746
TCFL5	241,3201532	274,5575875	194,9853051	387,8882526	313,3587919	403,2109824	178,8466751	0,631304368	0,219067418	2,881781203	0,003954342	0,049633546	1,54896481
GRK6	244,1262015	229,2900283	270,366222	372,4958616	369,027116	336,1581582	268,3745629	0,538788248	0,18712751	2,87925729	0,00398613	0,049823685	1,452751806
SACM1L	262,3655154	134,3265616	234,1833819	385,42547	333,1120682	387,1183046	364,07769	0,814157172	0,282787397	2,879043337	0,003988835	0,049823685	1,758270664
