# Supplementary material for: Analysis of the Role of FRMD5 in the Biology of Papillary Thyroid Carcinoma
Source: Int J Mol Sci. 2021 Jun 23;22(13):6726. doi: 10.3390/ijms22136726 (PMC8268710; doi:10.3390/ijms22136726)
Supplement: Supplementary file 1 [file ijms-22-06726-s001.zip › Supplementary Table S4.pdf]

Supplementary Table S4. Genes downregulated in BCPAP siFRMD5 (in comparison with BCPAP siNEG).

gene	BCPAP siNEG 1	BCPAP siNEG 2	BCPAP siNEG 3	BCPAP siFRMD5 1	BCPAP siFRMD5 2	BCPAP siFRMD5 3	baseMean	log2FoldChange	lfcSE	stat	pvalue	padj	Fold-change
FRMD5	277,798781	226,3377961	294,4881154	49,87134676	51,1789431	50,96014633	94,86368746	-2,390527682	0,225328463	-10,60907995	2,70398E-26	1,16073E-22	5,243491131
TRIM16	165,5568493	188,9428559	162,8227805	22,78073864	37,71080018	45,5959204	199,5791517	-2,314671528	0,25359335	-9,127493015	7,01038E-20	1,1285E-16	4,974913797
AMACR	1122,419317	874,3527472	845,2713483	292,4554285	366,3334874	303,078765	492,9189017	-1,564748475	0,190281018	-8,223355589	1,97887E-16	1,59274E-13	2,958259219
CDH6	668,5410057	738,5500695	588,9762308	192,712735	148,1495721	180,5956063	641,4781636	-1,935605246	0,241188178	-8,025290708	1,01286E-15	7,24644E-13	3,825385776
TNFRSF9	242,0216652	233,7183764	237,1986186	82,50321562	77,21735274	90,29780314	210,4052397	-1,511989453	0,190148971	-7,951604718	1,84111E-15	1,24788E-12	2,852030584
IDO1	281,3063413	162,3727668	205,036094	40,02021653	38,60867637	46,48995805	64,48617109	-2,374607928	0,308020388	-7,709255689	1,26554E-14	8,14879E-12	5,185948675
CYP1B1	1122,419317	991,9499935	1011,109366	395,8922959	261,2819727	364,7673632	358,9668833	-1,610749729	0,210444846	-7,654023193	1,94787E-14	1,14021E-11	3,054105141
ARID5B	110,1373955	151,055877	64,3250491	24,62782556	21,54902867	26,82112965	135,1199366	-2,170912105	0,317056529	-6,847082156	7,53714E-12	2,94131E-09	4,503079991
ADAMTS5	890,9203329	737,5659922	620,1336765	312,7733846	239,732944	260,1649576	264,1575869	-1,467092342	0,224302193	-6,540695485	6,12334E-11	2,13125E-08	2,764641363
ALDH1A1	155,0341682	212,0686742	125,6348615	43,09869473	39,50655257	40,23169447	51,41168767	-2,008034734	0,308519108	-6,508623545	7,58425E-11	2,44175E-08	4,02233916
LCA5	122,0631007	124,9778266	110,5586781	37,55743398	47,58743832	37,54958151	89,14738544	-1,552724335	0,24206157	-6,414584241	1,41208E-10	4,04105E-08	2,933706072
NR0B1	51,21038134	82,17046077	63,31997021	8,619738946	10,77451434	7,152301239	18,60394724	-2,896137063	0,455015916	-6,364913759	1,95399E-10	5,24241E-08	7,44430447
C8orf4	266,5745878	202,7199391	170,8634117	64,03234645	70,93221938	57,21840991	69,47554339	-1,73817059	0,276194069	-6,293294401	3,10798E-10	7,69703E-08	3,336118629
DYNC2LI1	202,7369891	353,2837774	231,1681452	85,58169382	79,91098133	87,61569018	143,5867965	-1,640765927	0,263942801	-6,216369299	5,0879E-10	1,17003E-07	3,118313393
DNAJB4	250,4398101	277,0177809	217,0970407	89,89156329	98,76638142	86,72165252	198,1605292	-1,439068729	0,232077104	-6,200821637	5,61691E-10	1,24715E-07	2,711457822
PTGS2	729,5725561	675,5691176	418,1128192	179,1674309	166,107096	224,4034514	203,5044571	-1,680783986	0,272955632	-6,157718644	7,38003E-10	1,61085E-07	3,206021247
RUNX2	221,6778151	165,8170376	214,081804	85,58169382	98,76638142	70,62897474	194,6276718	-1,233184011	0,206961356	-5,958523044	2,54528E-09	5,2025E-07	2,350852482
RAB2B	579,4489724	663,2681504	524,6511817	264,7491248	326,8269349	337,9462335	344,8430108	-0,932958366	0,162342877	-5,746838927	9,09273E-09	1,62634E-06	1,909186931
HTN3	340,9348676	287,3505933	262,3255909	131,1431711	113,1324005	127,8473846	105,4554351	-1,258635176	0,221749076	-5,675943268	1,37926E-08	2,36829E-06	2,392692794
GABARAPL1	317,0834571	425,1214258	282,4271687	146,5355621	152,6389531	126,0593093	182,0142704	-1,272158427	0,224683127	-5,662011413	1,49609E-08	2,50216E-06	2,415226392
CD68	1922,844593	1848,589348	1764,918535	1102,095194	1134,017634	1188,176043	1373,383838	-0,69380846	0,124474406	-5,573904576	2,49092E-08	3,81882E-06	1,617547928
CDKN2B	243,4246894	304,0799087	247,2494075	115,7507801	110,4387719	75,09916301	193,1742342	-1,397314954	0,253606934	-5,509766355	3,5931E-08	5,25818E-06	2,634108833
RASSF9	68,74818317	63,47299065	43,21839236	15,39239097	15,26389531	21,45690372	36,56425396	-1,773458739	0,328103384	-5,405182709	6,47424E-08	8,77634E-06	3,418725856
STBD1	119,2570524	141,7071419	106,5383626	30,78478195	45,79168593	61,68859819	118,0188546	-1,434576418	0,265347553	-5,406405301	6,43022E-08	8,77634E-06	2,703027928
TMEM167B	454,5798234	499,9113062	474,3972371	259,207864	279,2394965	277,151673	394,4298556	-0,811107428	0,150855091	-5,376732205	7,58499E-08	9,86661E-06	1,754557743
TFAP2B	132,5857818	96,4395827	110,5586781	27,09060811	35,01717159	41,12573212	36,90146288	-1,726635278	0,321888918	-5,364071827	8,13665E-08	1,04784E-05	3,309550484
TUBB1	37,88165195	53,63221691	22,11173563	1,847086917	5,387257168	3,57615062	11,0613026	-3,45747446	0,648462341	-5,331804549	9,72416E-08	1,18139E-05	10,98508753
ANKRD5	159,2432406	183,0383917	161,8177016	62,80095518	89,78761947	68,84089943	85,33288631	-1,196658743	0,225099151	-5,316140644	1,05991E-07	1,25225E-05	2,292082136
DYX1C1	108,7343713	108,2485112	87,44186362	40,02021653	47,58743832	36,65554385	56,10919447	-1,301586178	0,250146228	-5,203301244	1,95779E-07	2,25111E-05	2,464997491
LRIG3	296,739607	333,1101913	294,4881154	155,7709967	177,7794865	132,3175729	169,6541498	-0,990340958	0,192584968	-5,142358566	2,71311E-07	3,01202E-05	1,986654449
PCDH10	65,24062281	111,2007433	52,26410239	17,23947789	12,57026673	20,56286606	23,78912636	-2,191351568	0,435877075	-5,027453132	4,97037E-07	5,08003E-05	4,567331698
HCFC1R1	406,8770024	422,6612323	331,6760344	213,0306911	183,1667437	156,4565896	363,0407502	-1,070252964	0,213736397	-5,007350071	5,51845E-07	5,5958E-05	2,099801516
ADD3	249,036786	281,9381678	239,2087763	141,609997	116,7239053	118,9070081	105,6684281	-1,028129249	0,205450238	-5,004273821	5,6073E-07	5,64147E-05	2,039378063
NEAT1	544,3733688	1015,56785	515,6054717	288,7612547	272,9543632	265,5291835	428,0019279	-1,329089179	0,266147934	-4,993798602	5,92031E-07	5,78727E-05	2,512440062
MMD	1106,284539	1123,324323	947,7893953	454,9990772	579,1301456	575,7602497	687,8988756	-0,982971272	0,197096788	-4,987251605	6,12443E-07	5,9301E-05	1,976531945
SLC44A2	397,7573455	354,2678548	466,356606	200,1010827	245,1202011	210,0988489	286,5066862	-0,8931125	0,18000141	-4,961697252	6,98799E-07	6,61701E-05	1,85717852
TMX4	2012,638138	2052,785403	1599,080517	751,7643752	1120,549491	868,1105629	951,0662649	-1,048853582	0,213680638	-4,908510144	9,17709E-07	8,44161E-05	2,068885182
LOC100506844	486,1478667	666,2203825	464,3464482	289,3769503	277,4437442	290,5622378	311,9653328	-0,918121333	0,187840486	-4,887771272	1,01984E-06	9,12048E-05	1,889652998
ID3	332,5167227	181,0702369	286,4474843	116,9821714	104,1536386	107,2845186	151,8276551	-1,27939172	0,262330084	-4,877030119	1,07695E-06	9,5648E-05	2,427366108
HOXA3	958,265492	729,2013345	719,6364868	281,9886027	245,1202011	354,0389113	303,6242865	-1,450220167	0,298787151	-4,853689858	1,21185E-06	0,000106165	2,732497482
DZIP3	237,1110807	300,6356379	162,8227805	90,50725893	123,0090387	96,55606673	210,6984144	-1,187151986	0,245211518	-4,841338589	1,28967E-06	0,00011001	2,277027927
LPL	370,3983746	408,3921104	307,554141	189,0185612	174,1879818	173,443305	135,2495395	-1,019234975	0,210529104	-4,841302022	1,28991E-06	0,00011001	2,02684389
FBXO36	429,3253888	472,3571397	562,8441796	195,7912132	234,3456868	172,5492674	240,9243378	-1,280256763	0,264587124	-4,838696398	1,30694E-06	0,000110728	2,428821999
NEK11	58,92701415	68,88541621	50,25394461	25,2435212	17,0596477	11,62248951	32,2147841	-1,702973534	0,353530354	-4,817050399	1,45696E-06	0,000122632	3,255713015
TRIL	58,22550207	48,71183004	43,21839236	6,772652029	5,387257168	15,19864013	14,79285615	-2,475824877	0,514736056	-4,809892079	1,51012E-06	0,000125466	5,562852595
DACT1	159,2432406	253,3999239	144,7313605	59,10678134	57,46407646	78,67531363	62,92989699	-1,521275828	0,316471801	-4,806986989	1,53222E-06	0,000126487	2,870447816
DZIP1	120,6600766	86,10677027	79,40123248	37,55743398	40,40442876	54,53629695	270,9552596	-1,124966133	0,236198499	-4,762799677	1,90925E-06	0,000150843	2,180964267
CLUAP1	455,2813355	428,0736579	428,1636081	255,5136902	278,3416204	261,0589952	300,0598103	-0,723550967	0,152167718	-4,754957079	1,98489E-06	0,000155862	1,651241307

CD200	247,6337618	243,5591502	141,7161238	80,65612871	79,91098133	82,25146425	72,97730084	-1,387259282	0,293069579	-4,733549242	2,20627E-06	0,000171798	2,615812771
BTBD19	105,928323	83,15453814	111,563757	41,25160781	26,03840965	45,5959204	50,44312347	-1,402087795	0,297875456	-4,706959798	2,51438E-06	0,000192165	2,642837637
RCAN2	86,285985	61,5048359	57,28949685	15,39239097	18,85540009	23,24497903	21,88109065	-1,847911793	0,393903567	-4,691279664	2,71502E-06	0,000204468	3,599787621
DNAL4	175,3780183	396,5831819	199,0056207	95,43282404	92,48124805	76,88723832	209,0945288	-1,5447656	0,330056666	-4,680304196	2,8645E-06	0,000214471	2,917566623
GRB14	336,024283	347,8713519	193,9802262	107,1310412	100,5621338	140,3639118	162,1349799	-1,340176818	0,286429106	-4,678912818	2,884E-06	0,000214683	2,531823471
WAC	1461,249648	2015,882502	1178,957541	698,8145502	808,0885752	676,7865047	1165,700554	-1,093607675	0,234711275	-4,659374259	3,17172E-06	0,000230765	2,13407026
HHLA3	166,9598734	139,2469485	175,8888061	86,8130851	76,31947655	56,32437226	87,19813448	-1,123301826	0,241453044	-4,65225788	3,2832E-06	0,000235275	2,178449737
OASL	187,3037235	169,7533471	193,9802262	92,97004149	87,99186708	92,97991611	77,69126753	-1,004735243	0,215982702	-4,65192459	3,28851E-06	0,000235275	2,006575225
RAB27B	125,5706611	155,4842252	108,5485204	59,72247698	58,36195265	59,00648522	81,16513641	-1,145302394	0,246713821	-4,64223037	3,44668E-06	0,000245108	2,211924886
IL12A	49,8073572	58,06056509	56,28441796	20,93365173	10,77451434	6,258263584	23,0740676	-2,071329171	0,446771942	-4,636211398	3,54853E-06	0,000249716	4,20273698
CETN2	1253,602075	2379,007053	1295,546692	609,5386826	844,9014992	700,9255214	1292,163129	-1,194684592	0,257820676	-4,633781157	3,59047E-06	0,000251294	2,288947847
BNIP2	1052,26811	1668,995227	933,7182908	567,6713791	434,5720782	598,1111911	733,393754	-1,192359811	0,25770168	-4,626899638	3,7118E-06	0,000253932	2,285262371
MEIS2	669,2425178	1040,169785	683,4536467	374,9586441	387,8825161	380,860041	352,1397386	-1,066893663	0,230623871	-4,62611984	3,7258E-06	0,000253932	2,094917838
STATH	293,9335587	251,4317692	179,9091217	91,12295457	95,17487664	117,1189328	85,82619962	-1,262868091	0,272866686	-4,628150511	3,68946E-06	0,000253932	2,399723342
YIPF5	3721,521548	3994,862102	3500,689781	2488,026077	2441,325373	2752,741939	2245,585365	-0,546701275	0,11817843	-4,62606649	3,72676E-06	0,000253932	1,460741885
SNCA	164,8553372	118,0892849	137,6958082	71,42069412	61,05558124	66,15878646	68,18245395	-1,075820932	0,234684986	-4,584106341	4,55932E-06	0,000302654	2,107921194
GBP2	581,5535087	832,5294588	530,6816551	299,2280805	279,2394965	324,5356687	369,0865601	-1,108892523	0,242031558	-4,58160305	4,61425E-06	0,000304214	2,156800182
GCNT3	21,74687427	19,68154749	25,1269723	5,541260751	8,080885752	6,258263584	653,3741572	-1,746200698	0,382627163	-4,563713366	5,02567E-06	0,000328531	3,354739395
KIAA0020	749,2148941	858,6075092	653,3012799	451,3049034	470,487126	506,9193503	1036,355419	-0,666184334	0,146560688	-4,545450377	5,48179E-06	0,000351216	1,586870429
INPP4A	206,2445495	276,5257422	203,0259362	131,1431711	117,6217815	109,0725939	121,1537475	-0,940609794	0,207004512	-4,543909631	5,52203E-06	0,000352043	1,919339328
IFIT1	426,5193405	581,0976896	335,69635	139,76291	118,5196577	161,8208155	198,34868	-1,67850101	0,371516759	-4,51796849	6,24358E-06	0,000388429	3,200951927
CNIH2	154,3326561	179,1020822	130,660256	60,33817262	65,54496221	72,41705005	74,24039925	-1,234723498	0,274785402	-4,493410096	7,00916E-06	0,000429829	2,353362394
PPP6R3	1938,97937	2120,194703	1842,309609	1047,298282	1297,431101	1219,467361	1513,454917	-0,728670893	0,16228313	-4,490121006	7,11827E-06	0,000434451	1,657111746
NRG1	634,8684262	489,0864551	649,2809644	334,9384276	331,3163158	362,9792879	308,6486862	-0,782699145	0,174585898	-4,483175074	7,35405E-06	0,000446724	1,720346468
SNAPC3	546,477905	538,7823625	525,6562606	317,0832541	347,4780873	341,5223842	595,6687273	-0,68080277	0,152681754	-4,458966132	8,23559E-06	0,000488746	1,603031494
LRRC46	70,15120732	40,83921104	43,21839236	17,85517353	17,95752389	20,56286606	43,65962859	-1,455535693	0,326607773	-4,456524962	8,32989E-06	0,000492075	2,742583781
TUBA1A	683,9742713	650,4751445	599,0270197	334,322732	431,8784496	381,7540786	280,1142236	-0,75511984	0,169555522	-4,453525489	8,44717E-06	0,000496724	1,687771784
C5orf28	350,0545245	307,0321408	280,4170109	174,8575615	189,4518771	199,370397	230,8202346	-0,738490172	0,166276243	-4,441345069	8,93983E-06	0,000518591	1,668428858
TWF1	2101,028659	2806,096633	1776,979481	1240,011017	890,6931851	1227,5137	1345,875397	-0,993368971	0,223967689	-4,435322676	9,19345E-06	0,000530911	1,990828535
CHEK1	173,9749941	144,659374	168,8532539	73,26778104	101,46001	99,23817969	177,4102773	-0,837789874	0,189603345	-4,418645002	9,93216E-06	0,000565958	1,787309987
FAM49B	547,1794171	470,8810237	453,2905804	293,0711242	297,1970204	353,1448737	441,9696588	-0,643886809	0,146822611	-4,385474439	1,15733E-05	0,000639662	1,562533163
IFIT2	445,4601665	241,0989567	444,2448703	152,0768228	186,7582485	164,5029285	179,7129057	-1,165861521	0,266386654	-4,37657631	1,20558E-05	0,00065948	2,243671588
MED12L	109,4358834	122,5176331	103,5231259	50,4870424	54,77044788	42,01976978	48,59697302	-1,190118914	0,272847912	-4,361839925	1,28973E-05	0,000694944	2,281715494
CHMP2A	1313,932113	1382,628711	1298,561929	761,6155054	944,5657568	809,1040777	1003,30401	-0,668726598	0,153501925	-4,356470427	1,32177E-05	0,000706294	1,589669219
LPAR1	320,5910174	469,4049076	295,4931943	176,0889527	152,6389531	181,4896439	276,9802861	-1,092061419	0,252037713	-4,332928611	1,47139E-05	0,000779775	2,131784225
CDH18	300,9486794	458,0880178	263,3306698	145,3041708	156,2304579	157,3506273	123,6247447	-1,160443645	0,269544967	-4,305195008	1,66839E-05	0,00086635	2,235261539
LRRC73	46,29979683	46,2516366	39,1980768	15,39239097	14,36601911	12,51652717	18,23508224	-1,638035889	0,380823108	-4,30130382	1,69796E-05	0,000874653	3,112418131
SIPA1L2	292,5305345	135,310639	247,2494075	99,12699787	85,2982385	89,40376549	99,87661731	-1,295988897	0,301266932	-4,301796053	1,69419E-05	0,000874653	2,455452471
PPIA	333,2182348	516,6406216	295,4931943	189,6342568	164,3113436	182,3836816	296,3254278	-1,097519826	0,256675012	-4,275912236	1,90356E-05	0,000972781	2,139865065
TUBB2A	684,6757834	705,0914388	717,626329	401,4335566	453,4274783	429,1380743	819,5339819	-0,715584136	0,167540094	-4,27112173	1,94492E-05	0,000986091	1,642147976
EIF4A2	3236,075194	3011,276766	2887,591657	1839,082874	2268,933144	1817,578552	2316,573178	-0,624881518	0,146664925	-4,260606385	2,03873E-05	0,001017627	1,542084176
RBP1	575,2399	407,9000717	406,0518724	217,3405606	251,4053345	243,1782421	175,0929984	-0,966052826	0,22711353	-4,253611946	2,1035E-05	0,001041878	1,953488586
MNS1	94,00261781	120,5494784	96,48757365	46,79286856	45,79168593	50,96014633	58,02884579	-1,122393214	0,264451238	-4,244235057	2,1934E-05	0,001065911	2,177078177
ZDHHC3	1081,731617	581,5897283	1031,210943	448,2264252	521,6660691	387,1183046	675,267729	-0,988041852	0,233546449	-4,230601056	2,33068E-05	0,001124137	1,983491001
DPCD	595,5837501	706,5675549	613,0981242	420,5201214	404,9421638	425,5619237	454,4559579	-0,61599233	0,145649494	-4,229278902	2,34442E-05	0,001126544	1,532611812
CEP120	408,2800266	489,0864551	372,884269	210,5679085	205,6136486	282,5158989	276,3927579	-0,866767571	0,205399591	-4,219908934	2,44401E-05	0,001165702	1,823572516
ZMYND10	84,88296085	30,9984373	59,29965464	17,23947789	17,0596477	9,834414204	22,14500139	-1,973595609	0,467823493	-4,218675719	2,45741E-05	0,00116777	3,927457352
BSCL2	321,2925295	367,0608607	352,7826912	206,2580391	208,3072772	230,661715	241,1753968	-0,692134249	0,164148089	-4,216523355	2,48098E-05	0,00117033	1,615671893
TCTN2	133,2872939	210,5925581	169,8583328	83,7346069	81,70673372	80,46338894	148,8709135	-1,065573841	0,252698335	-4,216782198	2,47813E-05	0,00117033	2,093002218
DLX2	422,310268	279,4779743	314,5896933	187,1714742	167,9028484	164,5029285	193,8696529	-0,965777775	0,229337905	-4,211156354	2,54067E-05	0,001189772	1,953116187

MEX3C	549,9854654	542,2266333	527,6664184	323,8559061	323,2354301	299,5026144	449,1799566	-0,774909478	0,184957046	-4,189672653	2,79357E-05	0,001280271	1,711082686
IAH1	611,0170157	486,6262617	672,3977789	334,9384276	365,4356112	297,7145391	619,7161842	-0,824106449	0,196985498	-4,18358944	2,86942E-05	0,001292042	1,770438156
TP53	4712,056596	4039,145584	4407,270942	2492,951642	2701,70947	2810,854387	3533,628775	-0,716988192	0,171343806	-4,184500219	2,85794E-05	0,001292042	1,643746922
FAM76A	152,929632	174,673734	135,6856504	80,65612871	96,07275283	77,78127597	118,3330841	-0,873403702	0,20935472	-4,171884455	3,02091E-05	0,001346133	1,831979935
SMR3B	291,8290224	282,9222452	230,1630663	141,609997	155,3325817	125,1652717	102,2518487	-0,933868587	0,223838802	-4,172058556	3,0186E-05	0,001346133	1,91039185
MIS18BP1	388,6376885	580,6056509	401,026478	269,6746899	253,2010869	253,906694	476,0999546	-0,821712872	0,198288733	-4,144022	3,41267E-05	0,001510253	1,767503255
CRISPLD1	49,8073572	45,26755923	55,27933907	26,47491248	17,95752389	19,66882841	163,3319044	-1,198258574	0,289806467	-4,134685426	3,55441E-05	0,001556929	2,294625276
POP5	853,7401931	889,6059465	711,5958557	385,42547	496,5255357	582,912551	507,5102642	-0,74880028	0,181296459	-4,130253192	3,62364E-05	0,001581872	1,680394863
STPG1	311,4713605	361,1563964	335,69635	147,7669534	211,8987819	154,6685143	256,8277694	-0,974470309	0,236625922	-4,118189164	3,81861E-05	0,001661354	1,964919637
CKAP2	453,8783113	402,9796848	439,2194759	272,7531681	299,890649	288,7741625	565,5707897	-0,589410915	0,143389723	-4,110552003	3,94714E-05	0,001705749	1,504632246
PALM2	167,6613855	235,1944925	116,5891515	72,6520854	69,13646699	71,52301239	86,88936316	-1,290527525	0,31429052	-4,106161158	4,02289E-05	0,001726892	2,446174842
ACSM3	28,761995	40,83921104	35,17776123	13,54530406	14,36601911	8,940376549	33,86998814	-1,504567458	0,366527644	-4,104922191	4,04451E-05	0,001730405	2,83739589
MARK4	484,0433305	532,8778983	516,6105506	324,4716017	332,214192	299,5026144	410,979959	-0,681418596	0,16623307	-4,099175921	4,14624E-05	0,00176222	1,603715907
RAB3A	230,09596	264,224775	243,2290919	123,1391278	152,6389531	126,953347	127,0547018	-0,876614553	0,214127965	-4,093881674	4,24211E-05	0,001791143	1,836061715
CABIN1	279,2018051	295,2232123	243,2290919	150,8454315	184,9624961	150,198326	293,7617501	-0,756548545	0,184854554	-4,092669236	4,26436E-05	0,001794654	1,689444017
KIAA0513	107,3313472	178,1180048	117,5942304	73,26778104	59,25982885	67,94686177	229,8755961	-1,012582935	0,248338309	-4,077433486	4,55356E-05	0,001897757	2,017519944
TMEM60	185,1991873	171,7215018	207,0462518	104,052563	126,6005435	105,4964433	229,3552445	-0,744489944	0,182828403	-4,072069396	4,65973E-05	0,001935742	1,675381844
KCNK3	434,9374854	712,4720191	479,4226316	297,3809936	275,6479918	283,4099366	1244,653188	-0,927549601	0,228782503	-4,054285581	5,02878E-05	0,00206503	1,902042657
SMA5	1527,191783	1887,460404	1498,572628	1033,752978	1145,690024	1124,69937	815,9066393	-0,574095184	0,141769022	-4,049510796	5,13248E-05	0,002098289	1,488743474
SERTAD2	175,3780183	169,7533471	167,848175	81,27182434	89,78761947	82,25146425	177,165294	-1,019527574	0,251936821	-4,046758907	5,19317E-05	0,00211638	2,027255004
ARMC1	1210,809838	1183,845081	1169,911831	677,8808985	881,7144232	630,2965467	1279,388524	-0,703560317	0,173949299	-4,044628638	5,24061E-05	0,002128978	1,628518735
MAP1LC3A	82,07691256	73,80580309	85,43170584	25,85921684	29,62991442	40,23169447	28,20007113	-1,340402135	0,332384938	-4,032680131	5,51443E-05	0,002220551	2,532218916
PDE5A	86,98749707	87,09084764	38,1929979	22,78073864	25,14053345	9,834414204	27,57359316	-1,879359689	0,465886225	-4,033945604	5,4848E-05	0,002220551	3,679117338
PLA2G4A	55,41945378	60,02871984	56,28441796	27,09060811	20,65115248	19,66882841	42,14567536	-1,337043161	0,331563635	-4,032538608	5,51775E-05	0,002220551	2,526330102
TSPYL2	218,8717668	334,0942686	184,9345162	126,217606	87,99186708	99,23817969	116,324614	-1,235806152	0,306523739	-4,03168171	5,53792E-05	0,002221722	2,35512911
CMAS	930,9065211	1349,662119	959,850342	621,2368997	719,1988319	594,5350405	748,9815971	-0,745752207	0,185542249	-4,019312095	5,83683E-05	0,002333825	1,676848334
PLEKHG4B	33,67257951	33,95066942	24,12189341	17,85517353	4,489380973	5,364225929	102,7281168	-1,653864118	0,411548576	-4,018636475	5,85359E-05	0,002333825	3,146753386
ARL14	619,4351606	870,9084764	657,3215955	471,0071638	395,9634019	414,8334719	309,1707549	-0,745235919	0,186220723	-4,001895756	6,2837E-05	0,002455122	1,676248359
FBXW4	404,7724662	669,6646533	481,4327894	273,9845593	281,9331251	255,6947693	495,1336794	-0,940686147	0,234925924	-4,004181954	6,22325E-05	0,002455122	1,919440909
NCBP2	1236,064273	1210,415171	1048,297285	777,623592	849,3908802	823,4086801	834,8138732	-0,514170632	0,128721848	-3,994431724	6,48496E-05	0,002515461	1,428172884
GLCCI1	89,09203329	115,6290915	70,35552245	30,16908631	41,30230496	31,29131792	44,6391845	-1,433045096	0,35894768	-3,992350904	6,54215E-05	0,002530023	2,700160371
OPN3	237,8125928	221,4174093	211,0665674	139,76291	149,0474483	156,4565896	385,3053066	-0,595267459	0,149212527	-3,989393334	6,62425E-05	0,002554104	1,510752636
RABGGTA	420,2057318	747,4067659	516,6105506	272,7531681	334,0099444	264,6351458	347,7401054	-0,953368308	0,239047084	-3,98820305	6,65757E-05	0,002559288	1,936388334
TMCO3	152,929632	144,659374	111,563757	62,80095518	72,72797177	92,08587845	268,5369202	-0,861965714	0,217341532	-3,965950305	7,31041E-05	0,002752733	1,817513038
ST6GALNAC5	284,1123896	251,9238079	202,0208573	136,0687362	124,8047911	142,1519871	121,2412111	-0,875813392	0,221478848	-3,954388428	7,67307E-05	0,002880867	1,835042392
CLDND1	1740,451454	3187,918655	1902,614343	1236,316843	978,6850522	1116,653031	1214,256298	-1,036147973	0,262423858	-3,948375657	7,86833E-05	0,002928564	2,050744803
PEG10	1266,229292	995,886303	1088,50044	549,20051	773,0714036	581,1244757	453,9072678	-0,816570918	0,207012142	-3,944555672	7,99481E-05	0,002958538	1,761214855
POU2F1	179,5870907	212,5607129	164,8329383	107,7467368	105,949391	119,8010458	183,3443292	-0,748109881	0,189874019	-3,940032894	8,14704E-05	0,003006236	1,679590905
CSN3	172,57197	178,6100435	133,6754927	83,11891126	77,21735274	84,93357721	60,84394561	-0,987656476	0,25147125	-3,9275125	8,5829E-05	0,003122332	1,982961238
BIN3	406,8770024	398,059298	420,1229769	225,3446039	281,9331251	210,0988489	365,2143335	-0,772629564	0,196960018	-3,922773626	8,75354E-05	0,003166519	1,70838077
APOO	394,9512972	626,8572875	406,0518724	281,9886027	271,1586108	255,6947693	308,7596608	-0,822821115	0,210347898	-3,91171541	9,16429E-05	0,003278269	1,76886153
ATP1B1	498,775084	619,4767072	430,1737659	275,8316463	342,9887064	305,760878	687,1307357	-0,748959489	0,192009399	-3,900639724	9,59388E-05	0,003422439	1,680580313
HTR7	96,10715402	112,1848207	77,3910747	28,93769503	27,83416204	38,44361916	35,77758139	-1,592255075	0,409323966	-3,889962979	0,00010026	0,003556865	3,015202873
GPAM	615,9276002	752,3271528	537,7172073	428,5241647	379,8016304	375,4958151	318,7795021	-0,688690264	0,17711501	-3,888378873	0,000100916	0,00357032	1,61181958
RBM47	184,4976752	153,0240317	143,7262816	84,96599818	101,46001	108,1785562	185,2472996	-0,717763583	0,184958617	-3,880671228	0,000104169	0,003635795	1,644630607
SCAMP4	1165,211554	1253,714575	1224,186091	799,788635	890,6931851	803,7398517	1201,47294	-0,547142172	0,141141485	-3,876551045	0,000105948	0,003667723	1,461188366
SSH1	492,4614754	647,5229124	463,3413693	265,3648204	323,2354301	299,5026144	399,0201126	-0,855812833	0,220905746	-3,874108528	0,000107016	0,003690378	1,80977812
CCDC104	432,8329491	681,4735818	410,072188	262,2863422	258,5883441	297,7145391	367,3795837	-0,900685256	0,23300824	-3,865465256	0,000110878	0,003787486	1,866952544
C1orf233	621,5396968	550,0992523	574,9051263	307,2321238	416,6145543	371,9196644	290,3814035	-0,674909964	0,174793473	-3,861185151	0,000112838	0,003833218	1,596497135
SERPINB6	1549,64017	2359,325505	1465,405025	869,9779379	1073,859929	988,8056463	1210,768158	-0,875189819	0,226840831	-3,858167042	0,000114241	0,003852787	1,834249407

C2CD3	484,0433305	706,0755162	472,3870793	284,4513852	353,7632207	318,2774051	369,4737327	-0,801868169	0,208619186	-3,843693307	0,000121196	0,004036862	1,743357167
WIPI1	249,738298	283,4142838	259,3103542	181,6302135	153,5368293	167,1850415	155,5508487	-0,656404409	0,170784852	-3,843458015	0,000121313	0,004036862	1,576149529
COPG1	3432,498574	3772,460615	3152,932485	1965,916175	2499,687326	2174,299577	2676,404881	-0,642154805	0,167112684	-3,842645506	0,000121715	0,004039816	1,560658414
KLHDC3	813,0524928	1042,13794	782,956457	477,7798158	615,9430696	515,8597269	570,3917768	-0,715535514	0,186493299	-3,836789409	0,000124653	0,004096713	1,642092633
TMEM67	197,1248926	185,0065464	155,7872283	76,34625923	121,2132863	97,45010438	120,6832944	-0,878014959	0,228846781	-3,836693508	0,000124702	0,004096713	1,837844823
TRIM5	260,2609791	374,9334797	224,132593	137,3001275	181,3709913	137,6817989	265,2145953	-0,920577197	0,240120667	-3,833810768	0,000126173	0,004124007	1,892872447
SMAD7	429,3253888	447,7552054	358,8131645	240,7369948	266,6692298	236,0259409	376,1974726	-0,736426476	0,192426152	-3,827060254	0,000129683	0,00421731	1,66604397
BTC	33,67257951	54,1242556	46,23362904	13,54530406	22,44690487	11,62248951	24,11092543	-1,507699181	0,394506478	-3,821734918	0,000132516	0,004298595	2,843561843
ACVR1	482,6403063	629,317481	428,1636081	335,5541232	335,8056968	286,9860872	449,9800259	-0,687519902	0,180612953	-3,806592438	0,000140895	0,004547472	1,610512549
LOC100134868	16,13477768	18,69747011	12,06094671	5,541260751	6,285133363	1,78807531	35,70075584	-1,786618189	0,470038637	-3,801002829	0,000144112	0,004630167	3,450052198
PCDH18	51,91189341	29,52232123	29,14728787	11,0825215	13,46814292	6,258263584	15,46305892	-1,840077517	0,484636007	-3,796823781	0,000146562	0,004662878	3,58029265
TBC1D8	268,679124	267,1770072	243,2290919	177,9360397	169,6986008	144,8341001	145,9608226	-0,660902001	0,174048997	-3,797218101	0,000146329	0,004662878	1,581070831
SLC4A4	37,88165195	35,42678548	39,1980768	15,39239097	17,95752389	16,09267779	140,380926	-1,187244325	0,312888705	-3,794462073	0,000147964	0,004687126	2,277173672
ZNF720	56,12096585	89,55104108	57,28949685	24,01212992	26,93628584	19,66882841	46,54076777	-1,529977992	0,40324358	-3,794178179	0,000148133	0,004687126	2,887814337
SIX1	881,0991639	1197,130126	892,5100563	593,530596	482,1595165	608,839643	456,285883	-0,819132917	0,216743445	-3,779274233	0,000157286	0,004952398	1,764345274
MIER1	1374,262151	2326,850952	1325,699059	916,1551108	929,3018615	938,7395376	1068,978984	-0,853568612	0,22597416	-3,777284145	0,000158548	0,004975483	1,806965063
ZNF362	395,6528093	390,6787177	340,7217445	174,8575615	263,9756012	206,5226983	320,4281482	-0,809330387	0,215074348	-3,763026103	0,00016787	0,005234445	1,752397895
SAMD15	129,0782215	109,2325886	84,42662694	43,09869473	58,36195265	51,85418398	51,50176701	-1,085803231	0,288687559	-3,761170845	0,00016912	0,005253789	2,122556903
UNC5D	470,7146011	773,4848163	393,9909257	244,4311687	285,5246299	250,3305434	201,5397238	-1,073174322	0,285396404	-3,760293776	0,000169714	0,005253789	2,104057779
C20orf96	97,51017817	131,3743295	93,47233697	41,86730345	63,74920982	61,68859819	83,16406506	-0,967091609	0,25772396	-3,752431905	0,000175127	0,005408371	1,954895661
RTL1	131,8842698	162,3727668	150,7618338	61,5695639	72,72797177	88,50972783	55,65217782	-1,004888922	0,268313424	-3,745205535	0,000180246	0,00552669	2,006788982
SLC1A1	84,88296085	59,53668116	67,34028578	33,2475645	26,03840965	46,48995805	77,43497993	-1,001727334	0,267391111	-3,746300057	0,000179462	0,00552669	2,002396028
SLC31A1	873,3825311	1469,719559	974,9265254	525,18838	648,2666126	684,8328436	907,6370872	-0,838538186	0,223869932	-3,745648992	0,000179928	0,00552669	1,788237288
GMPPB	217,4687427	227,3218735	222,1224352	136,6844319	155,3325817	144,8341001	174,6723884	-0,613860534	0,164071754	-3,74141507	0,000182987	0,005570938	1,530348823
MAP1B	3899,004103	3561,868057	4689,698111	2742,924072	2848,961166	2741,11945	3147,661495	-0,543584482	0,145472941	-3,736670751	0,000186473	0,005663673	1,457589511
NDUFV2	886,0097484	1210,907209	760,8447214	444,5322513	584,5174027	575,7602497	723,7861266	-0,835312271	0,224192598	-3,725869082	0,000194643	0,005856584	1,784243196
UPF3A	176,0795304	187,9587785	162,8227805	110,825215	105,0515148	111,7547069	188,6179942	-0,688924333	0,184969413	-3,724531112	0,000195679	0,005874006	1,61208111
AKIRIN2	333,9197468	445,7870506	369,8690323	200,1010827	245,1202011	261,9530329	254,6781751	-0,70649666	0,189934551	-3,719684781	0,000199472	0,005954666	1,631836664
MYL9	2554,90697	2873,505933	2505,661678	1586,031966	1887,335761	1746,949578	1529,607685	-0,604613427	0,162559814	-3,719328971	0,000199753	0,005954666	1,520571259
CPNE8	92,59959366	55,60037166	84,42662694	33,2475645	40,40442876	42,91380743	80,35159599	-0,993908004	0,267439399	-3,716385871	0,000202093	0,005959056	1,991572506
EAF1	687,4818317	809,8956792	634,204781	364,4918183	457,0189831	423,7738484	507,3061681	-0,778042538	0,209428185	-3,715080366	0,000203139	0,005959056	1,714802633
LPCAT2	138,8993905	141,2151032	154,7821494	67,11082465	93,37912425	96,55606673	199,7825823	-0,766532184	0,206320277	-3,715253746	0,000203	0,005959056	1,701175737
TLE4	623,6442331	568,7967224	498,5191305	379,2685136	348,3759635	357,615062	443,3491811	-0,64044295	0,172304617	-3,716922735	0,000201664	0,005959056	1,558807686
LYPD1	157,1387044	176,14985	140,7110449	100,3583892	88,88974327	101,9202927	172,0582849	-0,708289264	0,190749983	-3,713181274	0,00020467	0,005990324	1,633865543
DPY30	1435,995214	2440,01985	1534,755468	967,2578488	1038,842757	1143,474161	1072,972144	-0,781986474	0,210854376	-3,708656609	0,000208362	0,006070776	1,719496852
CBX5	1689,241072	1947,981163	1634,258279	1110,099237	1255,23092	1149,732424	3518,047135	-0,585671238	0,157983321	-3,707171339	0,000209587	0,006087157	1,50073707
SYT1	74,36027976	61,01279722	64,3250491	32,63186887	20,65115248	25,03305434	23,16785015	-1,336933706	0,360691021	-3,706589929	0,000210069	0,006087157	2,526138441
PMF1	425,8178284	490,5625712	510,5800772	309,0792108	343,8865826	297,7145391	561,9659686	-0,585652956	0,15817675	-3,702522382	0,000213467	0,006122543	1,500718053
SNAP23	1452,831504	1557,794484	1207,09975	821,3379824	1011,008595	934,2693494	969,9649672	-0,610313498	0,164793897	-3,703495759	0,000212649	0,006122543	1,526590901
SLMO2	3475,992323	4408,666638	3677,583667	2378,432253	2755,582041	2731,285036	2134,795156	-0,556479459	0,150366593	-3,700818442	0,000214905	0,006150109	1,470676013
ZNF608	48,40433305	58,06056509	59,29965464	36,3260427	25,14053345	21,45690372	98,36467596	-0,970248966	0,262376594	-3,697924993	0,000217369	0,00618788	1,959178661
EPC2	615,2260882	768,0723908	507,5648406	382,9626874	407,6357924	418,4096225	435,473026	-0,649581754	0,176218135	-3,686236687	0,000227595	0,006427554	1,568713351
ID1	4500,199949	7491,781052	4710,804768	2901,773546	2417,980592	2936,019659	2617,29379	-1,016750466	0,277404725	-3,665224032	0,000247122	0,006920661	2,023356404
TLK2	618,0321365	720,8366768	575,9102052	378,652818	460,6104879	384,4361916	476,2228762	-0,648900976	0,177388819	-3,658071467	0,00025412	0,00707254	1,567973281
C19orf43	4952,675237	7221,651813	5011,323356	3444,8171	3738,756475	3430,422482	4200,76193	-0,695574659	0,190244346	-3,656217243	0,000255964	0,007104117	1,619529401
ZEB2	235,0065445	448,2472441	202,0208573	129,2960842	119,4175339	160,9267779	175,3907193	-1,117559159	0,305777973	-3,654805964	0,000257377	0,007106658	2,169795623
MNAT1	813,7540049	1033,773282	829,1900861	482,0896853	642,8793554	560,5616096	594,0003057	-0,669902248	0,183465008	-3,651389749	0,000260825	0,007177148	1,590965166
CCDC103	150,8250957	141,7071419	163,8278594	70,80499848	62,85133363	115,3308575	97,57061848	-0,876501888	0,240425112	-3,645633687	0,000266734	0,007324087	1,835918337
FBXW7	408,2800266	366,0767833	366,8537956	230,270169	275,6479918	272,6814847	313,7518349	-0,555242804	0,152395604	-3,643430573	0,000269028	0,007355723	1,469415914
TNPO2	570,3293155	512,7043121	770,8955103	390,9667307	408,5336686	338,8402712	515,1623023	-0,699985132	0,192408316	-3,638019126	0,000274743	0,007480213	1,624488052

IRF7	709,9302181	948,650589	711,5958557	380,4999049	377,1080018	478,3101454	408,6720144	-0,940836551	0,258775871	-3,63571978	0,000277206	0,007531335	1,919641026
HSPH1	1556,65529	1666,042995	1504,603102	938,3201538	1074,757805	1252,546754	1711,37132	-0,535244599	0,147339912	-3,632719691	0,00028045	0,0075891	1,449187833
SELK	544,3733688	867,4642056	649,2809644	376,8057311	480,3637642	411,2573212	487,4736386	-0,703568287	0,193809948	-3,630196967	0,000283205	0,007645942	1,628527732
C11orf70	121,3615887	125,4698652	110,5586781	65,87943337	64,64708602	46,48995805	44,53388412	-1,011658123	0,27920157	-3,623396971	0,000290759	0,00779847	2,016227066
GDI1	2101,028659	1907,141952	2200,117695	1314,510189	1396,197483	1274,897696	1643,267986	-0,63907924	0,177234867	-3,605832475	0,000311154	0,008279006	1,557334917
KLHL14	202,7369891	179,5941208	133,6754927	81,88751998	69,13646699	118,9070081	189,432994	-0,941825321	0,261337755	-3,603862446	0,000313523	0,008324844	1,920957128
LURAP1L	324,0985778	592,4145794	342,7319022	194,5598219	228,0605534	213,6749995	163,5512821	-0,988804133	0,274523234	-3,601895985	0,000315905	0,008347628	1,984539301
ANKRD13D	69,44969524	97,91569876	93,47233697	47,4085642	44,89380973	35,7615062	64,1220645	-1,021354288	0,284038076	-3,595835823	0,000323352	0,008480902	2,029823503
ZNF485	86,285985	120,0574397	85,43170584	57,25969442	49,38319071	51,85418398	62,66707188	-0,886385158	0,246866641	-3,59054247	0,00032999	0,008619913	1,848538585
TNIK	155,7356802	220,4333319	169,8583328	126,217606	113,1324005	92,97991611	230,5298362	-0,717067276	0,199911338	-3,586926509	0,000334599	0,008722592	1,643837029
CLCN3	230,7974721	278,9859357	199,0056207	123,1391278	137,3750578	143,0460248	244,8390954	-0,819547063	0,228830526	-3,581458638	0,000341681	0,008853463	1,764851827
FAM105A	239,215617	298,1754445	246,2443286	137,3001275	185,8603723	174,3373427	178,0935182	-0,664722162	0,185713677	-3,579284914	0,000344536	0,008891644	1,58526295
PIM1	225,8868876	340,9828103	203,0259362	118,8292583	155,3325817	129,63546	145,3261613	-0,938518121	0,26251461	-3,575108152	0,000350083	0,008998744	1,916558614
HPS1	73,65876768	150,0717996	96,48757365	54,18121623	39,50655257	61,68859819	176,0391989	-1,05032086	0,293954626	-3,573071372	0,000352819	0,009050991	2,070990392
ZBTB4	932,3095452	1407,230645	838,2357961	602,7660306	671,6113936	599,0052288	753,5990815	-0,764427477	0,214419195	-3,56510749	0,000363707	0,009274891	1,69869575
ESRG	404,7724662	444,8029733	367,8588745	245,0468643	199,3285152	275,3635977	161,7470859	-0,759690086	0,213245622	-3,562511989	0,000367323	0,009348591	1,693126875
SSFA2	1294,289775	1514,003041	1220,165775	959,2538055	912,2422138	846,6536592	910,396401	-0,568204206	0,159612323	-3,559901871	0,000370993	0,009404829	1,48267686
SLC25A22	893,7263812	1009,663386	1006,083971	632,3194212	659,0411269	672,3163165	761,4635475	-0,56751117	0,159645585	-3,554819068	0,000378239	0,009550913	1,481964789
PIK3CG	15,43326561	30,01435992	23,11681452	5,541260751	13,46814292	7,152301239	84,09545286	-1,448547802	0,407671101	-3,553226605	0,000380537	0,009582524	2,729331821
PRTG	89,09203329	85,61473158	97,49265254	40,63591217	61,95345743	49,17207102	82,67457728	-0,850342807	0,239336279	-3,552920643	0,000380979	0,009582524	1,802929279
C16orf55	178,1840666	179,5941208	160,8126228	67,11082465	87,09399088	92,97991611	149,8303544	-1,074766845	0,302907102	-3,548173151	0,000387913	0,009700088	2,10638163
NHSL1	187,3037235	232,7342991	162,8227805	116,9821714	113,1324005	110,8606692	124,9799648	-0,779209598	0,220049376	-3,541067068	0,000398512	0,009907415	1,716190374
ZMYM6	264,4700516	240,1148794	235,1884608	160,6965618	167,9028484	168,0790791	187,7069134	-0,576793359	0,162934797	-3,540025647	0,000400088	0,009927428	1,491530375
PCDHB2	153,631144	223,8776027	144,7313605	93,58573712	94,27700044	104,6024056	107,7195064	-0,845545772	0,239078861	-3,536681442	0,000405188	0,010034638	1,796944402
SKAP2	460,19192	610,6200109	325,6455611	227,1916908	258,5883441	249,4365057	385,5547547	-0,929619018	0,262909958	-3,535883642	0,000406414	0,010045672	1,904772924
RFC5	332,5167227	314,4127211	275,3916165	183,4773004	237,0393154	202,9465477	278,9949333	-0,571989238	0,162029576	-3,530153279	0,000415319	0,010207019	1,486571896
C10orf128	21,0453622	15,74523799	12,06094671	2,462782556	1,795752389	1,78807531	4,574846429	-2,99769543	0,850332975	-3,525319516	0,000422972	0,010335222	7,987230951
KCTD3	759,7375752	686,8860074	780,9462992	515,9529455	548,602355	513,1776139	764,3253297	-0,496509064	0,140860205	-3,524835579	0,000423746	0,010335222	1,41079568
KL	36,4786278	51,17202347	27,13713009	11,69821714	11,67239053	11,62248951	12,48173988	-1,726876389	0,490318596	-3,521947576	0,000428389	0,010428718	3,310103641
ENPP4	141,0039267	185,9906238	118,5993093	96,66421532	79,01310513	81,35742659	119,7664467	-0,79895672	0,227173926	-3,516938468	0,000436555	0,010527187	1,73984251
H1F0	1910,217375	1027,868818	1573,953545	762,8468967	984,0723094	823,4086801	753,1966944	-0,811604331	0,230725525	-3,517618309	0,000435438	0,010527187	1,755162164
IRF9	377,4134954	246,5113823	278,4068531	127,4489973	163,4134674	216,3571125	183,9977295	-0,835115499	0,237520879	-3,515966689	0,000438156	0,010527187	1,783999857
RNF146	125,5706611	158,4364573	134,6805716	81,88751998	79,01310513	85,82761487	162,6931738	-0,767411522	0,218248927	-3,516221288	0,000437736	0,010527187	1,702212938
ENPP2	68,0466711	69,86949359	54,27426018	24,01212992	35,01717159	39,33765681	79,83363318	-0,993627616	0,282980717	-3,511290899	0,000445936	0,010654481	1,991185482
LIN37	313,5758967	417,7408455	280,4170109	185,940083	208,3072772	185,0657946	228,7846005	-0,808952626	0,230479083	-3,509874366	0,000448318	0,010660567	1,751939101
TIGD2	138,1978784	134,3265616	139,705966	73,88347668	77,21735274	91,1918408	86,26800744	-0,769570012	0,219268137	-3,50972113	0,000448577	0,010660567	1,704761612
LRP12	206,2445495	229,2900283	200,0106995	144,0727795	121,2132863	134,9996859	237,9808009	-0,667059178	0,190342562	-3,504519277	0,000457432	0,010848647	1,587832992
SLC39A7	1964,233805	2325,866875	1844,319767	1286,18819	1371,954825	1280,261922	1472,047746	-0,639978952	0,182757355	-3,501795871	0,000462134	0,010919919	1,558306425
ZNF768	220,976303	299,6515605	235,1884608	134,2216493	152,6389531	185,9598322	180,9880826	-0,685576158	0,195807206	-3,501281542	0,000463026	0,010920978	1,608344165
JUP	1157,494921	1568,619335	1011,109366	692,6575938	774,867156	733,110877	770,9368899	-0,765594328	0,219181323	-3,492972471	0,000477676	0,011224935	1,700070209
TOR1A	1353,216789	1466,767327	1316,653349	967,2578488	1070,268424	986,1235333	1155,57106	-0,453401346	0,129821573	-3,492496172	0,000478528	0,011224935	1,369264679
OSCP1	125,5706611	134,8186003	107,5434415	61,5695639	87,09399088	58,11244757	69,078271	-0,842036335	0,241256265	-3,49021542	0,000482631	0,011268467	1,792578546
BAMBI	77,86784012	95,94754401	82,41646916	41,86730345	46,68956212	50,96014633	61,9543257	-0,887877088	0,254441109	-3,489519016	0,000483891	0,011268614	1,850451199
RABL5	605,4049191	691,8063943	598,0219409	336,7855145	384,2910113	387,1183046	444,3258128	-0,775844286	0,222434494	-3,487967503	0,000486707	0,011313747	1,712191758
SMG6	352,8605728	408,3921104	316,599851	184,0929961	263,077725	218,1451878	303,0355691	-0,702594535	0,20156961	-3,485617377	0,000491003	0,011393033	1,627428922
CCL5	108,7343713	91,02715714	81,41139027	38,78882526	52,97469549	42,91380743	34,65418724	-1,070340187	0,308000147	-3,475128818	0,000510608	0,011721232	2,099928471
CHN2	34,37409159	33,95066942	38,1929979	13,54530406	11,67239053	8,940376549	11,72298584	-1,624981929	0,467490661	-3,475966615	0,000509016	0,011721232	3,084383017
DCAF5	616,6291123	648,0149511	522,6410239	351,5622099	394,1676495	448,8069028	523,8525556	-0,585449845	0,168454172	-3,475425018	0,000510045	0,011721232	1,500506788
GNAI1	122,7646128	159,9125734	99,50281033	62,80095518	74,52372416	80,46338894	141,1623042	-0,828879161	0,238491588	-3,475506908	0,000509889	0,011721232	1,776304803
RBM10	830,5902946	1274,872239	909,5963974	480,858294	632,1048411	556,985459	679,9394422	-0,854084644	0,245895618	-3,473362607	0,00051398	0,011777651	1,807611505

ZNF532	409,6830507	447,7552054	405,0467936	249,9724294	296,2991442	244,9663174	523,0799878	-0,676137552	0,194718673	-3,472381678	0,000515862	0,011786032	1,597856171
ACPL2	669,9440299	678,029311	445,2499492	328,7814712	355,5589731	399,6348317	382,9074203	-0,729944883	0,210328723	-3,470495491	0,000519499	0,011840901	1,658575726
LRCH2	199,2294288	154,5001478	139,705966	105,2839543	62,85133363	61,68859819	60,58427972	-1,094812108	0,315540574	-3,46963972	0,000521157	0,011857701	2,135852631
NDNF	32,26955537	38,3790176	21,10665674	9,235434585	6,285133363	8,940376549	9,684681184	-1,91072658	0,551959186	-3,461717147	0,000536741	0,012126578	3,759984149
CSRP2	157,8402165	312,4445664	186,9446739	101,5897804	130,1920482	111,7547069	205,13254	-0,944077859	0,272807382	-3,460602315	0,000538968	0,012137137	1,923958738
DDB2	296,739607	391,1707564	370,8741112	201,9481696	248,7117059	160,9267779	423,9713577	-0,792284825	0,229671525	-3,449643251	0,000561328	0,012571791	1,731815003
NR3C2	34,37409159	33,95066942	29,14728787	14,77669534	10,77451434	12,51652717	17,85987478	-1,348767072	0,391448535	-3,445579563	0,000569836	0,012718112	2,546943705
SLC52A3	167,6613855	172,2135405	172,8735695	99,74269351	109,5408958	89,40376549	67,71316727	-0,779462905	0,226525989	-3,440942505	0,000579692	0,01286645	1,716491728
SREBF1	437,0420216	321,3012628	433,1890025	214,2620824	263,9756012	269,1053341	339,4738532	-0,672624621	0,195546115	-3,439723775	0,000582308	0,012884819	1,593970159
ABLIM1	25,95594671	40,83921104	31,15744566	12,92960842	17,0596477	16,09267779	141,1293622	-1,121071949	0,326023942	-3,438618469	0,000584691	0,012915345	2,175085254
ULBP2	392,846761	719,3605607	518,6207084	326,3186887	307,0736586	286,0920496	262,0061816	-0,827801519	0,241157821	-3,432613193	0,000597794	0,013159649	1,774978463
RARRES3	461,5949441	413,3124973	415,0975825	249,3567338	289,1161347	215,4630748	375,0073235	-0,775068178	0,225856687	-3,431681329	0,000599852	0,013182412	1,711270919
TAB2	1995,801848	2055,737635	1658,380172	1172,284497	1171,728434	1373,241838	1586,588966	-0,620076884	0,180814127	-3,429360819	0,000605005	0,013272998	1,536957086
ASCC1	32,97106744	32,96659204	43,21839236	19,08656481	19,75327628	8,940376549	45,44341639	-1,159944267	0,338502833	-3,426689977	0,000610986	0,013381429	2,234487953
ARL6IP6	815,8585411	822,1966464	761,8498003	532,5767277	595,2919171	571,2900615	692,5284246	-0,499883452	0,145906725	-3,426048063	0,000612432	0,013390321	1,41409932
DNAJC12	49,10584512	40,34717235	61,30981242	11,69821714	21,54902867	20,56286606	20,81844574	-1,495902289	0,436781348	-3,424830971	0,000615182	0,013409203	2,820404893
TBC1D14	106,6298351	100,3758922	105,5332837	60,33817262	51,1789431	75,09916301	95,12577855	-0,743877552	0,217231572	-3,42435285	0,000616265	0,013409203	1,674670832
ARL9	42,09072439	61,99687459	37,18791901	16,00808661	18,85540009	16,98671544	16,19574184	-1,461552773	0,427124086	-3,421845831	0,000621976	0,013431377	2,754062222
C7orf29	14,03024146	10,82485112	15,07618338	1,847086917	6,285133363	1,78807531	33,31751385	-2,049700509	0,598976839	-3,422002947	0,000621616	0,013431377	4,140200138
MX1	863,5613621	1017,043967	634,204781	392,198122	470,487126	559,667572	339,6340189	-0,824966691	0,241010106	-3,422954764	0,000619444	0,013431377	1,771494138
METTL14	147,3175354	157,9444186	95,48249476	57,25969442	79,01310513	78,67531363	95,62564864	-0,914818107	0,267915414	-3,414578109	0,000638809	0,013597651	1,885331357
PPP1R10	1373,560639	1962,250285	1351,83111	938,3201538	1087,328072	978,0771944	1027,030355	-0,643585739	0,188550665	-3,413330515	0,000641741	0,013637517	1,562207118
HIST1H1A	794,1116668	816,2921821	798,0326404	418,6730345	566,5598788	539,1047059	1201,924371	-0,661753194	0,19395879	-3,411823682	0,000645298	0,013690531	1,582003941
RRAD	7,716632805	6,396502934	9,04571003	1,847086917	0	0	10,14412822	-3,320739768	0,973799218	-3,410086706	0,000649422	0,013732776	9,991766536
FAM221A	54,71794171	45,26755923	48,24378683	23,39643428	30,52779062	20,56286606	44,36144762	-0,9952891	0,292156349	-3,40670022	0,000657533	0,013846529	1,993479957
C4orf52	1181,346331	1074,120454	1124,68328	601,5346393	833,2291087	736,6870276	871,9073061	-0,639509246	0,187945299	-3,402634964	0,000667394	0,013965924	1,557799161
UBQLN2	1415,651364	1757,070152	1189,008329	726,520854	948,1572616	871,6867135	1143,64003	-0,777943358	0,228606631	-3,402978104	0,000666556	0,013965924	1,714684751
KCTD18	263,7685395	222,4014866	199,0056207	136,0687362	106,8472672	138,5758365	190,4356499	-0,844737641	0,248597639	-3,39801152	0,000678776	0,014167378	1,795938118
LEPREL4	601,8973588	680,4895044	605,0574931	394,6609046	460,6104879	354,932949	415,6195059	-0,642353605	0,189079918	-3,397259802	0,000680643	0,014171801	1,560873484
KIF13A	308,6653122	242,5750728	315,5947721	163,1593443	180,4731151	171,6552297	198,0958478	-0,74845209	0,220416449	-3,395627196	0,000684716	0,014222204	1,679989353
PIAS3	411,787587	363,6165899	345,7471389	230,270169	284,6267537	238,7080539	494,2536737	-0,576269976	0,170309313	-3,383666844	0,000715247	0,01478484	1,490989374
ZSCAN21	58,22550207	79,21822864	51,2590235	30,78478195	36,81292398	25,92709199	40,95889711	-1,027581124	0,304154722	-3,378481577	0,000728873	0,015018286	2,038603387
PRR3	244,8277135	152,0399544	212,0716463	109,5938237	139,1708102	109,9666316	212,652459	-0,761373038	0,225583434	-3,375128334	0,000737813	0,015168261	1,695103117
CCDC23	362,6817418	601,7633145	377,9096635	243,815473	274,7501156	243,1782421	273,8881535	-0,820928167	0,244167938	-3,36214564	0,000773393	0,015684658	1,766542144
KIAA2013	1302,006408	2018,834734	1345,800637	897,6842416	891,5910613	860,9582617	905,2523417	-0,816961997	0,243212713	-3,35904314	0,000782129	0,015836874	1,761692341
VEGFA	1579,103677	2149,717025	1476,460893	1075,004586	1240,864901	985,2294957	1153,31473	-0,658095059	0,196244042	-3,353452434	0,000798101	0,016109637	1,577997652
UBA7	423,7132922	464,9765594	411,0772669	221,65043	285,5246299	245,8603551	289,2521098	-0,789903259	0,235841026	-3,349303857	0,000810149	0,016225658	1,728958523
HSPB1	2759,748496	3463,460319	2888,596736	2375,353775	2173,758267	2227,047798	3428,342166	-0,427606998	0,127692615	-3,34872145	0,000811854	0,016234553	1,34500077
FPR1	51,21038134	46,74367529	34,17268233	21,54934736	14,36601911	24,13901668	48,80486119	-1,14320502	0,341545405	-3,347153863	0,000816459	0,016276094	2,20871155
FTX	122,0631007	174,673734	142,7212027	88,66017201	92,48124805	91,1918408	142,4317756	-0,697008942	0,208344229	-3,345467955	0,000821439	0,016328192	1,621140287
CASP9	519,1189341	635,7139839	497,5140516	313,3890802	326,8269349	323,6416311	326,8926891	-0,779283723	0,233007083	-3,344463659	0,000824418	0,016358796	1,716278554
VEGFC	877,5916035	1021,964353	824,1646916	662,4885075	624,9218315	617,7800195	575,4895327	-0,516804467	0,154689298	-3,340919339	0,000835015	0,016518157	1,430782588
GTF2IRD1	400,5633938	482,6899522	381,929979	269,6746899	290,0140109	261,9530329	394,9888116	-0,625783179	0,187625718	-3,335273997	0,000852154	0,016805571	1,543048255
ARL6IP5	536,656736	777,9131645	515,6054717	347,868036	395,0655257	320,9595181	957,7365284	-0,784906845	0,235798459	-3,32871914	0,000872463	0,017023611	1,722981063
DGAT2	313,5758967	424,1373484	252,2748019	189,6342568	198,430639	170,7611921	166,4541059	-0,829095371	0,249146757	-3,32773896	0,000875539	0,017057771	1,77657103
C10orf118	396,3543213	543,2107107	286,4474843	206,2580391	248,7117059	223,5094137	299,1952186	-0,858656002	0,258143371	-3,326275619	0,000880149	0,017095856	1,813348229
KLF11	215,3642065	213,5447903	214,081804	128,6803885	154,4347055	115,3308575	363,6388668	-0,691435016	0,207854247	-3,326537829	0,000879321	0,017095856	1,614889013
FAM117A	264,4700516	248,4795371	262,3255909	159,4651705	184,9624961	160,0327402	161,5952757	-0,620632587	0,186646654	-3,325173925	0,000883634	0,017114608	1,537549212
FXR2	2157,149625	2689,975503	1847,335004	1139,652628	1352,201549	1462,645603	1585,514329	-0,760384669	0,22867815	-3,325130404	0,000883772	0,017114608	1,693942224
NEK10	16,13477768	21,15766355	12,06094671	4,925565112	3,591504779	1,78807531	7,974497362	-2,23564302	0,673449352	-3,319689912	0,000901175	0,017399295	4,70972564

C22orf13	965,2806127	977,6808715	836,2256383	583,6794657	689,5689175	580,230438	967,0251939	-0,585928883	0,176594742	-3,317929385	0,000906874	0,01743366	1,501005104
SHPRH	182,393139	241,0989567	182,9243584	116,9821714	128,3962958	128,7414223	166,2073451	-0,704153791	0,212215235	-3,318111402	0,000906283	0,01743366	1,629188788
USP31	425,8178284	571,7489546	380,9249001	278,2944288	273,8522394	316,4893298	425,8489028	-0,670587021	0,202167389	-3,316989069	0,000909932	0,017463638	1,591720494
BTBD3	618,7336485	900,9228363	607,0676509	461,7717292	485,7510213	449,7009404	458,4855798	-0,609012214	0,183817047	-3,313143279	0,000922537	0,017652945	1,525214565
FIBIN	54,71794171	45,26755923	49,24886572	23,39643428	20,65115248	16,98671544	17,96572248	-1,279692121	0,386305667	-3,312641339	0,000924194	0,017658416	2,427871593
GZF1	286,9184379	446,771128	275,3916165	205,0266478	198,430639	147,5162131	219,8916174	-0,874930187	0,264300748	-3,310358352	0,000931766	0,017776715	1,83391934
PRKRA	434,9374854	615,5403977	412,0823458	267,827603	302,5842776	244,9663174	536,9741828	-0,845285832	0,255856652	-3,303747729	0,000954016	0,018120683	1,796620664
AFAP1	755,5285028	791,6902478	649,2809644	481,4739897	562,0704979	546,2570071	435,9527459	-0,47027351	0,142473028	-3,300789738	0,000964131	0,018228859	1,385372085
CACNB3	596,2852622	751,3430754	575,9102052	309,0792108	365,4356112	364,7673632	560,2391052	-0,889940571	0,269615646	-3,300774957	0,000964182	0,018228859	1,853099787
PTPN23	663,6304212	790,2141317	561,8391007	424,2142953	450,7338497	413,0453966	643,8029555	-0,648524102	0,196957424	-3,292712146	0,00099226	0,018681762	1,567563735
GJC1	206,2445495	176,14985	186,9446739	94,20143276	130,1920482	124,271234	301,9683853	-0,71295983	0,216678938	-3,290397471	0,00100046	0,018781222	1,639163574
ZNF786	43,49374854	63,96502934	33,16760344	27,09060811	9,876638142	21,45690372	47,12476496	-1,261621391	0,383742396	-3,287677893	0,001010173	0,01890845	2,397650525
SEC22B	1335,678987	1462,338978	1152,825489	848,4285905	990,3574427	985,2294957	1130,13509	-0,486718311	0,148193666	-3,284339499	0,001022217	0,019078422	1,401253825
B3GNT2	101,7192506	109,2325886	102,518047	68,34221593	56,56620027	75,09916301	115,5842523	-0,649231959	0,197979591	-3,279287299	0,001040696	0,01933923	1,568333048
CLDN16	41,38921232	36,90290154	45,22855015	13,54530406	28,73203823	25,03305434	2564,802115	-0,90378619	0,275790182	-3,277078907	0,001048871	0,019435043	1,870969694
PDE4B	733,7816285	756,755501	568,874653	471,6228595	425,5933163	450,5949781	290,4440833	-0,613074361	0,187187975	-3,27518027	0,001055946	0,019538029	1,529515112
UBE2D4	126,2721732	242,5750728	145,7364394	92,35434585	97,86850522	92,97991611	169,3023497	-0,869813249	0,265681609	-3,273893341	0,001060766	0,019599067	1,827426333
MLLT11	138,8993905	110,7087046	160,8126228	65,26373773	96,07275283	74,20512536	662,4967733	-0,79966596	0,244437253	-3,271456909	0,001069949	0,019684003	1,74069804
NDUFB5	2260,973412	3381,289859	2160,919618	1413,021491	1746,369199	1742,479389	1829,539859	-0,671794568	0,205326521	-3,271835349	0,001068518	0,019684003	1,593053334
CNP	2188,016156	2523,666427	1998,096838	1248,630756	1485,087226	1452,811189	1566,998002	-0,681244921	0,208283179	-3,270763021	0,001072577	0,019704212	1,60352286
SRRM2	6690,320642	7977,915275	4850,510734	3682,475617	4338,537773	4128,66589	5693,142054	-0,684330285	0,20932735	-3,269187166	0,001078569	0,019786062	1,606955842
GPD2	284,8139017	432,9940448	294,4881154	165,6221269	187,6561247	217,2511501	448,6263141	-0,832228225	0,254952498	-3,264248164	0,00109755	0,02002018	1,780433097
FUNDC1	241,3201532	241,5909954	279,411932	163,1593443	183,1667437	148,4102507	160,0198865	-0,621101214	0,190315258	-3,263538724	0,001100301	0,020041983	1,538048732
GATA3	121,3615887	104,3122017	145,7364394	72,6520854	45,79168593	45,5959204	62,01065726	-1,166670069	0,357643636	-3,262102137	0,001105893	0,02011538	2,24492939
GLT8D2	24,55292256	26,57008911	22,11173563	10,46682586	10,77451434	13,41056482	51,31214368	-1,105053086	0,339011012	-3,25963773	0,001115546	0,020233805	2,151067931
RAB3GAP1	690,28788	1081,993073	666,3673055	443,9165557	531,5427073	529,2702917	685,1927209	-0,699737169	0,214641806	-3,260022743	0,001114033	0,020233805	1,624208867
TOM1L2	641,883547	577,1613801	596,0117831	437,7595993	447,142345	465,7936182	454,182599	-0,42660036	0,131255371	-3,250155452	0,001153419	0,020774455	1,344062624
ABAT	160,6462648	197,3075136	119,6043882	67,11082465	80,80885752	105,4964433	72,17388386	-0,926271477	0,285497258	-3,244414611	0,001176923	0,021138653	1,900358329
LOC100289341	75,06179183	58,55260378	64,3250491	30,78478195	32,32354301	37,54958151	44,72917072	-0,978109885	0,30182432	-3,240659615	0,001192535	0,021389226	1,96988292
RPSAP52	63,13608659	59,53668116	55,27933907	32,01617323	25,14053345	33,07939323	41,82102829	-0,978271682	0,3021952	-3,237217808	0,001207012	0,02158876	1,970103852
FAM115A	173,2734821	150,5638383	200,0106995	121,2920409	103,2557624	114,4368198	193,2697401	-0,616544149	0,1906533	-3,233849872	0,001221336	0,021765701	1,533198136
DCLK1	0,701512073	4,428348185	3,015236677	0	0	0	10,06079864	-4,280634657	1,324875335	-3,230971657	0,001233702	0,021944214	19,43566623
ARHGAP35	1562,968899	2050,32521	1544,806257	1159,354888	1194,175339	1282,049997	1403,842386	-0,506055091	0,156717512	-3,2290909	0,001241844	0,022058578	1,420161588
SLC25A4	852,3371689	1023,932508	811,098666	479,6269028	564,7641265	487,2505219	826,1691485	-0,81216676	0,251569733	-3,228396162	0,001244864	0,022081769	1,75584654
CLCF1	115,7494921	132,3584069	139,705966	84,35030254	87,99186708	73,3110877	118,2221646	-0,654841921	0,203194453	-3,222735219	0,001269729	0,022399405	1,574443429
C10orf88	168,3628976	209,6084808	158,802465	119,444954	115,8260291	108,1785562	120,2820088	-0,649280853	0,201552151	-3,221403749	0,001275643	0,022469697	1,5683862
HMG20B	535,2537118	715,4242512	598,0219409	413,1317738	390,5761447	442,5486392	677,0195038	-0,570490282	0,177112946	-3,221053545	0,001277203	0,022469697	1,485028153
C17orf75	294,6350707	475,8014106	357,8080856	222,2661257	255,8947155	184,1717569	234,1146195	-0,770625287	0,239402542	-3,218951977	0,0012866	0,022604146	1,706009034
MAZ	4966,705478	5947,271613	4743,972371	3032,916718	3674,109389	3179,197901	4118,839145	-0,663682724	0,206256978	-3,217746765	0,001292018	0,022668409	1,584121205
SLC35A2	528,9401032	697,7108585	464,3464482	380,4999049	367,2313636	363,8733255	342,1469616	-0,608320458	0,189157445	-3,215947744	0,001300145	0,022687355	1,524483418
SRA1	1354,619813	2133,971787	1637,273515	1113,793411	1083,736567	1045,130019	1212,743818	-0,661098462	0,205552386	-3,21620427	0,001298983	0,022687355	1,58128615
TIFA	382,3240799	519,100815	354,7928489	210,5679085	272,056487	266,4232212	465,8074706	-0,750968627	0,233490372	-3,216272345	0,001298675	0,022687355	1,682922367
TYMP	186,6022115	199,767707	153,7770705	108,3624325	98,76638142	112,6487445	71,66037895	-0,760339834	0,236398664	-3,21634573	0,001298343	0,022687355	1,693889582
PLXDC2	27,35897085	36,90290154	28,14220898	16,00808661	12,57026673	13,41056482	141,9216194	-1,138893461	0,354378281	-3,213778958	0,001310005	0,022797624	2,20212057
SYTL4	78,5693522	119,565401	84,42662694	47,4085642	59,25982885	46,48995805	72,0498624	-0,895445851	0,279964202	-3,198429816	0,001381782	0,023949642	1,860184669
CH25H	41,38921232	25,09397305	41,20823458	12,92960842	13,46814292	8,940376549	11,91912899	-1,592558566	0,498179893	-3,196754	0,001389834	0,024056831	3,015837229
IL4I1	813,7540049	1464,307133	720,6415657	474,7013376	518,9724405	463,1115052	399,2861764	-1,042782465	0,326294212	-3,195835003	0,001394268	0,024098646	2,060197239
RAB4B	185,1991873	290,7948642	182,9243584	114,5193888	149,0474483	124,271234	157,1936101	-0,773797084	0,242295083	-3,193614468	0,001405036	0,024194838	1,70976386
IFT43	544,3733688	1009,171348	670,3876211	388,5039482	413,9209257	399,6348317	508,4137495	-0,889015627	0,278637076	-3,19058626	0,001419844	0,024379676	1,851912105
UBTF	1236,765785	1578,952147	1205,089592	951,2497622	976,8892998	936,0574247	1101,083552	-0,490827425	0,153922537	-3,188795055	0,001428671	0,024433504	1,405250595

CYTIP	26,65745878	9,348735057	10,05078892	0,615695639	2,693628584	1,78807531	4,262865191	-3,235804387	1,016136672	-3,184418472	0,001450452	0,024740286	9,420504918
KIF20B	401,2649059	446,2790893	338,7115867	272,7531681	263,9756012	268,2112965	359,212693	-0,562960991	0,176816819	-3,183865618	0,001453225	0,024754797	1,477298118
PQLC2	742,1997734	897,4785655	707,5755401	402,0492523	503,7085452	517,6478022	515,1696745	-0,723782422	0,227362707	-3,183382328	0,001455653	0,024763402	1,651506242
CSRNP2	137,4963663	154,9921865	120,6094671	73,26778104	99,66425761	70,62897474	88,40122696	-0,771523861	0,242540075	-3,181016005	0,001467595	0,02493363	1,707071943
KEAP1	741,4982613	1230,096718	796,0224826	564,5929009	600,6791742	484,5684089	712,6050718	-0,747631899	0,23543962	-3,175471906	0,00149593	0,025348141	1,679034529
TNFSF9	107,3313472	102,8360856	95,48249476	63,41665081	48,48531451	69,73493708	109,030652	-0,750714058	0,236620278	-3,172653102	0,001510529	0,025494877	1,682625435
RERE	363,3832539	470,388985	337,7065078	230,270169	254,9968393	269,1053341	267,1120576	-0,640096098	0,201788846	-3,172108417	0,001513365	0,025509312	1,558432963
DNAJB2	237,1110807	210,5925581	286,4474843	140,9943013	159,8219627	151,9864013	144,4203661	-0,694124202	0,218884771	-3,171185461	0,001518182	0,025557053	1,617901976
SOCS7	311,4713605	617,0165138	307,554141	249,3567338	183,1667437	208,3107736	223,1027848	-0,949398944	0,299445261	-3,170525864	0,001521633	0,025581708	1,931067968
ZNF688	115,04798	115,6290915	106,5383626	69,5736072	63,74920982	68,84089943	88,84652925	-0,739014465	0,233606016	-3,16350785	0,001558801	0,026172419	1,669035297
POGK	739,3937251	1273,396123	762,8548792	532,5767277	609,6579362	545,3629695	918,2230955	-0,720050372	0,227706283	-3,162189302	0,001565877	0,02625699	1,647239548
DTX3	224,4838634	279,4779743	229,1579874	170,547692	148,1495721	151,0923637	184,634408	-0,642880367	0,203602973	-3,157519551	0,001591176	0,02657738	1,5614435
ULK2	48,40433305	67,40930015	35,17776123	27,70630375	24,24265726	25,03305434	62,08439043	-0,990387606	0,313931693	-3,15478694	0,001606154	0,02668909	1,986718687
DUSP18	186,6022115	171,2294632	171,8684906	126,8333016	115,8260291	118,9070081	190,9102297	-0,548388767	0,173857344	-3,154245634	0,001609136	0,026692635	1,462451486
NKAP	312,1728726	454,643747	319,6150877	195,7912132	226,2648011	277,151673	233,2588252	-0,642898752	0,203864332	-3,153561718	0,001612911	0,026698034	1,561463398
IP6K2	453,1767993	674,5850402	362,8334801	283,2199939	272,056487	303,078765	320,2129429	-0,7996418	0,253843427	-3,150137905	0,001631934	0,026909152	1,740668891
SMAD4	651,704716	591,4305021	611,0879665	380,4999049	476,7722594	464,8995805	506,4865177	-0,490025515	0,15560205	-3,149222755	0,001637054	0,026959049	1,404469715
BBS1	317,7849691	375,917557	368,8639534	241,9683861	225,3669249	239,6020915	226,4294264	-0,58734321	0,186684646	-3,146178446	0,00165419	0,027206468	1,502477316
ACP6	280,6048293	368,5369767	242,224013	161,3122574	192,1455057	159,1387026	212,1097449	-0,802875999	0,255575011	-3,141449528	0,001681138	0,027544138	1,744575457
TNFAIP6	91,19656951	67,40930015	66,33520688	26,47491248	43,09805734	43,80784509	38,29360183	-1,003466837	0,319606774	-3,139692015	0,001691255	0,027674697	2,004811836
EIF1B	1424,771021	1875,159437	1341,780321	830,573417	1037,047005	1043,341943	1317,578308	-0,674545165	0,215694485	-3,12731763	0,001764092	0,028684318	1,596093497
TUBG2	365,4877901	442,3427798	359,8182434	281,9886027	272,056487	275,3635977	216,8311589	-0,496533739	0,158754269	-3,127687475	0,001761874	0,028684318	1,41081981
GUCY1B3	147,3175354	150,5638383	123,6247037	88,66017201	51,1789431	98,34414204	117,8163018	-0,823148877	0,263260515	-3,126746433	0,001767523	0,028698354	1,769263438
FKRP	312,8743846	210,1005194	315,5947721	188,4028655	190,3497533	167,1850415	186,2471336	-0,612356026	0,195994391	-3,124354845	0,001781954	0,028796684	1,528753738
KRT222	21,74687427	21,15766355	20,10157784	11,69821714	10,77451434	2,682112965	29,35400016	-1,285536568	0,411549147	-3,123652615	0,001786212	0,028796684	2,437726999
SDE2	854,4417051	1250,762343	882,4592673	608,9229869	736,2584796	600,7933041	691,2012422	-0,620493783	0,19861779	-3,124059444	0,001783744	0,028796684	1,537401288
TMEM150A	112,2419317	102,3440469	119,6043882	80,65612871	55,66832407	62,58263584	73,60821921	-0,733380673	0,234835777	-3,1229512	0,001790474	0,028796684	1,662530345
COQ9	326,203114	363,1245512	374,8944268	230,8858646	235,243563	296,8205014	317,5719935	-0,481972926	0,154799297	-3,11353434	0,00184861	0,029609949	1,396652322
ACTR10	867,7704345	972,268446	874,4186362	524,5726844	721,8924605	680,3626554	784,734497	-0,497283122	0,159816001	-3,111597829	0,001860778	0,02972275	1,411552827
ZNF836	138,1978784	142,6912193	144,7313605	78,80904179	80,80885752	94,76799142	91,24589	-0,744762757	0,239567837	-3,108776064	0,00187864	0,029859857	1,675698687
MTR	360,5772056	518,1167377	397,0061624	274,600255	255,8947155	293,2443508	394,387148	-0,6335643	0,204064696	-3,104722737	0,001904575	0,030073035	1,55139311
PKP4	249,738298	356,2360096	242,224013	144,6884752	210,1030296	163,6088908	219,5526729	-0,718058063	0,231433704	-3,102651213	0,001917955	0,03023186	1,64496634
CDIPT	1359,530398	1547,95371	1294,541613	861,3581989	950,8508902	865,4284499	977,3406608	-0,650782604	0,209963011	-3,099510726	0,001938406	0,030516855	1,570019638
TM7SF2	190,8112839	81,67842208	163,8278594	86,19738946	55,66832407	65,26474881	91,99560349	-1,063925185	0,343458029	-3,097686166	0,001950379	0,030630457	2,090611784
CXCR5	77,16632805	84,13861552	63,31997021	33,86326014	35,01717159	43,80784509	28,10943255	-1,006104678	0,324926416	-3,096407763	0,001958808	0,03072537	2,008480811
IFT52	737,2891889	1287,173206	784,9666148	429,1398604	568,3556312	500,6610867	539,5583715	-0,908752876	0,294557952	-3,085141211	0,002034555	0,031643683	1,877421877
BBS2	494,5660116	831,0533427	486,4581838	306,6164282	342,0908302	383,5421539	432,355575	-0,814889234	0,264460196	-3,081330368	0,002060779	0,031897484	1,759163084
SLC43A2	186,6022115	305,0639861	220,1122774	112,0566063	176,8816104	104,6024056	127,6882266	-0,860667076	0,279304292	-3,081467425	0,00205983	0,031897484	1,815877746
C2orf47	403,3694421	716,4083286	420,1229769	296,765298	324,1333063	313,8072169	405,2392926	-0,724062863	0,235041828	-3,080570249	0,002066046	0,031940626	1,651827304
MOSPD2	430,7284129	316,8729146	393,9909257	224,1132126	310,6651634	214,5690372	298,6782555	-0,607204269	0,197255703	-3,078259637	0,002082134	0,032112245	1,523304406
GBA2	127,6751973	132,3584069	96,48757365	79,42473743	75,42160035	51,85418398	128,042022	-0,787607062	0,256068876	-3,07576256	0,00209965	0,032266455	1,726208896
HYDIN	101,0177385	97,42366007	118,5993093	60,95386826	53,87257168	67,05282412	44,38518299	-0,796065695	0,258792354	-3,07607888	0,002097424	0,032266455	1,736359521
SRPR	4126,995526	1960,774169	4097,706643	1741,187267	1974,429752	1699,565582	2247,912981	-0,911135544	0,296394845	-3,074060025	0,002111669	0,032373903	1,880525076
TGFB3	25,25443463	14,26912193	21,10665674	6,15695639	10,77451434	1,78807531	14,83770714	-1,686287749	0,548522339	-3,074237144	0,002110416	0,032373903	3,218275312
GSK3A	1932,665762	2568,441947	1858,390872	1258,481886	1391,708102	1247,182529	1495,625133	-0,706986469	0,230059347	-3,073061275	0,00211875	0,032443828	1,632390782
DEM1	348,6515004	343,9350424	406,0518724	208,7208216	276,545868	213,6749995	181,0540422	-0,652464031	0,212574193	-3,069347326	0,00214527	0,032771993	1,571850525
WASL	519,8204462	466,4526755	472,3870793	315,8518628	394,1676495	365,6614008	529,099789	-0,442346743	0,144195785	-3,067681513	0,002157264	0,032877214	1,358812828
SLC16A5	58,92701415	54,1242556	53,26918129	26,47491248	38,60867637	24,13901668	48,39298114	-0,9070128	0,295778509	-3,066527054	0,002165612	0,032965429	1,87515883
NAP1L4	3586,129718	3005,372302	2909,703393	1958,527828	2686,445574	1962,412652	2922,213094	-0,524457526	0,171278224	-3,062021039	0,00219848	0,033390925	1,438392622
PRKRIP1	394,2497851	538,2903238	323,6354033	200,1010827	280,1373727	253,0126563	239,2858294	-0,781968198	0,255402041	-3,061714762	0,002200731	0,033390925	1,719475068

SPEF1	30,16501915	21,15766355	34,17268233	6,772652029	8,978761947	10,72845186	11,63351842	-1,695313686	0,553728855	-3,061631467	0,002201343	0,033390925	3,238472933
DHRS2	386,5331523	503,355577	404,0417147	271,5217768	284,6267537	289,6682002	188,0454641	-0,616059963	0,201381337	-3,059171083	0,002219504	0,033626785	1,532683662
LOC442028	101,0177385	141,7071419	98,49773143	52,94982495	67,3407146	62,58263584	43,67464894	-0,910748281	0,297903303	-3,057194309	0,002234194	0,033690808	1,880020354
SLC9A7	873,3825311	1158,751108	981,9620777	574,4440312	721,8924605	545,3629695	1113,461464	-0,711542176	0,232793145	-3,05654265	0,002239056	0,033693532	1,637553653
COQ10A	164,8553372	309,0002956	164,8329383	122,5234322	87,09399088	112,6487445	105,5552976	-0,989532834	0,324299027	-3,051297576	0,002278546	0,034159627	1,985541939
ALDH3B1	168,3628976	135,8026777	185,9395951	104,052563	108,6430196	101,9202927	128,602665	-0,632041505	0,207212126	-3,050214856	0,002286777	0,034203386	1,549756447
KIAA0825	14,73175354	21,15766355	19,09649895	6,772652029	3,591504779	5,364225929	9,850644612	-1,783091816	0,585096103	-3,047519555	0,002307385	0,034337157	3,441629548
LZTFL1	455,2813355	643,5866029	397,0061624	278,9101245	334,9078206	338,8402712	317,5368628	-0,656087931	0,215249327	-3,048037084	0,002303415	0,034337157	1,575803813
NFKB2	961,7730523	1502,194112	856,3272161	535,6552059	703,9349366	676,7865047	823,83395	-0,794902972	0,260854636	-3,047302451	0,002309052	0,034337157	1,734960686
RALGAPB	1480,891986	1667,519111	1263,384167	1003,583892	1124,140996	975,3950815	850,5593236	-0,509001102	0,167099704	-3,046092191	0,002318367	0,034387123	1,423064548
ARPC3	4239,93897	6829,496979	3773,066161	2903,620633	2763,662927	3391,978863	4313,902634	-0,712684237	0,234259245	-3,042288632	0,002347866	0,03471392	1,638850481
FAM127C	208,3490857	229,2900283	192,9751473	147,7669534	133,783553	137,6817989	159,5262007	-0,590828472	0,19427825	-3,041145731	0,002356797	0,034765543	1,506111388
OSBPL11	517,0143979	430,0418126	462,3362904	304,1536457	397,7591542	303,9728027	424,4542313	-0,487878786	0,160443896	-3,04080616	0,002359457	0,034765543	1,402381419
ODF2L	89,79354537	117,1052076	105,5332837	55,41260751	66,44283841	55,4303346	109,4904523	-0,822323669	0,270509383	-3,039908118	0,002366503	0,03482952	1,768251725
LZTS2	289,0229741	317,3649533	270,366222	222,8818213	210,1030296	207,4167359	297,8117165	-0,455771519	0,150000849	-3,038459597	0,00237791	0,034917587	1,371516063
FBXO17	131,8842698	250,9397305	171,8684906	87,42878073	105,949391	103,708368	72,2976605	-0,907310754	0,298667825	-3,037859042	0,002382654	0,034947398	1,875546138
MLYCD	145,9145112	110,7087046	123,6247037	81,88751998	91,58337186	65,26474881	120,6527476	-0,667793445	0,219893937	-3,03688885	0,002390336	0,035020187	1,588641334
FAM227B	56,82247793	64,94910671	55,27933907	19,70226045	32,32354301	42,01976978	51,65686845	-0,940189109	0,309962599	-3,033234049	0,002419479	0,035326583	1,918779736
MYEF2	256,0519067	263,7327364	207,0462518	194,5598219	139,1708102	150,198326	277,2924686	-0,585252531	0,19308591	-3,031047326	0,00243707	0,035543141	1,500301581
C9orf64	324,0985778	343,4430037	281,4220898	187,1714742	221,7754201	212,7809619	131,5940719	-0,614857401	0,20297243	-3,029265603	0,002451491	0,035632387	1,531406622
SAT2	246,2307377	223,385564	251,269723	150,2297359	158,0262103	180,5956063	191,9015672	-0,56050139	0,185004652	-3,029661055	0,002448283	0,035632387	1,474781669
FNTA	1275,348949	1481,036449	1282,480666	863,2052858	1123,24312	998,6400605	990,9372153	-0,438216831	0,144741486	-3,027582784	0,002465182	0,035784625	1,354928602
SNORA68	636,2714504	765,6121973	1668,430961	379,2685136	584,5174027	518,5418398	731,8226979	-1,04992512	0,346858422	-3,026955819	0,002470301	0,035784625	2,070422384
SRI	458,0873838	649,9831058	466,356606	319,5460366	343,8865826	340,6283465	845,7450836	-0,651857552	0,215345187	-3,027035624	0,002469649	0,035784625	1,57118989
MMP16	236,4095687	233,7183764	180,9142006	152,0768228	117,6217815	155,5625519	330,4896973	-0,617074724	0,204136348	-3,0228557	0,002504016	0,036232269	1,533762099
GPS1	2607,520376	3175,617687	2655,418433	2028,101435	2334,478106	2033,041627	1871,112075	-0,400755223	0,132706319	-3,01986541	0,00252887	0,036550833	1,320198829
GLS	2034,385012	3439,350424	1844,319767	1395,782014	1450,967931	1623,572381	3268,997551	-0,712008187	0,236194159	-3,014503785	0,002574	0,037057346	1,638082691
RNMTL1	235,0065445	404,4558009	259,3103542	142,2256926	199,3285152	162,7148532	208,2875755	-0,839319108	0,278645336	-3,012141241	0,002594119	0,037221001	1,789205512
ACVR2A	439,1465578	584,5419604	346,7522178	289,3769503	232,5499344	258,3768823	226,2755041	-0,814086169	0,270593387	-3,008522042	0,002625218	0,037458398	1,758184131
AGPAT1	1432,487653	1917,474764	1246,297826	895,2214591	1092,715329	1044,235981	1197,30389	-0,6018327	0,200043166	-3,008514177	0,002625286	0,037458398	1,517643251
SPATA24	38,58316402	59,04464247	25,1269723	20,93365173	13,46814292	19,66882841	28,3894258	-1,196745121	0,397850192	-3,008029518	0,002629476	0,037458398	2,292219373
TSPAN17	220,976303	307,5241795	236,1935397	150,2297359	182,2688675	172,5492674	365,7765669	-0,605798006	0,201375463	-3,008300992	0,002627128	0,037458398	1,521820294
USP4	235,0065445	224,3696414	215,0868829	156,3866923	180,4731151	136,7877612	185,2985117	-0,511658901	0,170213643	-3,005980564	0,002647259	0,037670059	1,4256886
REEP2	49,10584512	74,29784177	55,27933907	27,70630375	25,14053345	37,54958151	31,55534663	-0,995682455	0,331294312	-3,005431779	0,002652041	0,037696448	1,994023558
HSPA4	9636,671349	16390,79275	9615,589762	6385,995167	6819,369699	7511,704376	8646,567469	-0,782944596	0,260793205	-3,002166392	0,002680656	0,038019261	1,720639182
NEK3	13,32872939	30,01435992	19,09649895	11,0825215	10,77451434	4,470188274	65,5529561	-1,247968385	0,415832711	-3,001130869	0,002689789	0,038106829	2,375067284
CHMP1B	1161,002481	979,6490263	1101,566466	639,0920733	794,6204323	732,2168393	840,1400185	-0,582362609	0,194394461	-2,995777786	0,002737458	0,038527857	1,497299273
CMPK2	106,6298351	93,97938926	100,5078892	43,09869473	58,36195265	66,15878646	42,74178108	-0,853059168	0,284752549	-2,995791155	0,002737338	0,038527857	1,8063271
GTF2B	100,3162265	205,1801326	118,5993093	82,50321562	74,52372416	72,41705005	101,3398554	-0,89212778	0,297660878	-2,997128094	0,002725362	0,038527857	1,855911324
USP42	248,3352739	289,3187481	252,2748019	176,0889527	211,8987819	173,443305	188,3685978	-0,497994083	0,166205313	-2,996258512	0,002733146	0,038527857	1,412248612
FRRS1	72,95725561	140,7230645	71,36060135	49,25565112	49,38319071	42,01976978	63,86446392	-1,028417191	0,343346448	-2,995275464	0,002741971	0,038549238	2,039785136
HDLBP	10002,15914	12458,9116	9487,944742	6535,609208	7763,935455	7977,497995	8729,812902	-0,520456145	0,173857188	-2,993584277	0,002757213	0,038636992	1,434408701
PAIP2B	79,27086427	129,4061747	89,4520214	51,10273803	55,66832407	36,65554385	41,18264068	-1,058952461	0,353725013	-2,993716649	0,002756018	0,038636992	2,083418205
AMD1	1374,262151	1556,318368	1503,598023	986,3444136	1227,396758	1104,136504	1351,724297	-0,419482534	0,140168691	-2,992697809	0,002765234	0,038684541	1,337447753
BCL3	82,77842463	85,12269289	76,38599581	50,4870424	26,03840965	51,85418398	67,55563106	-0,922700305	0,308333783	-2,992537162	0,00276669	0,038684541	1,895660098
RWDD1	1299,20036	1537,128859	1163,881357	774,5451138	958,9317759	969,1368179	1359,811978	-0,567514635	0,189707742	-2,991520683	0,002775917	0,038684541	1,481968348
TMEM65	414,5936352	362,6325125	340,7217445	259,8235596	295,4012681	277,151673	379,8430613	-0,429317237	0,143519135	-2,991358866	0,002777389	0,038684541	1,346596142
RWDD3	284,8139017	332,6181526	327,6557189	206,8737347	245,1202011	206,5226983	259,0952048	-0,522878352	0,174870131	-2,990095272	0,002788904	0,03878565	1,436819019
MLLT6	647,4956435	878,7810954	601,0371775	512,2587716	506,4021738	432,714225	476,8285189	-0,553638403	0,185244706	-2,988686779	0,002801792	0,038922844	1,467782705
FOXC2	246,2307377	355,7439709	255,2900386	180,3988222	178,6773627	167,1850415	115,2938311	-0,707022282	0,236732792	-2,986583631	0,002821137	0,039149351	1,632431305

C11orf74	167,6613855	272,097394	181,9192795	142,2256926	94,27700044	104,6024056	117,5438784	-0,865191982	0,289917833	-2,984266167	0,002842594	0,039404653	1,821582052
EBF1	137,4963663	68,88541621	108,5485204	51,10273803	62,85133363	43,80784509	51,03275638	-0,993099364	0,332863585	-2,983502577	0,002849696	0,039460634	1,990456529
ZBTB6	176,0795304	264,224775	165,8380172	117,597867	121,2132863	123,3771964	139,7332258	-0,749936094	0,251593635	-2,980743507	0,002875495	0,03977511	1,681718335
ACTR8	963,8775885	1549,429826	1012,114444	658,7943337	806,2928228	798,3756258	838,8198881	-0,641283862	0,215322582	-2,978247127	0,002899021	0,040057508	1,559716542
CCDC113	56,12096585	42,80736579	45,22855015	22,165043	26,03840965	21,45690372	19,92178121	-1,051098357	0,35307698	-2,976966542	0,002911158	0,040139068	2,07210679
SLC2A1-AS1	22,44838634	28,04620517	31,15744566	12,92960842	12,57026673	11,62248951	19,60506102	-1,126257133	0,378311155	-2,977065623	0,002910217	0,040139068	2,182916783
MICALL1	299,5456552	359,6802804	337,7065078	248,1253425	228,0605534	233,3438279	633,0772475	-0,490225301	0,164735939	-2,975824857	0,002922017	0,040245704	1,404664221
CCDC9	387,2346644	468,4208302	423,1382136	176,7046484	296,2991442	232,4497903	304,4201416	-0,860961051	0,289588879	-2,973045978	0,002948603	0,040394696	1,816247801
DSEL	268,679124	316,3808759	264,3357486	177,9360397	205,6136486	170,7611921	168,5488102	-0,619131938	0,208214475	-2,973529757	0,002943959	0,040394696	1,53595073
SLC25A51	155,7356802	218,9572158	174,8837272	96,04851968	101,46001	122,4831587	195,2732796	-0,785705307	0,264299447	-2,97278453	0,002951115	0,040394696	1,723934914
TSPY26P	54,01642963	80,69434471	68,34536467	28,32199939	33,2214192	38,44361916	25,66559558	-1,032237025	0,347098365	-2,973903448	0,002940376	0,040394696	2,045193045
UBE2T	1659,076053	2998,48376	1670,441119	1202,453583	1285,758711	1326,75188	1639,992469	-0,731007005	0,2459044	-2,972728447	0,002951655	0,040394696	1,65979723
DEPDC7	128,3767094	248,9715757	151,7669127	123,7548234	64,64708602	78,67531363	138,8445094	-0,983603122	0,330979698	-2,971792917	0,002960663	0,040461099	1,977397786
ZNF627	32,26955537	26,07805042	23,11681452	14,77669534	13,46814292	9,834414204	31,73831753	-1,088284441	0,366272175	-2,971245197	0,002965949	0,040461322	2,126210511
ACOX1	666,4364695	685,90193	628,1743076	498,7134676	499,2191642	533,74048	595,8300008	-0,372776135	0,125521606	-2,969816482	0,002979777	0,040594345	1,29484206
NTPCR	494,5660116	509,75208	566,8644952	363,260427	407,6357924	337,9462335	413,8688372	-0,501747366	0,168962006	-2,969586938	0,002982004	0,040594345	1,415927469
COG6	296,0380949	259,7964269	266,3459064	179,1674309	211,8987819	194,0061711	229,7309869	-0,493349164	0,166230958	-2,967853705	0,00299887	0,040737818	1,407709031
ZRANB1	566,8217551	941,7620474	537,7172073	414,9788607	405,84004	433,6082626	451,7982372	-0,708537919	0,238843039	-2,966542052	0,003011692	0,040868879	1,634147172
CHD8	886,7112605	1205,986822	814,1139027	647,7118122	606,9643076	687,5149566	680,0739965	-0,58353151	0,196736372	-2,966058107	0,003016435	0,040890157	1,498512907
RPS6KA3	1466,160233	1820,051104	1375,953003	1075,004586	1217,52012	1039,765793	1005,187511	-0,485825298	0,164177824	-2,959140796	0,003084981	0,04168771	1,400386732
SLC2A12	70,15120732	44,28348185	53,26918129	25,85921684	29,62991442	27,7151673	25,64227313	-1,011208007	0,342084184	-2,956020923	0,003116359	0,04202138	2,015598107
TUBG1	4421,630597	9612,959832	4674,621928	3401,718405	3655,253989	3110,357001	4025,205403	-0,880035777	0,297691485	-2,956200706	0,003114543	0,04202138	1,840420941
CASP10	119,2570524	120,0574397	109,5535992	74,49917232	82,60460991	53,64225929	84,21454885	-0,727105066	0,246101698	-2,954490246	0,003131861	0,042144305	1,655314178
ARNTL	353,5620849	252,4158466	286,4474843	197,0226045	206,5115248	191,3240581	174,1296143	-0,58405747	0,197725762	-2,953876438	0,003138096	0,042184137	1,499059316
SS18L2	105,226811	166,801115	135,6856504	76,96195487	71,83009557	85,82761487	119,0676854	-0,801178725	0,271364254	-2,952410693	0,003153033	0,042252606	1,74252424
IMPA1	271,4851723	330,1579591	296,4982732	228,4230821	218,1839153	199,370397	315,0067606	-0,475721684	0,161413767	-2,947218773	0,003206463	0,042879362	1,390613677
ZER1	489,6554271	854,6711997	439,2194759	323,2402105	318,7460491	361,1912126	334,9972482	-0,832818372	0,28279302	-2,944974993	0,003229808	0,043102034	1,781161548
AMIGO3	52,61340549	60,52075853	73,37075913	39,40452089	32,32354301	35,7615062	60,18803336	-0,778319492	0,264520811	-2,942375267	0,00325705	0,043193781	1,715131855
C6orf47	303,7547277	347,3793132	261,320512	198,2539957	232,5499344	211,8869242	283,029806	-0,513108538	0,174366948	-2,942693812	0,0032537	0,043193781	1,427121868
LOC100289361	16,13477768	14,26912193	14,07110449	4,309869473	8,080885752	3,57615062	15,95082318	-1,501590734	0,510232206	-2,94295561	0,00325095	0,043193781	2,831547504
MED13L	1606,462648	1226,160409	1317,658428	800,4043307	1046,025767	1059,434621	964,9758048	-0,515179286	0,175126143	-2,941761165	0,003263515	0,043193781	1,429171734
WDR36	1641,538251	1622,251552	1314,643191	1044,219804	1150,179405	1144,368198	1143,209276	-0,456971416	0,155291503	-2,942668509	0,003253966	0,043193781	1,372657235
LMO7	151,5266078	175,6578113	143,7262816	105,2839543	95,17487664	110,8606692	261,3256329	-0,601794247	0,20459972	-2,941324881	0,003268116	0,043210261	1,517602801
LYPD6B	75,7633039	121,5335557	78,39615359	39,40452089	40,40442876	55,4303346	35,34175379	-1,038458243	0,353314481	-2,939189582	0,003290717	0,043464468	2,054031409
SEC11A	2281,317262	2674,722304	2119,711384	1386,546579	1822,688675	1751,419766	2038,933249	-0,513496227	0,174905024	-2,935857497	0,003326271	0,043844131	1,427505424
RHOBTB1	24,55292256	45,75959791	35,17776123	14,77669534	19,75327628	10,72845186	32,07881947	-1,230678523	0,419260839	-2,935352908	0,003331685	0,043870596	2,346773364
CAMLG	798,3207393	1436,752967	830,1951649	545,5063361	647,3687364	512,2835762	684,1949139	-0,847390472	0,288843399	-2,933736674	0,003349082	0,044009673	1,799243532
ING5	233,6035204	253,8919626	234,1833819	135,4530406	199,3285152	166,2910038	248,1137984	-0,534404832	0,182412254	-2,929654234	0,003393394	0,044467781	1,448344532
XPNPEP3	231,4989841	217,9731384	189,9599106	142,2256926	171,4943532	149,3042884	271,0066379	-0,473831453	0,161740901	-2,929570998	0,003394303	0,044467781	1,388792877
TIPIN	554,8960499	1264,539426	673,4028578	447,6107295	500,1170404	466,6876558	548,7186495	-0,819565699	0,279833526	-2,928761644	0,003403153	0,044538416	1,764874625
TMEM127	1626,104986	1559,2706	1651,34462	1105,173672	1241,762777	1245,394453	1506,402743	-0,429391249	0,146676968	-2,927461998	0,003417408	0,044634263	1,346665226
C18orf32	175,3780183	268,6531232	170,8634117	129,2960842	125,7026673	124,271234	159,0653695	-0,703277276	0,240277557	-2,926937019	0,003423182	0,044664374	1,628199269
HDHD2	361,2787177	452,1835536	367,8588745	233,9643428	219,9796677	243,1782421	368,4690109	-0,762125216	0,260838475	-2,921828213	0,003479834	0,045265964	1,695987123
RBM15B	267,2760999	370,0130928	276,3966954	169,9319964	226,2648011	207,4167359	202,2579422	-0,605270166	0,207189906	-2,921330378	0,0034854	0,045292616	1,521263606
OTUD7B	282,7093655	405,4398783	308,5592199	193,9441263	228,9584296	230,661715	356,3257208	-0,614120194	0,210471746	-2,917827238	0,003524796	0,045666318	1,530624284
POLR2A	3088,757658	6014,188874	2997,145257	2050,882173	2372,188906	2428,206271	3024,597179	-0,821056823	0,281459863	-2,917136444	0,003532612	0,045721586	1,766699686
TMA16	1233,258225	1734,928411	1467,415183	844,118721	1139,404891	962,8785543	1057,834986	-0,591259394	0,202769447	-2,915919546	0,003546419	0,045854206	1,50656132
BTN2A2	171,1689459	207,1482873	177,8989639	123,7548234	131,0899244	109,9666316	142,0644388	-0,611119265	0,209668612	-2,914691232	0,003560406	0,045915523	1,527443764
MRPS11	989,1320232	1526,304008	895,5252929	627,3938561	802,701318	769,7664209	743,721058	-0,635233273	0,217951704	-2,914559788	0,003561906	0,045915523	1,553188869
PPM1L	196,4233805	123,5017105	153,7770705	97,27991096	97,86850522	92,08587845	82,9535777	-0,718356425	0,246497731	-2,914251676	0,003565423	0,045915523	1,645306568

ROPN1L	104,5252989	124,9778266	108,5485204	56,02830315	70,93221938	63,4766735	81,17116082	-0,83467812	0,286750408	-2,910817553	0,003604844	0,046362805	1,783459087
ZNF192	424,4148043	503,355577	389,9706102	255,5136902	320,5418015	262,8470705	366,6644643	-0,654602395	0,225280188	-2,905725532	0,003664027	0,046950586	1,57418205
PBXIP1	258,1564429	431,5179287	294,4881154	203,7952565	229,8563058	168,9731168	228,824199	-0,710825056	0,244746917	-2,904326899	0,003680437	0,047082944	1,636739876
AIMP2	1118,210245	1608,966507	1218,155617	789,9375048	944,5657568	887,7793913	1052,33376	-0,590675976	0,203565474	-2,901651076	0,003712017	0,047376968	1,505952197
OGDH	733,0801165	827,1170332	797,0275615	612,6171608	621,3303267	569,5019862	685,9234218	-0,386351087	0,133604859	-2,891744294	0,003831096	0,048655677	1,307083301
ZDHHC16	1173,629698	1591,253115	1324,69398	1022,670456	993,9489475	856,4880734	1172,967224	-0,509616003	0,176220031	-2,891930046	0,003828832	0,048655677	1,423671212
GMPPA	931,6080332	1377,708324	851,3018217	677,8808985	605,1685552	708,0778227	700,4033934	-0,667968057	0,231074724	-2,890701523	0,00384383	0,048721302	1,588833621
ABCA12	2,10453622	3,936309498	5,025394461	0	0,897876195	0	47,01699961	-3,669772737	1,270110606	-2,889333195	0,003860598	0,04888572	12,7265788
KIF13B	287,61995	309,0002956	276,3966954	198,8696914	207,409401	206,5226983	343,6664625	-0,513446754	0,177906702	-2,886045039	0,003901163	0,049254098	1,427456472
TERF2IP	766,0511839	872,8766312	669,3825422	514,1058585	558,4789931	506,9193503	600,3863764	-0,549235133	0,190271923	-2,886580032	0,003894537	0,049254098	1,463309694
GOLGA5	580,1504845	772,0087003	516,6105506	458,693251	404,9421638	420,1976978	461,9014456	-0,54385796	0,188504065	-2,885125901	0,003912572	0,049349752	1,457865838
PPP1R2	462,9979683	581,5897283	535,7070495	386,0411656	382,4952589	383,5421539	415,7114078	-0,456749159	0,158358735	-2,884268807	0,003923237	0,049435859	1,372445784
THTPA	110,8389076	143,6752967	114,5789937	88,04447637	78,11522894	68,84089943	95,4224371	-0,652129615	0,226258846	-2,882228143	0,003948737	0,049611548	1,571486213
PSMC6	2445,471087	3082,622376	2225,244667	1533,082141	1960,063733	2063,438907	2246,800014	-0,482003065	0,167299815	-2,881073504	0,003963232	0,049696691	1,3966815
DRD2	83,47993671	121,0415171	72,36568024	44,94578165	46,68956212	52,74822164	35,10589162	-0,950772579	0,330136722	-2,879935849	0,003977561	0,049823685	1,932907474
PPP2CA	2304,46716	2998,48376	2195,092301	1610,044096	1668,25397	1781,817046	1877,37084	-0,568014269	0,197289318	-2,879092873	0,003988209	0,049823685	1,482481672
ZNF430	255,3503946	333,1101913	275,3916165	163,77504	213,6945343	198,4763594	286,1092713	-0,590868861	0,205261219	-2,878619079	0,003994205	0,049842409	1,506153553
