# Supplementary material for: Analysis of the Role of FRMD5 in the Biology of Papillary Thyroid Carcinoma
Source: Int J Mol Sci. 2021 Jun 23;22(13):6726. doi: 10.3390/ijms22136726 (PMC8268710; doi:10.3390/ijms22136726)
Supplement: Supplementary file 1 [file ijms-22-06726-s001.zip › Supplementary Table S5.pdf]

Supplementary Table S5. Gene ontology (GO) enrichment analysis of the upregulated genes in TPC1 siFRMD5 (in comparison with TPC1 siNEG).

GOID	GOTerm	Ontology Source	Term PValue	Term PValue Corrected with Benjamini-Hochberg	Group PValue	Group PValue Corrected with Benjamini-Hochberg	GOLevels	GOGroups	% Associated Genes	Nr. Genes
GO:0044248	cellular catabolic process	GO_BiologicalProcess-EBI-UniProt-GOA-ACAP-ARAP_08.05.2020_00h00	3,8443E-07	0,000253914	1,7671E-13	1,2547E-11	[3]	Group73	5,66	138,00
GO:1901575	organic substance catabolic process	GO_BiologicalProcess-EBI-UniProt-GOA-ACAP-ARAP_08.05.2020_00h00	2,3971E-05	0,002261853	1,7671E-13	1,2547E-11	[3]	Group73	5,34	126,00
GO:0009894	regulation of catabolic process	GO_BiologicalProcess-EBI-UniProt-GOA-ACAP-ARAP_08.05.2020_00h00	0,0005648	0,010972164	1,7671E-13	1,2547E-11	[3, 4]	Group73	5,75	62,00
GO:0003723	RNA binding	GO_MolecularFunction-EBI-UniProt-GOA-ACAP-ARAP_08.05.2020_00h00	0,00021632	0,006969627	1,7671E-13	1,2547E-11	[4]	Group73	5,39	94,00
GO:0009057	macromolecule catabolic process	GO_BiologicalProcess-EBI-UniProt-GOA-ACAP-ARAP_08.05.2020_00h00	5,3046E-06	0,000875914	1,7671E-13	1,2547E-11	[4]	Group73	6,02	90,00
GO:0019439	aromatic compound catabolic process	GO_BiologicalProcess-EBI-UniProt-GOA-ACAP-ARAP_08.05.2020_00h00	0,00160706	0,018786948	1,7671E-13	1,2547E-11	[4]	Group73	6,15	40,00
GO:0043603	cellular amide metabolic process	GO_BiologicalProcess-EBI-UniProt-GOA-ACAP-ARAP_08.05.2020_00h00	1,4828E-05	0,001632299	1,7671E-13	1,2547E-11	[4]	Group73	6,22	73,00
GO:0044270	cellular nitrogen compound catabolic process	GO_BiologicalProcess-EBI-UniProt-GOA-ACAP-ARAP_08.05.2020_00h00	0,0018934	0,020009467	1,7671E-13	1,2547E-11	[4]	Group73	6,14	39,00
GO:0046700	heterocycle catabolic process	GO_BiologicalProcess-EBI-UniProt-GOA-ACAP-ARAP_08.05.2020_00h00	0,00169218	0,018784598	1,7671E-13	1,2547E-11	[4]	Group73	6,18	39,00
GO:1901361	organic cyclic compound catabolic process	GO_BiologicalProcess-EBI-UniProt-GOA-ACAP-ARAP_08.05.2020_00h00	0,00403709	0,02996067	1,7671E-13	1,2547E-11	[4]	Group73	5,84	40,00
GO:1901565	organonitrogen compound catabolic process	GO_BiologicalProcess-EBI-UniProt-GOA-ACAP-ARAP_08.05.2020_00h00	0,0006443	0,01165912	1,7671E-13	1,2547E-11	[4]	Group73	5,44	77,00
GO:1901566	organonitrogen compound biosynthetic process	GO_BiologicalProcess-EBI-UniProt-GOA-ACAP-ARAP_08.05.2020_00h00	1,2533E-06	0,000331134	1,7671E-13	1,2547E-11	[4]	Group73	5,85	112,00
GO:0006518	peptide metabolic process	GO_BiologicalProcess-EBI-UniProt-GOA-ACAP-ARAP_08.05.2020_00h00	0,00024483	0,007350477	1,7671E-13	1,2547E-11	[4, 5]	Group73	6,17	54,00
GO:0030163	protein catabolic process	GO_BiologicalProcess-EBI-UniProt-GOA-ACAP-ARAP_08.05.2020_00h00	0,00303904	0,025734456	1,7671E-13	1,2547E-11	[4, 5]	Group73	5,50	55,00
GO:0031329	regulation of cellular catabolic process	GO_BiologicalProcess-EBI-UniProt-GOA-ACAP-ARAP_08.05.2020_00h00	0,00029739	0,008017297	1,7671E-13	1,2547E-11	[4, 5]	Group73	6,09	55,00
GO:0034248	regulation of cellular amide metabolic process	GO_BiologicalProcess-EBI-UniProt-GOA-ACAP-ARAP_08.05.2020_00h00	0,00235406	0,022212256	1,7671E-13	1,2547E-11	[4, 5]	Group73	6,43	32,00
GO:0034655	nucleobase-containing compound catabolic process	GO_BiologicalProcess-EBI-UniProt-GOA-ACAP-ARAP_08.05.2020_00h00	0,00214339	0,021288881	1,7671E-13	1,2547E-11	[4, 5]	Group73	6,24	36,00
GO:0044265	cellular macromolecule catabolic process	GO_BiologicalProcess-EBI-UniProt-GOA-ACAP-ARAP_08.05.2020_00h00	5,3207E-06	0,000780953	1,7671E-13	1,2547E-11	[4, 5]	Group73	6,28	78,00
GO:0043604	amide biosynthetic process	GO_BiologicalProcess-EBI-UniProt-GOA-ACAP-ARAP_08.05.2020_00h00	0,0012976	0,016971523	1,7671E-13	1,2547E-11	[5]	Group73	5,83	51,00
GO:0010608	posttranscriptional regulation of gene expression	GO_BiologicalProcess-EBI-UniProt-GOA-ACAP-ARAP_08.05.2020_00h00	0,00098194	0,014740233	1,7671E-13	1,2547E-11	[5, 6]	Group73	6,19	43,00

GO:0043043	peptide biosynthetic process	GO_BiologicalProcess-EBI-UniProt-GOA-ACAP-ARAP_08.05.2020_00h00	0,00095896	0,014730037	1,7671E-13	1,2547E-11	[5, 6]	Group73	6,15	44,00
GO:0044257	cellular protein catabolic process	GO_BiologicalProcess-EBI-UniProt-GOA-ACAP-ARAP_08.05.2020_00h00	0,00194158	0,020037686	1,7671E-13	1,2547E-11	[5, 6]	Group73	5,80	48,00
GO:0006412	translation	GO_BiologicalProcess-EBI-UniProt-GOA-ACAP-ARAP_08.05.2020_00h00	0,00240929	0,022572109	1,7671E-13	1,2547E-11	[5, 6, 7]	Group73	5,98	41,00
GO:0051603	proteolysis involved in cellular protein catabolic process	GO_BiologicalProcess-EBI-UniProt-GOA-ACAP-ARAP_08.05.2020_00h00	0,00850725	0,042730317	1,7671E-13	1,2547E-11	[5, 6, 7]	Group73	5,52	42,00
GO:0006402	mRNA catabolic process	GO_BiologicalProcess-EBI-UniProt-GOA-ACAP-ARAP_08.05.2020_00h00	7,0249E-05	0,004034745	1,7671E-13	1,2547E-11	[5, 6, 7, 8]	Group73	7,99	31,00
GO:0016071	mRNA metabolic process	GO_BiologicalProcess-EBI-UniProt-GOA-ACAP-ARAP_08.05.2020_00h00	0,00452181	0,03211461	1,7671E-13	1,2547E-11	[6, 7]	Group73	5,53	49,00
GO:0016441	posttranscriptional gene silencing	GO_BiologicalProcess-EBI-UniProt-GOA-ACAP-ARAP_08.05.2020_00h00	0,00958268	0,045209702	1,7671E-13	1,2547E-11	[6, 7]	Group73	7,88	13,00
GO:0017148	negative regulation of translation	GO_BiologicalProcess-EBI-UniProt-GOA-ACAP-ARAP_08.05.2020_00h00	0,00273635	0,024423774	1,7671E-13	1,2547E-11	[5, 6, 7, 8, 9]	Group73	9,15	13,00
GO:0061013	regulation of mRNA catabolic process	GO_BiologicalProcess-EBI-UniProt-GOA-ACAP-ARAP_08.05.2020_00h00	0,0054584	0,034833573	1,7671E-13	1,2547E-11	[5, 6, 7, 8, 9]	Group73	7,69	16,00
GO:0000956	nuclear-transcribed mRNA catabolic process	GO_BiologicalProcess-EBI-UniProt-GOA-ACAP-ARAP_08.05.2020_00h00	0,00023791	0,007308858	1,7671E-13	1,2547E-11	[6, 7, 8, 9]	Group73	9,13	20,00
GO:0000288	nuclear-transcribed mRNA catabolic process, deadenylation-dependent decay	GO_BiologicalProcess-EBI-UniProt-GOA-ACAP-ARAP_08.05.2020_00h00	0,00059753	0,011117499	1,7671E-13	1,2547E-11	[7, 8, 9, 10]	Group73	12,99	10,00
GO:0043928	exonucleolytic catabolism of deadenylated mRNA	GO_BiologicalProcess-EBI-UniProt-GOA-ACAP-ARAP_08.05.2020_00h00	0,00128489	0,016973414	1,7671E-13	1,2547E-11	[8, 9, 10, 11]	Group73	18,18	6,00
GO:0019787	ubiquitin-like protein transferase activity	GO_MolecularFunction-EBI-UniProt-GOA-ACAP-ARAP_08.05.2020_00h00	0,00464497	0,032638337	1,3695E-07	4,8616E-06	[3]	Group72	6,14	32,00
GO:0044248	cellular catabolic process	GO_BiologicalProcess-EBI-UniProt-GOA-ACAP-ARAP_08.05.2020_00h00	3,8443E-07	0,000253914	1,3695E-07	4,8616E-06	[3]	Group72	5,66	138,00
GO:1901575	organic substance catabolic process	GO_BiologicalProcess-EBI-UniProt-GOA-ACAP-ARAP_08.05.2020_00h00	2,3971E-05	0,002261853	1,3695E-07	4,8616E-06	[3]	Group72	5,34	126,00
GO:0006914	autophagy	GO_BiologicalProcess-EBI-UniProt-GOA-ACAP-ARAP_08.05.2020_00h00	0,00152075	0,018098343	1,3695E-07	4,8616E-06	[3, 4]	Group72	6,42	35,00
GO:0009894	regulation of catabolic process	GO_BiologicalProcess-EBI-UniProt-GOA-ACAP-ARAP_08.05.2020_00h00	0,0005648	0,010972164	1,3695E-07	4,8616E-06	[3, 4]	Group72	5,75	62,00
GO:0009057	macromolecule catabolic process	GO_BiologicalProcess-EBI-UniProt-GOA-ACAP-ARAP_08.05.2020_00h00	5,3046E-06	0,000875914	1,3695E-07	4,8616E-06	[4]	Group72	6,02	90,00
GO:0009896	positive regulation of catabolic process	GO_BiologicalProcess-EBI-UniProt-GOA-ACAP-ARAP_08.05.2020_00h00	0,00740365	0,039436366	1,3695E-07	4,8616E-06	[3, 4, 5]	Group72	6,11	29,00
GO:1901565	organonitrogen compound catabolic process	GO_BiologicalProcess-EBI-UniProt-GOA-ACAP-ARAP_08.05.2020_00h00	0,0006443	0,01165912	1,3695E-07	4,8616E-06	[4]	Group72	5,44	77,00
GO:0016236	macroautophagy	GO_BiologicalProcess-EBI-UniProt-GOA-ACAP-ARAP_08.05.2020_00h00	0,00066954	0,011952255	1,3695E-07	4,8616E-06	[4, 5]	Group72	7,77	24,00
GO:0030163	protein catabolic process	GO_BiologicalProcess-EBI-UniProt-GOA-ACAP-ARAP_08.05.2020_00h00	0,00303904	0,025734456	1,3695E-07	4,8616E-06	[4, 5]	Group72	5,50	55,00
GO:0031329	regulation of cellular catabolic process	GO_BiologicalProcess-EBI-UniProt-GOA-ACAP-ARAP_08.05.2020_00h00	0,00029739	0,008017297	1,3695E-07	4,8616E-06	[4, 5]	Group72	6,09	55,00

GO:0044265	cellular macromolecule catabolic process	GO_BiologicalProcess-EBI-UniProt-GOA-ACAP-ARAP_08.05.2020_00h00	5,3207E-06	0,000780953	1,3695E-07	4,8616E-06	[4, 5]	Group72	6,28	78,00
GO:0010506	regulation of autophagy	GO_BiologicalProcess-EBI-UniProt-GOA-ACAP-ARAP_08.05.2020_00h00	0,01094008	0,048496123	1,3695E-07	4,8616E-06	[4, 5, 6]	Group72	6,32	23,00
GO:0031331	positive regulation of cellular catabolic process	GO_BiologicalProcess-EBI-UniProt-GOA-ACAP-ARAP_08.05.2020_00h00	0,00992724	0,046338815	1,3695E-07	4,8616E-06	[4, 5, 6]	Group72	6,22	25,00
GO:0010508	positive regulation of autophagy	GO_BiologicalProcess-EBI-UniProt-GOA-ACAP-ARAP_08.05.2020_00h00	0,00496658	0,033645403	1,3695E-07	4,8616E-06	[4, 5, 6, 7]	Group72	8,89	12,00
GO:0044257	cellular protein catabolic process	GO_BiologicalProcess-EBI-UniProt-GOA-ACAP-ARAP_08.05.2020_00h00	0,00194158	0,020037686	1,3695E-07	4,8616E-06	[5, 6]	Group72	5,80	48,00
GO:0051603	proteolysis involved in cellular protein catabolic process	GO_BiologicalProcess-EBI-UniProt-GOA-ACAP-ARAP_08.05.2020_00h00	0,00850725	0,042730317	1,3695E-07	4,8616E-06	[5, 6, 7]	Group72	5,52	42,00
GO:0061013	regulation of mRNA catabolic process	GO_BiologicalProcess-EBI-UniProt-GOA-ACAP-ARAP_08.05.2020_00h00	0,0054584	0,034833573	1,3695E-07	4,8616E-06	[5, 6, 7, 8, 9]	Group72	7,69	16,00
GO:0048519	negative regulation of biological process	GO_BiologicalProcess-EBI-UniProt-GOA-ACAP-ARAP_08.05.2020_00h00	0,00014397	0,005943411	3,1494E-07	7,4537E-06	[1, 2, 3]	Group74	4,54	261,00
GO:0019222	regulation of metabolic process	GO_BiologicalProcess-EBI-UniProt-GOA-ACAP-ARAP_08.05.2020_00h00	0,00011434	0,005208334	3,1494E-07	7,4537E-06	[2, 3]	Group74	4,41	322,00
GO:0023051	regulation of signaling	GO_BiologicalProcess-EBI-UniProt-GOA-ACAP-ARAP_08.05.2020_00h00	0,00546368	0,034699596	3,1494E-07	7,4537E-06	[2, 3]	Group74	4,48	172,00
GO:0048583	regulation of response to stimulus	GO_BiologicalProcess-EBI-UniProt-GOA-ACAP-ARAP_08.05.2020_00h00	0,00220964	0,021462729	3,1494E-07	7,4537E-06	[2, 3]	Group74	4,48	203,00
GO:0006725	cellular aromatic compound metabolic process	GO_BiologicalProcess-EBI-UniProt-GOA-ACAP-ARAP_08.05.2020_00h00	0,01084506	0,048563812	3,1494E-07	7,4537E-06	[3]	Group74	4,23	258,00
GO:0006793	phosphorus metabolic process	GO_BiologicalProcess-EBI-UniProt-GOA-ACAP-ARAP_08.05.2020_00h00	0,00139501	0,01789129	3,1494E-07	7,4537E-06	[3]	Group74	4,66	164,00
GO:0009892	negative regulation of metabolic process	GO_BiologicalProcess-EBI-UniProt-GOA-ACAP-ARAP_08.05.2020_00h00	4,5316E-05	0,003150687	3,1494E-07	7,4537E-06	[2, 3, 4]	Group74	4,97	169,00
GO:0010033	response to organic substance	GO_BiologicalProcess-EBI-UniProt-GOA-ACAP-ARAP_08.05.2020_00h00	7,0786E-05	0,003896196	3,1494E-07	7,4537E-06	[3]	Group74	4,91	173,00
GO:0023057	negative regulation of signaling	GO_BiologicalProcess-EBI-UniProt-GOA-ACAP-ARAP_08.05.2020_00h00	0,00616829	0,036376402	3,1494E-07	7,4537E-06	[2, 3, 4]	Group74	5,01	76,00
GO:0034641	cellular nitrogen compound metabolic process	GO_BiologicalProcess-EBI-UniProt-GOA-ACAP-ARAP_08.05.2020_00h00	0,00062952	0,011549957	3,1494E-07	7,4537E-06	[3]	Group74	4,38	290,00
GO:0043170	macromolecule metabolic process	GO_BiologicalProcess-EBI-UniProt-GOA-ACAP-ARAP_08.05.2020_00h00	0,00100883	0,014807319	3,1494E-07	7,4537E-06	[3]	Group74	4,17	410,00
GO:0044093	positive regulation of molecular function	GO_BiologicalProcess-EBI-UniProt-GOA-ACAP-ARAP_08.05.2020_00h00	6,4609E-05	0,004064203	3,1494E-07	7,4537E-06	[3]	Group74	5,42	106,00
GO:0044249	cellular biosynthetic process	GO_BiologicalProcess-EBI-UniProt-GOA-ACAP-ARAP_08.05.2020_00h00	9,566E-05	0,004680266	3,1494E-07	7,4537E-06	[3]	Group74	4,51	284,00
GO:0046483	heterocycle metabolic process	GO_BiologicalProcess-EBI-UniProt-GOA-ACAP-ARAP_08.05.2020_00h00	0,00909393	0,044165753	3,1494E-07	7,4537E-06	[3]	Group74	4,25	257,00
GO:0048522	positive regulation of cellular process	GO_BiologicalProcess-EBI-UniProt-GOA-ACAP-ARAP_08.05.2020_00h00	0,01040585	0,047237542	3,1494E-07	7,4537E-06	[2, 3, 4]	Group74	4,24	253,00
GO:0048523	negative regulation of cellular process	GO_BiologicalProcess-EBI-UniProt-GOA-ACAP-ARAP_08.05.2020_00h00	0,00153646	0,018122019	3,1494E-07	7,4537E-06	[2, 3, 4]	Group74	4,45	230,00

GO:0048585	negative regulation of response to stimulus	GO_BiologicalProcess-EBI-UniProt-GOA-ACAP-ARAP_08.05.2020_00h00	0,00602008	0,035984259	3,1494E-07	7,4537E-06	[2, 3, 4]	Group74	4,91	86,00
GO:0050790	regulation of catalytic activity	GO_BiologicalProcess-EBI-UniProt-GOA-ACAP-ARAP_08.05.2020_00h00	0,00041041	0,009511445	3,1494E-07	7,4537E-06	[3]	Group74	5,00	127,00
GO:0070887	cellular response to chemical stimulus	GO_BiologicalProcess-EBI-UniProt-GOA-ACAP-ARAP_08.05.2020_00h00	0,00076148	0,012573871	3,1494E-07	7,4537E-06	[3]	Group74	4,71	167,00
GO:1901360	organic cyclic compound metabolic process	GO_BiologicalProcess-EBI-UniProt-GOA-ACAP-ARAP_08.05.2020_00h00	0,00144468	0,01783571	3,1494E-07	7,4537E-06	[3]	Group74	4,35	276,00
GO:1901564	organonitrogen compound metabolic process	GO_BiologicalProcess-EBI-UniProt-GOA-ACAP-ARAP_08.05.2020_00h00	8,9487E-10	1,18213E-06	3,1494E-07	7,4537E-06	[3]	Group74	4,86	343,00
GO:1901576	organic substance biosynthetic process	GO_BiologicalProcess-EBI-UniProt-GOA-ACAP-ARAP_08.05.2020_00h00	6,6861E-06	0,000883237	3,1494E-07	7,4537E-06	[3]	Group74	4,62	296,00
GO:0009889	regulation of biosynthetic process	GO_BiologicalProcess-EBI-UniProt-GOA-ACAP-ARAP_08.05.2020_00h00	0,0029225	0,025398796	3,1494E-07	7,4537E-06	[3, 4]	Group74	4,46	201,00
GO:0010646	regulation of cell communication	GO_BiologicalProcess-EBI-UniProt-GOA-ACAP-ARAP_08.05.2020_00h00	0,00593206	0,036111753	3,1494E-07	7,4537E-06	[3, 4]	Group74	4,48	170,00
GO:0019538	protein metabolic process	GO_BiologicalProcess-EBI-UniProt-GOA-ACAP-ARAP_08.05.2020_00h00	9,7509E-06	0,001170999	3,1494E-07	7,4537E-06	[3, 4]	Group74	4,64	282,00
GO:0031323	regulation of cellular metabolic process	GO_BiologicalProcess-EBI-UniProt-GOA-ACAP-ARAP_08.05.2020_00h00	0,00034921	0,008871378	3,1494E-07	7,4537E-06	[3, 4]	Group74	4,42	290,00
GO:0044260	cellular macromolecule metabolic process	GO_BiologicalProcess-EBI-UniProt-GOA-ACAP-ARAP_08.05.2020_00h00	0,0004098	0,009667003	3,1494E-07	7,4537E-06	[3, 4]	Group74	4,28	366,00
GO:0051171	regulation of nitrogen compound metabolic process	GO_BiologicalProcess-EBI-UniProt-GOA-ACAP-ARAP_08.05.2020_00h00	0,00046425	0,009734524	3,1494E-07	7,4537E-06	[3, 4]	Group74	4,43	273,00
GO:0060255	regulation of macromolecule metabolic process	GO_BiologicalProcess-EBI-UniProt-GOA-ACAP-ARAP_08.05.2020_00h00	0,00015088	0,006039631	3,1494E-07	7,4537E-06	[3, 4]	Group74	4,45	299,00
GO:0080090	regulation of primary metabolic process	GO_BiologicalProcess-EBI-UniProt-GOA-ACAP-ARAP_08.05.2020_00h00	0,00031975	0,008282149	3,1494E-07	7,4537E-06	[3, 4]	Group74	4,44	282,00
GO:0080134	regulation of response to stress	GO_BiologicalProcess-EBI-UniProt-GOA-ACAP-ARAP_08.05.2020_00h00	3,1639E-05	0,002458551	3,1494E-07	7,4537E-06	[3, 4]	Group74	5,76	89,00
GO:0006796	phosphate-containing compound metabolic process	GO_BiologicalProcess-EBI-UniProt-GOA-ACAP-ARAP_08.05.2020_00h00	0,00248995	0,023001596	3,1494E-07	7,4537E-06	[4]	Group74	4,61	161,00
GO:0007166	cell surface receptor signaling pathway	GO_BiologicalProcess-EBI-UniProt-GOA-ACAP-ARAP_08.05.2020_00h00	0,01122932	0,049446441	3,1494E-07	7,4537E-06	[3, 4, 5]	Group74	4,47	150,00
GO:0009059	macromolecule biosynthetic process	GO_BiologicalProcess-EBI-UniProt-GOA-ACAP-ARAP_08.05.2020_00h00	0,00712991	0,038759742	3,1494E-07	7,4537E-06	[4]	Group74	4,32	226,00
GO:0009890	negative regulation of biosynthetic process	GO_BiologicalProcess-EBI-UniProt-GOA-ACAP-ARAP_08.05.2020_00h00	0,0074441	0,039492581	3,1494E-07	7,4537E-06	[3, 4, 5]	Group74	4,88	85,00
GO:0009966	regulation of signal transduction	GO_BiologicalProcess-EBI-UniProt-GOA-ACAP-ARAP_08.05.2020_00h00	0,00407019	0,030037535	3,1494E-07	7,4537E-06	[3, 4, 5]	Group74	4,59	151,00
GO:0010467	gene expression	GO_BiologicalProcess-EBI-UniProt-GOA-ACAP-ARAP_08.05.2020_00h00	0,00333153	0,026999731	3,1494E-07	7,4537E-06	[4]	Group74	4,32	259,00
GO:0010605	negative regulation of macromolecule metabolic process	GO_BiologicalProcess-EBI-UniProt-GOA-ACAP-ARAP_08.05.2020_00h00	0,00010225	0,004824166	3,1494E-07	7,4537E-06	[3, 4, 5]	Group74	4,97	155,00
GO:0010648	negative regulation of cell communication	GO_BiologicalProcess-EBI-UniProt-GOA-ACAP-ARAP_08.05.2020_00h00	0,00588365	0,035982855	3,1494E-07	7,4537E-06	[3, 4, 5]	Group74	5,02	76,00

GO:0031324	negative regulation of cellular metabolic process	GO_BiologicalProcess-EBI-UniProt-GOA-ACAP-ARAP_08.05.2020_00h00	0,00378904	0,029100727	3,1494E-07	7,4537E-06	[3, 4, 5]	Group74	4,69	131,00
GO:0034097	response to cytokine	GO_BiologicalProcess-EBI-UniProt-GOA-ACAP-ARAP_08.05.2020_00h00	0,00028832	0,00793489	3,1494E-07	7,4537E-06	[4]	Group74	5,68	72,00
GO:0035556	intracellular signal transduction	GO_BiologicalProcess-EBI-UniProt-GOA-ACAP-ARAP_08.05.2020_00h00	0,00738036	0,039471455	3,1494E-07	7,4537E-06	[3, 4, 5]	Group74	4,57	137,00
GO:0043085	positive regulation of catalytic activity	GO_BiologicalProcess-EBI-UniProt-GOA-ACAP-ARAP_08.05.2020_00h00	0,00046043	0,009810138	3,1494E-07	7,4537E-06	[4]	Group74	5,39	85,00
GO:0043412	macromolecule modification	GO_BiologicalProcess-EBI-UniProt-GOA-ACAP-ARAP_08.05.2020_00h00	0,00070997	0,012505024	3,1494E-07	7,4537E-06	[4]	Group74	4,56	210,00
GO:0044271	cellular nitrogen compound biosynthetic process	GO_BiologicalProcess-EBI-UniProt-GOA-ACAP-ARAP_08.05.2020_00h00	0,00350894	0,027756326	3,1494E-07	7,4537E-06	[4]	Group74	4,39	223,00
GO:0051172	negative regulation of nitrogen compound metabolic process	GO_BiologicalProcess-EBI-UniProt-GOA-ACAP-ARAP_08.05.2020_00h00	0,0032815	0,027092896	3,1494E-07	7,4537E-06	[3, 4, 5]	Group74	4,75	122,00
GO:0051336	regulation of hydrolase activity	GO_BiologicalProcess-EBI-UniProt-GOA-ACAP-ARAP_08.05.2020_00h00	0,00553121	0,034960426	3,1494E-07	7,4537E-06	[4]	Group74	5,10	70,00
GO:0051338	regulation of transferase activity	GO_BiologicalProcess-EBI-UniProt-GOA-ACAP-ARAP_08.05.2020_00h00	0,00758439	0,039916255	3,1494E-07	7,4537E-06	[4]	Group74	5,22	57,00
GO:0070848	response to growth factor	GO_BiologicalProcess-EBI-UniProt-GOA-ACAP-ARAP_08.05.2020_00h00	0,00339685	0,02719537	3,1494E-07	7,4537E-06	[4]	Group74	5,76	44,00
GO:0071310	cellular response to organic substance	GO_BiologicalProcess-EBI-UniProt-GOA-ACAP-ARAP_08.05.2020_00h00	0,00166787	0,018831223	3,1494E-07	7,4537E-06	[4]	Group74	4,76	138,00
GO:1901362	organic cyclic compound biosynthetic process	GO_BiologicalProcess-EBI-UniProt-GOA-ACAP-ARAP_08.05.2020_00h00	0,00641481	0,037495423	3,1494E-07	7,4537E-06	[4]	Group74	4,39	201,00
GO:1901566	organonitrogen compound biosynthetic process	GO_BiologicalProcess-EBI-UniProt-GOA-ACAP-ARAP_08.05.2020_00h00	1,2533E-06	0,000331134	3,1494E-07	7,4537E-06	[4]	Group74	5,85	112,00
GO:0009968	negative regulation of signal transduction	GO_BiologicalProcess-EBI-UniProt-GOA-ACAP-ARAP_08.05.2020_00h00	0,00563198	0,035093613	3,1494E-07	7,4537E-06	[3, 4, 5, 6]	Group74	5,11	69,00
GO:0010468	regulation of gene expression	GO_BiologicalProcess-EBI-UniProt-GOA-ACAP-ARAP_08.05.2020_00h00	0,0051635	0,03410491	3,1494E-07	7,4537E-06	[4, 5]	Group74	4,36	221,00
GO:0034645	cellular macromolecule biosynthetic process	GO_BiologicalProcess-EBI-UniProt-GOA-ACAP-ARAP_08.05.2020_00h00	0,00412162	0,030248124	3,1494E-07	7,4537E-06	[4, 5]	Group74	4,38	222,00
GO:0044267	cellular protein metabolic process	GO_BiologicalProcess-EBI-UniProt-GOA-ACAP-ARAP_08.05.2020_00h00	3,9564E-07	0,000174212	3,1494E-07	7,4537E-06	[4, 5]	Group74	4,86	266,00
GO:0051246	regulation of protein metabolic process	GO_BiologicalProcess-EBI-UniProt-GOA-ACAP-ARAP_08.05.2020_00h00	3,0554E-06	0,000672698	3,1494E-07	7,4537E-06	[4, 5]	Group74	5,28	159,00
GO:0007167	enzyme linked receptor protein signaling pathway	GO_BiologicalProcess-EBI-UniProt-GOA-ACAP-ARAP_08.05.2020_00h00	0,00289324	0,025311073	3,1494E-07	7,4537E-06	[4, 5, 6]	Group74	5,41	60,00
GO:0010558	negative regulation of macromolecule biosynthetic process	GO_BiologicalProcess-EBI-UniProt-GOA-ACAP-ARAP_08.05.2020_00h00	0,00486912	0,033500541	3,1494E-07	7,4537E-06	[4, 5, 6]	Group74	4,99	82,00
GO:0010629	negative regulation of gene expression	GO_BiologicalProcess-EBI-UniProt-GOA-ACAP-ARAP_08.05.2020_00h00	0,00042099	0,009425861	3,1494E-07	7,4537E-06	[4, 5, 6]	Group74	5,08	115,00
GO:0016310	phosphorylation	GO_BiologicalProcess-EBI-UniProt-GOA-ACAP-ARAP_08.05.2020_00h00	0,00926835	0,044521775	3,1494E-07	7,4537E-06	[5]	Group74	4,62	119,00
GO:0019221	cytokine-mediated signaling pathway	GO_BiologicalProcess-EBI-UniProt-GOA-ACAP-ARAP_08.05.2020_00h00	0,00074874	0,012520107	3,1494E-07	7,4537E-06	[4, 5, 6]	Group74	6,01	50,00

GO:0031098	stress-activated protein kinase signaling cascade	GO_BiologicalProcess-EBI-UniProt-GOA-ACAP-ARAP_08.05.2020_00h00	0,00570056	0,035188999	3,1494E-07	7,4537E-06	[4, 5, 6]	Group74	6,79	22,00
GO:0032268	regulation of cellular protein metabolic process	GO_BiologicalProcess-EBI-UniProt-GOA-ACAP-ARAP_08.05.2020_00h00	4,4564E-06	0,000840978	3,1494E-07	7,4537E-06	[4, 5, 6]	Group74	5,31	150,00
GO:0051247	positive regulation of protein metabolic process	GO_BiologicalProcess-EBI-UniProt-GOA-ACAP-ARAP_08.05.2020_00h00	0,00532142	0,034290701	3,1494E-07	7,4537E-06	[4, 5, 6]	Group74	4,90	90,00
GO:0051248	negative regulation of protein metabolic process	GO_BiologicalProcess-EBI-UniProt-GOA-ACAP-ARAP_08.05.2020_00h00	0,00654348	0,037582349	3,1494E-07	7,4537E-06	[4, 5, 6]	Group74	5,19	61,00
GO:0051347	positive regulation of transferase activity	GO_BiologicalProcess-EBI-UniProt-GOA-ACAP-ARAP_08.05.2020_00h00	0,00168055	0,018813601	3,1494E-07	7,4537E-06	[5]	Group74	5,94	45,00
GO:0071345	cellular response to cytokine stimulus	GO_BiologicalProcess-EBI-UniProt-GOA-ACAP-ARAP_08.05.2020_00h00	0,00042874	0,009439493	3,1494E-07	7,4537E-06	[5]	Group74	5,70	67,00
GO:0071363	cellular response to growth factor stimulus	GO_BiologicalProcess-EBI-UniProt-GOA-ACAP-ARAP_08.05.2020_00h00	0,00730586	0,039553437	3,1494E-07	7,4537E-06	[5]	Group74	5,60	41,00
GO:1902531	regulation of intracellular signal transduction	GO_BiologicalProcess-EBI-UniProt-GOA-ACAP-ARAP_08.05.2020_00h00	0,00851008	0,042582653	3,1494E-07	7,4537E-06	[4, 5, 6]	Group74	4,80	92,00
GO:0023014	signal transduction by protein phosphorylation	GO_BiologicalProcess-EBI-UniProt-GOA-ACAP-ARAP_08.05.2020_00h00	0,00262072	0,023875622	3,1494E-07	7,4537E-06	[3, 4, 5, 7, 8]	Group74	5,54	55,00
GO:0006464	cellular protein modification process	GO_BiologicalProcess-EBI-UniProt-GOA-ACAP-ARAP_08.05.2020_00h00	0,00040415	0,009706852	3,1494E-07	7,4537E-06	[5, 6]	Group74	4,63	203,00
GO:0032269	negative regulation of cellular protein metabolic process	GO_BiologicalProcess-EBI-UniProt-GOA-ACAP-ARAP_08.05.2020_00h00	0,00601296	0,036105122	3,1494E-07	7,4537E-06	[4, 5, 6, 7]	Group74	5,26	58,00
GO:0032270	positive regulation of cellular protein metabolic process	GO_BiologicalProcess-EBI-UniProt-GOA-ACAP-ARAP_08.05.2020_00h00	0,00477472	0,033196837	3,1494E-07	7,4537E-06	[4, 5, 6, 7]	Group74	4,96	85,00
GO:2000112	regulation of cellular macromolecule biosynthetic process	GO_BiologicalProcess-EBI-UniProt-GOA-ACAP-ARAP_08.05.2020_00h00	0,00792705	0,041226882	3,1494E-07	7,4537E-06	[5, 6]	Group74	4,42	181,00
GO:0031399	regulation of protein modification process	GO_BiologicalProcess-EBI-UniProt-GOA-ACAP-ARAP_08.05.2020_00h00	0,00182009	0,019547418	3,1494E-07	7,4537E-06	[5, 6, 7]	Group74	5,01	99,00
GO:2000113	negative regulation of cellular macromolecule biosynthetic process	GO_BiologicalProcess-EBI-UniProt-GOA-ACAP-ARAP_08.05.2020_00h00	0,00564347	0,035000116	3,1494E-07	7,4537E-06	[5, 6, 7]	Group74	5,00	78,00
GO:0006468	protein phosphorylation	GO_BiologicalProcess-EBI-UniProt-GOA-ACAP-ARAP_08.05.2020_00h00	0,0061519	0,036606602	3,1494E-07	7,4537E-06	[6, 7]	Group74	4,79	101,00
GO:0031401	positive regulation of protein modification process	GO_BiologicalProcess-EBI-UniProt-GOA-ACAP-ARAP_08.05.2020_00h00	0,00881382	0,043606952	3,1494E-07	7,4537E-06	[5, 6, 7, 8]	Group74	5,03	67,00
GO:0044249	cellular biosynthetic process	GO_BiologicalProcess-EBI-UniProt-GOA-ACAP-ARAP_08.05.2020_00h00	9,566E-05	0,004680266	8,6913E-07	1,5427E-05	[3]	Group61	4,51	284,00
GO:1901576	organic substance biosynthetic process	GO_BiologicalProcess-EBI-UniProt-GOA-ACAP-ARAP_08.05.2020_00h00	6,6861E-06	0,000883237	8,6913E-07	1,5427E-05	[3]	Group61	4,62	296,00
GO:0043603	cellular amide metabolic process	GO_BiologicalProcess-EBI-UniProt-GOA-ACAP-ARAP_08.05.2020_00h00	1,4828E-05	0,001632299	8,6913E-07	1,5427E-05	[4]	Group61	6,22	73,00
GO:1901566	organonitrogen compound biosynthetic process	GO_BiologicalProcess-EBI-UniProt-GOA-ACAP-ARAP_08.05.2020_00h00	1,2533E-06	0,000331134	8,6913E-07	1,5427E-05	[4]	Group61	5,85	112,00
GO:0006518	peptide metabolic process	GO_BiologicalProcess-EBI-UniProt-GOA-ACAP-ARAP_08.05.2020_00h00	0,00024483	0,007350477	8,6913E-07	1,5427E-05	[4, 5]	Group61	6,17	54,00
GO:0034248	regulation of cellular amide metabolic process	GO_BiologicalProcess-EBI-UniProt-GOA-ACAP-ARAP_08.05.2020_00h00	0,00235406	0,022212256	8,6913E-07	1,5427E-05	[4, 5]	Group61	6,43	32,00

GO:0043604	amide biosynthetic process	GO_BiologicalProcess-EBI-UniProt-GOA-ACAP-ARAP_08.05.2020_00h00	0,0012976	0,016971523	8,6913E-07	1,5427E-05	[5]	Group61	5,83	51,00
GO:0043043	peptide biosynthetic process	GO_BiologicalProcess-EBI-UniProt-GOA-ACAP-ARAP_08.05.2020_00h00	0,00095896	0,014730037	8,6913E-07	1,5427E-05	[5, 6]	Group61	6,15	44,00
GO:0006412	translation	GO_BiologicalProcess-EBI-UniProt-GOA-ACAP-ARAP_08.05.2020_00h00	0,00240929	0,022572109	8,6913E-07	1,5427E-05	[5, 6, 7]	Group61	5,98	41,00
GO:0060213	positive regulation of nuclear-transcribed mRNA poly(A) tail shortening	GO_BiologicalProcess-EBI-UniProt-GOA-ACAP-ARAP_08.05.2020_00h00	0,00902835	0,044009034	1,5636E-06	2,2203E-05	[7, 8, 9, 10, 11, 12, 13]	Group69	25,00	3,00
GO:0022603	regulation of anatomical structure morphogenesis	GO_BiologicalProcess-EBI-UniProt-GOA-ACAP-ARAP_08.05.2020_00h00	0,00042874	0,009439493	1,5636E-06	2,2203E-05	[3, 4]	Group69	5,70	67,00
GO:0003725	double-stranded RNA binding	GO_MolecularFunction-EBI-UniProt-GOA-ACAP-ARAP_08.05.2020_00h00	0,00059753	0,011117499	1,5636E-06	2,2203E-05	[5]	Group69	12,99	10,00
GO:0001738	morphogenesis of a polarized epithelium	GO_BiologicalProcess-EBI-UniProt-GOA-ACAP-ARAP_08.05.2020_00h00	0,00641635	0,037339181	1,5636E-06	2,2203E-05	[5, 6]	Group69	8,28	13,00
GO:0010608	posttranscriptional regulation of gene expression	GO_BiologicalProcess-EBI-UniProt-GOA-ACAP-ARAP_08.05.2020_00h00	0,00098194	0,014740233	1,5636E-06	2,2203E-05	[5, 6]	Group69	6,19	43,00
GO:2000027	regulation of animal organ morphogenesis	GO_BiologicalProcess-EBI-UniProt-GOA-ACAP-ARAP_08.05.2020_00h00	0,01016538	0,046626646	1,5636E-06	2,2203E-05	[4, 5, 6, 7]	Group69	6,76	19,00
GO:0016441	posttranscriptional gene silencing	GO_BiologicalProcess-EBI-UniProt-GOA-ACAP-ARAP_08.05.2020_00h00	0,00958268	0,045209702	1,5636E-06	2,2203E-05	[6, 7]	Group69	7,88	13,00
GO:0035567	non-canonical Wnt signaling pathway	GO_BiologicalProcess-EBI-UniProt-GOA-ACAP-ARAP_08.05.2020_00h00	0,00230622	0,021917373	1,5636E-06	2,2203E-05	[5, 6, 7, 8]	Group69	8,97	14,00
GO:0017148	negative regulation of translation	GO_BiologicalProcess-EBI-UniProt-GOA-ACAP-ARAP_08.05.2020_00h00	0,00273635	0,024423774	1,5636E-06	2,2203E-05	[5, 6, 7, 8, 9]	Group69	9,15	13,00
GO:0007223	Wnt signaling pathway, calcium modulating pathway	GO_BiologicalProcess-EBI-UniProt-GOA-ACAP-ARAP_08.05.2020_00h00	0,00457277	0,032302819	1,5636E-06	2,2203E-05	[6, 7, 8, 9]	Group69	14,29	6,00
GO:0010586	miRNA metabolic process	GO_BiologicalProcess-EBI-UniProt-GOA-ACAP-ARAP_08.05.2020_00h00	0,00483356	0,033430025	1,5636E-06	2,2203E-05	[7, 8]	Group69	16,67	5,00
GO:0035280	miRNA loading onto RISC involved in gene silencing by miRNA	GO_BiologicalProcess-EBI-UniProt-GOA-ACAP-ARAP_08.05.2020_00h00	0,00165342	0,018829085	1,5636E-06	2,2203E-05	[5, 6, 7, 8, 9, 10, 11]	Group69	42,86	3,00
GO:0000288	nuclear-transcribed mRNA catabolic process, deadenylation-dependent decay	GO_BiologicalProcess-EBI-UniProt-GOA-ACAP-ARAP_08.05.2020_00h00	0,00059753	0,011117499	1,5636E-06	2,2203E-05	[7, 8, 9, 10]	Group69	12,99	10,00
GO:0000289	nuclear-transcribed mRNA poly(A) tail shortening	GO_BiologicalProcess-EBI-UniProt-GOA-ACAP-ARAP_08.05.2020_00h00	0,01063148	0,047769345	1,5636E-06	2,2203E-05	[7, 8, 9, 10, 11]	Group69	13,89	5,00
GO:0006810	transport	GO_BiologicalProcess-EBI-UniProt-GOA-ACAP-ARAP_08.05.2020_00h00	3,0106E-05	0,002485606	1,1929E-05	0,00010587	[3]	Group70	4,64	258,00
GO:0008104	protein localization	GO_BiologicalProcess-EBI-UniProt-GOA-ACAP-ARAP_08.05.2020_00h00	0,00059278	0,011186681	1,1929E-05	0,00010587	[3]	Group70	4,83	148,00
GO:0016192	vesicle-mediated transport	GO_BiologicalProcess-EBI-UniProt-GOA-ACAP-ARAP_08.05.2020_00h00	0,00041163	0,009375341	1,1929E-05	0,00010587	[2, 4]	Group70	5,08	116,00
GO:0051649	establishment of localization in cell	GO_BiologicalProcess-EBI-UniProt-GOA-ACAP-ARAP_08.05.2020_00h00	0,0002794	0,008023772	1,1929E-05	0,00010587	[3]	Group70	4,92	146,00
GO:0045184	establishment of protein localization	GO_BiologicalProcess-EBI-UniProt-GOA-ACAP-ARAP_08.05.2020_00h00	0,00102526	0,014883237	1,1929E-05	0,00010587	[3, 4]	Group70	4,96	116,00

GO:0046907	intracellular transport	GO_BiologicalProcess-EBI-UniProt-GOA-ACAP-ARAP_08.05.2020_00h00	0,00044702	0,009680472	1,1929E-05	0,00010587	[3, 4]	Group70	5,21	100,00
GO:0060341	regulation of cellular localization	GO_BiologicalProcess-EBI-UniProt-GOA-ACAP-ARAP_08.05.2020_00h00	0,00680137	0,038070355	1,1929E-05	0,00010587	[3, 4]	Group70	5,25	57,00
GO:0034613	cellular protein localization	GO_BiologicalProcess-EBI-UniProt-GOA-ACAP-ARAP_08.05.2020_00h00	0,00303171	0,025837982	1,1929E-05	0,00010587	[4]	Group70	4,89	103,00
GO:0071702	organic substance transport	GO_BiologicalProcess-EBI-UniProt-GOA-ACAP-ARAP_08.05.2020_00h00	7,3678E-05	0,00389315	1,1929E-05	0,00010587	[4]	Group70	5,01	154,00
GO:0071705	nitrogen compound transport	GO_BiologicalProcess-EBI-UniProt-GOA-ACAP-ARAP_08.05.2020_00h00	0,00040292	0,009856539	1,1929E-05	0,00010587	[4]	Group70	4,96	132,00
GO:1903827	regulation of cellular protein localization	GO_BiologicalProcess-EBI-UniProt-GOA-ACAP-ARAP_08.05.2020_00h00	0,00649101	0,037443761	1,1929E-05	0,00010587	[4, 5]	Group70	5,86	35,00
GO:0042886	amide transport	GO_BiologicalProcess-EBI-UniProt-GOA-ACAP-ARAP_08.05.2020_00h00	0,00150298	0,01821504	1,1929E-05	0,00010587	[5]	Group70	4,92	114,00
GO:0072657	protein localization to membrane	GO_BiologicalProcess-EBI-UniProt-GOA-ACAP-ARAP_08.05.2020_00h00	0,00434593	0,031543817	1,1929E-05	0,00010587	[5]	Group70	5,81	40,00
GO:0015031	protein transport	GO_BiologicalProcess-EBI-UniProt-GOA-ACAP-ARAP_08.05.2020_00h00	0,00092304	0,014515868	1,1929E-05	0,00010587	[4, 5, 6, 7]	Group70	5,00	112,00
GO:0006886	intracellular protein transport	GO_BiologicalProcess-EBI-UniProt-GOA-ACAP-ARAP_08.05.2020_00h00	0,00553953	0,03484628	1,1929E-05	0,00010587	[4, 5, 6, 7, 8]	Group70	5,16	66,00
GO:0080135	regulation of cellular response to stress	GO_BiologicalProcess-EBI-UniProt-GOA-ACAP-ARAP_08.05.2020_00h00	3,2327E-05	0,002372467	1,1611E-05	0,00011777	[3, 4, 5]	Group46	6,72	53,00
GO:0031098	stress-activated protein kinase signaling cascade	GO_BiologicalProcess-EBI-UniProt-GOA-ACAP-ARAP_08.05.2020_00h00	0,00570056	0,035188999	1,1611E-05	0,00011777	[4, 5, 6]	Group46	6,79	22,00
GO:0023014	signal transduction by protein phosphorylation	GO_BiologicalProcess-EBI-UniProt-GOA-ACAP-ARAP_08.05.2020_00h00	0,00262072	0,023875622	1,1611E-05	0,00011777	[3, 4, 5, 7, 8]	Group46	5,54	55,00
GO:0007275	multicellular organism development	GO_BiologicalProcess-EBI-UniProt-GOA-ACAP-ARAP_08.05.2020_00h00	0,00186308	0,019847836	1,5386E-05	0,00012138	[2, 3]	Group71	4,38	253,00
GO:0009653	anatomical structure morphogenesis	GO_BiologicalProcess-EBI-UniProt-GOA-ACAP-ARAP_08.05.2020_00h00	0,00022289	0,007010267	1,5386E-05	0,00012138	[2, 3]	Group71	4,96	143,00
GO:0050793	regulation of developmental process	GO_BiologicalProcess-EBI-UniProt-GOA-ACAP-ARAP_08.05.2020_00h00	0,00020963	0,006923161	1,5386E-05	0,00012138	[2, 3]	Group71	4,99	140,00
GO:0051239	regulation of multicellular organismal process	GO_BiologicalProcess-EBI-UniProt-GOA-ACAP-ARAP_08.05.2020_00h00	0,00397082	0,029974045	1,5386E-05	0,00012138	[2, 3]	Group71	4,57	156,00
GO:0000902	cell morphogenesis	GO_BiologicalProcess-EBI-UniProt-GOA-ACAP-ARAP_08.05.2020_00h00	0,01023283	0,046773579	1,5386E-05	0,00012138	[3, 4]	Group71	5,12	58,00
GO:0022603	regulation of anatomical structure morphogenesis	GO_BiologicalProcess-EBI-UniProt-GOA-ACAP-ARAP_08.05.2020_00h00	0,00042874	0,009439493	1,5386E-05	0,00012138	[3, 4]	Group71	5,70	67,00
GO:0035295	tube development	GO_BiologicalProcess-EBI-UniProt-GOA-ACAP-ARAP_08.05.2020_00h00	0,00116638	0,016391337	1,5386E-05	0,00012138	[3, 4]	Group71	5,57	63,00
GO:0048731	system development	GO_BiologicalProcess-EBI-UniProt-GOA-ACAP-ARAP_08.05.2020_00h00	0,00093547	0,014538361	1,5386E-05	0,00012138	[3, 4]	Group71	4,48	232,00
GO:2000026	regulation of multicellular organismal development	GO_BiologicalProcess-EBI-UniProt-GOA-ACAP-ARAP_08.05.2020_00h00	0,00279194	0,024752729	1,5386E-05	0,00012138	[3, 4]	Group71	4,87	108,00
GO:0035239	tube morphogenesis	GO_BiologicalProcess-EBI-UniProt-GOA-ACAP-ARAP_08.05.2020_00h00	0,00109299	0,015693877	1,5386E-05	0,00012138	[3, 4, 5]	Group71	5,82	53,00

GO:0048513	animal organ development	GO_BiologicalProcess-EBI-UniProt-GOA-ACAP-ARAP_08.05.2020_00h00	0,00518778	0,033926021	1,5386E-05	0,00012138	[3, 4, 5]	Group71	4,49	173,00
GO:2000145	regulation of cell motility	GO_BiologicalProcess-EBI-UniProt-GOA-ACAP-ARAP_08.05.2020_00h00	0,0097313	0,045585286	1,5386E-05	0,00012138	[3, 4, 5]	Group71	5,24	52,00
GO:0072359	circulatory system development	GO_BiologicalProcess-EBI-UniProt-GOA-ACAP-ARAP_08.05.2020_00h00	0,00358849	0,027884666	1,5386E-05	0,00012138	[4, 5]	Group71	5,32	62,00
GO:0072358	cardiovascular system development	GO_BiologicalProcess-EBI-UniProt-GOA-ACAP-ARAP_08.05.2020_00h00	0,00514764	0,03417103	1,5386E-05	0,00012138	[4, 5, 6]	Group71	5,63	44,00
GO:2000027	regulation of animal organ morphogenesis	GO_BiologicalProcess-EBI-UniProt-GOA-ACAP-ARAP_08.05.2020_00h00	0,01016538	0,046626646	1,5386E-05	0,00012138	[4, 5, 6, 7]	Group71	6,76	19,00
GO:0001568	blood vessel development	GO_BiologicalProcess-EBI-UniProt-GOA-ACAP-ARAP_08.05.2020_00h00	0,00870441	0,04322752	1,5386E-05	0,00012138	[3, 5, 6, 7, 8]	Group71	5,54	41,00
GO:0001525	angiogenesis	GO_BiologicalProcess-EBI-UniProt-GOA-ACAP-ARAP_08.05.2020_00h00	0,009983	0,046435013	1,5386E-05	0,00012138	[3, 4, 5, 6, 7, 8, 9, 10]	Group71	5,82	32,00
GO:0048514	blood vessel morphogenesis	GO_BiologicalProcess-EBI-UniProt-GOA-ACAP-ARAP_08.05.2020_00h00	0,00502904	0,033552319	1,5386E-05	0,00012138	[4, 5, 6, 7, 8, 9]	Group71	5,84	38,00
GO:0043168	anion binding	GO_MolecularFunction-EBI-UniProt-GOA-ACAP-ARAP_08.05.2020_00h00	0,00117658	0,016023351	1,0632E-05	0,00012581	[3]	Group63	4,77	144,00
GO:0044093	positive regulation of molecular function	GO_BiologicalProcess-EBI-UniProt-GOA-ACAP-ARAP_08.05.2020_00h00	6,4609E-05	0,004064203	1,0632E-05	0,00012581	[3]	Group63	5,42	106,00
GO:0050790	regulation of catalytic activity	GO_BiologicalProcess-EBI-UniProt-GOA-ACAP-ARAP_08.05.2020_00h00	0,00041041	0,009511445	1,0632E-05	0,00012581	[3]	Group63	5,00	127,00
GO:0035639	purine ribonucleoside triphosphate binding	GO_MolecularFunction-EBI-UniProt-GOA-ACAP-ARAP_08.05.2020_00h00	0,00734326	0,03943269	1,0632E-05	0,00012581	[4]	Group63	4,82	93,00
GO:0043085	positive regulation of catalytic activity	GO_BiologicalProcess-EBI-UniProt-GOA-ACAP-ARAP_08.05.2020_00h00	0,00046043	0,009810138	1,0632E-05	0,00012581	[4]	Group63	5,39	85,00
GO:0051336	regulation of hydrolase activity	GO_BiologicalProcess-EBI-UniProt-GOA-ACAP-ARAP_08.05.2020_00h00	0,00553121	0,034960426	1,0632E-05	0,00012581	[4]	Group63	5,10	70,00
GO:0016462	pyrophosphatase activity	GO_MolecularFunction-EBI-UniProt-GOA-ACAP-ARAP_08.05.2020_00h00	0,00054986	0,010841349	1,0632E-05	0,00012581	[5]	Group63	5,44	79,00
GO:0017111	nucleoside-triphosphatase activity	GO_MolecularFunction-EBI-UniProt-GOA-ACAP-ARAP_08.05.2020_00h00	0,00074854	0,012677147	1,0632E-05	0,00012581	[6]	Group63	5,43	76,00
GO:0016887	ATPase activity	GO_MolecularFunction-EBI-UniProt-GOA-ACAP-ARAP_08.05.2020_00h00	0,00383025	0,029247137	1,0632E-05	0,00012581	[7]	Group63	6,17	33,00
GO:0004386	helicase activity	GO_MolecularFunction-EBI-UniProt-GOA-ACAP-ARAP_08.05.2020_00h00	0,00353666	0,027644545	1,0632E-05	0,00012581	[8]	Group63	8,29	15,00
GO:0032879	regulation of localization	GO_BiologicalProcess-EBI-UniProt-GOA-ACAP-ARAP_08.05.2020_00h00	0,00053678	0,010909115	1,7789E-05	0,0001263	[2, 3]	Group64	4,85	145,00
GO:0016043	cellular component organization	GO_BiologicalProcess-EBI-UniProt-GOA-ACAP-ARAP_08.05.2020_00h00	2,4018E-05	0,002115194	1,7789E-05	0,0001263	[3]	Group64	4,54	302,00
GO:0044085	cellular component biogenesis	GO_BiologicalProcess-EBI-UniProt-GOA-ACAP-ARAP_08.05.2020_00h00	0,00492259	0,033519275	1,7789E-05	0,0001263	[3]	Group64	4,56	153,00
GO:0051128	regulation of cellular component organization	GO_BiologicalProcess-EBI-UniProt-GOA-ACAP-ARAP_08.05.2020_00h00	6,5914E-05	0,003957818	1,7789E-05	0,0001263	[3, 4]	Group64	5,17	132,00
GO:0006996	organelle organization	GO_BiologicalProcess-EBI-UniProt-GOA-ACAP-ARAP_08.05.2020_00h00	0,00215967	0,021290433	1,7789E-05	0,0001263	[4]	Group64	4,53	189,00
GO:0051130	positive regulation of cellular component organization	GO_BiologicalProcess-EBI-UniProt-GOA-ACAP-ARAP_08.05.2020_00h00	0,00526356	0,034084134	1,7789E-05	0,0001263	[3, 4, 5]	Group64	5,15	67,00

GO:0097435	supramolecular fiber organization	GO_BiologicalProcess-EBI-UniProt-GOA-ACAP-ARAP_08.05.2020_00h00	0,00397494	0,029834643	1,7789E-05	0,0001263	[4]	Group64	5,74	43,00
GO:0033043	regulation of organelle organization	GO_BiologicalProcess-EBI-UniProt-GOA-ACAP-ARAP_08.05.2020_00h00	0,00330584	0,026956866	1,7789E-05	0,0001263	[4, 5]	Group64	5,19	72,00
GO:0007010	cytoskeleton organization	GO_BiologicalProcess-EBI-UniProt-GOA-ACAP-ARAP_08.05.2020_00h00	0,00829588	0,042149432	1,7789E-05	0,0001263	[5]	Group64	4,98	73,00
GO:0051017	actin filament bundle assembly	GO_BiologicalProcess-EBI-UniProt-GOA-ACAP-ARAP_08.05.2020_00h00	0,00958268	0,045209702	1,7789E-05	0,0001263	[5, 6, 7, 9]	Group64	7,88	13,00
GO:0043548	phosphatidylinositol 3-kinase binding	GO_MolecularFunction-EBI-UniProt-GOA-ACAP-ARAP_08.05.2020_00h00	0,0015113	0,018149337	2,3242E-05	0,00015002	[3]	Group52	17,65	6,00
GO:0005520	insulin-like growth factor binding	GO_MolecularFunction-EBI-UniProt-GOA-ACAP-ARAP_08.05.2020_00h00	0,00642184	0,037207231	2,3242E-05	0,00015002	[4]	Group52	15,63	5,00
GO:0043559	insulin binding	GO_MolecularFunction-EBI-UniProt-GOA-ACAP-ARAP_08.05.2020_00h00	0,00097195	0,01475805	2,3242E-05	0,00015002	[3, 4, 5]	Group52	50,00	3,00
GO:0043243	positive regulation of protein-containing complex disassembly	GO_BiologicalProcess-EBI-UniProt-GOA-ACAP-ARAP_08.05.2020_00h00	0,00312759	0,026149014	2,3242E-05	0,00015002	[4, 5, 6, 7]	Group52	15,38	6,00
GO:0010828	positive regulation of glucose transmembrane transport	GO_BiologicalProcess-EBI-UniProt-GOA-ACAP-ARAP_08.05.2020_00h00	0,00885185	0,043469477	2,3242E-05	0,00015002	[4, 5, 6, 7, 8, 9, 10, 11]	Group52	12,50	6,00
GO:0014068	positive regulation of phosphatidylinositol 3-kinase signaling	GO_BiologicalProcess-EBI-UniProt-GOA-ACAP-ARAP_08.05.2020_00h00	0,0013313	0,017241579	2,3242E-05	0,00015002	[5, 6, 7, 8, 9, 10]	Group52	11,00	11,00
GO:0043903	regulation of interspecies interactions between organisms	GO_BiologicalProcess-EBI-UniProt-GOA-ACAP-ARAP_08.05.2020_00h00	0,01024824	0,046682496	5,4863E-05	0,00029964	[2, 3]	Group53	7,02	17,00
GO:0051702	interaction with symbiont	GO_BiologicalProcess-EBI-UniProt-GOA-ACAP-ARAP_08.05.2020_00h00	0,00616433	0,036516078	5,4863E-05	0,00029964	[2, 3]	Group53	9,52	10,00
GO:0016032	viral process	GO_BiologicalProcess-EBI-UniProt-GOA-ACAP-ARAP_08.05.2020_00h00	0,00016263	0,006138108	5,4863E-05	0,00029964	[3]	Group53	6,11	59,00
GO:0051817	modulation of process of other organism involved in symbiotic interaction	GO_BiologicalProcess-EBI-UniProt-GOA-ACAP-ARAP_08.05.2020_00h00	0,00797737	0,041325882	5,4863E-05	0,00029964	[3]	Group53	9,17	10,00
GO:0019058	viral life cycle	GO_BiologicalProcess-EBI-UniProt-GOA-ACAP-ARAP_08.05.2020_00h00	0,00316945	0,026332362	5,4863E-05	0,00029964	[4]	Group53	6,92	24,00
GO:1903902	positive regulation of viral life cycle	GO_BiologicalProcess-EBI-UniProt-GOA-ACAP-ARAP_08.05.2020_00h00	0,00387927	0,029451212	5,4863E-05	0,00029964	[3, 4, 5, 6]	Group53	11,76	8,00
GO:0050839	cell adhesion molecule binding	GO_MolecularFunction-EBI-UniProt-GOA-ACAP-ARAP_08.05.2020_00h00	5,1606E-05	0,003408561	5,1606E-05	0,00030533	[3]	Group37	7,30	40,00
GO:0045296	cadherin binding	GO_MolecularFunction-EBI-UniProt-GOA-ACAP-ARAP_08.05.2020_00h00	0,00038678	0,009640416	5,1606E-05	0,00030533	[4]	Group37	7,67	27,00
GO:0016765	transferase activity, transferring alkyl or aryl (other than methyl) groups	GO_MolecularFunction-EBI-UniProt-GOA-ACAP-ARAP_08.05.2020_00h00	0,00999203	0,046313923	6,8927E-05	0,00034956	[3]	Group47	10,94	7,00
GO:0004659	prenyltransferase activity	GO_MolecularFunction-EBI-UniProt-GOA-ACAP-ARAP_08.05.2020_00h00	0,00593399	0,035957828	6,8927E-05	0,00034956	[4]	Group47	20,00	4,00
GO:0045338	farnesyl diphosphate metabolic process	GO_BiologicalProcess-EBI-UniProt-GOA-ACAP-ARAP_08.05.2020_00h00	0,00049996	0,010319564	6,8927E-05	0,00034956	[5, 6, 7]	Group47	60,00	3,00
GO:0006695	cholesterol biosynthetic process	GO_BiologicalProcess-EBI-UniProt-GOA-ACAP-ARAP_08.05.2020_00h00	0,000154	0,005983353	6,8927E-05	0,00034956	[6, 7]	Group47	14,10	11,00
GO:0006790	sulfur compound metabolic process	GO_BiologicalProcess-EBI-UniProt-GOA-ACAP-ARAP_08.05.2020_00h00	0,0080568	0,041574333	0,00011946	0,00056543	[3]	Group58	6,33	25,00

GO:0046148	pigment biosynthetic process	GO_BiologicalProcess-EBI-UniProt-GOA-ACAP-ARAP_08.05.2020_00h00	0,00353662	0,027808812	0,00011946	0,00056543	[3]	Group58	11,94	8,00
GO:0051186	cofactor metabolic process	GO_BiologicalProcess-EBI-UniProt-GOA-ACAP-ARAP_08.05.2020_00h00	9,0345E-05	0,004590212	0,00011946	0,00056543	[3]	Group58	7,41	36,00
GO:0006732	coenzyme metabolic process	GO_BiologicalProcess-EBI-UniProt-GOA-ACAP-ARAP_08.05.2020_00h00	0,00677903	0,038269661	0,00011946	0,00056543	[4]	Group58	7,04	19,00
GO:0051188	cofactor biosynthetic process	GO_BiologicalProcess-EBI-UniProt-GOA-ACAP-ARAP_08.05.2020_00h00	0,00397827	0,029690914	0,00011946	0,00056543	[4]	Group58	7,56	18,00
GO:0051597	response to methylmercury	GO_BiologicalProcess-EBI-UniProt-GOA-ACAP-ARAP_08.05.2020_00h00	0,00375033	0,028971875	0,00011946	0,00056543	[4]	Group58	33,33	3,00
GO:0010288	response to lead ion	GO_BiologicalProcess-EBI-UniProt-GOA-ACAP-ARAP_08.05.2020_00h00	0,00300434	0,025939472	0,00011946	0,00056543	[5]	Group58	18,52	5,00
GO:0006783	heme biosynthetic process	GO_BiologicalProcess-EBI-UniProt-GOA-ACAP-ARAP_08.05.2020_00h00	0,00026534	0,007789075	0,00011946	0,00056543	[4, 6, 7]	Group58	24,00	6,00
GO:0032879	regulation of localization	GO_BiologicalProcess-EBI-UniProt-GOA-ACAP-ARAP_08.05.2020_00h00	0,00053678	0,010909115	0,00013732	0,0005735	[2, 3]	Group56	4,85	145,00
GO:0040012	regulation of locomotion	GO_BiologicalProcess-EBI-UniProt-GOA-ACAP-ARAP_08.05.2020_00h00	0,00599816	0,036180668	0,00013732	0,0005735	[2, 3]	Group56	5,32	55,00
GO:0051128	regulation of cellular component organization	GO_BiologicalProcess-EBI-UniProt-GOA-ACAP-ARAP_08.05.2020_00h00	6,5914E-05	0,003957818	0,00013732	0,0005735	[3, 4]	Group56	5,17	132,00
GO:0060341	regulation of cellular localization	GO_BiologicalProcess-EBI-UniProt-GOA-ACAP-ARAP_08.05.2020_00h00	0,00680137	0,038070355	0,00013732	0,0005735	[3, 4]	Group56	5,25	57,00
GO:0035239	tube morphogenesis	GO_BiologicalProcess-EBI-UniProt-GOA-ACAP-ARAP_08.05.2020_00h00	0,00109299	0,015693877	0,00013732	0,0005735	[3, 4, 5]	Group56	5,82	53,00
GO:2000145	regulation of cell motility	GO_BiologicalProcess-EBI-UniProt-GOA-ACAP-ARAP_08.05.2020_00h00	0,0097313	0,045585286	0,00013732	0,0005735	[3, 4, 5]	Group56	5,24	52,00
GO:0072359	circulatory system development	GO_BiologicalProcess-EBI-UniProt-GOA-ACAP-ARAP_08.05.2020_00h00	0,00358849	0,027884666	0,00013732	0,0005735	[4, 5]	Group56	5,32	62,00
GO:0042802	identical protein binding	GO_MolecularFunction-EBI-UniProt-GOA-ACAP-ARAP_08.05.2020_00h00	0,00013338	0,005872969	0,00013338	0,00059185	[3]	Group36	5,35	104,00
GO:0042803	protein homodimerization activity	GO_MolecularFunction-EBI-UniProt-GOA-ACAP-ARAP_08.05.2020_00h00	0,00098608	0,014636023	0,00013338	0,00059185	[4]	Group36	6,15	44,00
GO:0070291	N-acylethanolamine metabolic process	GO_BiologicalProcess-EBI-UniProt-GOA-ACAP-ARAP_08.05.2020_00h00	0,00020575	0,00715261	0,00020575	0,00081158	[5, 6, 7, 8]	Group24	75,00	3,00
GO:0002262	myeloid cell homeostasis	GO_BiologicalProcess-EBI-UniProt-GOA-ACAP-ARAP_08.05.2020_00h00	0,01053875	0,047677021	0,00022285	0,00083274	[2, 5]	Group51	7,78	13,00
GO:0048872	homeostasis of number of cells	GO_BiologicalProcess-EBI-UniProt-GOA-ACAP-ARAP_08.05.2020_00h00	0,00142667	0,017948925	0,00022285	0,00083274	[4]	Group51	7,61	22,00
GO:0030099	myeloid cell differentiation	GO_BiologicalProcess-EBI-UniProt-GOA-ACAP-ARAP_08.05.2020_00h00	0,00282726	0,0248987	0,00022285	0,00083274	[4, 5, 6, 7, 8]	Group51	6,55	29,00
GO:0030316	osteoclast differentiation	GO_BiologicalProcess-EBI-UniProt-GOA-ACAP-ARAP_08.05.2020_00h00	0,00616433	0,036516078	0,00022285	0,00083274	[6, 7, 8, 9, 10]	Group51	9,52	10,00
GO:0000278	mitotic cell cycle	GO_BiologicalProcess-EBI-UniProt-GOA-ACAP-ARAP_08.05.2020_00h00	0,00954564	0,045358974	0,00027925	0,00099132	[3]	Group67	5,16	57,00
GO:1903047	mitotic cell cycle process	GO_BiologicalProcess-EBI-UniProt-GOA-ACAP-ARAP_08.05.2020_00h00	0,00144421	0,017998116	0,00027925	0,00099132	[3, 4]	Group67	5,77	52,00
GO:0006974	cellular response to DNA damage stimulus	GO_BiologicalProcess-EBI-UniProt-GOA-ACAP-ARAP_08.05.2020_00h00	0,00117532	0,016172897	0,00027925	0,00099132	[4]	Group67	5,80	53,00

GO:0007050	cell cycle arrest	GO_BiologicalProcess-EBI-UniProt-GOA-ACAP-ARAP_08.05.2020_00h00	0,00658446	0,037654012	0,00027925	0,00099132	[3, 4, 5, 6]	Group67	7,20	18,00
GO:0044772	mitotic cell cycle phase transition	GO_BiologicalProcess-EBI-UniProt-GOA-ACAP-ARAP_08.05.2020_00h00	0,00557376	0,034895413	0,00027925	0,00099132	[4, 5]	Group67	5,92	35,00
GO:0090068	positive regulation of cell cycle process	GO_BiologicalProcess-EBI-UniProt-GOA-ACAP-ARAP_08.05.2020_00h00	0,01055324	0,047579613	0,00027925	0,00099132	[3, 4, 5, 6]	Group67	6,62	20,00
GO:0042770	signal transduction in response to DNA damage	GO_BiologicalProcess-EBI-UniProt-GOA-ACAP-ARAP_08.05.2020_00h00	0,00328226	0,026930861	0,00027925	0,00099132	[4, 5, 6]	Group67	8,97	13,00
GO:0072331	signal transduction by p53 class mediator	GO_BiologicalProcess-EBI-UniProt-GOA-ACAP-ARAP_08.05.2020_00h00	0,00770793	0,040245749	0,00027925	0,00099132	[4, 5, 6]	Group67	6,83	20,00
GO:1901990	regulation of mitotic cell cycle phase transition	GO_BiologicalProcess-EBI-UniProt-GOA-ACAP-ARAP_08.05.2020_00h00	0,00690286	0,038153462	0,00027925	0,00099132	[5, 6, 7]	Group67	6,26	27,00
GO:2000045	regulation of G1/S transition of mitotic cell cycle	GO_BiologicalProcess-EBI-UniProt-GOA-ACAP-ARAP_08.05.2020_00h00	0,00711597	0,038843803	0,00027925	0,00099132	[6, 7, 8]	Group67	8,18	13,00
GO:0031571	mitotic G1 DNA damage checkpoint	GO_BiologicalProcess-EBI-UniProt-GOA-ACAP-ARAP_08.05.2020_00h00	0,01084532	0,048400886	0,00027925	0,00099132	[5, 6, 7, 8, 9, 10, 11]	Group67	10,77	7,00
GO:0032879	regulation of localization	GO_BiologicalProcess-EBI-UniProt-GOA-ACAP-ARAP_08.05.2020_00h00	0,00053678	0,010909115	0,00036152	0,00122229	[2, 3]	Group66	4,85	145,00
GO:0060341	regulation of cellular localization	GO_BiologicalProcess-EBI-UniProt-GOA-ACAP-ARAP_08.05.2020_00h00	0,00680137	0,038070355	0,00036152	0,00122229	[3, 4]	Group66	5,25	57,00
GO:0034613	cellular protein localization	GO_BiologicalProcess-EBI-UniProt-GOA-ACAP-ARAP_08.05.2020_00h00	0,00303171	0,025837982	0,00036152	0,00122229	[4]	Group66	4,89	103,00
GO:0032386	regulation of intracellular transport	GO_BiologicalProcess-EBI-UniProt-GOA-ACAP-ARAP_08.05.2020_00h00	0,0044923	0,032077484	0,00036152	0,00122229	[4, 5]	Group66	6,47	27,00
GO:1903827	regulation of cellular protein localization	GO_BiologicalProcess-EBI-UniProt-GOA-ACAP-ARAP_08.05.2020_00h00	0,00649101	0,037443761	0,00036152	0,00122229	[4, 5]	Group66	5,86	35,00
GO:0072657	protein localization to membrane	GO_BiologicalProcess-EBI-UniProt-GOA-ACAP-ARAP_08.05.2020_00h00	0,00434593	0,031543817	0,00036152	0,00122229	[5]	Group66	5,81	40,00
GO:1904375	regulation of protein localization to cell periphery	GO_BiologicalProcess-EBI-UniProt-GOA-ACAP-ARAP_08.05.2020_00h00	0,00302544	0,025951985	0,00036152	0,00122229	[5, 6]	Group66	9,45	12,00
GO:1905475	regulation of protein localization to membrane	GO_BiologicalProcess-EBI-UniProt-GOA-ACAP-ARAP_08.05.2020_00h00	0,00090187	0,014528869	0,00036152	0,00122229	[5, 6]	Group66	8,65	18,00
GO:1905477	positive regulation of protein localization to membrane	GO_BiologicalProcess-EBI-UniProt-GOA-ACAP-ARAP_08.05.2020_00h00	0,00174604	0,018905882	0,00036152	0,00122229	[4, 5, 6, 7]	Group66	9,63	13,00
GO:0072659	protein localization to plasma membrane	GO_BiologicalProcess-EBI-UniProt-GOA-ACAP-ARAP_08.05.2020_00h00	0,00169805	0,018692721	0,00036152	0,00122229	[6]	Group66	7,51	22,00
GO:1903078	positive regulation of protein localization to plasma membrane	GO_BiologicalProcess-EBI-UniProt-GOA-ACAP-ARAP_08.05.2020_00h00	0,00194354	0,01990249	0,00036152	0,00122229	[5, 6, 7, 8]	Group66	13,11	8,00
GO:0048583	regulation of response to stimulus	GO_BiologicalProcess-EBI-UniProt-GOA-ACAP-ARAP_08.05.2020_00h00	0,00220964	0,021462729	0,00040729	0,00131445	[2, 3]	Group62	4,48	203,00
GO:0033554	cellular response to stress	GO_BiologicalProcess-EBI-UniProt-GOA-ACAP-ARAP_08.05.2020_00h00	6,7283E-07	0,000222203	0,00040729	0,00131445	[3]	Group62	5,74	126,00
GO:0048585	negative regulation of response to stimulus	GO_BiologicalProcess-EBI-UniProt-GOA-ACAP-ARAP_08.05.2020_00h00	0,00602008	0,035984259	0,00040729	0,00131445	[2, 3, 4]	Group62	4,91	86,00
GO:0080134	regulation of response to stress	GO_BiologicalProcess-EBI-UniProt-GOA-ACAP-ARAP_08.05.2020_00h00	3,1639E-05	0,002458551	0,00040729	0,00131445	[3, 4]	Group62	5,76	89,00

GO:0006974	cellular response to DNA damage stimulus	GO_BiologicalProcess-EBI-UniProt-GOA-ACAP-ARAP_08.05.2020_00h00	0,00117532	0,016172897	0,00040729	0,00131445	[4]	Group62	5,80	53,00
GO:0080135	regulation of cellular response to stress	GO_BiologicalProcess-EBI-UniProt-GOA-ACAP-ARAP_08.05.2020_00h00	3,2327E-05	0,002372467	0,00040729	0,00131445	[3, 4, 5]	Group62	6,72	53,00
GO:0031098	stress-activated protein kinase signaling cascade	GO_BiologicalProcess-EBI-UniProt-GOA-ACAP-ARAP_08.05.2020_00h00	0,00570056	0,035188999	0,00040729	0,00131445	[4, 5, 6]	Group62	6,79	22,00
GO:0072331	signal transduction by p53 class mediator	GO_BiologicalProcess-EBI-UniProt-GOA-ACAP-ARAP_08.05.2020_00h00	0,00770793	0,040245749	0,00040729	0,00131445	[4, 5, 6]	Group62	6,83	20,00
GO:2001020	regulation of response to DNA damage stimulus	GO_BiologicalProcess-EBI-UniProt-GOA-ACAP-ARAP_08.05.2020_00h00	0,00415625	0,030333709	0,00040729	0,00131445	[4, 5, 6]	Group62	7,53	18,00
GO:0006282	regulation of DNA repair	GO_BiologicalProcess-EBI-UniProt-GOA-ACAP-ARAP_08.05.2020_00h00	0,00496658	0,033645403	0,00040729	0,00131445	[5, 6, 7, 8]	Group62	8,89	12,00
GO:0019221	cytokine-mediated signaling pathway	GO_BiologicalProcess-EBI-UniProt-GOA-ACAP-ARAP_08.05.2020_00h00	0,00074874	0,012520107	0,00074874	0,00204464	[4, 5, 6]	Group43	6,01	50,00
GO:0001959	regulation of cytokine-mediated signaling pathway	GO_BiologicalProcess-EBI-UniProt-GOA-ACAP-ARAP_08.05.2020_00h00	0,00229538	0,021972449	0,00074874	0,00204464	[4, 5, 6, 7]	Group43	8,67	15,00
GO:0001961	positive regulation of cytokine-mediated signaling pathway	GO_BiologicalProcess-EBI-UniProt-GOA-ACAP-ARAP_08.05.2020_00h00	0,00435788	0,031457711	0,00074874	0,00204464	[4, 5, 6, 7, 8]	Group43	12,73	7,00
GO:0046661	male sex differentiation	GO_BiologicalProcess-EBI-UniProt-GOA-ACAP-ARAP_08.05.2020_00h00	0,00074433	0,012769634	0,00074433	0,0021139	[3, 4, 5]	Group12	9,36	16,00
GO:0055102	lipase inhibitor activity	GO_BiologicalProcess-EBI-UniProt-GOA-ACAP-ARAP_08.05.2020_00h00	0,00071719	0,012465877	0,00071719	0,00212168	[6]	Group20	25,00	5,00
GO:0090559	regulation of membrane permeability	GO_BiologicalProcess-EBI-UniProt-GOA-ACAP-ARAP_08.05.2020_00h00	0,00669038	0,037931312	0,00069836	0,00215581	[3]	Group54	10,00	9,00
GO:0006839	mitochondrial transport	GO_BiologicalProcess-EBI-UniProt-GOA-ACAP-ARAP_08.05.2020_00h00	0,00472711	0,033039756	0,00069836	0,00215581	[4]	Group54	7,14	20,00
GO:0010917	negative regulation of mitochondrial membrane potential	GO_BiologicalProcess-EBI-UniProt-GOA-ACAP-ARAP_08.05.2020_00h00	0,00077017	0,01256045	0,00069836	0,00215581	[5]	Group54	33,33	4,00
GO:0008637	apoptotic mitochondrial changes	GO_BiologicalProcess-EBI-UniProt-GOA-ACAP-ARAP_08.05.2020_00h00	0,00440738	0,031642117	0,00069836	0,00215581	[5, 6]	Group54	9,02	12,00
GO:1902108	regulation of mitochondrial membrane permeability involved in apoptotic process	GO_BiologicalProcess-EBI-UniProt-GOA-ACAP-ARAP_08.05.2020_00h00	0,00264759	0,023955263	0,00069836	0,00215581	[5, 6, 7, 8]	Group54	12,50	8,00
GO:0097345	mitochondrial outer membrane permeabilization	GO_BiologicalProcess-EBI-UniProt-GOA-ACAP-ARAP_08.05.2020_00h00	0,00532364	0,034138508	0,00069836	0,00215581	[4, 5, 6, 7, 8, 9, 10, 11]	Group54	12,28	7,00
GO:0070087	chromo shadow domain binding	GO_MolecularFunction-EBI-UniProt-GOA-ACAP-ARAP_08.05.2020_00h00	0,00097195	0,01475805	0,00097195	0,00255588	[4]	Group23	50,00	3,00
GO:1903371	regulation of endoplasmic reticulum tubular network organization	GO_BiologicalProcess-EBI-UniProt-GOA-ACAP-ARAP_08.05.2020_00h00	0,00097195	0,01475805	0,00097195	0,00255588	[5, 6, 7]	Group29	50,00	3,00
GO:0010256	endomembrane system organization	GO_BiologicalProcess-EBI-UniProt-GOA-ACAP-ARAP_08.05.2020_00h00	0,00111057	0,015774863	0,00111057	0,00281609	[4]	Group41	6,74	32,00
GO:0007030	Golgi organization	GO_BiologicalProcess-EBI-UniProt-GOA-ACAP-ARAP_08.05.2020_00h00	0,00192354	0,020166637	0,00111057	0,00281609	[5]	Group41	9,15	14,00
GO:0033197	response to vitamin E	GO_BiologicalProcess-EBI-UniProt-GOA-ACAP-ARAP_08.05.2020_00h00	0,00146665	0,017939325	0,00146665	0,00325413	[4, 5, 7]	Group04	28,57	4,00
GO:0051058	negative regulation of small GTPase mediated signal transduction	GO_BiologicalProcess-EBI-UniProt-GOA-ACAP-ARAP_08.05.2020_00h00	0,00918873	0,044300407	0,00146115	0,00334649	[5, 6, 7, 8]	Group38	11,11	7,00

GO:0046578	regulation of Ras protein signal transduction	GO_BiologicalProcess-EBI-UniProt-GOA-ACAP-ARAP_08.05.2020_00h00		0,00624691	0,036676299	0,00146115	0,00334649	[6, 7, 8]	Group38	7,58	16,00
GO:0043559	insulin binding	GO_MolecularFunction-EBI-UniProt-GOA-ACAP-ARAP_08.05.2020_00h00		0,00097195	0,01475805	0,00143933	0,00340641	[3, 4, 5]	Group45	50,00	3,00
GO:0008643	carbohydrate transport	GO_BiologicalProcess-EBI-UniProt-GOA-ACAP-ARAP_08.05.2020_00h00		0,0030825	0,025936192	0,00143933	0,00340641	[5]	Group45	8,70	14,00
GO:0010828	positive regulation of glucose transmembrane transport	GO_BiologicalProcess-EBI-UniProt-GOA-ACAP-ARAP_08.05.2020_00h00		0,00885185	0,043469477	0,00143933	0,00340641	[4, 5, 6, 7, 8, 9, 10, 11]	Group45	12,50	6,00
GO:0007569	cell aging	GO_BiologicalProcess-EBI-UniProt-GOA-ACAP-ARAP_08.05.2020_00h00		0,00140467	0,017842019	0,00140467	0,00343902	[3]	Group44	10,34	12,00
GO:0090398	cellular senescence	GO_BiologicalProcess-EBI-UniProt-GOA-ACAP-ARAP_08.05.2020_00h00		0,00119379	0,016091736	0,00140467	0,00343902	[4]	Group44	12,86	9,00
GO:2000772	regulation of cellular senescence	GO_BiologicalProcess-EBI-UniProt-GOA-ACAP-ARAP_08.05.2020_00h00		0,00020483	0,007313081	0,00140467	0,00343902	[4, 5, 6]	Group44	18,18	8,00
GO:0048545	response to steroid hormone	GO_BiologicalProcess-EBI-UniProt-GOA-ACAP-ARAP_08.05.2020_00h00		0,00164864	0,018937808	0,00164864	0,00344274	[4, 5]	Group15	6,79	29,00
GO:0043169	cation binding	GO_MolecularFunction-EBI-UniProt-GOA-ACAP-ARAP_08.05.2020_00h00		0,00162443	0,018823465	0,00162443	0,00349499	[3]	Group42	4,51	203,00
GO:0046872	metal ion binding	GO_MolecularFunction-EBI-UniProt-GOA-ACAP-ARAP_08.05.2020_00h00		0,00120791	0,016117612	0,00162443	0,00349499	[4]	Group42	4,55	200,00
GO:0046914	transition metal ion binding	GO_MolecularFunction-EBI-UniProt-GOA-ACAP-ARAP_08.05.2020_00h00		0,00058682	0,011234617	0,00162443	0,00349499	[5]	Group42	5,68	65,00
GO:0070482	response to oxygen levels	GO_BiologicalProcess-EBI-UniProt-GOA-ACAP-ARAP_08.05.2020_00h00		0,0021618	0,021153586	0,0021618	0,00414831	[3]	Group49	6,75	28,00
GO:0001666	response to hypoxia	GO_BiologicalProcess-EBI-UniProt-GOA-ACAP-ARAP_08.05.2020_00h00		0,00817135	0,042001352	0,0021618	0,00414831	[3, 5]	Group49	6,40	24,00
GO:0036293	response to decreased oxygen levels	GO_BiologicalProcess-EBI-UniProt-GOA-ACAP-ARAP_08.05.2020_00h00		0,00334785	0,026966546	0,0021618	0,00414831	[4]	Group49	6,70	26,00
GO:0071453	cellular response to oxygen levels	GO_BiologicalProcess-EBI-UniProt-GOA-ACAP-ARAP_08.05.2020_00h00		0,00912035	0,044131801	0,0021618	0,00414831	[4]	Group49	7,11	17,00
GO:0010039	response to iron ion	GO_BiologicalProcess-EBI-UniProt-GOA-ACAP-ARAP_08.05.2020_00h00		0,00205382	0,020553729	0,00205382	0,00416631	[5]	Group33	16,67	6,00
GO:0060213	positive regulation of nuclear-transcribed mRNA poly(A) tail shortening	GO_BiologicalProcess-EBI-UniProt-GOA-ACAP-ARAP_08.05.2020_00h00		0,00902835	0,044009034	0,00214339	0,00422725	[7, 8, 9, 10, 11, 12, 13]	Group59	25,00	3,00
GO:0034655	nucleobase-containing compound catabolic process	GO_BiologicalProcess-EBI-UniProt-GOA-ACAP-ARAP_08.05.2020_00h00		0,00214339	0,021288881	0,00214339	0,00422725	[4, 5]	Group59	6,24	36,00
GO:0006402	mRNA catabolic process	GO_BiologicalProcess-EBI-UniProt-GOA-ACAP-ARAP_08.05.2020_00h00		7,0249E-05	0,004034745	0,00214339	0,00422725	[5, 6, 7, 8]	Group59	7,99	31,00
GO:0061013	regulation of mRNA catabolic process	GO_BiologicalProcess-EBI-UniProt-GOA-ACAP-ARAP_08.05.2020_00h00		0,0054584	0,034833573	0,00214339	0,00422725	[5, 6, 7, 8, 9]	Group59	7,69	16,00
GO:0000956	nuclear-transcribed mRNA catabolic process	GO_BiologicalProcess-EBI-UniProt-GOA-ACAP-ARAP_08.05.2020_00h00		0,00023791	0,007308858	0,00214339	0,00422725	[6, 7, 8, 9]	Group59	9,13	20,00
GO:0110156	methylguanosine-cap decapping	GO_BiologicalProcess-EBI-UniProt-GOA-ACAP-ARAP_08.05.2020_00h00		0,00194092	0,020188617	0,00214339	0,00422725	[7, 8]	Group59	26,67	4,00

GO:0000288	nuclear-transcribed mRNA catabolic process, deadenylation-dependent decay	GO_BiologicalProcess-EBI-UniProt-GOA-ACAP-ARAP_08.05.2020_00h00	0,00059753	0,011117499	0,00214339	0,00422725	[7, 8, 9, 10]	Group59	12,99	10,00
GO:0000289	nuclear-transcribed mRNA poly(A) tail shortening	GO_BiologicalProcess-EBI-UniProt-GOA-ACAP-ARAP_08.05.2020_00h00	0,01063148	0,047769345	0,00214339	0,00422725	[7, 8, 9, 10, 11]	Group59	13,89	5,00
GO:0043928	exonucleolytic catabolism of deadenylated mRNA	GO_BiologicalProcess-EBI-UniProt-GOA-ACAP-ARAP_08.05.2020_00h00	0,00128489	0,016973414	0,00214339	0,00422725	[8, 9, 10, 11]	Group59	18,18	6,00
GO:0050680	negative regulation of epithelial cell proliferation	GO_BiologicalProcess-EBI-UniProt-GOA-ACAP-ARAP_08.05.2020_00h00	0,00226702	0,021859324	0,00226702	0,00423574	[4, 5, 6]	Group16	9,35	13,00
GO:0034248	regulation of cellular amide metabolic process	GO_BiologicalProcess-EBI-UniProt-GOA-ACAP-ARAP_08.05.2020_00h00	0,00235406	0,022212256	0,00251239	0,00445949	[4, 5]	Group60	6,43	32,00
GO:0010608	posttranscriptional regulation of gene expression	GO_BiologicalProcess-EBI-UniProt-GOA-ACAP-ARAP_08.05.2020_00h00	0,00098194	0,014740233	0,00251239	0,00445949	[5, 6]	Group60	6,19	43,00
GO:0043043	peptide biosynthetic process	GO_BiologicalProcess-EBI-UniProt-GOA-ACAP-ARAP_08.05.2020_00h00	0,00095896	0,014730037	0,00251239	0,00445949	[5, 6]	Group60	6,15	44,00
GO:0006412	translation	GO_BiologicalProcess-EBI-UniProt-GOA-ACAP-ARAP_08.05.2020_00h00	0,00240929	0,022572109	0,00251239	0,00445949	[5, 6, 7]	Group60	5,98	41,00
GO:0006402	mRNA catabolic process	GO_BiologicalProcess-EBI-UniProt-GOA-ACAP-ARAP_08.05.2020_00h00	7,0249E-05	0,004034745	0,00251239	0,00445949	[5, 6, 7, 8]	Group60	7,99	31,00
GO:0016441	posttranscriptional gene silencing	GO_BiologicalProcess-EBI-UniProt-GOA-ACAP-ARAP_08.05.2020_00h00	0,00958268	0,045209702	0,00251239	0,00445949	[6, 7]	Group60	7,88	13,00
GO:0017148	negative regulation of translation	GO_BiologicalProcess-EBI-UniProt-GOA-ACAP-ARAP_08.05.2020_00h00	0,00273635	0,024423774	0,00251239	0,00445949	[5, 6, 7, 8, 9]	Group60	9,15	13,00
GO:0061013	regulation of mRNA catabolic process	GO_BiologicalProcess-EBI-UniProt-GOA-ACAP-ARAP_08.05.2020_00h00	0,0054584	0,034833573	0,00251239	0,00445949	[5, 6, 7, 8, 9]	Group60	7,69	16,00
GO:0000289	nuclear-transcribed mRNA poly(A) tail shortening	GO_BiologicalProcess-EBI-UniProt-GOA-ACAP-ARAP_08.05.2020_00h00	0,01063148	0,047769345	0,00251239	0,00445949	[7, 8, 9, 10, 11]	Group60	13,89	5,00
GO:0061024	membrane organization	GO_BiologicalProcess-EBI-UniProt-GOA-ACAP-ARAP_08.05.2020_00h00	0,0024574	0,022860711	0,0024574	0,00447372	[4]	Group22	5,56	55,00
GO:0002474	antigen processing and presentation of peptide antigen via MHC class I	GO_BiologicalProcess-EBI-UniProt-GOA-ACAP-ARAP_08.05.2020_00h00	0,00502893	0,03372189	0,00263126	0,00455657	[4]	Group35	9,80	10,00
GO:0002478	antigen processing and presentation of exogenous peptide antigen	GO_BiologicalProcess-EBI-UniProt-GOA-ACAP-ARAP_08.05.2020_00h00	0,00171105	0,018680111	0,00263126	0,00455657	[4]	Group35	8,65	16,00
GO:0060323	head morphogenesis	GO_BiologicalProcess-EBI-UniProt-GOA-ACAP-ARAP_08.05.2020_00h00	0,00273174	0,024548518	0,00273174	0,00461795	[3, 4, 5]	Group21	15,79	6,00
GO:0006629	lipid metabolic process	GO_BiologicalProcess-EBI-UniProt-GOA-ACAP-ARAP_08.05.2020_00h00	0,00937805	0,044885512	0,00287133	0,00474103	[3]	Group65	4,93	75,00
GO:1901615	organic hydroxy compound metabolic process	GO_BiologicalProcess-EBI-UniProt-GOA-ACAP-ARAP_08.05.2020_00h00	0,00013405	0,005712426	0,00287133	0,00474103	[3]	Group65	6,86	42,00
GO:0006066	alcohol metabolic process	GO_BiologicalProcess-EBI-UniProt-GOA-ACAP-ARAP_08.05.2020_00h00	1,998E-05	0,002030259	0,00287133	0,00474103	[3, 4]	Group65	8,17	34,00
GO:0008202	steroid metabolic process	GO_BiologicalProcess-EBI-UniProt-GOA-ACAP-ARAP_08.05.2020_00h00	0,00518595	0,034082759	0,00287133	0,00474103	[4]	Group65	6,65	24,00
GO:1901617	organic hydroxy compound biosynthetic process	GO_BiologicalProcess-EBI-UniProt-GOA-ACAP-ARAP_08.05.2020_00h00	0,00201228	0,020291785	0,00287133	0,00474103	[4]	Group65	7,41	22,00
GO:0016125	sterol metabolic process	GO_BiologicalProcess-EBI-UniProt-GOA-ACAP-ARAP_08.05.2020_00h00	0,00019292	0,007079005	0,00287133	0,00474103	[4, 5]	Group65	9,84	18,00

GO:0046165	alcohol biosynthetic process	GO_BiologicalProcess-EBI-UniProt-GOA-ACAP-ARAP_08.05.2020_00h00	0,00090762	0,014445367	0,00287133	0,00474103	[4, 5]	Group65	8,90	17,00
GO:1902652	secondary alcohol metabolic process	GO_BiologicalProcess-EBI-UniProt-GOA-ACAP-ARAP_08.05.2020_00h00	0,00020651	0,006994962	0,00287133	0,00474103	[4, 5]	Group65	9,78	18,00
GO:0045338	farnesyl diphosphate metabolic process	GO_BiologicalProcess-EBI-UniProt-GOA-ACAP-ARAP_08.05.2020_00h00	0,00049996	0,010319564	0,00287133	0,00474103	[5, 6, 7]	Group65	60,00	3,00
GO:0006695	cholesterol biosynthetic process	GO_BiologicalProcess-EBI-UniProt-GOA-ACAP-ARAP_08.05.2020_00h00	0,000154	0,005983353	0,00287133	0,00474103	[6, 7]	Group65	14,10	11,00
GO:0019842	vitamin binding	GO_MolecularFunction-EBI-UniProt-GOA-ACAP-ARAP_08.05.2020_00h00	0,01053875	0,047677021	0,00353469	0,0057037	[3]	Group50	7,78	13,00
GO:0048029	monosaccharide binding	GO_MolecularFunction-EBI-UniProt-GOA-ACAP-ARAP_08.05.2020_00h00	0,00578091	0,035518978	0,00353469	0,0057037	[3]	Group50	10,23	9,00
GO:0050662	coenzyme binding	GO_MolecularFunction-EBI-UniProt-GOA-ACAP-ARAP_08.05.2020_00h00	0,0067867	0,038149916	0,00353469	0,0057037	[3]	Group50	6,69	22,00
GO:0031418	L-ascorbic acid binding	GO_MolecularFunction-EBI-UniProt-GOA-ACAP-ARAP_08.05.2020_00h00	0,00844242	0,04256656	0,00353469	0,0057037	[4, 5]	Group50	18,18	4,00
GO:0031122	cytoplasmic microtubule organization	GO_BiologicalProcess-EBI-UniProt-GOA-ACAP-ARAP_08.05.2020_00h00	0,00387927	0,029451212	0,00387927	0,00612062	[4, 5, 7]	Group02	11,76	8,00
GO:0097001	ceramide binding	GO_MolecularFunction-EBI-UniProt-GOA-ACAP-ARAP_08.05.2020_00h00	0,00489063	0,033474229	0,00527774	0,00797275	[3, 4]	Group48	21,05	4,00
GO:0005319	lipid transporter activity	GO_BiologicalProcess-EBI-UniProt-GOA-ACAP-ARAP_08.05.2020_00h00	0,00748774	0,039565202	0,00527774	0,00797275	[5, 6]	Group48	8,13	13,00
GO:0035621	ER to Golgi ceramide transport	GO_BiologicalProcess-EBI-UniProt-GOA-ACAP-ARAP_08.05.2020_00h00	0,00049996	0,010319564	0,00527774	0,00797275	[5, 6, 7]	Group48	60,00	3,00
GO:0046624	sphingolipid transporter activity	GO_BiologicalProcess-EBI-UniProt-GOA-ACAP-ARAP_08.05.2020_00h00	0,00194092	0,020188617	0,00527774	0,00797275	[6, 7]	Group48	26,67	4,00
GO:0034770	histone H4-K20 methylation	GO_BiologicalProcess-EBI-UniProt-GOA-ACAP-ARAP_08.05.2020_00h00	0,00520889	0,033896301	0,00520889	0,00803982	[7, 8, 9, 10, 11]	Group05	30,00	3,00
GO:1905288	vascular associated smooth muscle cell apoptotic process	GO_BiologicalProcess-EBI-UniProt-GOA-ACAP-ARAP_08.05.2020_00h00	0,00520889	0,033896301	0,00520889	0,00803982	[7]	Group40	30,00	3,00
GO:1905461	positive regulation of vascular associated smooth muscle cell apoptotic process	GO_BiologicalProcess-EBI-UniProt-GOA-ACAP-ARAP_08.05.2020_00h00	0,00257175	0,023592267	0,00520889	0,00803982	[8, 9, 10]	Group40	37,50	3,00
GO:0044262	cellular carbohydrate metabolic process	GO_BiologicalProcess-EBI-UniProt-GOA-ACAP-ARAP_08.05.2020_00h00	0,00702309	0,038495868	0,00702309	0,00940829	[3, 4]	Group10	6,67	22,00
GO:0045995	regulation of embryonic development	GO_BiologicalProcess-EBI-UniProt-GOA-ACAP-ARAP_08.05.2020_00h00	0,00699011	0,038474753	0,00699011	0,00954419	[4, 5]	Group11	8,51	12,00
GO:0000287	magnesium ion binding	GO_MolecularFunction-EBI-UniProt-GOA-ACAP-ARAP_08.05.2020_00h00	0,00687178	0,038141254	0,00687178	0,00956659	[5]	Group00	7,33	17,00
GO:0006972	hyperosmotic response	GO_BiologicalProcess-EBI-UniProt-GOA-ACAP-ARAP_08.05.2020_00h00	0,00733915	0,039571501	0,00733915	0,00964962	[4]	Group31	15,15	5,00
GO:0070555	response to interleukin-1	GO_BiologicalProcess-EBI-UniProt-GOA-ACAP-ARAP_08.05.2020_00h00	0,00682191	0,03802425	0,00682191	0,00968712	[5]	Group25	7,51	16,00
GO:0120193	tight junction organization	GO_BiologicalProcess-EBI-UniProt-GOA-ACAP-ARAP_08.05.2020_00h00	0,00669038	0,037931312	0,00669038	0,00969423	[6]	Group27	10,00	9,00
GO:0040014	regulation of multicellular organism growth	GO_BiologicalProcess-EBI-UniProt-GOA-ACAP-ARAP_08.05.2020_00h00	0,00762354	0,039963081	0,00762354	0,0098413	[3, 4, 5]	Group07	10,53	8,00

GO:0071346	cellular response to interferon-gamma	GO_BiologicalProcess-EBI-UniProt-GOA-ACAP-ARAP_08.05.2020_00h00	0,00668725	0,038076986	0,00668725	0,00989156	[5, 6, 7, 8]	Group39	7,73	15,00
GO:0060333	interferon-gamma-mediated signaling pathway	GO_BiologicalProcess-EBI-UniProt-GOA-ACAP-ARAP_08.05.2020_00h00	0,00350715	0,027909299	0,00668725	0,00989156	[5, 6, 7, 8, 9]	Group39	10,31	10,00
GO:0001832	blastocyst growth	GO_BiologicalProcess-EBI-UniProt-GOA-ACAP-ARAP_08.05.2020_00h00	0,00844242	0,04256656	0,00844242	0,0099902	[3, 4, 8, 9]	Group01	18,18	4,00
GO:0046686	response to cadmium ion	GO_BiologicalProcess-EBI-UniProt-GOA-ACAP-ARAP_08.05.2020_00h00	0,00823525	0,04200295	0,00823525	0,01008108	[5]	Group13	10,39	8,00
GO:0051438	regulation of ubiquitin-protein transferase activity	GO_BiologicalProcess-EBI-UniProt-GOA-ACAP-ARAP_08.05.2020_00h00	0,00843374	0,042685699	0,00843374	0,01014907	[5, 8, 9, 10, 11]	Group18	11,29	7,00
GO:0042592	homeostatic process	GO_BiologicalProcess-EBI-UniProt-GOA-ACAP-ARAP_08.05.2020_00h00	0,00822063	0,042090878	0,00822063	0,01023973	[3]	Group08	4,75	100,00
GO:0048013	ephrin receptor signaling pathway	GO_BiologicalProcess-EBI-UniProt-GOA-ACAP-ARAP_08.05.2020_00h00	0,00883455	0,043546427	0,00883455	0,01028284	[6, 7, 8]	Group14	9,57	9,00
GO:0032966	negative regulation of collagen biosynthetic process	GO_BiologicalProcess-EBI-UniProt-GOA-ACAP-ARAP_08.05.2020_00h00	0,00902835	0,044009034	0,00902835	0,01033891	[4, 5, 6]	Group03	25,00	3,00
GO:0035457	cellular response to interferon-alpha	GO_BiologicalProcess-EBI-UniProt-GOA-ACAP-ARAP_08.05.2020_00h00	0,00902835	0,044009034	0,00902835	0,01033891	[6]	Group06	25,00	3,00
GO:0006465	signal peptide processing	GO_BiologicalProcess-EBI-UniProt-GOA-ACAP-ARAP_08.05.2020_00h00	0,00902835	0,044009034	0,00902835	0,01033891	[5, 6, 7]	Group28	25,00	3,00
GO:0006334	nucleosome assembly	GO_BiologicalProcess-EBI-UniProt-GOA-ACAP-ARAP_08.05.2020_00h00	0,0096128	0,045190427	0,0096128	0,01034104	[6, 7, 8, 9]	Group17	8,16	12,00
GO:0007166	cell surface receptor signaling pathway	GO_BiologicalProcess-EBI-UniProt-GOA-ACAP-ARAP_08.05.2020_00h00	0,01122932	0,049446441	0,00816427	0,01035112	[3, 4, 5]	Group55	4,47	150,00
GO:0034097	response to cytokine	GO_BiologicalProcess-EBI-UniProt-GOA-ACAP-ARAP_08.05.2020_00h00	0,00028832	0,00793489	0,00816427	0,01035112	[4]	Group55	5,68	72,00
GO:0071310	cellular response to organic substance	GO_BiologicalProcess-EBI-UniProt-GOA-ACAP-ARAP_08.05.2020_00h00	0,00166787	0,018831223	0,00816427	0,01035112	[4]	Group55	4,76	138,00
GO:0019221	cytokine-mediated signaling pathway	GO_BiologicalProcess-EBI-UniProt-GOA-ACAP-ARAP_08.05.2020_00h00	0,00074874	0,012520107	0,00816427	0,01035112	[4, 5, 6]	Group55	6,01	50,00
GO:0071345	cellular response to cytokine stimulus	GO_BiologicalProcess-EBI-UniProt-GOA-ACAP-ARAP_08.05.2020_00h00	0,00042874	0,009439493	0,00816427	0,01035112	[5]	Group55	5,70	67,00
GO:0001959	regulation of cytokine-mediated signaling pathway	GO_BiologicalProcess-EBI-UniProt-GOA-ACAP-ARAP_08.05.2020_00h00	0,00229538	0,021972449	0,00816427	0,01035112	[4, 5, 6, 7]	Group55	8,67	15,00
GO:0014015	positive regulation of gliogenesis	GO_BiologicalProcess-EBI-UniProt-GOA-ACAP-ARAP_08.05.2020_00h00	0,00956808	0,045302629	0,00956808	0,01045129	[5, 6, 7, 8, 9, 10]	Group34	10,13	8,00
GO:0007166	cell surface receptor signaling pathway	GO_BiologicalProcess-EBI-UniProt-GOA-ACAP-ARAP_08.05.2020_00h00	0,01122932	0,049446441	0,00952971	0,01057202	[3, 4, 5]	Group68	4,47	150,00
GO:0070848	response to growth factor	GO_BiologicalProcess-EBI-UniProt-GOA-ACAP-ARAP_08.05.2020_00h00	0,00339685	0,02719537	0,00952971	0,01057202	[4]	Group68	5,76	44,00
GO:0071310	cellular response to organic substance	GO_BiologicalProcess-EBI-UniProt-GOA-ACAP-ARAP_08.05.2020_00h00	0,00166787	0,018831223	0,00952971	0,01057202	[4]	Group68	4,76	138,00
GO:0071559	response to transforming growth factor beta	GO_BiologicalProcess-EBI-UniProt-GOA-ACAP-ARAP_08.05.2020_00h00	0,0002821	0,007928841	0,00952971	0,01057202	[3, 5]	Group68	8,59	22,00
GO:0090287	regulation of cellular response to growth factor stimulus	GO_BiologicalProcess-EBI-UniProt-GOA-ACAP-ARAP_08.05.2020_00h00	0,00116796	0,016240735	0,00952971	0,01057202	[3, 4, 6]	Group68	7,59	23,00

GO:0007167	enzyme linked receptor protein signaling pathway	GO_BiologicalProcess-EBI-UniProt-GOA-ACAP-ARAP_08.05.2020_00h00	0,00289324	0,025311073	0,00952971	0,01057202	[4, 5, 6]	Group68	5,41	60,00
GO:0071363	cellular response to growth factor stimulus	GO_BiologicalProcess-EBI-UniProt-GOA-ACAP-ARAP_08.05.2020_00h00	0,00730586	0,039553437	0,00952971	0,01057202	[5]	Group68	5,60	41,00
GO:0090288	negative regulation of cellular response to growth factor stimulus	GO_BiologicalProcess-EBI-UniProt-GOA-ACAP-ARAP_08.05.2020_00h00	0,01005185	0,0464283	0,00952971	0,01057202	[3, 4, 5, 6, 7]	Group68	7,83	13,00
GO:0007169	transmembrane receptor protein tyrosine kinase signaling pathway	GO_BiologicalProcess-EBI-UniProt-GOA-ACAP-ARAP_08.05.2020_00h00	0,00339685	0,02719537	0,00952971	0,01057202	[5, 6, 7]	Group68	5,76	44,00
GO:0007179	transforming growth factor beta receptor signaling pathway	GO_BiologicalProcess-EBI-UniProt-GOA-ACAP-ARAP_08.05.2020_00h00	0,00053682	0,010744583	0,00952971	0,01057202	[5, 6, 7, 8]	Group68	9,05	18,00
GO:0090101	negative regulation of transmembrane receptor protein serine/threonine kinase signaling pathway	GO_BiologicalProcess-EBI-UniProt-GOA-ACAP-ARAP_08.05.2020_00h00	0,00893731	0,043726634	0,00952971	0,01057202	[4, 5, 6, 7, 8, 9]	Group68	8,59	11,00
GO:0017015	regulation of transforming growth factor beta receptor signaling pathway	GO_BiologicalProcess-EBI-UniProt-GOA-ACAP-ARAP_08.05.2020_00h00	0,00030373	0,008024428	0,00952971	0,01057202	[5, 6, 7, 8, 9]	Group68	11,02	14,00
GO:0051607	defense response to virus	GO_BiologicalProcess-EBI-UniProt-GOA-ACAP-ARAP_08.05.2020_00h00	0,01005658	0,046288278	0,01005658	0,01065697	[3, 4, 5, 6]	Group19	6,90	18,00
GO:0007160	cell-matrix adhesion	GO_BiologicalProcess-EBI-UniProt-GOA-ACAP-ARAP_08.05.2020_00h00	0,00948471	0,045232154	0,00948471	0,01068912	[4]	Group32	7,08	17,00
GO:0043090	amino acid import	GO_BiologicalProcess-EBI-UniProt-GOA-ACAP-ARAP_08.05.2020_00h00	0,01063148	0,047769345	0,01063148	0,01110052	[6, 8, 9]	Group09	13,89	5,00
GO:0006446	regulation of translational initiation	GO_BiologicalProcess-EBI-UniProt-GOA-ACAP-ARAP_08.05.2020_00h00	0,01105509	0,048842039	0,01105509	0,01121302	[4, 6, 7, 8, 9]	Group26	9,88	8,00
GO:0043548	phosphatidylinositol 3-kinase binding	GO_MolecularFunction-EBI-UniProt-GOA-ACAP-ARAP_08.05.2020_00h00	0,0015113	0,018149337	0,01122656	0,01122656	[3]	Group57	17,65	6,00
GO:0036312	phosphatidylinositol 3-kinase regulatory subunit binding	GO_MolecularFunction-EBI-UniProt-GOA-ACAP-ARAP_08.05.2020_00h00	0,00520889	0,033896301	0,01122656	0,01122656	[4]	Group57	30,00	3,00
GO:0043559	insulin binding	GO_MolecularFunction-EBI-UniProt-GOA-ACAP-ARAP_08.05.2020_00h00	0,00097195	0,01475805	0,01122656	0,01122656	[3, 4, 5]	Group57	50,00	3,00
GO:0048015	phosphatidylinositol-mediated signaling	GO_BiologicalProcess-EBI-UniProt-GOA-ACAP-ARAP_08.05.2020_00h00	0,00497913	0,033558346	0,01122656	0,01122656	[5, 6, 7]	Group57	7,77	16,00
GO:0014065	phosphatidylinositol 3-kinase signaling	GO_BiologicalProcess-EBI-UniProt-GOA-ACAP-ARAP_08.05.2020_00h00	0,00869579	0,043347713	0,01122656	0,01122656	[6, 7, 8]	Group57	7,98	13,00
GO:0014066	regulation of phosphatidylinositol 3-kinase signaling	GO_BiologicalProcess-EBI-UniProt-GOA-ACAP-ARAP_08.05.2020_00h00	0,00199253	0,020247202	0,01122656	0,01122656	[5, 6, 7, 8, 9]	Group57	9,49	13,00
GO:0014068	positive regulation of phosphatidylinositol 3-kinase signaling	GO_BiologicalProcess-EBI-UniProt-GOA-ACAP-ARAP_08.05.2020_00h00	0,0013313	0,017241579	0,01122656	0,01122656	[5, 6, 7, 8, 9, 10]	Group57	11,00	11,00
GO:0006915	apoptotic process	GO_BiologicalProcess-EBI-UniProt-GOA-ACAP-ARAP_08.05.2020_00h00	0,01091582	0,048551518	0,01091582	0,01123222	[4]	Group30	4,72	97,00
