# Supplementary material for: Analysis of the Role of FRMD5 in the Biology of Papillary Thyroid Carcinoma
Source: Int J Mol Sci. 2021 Jun 23;22(13):6726. doi: 10.3390/ijms22136726 (PMC8268710; doi:10.3390/ijms22136726)
Supplement: Supplementary file 1 [file ijms-22-06726-s001.zip › Supplementary Table S6.pdf]

Supplementary Table S6. Gene ontology (GO) enrichment analysis of the downregulated genes in TPC1 siFRMD5 (in comparison with TPC1 siNEG).

GOID	GOTerm	Ontology Source	Term PValue	Term PValue Corrected with Benjamini-Hochberg	Group PValue	Group PValue Corrected with Benjamini-Hochberg	GOLevels	GOGroups	% Associated Genes	Nr. Genes
GO:0033554	cellular response to stress	GO_BiologicalProcess-EBI-UniProt-GOA-ACAP-ARAP_08.05.2020_00h00	4,0841E-08	1,39335E-06	1,8284E-15	1,8467E-13	[3]	Group091	6,24	137,00
GO:0006974	cellular response to DNA damage stimulus	GO_BiologicalProcess-EBI-UniProt-GOA-ACAP-ARAP_08.05.2020_00h00	1,068E-07	2,95603E-06	1,8284E-15	1,8467E-13	[4]	Group091	7,66	70,00
GO:0006259	DNA metabolic process	GO_BiologicalProcess-EBI-UniProt-GOA-ACAP-ARAP_08.05.2020_00h00	4,5874E-07	1,01965E-05	1,8284E-15	1,8467E-13	[4, 5, 6]	Group091	7,26	73,00
GO:0051276	chromosome organization	GO_BiologicalProcess-EBI-UniProt-GOA-ACAP-ARAP_08.05.2020_00h00	7,9756E-12	1,30003E-09	1,8284E-15	1,8467E-13	[5]	Group091	7,92	104,00
GO:0051052	regulation of DNA metabolic process	GO_BiologicalProcess-EBI-UniProt-GOA-ACAP-ARAP_08.05.2020_00h00	0,00325071	0,019306853	1,8284E-15	1,8467E-13	[4, 5, 6, 7]	Group091	7,01	27,00
GO:0000723	telomere maintenance	GO_BiologicalProcess-EBI-UniProt-GOA-ACAP-ARAP_08.05.2020_00h00	0,00071636	0,00695961	1,8284E-15	1,8467E-13	[5, 6, 7]	Group091	9,60	17,00
GO:0006281	DNA repair	GO_BiologicalProcess-EBI-UniProt-GOA-ACAP-ARAP_08.05.2020_00h00	1,3176E-05	0,000210103	1,8284E-15	1,8467E-13	[5, 6, 7]	Group091	7,74	46,00
GO:0006310	DNA recombination	GO_BiologicalProcess-EBI-UniProt-GOA-ACAP-ARAP_08.05.2020_00h00	0,00279497	0,017673336	1,8284E-15	1,8467E-13	[5, 6, 7]	Group091	7,38	24,00
GO:0032200	telomere organization	GO_BiologicalProcess-EBI-UniProt-GOA-ACAP-ARAP_08.05.2020_00h00	2,0731E-06	4,00159E-05	1,8284E-15	1,8467E-13	[6]	Group091	12,11	23,00
GO:0000726	non-recombinational repair	GO_BiologicalProcess-EBI-UniProt-GOA-ACAP-ARAP_08.05.2020_00h00	3,3384E-05	0,000484896	1,8284E-15	1,8467E-13	[6, 7, 8]	Group091	13,51	15,00
GO:0006302	double-strand break repair	GO_BiologicalProcess-EBI-UniProt-GOA-ACAP-ARAP_08.05.2020_00h00	1,8599E-05	0,000281284	1,8284E-15	1,8467E-13	[6, 7, 8]	Group091	9,71	27,00
GO:0016233	telomere capping	GO_BiologicalProcess-EBI-UniProt-GOA-ACAP-ARAP_08.05.2020_00h00	0,002222	0,015232097	1,8284E-15	1,8467E-13	[6, 7, 8]	Group091	13,56	8,00
GO:0016458	gene silencing	GO_BiologicalProcess-EBI-UniProt-GOA-ACAP-ARAP_08.05.2020_00h00	3,1514E-12	7,70521E-10	2,4838E-14	1,2543E-12	[2, 5, 6, 7]	Group078	14,39	39,00
GO:0040029	regulation of gene expression, epigenetic	GO_BiologicalProcess-EBI-UniProt-GOA-ACAP-ARAP_08.05.2020_00h00	1,9685E-14	7,21956E-12	2,4838E-14	1,2543E-12	[5, 6]	Group078	14,12	48,00
GO:0045814	negative regulation of gene expression, epigenetic	GO_BiologicalProcess-EBI-UniProt-GOA-ACAP-ARAP_08.05.2020_00h00	5,7268E-20	4,20059E-17	2,4838E-14	1,2543E-12	[5, 6, 7]	Group078	28,10	34,00
GO:0034401	chromatin organization involved in regulation of transcription	GO_BiologicalProcess-EBI-UniProt-GOA-ACAP-ARAP_08.05.2020_00h00	6,8998E-21	1,0122E-17	2,4838E-14	1,2543E-12	[5, 6, 7, 8, 9, 10, 11]	Group078	24,54	40,00
GO:0006342	chromatin silencing	GO_BiologicalProcess-EBI-UniProt-GOA-ACAP-ARAP_08.05.2020_00h00	5,9328E-12	1,08793E-09	2,4838E-14	1,2543E-12	[3, 6, 7, 8, 9, 10, 11, 12, 13]	Group078	27,03	20,00
GO:0031935	regulation of chromatin silencing	GO_BiologicalProcess-EBI-UniProt-GOA-ACAP-ARAP_08.05.2020_00h00	9,3095E-05	0,001167273	2,4838E-14	1,2543E-12	[4, 5, 6, 7, 8, 9, 10, 11, 12, 13, 14]	Group078	30,00	6,00
GO:0097549	chromatin organization involved in negative regulation of transcription	GO_BiologicalProcess-EBI-UniProt-GOA-ACAP-ARAP_08.05.2020_00h00	1,0992E-18	5,37508E-16	2,4838E-14	1,2543E-12	[6, 7, 8, 9, 10, 11, 12]	Group078	24,16	36,00
GO:0060968	regulation of gene silencing	GO_BiologicalProcess-EBI-UniProt-GOA-ACAP-ARAP_08.05.2020_00h00	4,5206E-08	1,50719E-06	7,0536E-14	2,3747E-12	[3, 4, 5, 6, 7, 8]	Group072	15,49	22,00

GO:0045815	positive regulation of gene expression, epigenetic	GO_BiologicalProcess-EBI-UniProt-GOA-ACAP-ARAP_08.05.2020_00h00	4,6028E-05	0,000643077	7,0536E-14	2,3747E-12	[5, 6, 7]	Group072	16,92	11,00
GO:1902275	regulation of chromatin organization	GO_BiologicalProcess-EBI-UniProt-GOA-ACAP-ARAP_08.05.2020_00h00	0,00835802	0,035956629	7,0536E-14	2,3747E-12	[5, 6, 7]	Group072	7,77	16,00
GO:0006342	chromatin silencing	GO_BiologicalProcess-EBI-UniProt-GOA-ACAP-ARAP_08.05.2020_00h00	5,9328E-12	1,08793E-09	7,0536E-14	2,3747E-12	[3, 6, 7, 8, 9, 10, 11, 12, 13]	Group072	27,03	20,00
GO:0031935	regulation of chromatin silencing	GO_BiologicalProcess-EBI-UniProt-GOA-ACAP-ARAP_08.05.2020_00h00	9,3095E-05	0,001167273	7,0536E-14	2,3747E-12	[4, 5, 6, 7, 8, 9, 10, 11, 12, 13, 14]	Group072	30,00	6,00
GO:0006348	chromatin silencing at telomere	GO_BiologicalProcess-EBI-UniProt-GOA-ACAP-ARAP_08.05.2020_00h00	0,00609474	0,029314715	7,0536E-14	2,3747E-12	[4, 7, 8, 9, 10, 11, 12, 13, 14]	Group072	30,00	3,00
GO:0000183	rDNA heterochromatin assembly	GO_BiologicalProcess-EBI-UniProt-GOA-ACAP-ARAP_08.05.2020_00h00	1,9475E-10	2,19768E-08	1,5032E-11	3,7957E-10	[7, 8, 9, 10, 11, 12, 13, 14]	Group104	35,00	14,00
GO:0050793	regulation of developmental process	GO_BiologicalProcess-EBI-UniProt-GOA-ACAP-ARAP_08.05.2020_00h00	5,3426E-07	1,16979E-05	1,5032E-11	3,7957E-10	[2, 3]	Group104	5,74	161,00
GO:0051239	regulation of multicellular organismal process	GO_BiologicalProcess-EBI-UniProt-GOA-ACAP-ARAP_08.05.2020_00h00	1,3622E-05	0,00021487	1,5032E-11	3,7957E-10	[2, 3]	Group104	5,31	181,00
GO:0046983	protein dimerization activity	GO_MolecularFunction-EBI-UniProt-GOA-ACAP-ARAP_08.05.2020_00h00	2,6335E-07	6,43893E-06	1,5032E-11	3,7957E-10	[3]	Group104	7,16	79,00
GO:0051093	negative regulation of developmental process	GO_BiologicalProcess-EBI-UniProt-GOA-ACAP-ARAP_08.05.2020_00h00	0,00806685	0,03511592	1,5032E-11	3,7957E-10	[2, 3, 4]	Group104	5,49	58,00
GO:0051094	positive regulation of developmental process	GO_BiologicalProcess-EBI-UniProt-GOA-ACAP-ARAP_08.05.2020_00h00	0,00128977	0,010118128	1,5032E-11	3,7957E-10	[2, 3, 4]	Group104	5,55	82,00
GO:0051240	positive regulation of multicellular organismal process	GO_BiologicalProcess-EBI-UniProt-GOA-ACAP-ARAP_08.05.2020_00h00	0,00121394	0,009731382	1,5032E-11	3,7957E-10	[2, 3, 4]	Group104	5,34	102,00
GO:0022603	regulation of anatomical structure morphogenesis	GO_BiologicalProcess-EBI-UniProt-GOA-ACAP-ARAP_08.05.2020_00h00	0,00284967	0,017713821	1,5032E-11	3,7957E-10	[3, 4]	Group104	5,62	66,00
GO:0035295	tube development	GO_BiologicalProcess-EBI-UniProt-GOA-ACAP-ARAP_08.05.2020_00h00	0,00965585	0,039567392	1,5032E-11	3,7957E-10	[3, 4]	Group104	5,39	61,00
GO:0045595	regulation of cell differentiation	GO_BiologicalProcess-EBI-UniProt-GOA-ACAP-ARAP_08.05.2020_00h00	1,4649E-06	2,90403E-05	1,5032E-11	3,7957E-10	[3, 4]	Group104	6,06	120,00
GO:0048729	tissue morphogenesis	GO_BiologicalProcess-EBI-UniProt-GOA-ACAP-ARAP_08.05.2020_00h00	0,01362663	0,048285682	1,5032E-11	3,7957E-10	[3, 4]	Group104	5,69	41,00
GO:2000026	regulation of multicellular organismal development	GO_BiologicalProcess-EBI-UniProt-GOA-ACAP-ARAP_08.05.2020_00h00	1,1047E-06	2,25077E-05	1,5032E-11	3,7957E-10	[3, 4]	Group104	5,95	132,00
GO:0002520	immune system development	GO_BiologicalProcess-EBI-UniProt-GOA-ACAP-ARAP_08.05.2020_00h00	2,2993E-06	4,32451E-05	1,5032E-11	3,7957E-10	[2, 4, 5]	Group104	6,92	74,00
GO:0003677	DNA binding	GO_MolecularFunction-EBI-UniProt-GOA-ACAP-ARAP_08.05.2020_00h00	2,631E-05	0,00038987	1,5032E-11	3,7957E-10	[4]	Group104	5,47	146,00
GO:0006996	organelle organization	GO_BiologicalProcess-EBI-UniProt-GOA-ACAP-ARAP_08.05.2020_00h00	4,4619E-12	9,3508E-10	1,5032E-11	3,7957E-10	[4]	Group104	5,89	246,00
GO:0009890	negative regulation of biosynthetic process	GO_BiologicalProcess-EBI-UniProt-GOA-ACAP-ARAP_08.05.2020_00h00	4,8092E-09	2,07501E-07	1,5032E-11	3,7957E-10	[3, 4, 5]	Group104	6,77	118,00
GO:0035239	tube morphogenesis	GO_BiologicalProcess-EBI-UniProt-GOA-ACAP-ARAP_08.05.2020_00h00	0,00081955	0,007756677	1,5032E-11	3,7957E-10	[3, 4, 5]	Group104	6,15	56,00
GO:0045596	negative regulation of cell differentiation	GO_BiologicalProcess-EBI-UniProt-GOA-ACAP-ARAP_08.05.2020_00h00	0,00273721	0,01738303	1,5032E-11	3,7957E-10	[3, 4, 5]	Group104	6,06	47,00
GO:0045597	positive regulation of cell differentiation	GO_BiologicalProcess-EBI-UniProt-GOA-ACAP-ARAP_08.05.2020_00h00	0,00057936	0,005943533	1,5032E-11	3,7957E-10	[3, 4, 5]	Group104	6,03	64,00

GO:0046982	protein heterodimerization activity	GO_MolecularFunction-EBI-UniProt-GOA-ACAP-ARAP_08.05.2020_00h00	1,0363E-10	1,52032E-08	1,5032E-11	3,7957E-10	[4]	Group104	12,21	42,00
GO:0051130	positive regulation of cellular component organization	GO_BiologicalProcess-EBI-UniProt-GOA-ACAP-ARAP_08.05.2020_00h00	0,00397208	0,021988861	1,5032E-11	3,7957E-10	[3, 4, 5]	Group104	5,46	71,00
GO:0048534	hematopoietic or lymphoid organ development	GO_BiologicalProcess-EBI-UniProt-GOA-ACAP-ARAP_08.05.2020_00h00	8,8905E-07	1,89019E-05	1,5032E-11	3,7957E-10	[3, 4, 5, 6]	Group104	7,16	72,00
GO:0072359	circulatory system development	GO_BiologicalProcess-EBI-UniProt-GOA-ACAP-ARAP_08.05.2020_00h00	0,00149302	0,01123211	1,5032E-11	3,7957E-10	[4, 5]	Group104	5,75	67,00
GO:0006259	DNA metabolic process	GO_BiologicalProcess-EBI-UniProt-GOA-ACAP-ARAP_08.05.2020_00h00	4,5874E-07	1,01965E-05	1,5032E-11	3,7957E-10	[4, 5, 6]	Group104	7,26	73,00
GO:0006325	chromatin organization	GO_BiologicalProcess-EBI-UniProt-GOA-ACAP-ARAP_08.05.2020_00h00	2,6725E-13	7,84126E-11	1,5032E-11	3,7957E-10	[4, 6]	Group104	9,48	81,00
GO:0010558	negative regulation of macromolecule biosynthetic process	GO_BiologicalProcess-EBI-UniProt-GOA-ACAP-ARAP_08.05.2020_00h00	1,1945E-09	9,223E-08	1,5032E-11	3,7957E-10	[4, 5, 6]	Group104	7,00	115,00
GO:0010720	positive regulation of cell development	GO_BiologicalProcess-EBI-UniProt-GOA-ACAP-ARAP_08.05.2020_00h00	0,01184334	0,044209119	1,5032E-11	3,7957E-10	[4, 5, 6]	Group104	5,91	36,00
GO:0016458	gene silencing	GO_BiologicalProcess-EBI-UniProt-GOA-ACAP-ARAP_08.05.2020_00h00	3,1514E-12	7,70521E-10	1,5032E-11	3,7957E-10	[2, 5, 6, 7]	Group104	14,39	39,00
GO:0030334	regulation of cell migration	GO_BiologicalProcess-EBI-UniProt-GOA-ACAP-ARAP_08.05.2020_00h00	0,01196401	0,044209588	1,5032E-11	3,7957E-10	[4, 5, 6]	Group104	5,50	51,00
GO:0045934	negative regulation of nucleobase-containing compound metabolic process	GO_BiologicalProcess-EBI-UniProt-GOA-ACAP-ARAP_08.05.2020_00h00	9,9987E-09	4,19088E-07	1,5032E-11	3,7957E-10	[4, 5, 6]	Group104	6,85	110,00
GO:0051276	chromosome organization	GO_BiologicalProcess-EBI-UniProt-GOA-ACAP-ARAP_08.05.2020_00h00	7,9756E-12	1,30003E-09	1,5032E-11	3,7957E-10	[5]	Group104	7,92	104,00
GO:0065003	protein-containing complex assembly	GO_BiologicalProcess-EBI-UniProt-GOA-ACAP-ARAP_08.05.2020_00h00	1,2949E-09	9,04551E-08	1,5032E-11	3,7957E-10	[5]	Group104	6,87	121,00
GO:0071824	protein-DNA complex subunit organization	GO_BiologicalProcess-EBI-UniProt-GOA-ACAP-ARAP_08.05.2020_00h00	2,5592E-09	1,29459E-07	1,5032E-11	3,7957E-10	[5]	Group104	11,94	37,00
GO:0072358	cardiovascular system development	GO_BiologicalProcess-EBI-UniProt-GOA-ACAP-ARAP_08.05.2020_00h00	0,00195144	0,013964712	1,5032E-11	3,7957E-10	[4, 5, 6]	Group104	6,14	48,00
GO:2001020	regulation of response to DNA damage stimulus	GO_BiologicalProcess-EBI-UniProt-GOA-ACAP-ARAP_08.05.2020_00h00	0,0073145	0,032615103	1,5032E-11	3,7957E-10	[4, 5, 6]	Group104	7,53	18,00
GO:0010608	posttranscriptional regulation of gene expression	GO_BiologicalProcess-EBI-UniProt-GOA-ACAP-ARAP_08.05.2020_00h00	0,00093836	0,008445232	1,5032E-11	3,7957E-10	[5, 6]	Group104	6,47	45,00
GO:0030097	hemopoiesis	GO_BiologicalProcess-EBI-UniProt-GOA-ACAP-ARAP_08.05.2020_00h00	1,4609E-06	2,9358E-05	1,5032E-11	3,7957E-10	[4, 5, 6, 7]	Group104	7,17	69,00
GO:0040029	regulation of gene expression, epigenetic	GO_BiologicalProcess-EBI-UniProt-GOA-ACAP-ARAP_08.05.2020_00h00	1,9685E-14	7,21956E-12	1,5032E-11	3,7957E-10	[5, 6]	Group104	14,12	48,00
GO:0060968	regulation of gene silencing	GO_BiologicalProcess-EBI-UniProt-GOA-ACAP-ARAP_08.05.2020_00h00	4,5206E-08	1,50719E-06	1,5032E-11	3,7957E-10	[3, 4, 5, 6, 7, 8]	Group104	15,49	22,00
GO:1903706	regulation of hemopoiesis	GO_BiologicalProcess-EBI-UniProt-GOA-ACAP-ARAP_08.05.2020_00h00	6,525E-06	0,000110026	1,5032E-11	3,7957E-10	[3, 4, 5, 6, 7, 8]	Group104	8,27	42,00
GO:0000723	telomere maintenance	GO_BiologicalProcess-EBI-UniProt-GOA-ACAP-ARAP_08.05.2020_00h00	0,00071636	0,00695961	1,5032E-11	3,7957E-10	[5, 6, 7]	Group104	9,60	17,00
GO:0002521	leukocyte differentiation	GO_BiologicalProcess-EBI-UniProt-GOA-ACAP-ARAP_08.05.2020_00h00	0,00172079	0,012749455	1,5032E-11	3,7957E-10	[4, 5, 6, 7, 8]	Group104	6,63	37,00
GO:0006281	DNA repair	GO_BiologicalProcess-EBI-UniProt-GOA-ACAP-ARAP_08.05.2020_00h00	1,3176E-05	0,000210103	1,5032E-11	3,7957E-10	[5, 6, 7]	Group104	7,74	46,00

GO:0006333	chromatin assembly or disassembly	GO_BiologicalProcess-EBI-UniProt-GOA-ACAP-ARAP_08.05.2020_00h00	1,4678E-09	9,36214E-08	1,5032E-11	3,7957E-10	[5, 7]	Group104	13,50	32,00
GO:0006338	chromatin remodeling	GO_BiologicalProcess-EBI-UniProt-GOA-ACAP-ARAP_08.05.2020_00h00	3,8911E-07	8,78189E-06	1,5032E-11	3,7957E-10	[5, 7]	Group104	11,62	28,00
GO:0030099	myeloid cell differentiation	GO_BiologicalProcess-EBI-UniProt-GOA-ACAP-ARAP_08.05.2020_00h00	3,1838E-06	5,6273E-05	1,5032E-11	3,7957E-10	[4, 5, 6, 7, 8]	Group104	8,80	39,00
GO:0031047	gene silencing by RNA	GO_BiologicalProcess-EBI-UniProt-GOA-ACAP-ARAP_08.05.2020_00h00	0,00055964	0,005781624	1,5032E-11	3,7957E-10	[3, 6, 7, 8]	Group104	9,52	18,00
GO:0032200	telomere organization	GO_BiologicalProcess-EBI-UniProt-GOA-ACAP-ARAP_08.05.2020_00h00	2,0731E-06	4,00159E-05	1,5032E-11	3,7957E-10	[6]	Group104	12,11	23,00
GO:0034622	cellular protein-containing complex assembly	GO_BiologicalProcess-EBI-UniProt-GOA-ACAP-ARAP_08.05.2020_00h00	2,9425E-07	7,07637E-06	1,5032E-11	3,7957E-10	[6]	Group104	7,07	81,00
GO:0034728	nucleosome organization	GO_BiologicalProcess-EBI-UniProt-GOA-ACAP-ARAP_08.05.2020_00h00	3,468E-09	1,64113E-07	1,5032E-11	3,7957E-10	[5, 6, 7]	Group104	14,43	28,00
GO:0038111	interleukin-7-mediated signaling pathway	GO_BiologicalProcess-EBI-UniProt-GOA-ACAP-ARAP_08.05.2020_00h00	9,2259E-06	0,000150381	1,5032E-11	3,7957E-10	[5, 6, 7]	Group104	28,57	8,00
GO:0045814	negative regulation of gene expression, epigenetic	GO_BiologicalProcess-EBI-UniProt-GOA-ACAP-ARAP_08.05.2020_00h00	5,7268E-20	4,20059E-17	1,5032E-11	3,7957E-10	[5, 6, 7]	Group104	28,10	34,00
GO:0051253	negative regulation of RNA metabolic process	GO_BiologicalProcess-EBI-UniProt-GOA-ACAP-ARAP_08.05.2020_00h00	1,6995E-09	9,97274E-08	1,5032E-11	3,7957E-10	[4, 5, 6, 7, 8]	Group104	7,14	107,00
GO:0071103	DNA conformation change	GO_BiologicalProcess-EBI-UniProt-GOA-ACAP-ARAP_08.05.2020_00h00	6,4405E-08	1,88965E-06	1,5032E-11	3,7957E-10	[6]	Group104	10,26	39,00
GO:1901342	regulation of vasculature development	GO_BiologicalProcess-EBI-UniProt-GOA-ACAP-ARAP_08.05.2020_00h00	0,01138038	0,043590143	1,5032E-11	3,7957E-10	[4, 5, 6, 7, 8]	Group104	6,58	24,00
GO:2000113	negative regulation of cellular macromolecule biosynthetic process	GO_BiologicalProcess-EBI-UniProt-GOA-ACAP-ARAP_08.05.2020_00h00	1,8643E-09	9,76766E-08	1,5032E-11	3,7957E-10	[5, 6, 7]	Group104	7,06	110,00
GO:0030098	lymphocyte differentiation	GO_BiologicalProcess-EBI-UniProt-GOA-ACAP-ARAP_08.05.2020_00h00	0,00987359	0,039683726	1,5032E-11	3,7957E-10	[4, 5, 6, 7, 8, 9]	Group104	6,58	25,00
GO:0045637	regulation of myeloid cell differentiation	GO_BiologicalProcess-EBI-UniProt-GOA-ACAP-ARAP_08.05.2020_00h00	6,4388E-05	0,000874597	1,5032E-11	3,7957E-10	[4, 5, 6, 7, 8, 9]	Group104	9,40	25,00
GO:0048514	blood vessel morphogenesis	GO_BiologicalProcess-EBI-UniProt-GOA-ACAP-ARAP_08.05.2020_00h00	0,00257482	0,016787833	1,5032E-11	3,7957E-10	[4, 5, 6, 7, 8, 9]	Group104	6,30	41,00
GO:0050769	positive regulation of neurogenesis	GO_BiologicalProcess-EBI-UniProt-GOA-ACAP-ARAP_08.05.2020_00h00	0,0070058	0,03172072	1,5032E-11	3,7957E-10	[4, 5, 6, 7, 8, 9]	Group104	6,26	33,00
GO:0065004	protein-DNA complex assembly	GO_BiologicalProcess-EBI-UniProt-GOA-ACAP-ARAP_08.05.2020_00h00	2,7077E-10	2,8373E-08	1,5032E-11	3,7957E-10	[6, 7]	Group104	13,46	35,00
GO:0000726	non-recombinational repair	GO_BiologicalProcess-EBI-UniProt-GOA-ACAP-ARAP_08.05.2020_00h00	3,3384E-05	0,000484896	1,5032E-11	3,7957E-10	[6, 7, 8]	Group104	13,51	15,00
GO:0006302	double-strand break repair	GO_BiologicalProcess-EBI-UniProt-GOA-ACAP-ARAP_08.05.2020_00h00	1,8599E-05	0,000281284	1,5032E-11	3,7957E-10	[6, 7, 8]	Group104	9,71	27,00
GO:0006323	DNA packaging	GO_BiologicalProcess-EBI-UniProt-GOA-ACAP-ARAP_08.05.2020_00h00	1,2977E-09	8,65349E-08	1,5032E-11	3,7957E-10	[7]	Group104	12,98	34,00
GO:0016233	telomere capping	GO_BiologicalProcess-EBI-UniProt-GOA-ACAP-ARAP_08.05.2020_00h00	0,002222	0,015232097	1,5032E-11	3,7957E-10	[6, 7, 8]	Group104	13,56	8,00
GO:0016570	histone modification	GO_BiologicalProcess-EBI-UniProt-GOA-ACAP-ARAP_08.05.2020_00h00	0,00012397	0,00150305	1,5032E-11	3,7957E-10	[6, 7, 8]	Group104	7,63	37,00
GO:0030219	megakaryocyte differentiation	GO_BiologicalProcess-EBI-UniProt-GOA-ACAP-ARAP_08.05.2020_00h00	3,3816E-07	8,00123E-06	1,5032E-11	3,7957E-10	[5, 6, 7, 8, 9]	Group104	17,17	17,00

GO:0006334	nucleosome assembly	GO_BiologicalProcess-EBI-UniProt-GOA-ACAP-ARAP_08.05.2020_00h00	7,4995E-10	6,47167E-08	1,5032E-11	3,7957E-10	[6, 7, 8, 9]	Group104	17,01	25,00
GO:0018205	peptidyl-lysine modification	GO_BiologicalProcess-EBI-UniProt-GOA-ACAP-ARAP_08.05.2020_00h00	2,6372E-06	4,83594E-05	1,5032E-11	3,7957E-10	[7, 8]	Group104	8,98	38,00
GO:0045652	regulation of megakaryocyte differentiation	GO_BiologicalProcess-EBI-UniProt-GOA-ACAP-ARAP_08.05.2020_00h00	2,8934E-06	5,17628E-05	1,5032E-11	3,7957E-10	[5, 6, 7, 8, 9, 10]	Group104	17,50	14,00
GO:0034401	chromatin organization involved in regulation of transcription	GO_BiologicalProcess-EBI-UniProt-GOA-ACAP-ARAP_08.05.2020_00h00	6,8998E-21	1,0122E-17	1,5032E-11	3,7957E-10	[5, 6, 7, 8, 9, 10, 11]	Group104	24,54	40,00
GO:0035195	gene silencing by miRNA	GO_BiologicalProcess-EBI-UniProt-GOA-ACAP-ARAP_08.05.2020_00h00	0,00011795	0,001441976	1,5032E-11	3,7957E-10	[5, 8, 9, 10]	Group104	11,18	17,00
GO:0045653	negative regulation of megakaryocyte differentiation	GO_BiologicalProcess-EBI-UniProt-GOA-ACAP-ARAP_08.05.2020_00h00	4,7735E-05	0,000660629	1,5032E-11	3,7957E-10	[5, 6, 7, 8, 9, 10, 11]	Group104	33,33	6,00
GO:0045892	negative regulation of transcription, DNA-templated	GO_BiologicalProcess-EBI-UniProt-GOA-ACAP-ARAP_08.05.2020_00h00	1,4756E-09	9,01966E-08	1,5032E-11	3,7957E-10	[5, 6, 7, 8, 9, 10, 11]	Group104	7,37	99,00
GO:0060964	regulation of gene silencing by miRNA	GO_BiologicalProcess-EBI-UniProt-GOA-ACAP-ARAP_08.05.2020_00h00	1,7641E-05	0,000269584	1,5032E-11	3,7957E-10	[5, 6, 7, 8, 9, 10, 11]	Group104	13,56	16,00
GO:1903507	negative regulation of nucleic acid-templated transcription	GO_BiologicalProcess-EBI-UniProt-GOA-ACAP-ARAP_08.05.2020_00h00	2,9412E-10	2,87649E-08	1,5032E-11	3,7957E-10	[6, 7, 8, 9, 10]	Group104	7,44	104,00
GO:0006335	DNA replication-dependent nucleosome assembly	GO_BiologicalProcess-EBI-UniProt-GOA-ACAP-ARAP_08.05.2020_00h00	2,1962E-08	8,26103E-07	1,5032E-11	3,7957E-10	[7, 8, 9, 10]	Group104	34,38	11,00
GO:0034080	CENP-A containing nucleosome assembly	GO_BiologicalProcess-EBI-UniProt-GOA-ACAP-ARAP_08.05.2020_00h00	0,00185713	0,013554285	1,5032E-11	3,7957E-10	[6, 7, 8, 9, 10, 11]	Group104	15,56	7,00
GO:0006342	chromatin silencing	GO_BiologicalProcess-EBI-UniProt-GOA-ACAP-ARAP_08.05.2020_00h00	5,9328E-12	1,08793E-09	1,5032E-11	3,7957E-10	[3, 6, 7, 8, 9, 10, 11, 12, 13]	Group104	27,03	20,00
GO:0031935	regulation of chromatin silencing	GO_BiologicalProcess-EBI-UniProt-GOA-ACAP-ARAP_08.05.2020_00h00	9,3095E-05	0,001167273	1,5032E-11	3,7957E-10	[4, 5, 6, 7, 8, 9, 10, 11, 12, 13, 14]	Group104	30,00	6,00
GO:0097549	chromatin organization involved in negative regulation of transcription	GO_BiologicalProcess-EBI-UniProt-GOA-ACAP-ARAP_08.05.2020_00h00	1,0992E-18	5,37508E-16	1,5032E-11	3,7957E-10	[6, 7, 8, 9, 10, 11, 12]	Group104	24,16	36,00
GO:0031507	heterochromatin assembly	GO_BiologicalProcess-EBI-UniProt-GOA-ACAP-ARAP_08.05.2020_00h00	2,7094E-09	1,32488E-07	1,5032E-11	3,7957E-10	[6, 7, 8, 9, 10, 11, 12, 13]	Group104	21,95	18,00
GO:0048518	positive regulation of biological process	GO_BiologicalProcess-EBI-UniProt-GOA-ACAP-ARAP_08.05.2020_00h00	0,00105434	0,00899257	3,8786E-11	7,8348E-10	[1, 2, 3]	Group105	4,59	301,00
GO:0048519	negative regulation of biological process	GO_BiologicalProcess-EBI-UniProt-GOA-ACAP-ARAP_08.05.2020_00h00	1,6627E-08	6,41897E-07	3,8786E-11	7,8348E-10	[1, 2, 3]	Group105	5,20	299,00
GO:0019222	regulation of metabolic process	GO_BiologicalProcess-EBI-UniProt-GOA-ACAP-ARAP_08.05.2020_00h00	0,00015854	0,001875573	3,8786E-11	7,8348E-10	[2, 3]	Group105	4,63	338,00
GO:0003676	nucleic acid binding	GO_MolecularFunction-EBI-UniProt-GOA-ACAP-ARAP_08.05.2020_00h00	8,2827E-06	0,000136525	3,8786E-11	7,8348E-10	[3]	Group105	5,15	220,00
GO:0006725	cellular aromatic compound metabolic process	GO_BiologicalProcess-EBI-UniProt-GOA-ACAP-ARAP_08.05.2020_00h00	1,2125E-07	3,23404E-06	3,8786E-11	7,8348E-10	[3]	Group105	5,07	309,00
GO:0008134	transcription factor binding	GO_MolecularFunction-EBI-UniProt-GOA-ACAP-ARAP_08.05.2020_00h00	1,4931E-05	0,00023302	3,8786E-11	7,8348E-10	[3]	Group105	7,31	53,00
GO:0009892	negative regulation of metabolic process	GO_BiologicalProcess-EBI-UniProt-GOA-ACAP-ARAP_08.05.2020_00h00	1,4233E-07	3,66315E-06	3,8786E-11	7,8348E-10	[2, 3, 4]	Group105	5,62	191,00
GO:0009893	positive regulation of metabolic process	GO_BiologicalProcess-EBI-UniProt-GOA-ACAP-ARAP_08.05.2020_00h00	0,0014638	0,011126414	3,8786E-11	7,8348E-10	[2, 3, 4]	Group105	4,81	197,00

GO:0034641	cellular nitrogen compound metabolic process	GO_BiologicalProcess-EBI-UniProt-GOA-ACAP-ARAP_08.05.2020_00h00	1,1688E-07	3,17519E-06	3,8786E-11	7,8348E-10	[3]	Group105	5,00	331,00
GO:0043170	macromolecule metabolic process	GO_BiologicalProcess-EBI-UniProt-GOA-ACAP-ARAP_08.05.2020_00h00	8,5739E-07	1,8497E-05	3,8786E-11	7,8348E-10	[3]	Group105	4,63	455,00
GO:0044249	cellular biosynthetic process	GO_BiologicalProcess-EBI-UniProt-GOA-ACAP-ARAP_08.05.2020_00h00	1,1763E-10	1,56875E-08	3,8786E-11	7,8348E-10	[3]	Group105	5,28	333,00
GO:0046483	heterocycle metabolic process	GO_BiologicalProcess-EBI-UniProt-GOA-ACAP-ARAP_08.05.2020_00h00	5,3072E-08	1,69254E-06	3,8786E-11	7,8348E-10	[3]	Group105	5,11	309,00
GO:0048522	positive regulation of cellular process	GO_BiologicalProcess-EBI-UniProt-GOA-ACAP-ARAP_08.05.2020_00h00	6,6865E-05	0,000899924	3,8786E-11	7,8348E-10	[2, 3, 4]	Group105	4,80	286,00
GO:0048523	negative regulation of cellular process	GO_BiologicalProcess-EBI-UniProt-GOA-ACAP-ARAP_08.05.2020_00h00	1,144E-08	4,66176E-07	3,8786E-11	7,8348E-10	[2, 3, 4]	Group105	5,32	275,00
GO:0048584	positive regulation of response to stimulus	GO_BiologicalProcess-EBI-UniProt-GOA-ACAP-ARAP_08.05.2020_00h00	0,00860397	0,036691941	3,8786E-11	7,8348E-10	[2, 3, 4]	Group105	4,86	126,00
GO:1901360	organic cyclic compound metabolic process	GO_BiologicalProcess-EBI-UniProt-GOA-ACAP-ARAP_08.05.2020_00h00	9,4412E-07	1,95074E-05	3,8786E-11	7,8348E-10	[3]	Group105	4,95	314,00
GO:1901564	organonitrogen compound metabolic process	GO_BiologicalProcess-EBI-UniProt-GOA-ACAP-ARAP_08.05.2020_00h00	1,8206E-06	3,56114E-05	3,8786E-11	7,8348E-10	[3]	Group105	4,85	342,00
GO:1901576	organic substance biosynthetic process	GO_BiologicalProcess-EBI-UniProt-GOA-ACAP-ARAP_08.05.2020_00h00	3,7644E-10	3,45146E-08	3,8786E-11	7,8348E-10	[3]	Group105	5,23	335,00
GO:0006139	nucleobase-containing compound metabolic process	GO_BiologicalProcess-EBI-UniProt-GOA-ACAP-ARAP_08.05.2020_00h00	2,5057E-08	9,18966E-07	3,8786E-11	7,8348E-10	[3, 4]	Group105	5,16	303,00
GO:0009889	regulation of biosynthetic process	GO_BiologicalProcess-EBI-UniProt-GOA-ACAP-ARAP_08.05.2020_00h00	2,7732E-06	5,02265E-05	3,8786E-11	7,8348E-10	[3, 4]	Group105	5,17	233,00
GO:0019538	protein metabolic process	GO_BiologicalProcess-EBI-UniProt-GOA-ACAP-ARAP_08.05.2020_00h00	8,5578E-05	0,001091683	3,8786E-11	7,8348E-10	[3, 4]	Group105	4,77	290,00
GO:0031323	regulation of cellular metabolic process	GO_BiologicalProcess-EBI-UniProt-GOA-ACAP-ARAP_08.05.2020_00h00	6,7258E-05	0,000896979	3,8786E-11	7,8348E-10	[3, 4]	Group105	4,74	311,00
GO:0044260	cellular macromolecule metabolic process	GO_BiologicalProcess-EBI-UniProt-GOA-ACAP-ARAP_08.05.2020_00h00	8,6487E-08	2,43993E-06	3,8786E-11	7,8348E-10	[3, 4]	Group105	4,80	411,00
GO:0051171	regulation of nitrogen compound metabolic process	GO_BiologicalProcess-EBI-UniProt-GOA-ACAP-ARAP_08.05.2020_00h00	0,00052879	0,005540913	3,8786E-11	7,8348E-10	[3, 4]	Group105	4,66	287,00
GO:0060255	regulation of macromolecule metabolic process	GO_BiologicalProcess-EBI-UniProt-GOA-ACAP-ARAP_08.05.2020_00h00	0,00033958	0,003690134	3,8786E-11	7,8348E-10	[3, 4]	Group105	4,64	312,00
GO:0080090	regulation of primary metabolic process	GO_BiologicalProcess-EBI-UniProt-GOA-ACAP-ARAP_08.05.2020_00h00	0,00023645	0,002709926	3,8786E-11	7,8348E-10	[3, 4]	Group105	4,69	298,00
GO:0003677	DNA binding	GO_MolecularFunction-EBI-UniProt-GOA-ACAP-ARAP_08.05.2020_00h00	2,631E-05	0,00038987	3,8786E-11	7,8348E-10	[4]	Group105	5,47	146,00
GO:0003723	RNA binding	GO_MolecularFunction-EBI-UniProt-GOA-ACAP-ARAP_08.05.2020_00h00	0,00594192	0,02895944	3,8786E-11	7,8348E-10	[4]	Group105	5,16	90,00
GO:0009059	macromolecule biosynthetic process	GO_BiologicalProcess-EBI-UniProt-GOA-ACAP-ARAP_08.05.2020_00h00	1,3333E-08	5,28628E-07	3,8786E-11	7,8348E-10	[4]	Group105	5,30	277,00
GO:0009890	negative regulation of biosynthetic process	GO_BiologicalProcess-EBI-UniProt-GOA-ACAP-ARAP_08.05.2020_00h00	4,8092E-09	2,07501E-07	3,8786E-11	7,8348E-10	[3, 4, 5]	Group105	6,77	118,00
GO:0009891	positive regulation of biosynthetic process	GO_BiologicalProcess-EBI-UniProt-GOA-ACAP-ARAP_08.05.2020_00h00	0,00021445	0,002496809	3,8786E-11	7,8348E-10	[3, 4, 5]	Group105	5,43	120,00
GO:0010467	gene expression	GO_BiologicalProcess-EBI-UniProt-GOA-ACAP-ARAP_08.05.2020_00h00	3,6187E-05	0,000515404	3,8786E-11	7,8348E-10	[4]	Group105	4,83	289,00

GO:0010604	positive regulation of macromolecule metabolic process	GO_BiologicalProcess-EBI-UniProt-GOA-ACAP-ARAP_08.05.2020_00h00	0,00104636	0,009029456	3,8786E-11	7,8348E-10	[3, 4, 5]	Group105	4,88	185,00
GO:0010605	negative regulation of macromolecule metabolic process	GO_BiologicalProcess-EBI-UniProt-GOA-ACAP-ARAP_08.05.2020_00h00	5,4703E-08	1,70742E-06	3,8786E-11	7,8348E-10	[3, 4, 5]	Group105	5,77	180,00
GO:0018130	heterocycle biosynthetic process	GO_BiologicalProcess-EBI-UniProt-GOA-ACAP-ARAP_08.05.2020_00h00	7,8445E-10	6,39326E-08	3,8786E-11	7,8348E-10	[4]	Group105	5,60	247,00
GO:0019438	aromatic compound biosynthetic process	GO_BiologicalProcess-EBI-UniProt-GOA-ACAP-ARAP_08.05.2020_00h00	1,8552E-09	1,00801E-07	3,8786E-11	7,8348E-10	[4]	Group105	5,56	246,00
GO:0031324	negative regulation of cellular metabolic process	GO_BiologicalProcess-EBI-UniProt-GOA-ACAP-ARAP_08.05.2020_00h00	3,2119E-08	1,12185E-06	3,8786E-11	7,8348E-10	[3, 4, 5]	Group105	5,94	166,00
GO:0031325	positive regulation of cellular metabolic process	GO_BiologicalProcess-EBI-UniProt-GOA-ACAP-ARAP_08.05.2020_00h00	0,00059574	0,005985978	3,8786E-11	7,8348E-10	[3, 4, 5]	Group105	4,96	179,00
GO:0043412	macromolecule modification	GO_BiologicalProcess-EBI-UniProt-GOA-ACAP-ARAP_08.05.2020_00h00	0,00093585	0,008527306	3,8786E-11	7,8348E-10	[4]	Group105	4,78	220,00
GO:0044271	cellular nitrogen compound biosynthetic process	GO_BiologicalProcess-EBI-UniProt-GOA-ACAP-ARAP_08.05.2020_00h00	1,3139E-10	1,60621E-08	3,8786E-11	7,8348E-10	[4]	Group105	5,52	280,00
GO:0051172	negative regulation of nitrogen compound metabolic process	GO_BiologicalProcess-EBI-UniProt-GOA-ACAP-ARAP_08.05.2020_00h00	6,0798E-08	1,82022E-06	3,8786E-11	7,8348E-10	[3, 4, 5]	Group105	6,00	154,00
GO:0051173	positive regulation of nitrogen compound metabolic process	GO_BiologicalProcess-EBI-UniProt-GOA-ACAP-ARAP_08.05.2020_00h00	0,00064401	0,006426958	3,8786E-11	7,8348E-10	[3, 4, 5]	Group105	4,99	171,00
GO:1901362	organic cyclic compound biosynthetic process	GO_BiologicalProcess-EBI-UniProt-GOA-ACAP-ARAP_08.05.2020_00h00	4,7312E-09	2,10323E-07	3,8786E-11	7,8348E-10	[4]	Group105	5,48	251,00
GO:0010468	regulation of gene expression	GO_BiologicalProcess-EBI-UniProt-GOA-ACAP-ARAP_08.05.2020_00h00	0,00010021	0,001235371	3,8786E-11	7,8348E-10	[4, 5]	Group105	4,88	247,00
GO:0010556	regulation of macromolecule biosynthetic process	GO_BiologicalProcess-EBI-UniProt-GOA-ACAP-ARAP_08.05.2020_00h00	6,0315E-06	0,000102886	3,8786E-11	7,8348E-10	[4, 5]	Group105	5,18	220,00
GO:0019219	regulation of nucleobase-containing compound metabolic process	GO_BiologicalProcess-EBI-UniProt-GOA-ACAP-ARAP_08.05.2020_00h00	0,00014047	0,001689125	3,8786E-11	7,8348E-10	[4, 5]	Group105	4,97	210,00
GO:0031326	regulation of cellular biosynthetic process	GO_BiologicalProcess-EBI-UniProt-GOA-ACAP-ARAP_08.05.2020_00h00	4,6178E-06	7,9698E-05	3,8786E-11	7,8348E-10	[4, 5]	Group105	5,16	228,00
GO:0034645	cellular macromolecule biosynthetic process	GO_BiologicalProcess-EBI-UniProt-GOA-ACAP-ARAP_08.05.2020_00h00	8,1257E-08	2,33734E-06	3,8786E-11	7,8348E-10	[4, 5]	Group105	5,25	266,00
GO:0034654	nucleobase-containing compound biosynthetic process	GO_BiologicalProcess-EBI-UniProt-GOA-ACAP-ARAP_08.05.2020_00h00	1,2782E-09	9,37542E-08	3,8786E-11	7,8348E-10	[4, 5]	Group105	5,60	243,00
GO:0044267	cellular protein metabolic process	GO_BiologicalProcess-EBI-UniProt-GOA-ACAP-ARAP_08.05.2020_00h00	2,264E-06	4,31332E-05	3,8786E-11	7,8348E-10	[4, 5]	Group105	5,03	275,00
GO:0051246	regulation of protein metabolic process	GO_BiologicalProcess-EBI-UniProt-GOA-ACAP-ARAP_08.05.2020_00h00	0,00302843	0,018588707	3,8786E-11	7,8348E-10	[4, 5]	Group105	4,91	148,00
GO:0090304	nucleic acid metabolic process	GO_BiologicalProcess-EBI-UniProt-GOA-ACAP-ARAP_08.05.2020_00h00	3,5913E-07	8,232E-06	3,8786E-11	7,8348E-10	[4, 5]	Group105	5,14	273,00
GO:0003690	double-stranded DNA binding	GO_MolecularFunction-EBI-UniProt-GOA-ACAP-ARAP_08.05.2020_00h00	0,01251934	0,045460086	3,8786E-11	7,8348E-10	[5]	Group105	5,29	63,00
GO:0006325	chromatin organization	GO_BiologicalProcess-EBI-UniProt-GOA-ACAP-ARAP_08.05.2020_00h00	2,6725E-13	7,84126E-11	3,8786E-11	7,8348E-10	[4, 6]	Group105	9,48	81,00
GO:0010557	positive regulation of macromolecule biosynthetic process	GO_BiologicalProcess-EBI-UniProt-GOA-ACAP-ARAP_08.05.2020_00h00	8,2418E-05	0,001060587	3,8786E-11	7,8348E-10	[4, 5, 6]	Group105	5,60	116,00

GO:0010558	negative regulation of macromolecule biosynthetic process	GO_BiologicalProcess-EBI-UniProt-GOA-ACAP-ARAP_08.05.2020_00h00	1,1945E-09	9,223E-08	3,8786E-11	7,8348E-10	[4, 5, 6]	Group105	7,00	115,00
GO:0010629	negative regulation of gene expression	GO_BiologicalProcess-EBI-UniProt-GOA-ACAP-ARAP_08.05.2020_00h00	2,5845E-08	9,24739E-07	3,8786E-11	7,8348E-10	[4, 5, 6]	Group105	6,23	141,00
GO:0016458	gene silencing	GO_BiologicalProcess-EBI-UniProt-GOA-ACAP-ARAP_08.05.2020_00h00	3,1514E-12	7,70521E-10	3,8786E-11	7,8348E-10	[2, 5, 6, 7]	Group105	14,39	39,00
GO:0032268	regulation of cellular protein metabolic process	GO_BiologicalProcess-EBI-UniProt-GOA-ACAP-ARAP_08.05.2020_00h00	0,00110744	0,009230763	3,8786E-11	7,8348E-10	[4, 5, 6]	Group105	5,06	143,00
GO:0045934	negative regulation of nucleobase-containing compound metabolic process	GO_BiologicalProcess-EBI-UniProt-GOA-ACAP-ARAP_08.05.2020_00h00	9,9987E-09	4,19088E-07	3,8786E-11	7,8348E-10	[4, 5, 6]	Group105	6,85	110,00
GO:0045935	positive regulation of nucleobase-containing compound metabolic process	GO_BiologicalProcess-EBI-UniProt-GOA-ACAP-ARAP_08.05.2020_00h00	0,00017084	0,002004963	3,8786E-11	7,8348E-10	[4, 5, 6]	Group105	5,53	113,00
GO:0051276	chromosome organization	GO_BiologicalProcess-EBI-UniProt-GOA-ACAP-ARAP_08.05.2020_00h00	7,9756E-12	1,30003E-09	3,8786E-11	7,8348E-10	[5]	Group105	7,92	104,00
GO:0006464	cellular protein modification process	GO_BiologicalProcess-EBI-UniProt-GOA-ACAP-ARAP_08.05.2020_00h00	0,00113649	0,00941937	3,8786E-11	7,8348E-10	[5, 6]	Group105	4,79	210,00
GO:0016070	RNA metabolic process	GO_BiologicalProcess-EBI-UniProt-GOA-ACAP-ARAP_08.05.2020_00h00	3,4994E-07	8,1486E-06	3,8786E-11	7,8348E-10	[5, 6]	Group105	5,23	251,00
GO:0032270	positive regulation of cellular protein metabolic process	GO_BiologicalProcess-EBI-UniProt-GOA-ACAP-ARAP_08.05.2020_00h00	0,01391572	0,048955314	3,8786E-11	7,8348E-10	[4, 5, 6, 7]	Group105	5,02	86,00
GO:0040029	regulation of gene expression, epigenetic	GO_BiologicalProcess-EBI-UniProt-GOA-ACAP-ARAP_08.05.2020_00h00	1,9685E-14	7,21956E-12	3,8786E-11	7,8348E-10	[5, 6]	Group105	14,12	48,00
GO:0051252	regulation of RNA metabolic process	GO_BiologicalProcess-EBI-UniProt-GOA-ACAP-ARAP_08.05.2020_00h00	3,255E-05	0,000477514	3,8786E-11	7,8348E-10	[4, 5, 6, 7]	Group105	5,13	202,00
GO:2000112	regulation of cellular macromolecule biosynthetic process	GO_BiologicalProcess-EBI-UniProt-GOA-ACAP-ARAP_08.05.2020_00h00	1,5143E-05	0,000233845	3,8786E-11	7,8348E-10	[5, 6]	Group105	5,15	211,00
GO:0031399	regulation of protein modification process	GO_BiologicalProcess-EBI-UniProt-GOA-ACAP-ARAP_08.05.2020_00h00	0,00122922	0,009747351	3,8786E-11	7,8348E-10	[5, 6, 7]	Group105	5,31	105,00
GO:0045814	negative regulation of gene expression, epigenetic	GO_BiologicalProcess-EBI-UniProt-GOA-ACAP-ARAP_08.05.2020_00h00	5,7268E-20	4,20059E-17	3,8786E-11	7,8348E-10	[5, 6, 7]	Group105	28,10	34,00
GO:0051253	negative regulation of RNA metabolic process	GO_BiologicalProcess-EBI-UniProt-GOA-ACAP-ARAP_08.05.2020_00h00	1,6995E-09	9,97274E-08	3,8786E-11	7,8348E-10	[4, 5, 6, 7, 8]	Group105	7,14	107,00
GO:0051254	positive regulation of RNA metabolic process	GO_BiologicalProcess-EBI-UniProt-GOA-ACAP-ARAP_08.05.2020_00h00	0,0002428	0,002761146	3,8786E-11	7,8348E-10	[4, 5, 6, 7, 8]	Group105	5,57	104,00
GO:2000113	negative regulation of cellular macromolecule biosynthetic process	GO_BiologicalProcess-EBI-UniProt-GOA-ACAP-ARAP_08.05.2020_00h00	1,8643E-09	9,76766E-08	3,8786E-11	7,8348E-10	[5, 6, 7]	Group105	7,06	110,00
GO:0006351	transcription, DNA-templated	GO_BiologicalProcess-EBI-UniProt-GOA-ACAP-ARAP_08.05.2020_00h00	9,1187E-07	1,91103E-05	3,8786E-11	7,8348E-10	[5, 6, 7, 8, 9]	Group105	5,40	203,00
GO:0097659	nucleic acid-templated transcription	GO_BiologicalProcess-EBI-UniProt-GOA-ACAP-ARAP_08.05.2020_00h00	1,822E-07	4,53027E-06	3,8786E-11	7,8348E-10	[6, 7, 8]	Group105	5,48	209,00
GO:0006355	regulation of transcription, DNA-templated	GO_BiologicalProcess-EBI-UniProt-GOA-ACAP-ARAP_08.05.2020_00h00	7,2213E-06	0,000120383	3,8786E-11	7,8348E-10	[5, 6, 7, 8, 9, 10]	Group105	5,31	190,00
GO:1903506	regulation of nucleic acid-templated transcription	GO_BiologicalProcess-EBI-UniProt-GOA-ACAP-ARAP_08.05.2020_00h00	2,4081E-06	4,47175E-05	3,8786E-11	7,8348E-10	[6, 7, 8, 9]	Group105	5,37	196,00
GO:0006366	transcription by RNA polymerase II	GO_BiologicalProcess-EBI-UniProt-GOA-ACAP-ARAP_08.05.2020_00h00	0,00105508	0,008946861	3,8786E-11	7,8348E-10	[6, 7, 8, 9, 10]	Group105	5,17	126,00

GO:0034401	chromatin organization involved in regulation of transcription	GO_BiologicalProcess-EBI-UniProt-GOA-ACAP-ARAP_08.05.2020_00h00	6,8998E-21	1,0122E-17	3,8786E-11	7,8348E-10	[5, 6, 7, 8, 9, 10, 11]	Group105	24,54	40,00
GO:0045892	negative regulation of transcription, DNA-templated	GO_BiologicalProcess-EBI-UniProt-GOA-ACAP-ARAP_08.05.2020_00h00	1,4756E-09	9,01966E-08	3,8786E-11	7,8348E-10	[5, 6, 7, 8, 9, 10, 11]	Group105	7,37	99,00
GO:0045893	positive regulation of transcription, DNA-templated	GO_BiologicalProcess-EBI-UniProt-GOA-ACAP-ARAP_08.05.2020_00h00	0,00098052	0,008717741	3,8786E-11	7,8348E-10	[5, 6, 7, 8, 9, 10, 11]	Group105	5,48	92,00
GO:1903507	negative regulation of nucleic acid-templated transcription	GO_BiologicalProcess-EBI-UniProt-GOA-ACAP-ARAP_08.05.2020_00h00	2,9412E-10	2,87649E-08	3,8786E-11	7,8348E-10	[6, 7, 8, 9, 10]	Group105	7,44	104,00
GO:1903508	positive regulation of nucleic acid-templated transcription	GO_BiologicalProcess-EBI-UniProt-GOA-ACAP-ARAP_08.05.2020_00h00	9,8332E-05	0,001222489	3,8786E-11	7,8348E-10	[6, 7, 8, 9, 10]	Group105	5,73	102,00
GO:0003712	transcription coregulator activity	GO_BiologicalProcess-EBI-UniProt-GOA-ACAP-ARAP_08.05.2020_00h00	5,4984E-08	1,68046E-06	3,8786E-11	7,8348E-10	[7, 8, 9, 10]	Group105	8,65	55,00
GO:0006357	regulation of transcription by RNA polymerase II	GO_BiologicalProcess-EBI-UniProt-GOA-ACAP-ARAP_08.05.2020_00h00	0,0060812	0,029345805	3,8786E-11	7,8348E-10	[6, 7, 8, 9, 10, 11]	Group105	4,98	114,00
GO:0000122	negative regulation of transcription by RNA polymerase II	GO_BiologicalProcess-EBI-UniProt-GOA-ACAP-ARAP_08.05.2020_00h00	0,01389223	0,048990164	3,8786E-11	7,8348E-10	[6, 7, 8, 9, 10, 11, 12]	Group105	5,45	51,00
GO:0003713	transcription coactivator activity	GO_BiologicalProcess-EBI-UniProt-GOA-ACAP-ARAP_08.05.2020_00h00	1,168E-05	0,000188293	3,8786E-11	7,8348E-10	[7, 8, 9, 10, 11]	Group105	8,99	33,00
GO:0097549	chromatin organization involved in negative regulation of transcription	GO_BiologicalProcess-EBI-UniProt-GOA-ACAP-ARAP_08.05.2020_00h00	1,0992E-18	5,37508E-16	3,8786E-11	7,8348E-10	[6, 7, 8, 9, 10, 11, 12]	Group105	24,16	36,00
GO:0016043	cellular component organization	GO_BiologicalProcess-EBI-UniProt-GOA-ACAP-ARAP_08.05.2020_00h00	1,8257E-09	1,03013E-07	2,4557E-10	4,1337E-09	[3]	Group080	5,14	342,00
GO:0044085	cellular component biogenesis	GO_BiologicalProcess-EBI-UniProt-GOA-ACAP-ARAP_08.05.2020_00h00	5,2892E-08	1,72428E-06	2,4557E-10	4,1337E-09	[3]	Group080	5,69	191,00
GO:0006996	organelle organization	GO_BiologicalProcess-EBI-UniProt-GOA-ACAP-ARAP_08.05.2020_00h00	4,4619E-12	9,3508E-10	2,4557E-10	4,1337E-09	[4]	Group080	5,89	246,00
GO:0022607	cellular component assembly	GO_BiologicalProcess-EBI-UniProt-GOA-ACAP-ARAP_08.05.2020_00h00	1,375E-07	3,60206E-06	2,4557E-10	4,1337E-09	[4]	Group080	5,71	178,00
GO:0043933	protein-containing complex subunit organization	GO_BiologicalProcess-EBI-UniProt-GOA-ACAP-ARAP_08.05.2020_00h00	3,9924E-09	1,83027E-07	2,4557E-10	4,1337E-09	[4]	Group080	6,52	134,00
GO:0051276	chromosome organization	GO_BiologicalProcess-EBI-UniProt-GOA-ACAP-ARAP_08.05.2020_00h00	7,9756E-12	1,30003E-09	2,4557E-10	4,1337E-09	[5]	Group080	7,92	104,00
GO:0065003	protein-containing complex assembly	GO_BiologicalProcess-EBI-UniProt-GOA-ACAP-ARAP_08.05.2020_00h00	1,2949E-09	9,04551E-08	2,4557E-10	4,1337E-09	[5]	Group080	6,87	121,00
GO:0034622	cellular protein-containing complex assembly	GO_BiologicalProcess-EBI-UniProt-GOA-ACAP-ARAP_08.05.2020_00h00	2,9425E-07	7,07637E-06	2,4557E-10	4,1337E-09	[6]	Group080	7,07	81,00
GO:0006325	chromatin organization	GO_BiologicalProcess-EBI-UniProt-GOA-ACAP-ARAP_08.05.2020_00h00	2,6725E-13	7,84126E-11	6,6803E-10	9,6387E-09	[4, 6]	Group095	9,48	81,00
GO:0051276	chromosome organization	GO_BiologicalProcess-EBI-UniProt-GOA-ACAP-ARAP_08.05.2020_00h00	7,9756E-12	1,30003E-09	6,6803E-10	9,6387E-09	[5]	Group095	7,92	104,00
GO:0033044	regulation of chromosome organization	GO_BiologicalProcess-EBI-UniProt-GOA-ACAP-ARAP_08.05.2020_00h00	0,0056442	0,028067946	6,6803E-10	9,6387E-09	[5, 6]	Group095	6,81	26,00
GO:0006479	protein methylation	GO_BiologicalProcess-EBI-UniProt-GOA-ACAP-ARAP_08.05.2020_00h00	0,00525204	0,026568068	6,6803E-10	9,6387E-09	[4, 5, 6, 7, 8]	Group095	8,16	16,00
GO:1902275	regulation of chromatin organization	GO_BiologicalProcess-EBI-UniProt-GOA-ACAP-ARAP_08.05.2020_00h00	0,00835802	0,035956629	6,6803E-10	9,6387E-09	[5, 6, 7]	Group095	7,77	16,00

GO:0018193	peptidyl-amino acid modification	GO_BiologicalProcess-EBI-UniProt-GOA-ACAP-ARAP_08.05.2020_00h00	9,2461E-05	0,001169311	6,6803E-10	9,6387E-09	[6, 7]	Group095	6,02	83,00
GO:0043543	protein acylation	GO_BiologicalProcess-EBI-UniProt-GOA-ACAP-ARAP_08.05.2020_00h00	0,00368337	0,02110742	6,6803E-10	9,6387E-09	[6, 7]	Group095	7,72	20,00
GO:0016570	histone modification	GO_BiologicalProcess-EBI-UniProt-GOA-ACAP-ARAP_08.05.2020_00h00	0,00012397	0,00150305	6,6803E-10	9,6387E-09	[6, 7, 8]	Group095	7,63	37,00
GO:0016571	histone methylation	GO_BiologicalProcess-EBI-UniProt-GOA-ACAP-ARAP_08.05.2020_00h00	0,00283803	0,017792288	6,6803E-10	9,6387E-09	[5, 6, 7, 8, 9]	Group095	9,27	14,00
GO:0018022	peptidyl-lysine methylation	GO_BiologicalProcess-EBI-UniProt-GOA-ACAP-ARAP_08.05.2020_00h00	0,00439728	0,023121173	6,6803E-10	9,6387E-09	[5, 6, 7, 8, 9]	Group095	9,15	13,00
GO:0018205	peptidyl-lysine modification	GO_BiologicalProcess-EBI-UniProt-GOA-ACAP-ARAP_08.05.2020_00h00	2,6372E-06	4,83594E-05	6,6803E-10	9,6387E-09	[7, 8]	Group095	8,98	38,00
GO:0006475	internal protein amino acid acetylation	GO_BiologicalProcess-EBI-UniProt-GOA-ACAP-ARAP_08.05.2020_00h00	0,00191473	0,01390548	6,6803E-10	9,6387E-09	[8, 9]	Group095	9,04	16,00
GO:0016573	histone acetylation	GO_BiologicalProcess-EBI-UniProt-GOA-ACAP-ARAP_08.05.2020_00h00	0,00262382	0,01703164	6,6803E-10	9,6387E-09	[7, 8, 9, 10, 11]	Group095	9,04	15,00
GO:0031935	regulation of chromatin silencing	GO_BiologicalProcess-EBI-UniProt-GOA-ACAP-ARAP_08.05.2020_00h00	9,3095E-05	0,001167273	6,6803E-10	9,6387E-09	[4, 5, 6, 7, 8, 9, 10, 11, 12, 13, 14]	Group095	30,00	6,00
GO:0070734	histone H3-K27 methylation	GO_BiologicalProcess-EBI-UniProt-GOA-ACAP-ARAP_08.05.2020_00h00	0,01397906	0,049060475	6,6803E-10	9,6387E-09	[7, 8, 9, 10, 11]	Group095	16,67	4,00
GO:0006352	DNA-templated transcription, initiation	GO_BiologicalProcess-EBI-UniProt-GOA-ACAP-ARAP_08.05.2020_00h00	1,7056E-07	4,31389E-06	8,8085E-09	1,1121E-07	[6, 7, 8, 9, 10]	Group049	11,31	31,00
GO:0003713	transcription coactivator activity	GO_BiologicalProcess-EBI-UniProt-GOA-ACAP-ARAP_08.05.2020_00h00	1,168E-05	0,000188293	8,8085E-09	1,1121E-07	[7, 8, 9, 10, 11]	Group049	8,99	33,00
GO:0006367	transcription initiation from RNA polymerase II promoter	GO_BiologicalProcess-EBI-UniProt-GOA-ACAP-ARAP_08.05.2020_00h00	0,0006665	0,006562146	8,8085E-09	1,1121E-07	[7, 8, 9, 10, 11]	Group049	9,13	19,00
GO:0009890	negative regulation of biosynthetic process	GO_BiologicalProcess-EBI-UniProt-GOA-ACAP-ARAP_08.05.2020_00h00	4,8092E-09	2,07501E-07	4,5037E-08	5,0541E-07	[3, 4, 5]	Group094	6,77	118,00
GO:0031324	negative regulation of cellular metabolic process	GO_BiologicalProcess-EBI-UniProt-GOA-ACAP-ARAP_08.05.2020_00h00	3,2119E-08	1,12185E-06	4,5037E-08	5,0541E-07	[3, 4, 5]	Group094	5,94	166,00
GO:0051172	negative regulation of nitrogen compound metabolic process	GO_BiologicalProcess-EBI-UniProt-GOA-ACAP-ARAP_08.05.2020_00h00	6,0798E-08	1,82022E-06	4,5037E-08	5,0541E-07	[3, 4, 5]	Group094	6,00	154,00
GO:0003690	double-stranded DNA binding	GO_MolecularFunction-EBI-UniProt-GOA-ACAP-ARAP_08.05.2020_00h00	0,01251934	0,045460086	4,5037E-08	5,0541E-07	[5]	Group094	5,29	63,00
GO:0010558	negative regulation of macromolecule biosynthetic process	GO_BiologicalProcess-EBI-UniProt-GOA-ACAP-ARAP_08.05.2020_00h00	1,1945E-09	9,223E-08	4,5037E-08	5,0541E-07	[4, 5, 6]	Group094	7,00	115,00
GO:0010629	negative regulation of gene expression	GO_BiologicalProcess-EBI-UniProt-GOA-ACAP-ARAP_08.05.2020_00h00	2,5845E-08	9,24739E-07	4,5037E-08	5,0541E-07	[4, 5, 6]	Group094	6,23	141,00
GO:0045934	negative regulation of nucleobase-containing compound metabolic process	GO_BiologicalProcess-EBI-UniProt-GOA-ACAP-ARAP_08.05.2020_00h00	9,9987E-09	4,19088E-07	4,5037E-08	5,0541E-07	[4, 5, 6]	Group094	6,85	110,00
GO:0051253	negative regulation of RNA metabolic process	GO_BiologicalProcess-EBI-UniProt-GOA-ACAP-ARAP_08.05.2020_00h00	1,6995E-09	9,97274E-08	4,5037E-08	5,0541E-07	[4, 5, 6, 7, 8]	Group094	7,14	107,00
GO:2000113	negative regulation of cellular macromolecule biosynthetic process	GO_BiologicalProcess-EBI-UniProt-GOA-ACAP-ARAP_08.05.2020_00h00	1,8643E-09	9,76766E-08	4,5037E-08	5,0541E-07	[5, 6, 7]	Group094	7,06	110,00
GO:0006366	transcription by RNA polymerase II	GO_BiologicalProcess-EBI-UniProt-GOA-ACAP-ARAP_08.05.2020_00h00	0,00105508	0,008946861	4,5037E-08	5,0541E-07	[6, 7, 8, 9, 10]	Group094	5,17	126,00

GO:0045892	negative regulation of transcription, DNA-templated	GO_BiologicalProcess-EBI-UniProt-GOA-ACAP-ARAP_08.05.2020_00h00	1,4756E-09	9,01966E-08	4,5037E-08	5,0541E-07	[5, 6, 7, 8, 9, 10, 11]	Group094	7,37	99,00
GO:1903507	negative regulation of nucleic acid-templated transcription	GO_BiologicalProcess-EBI-UniProt-GOA-ACAP-ARAP_08.05.2020_00h00	2,9412E-10	2,87649E-08	4,5037E-08	5,0541E-07	[6, 7, 8, 9, 10]	Group094	7,44	104,00
GO:0006357	regulation of transcription by RNA polymerase II	GO_BiologicalProcess-EBI-UniProt-GOA-ACAP-ARAP_08.05.2020_00h00	0,0060812	0,029345805	4,5037E-08	5,0541E-07	[6, 7, 8, 9, 10, 11]	Group094	4,98	114,00
GO:0000122	negative regulation of transcription by RNA polymerase II	GO_BiologicalProcess-EBI-UniProt-GOA-ACAP-ARAP_08.05.2020_00h00	0,01389223	0,048990164	4,5037E-08	5,0541E-07	[6, 7, 8, 9, 10, 11, 12]	Group094	5,45	51,00
GO:0048518	positive regulation of biological process	GO_BiologicalProcess-EBI-UniProt-GOA-ACAP-ARAP_08.05.2020_00h00	0,00105434	0,00899257	2,1593E-07	1,9826E-06	[1, 2, 3]	Group102	4,59	301,00
GO:0019222	regulation of metabolic process	GO_BiologicalProcess-EBI-UniProt-GOA-ACAP-ARAP_08.05.2020_00h00	0,00015854	0,001875573	2,1593E-07	1,9826E-06	[2, 3]	Group102	4,63	338,00
GO:0023051	regulation of signaling	GO_BiologicalProcess-EBI-UniProt-GOA-ACAP-ARAP_08.05.2020_00h00	0,00106459	0,008975607	2,1593E-07	1,9826E-06	[2, 3]	Group102	4,87	187,00
GO:0048583	regulation of response to stimulus	GO_BiologicalProcess-EBI-UniProt-GOA-ACAP-ARAP_08.05.2020_00h00	0,0140207	0,049089178	2,1593E-07	1,9826E-06	[2, 3]	Group102	4,55	206,00
GO:0051239	regulation of multicellular organismal process	GO_BiologicalProcess-EBI-UniProt-GOA-ACAP-ARAP_08.05.2020_00h00	1,3622E-05	0,00021487	2,1593E-07	1,9826E-06	[2, 3]	Group102	5,31	181,00
GO:0009893	positive regulation of metabolic process	GO_BiologicalProcess-EBI-UniProt-GOA-ACAP-ARAP_08.05.2020_00h00	0,0014638	0,011126414	2,1593E-07	1,9826E-06	[2, 3, 4]	Group102	4,81	197,00
GO:0023056	positive regulation of signaling	GO_BiologicalProcess-EBI-UniProt-GOA-ACAP-ARAP_08.05.2020_00h00	0,00616386	0,029550245	2,1593E-07	1,9826E-06	[2, 3, 4]	Group102	5,05	103,00
GO:0023057	negative regulation of signaling	GO_BiologicalProcess-EBI-UniProt-GOA-ACAP-ARAP_08.05.2020_00h00	0,0039485	0,02194107	2,1593E-07	1,9826E-06	[2, 3, 4]	Group102	5,34	81,00
GO:0043170	macromolecule metabolic process	GO_BiologicalProcess-EBI-UniProt-GOA-ACAP-ARAP_08.05.2020_00h00	8,5739E-07	1,8497E-05	2,1593E-07	1,9826E-06	[3]	Group102	4,63	455,00
GO:0044092	negative regulation of molecular function	GO_BiologicalProcess-EBI-UniProt-GOA-ACAP-ARAP_08.05.2020_00h00	0,00552191	0,027553212	2,1593E-07	1,9826E-06	[3]	Group102	5,43	68,00
GO:0048522	positive regulation of cellular process	GO_BiologicalProcess-EBI-UniProt-GOA-ACAP-ARAP_08.05.2020_00h00	6,6865E-05	0,000899924	2,1593E-07	1,9826E-06	[2, 3, 4]	Group102	4,80	286,00
GO:0048584	positive regulation of response to stimulus	GO_BiologicalProcess-EBI-UniProt-GOA-ACAP-ARAP_08.05.2020_00h00	0,00860397	0,036691941	2,1593E-07	1,9826E-06	[2, 3, 4]	Group102	4,86	126,00
GO:0051240	positive regulation of multicellular organismal process	GO_BiologicalProcess-EBI-UniProt-GOA-ACAP-ARAP_08.05.2020_00h00	0,00121394	0,009731382	2,1593E-07	1,9826E-06	[2, 3, 4]	Group102	5,34	102,00
GO:1901564	organonitrogen compound metabolic process	GO_BiologicalProcess-EBI-UniProt-GOA-ACAP-ARAP_08.05.2020_00h00	1,8206E-06	3,56114E-05	2,1593E-07	1,9826E-06	[3]	Group102	4,85	342,00
GO:0010646	regulation of cell communication	GO_BiologicalProcess-EBI-UniProt-GOA-ACAP-ARAP_08.05.2020_00h00	0,00083746	0,007875319	2,1593E-07	1,9826E-06	[3, 4]	Group102	4,90	186,00
GO:0010941	regulation of cell death	GO_BiologicalProcess-EBI-UniProt-GOA-ACAP-ARAP_08.05.2020_00h00	0,00652066	0,03065965	2,1593E-07	1,9826E-06	[3, 4]	Group102	5,13	92,00
GO:0019538	protein metabolic process	GO_BiologicalProcess-EBI-UniProt-GOA-ACAP-ARAP_08.05.2020_00h00	8,5578E-05	0,001091683	2,1593E-07	1,9826E-06	[3, 4]	Group102	4,77	290,00
GO:0031323	regulation of cellular metabolic process	GO_BiologicalProcess-EBI-UniProt-GOA-ACAP-ARAP_08.05.2020_00h00	6,7258E-05	0,000896979	2,1593E-07	1,9826E-06	[3, 4]	Group102	4,74	311,00
GO:0044260	cellular macromolecule metabolic process	GO_BiologicalProcess-EBI-UniProt-GOA-ACAP-ARAP_08.05.2020_00h00	8,6487E-08	2,43993E-06	2,1593E-07	1,9826E-06	[3, 4]	Group102	4,80	411,00

GO:0051171	regulation of nitrogen compound metabolic process	GO_BiologicalProcess-EBI-UniProt-GOA-ACAP-ARAP_08.05.2020_00h00	0,00052879	0,005540913	2,1593E-07	1,9826E-06	[3, 4]	Group102	4,66	287,00
GO:0060255	regulation of macromolecule metabolic process	GO_BiologicalProcess-EBI-UniProt-GOA-ACAP-ARAP_08.05.2020_00h00	0,00033958	0,003690134	2,1593E-07	1,9826E-06	[3, 4]	Group102	4,64	312,00
GO:0080090	regulation of primary metabolic process	GO_BiologicalProcess-EBI-UniProt-GOA-ACAP-ARAP_08.05.2020_00h00	0,00023645	0,002709926	2,1593E-07	1,9826E-06	[3, 4]	Group102	4,69	298,00
GO:0006796	phosphate-containing compound metabolic process	GO_BiologicalProcess-EBI-UniProt-GOA-ACAP-ARAP_08.05.2020_00h00	0,0036411	0,021029476	2,1593E-07	1,9826E-06	[4]	Group102	4,81	168,00
GO:0009891	positive regulation of biosynthetic process	GO_BiologicalProcess-EBI-UniProt-GOA-ACAP-ARAP_08.05.2020_00h00	0,00021445	0,002496809	2,1593E-07	1,9826E-06	[3, 4, 5]	Group102	5,43	120,00
GO:0009966	regulation of signal transduction	GO_BiologicalProcess-EBI-UniProt-GOA-ACAP-ARAP_08.05.2020_00h00	0,00121141	0,009764508	2,1593E-07	1,9826E-06	[3, 4, 5]	Group102	4,95	163,00
GO:0010604	positive regulation of macromolecule metabolic process	GO_BiologicalProcess-EBI-UniProt-GOA-ACAP-ARAP_08.05.2020_00h00	0,00104636	0,009029456	2,1593E-07	1,9826E-06	[3, 4, 5]	Group102	4,88	185,00
GO:0010605	negative regulation of macromolecule metabolic process	GO_BiologicalProcess-EBI-UniProt-GOA-ACAP-ARAP_08.05.2020_00h00	5,4703E-08	1,70742E-06	2,1593E-07	1,9826E-06	[3, 4, 5]	Group102	5,77	180,00
GO:0010647	positive regulation of cell communication	GO_BiologicalProcess-EBI-UniProt-GOA-ACAP-ARAP_08.05.2020_00h00	0,00550487	0,027561951	2,1593E-07	1,9826E-06	[3, 4, 5]	Group102	5,07	103,00
GO:0010648	negative regulation of cell communication	GO_BiologicalProcess-EBI-UniProt-GOA-ACAP-ARAP_08.05.2020_00h00	0,00375159	0,02124938	2,1593E-07	1,9826E-06	[3, 4, 5]	Group102	5,35	81,00
GO:0031324	negative regulation of cellular metabolic process	GO_BiologicalProcess-EBI-UniProt-GOA-ACAP-ARAP_08.05.2020_00h00	3,2119E-08	1,12185E-06	2,1593E-07	1,9826E-06	[3, 4, 5]	Group102	5,94	166,00
GO:0031325	positive regulation of cellular metabolic process	GO_BiologicalProcess-EBI-UniProt-GOA-ACAP-ARAP_08.05.2020_00h00	0,00059574	0,005985978	2,1593E-07	1,9826E-06	[3, 4, 5]	Group102	4,96	179,00
GO:0043412	macromolecule modification	GO_BiologicalProcess-EBI-UniProt-GOA-ACAP-ARAP_08.05.2020_00h00	0,00093585	0,008527306	2,1593E-07	1,9826E-06	[4]	Group102	4,78	220,00
GO:0051172	negative regulation of nitrogen compound metabolic process	GO_BiologicalProcess-EBI-UniProt-GOA-ACAP-ARAP_08.05.2020_00h00	6,0798E-08	1,82022E-06	2,1593E-07	1,9826E-06	[3, 4, 5]	Group102	6,00	154,00
GO:0051173	positive regulation of nitrogen compound metabolic process	GO_BiologicalProcess-EBI-UniProt-GOA-ACAP-ARAP_08.05.2020_00h00	0,00064401	0,006426958	2,1593E-07	1,9826E-06	[3, 4, 5]	Group102	4,99	171,00
GO:0009967	positive regulation of signal transduction	GO_BiologicalProcess-EBI-UniProt-GOA-ACAP-ARAP_08.05.2020_00h00	0,00303475	0,018472932	2,1593E-07	1,9826E-06	[3, 4, 5, 6]	Group102	5,26	93,00
GO:0009968	negative regulation of signal transduction	GO_BiologicalProcess-EBI-UniProt-GOA-ACAP-ARAP_08.05.2020_00h00	0,00653157	0,030612811	2,1593E-07	1,9826E-06	[3, 4, 5, 6]	Group102	5,33	72,00
GO:0044267	cellular protein metabolic process	GO_BiologicalProcess-EBI-UniProt-GOA-ACAP-ARAP_08.05.2020_00h00	2,264E-06	4,31332E-05	2,1593E-07	1,9826E-06	[4, 5]	Group102	5,03	275,00
GO:0051246	regulation of protein metabolic process	GO_BiologicalProcess-EBI-UniProt-GOA-ACAP-ARAP_08.05.2020_00h00	0,00302843	0,018588707	2,1593E-07	1,9826E-06	[4, 5]	Group102	4,91	148,00
GO:0010557	positive regulation of macromolecule biosynthetic process	GO_BiologicalProcess-EBI-UniProt-GOA-ACAP-ARAP_08.05.2020_00h00	8,2418E-05	0,001060587	2,1593E-07	1,9826E-06	[4, 5, 6]	Group102	5,60	116,00
GO:0016310	phosphorylation	GO_BiologicalProcess-EBI-UniProt-GOA-ACAP-ARAP_08.05.2020_00h00	0,009714	0,039694816	2,1593E-07	1,9826E-06	[5]	Group102	4,85	125,00
GO:0032268	regulation of cellular protein metabolic process	GO_BiologicalProcess-EBI-UniProt-GOA-ACAP-ARAP_08.05.2020_00h00	0,00110744	0,009230763	2,1593E-07	1,9826E-06	[4, 5, 6]	Group102	5,06	143,00
GO:0045935	positive regulation of nucleobase-containing compound metabolic process	GO_BiologicalProcess-EBI-UniProt-GOA-ACAP-ARAP_08.05.2020_00h00	0,00017084	0,002004963	2,1593E-07	1,9826E-06	[4, 5, 6]	Group102	5,53	113,00

GO:0006464	cellular protein modification process	GO_BiologicalProcess-EBI-UniProt-GOA-ACAP-ARAP_08.05.2020_00h00	0,00113649	0,00941937	2,1593E-07	1,9826E-06	[5, 6]	Group102	4,79	210,00
GO:0019220	regulation of phosphate metabolic process	GO_BiologicalProcess-EBI-UniProt-GOA-ACAP-ARAP_08.05.2020_00h00	0,00670985	0,031051561	2,1593E-07	1,9826E-06	[5, 6]	Group102	5,08	98,00
GO:0032270	positive regulation of cellular protein metabolic process	GO_BiologicalProcess-EBI-UniProt-GOA-ACAP-ARAP_08.05.2020_00h00	0,01391572	0,048955314	2,1593E-07	1,9826E-06	[4, 5, 6, 7]	Group102	5,02	86,00
GO:0042981	regulation of apoptotic process	GO_BiologicalProcess-EBI-UniProt-GOA-ACAP-ARAP_08.05.2020_00h00	0,00142297	0,010872362	2,1593E-07	1,9826E-06	[5, 6]	Group102	5,44	89,00
GO:0031399	regulation of protein modification process	GO_BiologicalProcess-EBI-UniProt-GOA-ACAP-ARAP_08.05.2020_00h00	0,00122922	0,009747351	2,1593E-07	1,9826E-06	[5, 6, 7]	Group102	5,31	105,00
GO:0045936	negative regulation of phosphate metabolic process	GO_BiologicalProcess-EBI-UniProt-GOA-ACAP-ARAP_08.05.2020_00h00	0,00908706	0,037979245	2,1593E-07	1,9826E-06	[5, 6, 7]	Group102	6,02	36,00
GO:0051254	positive regulation of RNA metabolic process	GO_BiologicalProcess-EBI-UniProt-GOA-ACAP-ARAP_08.05.2020_00h00	0,0002428	0,002761146	2,1593E-07	1,9826E-06	[4, 5, 6, 7, 8]	Group102	5,57	104,00
GO:0018193	peptidyl-amino acid modification	GO_BiologicalProcess-EBI-UniProt-GOA-ACAP-ARAP_08.05.2020_00h00	9,2461E-05	0,001169311	2,1593E-07	1,9826E-06	[6, 7]	Group102	6,02	83,00
GO:0031400	negative regulation of protein modification process	GO_BiologicalProcess-EBI-UniProt-GOA-ACAP-ARAP_08.05.2020_00h00	0,00745149	0,03282683	2,1593E-07	1,9826E-06	[5, 6, 7, 8]	Group102	6,02	38,00
GO:0042325	regulation of phosphorylation	GO_BiologicalProcess-EBI-UniProt-GOA-ACAP-ARAP_08.05.2020_00h00	0,0058829	0,028960451	2,1593E-07	1,9826E-06	[6, 7]	Group102	5,17	89,00
GO:0001932	regulation of protein phosphorylation	GO_BiologicalProcess-EBI-UniProt-GOA-ACAP-ARAP_08.05.2020_00h00	0,01202917	0,044227567	2,1593E-07	1,9826E-06	[6, 7, 8]	Group102	5,12	79,00
GO:0044085	cellular component biogenesis	GO_BiologicalProcess-EBI-UniProt-GOA-ACAP-ARAP_08.05.2020_00h00	5,2892E-08	1,72428E-06	1,9697E-07	1,9894E-06	[3]	Group089	5,69	191,00
GO:0022607	cellular component assembly	GO_BiologicalProcess-EBI-UniProt-GOA-ACAP-ARAP_08.05.2020_00h00	1,375E-07	3,60206E-06	1,9697E-07	1,9894E-06	[4]	Group089	5,71	178,00
GO:0043933	protein-containing complex subunit organization	GO_BiologicalProcess-EBI-UniProt-GOA-ACAP-ARAP_08.05.2020_00h00	3,9924E-09	1,83027E-07	1,9697E-07	1,9894E-06	[4]	Group089	6,52	134,00
GO:0065003	protein-containing complex assembly	GO_BiologicalProcess-EBI-UniProt-GOA-ACAP-ARAP_08.05.2020_00h00	1,2949E-09	9,04551E-08	1,9697E-07	1,9894E-06	[5]	Group089	6,87	121,00
GO:0071824	protein-DNA complex subunit organization	GO_BiologicalProcess-EBI-UniProt-GOA-ACAP-ARAP_08.05.2020_00h00	2,5592E-09	1,29459E-07	1,9697E-07	1,9894E-06	[5]	Group089	11,94	37,00
GO:0006333	chromatin assembly or disassembly	GO_BiologicalProcess-EBI-UniProt-GOA-ACAP-ARAP_08.05.2020_00h00	1,4678E-09	9,36214E-08	1,9697E-07	1,9894E-06	[5, 7]	Group089	13,50	32,00
GO:0034622	cellular protein-containing complex assembly	GO_BiologicalProcess-EBI-UniProt-GOA-ACAP-ARAP_08.05.2020_00h00	2,9425E-07	7,07637E-06	1,9697E-07	1,9894E-06	[6]	Group089	7,07	81,00
GO:0034728	nucleosome organization	GO_BiologicalProcess-EBI-UniProt-GOA-ACAP-ARAP_08.05.2020_00h00	3,468E-09	1,64113E-07	1,9697E-07	1,9894E-06	[5, 6, 7]	Group089	14,43	28,00
GO:0071103	DNA conformation change	GO_BiologicalProcess-EBI-UniProt-GOA-ACAP-ARAP_08.05.2020_00h00	6,4405E-08	1,88965E-06	1,9697E-07	1,9894E-06	[6]	Group089	10,26	39,00
GO:0065004	protein-DNA complex assembly	GO_BiologicalProcess-EBI-UniProt-GOA-ACAP-ARAP_08.05.2020_00h00	2,7077E-10	2,8373E-08	1,9697E-07	1,9894E-06	[6, 7]	Group089	13,46	35,00
GO:0006334	nucleosome assembly	GO_BiologicalProcess-EBI-UniProt-GOA-ACAP-ARAP_08.05.2020_00h00	7,4995E-10	6,47167E-08	1,9697E-07	1,9894E-06	[6, 7, 8, 9]	Group089	17,01	25,00
GO:0050772	positive regulation of axonogenesis	GO_BiologicalProcess-EBI-UniProt-GOA-ACAP-ARAP_08.05.2020_00h00	0,0013666	0,010551599	3,4796E-07	2,9287E-06	[6, 7, 8, 9, 10, 11, 12, 13, 14]	Group093	11,58	11,00
GO:0051094	positive regulation of developmental process	GO_BiologicalProcess-EBI-UniProt-GOA-ACAP-ARAP_08.05.2020_00h00	0,00128977	0,010118128	3,4796E-07	2,9287E-06	[2, 3, 4]	Group093	5,55	82,00

GO:0051240	positive regulation of multicellular organismal process	GO_BiologicalProcess-EBI-UniProt-GOA-ACAP-ARAP_08.05.2020_00h00	0,00121394	0,009731382	3,4796E-07	2,9287E-06	[2, 3, 4]	Group093	5,34	102,00
GO:0022603	regulation of anatomical structure morphogenesis	GO_BiologicalProcess-EBI-UniProt-GOA-ACAP-ARAP_08.05.2020_00h00	0,00284967	0,017713821	3,4796E-07	2,9287E-06	[3, 4]	Group093	5,62	66,00
GO:0045595	regulation of cell differentiation	GO_BiologicalProcess-EBI-UniProt-GOA-ACAP-ARAP_08.05.2020_00h00	1,4649E-06	2,90403E-05	3,4796E-07	2,9287E-06	[3, 4]	Group093	6,06	120,00
GO:0051128	regulation of cellular component organization	GO_BiologicalProcess-EBI-UniProt-GOA-ACAP-ARAP_08.05.2020_00h00	0,00053505	0,005566799	3,4796E-07	2,9287E-06	[3, 4]	Group093	5,21	133,00
GO:0060560	developmental growth involved in morphogenesis	GO_BiologicalProcess-EBI-UniProt-GOA-ACAP-ARAP_08.05.2020_00h00	0,00172929	0,012748064	3,4796E-07	2,9287E-06	[3, 4]	Group093	8,08	21,00
GO:0045597	positive regulation of cell differentiation	GO_BiologicalProcess-EBI-UniProt-GOA-ACAP-ARAP_08.05.2020_00h00	0,00057936	0,005943533	3,4796E-07	2,9287E-06	[3, 4, 5]	Group093	6,03	64,00
GO:0051130	positive regulation of cellular component organization	GO_BiologicalProcess-EBI-UniProt-GOA-ACAP-ARAP_08.05.2020_00h00	0,00397208	0,021988861	3,4796E-07	2,9287E-06	[3, 4, 5]	Group093	5,46	71,00
GO:0033043	regulation of organelle organization	GO_BiologicalProcess-EBI-UniProt-GOA-ACAP-ARAP_08.05.2020_00h00	0,00119093	0,009706078	3,4796E-07	2,9287E-06	[4, 5]	Group093	5,62	78,00
GO:0010638	positive regulation of organelle organization	GO_BiologicalProcess-EBI-UniProt-GOA-ACAP-ARAP_08.05.2020_00h00	0,00249017	0,016381536	3,4796E-07	2,9287E-06	[4, 5, 6]	Group093	6,23	43,00
GO:0010720	positive regulation of cell development	GO_BiologicalProcess-EBI-UniProt-GOA-ACAP-ARAP_08.05.2020_00h00	0,01184334	0,044209119	3,4796E-07	2,9287E-06	[4, 5, 6]	Group093	5,91	36,00
GO:0010770	positive regulation of cell morphogenesis involved in differentiation	GO_BiologicalProcess-EBI-UniProt-GOA-ACAP-ARAP_08.05.2020_00h00	0,00329477	0,019333733	3,4796E-07	2,9287E-06	[5, 6, 7]	Group093	8,82	15,00
GO:0050769	positive regulation of neurogenesis	GO_BiologicalProcess-EBI-UniProt-GOA-ACAP-ARAP_08.05.2020_00h00	0,0070058	0,03172072	3,4796E-07	2,9287E-06	[4, 5, 6, 7, 8, 9]	Group093	6,26	33,00
GO:0006259	DNA metabolic process	GO_BiologicalProcess-EBI-UniProt-GOA-ACAP-ARAP_08.05.2020_00h00	4,5874E-07	1,01965E-05	4,2095E-07	3,2705E-06	[4, 5, 6]	Group054	7,26	73,00
GO:2001020	regulation of response to DNA damage stimulus	GO_BiologicalProcess-EBI-UniProt-GOA-ACAP-ARAP_08.05.2020_00h00	0,0073145	0,032615103	4,2095E-07	3,2705E-06	[4, 5, 6]	Group054	7,53	18,00
GO:0051052	regulation of DNA metabolic process	GO_BiologicalProcess-EBI-UniProt-GOA-ACAP-ARAP_08.05.2020_00h00	0,00325071	0,019306853	4,2095E-07	3,2705E-06	[4, 5, 6, 7]	Group054	7,01	27,00
GO:0000726	non-recombinational repair	GO_BiologicalProcess-EBI-UniProt-GOA-ACAP-ARAP_08.05.2020_00h00	3,3384E-05	0,000484896	4,2095E-07	3,2705E-06	[6, 7, 8]	Group054	13,51	15,00
GO:0002520	immune system development	GO_BiologicalProcess-EBI-UniProt-GOA-ACAP-ARAP_08.05.2020_00h00	2,2993E-06	4,32451E-05	2,2993E-06	1,6588E-05	[2, 4, 5]	Group071	6,92	74,00
GO:0048534	hematopoietic or lymphoid organ development	GO_BiologicalProcess-EBI-UniProt-GOA-ACAP-ARAP_08.05.2020_00h00	8,8905E-07	1,89019E-05	2,2993E-06	1,6588E-05	[3, 4, 5, 6]	Group071	7,16	72,00
GO:0030097	hemopoiesis	GO_BiologicalProcess-EBI-UniProt-GOA-ACAP-ARAP_08.05.2020_00h00	1,4609E-06	2,9358E-05	2,2993E-06	1,6588E-05	[4, 5, 6, 7]	Group071	7,17	69,00
GO:0002521	leukocyte differentiation	GO_BiologicalProcess-EBI-UniProt-GOA-ACAP-ARAP_08.05.2020_00h00	0,00172079	0,012749455	2,2993E-06	1,6588E-05	[4, 5, 6, 7, 8]	Group071	6,63	37,00
GO:0030098	lymphocyte differentiation	GO_BiologicalProcess-EBI-UniProt-GOA-ACAP-ARAP_08.05.2020_00h00	0,00987359	0,039683726	2,2993E-06	1,6588E-05	[4, 5, 6, 7, 8, 9]	Group071	6,58	25,00
GO:0033077	T cell differentiation in thymus	GO_BiologicalProcess-EBI-UniProt-GOA-ACAP-ARAP_08.05.2020_00h00	0,00406016	0,022224842	2,2993E-06	1,6588E-05	[6, 7, 8, 9, 10, 11]	Group071	11,39	9,00
GO:0051145	smooth muscle cell differentiation	GO_BiologicalProcess-EBI-UniProt-GOA-ACAP-ARAP_08.05.2020_00h00	0,01034567	0,040908623	2,796E-06	1,8826E-05	[5]	Group092	11,48	7,00

GO:2000648	positive regulation of stem cell proliferation	GO_BiologicalProcess-EBI-UniProt-GOA-ACAP-ARAP_08.05.2020_00h00	0,00936475	0,03869886	2,796E-06	1,8826E-05	[4, 5, 6]	Group092	13,04	6,00
GO:0045446	endothelial cell differentiation	GO_BiologicalProcess-EBI-UniProt-GOA-ACAP-ARAP_08.05.2020_00h00	0,0102085	0,040806182	2,796E-06	1,8826E-05	[5, 6]	Group092	9,35	10,00
GO:2000047	regulation of cell-cell adhesion mediated by cadherin	GO_BiologicalProcess-EBI-UniProt-GOA-ACAP-ARAP_08.05.2020_00h00	0,00146997	0,011115676	2,796E-06	1,8826E-05	[5, 6]	Group092	22,73	5,00
GO:2000048	negative regulation of cell-cell adhesion mediated by cadherin	GO_BiologicalProcess-EBI-UniProt-GOA-ACAP-ARAP_08.05.2020_00h00	0,00609474	0,029314715	2,796E-06	1,8826E-05	[5, 6, 7]	Group092	30,00	3,00
GO:0010718	positive regulation of epithelial to mesenchymal transition	GO_BiologicalProcess-EBI-UniProt-GOA-ACAP-ARAP_08.05.2020_00h00	0,01147846	0,043511376	2,796E-06	1,8826E-05	[3, 4, 5, 6, 7, 8, 9, 10]	Group092	12,50	6,00
GO:0035886	vascular smooth muscle cell differentiation	GO_BiologicalProcess-EBI-UniProt-GOA-ACAP-ARAP_08.05.2020_00h00	0,00450535	0,023437398	2,796E-06	1,8826E-05	[5, 6, 7, 8]	Group092	17,86	5,00
GO:0060528	secretory columnal luminar epithelial cell differentiation involved in prostate glandular acinus development	GO_BiologicalProcess-EBI-UniProt-GOA-ACAP-ARAP_08.05.2020_00h00	0,00024314	0,002743778	2,796E-06	1,8826E-05	[4, 5, 6, 7, 8, 9]	Group092	75,00	3,00
GO:0060976	coronary vasculature development	GO_BiologicalProcess-EBI-UniProt-GOA-ACAP-ARAP_08.05.2020_00h00	0,00344449	0,020051828	2,796E-06	1,8826E-05	[4, 5, 6, 7, 8, 9]	Group092	14,00	7,00
GO:0001570	vasculogenesis	GO_BiologicalProcess-EBI-UniProt-GOA-ACAP-ARAP_08.05.2020_00h00	0,00884597	0,037290338	2,796E-06	1,8826E-05	[4, 5, 6, 7, 8, 9, 10]	Group092	10,11	9,00
GO:0048845	venous blood vessel morphogenesis	GO_BiologicalProcess-EBI-UniProt-GOA-ACAP-ARAP_08.05.2020_00h00	0,00439531	0,02319398	2,796E-06	1,8826E-05	[5, 6, 7, 8, 9, 10]	Group092	33,33	3,00
GO:0060947	cardiac vascular smooth muscle cell differentiation	GO_BiologicalProcess-EBI-UniProt-GOA-ACAP-ARAP_08.05.2020_00h00	0,003019	0,018608693	2,796E-06	1,8826E-05	[5, 6, 7, 8, 9, 10]	Group092	37,50	3,00
GO:0035909	aorta morphogenesis	GO_BiologicalProcess-EBI-UniProt-GOA-ACAP-ARAP_08.05.2020_00h00	0,00810163	0,03516297	2,796E-06	1,8826E-05	[6, 7, 8, 9, 10, 11]	Group092	15,63	5,00
GO:0019899	enzyme binding	GO_MolecularFunction-EBI-UniProt-GOA-ACAP-ARAP_08.05.2020_00h00	3,3592E-06	5,86663E-05	3,3592E-06	1,9958E-05	[3]	Group051	5,77	137,00
GO:0019900	kinase binding	GO_MolecularFunction-EBI-UniProt-GOA-ACAP-ARAP_08.05.2020_00h00	0,00325059	0,019384639	3,3592E-06	1,9958E-05	[4]	Group051	6,01	47,00
GO:0019901	protein kinase binding	GO_MolecularFunction-EBI-UniProt-GOA-ACAP-ARAP_08.05.2020_00h00	0,01073362	0,041546736	3,3592E-06	1,9958E-05	[5]	Group051	5,81	40,00
GO:0045815	positive regulation of gene expression, epigenetic	GO_BiologicalProcess-EBI-UniProt-GOA-ACAP-ARAP_08.05.2020_00h00	4,6028E-05	0,000643077	3,2749E-06	2,0673E-05	[5, 6, 7]	Group053	16,92	11,00
GO:0031935	regulation of chromatin silencing	GO_BiologicalProcess-EBI-UniProt-GOA-ACAP-ARAP_08.05.2020_00h00	9,3095E-05	0,001167273	3,2749E-06	2,0673E-05	[4, 5, 6, 7, 8, 9, 10, 11, 12, 13, 14]	Group053	30,00	6,00
GO:0048096	chromatin-mediated maintenance of transcription	GO_BiologicalProcess-EBI-UniProt-GOA-ACAP-ARAP_08.05.2020_00h00	0,00180454	0,013236315	3,2749E-06	2,0673E-05	[6, 7, 8, 9, 10, 11, 12]	Group053	28,57	4,00
GO:0030374	nuclear receptor transcription coactivator activity	GO_BiologicalProcess-EBI-UniProt-GOA-ACAP-ARAP_08.05.2020_00h00	7,2046E-05	0,000935317	4,6302E-06	2,598E-05	[8, 9, 10, 11, 12]	Group084	15,00	12,00
GO:0008134	transcription factor binding	GO_MolecularFunction-EBI-UniProt-GOA-ACAP-ARAP_08.05.2020_00h00	1,4931E-05	0,00023302	4,6302E-06	2,598E-05	[3]	Group084	7,31	53,00
GO:0045935	positive regulation of nucleobase-containing compound metabolic process	GO_BiologicalProcess-EBI-UniProt-GOA-ACAP-ARAP_08.05.2020_00h00	0,00017084	0,002004963	4,6302E-06	2,598E-05	[4, 5, 6]	Group084	5,53	113,00
GO:0051254	positive regulation of RNA metabolic process	GO_BiologicalProcess-EBI-UniProt-GOA-ACAP-ARAP_08.05.2020_00h00	0,0002428	0,002761146	4,6302E-06	2,598E-05	[4, 5, 6, 7, 8]	Group084	5,57	104,00
GO:1903508	positive regulation of nucleic acid-templated transcription	GO_BiologicalProcess-EBI-UniProt-GOA-ACAP-ARAP_08.05.2020_00h00	9,8332E-05	0,001222489	4,6302E-06	2,598E-05	[6, 7, 8, 9, 10]	Group084	5,73	102,00

GO:0003712	transcription coregulator activity	GO_BiologicalProcess-EBI-UniProt-GOA-ACAP-ARAP_08.05.2020_00h00	5,4984E-08	1,68046E-06	4,6302E-06	2,598E-05	[7, 8, 9, 10]	Group084	8,65	55,00
GO:0003713	transcription coactivator activity	GO_BiologicalProcess-EBI-UniProt-GOA-ACAP-ARAP_08.05.2020_00h00	1,168E-05	0,000188293	4,6302E-06	2,598E-05	[7, 8, 9, 10, 11]	Group084	8,99	33,00
GO:0003714	transcription corepressor activity	GO_BiologicalProcess-EBI-UniProt-GOA-ACAP-ARAP_08.05.2020_00h00	0,00295276	0,018277241	4,6302E-06	2,598E-05	[7, 8, 9, 10, 11]	Group084	7,87	20,00
GO:0006367	transcription initiation from RNA polymerase II promoter	GO_BiologicalProcess-EBI-UniProt-GOA-ACAP-ARAP_08.05.2020_00h00	0,0006665	0,006562146	4,6302E-06	2,598E-05	[7, 8, 9, 10, 11]	Group084	9,13	19,00
GO:0043966	histone H3 acetylation	GO_BiologicalProcess-EBI-UniProt-GOA-ACAP-ARAP_08.05.2020_00h00	0,01034567	0,040908623	7,0972E-06	3,7727E-05	[8, 9, 10, 11, 12]	Group075	11,48	7,00
GO:0043543	protein acylation	GO_BiologicalProcess-EBI-UniProt-GOA-ACAP-ARAP_08.05.2020_00h00	0,00368337	0,02110742	7,0972E-06	3,7727E-05	[6, 7]	Group075	7,72	20,00
GO:0016570	histone modification	GO_BiologicalProcess-EBI-UniProt-GOA-ACAP-ARAP_08.05.2020_00h00	0,00012397	0,00150305	7,0972E-06	3,7727E-05	[6, 7, 8]	Group075	7,63	37,00
GO:0018205	peptidyl-lysine modification	GO_BiologicalProcess-EBI-UniProt-GOA-ACAP-ARAP_08.05.2020_00h00	2,6372E-06	4,83594E-05	7,0972E-06	3,7727E-05	[7, 8]	Group075	8,98	38,00
GO:0006475	internal protein amino acid acetylation	GO_BiologicalProcess-EBI-UniProt-GOA-ACAP-ARAP_08.05.2020_00h00	0,00191473	0,01390548	7,0972E-06	3,7727E-05	[8, 9]	Group075	9,04	16,00
GO:0016573	histone acetylation	GO_BiologicalProcess-EBI-UniProt-GOA-ACAP-ARAP_08.05.2020_00h00	0,00262382	0,01703164	7,0972E-06	3,7727E-05	[7, 8, 9, 10, 11]	Group075	9,04	15,00
GO:0008134	transcription factor binding	GO_MolecularFunction-EBI-UniProt-GOA-ACAP-ARAP_08.05.2020_00h00	1,4931E-05	0,00023302	1,314E-05	6,6356E-05	[3]	Group098	7,31	53,00
GO:0030522	intracellular receptor signaling pathway	GO_BiologicalProcess-EBI-UniProt-GOA-ACAP-ARAP_08.05.2020_00h00	0,00076096	0,007296289	1,314E-05	6,6356E-05	[3, 4, 5]	Group098	8,14	24,00
GO:0051427	hormone receptor binding	GO_MolecularFunction-EBI-UniProt-GOA-ACAP-ARAP_08.05.2020_00h00	0,00578301	0,028661067	1,314E-05	6,6356E-05	[4]	Group098	8,08	16,00
GO:0140297	DNA-binding transcription factor binding	GO_MolecularFunction-EBI-UniProt-GOA-ACAP-ARAP_08.05.2020_00h00	0,00583479	0,028820314	1,314E-05	6,6356E-05	[4]	Group098	6,79	26,00
GO:0009755	hormone-mediated signaling pathway	GO_BiologicalProcess-EBI-UniProt-GOA-ACAP-ARAP_08.05.2020_00h00	0,01050162	0,041192181	1,314E-05	6,6356E-05	[3, 4, 5, 6]	Group098	7,26	18,00
GO:0032870	cellular response to hormone stimulus	GO_BiologicalProcess-EBI-UniProt-GOA-ACAP-ARAP_08.05.2020_00h00	0,00399949	0,021974724	1,314E-05	6,6356E-05	[4, 5]	Group098	5,97	46,00
GO:0048384	retinoic acid receptor signaling pathway	GO_BiologicalProcess-EBI-UniProt-GOA-ACAP-ARAP_08.05.2020_00h00	0,00021557	0,002490113	1,314E-05	6,6356E-05	[4, 5, 6]	Group098	21,88	7,00
GO:0061629	RNA polymerase II-specific DNA-binding transcription factor binding	GO_MolecularFunction-EBI-UniProt-GOA-ACAP-ARAP_08.05.2020_00h00	0,00928244	0,038576037	1,314E-05	6,6356E-05	[5]	Group098	6,98	21,00
GO:0071383	cellular response to steroid hormone stimulus	GO_BiologicalProcess-EBI-UniProt-GOA-ACAP-ARAP_08.05.2020_00h00	0,00606695	0,029373631	1,314E-05	6,6356E-05	[5, 6]	Group098	7,38	20,00
GO:0030518	intracellular steroid hormone receptor signaling pathway	GO_BiologicalProcess-EBI-UniProt-GOA-ACAP-ARAP_08.05.2020_00h00	0,00366435	0,021080799	1,314E-05	6,6356E-05	[4, 5, 6, 7, 8]	Group098	9,35	13,00
GO:0016922	nuclear receptor binding	GO_MolecularFunction-EBI-UniProt-GOA-ACAP-ARAP_08.05.2020_00h00	0,00651478	0,030730493	1,314E-05	6,6356E-05	[6, 7]	Group098	9,48	11,00
GO:0033143	regulation of intracellular steroid hormone receptor signaling pathway	GO_BiologicalProcess-EBI-UniProt-GOA-ACAP-ARAP_08.05.2020_00h00	0,00136584	0,01060153	1,314E-05	6,6356E-05	[4, 5, 6, 7, 8, 9]	Group098	12,35	10,00
GO:0030521	androgen receptor signaling pathway	GO_BiologicalProcess-EBI-UniProt-GOA-ACAP-ARAP_08.05.2020_00h00	0,01330857	0,047387552	1,314E-05	6,6356E-05	[5, 6, 7, 8, 9]	Group098	10,94	7,00
GO:0050847	progesterone receptor signaling pathway	GO_BiologicalProcess-EBI-UniProt-GOA-ACAP-ARAP_08.05.2020_00h00	0,003019	0,018608693	1,314E-05	6,6356E-05	[5, 6, 7, 8, 9]	Group098	37,50	3,00

GO:0033146	regulation of intracellular estrogen receptor signaling pathway	GO_BiologicalProcess-EBI-UniProt-GOA-ACAP-ARAP_08.05.2020_00h00	0,00841935	0,036114566	1,314E-05	6,6356E-05	[5, 6, 7, 8, 9, 10]	Group098	13,33	6,00
GO:0033148	positive regulation of intracellular estrogen receptor signaling pathway	GO_BiologicalProcess-EBI-UniProt-GOA-ACAP-ARAP_08.05.2020_00h00	0,00307946	0,018514641	1,314E-05	6,6356E-05	[5, 6, 7, 8, 9, 10, 11]	Group098	25,00	4,00
GO:0050772	positive regulation of axonogenesis	GO_BiologicalProcess-EBI-UniProt-GOA-ACAP-ARAP_08.05.2020_00h00	0,0013666	0,010551599	1,8581E-05	8,9364E-05	[6, 7, 8, 9, 10, 11, 12, 13, 14]	Group086	11,58	11,00
GO:0060560	developmental growth involved in morphogenesis	GO_BiologicalProcess-EBI-UniProt-GOA-ACAP-ARAP_08.05.2020_00h00	0,00172929	0,012748064	1,8581E-05	8,9364E-05	[3, 4]	Group086	8,08	21,00
GO:0001558	regulation of cell growth	GO_BiologicalProcess-EBI-UniProt-GOA-ACAP-ARAP_08.05.2020_00h00	0,00221933	0,015357364	1,8581E-05	8,9364E-05	[3, 4, 5]	Group086	6,89	31,00
GO:0032535	regulation of cellular component size	GO_BiologicalProcess-EBI-UniProt-GOA-ACAP-ARAP_08.05.2020_00h00	0,00677551	0,031158835	1,8581E-05	8,9364E-05	[4]	Group086	6,63	27,00
GO:0061138	morphogenesis of a branching epithelium	GO_BiologicalProcess-EBI-UniProt-GOA-ACAP-ARAP_08.05.2020_00h00	0,00284655	0,017769769	1,8581E-05	8,9364E-05	[3, 4, 5, 6]	Group086	8,46	17,00
GO:0010720	positive regulation of cell development	GO_BiologicalProcess-EBI-UniProt-GOA-ACAP-ARAP_08.05.2020_00h00	0,01184334	0,044209119	1,8581E-05	8,9364E-05	[4, 5, 6]	Group086	5,91	36,00
GO:0060562	epithelial tube morphogenesis	GO_BiologicalProcess-EBI-UniProt-GOA-ACAP-ARAP_08.05.2020_00h00	0,00102574	0,009010539	1,8581E-05	8,9364E-05	[4, 5, 6]	Group086	7,61	27,00
GO:0048754	branching morphogenesis of an epithelial tube	GO_BiologicalProcess-EBI-UniProt-GOA-ACAP-ARAP_08.05.2020_00h00	0,00247505	0,016355416	1,8581E-05	8,9364E-05	[4, 5, 6, 7]	Group086	9,09	15,00
GO:0010770	positive regulation of cell morphogenesis involved in differentiation	GO_BiologicalProcess-EBI-UniProt-GOA-ACAP-ARAP_08.05.2020_00h00	0,00329477	0,019333733	1,8581E-05	8,9364E-05	[5, 6, 7]	Group086	8,82	15,00
GO:0050769	positive regulation of neurogenesis	GO_BiologicalProcess-EBI-UniProt-GOA-ACAP-ARAP_08.05.2020_00h00	0,0070058	0,03172072	1,8581E-05	8,9364E-05	[4, 5, 6, 7, 8, 9]	Group086	6,26	33,00
GO:0030374	nuclear receptor transcription coactivator activity	GO_BiologicalProcess-EBI-UniProt-GOA-ACAP-ARAP_08.05.2020_00h00	7,2046E-05	0,000935317	2,8517E-05	0,00013092	[8, 9, 10, 11, 12]	Group063	15,00	12,00
GO:0071391	cellular response to estrogen stimulus	GO_BiologicalProcess-EBI-UniProt-GOA-ACAP-ARAP_08.05.2020_00h00	0,00092442	0,008475746	2,8517E-05	0,00013092	[3, 4]	Group063	25,00	5,00
GO:0045445	myoblast differentiation	GO_BiologicalProcess-EBI-UniProt-GOA-ACAP-ARAP_08.05.2020_00h00	0,00884597	0,037290338	2,8517E-05	0,00013092	[4]	Group063	10,11	9,00
GO:0002153	steroid receptor RNA activator RNA binding	GO_MolecularFunction-EBI-UniProt-GOA-ACAP-ARAP_08.05.2020_00h00	3,4908E-05	0,000502063	2,8517E-05	0,00013092	[6]	Group063	66,67	4,00
GO:0003713	transcription coactivator activity	GO_BiologicalProcess-EBI-UniProt-GOA-ACAP-ARAP_08.05.2020_00h00	1,168E-05	0,000188293	2,8517E-05	0,00013092	[7, 8, 9, 10, 11]	Group063	8,99	33,00
GO:0033043	regulation of organelle organization	GO_BiologicalProcess-EBI-UniProt-GOA-ACAP-ARAP_08.05.2020_00h00	0,00119093	0,009706078	5,7509E-05	0,00025254	[4, 5]	Group067	5,62	78,00
GO:0010638	positive regulation of organelle organization	GO_BiologicalProcess-EBI-UniProt-GOA-ACAP-ARAP_08.05.2020_00h00	0,00249017	0,016381536	5,7509E-05	0,00025254	[4, 5, 6]	Group067	6,23	43,00
GO:0033044	regulation of chromosome organization	GO_BiologicalProcess-EBI-UniProt-GOA-ACAP-ARAP_08.05.2020_00h00	0,0056442	0,028067946	5,7509E-05	0,00025254	[5, 6]	Group067	6,81	26,00
GO:1902275	regulation of chromatin organization	GO_BiologicalProcess-EBI-UniProt-GOA-ACAP-ARAP_08.05.2020_00h00	0,00835802	0,035956629	5,7509E-05	0,00025254	[5, 6, 7]	Group067	7,77	16,00
GO:0016570	histone modification	GO_BiologicalProcess-EBI-UniProt-GOA-ACAP-ARAP_08.05.2020_00h00	0,00012397	0,00150305	5,7509E-05	0,00025254	[6, 7, 8]	Group067	7,63	37,00
GO:0019827	stem cell population maintenance	GO_BiologicalProcess-EBI-UniProt-GOA-ACAP-ARAP_08.05.2020_00h00	0,00086558	0,00808796	6,0089E-05	0,00025287	[2, 3]	Group101	9,44	17,00

GO:0035019	somatic stem cell population maintenance	GO_BiologicalProcess-EBI-UniProt-GOA-ACAP-ARAP_08.05.2020_00h00	0,00150131	0,011236882	6,0089E-05	0,00025287	[3, 4]	Group101	12,20	10,00
GO:0048729	tissue morphogenesis	GO_BiologicalProcess-EBI-UniProt-GOA-ACAP-ARAP_08.05.2020_00h00	0,01362663	0,048285682	6,0089E-05	0,00025287	[3, 4]	Group101	5,69	41,00
GO:0048103	somatic stem cell division	GO_BiologicalProcess-EBI-UniProt-GOA-ACAP-ARAP_08.05.2020_00h00	0,01052948	0,041191343	6,0089E-05	0,00025287	[4]	Group101	25,00	3,00
GO:0001655	urogenital system development	GO_BiologicalProcess-EBI-UniProt-GOA-ACAP-ARAP_08.05.2020_00h00	0,01173816	0,044267047	6,0089E-05	0,00025287	[4, 5]	Group101	6,56	24,00
GO:0060541	respiratory system development	GO_BiologicalProcess-EBI-UniProt-GOA-ACAP-ARAP_08.05.2020_00h00	0,00734388	0,032548264	6,0089E-05	0,00025287	[4, 5]	Group101	7,69	17,00
GO:0061138	morphogenesis of a branching epithelium	GO_BiologicalProcess-EBI-UniProt-GOA-ACAP-ARAP_08.05.2020_00h00	0,00284655	0,017769769	6,0089E-05	0,00025287	[3, 4, 5, 6]	Group101	8,46	17,00
GO:2000736	regulation of stem cell differentiation	GO_BiologicalProcess-EBI-UniProt-GOA-ACAP-ARAP_08.05.2020_00h00	0,01182202	0,044355243	6,0089E-05	0,00025287	[4, 5]	Group101	8,73	11,00
GO:0030856	regulation of epithelial cell differentiation	GO_BiologicalProcess-EBI-UniProt-GOA-ACAP-ARAP_08.05.2020_00h00	0,00151781	0,011302683	6,0089E-05	0,00025287	[4, 5, 6]	Group101	9,55	15,00
GO:0048732	gland development	GO_BiologicalProcess-EBI-UniProt-GOA-ACAP-ARAP_08.05.2020_00h00	0,00223689	0,015262903	6,0089E-05	0,00025287	[4, 5, 6]	Group101	6,69	34,00
GO:0051145	smooth muscle cell differentiation	GO_BiologicalProcess-EBI-UniProt-GOA-ACAP-ARAP_08.05.2020_00h00	0,01034567	0,040908623	6,0089E-05	0,00025287	[5]	Group101	11,48	7,00
GO:0060485	mesenchyme development	GO_BiologicalProcess-EBI-UniProt-GOA-ACAP-ARAP_08.05.2020_00h00	0,00693329	0,031685803	6,0089E-05	0,00025287	[4, 5, 6]	Group101	7,17	21,00
GO:0060562	epithelial tube morphogenesis	GO_BiologicalProcess-EBI-UniProt-GOA-ACAP-ARAP_08.05.2020_00h00	0,00102574	0,009010539	6,0089E-05	0,00025287	[4, 5, 6]	Group101	7,61	27,00
GO:0061009	common bile duct development	GO_BiologicalProcess-EBI-UniProt-GOA-ACAP-ARAP_08.05.2020_00h00	3,4908E-05	0,000502063	6,0089E-05	0,00025287	[4, 5, 6]	Group101	66,67	4,00
GO:2000737	negative regulation of stem cell differentiation	GO_BiologicalProcess-EBI-UniProt-GOA-ACAP-ARAP_08.05.2020_00h00	0,00222151	0,015300271	6,0089E-05	0,00025287	[4, 5, 6]	Group101	20,83	5,00
GO:0003279	cardiac septum development	GO_BiologicalProcess-EBI-UniProt-GOA-ACAP-ARAP_08.05.2020_00h00	0,00886469	0,03726219	6,0089E-05	0,00025287	[3, 4, 6, 7, 8]	Group101	9,09	11,00
GO:0001822	kidney development	GO_BiologicalProcess-EBI-UniProt-GOA-ACAP-ARAP_08.05.2020_00h00	0,01183102	0,044275783	6,0089E-05	0,00025287	[4, 5, 6, 7]	Group101	6,82	21,00
GO:0030857	negative regulation of epithelial cell differentiation	GO_BiologicalProcess-EBI-UniProt-GOA-ACAP-ARAP_08.05.2020_00h00	2,2595E-05	0,000338235	6,0089E-05	0,00025287	[4, 5, 6, 7]	Group101	20,00	10,00
GO:0048754	branching morphogenesis of an epithelial tube	GO_BiologicalProcess-EBI-UniProt-GOA-ACAP-ARAP_08.05.2020_00h00	0,00247505	0,016355416	6,0089E-05	0,00025287	[4, 5, 6, 7]	Group101	9,09	15,00
GO:0061323	cell proliferation involved in heart morphogenesis	GO_BiologicalProcess-EBI-UniProt-GOA-ACAP-ARAP_08.05.2020_00h00	0,00092442	0,008475746	6,0089E-05	0,00025287	[3, 5, 6, 7, 8]	Group101	25,00	5,00
GO:0021783	preganglionic parasympathetic fiber development	GO_BiologicalProcess-EBI-UniProt-GOA-ACAP-ARAP_08.05.2020_00h00	0,00722575	0,032317616	6,0089E-05	0,00025287	[4, 5, 6, 7, 8]	Group101	20,00	4,00
GO:0060674	placenta blood vessel development	GO_BiologicalProcess-EBI-UniProt-GOA-ACAP-ARAP_08.05.2020_00h00	0,00412203	0,022396339	6,0089E-05	0,00025287	[3, 4, 5, 6, 7, 8, 9]	Group101	15,38	6,00
GO:0061005	cell differentiation involved in kidney development	GO_BiologicalProcess-EBI-UniProt-GOA-ACAP-ARAP_08.05.2020_00h00	0,01226081	0,04496653	6,0089E-05	0,00025287	[4, 5, 6, 7, 8]	Group101	11,11	7,00
GO:0090183	regulation of kidney development	GO_BiologicalProcess-EBI-UniProt-GOA-ACAP-ARAP_08.05.2020_00h00	0,00865794	0,036708646	6,0089E-05	0,00025287	[4, 5, 6, 7, 8]	Group101	11,86	7,00
GO:2000048	negative regulation of cell-cell adhesion mediated by cadherin	GO_BiologicalProcess-EBI-UniProt-GOA-ACAP-ARAP_08.05.2020_00h00	0,00609474	0,029314715	6,0089E-05	0,00025287	[5, 6, 7]	Group101	30,00	3,00

GO:0035886	vascular smooth muscle cell differentiation	GO_BiologicalProcess-EBI-UniProt-GOA-ACAP-ARAP_08.05.2020_00h00	0,00450535	0,023437398	6,0089E-05	0,00025287	[5, 6, 7, 8]	Group101	17,86	5,00
GO:0060976	coronary vasculature development	GO_BiologicalProcess-EBI-UniProt-GOA-ACAP-ARAP_08.05.2020_00h00	0,00344449	0,020051828	6,0089E-05	0,00025287	[4, 5, 6, 7, 8, 9]	Group101	14,00	7,00
GO:0072049	comma-shaped body morphogenesis	GO_BiologicalProcess-EBI-UniProt-GOA-ACAP-ARAP_08.05.2020_00h00	0,00194417	0,014049736	6,0089E-05	0,00025287	[3, 4, 5, 6, 7, 8, 9, 10]	Group101	42,86	3,00
GO:0072050	S-shaped body morphogenesis	GO_BiologicalProcess-EBI-UniProt-GOA-ACAP-ARAP_08.05.2020_00h00	0,00609474	0,029314715	6,0089E-05	0,00025287	[3, 4, 5, 6, 7, 8, 9, 10]	Group101	30,00	3,00
GO:0072073	kidney epithelium development	GO_BiologicalProcess-EBI-UniProt-GOA-ACAP-ARAP_08.05.2020_00h00	0,01053228	0,041092683	6,0089E-05	0,00025287	[5, 6, 7, 8]	Group101	8,23	13,00
GO:2000826	regulation of heart morphogenesis	GO_BiologicalProcess-EBI-UniProt-GOA-ACAP-ARAP_08.05.2020_00h00	0,00754438	0,033136562	6,0089E-05	0,00025287	[5, 6, 7, 8]	Group101	13,64	6,00
GO:0072012	glomerulus vasculature development	GO_BiologicalProcess-EBI-UniProt-GOA-ACAP-ARAP_08.05.2020_00h00	0,00382396	0,021575958	6,0089E-05	0,00025287	[4, 5, 6, 7, 8, 9, 10]	Group101	18,52	5,00
GO:0060251	regulation of glial cell proliferation	GO_BiologicalProcess-EBI-UniProt-GOA-ACAP-ARAP_08.05.2020_00h00	0,01048958	0,041366164	6,0089E-05	0,00025287	[4, 5, 7, 8, 9, 10]	Group101	14,71	5,00
GO:0014014	negative regulation of gliogenesis	GO_BiologicalProcess-EBI-UniProt-GOA-ACAP-ARAP_08.05.2020_00h00	0,01147846	0,043511376	6,0089E-05	0,00025287	[5, 6, 7, 8, 9, 10]	Group101	12,50	6,00
GO:0048845	venous blood vessel morphogenesis	GO_BiologicalProcess-EBI-UniProt-GOA-ACAP-ARAP_08.05.2020_00h00	0,00439531	0,02319398	6,0089E-05	0,00025287	[5, 6, 7, 8, 9, 10]	Group101	33,33	3,00
GO:0060947	cardiac vascular smooth muscle cell differentiation	GO_BiologicalProcess-EBI-UniProt-GOA-ACAP-ARAP_08.05.2020_00h00	0,003019	0,018608693	6,0089E-05	0,00025287	[5, 6, 7, 8, 9, 10]	Group101	37,50	3,00
GO:0061101	neuroendocrine cell differentiation	GO_BiologicalProcess-EBI-UniProt-GOA-ACAP-ARAP_08.05.2020_00h00	0,00439531	0,02319398	6,0089E-05	0,00025287	[7, 8]	Group101	33,33	3,00
GO:0072080	nephron tubule development	GO_BiologicalProcess-EBI-UniProt-GOA-ACAP-ARAP_08.05.2020_00h00	0,00224538	0,015179563	6,0089E-05	0,00025287	[5, 6, 7, 8, 9, 10]	Group101	10,89	11,00
GO:1902747	negative regulation of lens fiber cell differentiation	GO_BiologicalProcess-EBI-UniProt-GOA-ACAP-ARAP_08.05.2020_00h00	0,00114476	0,009434626	6,0089E-05	0,00025287	[3, 4, 5, 6, 7, 8, 9, 10, 11, 12]	Group101	50,00	3,00
GO:0035904	aorta development	GO_BiologicalProcess-EBI-UniProt-GOA-ACAP-ARAP_08.05.2020_00h00	0,00789435	0,03457018	6,0089E-05	0,00025287	[5, 7, 8, 9, 10]	Group101	12,07	7,00
GO:0002328	pro-B cell differentiation	GO_BiologicalProcess-EBI-UniProt-GOA-ACAP-ARAP_08.05.2020_00h00	6,1558E-05	0,000843971	6,0089E-05	0,00025287	[6, 7, 8, 9, 10]	Group101	41,67	5,00
GO:0001657	ureteric bud development	GO_BiologicalProcess-EBI-UniProt-GOA-ACAP-ARAP_08.05.2020_00h00	0,00437197	0,023237992	6,0089E-05	0,00025287	[5, 6, 8, 9, 10, 11]	Group101	10,00	11,00
GO:0003266	regulation of secondary heart field cardioblast proliferation	GO_BiologicalProcess-EBI-UniProt-GOA-ACAP-ARAP_08.05.2020_00h00	0,00042998	0,004570884	6,0089E-05	0,00025287	[6, 7, 8, 9, 10, 11]	Group101	40,00	4,00
GO:0035909	aorta morphogenesis	GO_BiologicalProcess-EBI-UniProt-GOA-ACAP-ARAP_08.05.2020_00h00	0,00810163	0,03516297	6,0089E-05	0,00025287	[6, 7, 8, 9, 10, 11]	Group101	15,63	5,00
GO:0048710	regulation of astrocyte differentiation	GO_BiologicalProcess-EBI-UniProt-GOA-ACAP-ARAP_08.05.2020_00h00	0,00706214	0,031877402	6,0089E-05	0,00025287	[6, 7, 8, 9, 10, 11]	Group101	16,13	5,00
GO:2000973	regulation of pro-B cell differentiation	GO_BiologicalProcess-EBI-UniProt-GOA-ACAP-ARAP_08.05.2020_00h00	0,00015279	0,001822294	6,0089E-05	0,00025287	[6, 7, 8, 9, 10, 11]	Group101	50,00	4,00
GO:0045608	negative regulation of inner ear auditory receptor cell differentiation	GO_BiologicalProcess-EBI-UniProt-GOA-ACAP-ARAP_08.05.2020_00h00	0,00058983	0,005967495	6,0089E-05	0,00025287	[5, 6, 7, 8, 9, 10, 11, 12, 13]	Group101	60,00	3,00
GO:0060677	ureteric bud elongation	GO_BiologicalProcess-EBI-UniProt-GOA-ACAP-ARAP_08.05.2020_00h00	0,00439531	0,02319398	6,0089E-05	0,00025287	[4, 5, 7, 8, 9, 10, 11, 12, 13, 14]	Group101	33,33	3,00
GO:0060675	ureteric bud morphogenesis	GO_BiologicalProcess-EBI-UniProt-GOA-ACAP-ARAP_08.05.2020_00h00	0,00194483	0,013985601	6,0089E-05	0,00025287	[6, 7, 8, 9, 10, 11, 12, 13]	Group101	12,68	9,00

GO:0033365	protein localization to organelle	GO_BiologicalProcess-EBI-UniProt-GOA-ACAP-ARAP_08.05.2020_00h00	6,7926E-05	0,00089773	6,7926E-05	0,00027442	[5]	Group052	6,44	67,00
GO:0034504	protein localization to nucleus	GO_BiologicalProcess-EBI-UniProt-GOA-ACAP-ARAP_08.05.2020_00h00	0,00091803	0,008470153	6,7926E-05	0,00027442	[6]	Group052	8,03	24,00
GO:1900180	regulation of protein localization to nucleus	GO_BiologicalProcess-EBI-UniProt-GOA-ACAP-ARAP_08.05.2020_00h00	0,00344332	0,020124931	6,7926E-05	0,00027442	[5, 6, 7]	Group052	9,42	13,00
GO:0022402	cell cycle process	GO_BiologicalProcess-EBI-UniProt-GOA-ACAP-ARAP_08.05.2020_00h00	0,00373411	0,021232305	8,4728E-05	0,00032913	[2, 3]	Group100	5,38	78,00
GO:0000278	mitotic cell cycle	GO_BiologicalProcess-EBI-UniProt-GOA-ACAP-ARAP_08.05.2020_00h00	4,3179E-05	0,000609079	8,4728E-05	0,00032913	[3]	Group100	6,43	71,00
GO:0051726	regulation of cell cycle	GO_BiologicalProcess-EBI-UniProt-GOA-ACAP-ARAP_08.05.2020_00h00	0,00115141	0,009436447	8,4728E-05	0,00032913	[3, 4]	Group100	5,69	74,00
GO:1903047	mitotic cell cycle process	GO_BiologicalProcess-EBI-UniProt-GOA-ACAP-ARAP_08.05.2020_00h00	0,00106744	0,008948199	8,4728E-05	0,00032913	[3, 4]	Group100	6,10	55,00
GO:0045786	negative regulation of cell cycle	GO_BiologicalProcess-EBI-UniProt-GOA-ACAP-ARAP_08.05.2020_00h00	0,01142227	0,043636654	8,4728E-05	0,00032913	[3, 4, 5]	Group100	5,86	38,00
GO:0000075	cell cycle checkpoint	GO_BiologicalProcess-EBI-UniProt-GOA-ACAP-ARAP_08.05.2020_00h00	0,01304005	0,046772026	8,4728E-05	0,00032913	[2, 4, 5, 6]	Group100	7,23	17,00
GO:0007346	regulation of mitotic cell cycle	GO_BiologicalProcess-EBI-UniProt-GOA-ACAP-ARAP_08.05.2020_00h00	0,00024562	0,002750539	8,4728E-05	0,00032913	[4, 5]	Group100	6,76	48,00
GO:0044843	cell cycle G1/S phase transition	GO_BiologicalProcess-EBI-UniProt-GOA-ACAP-ARAP_08.05.2020_00h00	0,01147434	0,043721698	8,4728E-05	0,00032913	[4, 5]	Group100	6,94	20,00
GO:0007093	mitotic cell cycle checkpoint	GO_BiologicalProcess-EBI-UniProt-GOA-ACAP-ARAP_08.05.2020_00h00	0,00980666	0,039631858	8,4728E-05	0,00032913	[3, 4, 5, 6, 7]	Group100	8,05	14,00
GO:0045930	negative regulation of mitotic cell cycle	GO_BiologicalProcess-EBI-UniProt-GOA-ACAP-ARAP_08.05.2020_00h00	0,01292284	0,046465218	8,4728E-05	0,00032913	[4, 5, 6]	Group100	6,67	22,00
GO:0045931	positive regulation of mitotic cell cycle	GO_BiologicalProcess-EBI-UniProt-GOA-ACAP-ARAP_08.05.2020_00h00	0,01106744	0,042502429	8,4728E-05	0,00032913	[4, 5, 6]	Group100	8,18	13,00
GO:0031570	DNA integrity checkpoint	GO_BiologicalProcess-EBI-UniProt-GOA-ACAP-ARAP_08.05.2020_00h00	0,00410062	0,022362851	8,4728E-05	0,00032913	[3, 5, 6, 7]	Group100	8,62	15,00
GO:0140014	mitotic nuclear division	GO_BiologicalProcess-EBI-UniProt-GOA-ACAP-ARAP_08.05.2020_00h00	0,00220162	0,015379864	8,4728E-05	0,00032913	[4, 5, 7]	Group100	7,77	22,00
GO:1901988	negative regulation of cell cycle phase transition	GO_BiologicalProcess-EBI-UniProt-GOA-ACAP-ARAP_08.05.2020_00h00	0,0141845	0,04954442	8,4728E-05	0,00032913	[4, 5, 6, 7]	Group100	7,03	18,00
GO:0000077	DNA damage checkpoint	GO_BiologicalProcess-EBI-UniProt-GOA-ACAP-ARAP_08.05.2020_00h00	0,01280023	0,046251085	8,4728E-05	0,00032913	[4, 5, 6, 7, 8]	Group100	8,02	13,00
GO:0030330	DNA damage response, signal transduction by p53 class mediator	GO_BiologicalProcess-EBI-UniProt-GOA-ACAP-ARAP_08.05.2020_00h00	0,00694048	0,031620145	8,4728E-05	0,00032913	[5, 6, 7]	Group100	9,40	11,00
GO:0006270	DNA replication initiation	GO_BiologicalProcess-EBI-UniProt-GOA-ACAP-ARAP_08.05.2020_00h00	0,00754438	0,033136562	8,4728E-05	0,00032913	[5, 6, 7, 8]	Group100	13,64	6,00
GO:0044773	mitotic DNA damage checkpoint	GO_BiologicalProcess-EBI-UniProt-GOA-ACAP-ARAP_08.05.2020_00h00	0,00262383	0,016956635	8,4728E-05	0,00032913	[5, 6, 7, 8, 9]	Group100	10,68	11,00
GO:2000134	negative regulation of G1/S transition of mitotic cell cycle	GO_BiologicalProcess-EBI-UniProt-GOA-ACAP-ARAP_08.05.2020_00h00	0,0102085	0,040806182	8,4728E-05	0,00032913	[6, 7, 8, 9]	Group100	9,35	10,00
GO:0031571	mitotic G1 DNA damage checkpoint	GO_BiologicalProcess-EBI-UniProt-GOA-ACAP-ARAP_08.05.2020_00h00	0,00103098	0,009002645	8,4728E-05	0,00032913	[5, 6, 7, 8, 9, 10, 11]	Group100	13,85	9,00
GO:0008134	transcription factor binding	GO_MolecularFunction-EBI-UniProt-GOA-ACAP-ARAP_08.05.2020_00h00	1,4931E-05	0,00023302	0,0002087	0,0007807	[3]	Group070	7,31	53,00

GO:0051427	hormone receptor binding	GO_MolecularFunction-EBI-UniProt-GOA-ACAP-ARAP_08.05.2020_00h00	0,00578301	0,028661067	0,0002087	0,0007807	[4]	Group070	8,08	16,00
GO:0140297	DNA-binding transcription factor binding	GO_MolecularFunction-EBI-UniProt-GOA-ACAP-ARAP_08.05.2020_00h00	0,00583479	0,028820314	0,0002087	0,0007807	[4]	Group070	6,79	26,00
GO:0061629	RNA polymerase II-specific DNA-binding transcription factor binding	GO_MolecularFunction-EBI-UniProt-GOA-ACAP-ARAP_08.05.2020_00h00	0,00928244	0,038576037	0,0002087	0,0007807	[5]	Group070	6,98	21,00
GO:0003712	transcription coregulator activity	GO_BiologicalProcess-EBI-UniProt-GOA-ACAP-ARAP_08.05.2020_00h00	5,4984E-08	1,68046E-06	0,0002087	0,0007807	[7, 8, 9, 10]	Group070	8,65	55,00
GO:0006357	regulation of transcription by RNA polymerase II	GO_BiologicalProcess-EBI-UniProt-GOA-ACAP-ARAP_08.05.2020_00h00	0,0060812	0,029345805	0,0002087	0,0007807	[6, 7, 8, 9, 10, 11]	Group070	4,98	114,00
GO:0032535	regulation of cellular component size	GO_BiologicalProcess-EBI-UniProt-GOA-ACAP-ARAP_08.05.2020_00h00	0,00677551	0,031158835	0,00023345	0,00084207	[4]	Group056	6,63	27,00
GO:0033043	regulation of organelle organization	GO_BiologicalProcess-EBI-UniProt-GOA-ACAP-ARAP_08.05.2020_00h00	0,00119093	0,009706078	0,00023345	0,00084207	[4, 5]	Group056	5,62	78,00
GO:0010638	positive regulation of organelle organization	GO_BiologicalProcess-EBI-UniProt-GOA-ACAP-ARAP_08.05.2020_00h00	0,00249017	0,016381536	0,00023345	0,00084207	[4, 5, 6]	Group056	6,23	43,00
GO:0051493	regulation of cytoskeleton organization	GO_BiologicalProcess-EBI-UniProt-GOA-ACAP-ARAP_08.05.2020_00h00	0,01067142	0,041415282	0,00023345	0,00084207	[5, 6]	Group056	5,99	35,00
GO:0044843	cell cycle G1/S phase transition	GO_BiologicalProcess-EBI-UniProt-GOA-ACAP-ARAP_08.05.2020_00h00	0,01147434	0,043721698	0,00031389	0,00105677	[4, 5]	Group081	6,94	20,00
GO:0006260	DNA replication	GO_BiologicalProcess-EBI-UniProt-GOA-ACAP-ARAP_08.05.2020_00h00	0,00100659	0,00889563	0,00031389	0,00105677	[5, 6]	Group081	7,97	24,00
GO:0006261	DNA-dependent DNA replication	GO_BiologicalProcess-EBI-UniProt-GOA-ACAP-ARAP_08.05.2020_00h00	0,00036649	0,003924393	0,00031389	0,00105677	[6, 7]	Group081	10,18	17,00
GO:0006270	DNA replication initiation	GO_BiologicalProcess-EBI-UniProt-GOA-ACAP-ARAP_08.05.2020_00h00	0,00754438	0,033136562	0,00031389	0,00105677	[5, 6, 7, 8]	Group081	13,64	6,00
GO:0033260	nuclear DNA replication	GO_BiologicalProcess-EBI-UniProt-GOA-ACAP-ARAP_08.05.2020_00h00	0,01330857	0,047387552	0,00031389	0,00105677	[4, 5, 8, 9]	Group081	10,94	7,00
GO:0006271	DNA strand elongation involved in DNA replication	GO_BiologicalProcess-EBI-UniProt-GOA-ACAP-ARAP_08.05.2020_00h00	0,00722575	0,032317616	0,00031389	0,00105677	[6, 7, 8]	Group081	20,00	4,00
GO:0000083	regulation of transcription involved in G1/S transition of mitotic cell cycle	GO_BiologicalProcess-EBI-UniProt-GOA-ACAP-ARAP_08.05.2020_00h00	0,0133187	0,04730878	0,00031389	0,00105677	[4, 5, 6, 7, 8, 9, 10, 11]	Group081	13,89	5,00
GO:0000727	double-strand break repair via break-induced replication	GO_BiologicalProcess-EBI-UniProt-GOA-ACAP-ARAP_08.05.2020_00h00	0,00065447	0,006487218	0,00031389	0,00105677	[8, 9, 10]	Group081	36,36	4,00
GO:0050772	positive regulation of axonogenesis	GO_BiologicalProcess-EBI-UniProt-GOA-ACAP-ARAP_08.05.2020_00h00	0,0013666	0,010551599	0,00030644	0,00106727	[6, 7, 8, 9, 10, 11, 12, 13, 14]	Group103	11,58	11,00
GO:0048518	positive regulation of biological process	GO_BiologicalProcess-EBI-UniProt-GOA-ACAP-ARAP_08.05.2020_00h00	0,00105434	0,00899257	0,00030644	0,00106727	[1, 2, 3]	Group103	4,59	301,00
GO:0023051	regulation of signaling	GO_BiologicalProcess-EBI-UniProt-GOA-ACAP-ARAP_08.05.2020_00h00	0,00106459	0,008975607	0,00030644	0,00106727	[2, 3]	Group103	4,87	187,00
GO:0048583	regulation of response to stimulus	GO_BiologicalProcess-EBI-UniProt-GOA-ACAP-ARAP_08.05.2020_00h00	0,0140207	0,049089178	0,00030644	0,00106727	[2, 3]	Group103	4,55	206,00
GO:0050793	regulation of developmental process	GO_BiologicalProcess-EBI-UniProt-GOA-ACAP-ARAP_08.05.2020_00h00	5,3426E-07	1,16979E-05	0,00030644	0,00106727	[2, 3]	Group103	5,74	161,00
GO:0051239	regulation of multicellular organismal process	GO_BiologicalProcess-EBI-UniProt-GOA-ACAP-ARAP_08.05.2020_00h00	1,3622E-05	0,00021487	0,00030644	0,00106727	[2, 3]	Group103	5,31	181,00
GO:0048522	positive regulation of cellular process	GO_BiologicalProcess-EBI-UniProt-GOA-ACAP-ARAP_08.05.2020_00h00	6,6865E-05	0,000899924	0,00030644	0,00106727	[2, 3, 4]	Group103	4,80	286,00

GO:0048584	positive regulation of response to stimulus	GO_BiologicalProcess-EBI-UniProt-GOA-ACAP-ARAP_08.05.2020_00h00	0,00860397	0,036691941	0,00030644	0,00106727	[2, 3, 4]	Group103	4,86	126,00
GO:0051093	negative regulation of developmental process	GO_BiologicalProcess-EBI-UniProt-GOA-ACAP-ARAP_08.05.2020_00h00	0,00806685	0,03511592	0,00030644	0,00106727	[2, 3, 4]	Group103	5,49	58,00
GO:0051094	positive regulation of developmental process	GO_BiologicalProcess-EBI-UniProt-GOA-ACAP-ARAP_08.05.2020_00h00	0,00128977	0,010118128	0,00030644	0,00106727	[2, 3, 4]	Group103	5,55	82,00
GO:0051240	positive regulation of multicellular organismal process	GO_BiologicalProcess-EBI-UniProt-GOA-ACAP-ARAP_08.05.2020_00h00	0,00121394	0,009731382	0,00030644	0,00106727	[2, 3, 4]	Group103	5,34	102,00
GO:0009790	embryo development	GO_BiologicalProcess-EBI-UniProt-GOA-ACAP-ARAP_08.05.2020_00h00	0,00450283	0,023507655	0,00030644	0,00106727	[3, 4]	Group103	5,56	63,00
GO:0010646	regulation of cell communication	GO_BiologicalProcess-EBI-UniProt-GOA-ACAP-ARAP_08.05.2020_00h00	0,00083746	0,007875319	0,00030644	0,00106727	[3, 4]	Group103	4,90	186,00
GO:0022603	regulation of anatomical structure morphogenesis	GO_BiologicalProcess-EBI-UniProt-GOA-ACAP-ARAP_08.05.2020_00h00	0,00284967	0,017713821	0,00030644	0,00106727	[3, 4]	Group103	5,62	66,00
GO:0030155	regulation of cell adhesion	GO_BiologicalProcess-EBI-UniProt-GOA-ACAP-ARAP_08.05.2020_00h00	0,0002975	0,003281432	0,00030644	0,00106727	[3, 4]	Group103	6,62	50,00
GO:0035295	tube development	GO_BiologicalProcess-EBI-UniProt-GOA-ACAP-ARAP_08.05.2020_00h00	0,00965585	0,039567392	0,00030644	0,00106727	[3, 4]	Group103	5,39	61,00
GO:0045595	regulation of cell differentiation	GO_BiologicalProcess-EBI-UniProt-GOA-ACAP-ARAP_08.05.2020_00h00	1,4649E-06	2,90403E-05	0,00030644	0,00106727	[3, 4]	Group103	6,06	120,00
GO:0048729	tissue morphogenesis	GO_BiologicalProcess-EBI-UniProt-GOA-ACAP-ARAP_08.05.2020_00h00	0,01362663	0,048285682	0,00030644	0,00106727	[3, 4]	Group103	5,69	41,00
GO:0060560	developmental growth involved in morphogenesis	GO_BiologicalProcess-EBI-UniProt-GOA-ACAP-ARAP_08.05.2020_00h00	0,00172929	0,012748064	0,00030644	0,00106727	[3, 4]	Group103	8,08	21,00
GO:2000026	regulation of multicellular organismal development	GO_BiologicalProcess-EBI-UniProt-GOA-ACAP-ARAP_08.05.2020_00h00	1,1047E-06	2,25077E-05	0,00030644	0,00106727	[3, 4]	Group103	5,95	132,00
GO:0002520	immune system development	GO_BiologicalProcess-EBI-UniProt-GOA-ACAP-ARAP_08.05.2020_00h00	2,2993E-06	4,32451E-05	0,00030644	0,00106727	[2, 4, 5]	Group103	6,92	74,00
GO:0035239	tube morphogenesis	GO_BiologicalProcess-EBI-UniProt-GOA-ACAP-ARAP_08.05.2020_00h00	0,00081955	0,007756677	0,00030644	0,00106727	[3, 4, 5]	Group103	6,15	56,00
GO:0045596	negative regulation of cell differentiation	GO_BiologicalProcess-EBI-UniProt-GOA-ACAP-ARAP_08.05.2020_00h00	0,00273721	0,01738303	0,00030644	0,00106727	[3, 4, 5]	Group103	6,06	47,00
GO:0045597	positive regulation of cell differentiation	GO_BiologicalProcess-EBI-UniProt-GOA-ACAP-ARAP_08.05.2020_00h00	0,00057936	0,005943533	0,00030644	0,00106727	[3, 4, 5]	Group103	6,03	64,00
GO:0051130	positive regulation of cellular component organization	GO_BiologicalProcess-EBI-UniProt-GOA-ACAP-ARAP_08.05.2020_00h00	0,00397208	0,021988861	0,00030644	0,00106727	[3, 4, 5]	Group103	5,46	71,00
GO:0001655	urogenital system development	GO_BiologicalProcess-EBI-UniProt-GOA-ACAP-ARAP_08.05.2020_00h00	0,01173816	0,044267047	0,00030644	0,00106727	[4, 5]	Group103	6,56	24,00
GO:0001667	ameboidal-type cell migration	GO_BiologicalProcess-EBI-UniProt-GOA-ACAP-ARAP_08.05.2020_00h00	0,00238158	0,016026519	0,00030644	0,00106727	[4, 5]	Group103	6,93	30,00
GO:0009792	embryo development ending in birth or egg hatching	GO_BiologicalProcess-EBI-UniProt-GOA-ACAP-ARAP_08.05.2020_00h00	0,00035885	0,003870864	0,00030644	0,00106727	[4, 5]	Group103	6,65	48,00
GO:0048534	hematopoietic or lymphoid organ development	GO_BiologicalProcess-EBI-UniProt-GOA-ACAP-ARAP_08.05.2020_00h00	8,8905E-07	1,89019E-05	0,00030644	0,00106727	[3, 4, 5, 6]	Group103	7,16	72,00
GO:0060541	respiratory system development	GO_BiologicalProcess-EBI-UniProt-GOA-ACAP-ARAP_08.05.2020_00h00	0,00734388	0,032548264	0,00030644	0,00106727	[4, 5]	Group103	7,69	17,00

GO:0061138	morphogenesis of a branching epithelium	GO_BiologicalProcess-EBI-UniProt-GOA-ACAP-ARAP_08.05.2020_00h00	0,00284655	0,017769769	0,00030644	0,00106727	[3, 4, 5, 6]	Group103	8,46	17,00
GO:0072359	circulatory system development	GO_BiologicalProcess-EBI-UniProt-GOA-ACAP-ARAP_08.05.2020_00h00	0,00149302	0,01123211	0,00030644	0,00106727	[4, 5]	Group103	5,75	67,00
GO:0010631	epithelial cell migration	GO_BiologicalProcess-EBI-UniProt-GOA-ACAP-ARAP_08.05.2020_00h00	0,00058844	0,005994724	0,00030644	0,00106727	[4, 5, 6]	Group103	8,14	25,00
GO:0010632	regulation of epithelial cell migration	GO_BiologicalProcess-EBI-UniProt-GOA-ACAP-ARAP_08.05.2020_00h00	0,00137465	0,010558186	0,00030644	0,00106727	[3, 4, 5, 6, 7]	Group103	8,40	20,00
GO:0010720	positive regulation of cell development	GO_BiologicalProcess-EBI-UniProt-GOA-ACAP-ARAP_08.05.2020_00h00	0,01184334	0,044209119	0,00030644	0,00106727	[4, 5, 6]	Group103	5,91	36,00
GO:0030334	regulation of cell migration	GO_BiologicalProcess-EBI-UniProt-GOA-ACAP-ARAP_08.05.2020_00h00	0,01196401	0,044209588	0,00030644	0,00106727	[4, 5, 6]	Group103	5,50	51,00
GO:0048732	gland development	GO_BiologicalProcess-EBI-UniProt-GOA-ACAP-ARAP_08.05.2020_00h00	0,00223689	0,015262903	0,00030644	0,00106727	[4, 5, 6]	Group103	6,69	34,00
GO:0051145	smooth muscle cell differentiation	GO_BiologicalProcess-EBI-UniProt-GOA-ACAP-ARAP_08.05.2020_00h00	0,01034567	0,040908623	0,00030644	0,00106727	[5]	Group103	11,48	7,00
GO:0060562	epithelial tube morphogenesis	GO_BiologicalProcess-EBI-UniProt-GOA-ACAP-ARAP_08.05.2020_00h00	0,00102574	0,009010539	0,00030644	0,00106727	[4, 5, 6]	Group103	7,61	27,00
GO:0072358	cardiovascular system development	GO_BiologicalProcess-EBI-UniProt-GOA-ACAP-ARAP_08.05.2020_00h00	0,00195144	0,013964712	0,00030644	0,00106727	[4, 5, 6]	Group103	6,14	48,00
GO:0001822	kidney development	GO_BiologicalProcess-EBI-UniProt-GOA-ACAP-ARAP_08.05.2020_00h00	0,01183102	0,044275783	0,00030644	0,00106727	[4, 5, 6, 7]	Group103	6,82	21,00
GO:0030097	hemopoiesis	GO_BiologicalProcess-EBI-UniProt-GOA-ACAP-ARAP_08.05.2020_00h00	1,4609E-06	2,9358E-05	0,00030644	0,00106727	[4, 5, 6, 7]	Group103	7,17	69,00
GO:0048754	branching morphogenesis of an epithelial tube	GO_BiologicalProcess-EBI-UniProt-GOA-ACAP-ARAP_08.05.2020_00h00	0,00247505	0,016355416	0,00030644	0,00106727	[4, 5, 6, 7]	Group103	9,09	15,00
GO:1903706	regulation of hemopoiesis	GO_BiologicalProcess-EBI-UniProt-GOA-ACAP-ARAP_08.05.2020_00h00	6,525E-06	0,000110026	0,00030644	0,00106727	[3, 4, 5, 6, 7, 8]	Group103	8,27	42,00
GO:0010594	regulation of endothelial cell migration	GO_BiologicalProcess-EBI-UniProt-GOA-ACAP-ARAP_08.05.2020_00h00	0,00980666	0,039631858	0,00030644	0,00106727	[4, 5, 6, 7, 8]	Group103	8,05	14,00
GO:0010770	positive regulation of cell morphogenesis involved in differentiation	GO_BiologicalProcess-EBI-UniProt-GOA-ACAP-ARAP_08.05.2020_00h00	0,00329477	0,019333733	0,00030644	0,00106727	[5, 6, 7]	Group103	8,82	15,00
GO:0043542	endothelial cell migration	GO_BiologicalProcess-EBI-UniProt-GOA-ACAP-ARAP_08.05.2020_00h00	0,00432958	0,023180651	0,00030644	0,00106727	[5, 6, 7]	Group103	7,93	18,00
GO:1901342	regulation of vasculature development	GO_BiologicalProcess-EBI-UniProt-GOA-ACAP-ARAP_08.05.2020_00h00	0,01138038	0,043590143	0,00030644	0,00106727	[4, 5, 6, 7, 8]	Group103	6,58	24,00
GO:0048514	blood vessel morphogenesis	GO_BiologicalProcess-EBI-UniProt-GOA-ACAP-ARAP_08.05.2020_00h00	0,00257482	0,016787833	0,00030644	0,00106727	[4, 5, 6, 7, 8, 9]	Group103	6,30	41,00
GO:0050769	positive regulation of neurogenesis	GO_BiologicalProcess-EBI-UniProt-GOA-ACAP-ARAP_08.05.2020_00h00	0,0070058	0,03172072	0,00030644	0,00106727	[4, 5, 6, 7, 8, 9]	Group103	6,26	33,00
GO:0060976	coronary vasculature development	GO_BiologicalProcess-EBI-UniProt-GOA-ACAP-ARAP_08.05.2020_00h00	0,00344449	0,020051828	0,00030644	0,00106727	[4, 5, 6, 7, 8, 9]	Group103	14,00	7,00
GO:0072073	kidney epithelium development	GO_BiologicalProcess-EBI-UniProt-GOA-ACAP-ARAP_08.05.2020_00h00	0,01053228	0,041092683	0,00030644	0,00106727	[5, 6, 7, 8]	Group103	8,23	13,00
GO:0001570	vasculogenesis	GO_BiologicalProcess-EBI-UniProt-GOA-ACAP-ARAP_08.05.2020_00h00	0,00884597	0,037290338	0,00030644	0,00106727	[4, 5, 6, 7, 8, 9, 10]	Group103	10,11	9,00
GO:0002040	sprouting angiogenesis	GO_BiologicalProcess-EBI-UniProt-GOA-ACAP-ARAP_08.05.2020_00h00	0,00390121	0,021843806	0,00030644	0,00106727	[4, 5, 6, 7, 8, 9, 10, 11]	Group103	9,68	12,00

GO:0072080	nephron tubule development	GO_BiologicalProcess-EBI-UniProt-GOA-ACAP-ARAP_08.05.2020_00h00	0,00224538	0,015179563	0,00030644	0,00106727	[5, 6, 7, 8, 9, 10]	Group103	10,89	11,00
GO:0001657	ureteric bud development	GO_BiologicalProcess-EBI-UniProt-GOA-ACAP-ARAP_08.05.2020_00h00	0,00437197	0,023237992	0,00030644	0,00106727	[5, 6, 8, 9, 10, 11]	Group103	10,00	11,00
GO:1903670	regulation of sprouting angiogenesis	GO_BiologicalProcess-EBI-UniProt-GOA-ACAP-ARAP_08.05.2020_00h00	0,00972408	0,03962563	0,00030644	0,00106727	[5, 6, 7, 8, 9, 10, 11, 12]	Group103	10,67	8,00
GO:0060675	ureteric bud morphogenesis	GO_BiologicalProcess-EBI-UniProt-GOA-ACAP-ARAP_08.05.2020_00h00	0,00194483	0,013985601	0,00030644	0,00106727	[6, 7, 8, 9, 10, 11, 12, 13]	Group103	12,68	9,00
GO:0008104	protein localization	GO_BiologicalProcess-EBI-UniProt-GOA-ACAP-ARAP_08.05.2020_00h00	0,004388	0,023238959	0,0003961	0,00129052	[3]	Group082	4,86	149,00
GO:0051649	establishment of localization in cell	GO_BiologicalProcess-EBI-UniProt-GOA-ACAP-ARAP_08.05.2020_00h00	0,00120636	0,009777542	0,0003961	0,00129052	[3]	Group082	5,02	149,00
GO:0045184	establishment of protein localization	GO_BiologicalProcess-EBI-UniProt-GOA-ACAP-ARAP_08.05.2020_00h00	0,00678293	0,03109551	0,0003961	0,00129052	[3, 4]	Group082	4,96	116,00
GO:0046907	intracellular transport	GO_BiologicalProcess-EBI-UniProt-GOA-ACAP-ARAP_08.05.2020_00h00	0,00093649	0,008480422	0,0003961	0,00129052	[3, 4]	Group082	5,37	103,00
GO:0033365	protein localization to organelle	GO_BiologicalProcess-EBI-UniProt-GOA-ACAP-ARAP_08.05.2020_00h00	6,7926E-05	0,00089773	0,0003961	0,00129052	[5]	Group082	6,44	67,00
GO:0072594	establishment of protein localization to organelle	GO_BiologicalProcess-EBI-UniProt-GOA-ACAP-ARAP_08.05.2020_00h00	0,00986942	0,039775934	0,0003961	0,00129052	[4, 5, 6]	Group082	5,95	37,00
GO:0015031	protein transport	GO_BiologicalProcess-EBI-UniProt-GOA-ACAP-ARAP_08.05.2020_00h00	0,01433205	0,049940895	0,0003961	0,00129052	[4, 5, 6, 7]	Group082	4,87	109,00
GO:0034504	protein localization to nucleus	GO_BiologicalProcess-EBI-UniProt-GOA-ACAP-ARAP_08.05.2020_00h00	0,00091803	0,008470153	0,0003961	0,00129052	[6]	Group082	8,03	24,00
GO:0051128	regulation of cellular component organization	GO_BiologicalProcess-EBI-UniProt-GOA-ACAP-ARAP_08.05.2020_00h00	0,00053505	0,005566799	0,00053505	0,00168875	[3, 4]	Group074	5,21	133,00
GO:0051130	positive regulation of cellular component organization	GO_BiologicalProcess-EBI-UniProt-GOA-ACAP-ARAP_08.05.2020_00h00	0,00397208	0,021988861	0,00053505	0,00168875	[3, 4, 5]	Group074	5,46	71,00
GO:0033043	regulation of organelle organization	GO_BiologicalProcess-EBI-UniProt-GOA-ACAP-ARAP_08.05.2020_00h00	0,00119093	0,009706078	0,00053505	0,00168875	[4, 5]	Group074	5,62	78,00
GO:0010638	positive regulation of organelle organization	GO_BiologicalProcess-EBI-UniProt-GOA-ACAP-ARAP_08.05.2020_00h00	0,00249017	0,016381536	0,00053505	0,00168875	[4, 5, 6]	Group074	6,23	43,00
GO:0033044	regulation of chromosome organization	GO_BiologicalProcess-EBI-UniProt-GOA-ACAP-ARAP_08.05.2020_00h00	0,0056442	0,028067946	0,00053505	0,00168875	[5, 6]	Group074	6,81	26,00
GO:0051493	regulation of cytoskeleton organization	GO_BiologicalProcess-EBI-UniProt-GOA-ACAP-ARAP_08.05.2020_00h00	0,01067142	0,041415282	0,00053505	0,00168875	[5, 6]	Group074	5,99	35,00
GO:0060534	trachea cartilage development	GO_BiologicalProcess-EBI-UniProt-GOA-ACAP-ARAP_08.05.2020_00h00	0,003019	0,018608693	0,00055479	0,00169801	[5, 6, 7]	Group057	37,50	3,00
GO:0060740	prostate gland epithelium morphogenesis	GO_BiologicalProcess-EBI-UniProt-GOA-ACAP-ARAP_08.05.2020_00h00	0,00526885	0,026561538	0,00055479	0,00169801	[3, 4, 5, 6, 7, 8, 9]	Group057	17,24	5,00
GO:0070384	Harderian gland development	GO_BiologicalProcess-EBI-UniProt-GOA-ACAP-ARAP_08.05.2020_00h00	0,00024314	0,002743778	0,00055479	0,00169801	[5, 6, 7]	Group057	75,00	3,00
GO:0060528	secretory columnal luminar epithelial cell differentiation involved in prostate glandular acinus development	GO_BiologicalProcess-EBI-UniProt-GOA-ACAP-ARAP_08.05.2020_00h00	0,00024314	0,002743778	0,00055479	0,00169801	[4, 5, 6, 7, 8, 9]	Group057	75,00	3,00
GO:1903828	negative regulation of cellular protein localization	GO_BiologicalProcess-EBI-UniProt-GOA-ACAP-ARAP_08.05.2020_00h00	0,00940467	0,038754635	0,00059223	0,00170899	[3, 4, 5, 6]	Group059	9,02	11,00

GO:0034504	protein localization to nucleus	GO_BiologicalProcess-EBI-UniProt-GOA-ACAP-ARAP_08.05.2020_00h00	0,00091803	0,008470153	0,00059223	0,00170899	[6]	Group059	8,03	24,00
GO:1900180	regulation of protein localization to nucleus	GO_BiologicalProcess-EBI-UniProt-GOA-ACAP-ARAP_08.05.2020_00h00	0,00344332	0,020124931	0,00059223	0,00170899	[5, 6, 7]	Group059	9,42	13,00
GO:1900181	negative regulation of protein localization to nucleus	GO_BiologicalProcess-EBI-UniProt-GOA-ACAP-ARAP_08.05.2020_00h00	0,0092426	0,038519599	0,00059223	0,00170899	[4, 5, 6, 7, 8]	Group059	15,15	5,00
GO:2000047	regulation of cell-cell adhesion mediated by cadherin	GO_BiologicalProcess-EBI-UniProt-GOA-ACAP-ARAP_08.05.2020_00h00	0,00146997	0,011115676	0,00064021	0,00174761	[5, 6]	Group044	22,73	5,00
GO:0060740	prostate gland epithelium morphogenesis	GO_BiologicalProcess-EBI-UniProt-GOA-ACAP-ARAP_08.05.2020_00h00	0,00526885	0,026561538	0,00064021	0,00174761	[3, 4, 5, 6, 7, 8, 9]	Group044	17,24	5,00
GO:0060528	secretory columnal luminar epithelial cell differentiation involved in prostate glandular acinus development	GO_BiologicalProcess-EBI-UniProt-GOA-ACAP-ARAP_08.05.2020_00h00	0,00024314	0,002743778	0,00064021	0,00174761	[4, 5, 6, 7, 8, 9]	Group044	75,00	3,00
GO:0045917	positive regulation of complement activation	GO_BiologicalProcess-EBI-UniProt-GOA-ACAP-ARAP_08.05.2020_00h00	0,00058983	0,005967495	0,00058983	0,00175215	[4, 5, 6, 7, 8, 9]	Group031	60,00	3,00
GO:0060348	bone development	GO_BiologicalProcess-EBI-UniProt-GOA-ACAP-ARAP_08.05.2020_00h00	0,00251774	0,01648893	0,000634	0,00177872	[4, 5, 6]	Group048	8,15	19,00
GO:0098751	bone cell development	GO_BiologicalProcess-EBI-UniProt-GOA-ACAP-ARAP_08.05.2020_00h00	0,0133187	0,04730878	0,000634	0,00177872	[4, 5, 6, 7]	Group048	13,89	5,00
GO:0061515	myeloid cell development	GO_BiologicalProcess-EBI-UniProt-GOA-ACAP-ARAP_08.05.2020_00h00	0,00372574	0,021267148	0,000634	0,00177872	[4, 5, 6, 7, 8, 9]	Group048	11,54	9,00
GO:0016032	viral process	GO_BiologicalProcess-EBI-UniProt-GOA-ACAP-ARAP_08.05.2020_00h00	0,0006823	0,006672869	0,0006823	0,00181347	[3]	Group016	6,11	59,00
GO:0050772	positive regulation of axonogenesis	GO_BiologicalProcess-EBI-UniProt-GOA-ACAP-ARAP_08.05.2020_00h00	0,0013666	0,010551599	0,00075655	0,00195927	[6, 7, 8, 9, 10, 11, 12, 13, 14]	Group061	11,58	11,00
GO:0032535	regulation of cellular component size	GO_BiologicalProcess-EBI-UniProt-GOA-ACAP-ARAP_08.05.2020_00h00	0,00677551	0,031158835	0,00075655	0,00195927	[4]	Group061	6,63	27,00
GO:0051493	regulation of cytoskeleton organization	GO_BiologicalProcess-EBI-UniProt-GOA-ACAP-ARAP_08.05.2020_00h00	0,01067142	0,041415282	0,00075655	0,00195927	[5, 6]	Group061	5,99	35,00
GO:0010770	positive regulation of cell morphogenesis involved in differentiation	GO_BiologicalProcess-EBI-UniProt-GOA-ACAP-ARAP_08.05.2020_00h00	0,00329477	0,019333733	0,00075655	0,00195927	[5, 6, 7]	Group061	8,82	15,00
GO:0019827	stem cell population maintenance	GO_BiologicalProcess-EBI-UniProt-GOA-ACAP-ARAP_08.05.2020_00h00	0,00086558	0,00808796	0,00086558	0,00213229	[2, 3]	Group055	9,44	17,00
GO:0035019	somatic stem cell population maintenance	GO_BiologicalProcess-EBI-UniProt-GOA-ACAP-ARAP_08.05.2020_00h00	0,00150131	0,011236882	0,00086558	0,00213229	[3, 4]	Group055	12,20	10,00
GO:2000036	regulation of stem cell population maintenance	GO_BiologicalProcess-EBI-UniProt-GOA-ACAP-ARAP_08.05.2020_00h00	0,00272172	0,01735981	0,00086558	0,00213229	[3, 4]	Group055	16,67	6,00
GO:1904672	regulation of somatic stem cell population maintenance	GO_BiologicalProcess-EBI-UniProt-GOA-ACAP-ARAP_08.05.2020_00h00	0,00609474	0,029314715	0,00086558	0,00213229	[4, 5]	Group055	30,00	3,00
GO:1901135	carbohydrate derivative metabolic process	GO_BiologicalProcess-EBI-UniProt-GOA-ACAP-ARAP_08.05.2020_00h00	0,01060116	0,041251745	0,00084797	0,00214114	[3]	Group085	5,31	65,00
GO:1901137	carbohydrate derivative biosynthetic process	GO_BiologicalProcess-EBI-UniProt-GOA-ACAP-ARAP_08.05.2020_00h00	0,01074612	0,04148569	0,00084797	0,00214114	[4]	Group085	5,75	42,00
GO:1901566	organonitrogen compound biosynthetic process	GO_BiologicalProcess-EBI-UniProt-GOA-ACAP-ARAP_08.05.2020_00h00	0,00087312	0,008106748	0,00084797	0,00214114	[4]	Group085	5,38	103,00
GO:0006753	nucleoside phosphate metabolic process	GO_BiologicalProcess-EBI-UniProt-GOA-ACAP-ARAP_08.05.2020_00h00	0,00843541	0,036077995	0,00084797	0,00214114	[4, 5, 6]	Group085	6,05	36,00

GO:0072522	purine-containing compound biosynthetic process	GO_BiologicalProcess-EBI-UniProt-GOA-ACAP-ARAP_08.05.2020_00h00	0,00871317	0,036836375	0,00084797	0,00214114	[5]	Group085	7,56	17,00
GO:0009165	nucleotide biosynthetic process	GO_BiologicalProcess-EBI-UniProt-GOA-ACAP-ARAP_08.05.2020_00h00	0,00030839	0,003376185	0,00084797	0,00214114	[6, 7, 8]	Group085	8,36	26,00
GO:0009199	ribonucleoside triphosphate metabolic process	GO_BiologicalProcess-EBI-UniProt-GOA-ACAP-ARAP_08.05.2020_00h00	0,01164843	0,044041872	0,00084797	0,00214114	[6, 7, 8]	Group085	9,68	9,00
GO:0009260	ribonucleotide biosynthetic process	GO_BiologicalProcess-EBI-UniProt-GOA-ACAP-ARAP_08.05.2020_00h00	0,00268579	0,017205471	0,00084797	0,00214114	[6, 7, 8, 9]	Group085	8,29	18,00
GO:0042776	mitochondrial ATP synthesis coupled proton transport	GO_BiologicalProcess-EBI-UniProt-GOA-ACAP-ARAP_08.05.2020_00h00	0,01397906	0,049060475	0,00084797	0,00214114	[4, 5, 6, 7, 9, 10, 11, 12, 13]	Group085	16,67	4,00
GO:0009152	purine ribonucleotide biosynthetic process	GO_BiologicalProcess-EBI-UniProt-GOA-ACAP-ARAP_08.05.2020_00h00	0,00697539	0,031680774	0,00084797	0,00214114	[7, 8, 9, 10]	Group085	7,92	16,00
GO:0000491	small nucleolar ribonucleoprotein complex assembly	GO_BiologicalProcess-EBI-UniProt-GOA-ACAP-ARAP_08.05.2020_00h00	0,00095093	0,008506158	0,00095093	0,00218281	[6, 7, 8]	Group010	33,33	4,00
GO:0030291	protein serine/threonine kinase inhibitor activity	GO_BiologicalProcess-EBI-UniProt-GOA-ACAP-ARAP_08.05.2020_00h00	0,00021557	0,002490113	0,00093892	0,00220537	[8, 9, 10, 11, 12]	Group090	21,88	7,00
GO:0060541	respiratory system development	GO_BiologicalProcess-EBI-UniProt-GOA-ACAP-ARAP_08.05.2020_00h00	0,00734388	0,032548264	0,00093892	0,00220537	[4, 5]	Group090	7,69	17,00
GO:0030856	regulation of epithelial cell differentiation	GO_BiologicalProcess-EBI-UniProt-GOA-ACAP-ARAP_08.05.2020_00h00	0,00151781	0,011302683	0,00093892	0,00220537	[4, 5, 6]	Group090	9,55	15,00
GO:0060485	mesenchyme development	GO_BiologicalProcess-EBI-UniProt-GOA-ACAP-ARAP_08.05.2020_00h00	0,00693329	0,031685803	0,00093892	0,00220537	[4, 5, 6]	Group090	7,17	21,00
GO:0030857	negative regulation of epithelial cell differentiation	GO_BiologicalProcess-EBI-UniProt-GOA-ACAP-ARAP_08.05.2020_00h00	2,2595E-05	0,000338235	0,00093892	0,00220537	[4, 5, 6, 7]	Group090	20,00	10,00
GO:0001837	epithelial to mesenchymal transition	GO_BiologicalProcess-EBI-UniProt-GOA-ACAP-ARAP_08.05.2020_00h00	7,1331E-05	0,000934306	0,00093892	0,00220537	[5, 6, 7, 8]	Group090	11,64	17,00
GO:0010717	regulation of epithelial to mesenchymal transition	GO_BiologicalProcess-EBI-UniProt-GOA-ACAP-ARAP_08.05.2020_00h00	0,0010473	0,008984704	0,00093892	0,00220537	[4, 5, 6, 7, 8, 9]	Group090	11,96	11,00
GO:0010718	positive regulation of epithelial to mesenchymal transition	GO_BiologicalProcess-EBI-UniProt-GOA-ACAP-ARAP_08.05.2020_00h00	0,01147846	0,043511376	0,00093892	0,00220537	[3, 4, 5, 6, 7, 8, 9, 10]	Group090	12,50	6,00
GO:0010719	negative regulation of epithelial to mesenchymal transition	GO_BiologicalProcess-EBI-UniProt-GOA-ACAP-ARAP_08.05.2020_00h00	0,01184689	0,044110134	0,00093892	0,00220537	[3, 4, 5, 6, 7, 8, 9, 10]	Group090	14,29	5,00
GO:2000826	regulation of heart morphogenesis	GO_BiologicalProcess-EBI-UniProt-GOA-ACAP-ARAP_08.05.2020_00h00	0,00754438	0,033136562	0,00093892	0,00220537	[5, 6, 7, 8]	Group090	13,64	6,00
GO:1902747	negative regulation of lens fiber cell differentiation	GO_BiologicalProcess-EBI-UniProt-GOA-ACAP-ARAP_08.05.2020_00h00	0,00114476	0,009434626	0,00093892	0,00220537	[3, 4, 5, 6, 7, 8, 9, 10, 11, 12]	Group090	50,00	3,00
GO:0004860	protein kinase inhibitor activity	GO_BiologicalProcess-EBI-UniProt-GOA-ACAP-ARAP_08.05.2020_00h00	0,00026167	0,002908152	0,00093892	0,00220537	[7, 8, 9, 10, 11]	Group090	15,15	10,00
GO:0043412	macromolecule modification	GO_BiologicalProcess-EBI-UniProt-GOA-ACAP-ARAP_08.05.2020_00h00	0,00093585	0,008527306	0,00093585	0,0022505	[4]	Group065	4,78	220,00
GO:0006464	cellular protein modification process	GO_BiologicalProcess-EBI-UniProt-GOA-ACAP-ARAP_08.05.2020_00h00	0,00113649	0,00941937	0,00093585	0,0022505	[5, 6]	Group065	4,79	210,00
GO:0018193	peptidyl-amino acid modification	GO_BiologicalProcess-EBI-UniProt-GOA-ACAP-ARAP_08.05.2020_00h00	9,2461E-05	0,001169311	0,00093585	0,0022505	[6, 7]	Group065	6,02	83,00
GO:0016570	histone modification	GO_BiologicalProcess-EBI-UniProt-GOA-ACAP-ARAP_08.05.2020_00h00	0,00012397	0,00150305	0,00093585	0,0022505	[6, 7, 8]	Group065	7,63	37,00

GO:0018205	peptidyl-lysine modification	GO_BiologicalProcess-EBI-UniProt-GOA-ACAP-ARAP_08.05.2020_00h00	2,6372E-06	4,83594E-05	0,00093585	0,0022505	[7, 8]	Group065	8,98	38,00
GO:0051642	centrosome localization	GO_BiologicalProcess-EBI-UniProt-GOA-ACAP-ARAP_08.05.2020_00h00	0,00121765	0,009708149	0,00121765	0,00273296	[3, 4]	Group003	19,35	6,00
GO:0051348	negative regulation of transferase activity	GO_BiologicalProcess-EBI-UniProt-GOA-ACAP-ARAP_08.05.2020_00h00	0,00220605	0,015337819	0,00126559	0,00277879	[5]	Group064	7,64	23,00
GO:0033673	negative regulation of kinase activity	GO_BiologicalProcess-EBI-UniProt-GOA-ACAP-ARAP_08.05.2020_00h00	0,00238547	0,0159794	0,00126559	0,00277879	[6, 7, 8, 9]	Group064	7,87	21,00
GO:0006469	negative regulation of protein kinase activity	GO_BiologicalProcess-EBI-UniProt-GOA-ACAP-ARAP_08.05.2020_00h00	0,0043703	0,023313547	0,00126559	0,00277879	[7, 8, 9, 10]	Group064	7,76	19,00
GO:0050730	regulation of peptidyl-tyrosine phosphorylation	GO_BiologicalProcess-EBI-UniProt-GOA-ACAP-ARAP_08.05.2020_00h00	0,00470406	0,024213506	0,00126559	0,00277879	[7, 8, 9, 10]	Group064	7,42	21,00
GO:0050732	negative regulation of peptidyl-tyrosine phosphorylation	GO_BiologicalProcess-EBI-UniProt-GOA-ACAP-ARAP_08.05.2020_00h00	0,0059004	0,028949445	0,00126559	0,00277879	[7, 8, 9, 10, 11]	Group064	12,73	7,00
GO:0050772	positive regulation of axonogenesis	GO_BiologicalProcess-EBI-UniProt-GOA-ACAP-ARAP_08.05.2020_00h00	0,0013666	0,010551599	0,00155291	0,00333711	[6, 7, 8, 9, 10, 11, 12, 13, 14]	Group045	11,58	11,00
GO:0040008	regulation of growth	GO_BiologicalProcess-EBI-UniProt-GOA-ACAP-ARAP_08.05.2020_00h00	0,00210161	0,01482239	0,00155291	0,00333711	[2, 3]	Group045	6,22	45,00
GO:0001558	regulation of cell growth	GO_BiologicalProcess-EBI-UniProt-GOA-ACAP-ARAP_08.05.2020_00h00	0,00221933	0,015357364	0,00155291	0,00333711	[3, 4, 5]	Group045	6,89	31,00
GO:0019827	stem cell population maintenance	GO_BiologicalProcess-EBI-UniProt-GOA-ACAP-ARAP_08.05.2020_00h00	0,00086558	0,00808796	0,00184444	0,0038018	[2, 3]	Group096	9,44	17,00
GO:0035019	somatic stem cell population maintenance	GO_BiologicalProcess-EBI-UniProt-GOA-ACAP-ARAP_08.05.2020_00h00	0,00150131	0,011236882	0,00184444	0,0038018	[3, 4]	Group096	12,20	10,00
GO:2000036	regulation of stem cell population maintenance	GO_BiologicalProcess-EBI-UniProt-GOA-ACAP-ARAP_08.05.2020_00h00	0,00272172	0,01735981	0,00184444	0,0038018	[3, 4]	Group096	16,67	6,00
GO:1904672	regulation of somatic stem cell population maintenance	GO_BiologicalProcess-EBI-UniProt-GOA-ACAP-ARAP_08.05.2020_00h00	0,00609474	0,029314715	0,00184444	0,0038018	[4, 5]	Group096	30,00	3,00
GO:2000736	regulation of stem cell differentiation	GO_BiologicalProcess-EBI-UniProt-GOA-ACAP-ARAP_08.05.2020_00h00	0,01182202	0,044355243	0,00184444	0,0038018	[4, 5]	Group096	8,73	11,00
GO:0061009	common bile duct development	GO_BiologicalProcess-EBI-UniProt-GOA-ACAP-ARAP_08.05.2020_00h00	3,4908E-05	0,000502063	0,00184444	0,0038018	[4, 5, 6]	Group096	66,67	4,00
GO:2000737	negative regulation of stem cell differentiation	GO_BiologicalProcess-EBI-UniProt-GOA-ACAP-ARAP_08.05.2020_00h00	0,00222151	0,015300271	0,00184444	0,0038018	[4, 5, 6]	Group096	20,83	5,00
GO:0003279	cardiac septum development	GO_BiologicalProcess-EBI-UniProt-GOA-ACAP-ARAP_08.05.2020_00h00	0,00886469	0,03726219	0,00184444	0,0038018	[3, 4, 6, 7, 8]	Group096	9,09	11,00
GO:0061005	cell differentiation involved in kidney development	GO_BiologicalProcess-EBI-UniProt-GOA-ACAP-ARAP_08.05.2020_00h00	0,01226081	0,04496653	0,00184444	0,0038018	[4, 5, 6, 7, 8]	Group096	11,11	7,00
GO:0072049	comma-shaped body morphogenesis	GO_BiologicalProcess-EBI-UniProt-GOA-ACAP-ARAP_08.05.2020_00h00	0,00194417	0,014049736	0,00184444	0,0038018	[3, 4, 5, 6, 7, 8, 9, 10]	Group096	42,86	3,00
GO:0072050	S-shaped body morphogenesis	GO_BiologicalProcess-EBI-UniProt-GOA-ACAP-ARAP_08.05.2020_00h00	0,00609474	0,029314715	0,00184444	0,0038018	[3, 4, 5, 6, 7, 8, 9, 10]	Group096	30,00	3,00
GO:0072073	kidney epithelium development	GO_BiologicalProcess-EBI-UniProt-GOA-ACAP-ARAP_08.05.2020_00h00	0,01053228	0,041092683	0,00184444	0,0038018	[5, 6, 7, 8]	Group096	8,23	13,00
GO:0072080	nephron tubule development	GO_BiologicalProcess-EBI-UniProt-GOA-ACAP-ARAP_08.05.2020_00h00	0,00224538	0,015179563	0,00184444	0,0038018	[5, 6, 7, 8, 9, 10]	Group096	10,89	11,00
GO:0001657	ureteric bud development	GO_BiologicalProcess-EBI-UniProt-GOA-ACAP-ARAP_08.05.2020_00h00	0,00437197	0,023237992	0,00184444	0,0038018	[5, 6, 8, 9, 10, 11]	Group096	10,00	11,00

GO:0060675	ureteric bud morphogenesis	GO_BiologicalProcess-EBI-UniProt-GOA-ACAP-ARAP_08.05.2020_00h00	0,00194483	0,013985601	0,00184444	0,0038018	[6, 7, 8, 9, 10, 11, 12, 13]	Group096	12,68	9,00
GO:0030291	protein serine/threonine kinase inhibitor activity	GO_BiologicalProcess-EBI-UniProt-GOA-ACAP-ARAP_08.05.2020_00h00	0,00021557	0,002490113	0,00181235	0,00381349	[8, 9, 10, 11, 12]	Group099	21,88	7,00
GO:0044092	negative regulation of molecular function	GO_BiologicalProcess-EBI-UniProt-GOA-ACAP-ARAP_08.05.2020_00h00	0,00552191	0,027553212	0,00181235	0,00381349	[3]	Group099	5,43	68,00
GO:0051348	negative regulation of transferase activity	GO_BiologicalProcess-EBI-UniProt-GOA-ACAP-ARAP_08.05.2020_00h00	0,00220605	0,015337819	0,00181235	0,00381349	[5]	Group099	7,64	23,00
GO:0019220	regulation of phosphate metabolic process	GO_BiologicalProcess-EBI-UniProt-GOA-ACAP-ARAP_08.05.2020_00h00	0,00670985	0,031051561	0,00181235	0,00381349	[5, 6]	Group099	5,08	98,00
GO:0031399	regulation of protein modification process	GO_BiologicalProcess-EBI-UniProt-GOA-ACAP-ARAP_08.05.2020_00h00	0,00122922	0,009747351	0,00181235	0,00381349	[5, 6, 7]	Group099	5,31	105,00
GO:0045936	negative regulation of phosphate metabolic process	GO_BiologicalProcess-EBI-UniProt-GOA-ACAP-ARAP_08.05.2020_00h00	0,00908706	0,037979245	0,00181235	0,00381349	[5, 6, 7]	Group099	6,02	36,00
GO:0010719	negative regulation of epithelial to mesenchymal transition	GO_BiologicalProcess-EBI-UniProt-GOA-ACAP-ARAP_08.05.2020_00h00	0,01184689	0,044110134	0,00181235	0,00381349	[3, 4, 5, 6, 7, 8, 9, 10]	Group099	14,29	5,00
GO:0031400	negative regulation of protein modification process	GO_BiologicalProcess-EBI-UniProt-GOA-ACAP-ARAP_08.05.2020_00h00	0,00745149	0,03282683	0,00181235	0,00381349	[5, 6, 7, 8]	Group099	6,02	38,00
GO:0042325	regulation of phosphorylation	GO_BiologicalProcess-EBI-UniProt-GOA-ACAP-ARAP_08.05.2020_00h00	0,0058829	0,028960451	0,00181235	0,00381349	[6, 7]	Group099	5,17	89,00
GO:0001932	regulation of protein phosphorylation	GO_BiologicalProcess-EBI-UniProt-GOA-ACAP-ARAP_08.05.2020_00h00	0,01202917	0,044227567	0,00181235	0,00381349	[6, 7, 8]	Group099	5,12	79,00
GO:0033673	negative regulation of kinase activity	GO_BiologicalProcess-EBI-UniProt-GOA-ACAP-ARAP_08.05.2020_00h00	0,00238547	0,0159794	0,00181235	0,00381349	[6, 7, 8, 9]	Group099	7,87	21,00
GO:1902747	negative regulation of lens fiber cell differentiation	GO_BiologicalProcess-EBI-UniProt-GOA-ACAP-ARAP_08.05.2020_00h00	0,00114476	0,009434626	0,00181235	0,00381349	[3, 4, 5, 6, 7, 8, 9, 10, 11, 12]	Group099	50,00	3,00
GO:0019887	protein kinase regulator activity	GO_BiologicalProcess-EBI-UniProt-GOA-ACAP-ARAP_08.05.2020_00h00	0,00666023	0,031017654	0,00181235	0,00381349	[6, 7, 8, 9, 10]	Group099	7,96	16,00
GO:0006469	negative regulation of protein kinase activity	GO_BiologicalProcess-EBI-UniProt-GOA-ACAP-ARAP_08.05.2020_00h00	0,0043703	0,023313547	0,00181235	0,00381349	[7, 8, 9, 10]	Group099	7,76	19,00
GO:0004860	protein kinase inhibitor activity	GO_BiologicalProcess-EBI-UniProt-GOA-ACAP-ARAP_08.05.2020_00h00	0,00026167	0,002908152	0,00181235	0,00381349	[7, 8, 9, 10, 11]	Group099	15,15	10,00
GO:0050732	negative regulation of peptidyl-tyrosine phosphorylation	GO_BiologicalProcess-EBI-UniProt-GOA-ACAP-ARAP_08.05.2020_00h00	0,0059004	0,028949445	0,00181235	0,00381349	[7, 8, 9, 10, 11]	Group099	12,73	7,00
GO:0071901	negative regulation of protein serine/threonine kinase activity	GO_BiologicalProcess-EBI-UniProt-GOA-ACAP-ARAP_08.05.2020_00h00	0,00466629	0,024188856	0,00181235	0,00381349	[8, 9, 10, 11]	Group099	9,09	13,00
GO:0031490	chromatin DNA binding	GO_MolecularFunction-EBI-UniProt-GOA-ACAP-ARAP_08.05.2020_00h00	0,00224538	0,015179563	0,00224538	0,00444672	[3, 5]	Group021	10,89	11,00
GO:0005975	carbohydrate metabolic process	GO_BiologicalProcess-EBI-UniProt-GOA-ACAP-ARAP_08.05.2020_00h00	0,00648131	0,03067125	0,0022231	0,00449067	[3]	Group076	5,97	41,00
GO:0042180	cellular ketone metabolic process	GO_BiologicalProcess-EBI-UniProt-GOA-ACAP-ARAP_08.05.2020_00h00	0,01322385	0,047315594	0,0022231	0,00449067	[3]	Group076	6,96	19,00
GO:0044262	cellular carbohydrate metabolic process	GO_BiologicalProcess-EBI-UniProt-GOA-ACAP-ARAP_08.05.2020_00h00	0,01292284	0,046465218	0,0022231	0,00449067	[3, 4]	Group076	6,67	22,00
GO:0062012	regulation of small molecule metabolic process	GO_BiologicalProcess-EBI-UniProt-GOA-ACAP-ARAP_08.05.2020_00h00	0,00980614	0,039739247	0,0022231	0,00449067	[3, 4]	Group076	6,26	30,00

GO:0042181	ketone biosynthetic process	GO_BiologicalProcess-EBI-UniProt-GOA-ACAP-ARAP_08.05.2020_00h00	0,00240326	0,016025348	0,0022231	0,00449067	[4]	Group076	14,89	7,00
GO:0010565	regulation of cellular ketone metabolic process	GO_BiologicalProcess-EBI-UniProt-GOA-ACAP-ARAP_08.05.2020_00h00	0,01186175	0,04405364	0,0022231	0,00449067	[4, 5]	Group076	7,65	15,00
GO:0010566	regulation of ketone biosynthetic process	GO_BiologicalProcess-EBI-UniProt-GOA-ACAP-ARAP_08.05.2020_00h00	0,00390163	0,021763094	0,0022231	0,00449067	[5, 6]	Group076	23,53	4,00
GO:0071560	cellular response to transforming growth factor beta stimulus	GO_BiologicalProcess-EBI-UniProt-GOA-ACAP-ARAP_08.05.2020_00h00	0,00245953	0,016326362	0,00255268	0,00495808	[4, 6]	Group047	8,00	20,00
GO:0007178	transmembrane receptor protein serine/threonine kinase signaling pathway	GO_BiologicalProcess-EBI-UniProt-GOA-ACAP-ARAP_08.05.2020_00h00	0,00741431	0,032761414	0,00255268	0,00495808	[5, 6, 7]	Group047	6,74	25,00
GO:0007179	transforming growth factor beta receptor signaling pathway	GO_BiologicalProcess-EBI-UniProt-GOA-ACAP-ARAP_08.05.2020_00h00	0,00606435	0,029458281	0,00255268	0,00495808	[5, 6, 7, 8]	Group047	8,04	16,00
GO:0043094	cellular metabolic compound salvage	GO_BiologicalProcess-EBI-UniProt-GOA-ACAP-ARAP_08.05.2020_00h00	0,00272172	0,01735981	0,00272172	0,00518666	[3]	Group042	16,67	6,00
GO:0043097	pyrimidine nucleoside salvage	GO_BiologicalProcess-EBI-UniProt-GOA-ACAP-ARAP_08.05.2020_00h00	0,01052948	0,041191343	0,00272172	0,00518666	[5, 6, 7, 8, 9]	Group042	25,00	3,00
GO:0042127	regulation of cell population proliferation	GO_BiologicalProcess-EBI-UniProt-GOA-ACAP-ARAP_08.05.2020_00h00	0,00279691	0,017609714	0,00279691	0,00523125	[3, 4]	Group041	5,28	93,00
GO:0008285	negative regulation of cell population proliferation	GO_BiologicalProcess-EBI-UniProt-GOA-ACAP-ARAP_08.05.2020_00h00	0,00047703	0,005034603	0,00279691	0,00523125	[3, 4, 5]	Group041	6,52	49,00
GO:0001221	transcription cofactor binding	GO_MolecularFunction-EBI-UniProt-GOA-ACAP-ARAP_08.05.2020_00h00	0,0030653	0,018505316	0,0030653	0,00543149	[4]	Group038	14,29	7,00
GO:0001222	transcription corepressor binding	GO_MolecularFunction-EBI-UniProt-GOA-ACAP-ARAP_08.05.2020_00h00	0,00133056	0,010382594	0,0030653	0,00543149	[5]	Group038	30,77	4,00
GO:0010632	regulation of epithelial cell migration	GO_BiologicalProcess-EBI-UniProt-GOA-ACAP-ARAP_08.05.2020_00h00	0,00137465	0,010558186	0,00319385	0,00546743	[3, 4, 5, 6, 7]	Group060	8,40	20,00
GO:0010594	regulation of endothelial cell migration	GO_BiologicalProcess-EBI-UniProt-GOA-ACAP-ARAP_08.05.2020_00h00	0,00980666	0,039631858	0,00319385	0,00546743	[4, 5, 6, 7, 8]	Group060	8,05	14,00
GO:0001570	vasculogenesis	GO_BiologicalProcess-EBI-UniProt-GOA-ACAP-ARAP_08.05.2020_00h00	0,00884597	0,037290338	0,00319385	0,00546743	[4, 5, 6, 7, 8, 9, 10]	Group060	10,11	9,00
GO:0048845	venous blood vessel morphogenesis	GO_BiologicalProcess-EBI-UniProt-GOA-ACAP-ARAP_08.05.2020_00h00	0,00439531	0,02319398	0,00319385	0,00546743	[5, 6, 7, 8, 9, 10]	Group060	33,33	3,00
GO:0019904	protein domain specific binding	GO_MolecularFunction-EBI-UniProt-GOA-ACAP-ARAP_08.05.2020_00h00	0,0030593	0,018545408	0,0030593	0,00551766	[3]	Group020	6,06	46,00
GO:0075713	establishment of integrated proviral latency	GO_BiologicalProcess-EBI-UniProt-GOA-ACAP-ARAP_08.05.2020_00h00	0,003019	0,018608693	0,003019	0,00554398	[5, 6]	Group006	37,50	3,00
GO:0006114	glycerol biosynthetic process	GO_BiologicalProcess-EBI-UniProt-GOA-ACAP-ARAP_08.05.2020_00h00	0,003019	0,018608693	0,003019	0,00554398	[6, 7, 8]	Group007	37,50	3,00
GO:0051188	cofactor biosynthetic process	GO_BiologicalProcess-EBI-UniProt-GOA-ACAP-ARAP_08.05.2020_00h00	0,00318782	0,019087896	0,00318782	0,00555121	[4]	Group001	7,98	19,00
GO:0042181	ketone biosynthetic process	GO_BiologicalProcess-EBI-UniProt-GOA-ACAP-ARAP_08.05.2020_00h00	0,00240326	0,016025348	0,00376698	0,00634108	[4]	Group043	14,89	7,00
GO:0010565	regulation of cellular ketone metabolic process	GO_BiologicalProcess-EBI-UniProt-GOA-ACAP-ARAP_08.05.2020_00h00	0,01186175	0,04405364	0,00376698	0,00634108	[4, 5]	Group043	7,65	15,00
GO:0010566	regulation of ketone biosynthetic process	GO_BiologicalProcess-EBI-UniProt-GOA-ACAP-ARAP_08.05.2020_00h00	0,00390163	0,021763094	0,00376698	0,00634108	[5, 6]	Group043	23,53	4,00
GO:0019211	phosphatase activator activity	GO_BiologicalProcess-EBI-UniProt-GOA-ACAP-ARAP_08.05.2020_00h00	0,00390163	0,021763094	0,00390163	0,00646008	[6, 8]	Group019	23,53	4,00

GO:0042809	vitamin D receptor binding	GO_MolecularFunction-EBI-UniProt-GOA-ACAP-ARAP_08.05.2020_00h00	0,00390163	0,021763094	0,00390163	0,00646008	[6, 7]	Group030	23,53	4,00
GO:0050839	cell adhesion molecule binding	GO_MolecularFunction-EBI-UniProt-GOA-ACAP-ARAP_08.05.2020_00h00	0,00412503	0,022329962	0,00412503	0,00661314	[3]	Group032	6,39	35,00
GO:0045296	cadherin binding	GO_MolecularFunction-EBI-UniProt-GOA-ACAP-ARAP_08.05.2020_00h00	0,00384715	0,021623628	0,00412503	0,00661314	[4]	Group032	7,10	25,00
GO:0030522	intracellular receptor signaling pathway	GO_BiologicalProcess-EBI-UniProt-GOA-ACAP-ARAP_08.05.2020_00h00	0,00076096	0,007296289	0,00411884	0,00670972	[3, 4, 5]	Group073	8,14	24,00
GO:0071310	cellular response to organic substance	GO_BiologicalProcess-EBI-UniProt-GOA-ACAP-ARAP_08.05.2020_00h00	0,00732946	0,032582801	0,00411884	0,00670972	[4]	Group073	4,83	140,00
GO:0009755	hormone-mediated signaling pathway	GO_BiologicalProcess-EBI-UniProt-GOA-ACAP-ARAP_08.05.2020_00h00	0,01050162	0,041192181	0,00411884	0,00670972	[3, 4, 5, 6]	Group073	7,26	18,00
GO:0032870	cellular response to hormone stimulus	GO_BiologicalProcess-EBI-UniProt-GOA-ACAP-ARAP_08.05.2020_00h00	0,00399949	0,021974724	0,00411884	0,00670972	[4, 5]	Group073	5,97	46,00
GO:0071383	cellular response to steroid hormone stimulus	GO_BiologicalProcess-EBI-UniProt-GOA-ACAP-ARAP_08.05.2020_00h00	0,00606695	0,029373631	0,00411884	0,00670972	[5, 6]	Group073	7,38	20,00
GO:0030518	intracellular steroid hormone receptor signaling pathway	GO_BiologicalProcess-EBI-UniProt-GOA-ACAP-ARAP_08.05.2020_00h00	0,00366435	0,021080799	0,00411884	0,00670972	[4, 5, 6, 7, 8]	Group073	9,35	13,00
GO:0042802	identical protein binding	GO_MolecularFunction-EBI-UniProt-GOA-ACAP-ARAP_08.05.2020_00h00	0,00426695	0,023013318	0,00426695	0,00673379	[3]	Group027	5,14	100,00
GO:0070898	RNA polymerase III preinitiation complex assembly	GO_BiologicalProcess-EBI-UniProt-GOA-ACAP-ARAP_08.05.2020_00h00	0,003019	0,018608693	0,00447734	0,00674942	[8, 9, 10, 11, 12]	Group046	37,50	3,00
GO:0006383	transcription by RNA polymerase III	GO_BiologicalProcess-EBI-UniProt-GOA-ACAP-ARAP_08.05.2020_00h00	0,00532789	0,026767179	0,00447734	0,00674942	[6, 7, 8, 9, 10]	Group046	12,96	7,00
GO:0070897	transcription preinitiation complex assembly	GO_BiologicalProcess-EBI-UniProt-GOA-ACAP-ARAP_08.05.2020_00h00	0,01265239	0,045829773	0,00447734	0,00674942	[7, 8, 9, 10, 11]	Group046	12,24	6,00
GO:0060485	mesenchyme development	GO_BiologicalProcess-EBI-UniProt-GOA-ACAP-ARAP_08.05.2020_00h00	0,00693329	0,031685803	0,00445098	0,00681136	[4, 5, 6]	Group077	7,17	21,00
GO:2000048	negative regulation of cell-cell adhesion mediated by cadherin	GO_BiologicalProcess-EBI-UniProt-GOA-ACAP-ARAP_08.05.2020_00h00	0,00609474	0,029314715	0,00445098	0,00681136	[5, 6, 7]	Group077	30,00	3,00
GO:0001837	epithelial to mesenchymal transition	GO_BiologicalProcess-EBI-UniProt-GOA-ACAP-ARAP_08.05.2020_00h00	7,1331E-05	0,000934306	0,00445098	0,00681136	[5, 6, 7, 8]	Group077	11,64	17,00
GO:0010717	regulation of epithelial to mesenchymal transition	GO_BiologicalProcess-EBI-UniProt-GOA-ACAP-ARAP_08.05.2020_00h00	0,0010473	0,008984704	0,00445098	0,00681136	[4, 5, 6, 7, 8, 9]	Group077	11,96	11,00
GO:0010718	positive regulation of epithelial to mesenchymal transition	GO_BiologicalProcess-EBI-UniProt-GOA-ACAP-ARAP_08.05.2020_00h00	0,01147846	0,043511376	0,00445098	0,00681136	[3, 4, 5, 6, 7, 8, 9, 10]	Group077	12,50	6,00
GO:0010719	negative regulation of epithelial to mesenchymal transition	GO_BiologicalProcess-EBI-UniProt-GOA-ACAP-ARAP_08.05.2020_00h00	0,01184689	0,044110134	0,00445098	0,00681136	[3, 4, 5, 6, 7, 8, 9, 10]	Group077	14,29	5,00
GO:1902747	negative regulation of lens fiber cell differentiation	GO_BiologicalProcess-EBI-UniProt-GOA-ACAP-ARAP_08.05.2020_00h00	0,00114476	0,009434626	0,00445098	0,00681136	[3, 4, 5, 6, 7, 8, 9, 10, 11, 12]	Group077	50,00	3,00
GO:0051020	GTPase binding	GO_MolecularFunction-EBI-UniProt-GOA-ACAP-ARAP_08.05.2020_00h00	0,01147669	0,043617357	0,00444462	0,00690626	[4]	Group050	5,96	35,00
GO:0007264	small GTPase mediated signal transduction	GO_BiologicalProcess-EBI-UniProt-GOA-ACAP-ARAP_08.05.2020_00h00	0,00349706	0,020277437	0,00444462	0,00690626	[4, 5, 6]	Group050	6,31	38,00
GO:0051056	regulation of small GTPase mediated signal transduction	GO_BiologicalProcess-EBI-UniProt-GOA-ACAP-ARAP_08.05.2020_00h00	0,00675635	0,031168437	0,00444462	0,00690626	[5, 6, 7]	Group050	6,88	24,00

GO:2001267	regulation of cysteine-type endopeptidase activity involved in apoptotic signaling pathway	GO_BiologicalProcess-EBI-UniProt-GOA-ACAP-ARAP_08.05.2020_00h00	0,00486045	0,024931068	0,00486045	0,00701294	[5, 6, 7, 8, 9, 10, 11, 12]	Group009	22,22	4,00
GO:1903320	regulation of protein modification by small protein conjugation or removal	GO_BiologicalProcess-EBI-UniProt-GOA-ACAP-ARAP_08.05.2020_00h00	0,00497767	0,025354998	0,00472986	0,00702524	[6, 7, 8]	Group035	7,66	19,00
GO:0032446	protein modification by small protein conjugation	GO_BiologicalProcess-EBI-UniProt-GOA-ACAP-ARAP_08.05.2020_00h00	0,00658032	0,030743105	0,00472986	0,00702524	[7, 8]	Group035	5,60	55,00
GO:0048729	tissue morphogenesis	GO_BiologicalProcess-EBI-UniProt-GOA-ACAP-ARAP_08.05.2020_00h00	0,01362663	0,048285682	0,00485452	0,0071059	[3, 4]	Group097	5,69	41,00
GO:0001655	urogenital system development	GO_BiologicalProcess-EBI-UniProt-GOA-ACAP-ARAP_08.05.2020_00h00	0,01173816	0,044267047	0,00485452	0,0071059	[4, 5]	Group097	6,56	24,00
GO:0060541	respiratory system development	GO_BiologicalProcess-EBI-UniProt-GOA-ACAP-ARAP_08.05.2020_00h00	0,00734388	0,032548264	0,00485452	0,0071059	[4, 5]	Group097	7,69	17,00
GO:0061138	morphogenesis of a branching epithelium	GO_BiologicalProcess-EBI-UniProt-GOA-ACAP-ARAP_08.05.2020_00h00	0,00284655	0,017769769	0,00485452	0,0071059	[3, 4, 5, 6]	Group097	8,46	17,00
GO:0051145	smooth muscle cell differentiation	GO_BiologicalProcess-EBI-UniProt-GOA-ACAP-ARAP_08.05.2020_00h00	0,01034567	0,040908623	0,00485452	0,0071059	[5]	Group097	11,48	7,00
GO:0060485	mesenchyme development	GO_BiologicalProcess-EBI-UniProt-GOA-ACAP-ARAP_08.05.2020_00h00	0,00693329	0,031685803	0,00485452	0,0071059	[4, 5, 6]	Group097	7,17	21,00
GO:0060562	epithelial tube morphogenesis	GO_BiologicalProcess-EBI-UniProt-GOA-ACAP-ARAP_08.05.2020_00h00	0,00102574	0,009010539	0,00485452	0,0071059	[4, 5, 6]	Group097	7,61	27,00
GO:0030857	negative regulation of epithelial cell differentiation	GO_BiologicalProcess-EBI-UniProt-GOA-ACAP-ARAP_08.05.2020_00h00	2,2595E-05	0,000338235	0,00485452	0,0071059	[4, 5, 6, 7]	Group097	20,00	10,00
GO:0048754	branching morphogenesis of an epithelial tube	GO_BiologicalProcess-EBI-UniProt-GOA-ACAP-ARAP_08.05.2020_00h00	0,00247505	0,016355416	0,00485452	0,0071059	[4, 5, 6, 7]	Group097	9,09	15,00
GO:0061323	cell proliferation involved in heart morphogenesis	GO_BiologicalProcess-EBI-UniProt-GOA-ACAP-ARAP_08.05.2020_00h00	0,00092442	0,008475746	0,00485452	0,0071059	[3, 5, 6, 7, 8]	Group097	25,00	5,00
GO:0035886	vascular smooth muscle cell differentiation	GO_BiologicalProcess-EBI-UniProt-GOA-ACAP-ARAP_08.05.2020_00h00	0,00450535	0,023437398	0,00485452	0,0071059	[5, 6, 7, 8]	Group097	17,86	5,00
GO:2000826	regulation of heart morphogenesis	GO_BiologicalProcess-EBI-UniProt-GOA-ACAP-ARAP_08.05.2020_00h00	0,00754438	0,033136562	0,00485452	0,0071059	[5, 6, 7, 8]	Group097	13,64	6,00
GO:0072080	nephron tubule development	GO_BiologicalProcess-EBI-UniProt-GOA-ACAP-ARAP_08.05.2020_00h00	0,00224538	0,015179563	0,00485452	0,0071059	[5, 6, 7, 8, 9, 10]	Group097	10,89	11,00
GO:0035904	aorta development	GO_BiologicalProcess-EBI-UniProt-GOA-ACAP-ARAP_08.05.2020_00h00	0,00789435	0,03457018	0,00485452	0,0071059	[5, 7, 8, 9, 10]	Group097	12,07	7,00
GO:0035909	aorta morphogenesis	GO_BiologicalProcess-EBI-UniProt-GOA-ACAP-ARAP_08.05.2020_00h00	0,00810163	0,03516297	0,00485452	0,0071059	[6, 7, 8, 9, 10, 11]	Group097	15,63	5,00
GO:0051640	organelle localization	GO_BiologicalProcess-EBI-UniProt-GOA-ACAP-ARAP_08.05.2020_00h00	0,00632761	0,03013834	0,00511219	0,00727228	[3]	Group079	5,98	41,00
GO:0006903	vesicle targeting	GO_BiologicalProcess-EBI-UniProt-GOA-ACAP-ARAP_08.05.2020_00h00	0,00224538	0,015179563	0,00511219	0,00727228	[2, 3, 5, 6]	Group079	10,89	11,00
GO:0006900	vesicle budding from membrane	GO_BiologicalProcess-EBI-UniProt-GOA-ACAP-ARAP_08.05.2020_00h00	0,00224419	0,015241823	0,00511219	0,00727228	[3, 5, 6]	Group079	10,34	12,00
GO:0016050	vesicle organization	GO_BiologicalProcess-EBI-UniProt-GOA-ACAP-ARAP_08.05.2020_00h00	0,00398962	0,022002891	0,00511219	0,00727228	[5]	Group079	7,08	25,00
GO:0006901	vesicle coating	GO_BiologicalProcess-EBI-UniProt-GOA-ACAP-ARAP_08.05.2020_00h00	0,00074598	0,007199673	0,00511219	0,00727228	[4, 6, 7]	Group079	13,33	10,00
GO:0048194	Golgi vesicle budding	GO_BiologicalProcess-EBI-UniProt-GOA-ACAP-ARAP_08.05.2020_00h00	0,00197443	0,014060638	0,00511219	0,00727228	[4, 6, 7]	Group079	11,76	10,00

GO:0048208	COPII vesicle coating	GO_BiologicalProcess-EBI-UniProt-GOA-ACAP-ARAP_08.05.2020_00h00	0,00646385	0,030687595	0,00511219	0,00727228	[5, 6, 7, 8, 9]	Group079	11,43	8,00
GO:0009790	embryo development	GO_BiologicalProcess-EBI-UniProt-GOA-ACAP-ARAP_08.05.2020_00h00	0,00450283	0,023507655	0,00518904	0,00727906	[3, 4]	Group083	5,56	63,00
GO:0035295	tube development	GO_BiologicalProcess-EBI-UniProt-GOA-ACAP-ARAP_08.05.2020_00h00	0,00965585	0,039567392	0,00518904	0,00727906	[3, 4]	Group083	5,39	61,00
GO:0048729	tissue morphogenesis	GO_BiologicalProcess-EBI-UniProt-GOA-ACAP-ARAP_08.05.2020_00h00	0,01362663	0,048285682	0,00518904	0,00727906	[3, 4]	Group083	5,69	41,00
GO:0035239	tube morphogenesis	GO_BiologicalProcess-EBI-UniProt-GOA-ACAP-ARAP_08.05.2020_00h00	0,00081955	0,007756677	0,00518904	0,00727906	[3, 4, 5]	Group083	6,15	56,00
GO:0009792	embryo development ending in birth or egg hatching	GO_BiologicalProcess-EBI-UniProt-GOA-ACAP-ARAP_08.05.2020_00h00	0,00035885	0,003870864	0,00518904	0,00727906	[4, 5]	Group083	6,65	48,00
GO:0072359	circulatory system development	GO_BiologicalProcess-EBI-UniProt-GOA-ACAP-ARAP_08.05.2020_00h00	0,00149302	0,01123211	0,00518904	0,00727906	[4, 5]	Group083	5,75	67,00
GO:0060562	epithelial tube morphogenesis	GO_BiologicalProcess-EBI-UniProt-GOA-ACAP-ARAP_08.05.2020_00h00	0,00102574	0,009010539	0,00518904	0,00727906	[4, 5, 6]	Group083	7,61	27,00
GO:0001701	in utero embryonic development	GO_BiologicalProcess-EBI-UniProt-GOA-ACAP-ARAP_08.05.2020_00h00	0,00080389	0,007657848	0,00518904	0,00727906	[6, 7]	Group083	7,35	31,00
GO:0012501	programmed cell death	GO_BiologicalProcess-EBI-UniProt-GOA-ACAP-ARAP_08.05.2020_00h00	0,00669526	0,031082088	0,00528282	0,00730911	[3]	Group087	4,99	110,00
GO:0023056	positive regulation of signaling	GO_BiologicalProcess-EBI-UniProt-GOA-ACAP-ARAP_08.05.2020_00h00	0,00616386	0,029550245	0,00528282	0,00730911	[2, 3, 4]	Group087	5,05	103,00
GO:0010941	regulation of cell death	GO_BiologicalProcess-EBI-UniProt-GOA-ACAP-ARAP_08.05.2020_00h00	0,00652066	0,03065965	0,00528282	0,00730911	[3, 4]	Group087	5,13	92,00
GO:0006915	apoptotic process	GO_BiologicalProcess-EBI-UniProt-GOA-ACAP-ARAP_08.05.2020_00h00	0,0021518	0,015103772	0,00528282	0,00730911	[4]	Group087	5,20	107,00
GO:0010647	positive regulation of cell communication	GO_BiologicalProcess-EBI-UniProt-GOA-ACAP-ARAP_08.05.2020_00h00	0,00550487	0,027561951	0,00528282	0,00730911	[3, 4, 5]	Group087	5,07	103,00
GO:0010942	positive regulation of cell death	GO_BiologicalProcess-EBI-UniProt-GOA-ACAP-ARAP_08.05.2020_00h00	0,00325328	0,0192442	0,00528282	0,00730911	[3, 4, 5]	Group087	6,07	45,00
GO:0009967	positive regulation of signal transduction	GO_BiologicalProcess-EBI-UniProt-GOA-ACAP-ARAP_08.05.2020_00h00	0,00303475	0,018472932	0,00528282	0,00730911	[3, 4, 5, 6]	Group087	5,26	93,00
GO:0042981	regulation of apoptotic process	GO_BiologicalProcess-EBI-UniProt-GOA-ACAP-ARAP_08.05.2020_00h00	0,00142297	0,010872362	0,00528282	0,00730911	[5, 6]	Group087	5,44	89,00
GO:0043065	positive regulation of apoptotic process	GO_BiologicalProcess-EBI-UniProt-GOA-ACAP-ARAP_08.05.2020_00h00	0,00326111	0,019213051	0,00528282	0,00730911	[5, 6, 7]	Group087	6,18	42,00
GO:2001235	positive regulation of apoptotic signaling pathway	GO_BiologicalProcess-EBI-UniProt-GOA-ACAP-ARAP_08.05.2020_00h00	0,00104054	0,009032417	0,00528282	0,00730911	[4, 5, 6, 7, 8]	Group087	9,29	17,00
GO:0036498	IRE1-mediated unfolded protein response	GO_BiologicalProcess-EBI-UniProt-GOA-ACAP-ARAP_08.05.2020_00h00	0,00592616	0,028978905	0,00592616	0,0080884	[4, 5, 6, 7, 8]	Group025	11,59	8,00
GO:0035646	endosome to melanosome transport	GO_BiologicalProcess-EBI-UniProt-GOA-ACAP-ARAP_08.05.2020_00h00	0,00609474	0,029314715	0,00609474	0,00820759	[5, 6, 7]	Group024	30,00	3,00
GO:0001522	pseudouridine synthesis	GO_BiologicalProcess-EBI-UniProt-GOA-ACAP-ARAP_08.05.2020_00h00	0,00722575	0,032317616	0,00722575	0,00947793	[6, 7, 8]	Group028	20,00	4,00
GO:0005080	protein kinase C binding	GO_MolecularFunction-EBI-UniProt-GOA-ACAP-ARAP_08.05.2020_00h00	0,00718159	0,032317147	0,00718159	0,00954395	[6]	Group005	12,28	7,00
GO:0051145	smooth muscle cell differentiation	GO_BiologicalProcess-EBI-UniProt-GOA-ACAP-ARAP_08.05.2020_00h00	0,01034567	0,040908623	0,00794445	0,01028705	[5]	Group069	11,48	7,00

GO:0035886	vascular smooth muscle cell differentiation	GO_BiologicalProcess-EBI-UniProt-GOA-ACAP-ARAP_08.05.2020_00h00	0,00450535	0,023437398	0,00794445	0,01028705	[5, 6, 7, 8]	Group069	17,86	5,00
GO:0060976	coronary vasculature development	GO_BiologicalProcess-EBI-UniProt-GOA-ACAP-ARAP_08.05.2020_00h00	0,00344449	0,020051828	0,00794445	0,01028705	[4, 5, 6, 7, 8, 9]	Group069	14,00	7,00
GO:0002040	sprouting angiogenesis	GO_BiologicalProcess-EBI-UniProt-GOA-ACAP-ARAP_08.05.2020_00h00	0,00390121	0,021843806	0,00794445	0,01028705	[4, 5, 6, 7, 8, 9, 10, 11]	Group069	9,68	12,00
GO:0060947	cardiac vascular smooth muscle cell differentiation	GO_BiologicalProcess-EBI-UniProt-GOA-ACAP-ARAP_08.05.2020_00h00	0,003019	0,018608693	0,00794445	0,01028705	[5, 6, 7, 8, 9, 10]	Group069	37,50	3,00
GO:0035904	aorta development	GO_BiologicalProcess-EBI-UniProt-GOA-ACAP-ARAP_08.05.2020_00h00	0,00789435	0,03457018	0,00794445	0,01028705	[5, 7, 8, 9, 10]	Group069	12,07	7,00
GO:0034097	response to cytokine	GO_BiologicalProcess-EBI-UniProt-GOA-ACAP-ARAP_08.05.2020_00h00	0,01025411	0,04076634	0,00817803	0,01032476	[4]	Group058	5,29	67,00
GO:0071310	cellular response to organic substance	GO_BiologicalProcess-EBI-UniProt-GOA-ACAP-ARAP_08.05.2020_00h00	0,00732946	0,032582801	0,00817803	0,01032476	[4]	Group058	4,83	140,00
GO:0032870	cellular response to hormone stimulus	GO_BiologicalProcess-EBI-UniProt-GOA-ACAP-ARAP_08.05.2020_00h00	0,00399949	0,021974724	0,00817803	0,01032476	[4, 5]	Group058	5,97	46,00
GO:0071345	cellular response to cytokine stimulus	GO_BiologicalProcess-EBI-UniProt-GOA-ACAP-ARAP_08.05.2020_00h00	0,00954548	0,039224699	0,00817803	0,01032476	[5]	Group058	5,36	63,00
GO:0051098	regulation of binding	GO_BiologicalProcess-EBI-UniProt-GOA-ACAP-ARAP_08.05.2020_00h00	0,00830744	0,03584414	0,00830744	0,01035865	[3]	Group033	6,60	26,00
GO:0051101	regulation of DNA binding	GO_BiologicalProcess-EBI-UniProt-GOA-ACAP-ARAP_08.05.2020_00h00	0,00722218	0,032400433	0,00830744	0,01035865	[4]	Group033	8,96	12,00
GO:0007028	cytoplasm organization	GO_BiologicalProcess-EBI-UniProt-GOA-ACAP-ARAP_08.05.2020_00h00	0,00813485	0,035203002	0,00813485	0,01040024	[4]	Group012	27,27	3,00
GO:0070932	histone H3 deacetylation	GO_BiologicalProcess-EBI-UniProt-GOA-ACAP-ARAP_08.05.2020_00h00	0,00864971	0,036780071	0,00864971	0,01052555	[8, 9, 10]	Group004	19,05	4,00
GO:0010942	positive regulation of cell death	GO_BiologicalProcess-EBI-UniProt-GOA-ACAP-ARAP_08.05.2020_00h00	0,00325328	0,0192442	0,00856299	0,0105471	[3, 4, 5]	Group068	6,07	45,00
GO:0097192	extrinsic apoptotic signaling pathway in absence of ligand	GO_BiologicalProcess-EBI-UniProt-GOA-ACAP-ARAP_08.05.2020_00h00	0,01050082	0,04129947	0,00856299	0,0105471	[4, 5, 6, 7]	Group068	10,53	8,00
GO:0043065	positive regulation of apoptotic process	GO_BiologicalProcess-EBI-UniProt-GOA-ACAP-ARAP_08.05.2020_00h00	0,00326111	0,019213051	0,00856299	0,0105471	[5, 6, 7]	Group068	6,18	42,00
GO:2001235	positive regulation of apoptotic signaling pathway	GO_BiologicalProcess-EBI-UniProt-GOA-ACAP-ARAP_08.05.2020_00h00	0,00104054	0,009032417	0,00856299	0,0105471	[4, 5, 6, 7, 8]	Group068	9,29	17,00
GO:2001236	regulation of extrinsic apoptotic signaling pathway	GO_BiologicalProcess-EBI-UniProt-GOA-ACAP-ARAP_08.05.2020_00h00	0,01237392	0,045155589	0,00856299	0,0105471	[5, 6, 7, 8]	Group068	7,82	14,00
GO:2001238	positive regulation of extrinsic apoptotic signaling pathway	GO_BiologicalProcess-EBI-UniProt-GOA-ACAP-ARAP_08.05.2020_00h00	0,0059004	0,028949445	0,00856299	0,0105471	[5, 6, 7, 8, 9]	Group068	12,73	7,00
GO:0006364	rRNA processing	GO_BiologicalProcess-EBI-UniProt-GOA-ACAP-ARAP_08.05.2020_00h00	0,00908557	0,038081523	0,00908557	0,01092432	[6, 7, 8, 9]	Group011	7,52	17,00
GO:0006665	sphingolipid metabolic process	GO_BiologicalProcess-EBI-UniProt-GOA-ACAP-ARAP_08.05.2020_00h00	0,00934755	0,03873688	0,00934755	0,01110709	[4, 5, 6]	Group036	8,09	14,00
GO:0001573	ganglioside metabolic process	GO_BiologicalProcess-EBI-UniProt-GOA-ACAP-ARAP_08.05.2020_00h00	0,01024521	0,040841632	0,00934755	0,01110709	[6, 7, 8]	Group036	18,18	4,00
GO:0044248	cellular catabolic process	GO_BiologicalProcess-EBI-UniProt-GOA-ACAP-ARAP_08.05.2020_00h00	0,00978321	0,039756157	0,00962754	0,01130676	[3]	Group088	4,88	119,00

GO:1901575	organic substance catabolic process	GO_BiologicalProcess-EBI-UniProt-GOA-ACAP-ARAP_08.05.2020_00h00	0,00469808	0,024267906	0,00962754	0,01130676	[3]	Group088	5,00	118,00
GO:0009057	macromolecule catabolic process	GO_BiologicalProcess-EBI-UniProt-GOA-ACAP-ARAP_08.05.2020_00h00	0,00264444	0,017014867	0,00962754	0,01130676	[4]	Group088	5,42	81,00
GO:0019439	aromatic compound catabolic process	GO_BiologicalProcess-EBI-UniProt-GOA-ACAP-ARAP_08.05.2020_00h00	0,00429309	0,023069486	0,00962754	0,01130676	[4]	Group088	6,15	40,00
GO:0044270	cellular nitrogen compound catabolic process	GO_BiologicalProcess-EBI-UniProt-GOA-ACAP-ARAP_08.05.2020_00h00	0,00492534	0,025175845	0,00962754	0,01130676	[4]	Group088	6,14	39,00
GO:0046700	heterocycle catabolic process	GO_BiologicalProcess-EBI-UniProt-GOA-ACAP-ARAP_08.05.2020_00h00	0,00443854	0,023254775	0,00962754	0,01130676	[4]	Group088	6,18	39,00
GO:1901361	organic cyclic compound catabolic process	GO_BiologicalProcess-EBI-UniProt-GOA-ACAP-ARAP_08.05.2020_00h00	0,006177	0,029516801	0,00962754	0,01130676	[4]	Group088	5,99	41,00
GO:0034655	nucleobase-containing compound catabolic process	GO_BiologicalProcess-EBI-UniProt-GOA-ACAP-ARAP_08.05.2020_00h00	0,00303302	0,01853935	0,00962754	0,01130676	[4, 5]	Group088	6,41	37,00
GO:0044265	cellular macromolecule catabolic process	GO_BiologicalProcess-EBI-UniProt-GOA-ACAP-ARAP_08.05.2020_00h00	0,00126989	0,010015749	0,00962754	0,01130676	[4, 5]	Group088	5,72	71,00
GO:0006401	RNA catabolic process	GO_BiologicalProcess-EBI-UniProt-GOA-ACAP-ARAP_08.05.2020_00h00	0,01374993	0,048605185	0,00962754	0,01130676	[5, 6, 7]	Group088	6,26	27,00
GO:0009991	response to extracellular stimulus	GO_BiologicalProcess-EBI-UniProt-GOA-ACAP-ARAP_08.05.2020_00h00	0,01026558	0,040701646	0,01026558	0,01164971	[3]	Group023	5,97	36,00
GO:0039702	viral budding via host ESCRT complex	GO_BiologicalProcess-EBI-UniProt-GOA-ACAP-ARAP_08.05.2020_00h00	0,01024521	0,040841632	0,01024521	0,0117587	[5, 6, 7]	Group026	18,18	4,00
GO:0032088	negative regulation of NF-kappaB transcription factor activity	GO_BiologicalProcess-EBI-UniProt-GOA-ACAP-ARAP_08.05.2020_00h00	0,01017478	0,040782515	0,01017478	0,0118121	[3, 4, 5, 8, 9, 10, 11, 12, 13, 14]	Group022	9,89	9,00
GO:0051549	positive regulation of keratinocyte migration	GO_BiologicalProcess-EBI-UniProt-GOA-ACAP-ARAP_08.05.2020_00h00	0,01052948	0,041191343	0,01052948	0,01181642	[4, 5, 6, 7, 8, 9]	Group002	25,00	3,00
GO:0014029	neural crest formation	GO_BiologicalProcess-EBI-UniProt-GOA-ACAP-ARAP_08.05.2020_00h00	0,01052948	0,041191343	0,01052948	0,01181642	[3, 4, 5, 6, 7, 8, 9]	Group014	25,00	3,00
GO:0006091	generation of precursor metabolites and energy	GO_BiologicalProcess-EBI-UniProt-GOA-ACAP-ARAP_08.05.2020_00h00	0,01093451	0,042102177	0,01093451	0,01213611	[3]	Group037	5,98	35,00
GO:0015980	energy derivation by oxidation of organic compounds	GO_BiologicalProcess-EBI-UniProt-GOA-ACAP-ARAP_08.05.2020_00h00	0,00499962	0,025378708	0,01093451	0,01213611	[3, 4]	Group037	7,26	22,00
GO:0002366	leukocyte activation involved in immune response	GO_BiologicalProcess-EBI-UniProt-GOA-ACAP-ARAP_08.05.2020_00h00	0,01188678	0,044035126	0,01188678	0,0129093	[3, 4]	Group029	5,66	44,00
GO:0032011	ARF protein signal transduction	GO_BiologicalProcess-EBI-UniProt-GOA-ACAP-ARAP_08.05.2020_00h00	0,01201947	0,044302911	0,01201947	0,01291453	[6, 7, 8]	Group039	17,39	4,00
GO:0032012	regulation of ARF protein signal transduction	GO_BiologicalProcess-EBI-UniProt-GOA-ACAP-ARAP_08.05.2020_00h00	0,01201947	0,044302911	0,01201947	0,01291453	[7, 8, 9]	Group039	17,39	4,00
GO:0016055	Wnt signaling pathway	GO_BiologicalProcess-EBI-UniProt-GOA-ACAP-ARAP_08.05.2020_00h00	0,01180008	0,044386465	0,01180008	0,01295444	[4, 5, 6, 7]	Group017	6,03	33,00
GO:0007044	cell-substrate junction assembly	GO_BiologicalProcess-EBI-UniProt-GOA-ACAP-ARAP_08.05.2020_00h00	0,0122656	0,0448719	0,0122656	0,01304027	[6]	Group013	9,09	10,00
GO:0050662	coenzyme binding	GO_MolecularFunction-EBI-UniProt-GOA-ACAP-ARAP_08.05.2020_00h00	0,01251242	0,045547713	0,01251242	0,01316411	[3]	Group000	6,69	22,00
GO:0007599	hemostasis	GO_BiologicalProcess-EBI-UniProt-GOA-ACAP-ARAP_08.05.2020_00h00	0,01286614	0,046374995	0,01286614	0,0133967	[4]	Group015	6,50	24,00
GO:0017004	cytochrome complex assembly	GO_BiologicalProcess-EBI-UniProt-GOA-ACAP-ARAP_08.05.2020_00h00	0,0133187	0,04730878	0,0133187	0,01345188	[7]	Group018	13,89	5,00

GO:0006123	mitochondrial electron transport, cytochrome c to oxygen	GO_BiologicalProcess-EBI-UniProt-GOA-ACAP-ARAP_08.05.2020_00h00	0,00864971	0,036780071	0,0133187	0,01345188	[6, 7, 8, 9]	Group034	19,05	4,00
GO:0004129	cytochrome-c oxidase activity	GO_BiologicalProcess-EBI-UniProt-GOA-ACAP-ARAP_08.05.2020_00h00	0,01048958	0,041366164	0,0133187	0,01345188	[5, 6, 8, 9, 10, 11]	Group034	14,71	5,00
GO:0030515	snoRNA binding	GO_MolecularFunction-EBI-UniProt-GOA-ACAP-ARAP_08.05.2020_00h00	0,0133187	0,04730878	0,0133187	0,01345188	[5]	Group040	13,89	5,00
GO:0034511	U3 snoRNA binding	GO_MolecularFunction-EBI-UniProt-GOA-ACAP-ARAP_08.05.2020_00h00	0,00439531	0,02319398	0,0133187	0,01345188	[6]	Group040	33,33	3,00
GO:1902337	regulation of apoptotic process involved in morphogenesis	GO_BiologicalProcess-EBI-UniProt-GOA-ACAP-ARAP_08.05.2020_00h00	0,01328916	0,047433581	0,01328916	0,01355763	[4, 5, 7, 8]	Group008	23,08	3,00
GO:0042180	cellular ketone metabolic process	GO_BiologicalProcess-EBI-UniProt-GOA-ACAP-ARAP_08.05.2020_00h00	0,01322385	0,047315594	0,01322385	0,01362867	[3]	Group062	6,96	19,00
GO:0042181	ketone biosynthetic process	GO_BiologicalProcess-EBI-UniProt-GOA-ACAP-ARAP_08.05.2020_00h00	0,00240326	0,016025348	0,01322385	0,01362867	[4]	Group062	14,89	7,00
GO:0010566	regulation of ketone biosynthetic process	GO_BiologicalProcess-EBI-UniProt-GOA-ACAP-ARAP_08.05.2020_00h00	0,00390163	0,021763094	0,01322385	0,01362867	[5, 6]	Group062	23,53	4,00
GO:0006744	ubiquinone biosynthetic process	GO_BiologicalProcess-EBI-UniProt-GOA-ACAP-ARAP_08.05.2020_00h00	0,00390163	0,021763094	0,01322385	0,01362867	[6, 7]	Group062	23,53	4,00
GO:0030155	regulation of cell adhesion	GO_BiologicalProcess-EBI-UniProt-GOA-ACAP-ARAP_08.05.2020_00h00	0,0002975	0,003281432	0,02375092	0,02375092	[3, 4]	Group066	6,62	50,00
GO:0035295	tube development	GO_BiologicalProcess-EBI-UniProt-GOA-ACAP-ARAP_08.05.2020_00h00	0,00965585	0,039567392	0,02375092	0,02375092	[3, 4]	Group066	5,39	61,00
GO:0007162	negative regulation of cell adhesion	GO_BiologicalProcess-EBI-UniProt-GOA-ACAP-ARAP_08.05.2020_00h00	0,00803831	0,035095848	0,02375092	0,02375092	[3, 4, 5]	Group066	7,07	21,00
GO:0022407	regulation of cell-cell adhesion	GO_BiologicalProcess-EBI-UniProt-GOA-ACAP-ARAP_08.05.2020_00h00	0,00207315	0,014692351	0,02375092	0,02375092	[4, 5]	Group066	6,92	31,00
GO:0030334	regulation of cell migration	GO_BiologicalProcess-EBI-UniProt-GOA-ACAP-ARAP_08.05.2020_00h00	0,01196401	0,044209588	0,02375092	0,02375092	[4, 5, 6]	Group066	5,50	51,00
