# Supplementary material for: Analysis of the Role of FRMD5 in the Biology of Papillary Thyroid Carcinoma
Source: Int J Mol Sci. 2021 Jun 23;22(13):6726. doi: 10.3390/ijms22136726 (PMC8268710; doi:10.3390/ijms22136726)
Supplement: Supplementary file 1 [file ijms-22-06726-s001.zip › Supplementary Table S7.pdf]

Supplementary Table S7. Gene ontology (GO) enrichment analysis of the upregulated genes in BCPAP siFRMD5 (in comparison with BCPAP siNEG).											
GOID	GOTerm	Ontology Source	Term PValue	Term PValue Corrected with Benjamini-Hochberg	Group PValue	Group PValue Corrected with Benjamini-Hochberg	GOLevels	GOGroups	% Associated Genes	Nr. Genes
GO:0032879	regulation of localization	GO_BiologicalProcess-EBI-UniProt-GOA-ACAP-ARAP_08.05.2020_00h00	1,28192E-10	1,5335E-08	4,00741E-12	3,5666E-10	[2, 3]	Group185	4,45	133,00
GO:0040012	regulation of locomotion	GO_BiologicalProcess-EBI-UniProt-GOA-ACAP-ARAP_08.05.2020_00h00	1,89996E-12	1,81826E-09	4,00741E-12	3,5666E-10	[2, 3]	Group185	6,58	68,00
GO:0048870	cell motility	GO_BiologicalProcess-EBI-UniProt-GOA-ACAP-ARAP_08.05.2020_00h00	1,18486E-09	6,29948E-08	4,00741E-12	3,5666E-10	[2, 3]	Group185	5,00	90,00
GO:0031589	cell-substrate adhesion	GO_BiologicalProcess-EBI-UniProt-GOA-ACAP-ARAP_08.05.2020_00h00	3,26496E-10	3,47175E-08	4,00741E-12	3,5666E-10	[3]	Group185	9,07	34,00
GO:0040017	positive regulation of locomotion	GO_BiologicalProcess-EBI-UniProt-GOA-ACAP-ARAP_08.05.2020_00h00	2,08823E-09	9,51635E-08	4,00741E-12	3,5666E-10	[2, 3, 4]	Group185	7,20	43,00
GO:0006935	chemotaxis	GO_BiologicalProcess-EBI-UniProt-GOA-ACAP-ARAP_08.05.2020_00h00	0,001700784	0,00538957	4,00741E-12	3,5666E-10	[3, 4]	Group185	4,56	32,00
GO:0030155	regulation of cell adhesion	GO_BiologicalProcess-EBI-UniProt-GOA-ACAP-ARAP_08.05.2020_00h00	1,32928E-08	3,34768E-07	4,00741E-12	3,5666E-10	[3, 4]	Group185	6,36	48,00
GO:0051270	regulation of cellular component movement	GO_BiologicalProcess-EBI-UniProt-GOA-ACAP-ARAP_08.05.2020_00h00	4,42024E-11	2,11509E-08	4,00741E-12	3,5666E-10	[3, 4]	Group185	6,18	67,00
GO:0001935	endothelial cell proliferation	GO_BiologicalProcess-EBI-UniProt-GOA-ACAP-ARAP_08.05.2020_00h00	0,003633375	0,00934715	4,00741E-12	3,5666E-10	[4]	Group185	6,79	11,00
GO:0051271	negative regulation of cellular component movement	GO_BiologicalProcess-EBI-UniProt-GOA-ACAP-ARAP_08.05.2020_00h00	0,001396025	0,004606881	4,00741E-12	3,5666E-10	[3, 4, 5]	Group185	5,67	19,00
GO:0001667	ameboidal-type cell migration	GO_BiologicalProcess-EBI-UniProt-GOA-ACAP-ARAP_08.05.2020_00h00	3,07176E-05	0,00023147	4,00741E-12	3,5666E-10	[4, 5]	Group185	6,24	27,00
GO:0010631	epithelial cell migration	GO_BiologicalProcess-EBI-UniProt-GOA-ACAP-ARAP_08.05.2020_00h00	0,00018162	0,00096028	4,00741E-12	3,5666E-10	[4, 5, 6]	Group185	6,51	20,00
GO:0010632	regulation of epithelial cell migration	GO_BiologicalProcess-EBI-UniProt-GOA-ACAP-ARAP_08.05.2020_00h00	0,000186103	0,000978573	4,00741E-12	3,5666E-10	[3, 4, 5, 6, 7]	Group185	7,14	17,00
GO:0048661	positive regulation of smooth muscle cell proliferation	GO_BiologicalProcess-EBI-UniProt-GOA-ACAP-ARAP_08.05.2020_00h00	0,00013222	0,000762259	4,00741E-12	3,5666E-10	[4, 5, 6]	Group185	10,99	10,00
GO:0010634	positive regulation of epithelial cell migration	GO_BiologicalProcess-EBI-UniProt-GOA-ACAP-ARAP_08.05.2020_00h00	0,000839522	0,003066498	4,00741E-12	3,5666E-10	[3, 4, 5, 6, 7, 8]	Group185	7,69	12,00
GO:0030335	positive regulation of cell migration	GO_BiologicalProcess-EBI-UniProt-GOA-ACAP-ARAP_08.05.2020_00h00	8,27203E-10	5,65453E-08	4,00741E-12	3,5666E-10	[4, 5, 6, 7]	Group185	7,54	42,00
GO:0001568	blood vessel development	GO_BiologicalProcess-EBI-UniProt-GOA-ACAP-ARAP_08.05.2020_00h00	7,05492E-09	2,10986E-07	4,00741E-12	3,5666E-10	[3, 5, 6, 7, 8]	Group185	6,49	48,00
GO:0010594	regulation of endothelial cell migration	GO_BiologicalProcess-EBI-UniProt-GOA-ACAP-ARAP_08.05.2020_00h00	0,000681572	0,002640746	4,00741E-12	3,5666E-10	[4, 5, 6, 7, 8]	Group185	7,47	13,00
GO:0043542	endothelial cell migration	GO_BiologicalProcess-EBI-UniProt-GOA-ACAP-ARAP_08.05.2020_00h00	0,000331147	0,001553468	4,00741E-12	3,5666E-10	[5, 6, 7]	Group185	7,05	16,00
GO:1901342	regulation of vasculature development	GO_BiologicalProcess-EBI-UniProt-GOA-ACAP-ARAP_08.05.2020_00h00	1,26962E-05	0,00011147	4,00741E-12	3,5666E-10	[4, 5, 6, 7, 8]	Group185	6,85	25,00

GO:1904018	positive regulation of vasculature development	GO_BiologicalProcess-EBI-UniProt-GOA-ACAP-ARAP_08.05.2020_00h00	0,000129235	0,000758763	4,00741E-12	3,5666E-10	[3, 4, 5, 6, 7, 8, 9]	Group185	7,66	16,00
GO:0010595	positive regulation of endothelial cell migration	GO_BiologicalProcess-EBI-UniProt-GOA-ACAP-ARAP_08.05.2020_00h00	0,00920515	0,019361163	4,00741E-12	3,5666E-10	[4, 5, 6, 7, 8, 9]	Group185	7,14	8,00
GO:0048514	blood vessel morphogenesis	GO_BiologicalProcess-EBI-UniProt-GOA-ACAP-ARAP_08.05.2020_00h00	9,25827E-09	2,60593E-07	4,00741E-12	3,5666E-10	[4, 5, 6, 7, 8, 9]	Group185	6,76	44,00
GO:0043534	blood vessel endothelial cell migration	GO_BiologicalProcess-EBI-UniProt-GOA-ACAP-ARAP_08.05.2020_00h00	0,00532592	0,012492415	4,00741E-12	3,5666E-10	[6, 7, 8]	Group185	7,26	9,00
GO:0043535	regulation of blood vessel endothelial cell migration	GO_BiologicalProcess-EBI-UniProt-GOA-ACAP-ARAP_08.05.2020_00h00	0,004171744	0,010316174	4,00741E-12	3,5666E-10	[5, 6, 7, 8, 9]	Group185	8,16	8,00
GO:0043536	positive regulation of blood vessel endothelial cell migration	GO_BiologicalProcess-EBI-UniProt-GOA-ACAP-ARAP_08.05.2020_00h00	0,02016817	0,034589496	4,00741E-12	3,5666E-10	[5, 6, 7, 8, 9, 10]	Group185	8,33	5,00
GO:0045765	regulation of angiogenesis	GO_BiologicalProcess-EBI-UniProt-GOA-ACAP-ARAP_08.05.2020_00h00	5,97633E-05	0,000405628	4,00741E-12	3,5666E-10	[4, 5, 6, 7, 8, 9, 10, 11]	Group185	6,69	22,00
GO:1902895	positive regulation of pri-miRNA transcription by RNA polymerase II	GO_BiologicalProcess-EBI-UniProt-GOA-ACAP-ARAP_08.05.2020_00h00	0,000732948	0,002794546	2,63149E-12	4,6841E-10	[7, 8, 9, 10, 11, 12, 13]	Group192	14,29	6,00
GO:0009653	anatomical structure morphogenesis	GO_BiologicalProcess-EBI-UniProt-GOA-ACAP-ARAP_08.05.2020_00h00	9,29458E-11	1,48249E-08	2,63149E-12	4,6841E-10	[2, 3]	Group192	4,51	130,00
GO:0023051	regulation of signaling	GO_BiologicalProcess-EBI-UniProt-GOA-ACAP-ARAP_08.05.2020_00h00	4,33256E-10	3,76933E-08	2,63149E-12	4,6841E-10	[2, 3]	Group192	4,12	158,00
GO:0032879	regulation of localization	GO_BiologicalProcess-EBI-UniProt-GOA-ACAP-ARAP_08.05.2020_00h00	1,28192E-10	1,5335E-08	2,63149E-12	4,6841E-10	[2, 3]	Group192	4,45	133,00
GO:0040012	regulation of locomotion	GO_BiologicalProcess-EBI-UniProt-GOA-ACAP-ARAP_08.05.2020_00h00	1,89996E-12	1,81826E-09	2,63149E-12	4,6841E-10	[2, 3]	Group192	6,58	68,00
GO:0048583	regulation of response to stimulus	GO_BiologicalProcess-EBI-UniProt-GOA-ACAP-ARAP_08.05.2020_00h00	6,27191E-11	2,00074E-08	2,63149E-12	4,6841E-10	[2, 3]	Group192	4,02	182,00
GO:0048870	cell motility	GO_BiologicalProcess-EBI-UniProt-GOA-ACAP-ARAP_08.05.2020_00h00	1,18486E-09	6,29948E-08	2,63149E-12	4,6841E-10	[2, 3]	Group192	5,00	90,00
GO:0050793	regulation of developmental process	GO_BiologicalProcess-EBI-UniProt-GOA-ACAP-ARAP_08.05.2020_00h00	1,13592E-09	6,39455E-08	2,63149E-12	4,6841E-10	[2, 3]	Group192	4,42	124,00
GO:0051239	regulation of multicellular organismal process	GO_BiologicalProcess-EBI-UniProt-GOA-ACAP-ARAP_08.05.2020_00h00	8,97701E-09	2,60333E-07	2,63149E-12	4,6841E-10	[2, 3]	Group192	4,10	140,00
GO:0009888	tissue development	GO_BiologicalProcess-EBI-UniProt-GOA-ACAP-ARAP_08.05.2020_00h00	5,58784E-08	1,00898E-06	2,63149E-12	4,6841E-10	[3]	Group192	4,48	98,00
GO:0023056	positive regulation of signaling	GO_BiologicalProcess-EBI-UniProt-GOA-ACAP-ARAP_08.05.2020_00h00	6,42911E-11	1,53817E-08	2,63149E-12	4,6841E-10	[2, 3, 4]	Group192	5,00	102,00
GO:0040017	positive regulation of locomotion	GO_BiologicalProcess-EBI-UniProt-GOA-ACAP-ARAP_08.05.2020_00h00	2,08823E-09	9,51635E-08	2,63149E-12	4,6841E-10	[2, 3, 4]	Group192	7,20	43,00
GO:0048584	positive regulation of response to stimulus	GO_BiologicalProcess-EBI-UniProt-GOA-ACAP-ARAP_08.05.2020_00h00	2,07976E-07	3,15925E-06	2,63149E-12	4,6841E-10	[2, 3, 4]	Group192	4,21	109,00
GO:0048646	anatomical structure formation involved in morphogenesis	GO_BiologicalProcess-EBI-UniProt-GOA-ACAP-ARAP_08.05.2020_00h00	2,69379E-08	5,60425E-07	2,63149E-12	4,6841E-10	[2, 3, 4]	Group192	5,40	64,00
GO:0051093	negative regulation of developmental process	GO_BiologicalProcess-EBI-UniProt-GOA-ACAP-ARAP_08.05.2020_00h00	0,002781123	0,007692298	2,63149E-12	4,6841E-10	[2, 3, 4]	Group192	4,07	43,00
GO:0051094	positive regulation of developmental process	GO_BiologicalProcess-EBI-UniProt-GOA-ACAP-ARAP_08.05.2020_00h00	8,57256E-07	1,07947E-05	2,63149E-12	4,6841E-10	[2, 3, 4]	Group192	4,74	70,00

GO:0051240	positive regulation of multicellular organismal process	GO_BiologicalProcess-EBI-UniProt-GOA-ACAP-ARAP_08.05.2020_00h00	3,52846E-07	5,03991E-06	2,63149E-12	4,6841E-10	[2, 3, 4]	Group192	4,50	86,00
GO:0051241	negative regulation of multicellular organismal process	GO_BiologicalProcess-EBI-UniProt-GOA-ACAP-ARAP_08.05.2020_00h00	0,001074145	0,003697685	2,63149E-12	4,6841E-10	[2, 3, 4]	Group192	4,04	53,00
GO:0000902	cell morphogenesis	GO_BiologicalProcess-EBI-UniProt-GOA-ACAP-ARAP_08.05.2020_00h00	0,000202677	0,001031712	2,63149E-12	4,6841E-10	[3, 4]	Group192	4,42	50,00
GO:0006935	chemotaxis	GO_BiologicalProcess-EBI-UniProt-GOA-ACAP-ARAP_08.05.2020_00h00	0,001700784	0,00538957	2,63149E-12	4,6841E-10	[3, 4]	Group192	4,56	32,00
GO:0009790	embryo development	GO_BiologicalProcess-EBI-UniProt-GOA-ACAP-ARAP_08.05.2020_00h00	0,000211308	0,001058752	2,63149E-12	4,6841E-10	[3, 4]	Group192	4,41	50,00
GO:0010646	regulation of cell communication	GO_BiologicalProcess-EBI-UniProt-GOA-ACAP-ARAP_08.05.2020_00h00	3,57848E-10	3,4246E-08	2,63149E-12	4,6841E-10	[3, 4]	Group192	4,14	157,00
GO:0010941	regulation of cell death	GO_BiologicalProcess-EBI-UniProt-GOA-ACAP-ARAP_08.05.2020_00h00	3,69996E-07	5,20716E-06	2,63149E-12	4,6841E-10	[3, 4]	Group192	4,57	82,00
GO:0022603	regulation of anatomical structure morphogenesis	GO_BiologicalProcess-EBI-UniProt-GOA-ACAP-ARAP_08.05.2020_00h00	5,07994E-07	6,84718E-06	2,63149E-12	4,6841E-10	[3, 4]	Group192	5,11	60,00
GO:0035295	tube development	GO_BiologicalProcess-EBI-UniProt-GOA-ACAP-ARAP_08.05.2020_00h00	2,65621E-08	5,64888E-07	2,63149E-12	4,6841E-10	[3, 4]	Group192	5,48	62,00
GO:0045595	regulation of cell differentiation	GO_BiologicalProcess-EBI-UniProt-GOA-ACAP-ARAP_08.05.2020_00h00	5,09874E-05	0,000353586	2,63149E-12	4,6841E-10	[3, 4]	Group192	4,04	80,00
GO:0048468	cell development	GO_BiologicalProcess-EBI-UniProt-GOA-ACAP-ARAP_08.05.2020_00h00	1,28955E-05	0,00011219	2,63149E-12	4,6841E-10	[3, 4]	Group192	4,03	93,00
GO:0048729	tissue morphogenesis	GO_BiologicalProcess-EBI-UniProt-GOA-ACAP-ARAP_08.05.2020_00h00	7,67653E-05	0,000486519	2,63149E-12	4,6841E-10	[3, 4]	Group192	5,14	37,00
GO:0051270	regulation of cellular component movement	GO_BiologicalProcess-EBI-UniProt-GOA-ACAP-ARAP_08.05.2020_00h00	4,42024E-11	2,11509E-08	2,63149E-12	4,6841E-10	[3, 4]	Group192	6,18	67,00
GO:2000026	regulation of multicellular organismal development	GO_BiologicalProcess-EBI-UniProt-GOA-ACAP-ARAP_08.05.2020_00h00	3,43602E-09	1,42968E-07	2,63149E-12	4,6841E-10	[3, 4]	Group192	4,64	103,00
GO:0001935	endothelial cell proliferation	GO_BiologicalProcess-EBI-UniProt-GOA-ACAP-ARAP_08.05.2020_00h00	0,003633375	0,00934715	2,63149E-12	4,6841E-10	[4]	Group192	6,79	11,00
GO:0007166	cell surface receptor signaling pathway	GO_BiologicalProcess-EBI-UniProt-GOA-ACAP-ARAP_08.05.2020_00h00	2,092E-08	4,76678E-07	2,63149E-12	4,6841E-10	[3, 4, 5]	Group192	4,08	137,00
GO:0010647	positive regulation of cell communication	GO_BiologicalProcess-EBI-UniProt-GOA-ACAP-ARAP_08.05.2020_00h00	1,14659E-10	1,56756E-08	2,63149E-12	4,6841E-10	[3, 4, 5]	Group192	4,98	101,00
GO:0035239	tube morphogenesis	GO_BiologicalProcess-EBI-UniProt-GOA-ACAP-ARAP_08.05.2020_00h00	6,64249E-09	2,11896E-07	2,63149E-12	4,6841E-10	[3, 4, 5]	Group192	6,04	55,00
GO:0045597	positive regulation of cell differentiation	GO_BiologicalProcess-EBI-UniProt-GOA-ACAP-ARAP_08.05.2020_00h00	0,00015702	0,000878762	2,63149E-12	4,6841E-10	[3, 4, 5]	Group192	4,52	48,00
GO:0048588	developmental cell growth	GO_BiologicalProcess-EBI-UniProt-GOA-ACAP-ARAP_08.05.2020_00h00	0,01525921	0,027815361	2,63149E-12	4,6841E-10	[3, 4, 5]	Group192	5,18	13,00
GO:0048598	embryonic morphogenesis	GO_BiologicalProcess-EBI-UniProt-GOA-ACAP-ARAP_08.05.2020_00h00	0,000934627	0,003362548	2,63149E-12	4,6841E-10	[3, 4, 5]	Group192	4,79	31,00
GO:0051130	positive regulation of cellular component organization	GO_BiologicalProcess-EBI-UniProt-GOA-ACAP-ARAP_08.05.2020_00h00	7,13305E-08	1,24115E-06	2,63149E-12	4,6841E-10	[3, 4, 5]	Group192	5,15	67,00
GO:0060429	epithelium development	GO_BiologicalProcess-EBI-UniProt-GOA-ACAP-ARAP_08.05.2020_00h00	5,08926E-06	5,18131E-05	2,63149E-12	4,6841E-10	[4]	Group192	4,64	64,00

GO:0060548	negative regulation of cell death	GO_BiologicalProcess-EBI-UniProt-GOA-ACAP-ARAP_08.05.2020_00h00	6,67908E-06	6,3286E-05	2,63149E-12	4,6841E-10	[3, 4, 5]	Group192	4,93	53,00
GO:0000904	cell morphogenesis involved in differentiation	GO_BiologicalProcess-EBI-UniProt-GOA-ACAP-ARAP_08.05.2020_00h00	0,000202059	0,001034068	2,63149E-12	4,6841E-10	[4, 5]	Group192	4,80	39,00
GO:0001667	ameboidal-type cell migration	GO_BiologicalProcess-EBI-UniProt-GOA-ACAP-ARAP_08.05.2020_00h00	3,07176E-05	0,00023147	2,63149E-12	4,6841E-10	[4, 5]	Group192	6,24	27,00
GO:0002009	morphogenesis of an epithelium	GO_BiologicalProcess-EBI-UniProt-GOA-ACAP-ARAP_08.05.2020_00h00	0,000210357	0,001059536	2,63149E-12	4,6841E-10	[4, 5]	Group192	5,24	31,00
GO:0009887	animal organ morphogenesis	GO_BiologicalProcess-EBI-UniProt-GOA-ACAP-ARAP_08.05.2020_00h00	0,000679847	0,002644772	2,63149E-12	4,6841E-10	[3, 4, 5, 6]	Group192	4,21	49,00
GO:0022604	regulation of cell morphogenesis	GO_BiologicalProcess-EBI-UniProt-GOA-ACAP-ARAP_08.05.2020_00h00	0,025190763	0,040860273	2,63149E-12	4,6841E-10	[4, 5]	Group192	4,11	22,00
GO:0031344	regulation of cell projection organization	GO_BiologicalProcess-EBI-UniProt-GOA-ACAP-ARAP_08.05.2020_00h00	0,004833171	0,011592343	2,63149E-12	4,6841E-10	[4, 5]	Group192	4,26	32,00
GO:0048534	hematopoietic or lymphoid organ development	GO_BiologicalProcess-EBI-UniProt-GOA-ACAP-ARAP_08.05.2020_00h00	2,0816E-05	0,000167403	2,63149E-12	4,6841E-10	[3, 4, 5, 6]	Group192	4,88	49,00
GO:0072359	circulatory system development	GO_BiologicalProcess-EBI-UniProt-GOA-ACAP-ARAP_08.05.2020_00h00	3,81703E-07	5,29405E-06	2,63149E-12	4,6841E-10	[4, 5]	Group192	5,15	60,00
GO:0010631	epithelial cell migration	GO_BiologicalProcess-EBI-UniProt-GOA-ACAP-ARAP_08.05.2020_00h00	0,00018162	0,00096028	2,63149E-12	4,6841E-10	[4, 5, 6]	Group192	6,51	20,00
GO:0010632	regulation of epithelial cell migration	GO_BiologicalProcess-EBI-UniProt-GOA-ACAP-ARAP_08.05.2020_00h00	0,000186103	0,000978573	2,63149E-12	4,6841E-10	[3, 4, 5, 6, 7]	Group192	7,14	17,00
GO:0031346	positive regulation of cell projection organization	GO_BiologicalProcess-EBI-UniProt-GOA-ACAP-ARAP_08.05.2020_00h00	0,009294322	0,019420668	2,63149E-12	4,6841E-10	[4, 5, 6]	Group192	4,67	20,00
GO:0048661	positive regulation of smooth muscle cell proliferation	GO_BiologicalProcess-EBI-UniProt-GOA-ACAP-ARAP_08.05.2020_00h00	0,00013222	0,000762259	2,63149E-12	4,6841E-10	[4, 5, 6]	Group192	10,99	10,00
GO:0051962	positive regulation of nervous system development	GO_BiologicalProcess-EBI-UniProt-GOA-ACAP-ARAP_08.05.2020_00h00	0,024883866	0,040431	2,63149E-12	4,6841E-10	[3, 4, 5, 6, 7]	Group192	4,03	24,00
GO:0060562	epithelial tube morphogenesis	GO_BiologicalProcess-EBI-UniProt-GOA-ACAP-ARAP_08.05.2020_00h00	0,025199339	0,040805023	2,63149E-12	4,6841E-10	[4, 5, 6]	Group192	4,51	16,00
GO:0001944	vasculature development	GO_BiologicalProcess-EBI-UniProt-GOA-ACAP-ARAP_08.05.2020_00h00	3,67366E-09	1,46487E-07	2,63149E-12	4,6841E-10	[4, 5, 6, 7]	Group192	6,47	50,00
GO:0010634	positive regulation of epithelial cell migration	GO_BiologicalProcess-EBI-UniProt-GOA-ACAP-ARAP_08.05.2020_00h00	0,000839522	0,003066498	2,63149E-12	4,6841E-10	[3, 4, 5, 6, 7, 8]	Group192	7,69	12,00
GO:0010769	regulation of cell morphogenesis involved in differentiation	GO_BiologicalProcess-EBI-UniProt-GOA-ACAP-ARAP_08.05.2020_00h00	0,014062108	0,026284058	2,63149E-12	4,6841E-10	[5, 6]	Group192	4,83	16,00
GO:0030335	positive regulation of cell migration	GO_BiologicalProcess-EBI-UniProt-GOA-ACAP-ARAP_08.05.2020_00h00	8,27203E-10	5,65453E-08	2,63149E-12	4,6841E-10	[4, 5, 6, 7]	Group192	7,54	42,00
GO:0120035	regulation of plasma membrane bounded cell projection organization	GO_BiologicalProcess-EBI-UniProt-GOA-ACAP-ARAP_08.05.2020_00h00	0,012516662	0,024396019	2,63149E-12	4,6841E-10	[5, 6]	Group192	4,04	30,00
GO:0001568	blood vessel development	GO_BiologicalProcess-EBI-UniProt-GOA-ACAP-ARAP_08.05.2020_00h00	7,05492E-09	2,10986E-07	2,63149E-12	4,6841E-10	[3, 5, 6, 7, 8]	Group192	6,49	48,00
GO:0010594	regulation of endothelial cell migration	GO_BiologicalProcess-EBI-UniProt-GOA-ACAP-ARAP_08.05.2020_00h00	0,000681572	0,002640746	2,63149E-12	4,6841E-10	[4, 5, 6, 7, 8]	Group192	7,47	13,00
GO:0043542	endothelial cell migration	GO_BiologicalProcess-EBI-UniProt-GOA-ACAP-ARAP_08.05.2020_00h00	0,000331147	0,001553468	2,63149E-12	4,6841E-10	[5, 6, 7]	Group192	7,05	16,00

GO:0045937	positive regulation of phosphate metabolic process	GO_BiologicalProcess-EBI-UniProt-GOA-ACAP-ARAP_08.05.2020_00h00	1,12601E-09	6,73495E-08	2,63149E-12	4,6841E-10	[5, 6, 7]	Group192	5,60	70,00
GO:0120039	plasma membrane bounded cell projection morphogenesis	GO_BiologicalProcess-EBI-UniProt-GOA-ACAP-ARAP_08.05.2020_00h00	0,009786652	0,020228567	2,63149E-12	4,6841E-10	[5, 6, 7]	Group192	4,12	30,00
GO:1901342	regulation of vasculature development	GO_BiologicalProcess-EBI-UniProt-GOA-ACAP-ARAP_08.05.2020_00h00	1,26962E-05	0,00011147	2,63149E-12	4,6841E-10	[4, 5, 6, 7, 8]	Group192	6,85	25,00
GO:1901343	negative regulation of vasculature development	GO_BiologicalProcess-EBI-UniProt-GOA-ACAP-ARAP_08.05.2020_00h00	0,028789286	0,045166142	2,63149E-12	4,6841E-10	[3, 4, 5, 6, 7, 8, 9]	Group192	5,80	8,00
GO:1904018	positive regulation of vasculature development	GO_BiologicalProcess-EBI-UniProt-GOA-ACAP-ARAP_08.05.2020_00h00	0,000129235	0,000758763	2,63149E-12	4,6841E-10	[3, 4, 5, 6, 7, 8, 9]	Group192	7,66	16,00
GO:1904707	positive regulation of vascular smooth muscle cell proliferation	GO_BiologicalProcess-EBI-UniProt-GOA-ACAP-ARAP_08.05.2020_00h00	0,015443421	0,028044315	2,63149E-12	4,6841E-10	[5, 6, 7]	Group192	10,81	4,00
GO:0001525	angiogenesis	GO_BiologicalProcess-EBI-UniProt-GOA-ACAP-ARAP_08.05.2020_00h00	5,95789E-09	2,11174E-07	2,63149E-12	4,6841E-10	[3, 4, 5, 6, 7, 8, 9, 10]	Group192	7,27	40,00
GO:0010595	positive regulation of endothelial cell migration	GO_BiologicalProcess-EBI-UniProt-GOA-ACAP-ARAP_08.05.2020_00h00	0,00920515	0,019361163	2,63149E-12	4,6841E-10	[4, 5, 6, 7, 8, 9]	Group192	7,14	8,00
GO:0048514	blood vessel morphogenesis	GO_BiologicalProcess-EBI-UniProt-GOA-ACAP-ARAP_08.05.2020_00h00	9,25827E-09	2,60593E-07	2,63149E-12	4,6841E-10	[4, 5, 6, 7, 8, 9]	Group192	6,76	44,00
GO:0042327	positive regulation of phosphorylation	GO_BiologicalProcess-EBI-UniProt-GOA-ACAP-ARAP_08.05.2020_00h00	3,4551E-08	6,74801E-07	2,63149E-12	4,6841E-10	[6, 7, 8]	Group192	5,40	63,00
GO:0043534	blood vessel endothelial cell migration	GO_BiologicalProcess-EBI-UniProt-GOA-ACAP-ARAP_08.05.2020_00h00	0,00532592	0,012492415	2,63149E-12	4,6841E-10	[6, 7, 8]	Group192	7,26	9,00
GO:0043535	regulation of blood vessel endothelial cell migration	GO_BiologicalProcess-EBI-UniProt-GOA-ACAP-ARAP_08.05.2020_00h00	0,004171744	0,010316174	2,63149E-12	4,6841E-10	[5, 6, 7, 8, 9]	Group192	8,16	8,00
GO:0048667	cell morphogenesis involved in neuron differentiation	GO_BiologicalProcess-EBI-UniProt-GOA-ACAP-ARAP_08.05.2020_00h00	0,006632533	0,014899846	2,63149E-12	4,6841E-10	[5, 6, 8, 9, 10]	Group192	4,33	28,00
GO:0002040	sprouting angiogenesis	GO_BiologicalProcess-EBI-UniProt-GOA-ACAP-ARAP_08.05.2020_00h00	0,00041584	0,001833911	2,63149E-12	4,6841E-10	[4, 5, 6, 7, 8, 9, 10, 11]	Group192	8,87	11,00
GO:0043536	positive regulation of blood vessel endothelial cell migration	GO_BiologicalProcess-EBI-UniProt-GOA-ACAP-ARAP_08.05.2020_00h00	0,02016817	0,034589496	2,63149E-12	4,6841E-10	[5, 6, 7, 8, 9, 10]	Group192	8,33	5,00
GO:0045765	regulation of angiogenesis	GO_BiologicalProcess-EBI-UniProt-GOA-ACAP-ARAP_08.05.2020_00h00	5,97633E-05	0,000405628	2,63149E-12	4,6841E-10	[4, 5, 6, 7, 8, 9, 10, 11]	Group192	6,69	22,00
GO:0045766	positive regulation of angiogenesis	GO_BiologicalProcess-EBI-UniProt-GOA-ACAP-ARAP_08.05.2020_00h00	0,00132399	0,004430275	2,63149E-12	4,6841E-10	[4, 5, 6, 7, 8, 9, 10, 11, 12]	Group192	6,95	13,00
GO:0007411	axon guidance	GO_BiologicalProcess-EBI-UniProt-GOA-ACAP-ARAP_08.05.2020_00h00	0,014432535	0,026663968	2,63149E-12	4,6841E-10	[4, 5, 6, 7, 8, 9, 10, 11, 12, 13]	Group192	4,93	15,00
GO:0010975	regulation of neuron projection development	GO_BiologicalProcess-EBI-UniProt-GOA-ACAP-ARAP_08.05.2020_00h00	0,011319031	0,022567319	2,63149E-12	4,6841E-10	[6, 7, 8, 9, 10, 11]	Group192	4,33	24,00
GO:1903670	regulation of sprouting angiogenesis	GO_BiologicalProcess-EBI-UniProt-GOA-ACAP-ARAP_08.05.2020_00h00	0,003441989	0,008951043	2,63149E-12	4,6841E-10	[5, 6, 7, 8, 9, 10, 11, 12]	Group192	9,33	7,00
GO:0061564	axon development	GO_BiologicalProcess-EBI-UniProt-GOA-ACAP-ARAP_08.05.2020_00h00	0,00784214	0,01709551	2,63149E-12	4,6841E-10	[6, 7, 9, 10, 11]	Group192	4,42	25,00
GO:0050770	regulation of axonogenesis	GO_BiologicalProcess-EBI-UniProt-GOA-ACAP-ARAP_08.05.2020_00h00	0,00706902	0,015696175	2,63149E-12	4,6841E-10	[6, 7, 8, 9, 10, 11, 12, 13]	Group192	5,94	12,00
GO:0012501	programmed cell death	GO_BiologicalProcess-EBI-UniProt-GOA-ACAP-ARAP_08.05.2020_00h00	9,88592E-07	1,19757E-05	1,45453E-11	6,4727E-10	[3]	Group187	4,27	94,00

GO:0044092	negative regulation of molecular function	GO_BiologicalProcess-EBI-UniProt-GOA-ACAP-ARAP_08.05.2020_00h00	0,001134739	0,003864575	1,45453E-11	6,4727E-10	[3]	Group187	4,07	51,00
GO:0010941	regulation of cell death	GO_BiologicalProcess-EBI-UniProt-GOA-ACAP-ARAP_08.05.2020_00h00	3,69996E-07	5,20716E-06	1,45453E-11	6,4727E-10	[3, 4]	Group187	4,57	82,00
GO:0010942	positive regulation of cell death	GO_BiologicalProcess-EBI-UniProt-GOA-ACAP-ARAP_08.05.2020_00h00	5,54439E-06	5,52706E-05	1,45453E-11	6,4727E-10	[3, 4, 5]	Group187	5,53	41,00
GO:0043086	negative regulation of catalytic activity	GO_BiologicalProcess-EBI-UniProt-GOA-ACAP-ARAP_08.05.2020_00h00	0,003219853	0,008512152	1,45453E-11	6,4727E-10	[4]	Group187	4,20	37,00
GO:0051336	regulation of hydrolase activity	GO_BiologicalProcess-EBI-UniProt-GOA-ACAP-ARAP_08.05.2020_00h00	8,71715E-06	7,94506E-05	1,45453E-11	6,4727E-10	[4]	Group187	4,59	63,00
GO:0097190	apoptotic signaling pathway	GO_BiologicalProcess-EBI-UniProt-GOA-ACAP-ARAP_08.05.2020_00h00	1,38238E-06	1,63326E-05	1,45453E-11	6,4727E-10	[3, 4, 5]	Group187	6,00	39,00
GO:0006508	proteolysis	GO_BiologicalProcess-EBI-UniProt-GOA-ACAP-ARAP_08.05.2020_00h00	5,7795E-05	0,00039507	1,45453E-11	6,4727E-10	[4, 5]	Group187	4,07	77,00
GO:0030307	positive regulation of cell growth	GO_BiologicalProcess-EBI-UniProt-GOA-ACAP-ARAP_08.05.2020_00h00	0,020310845	0,034771878	1,45453E-11	6,4727E-10	[3, 4, 5, 6]	Group187	5,56	10,00
GO:0051248	negative regulation of protein metabolic process	GO_BiologicalProcess-EBI-UniProt-GOA-ACAP-ARAP_08.05.2020_00h00	0,000265156	0,001301303	1,45453E-11	6,4727E-10	[4, 5, 6]	Group187	4,34	51,00
GO:0051345	positive regulation of hydrolase activity	GO_BiologicalProcess-EBI-UniProt-GOA-ACAP-ARAP_08.05.2020_00h00	9,69574E-06	8,7536E-05	1,45453E-11	6,4727E-10	[5]	Group187	5,30	43,00
GO:0001836	release of cytochrome c from mitochondria	GO_BiologicalProcess-EBI-UniProt-GOA-ACAP-ARAP_08.05.2020_00h00	0,001802429	0,005618646	1,45453E-11	6,4727E-10	[4, 5, 6, 7]	Group187	10,45	7,00
GO:0008233	peptidase activity	GO_BiologicalProcess-EBI-UniProt-GOA-ACAP-ARAP_08.05.2020_00h00	0,0002754	0,001331101	1,45453E-11	6,4727E-10	[5, 6]	Group187	4,39	49,00
GO:0030162	regulation of proteolysis	GO_BiologicalProcess-EBI-UniProt-GOA-ACAP-ARAP_08.05.2020_00h00	0,004999209	0,011901102	1,45453E-11	6,4727E-10	[5, 6]	Group187	4,21	33,00
GO:0032269	negative regulation of cellular protein metabolic process	GO_BiologicalProcess-EBI-UniProt-GOA-ACAP-ARAP_08.05.2020_00h00	0,00020134	0,001035927	1,45453E-11	6,4727E-10	[4, 5, 6, 7]	Group187	4,45	49,00
GO:0042177	negative regulation of protein catabolic process	GO_BiologicalProcess-EBI-UniProt-GOA-ACAP-ARAP_08.05.2020_00h00	0,032142218	0,048748184	1,45453E-11	6,4727E-10	[4, 5, 6, 7]	Group187	5,67	8,00
GO:0042981	regulation of apoptotic process	GO_BiologicalProcess-EBI-UniProt-GOA-ACAP-ARAP_08.05.2020_00h00	1,11326E-06	1,33174E-05	1,45453E-11	6,4727E-10	[5, 6]	Group187	4,59	75,00
GO:0043065	positive regulation of apoptotic process	GO_BiologicalProcess-EBI-UniProt-GOA-ACAP-ARAP_08.05.2020_00h00	5,25908E-05	0,000362082	1,45453E-11	6,4727E-10	[5, 6, 7]	Group187	5,29	36,00
GO:0045861	negative regulation of proteolysis	GO_BiologicalProcess-EBI-UniProt-GOA-ACAP-ARAP_08.05.2020_00h00	0,006054374	0,013861331	1,45453E-11	6,4727E-10	[5, 6, 7, 8]	Group187	4,96	19,00
GO:0045862	positive regulation of proteolysis	GO_BiologicalProcess-EBI-UniProt-GOA-ACAP-ARAP_08.05.2020_00h00	0,023925476	0,039273895	1,45453E-11	6,4727E-10	[5, 6, 7, 8]	Group187	4,45	17,00
GO:0070011	peptidase activity, acting on L-amino acid peptides	GO_BiologicalProcess-EBI-UniProt-GOA-ACAP-ARAP_08.05.2020_00h00	0,00039584	0,00177018	1,45453E-11	6,4727E-10	[6, 7]	Group187	4,37	47,00
GO:0004175	endopeptidase activity	GO_BiologicalProcess-EBI-UniProt-GOA-ACAP-ARAP_08.05.2020_00h00	0,000555813	0,002282888	1,45453E-11	6,4727E-10	[7, 8]	Group187	4,52	40,00
GO:0008234	cysteine-type peptidase activity	GO_BiologicalProcess-EBI-UniProt-GOA-ACAP-ARAP_08.05.2020_00h00	0,000334646	0,001554642	1,45453E-11	6,4727E-10	[7, 8]	Group187	5,49	26,00
GO:0010952	positive regulation of peptidase activity	GO_BiologicalProcess-EBI-UniProt-GOA-ACAP-ARAP_08.05.2020_00h00	0,000395714	0,001777926	1,45453E-11	6,4727E-10	[6, 7, 8, 9]	Group187	7,21	15,00

GO:0052548	regulation of endopeptidase activity	GO_BiologicalProcess-EBI-UniProt-GOA-ACAP-ARAP_08.05.2020_00h00	0,000937128	0,003333945	1,45453E-11	6,4727E-10	[6, 7, 8, 9]	Group187	5,29	24,00
GO:0097153	cysteine-type endopeptidase activity involved in apoptotic process	GO_BiologicalProcess-EBI-UniProt-GOA-ACAP-ARAP_08.05.2020_00h00	0,000249894	0,001232725	1,45453E-11	6,4727E-10	[5, 9, 10]	Group187	6,97	17,00
GO:0004197	cysteine-type endopeptidase activity	GO_BiologicalProcess-EBI-UniProt-GOA-ACAP-ARAP_08.05.2020_00h00	0,000848939	0,003089106	1,45453E-11	6,4727E-10	[8, 9]	Group187	5,65	21,00
GO:0010951	negative regulation of endopeptidase activity	GO_BiologicalProcess-EBI-UniProt-GOA-ACAP-ARAP_08.05.2020_00h00	0,013479644	0,025748543	1,45453E-11	6,4727E-10	[7, 8, 9, 10]	Group187	5,11	14,00
GO:0033135	regulation of peptidyl-serine phosphorylation	GO_BiologicalProcess-EBI-UniProt-GOA-ACAP-ARAP_08.05.2020_00h00	0,011230711	0,022437977	1,45453E-11	6,4727E-10	[7, 8, 9, 10]	Group187	6,10	10,00
GO:0043281	regulation of cysteine-type endopeptidase activity involved in apoptotic process	GO_BiologicalProcess-EBI-UniProt-GOA-ACAP-ARAP_08.05.2020_00h00	0,000159916	0,000884625	1,45453E-11	6,4727E-10	[6, 7, 8, 9, 10, 11]	Group187	7,23	17,00
GO:0043027	cysteine-type endopeptidase inhibitor activity involved in apoptotic process	GO_BiologicalProcess-EBI-UniProt-GOA-ACAP-ARAP_08.05.2020_00h00	0,032678593	0,04924947	1,45453E-11	6,4727E-10	[5, 6, 7, 8, 9, 10, 11, 12, 13]	Group187	11,11	3,00
GO:0043154	negative regulation of cysteine-type endopeptidase activity involved in apoptotic process	GO_BiologicalProcess-EBI-UniProt-GOA-ACAP-ARAP_08.05.2020_00h00	0,000993196	0,003468936	1,45453E-11	6,4727E-10	[6, 7, 8, 9, 10, 11, 12]	Group187	9,28	9,00
GO:0043280	positive regulation of cysteine-type endopeptidase activity involved in apoptotic process	GO_BiologicalProcess-EBI-UniProt-GOA-ACAP-ARAP_08.05.2020_00h00	0,000360827	0,001652209	1,45453E-11	6,4727E-10	[6, 7, 8, 9, 10, 11, 12]	Group187	8,45	12,00
GO:1902895	positive regulation of pri-miRNA transcription by RNA polymerase II	GO_BiologicalProcess-EBI-UniProt-GOA-ACAP-ARAP_08.05.2020_00h00	0,000732948	0,002794546	1,11787E-11	6,6327E-10	[7, 8, 9, 10, 11, 12, 13]	Group191	14,29	6,00
GO:0033002	muscle cell proliferation	GO_BiologicalProcess-EBI-UniProt-GOA-ACAP-ARAP_08.05.2020_00h00	3,12928E-05	0,000233963	1,11787E-11	6,6327E-10	[3]	Group191	8,25	17,00
GO:0050673	epithelial cell proliferation	GO_BiologicalProcess-EBI-UniProt-GOA-ACAP-ARAP_08.05.2020_00h00	3,83793E-06	3,94935E-05	1,11787E-11	6,6327E-10	[3]	Group191	6,71	29,00
GO:0035019	somatic stem cell population maintenance	GO_BiologicalProcess-EBI-UniProt-GOA-ACAP-ARAP_08.05.2020_00h00	0,020465165	0,034911167	1,11787E-11	6,6327E-10	[3, 4]	Group191	7,32	6,00
GO:0001935	endothelial cell proliferation	GO_BiologicalProcess-EBI-UniProt-GOA-ACAP-ARAP_08.05.2020_00h00	0,003633375	0,00934715	1,11787E-11	6,6327E-10	[4]	Group191	6,79	11,00
GO:0008284	positive regulation of cell population proliferation	GO_BiologicalProcess-EBI-UniProt-GOA-ACAP-ARAP_08.05.2020_00h00	8,18119E-08	1,37358E-06	1,11787E-11	6,6327E-10	[3, 4, 5]	Group191	5,61	55,00
GO:0046620	regulation of organ growth	GO_BiologicalProcess-EBI-UniProt-GOA-ACAP-ARAP_08.05.2020_00h00	0,024403341	0,039853238	1,11787E-11	6,6327E-10	[3, 4, 5]	Group191	6,42	7,00
GO:0048659	smooth muscle cell proliferation	GO_BiologicalProcess-EBI-UniProt-GOA-ACAP-ARAP_08.05.2020_00h00	0,000131848	0,000764721	1,11787E-11	6,6327E-10	[4]	Group191	8,84	13,00
GO:0062013	positive regulation of small molecule metabolic process	GO_BiologicalProcess-EBI-UniProt-GOA-ACAP-ARAP_08.05.2020_00h00	0,008775524	0,018580036	1,11787E-11	6,6327E-10	[3, 4, 5]	Group191	6,33	10,00
GO:0006109	regulation of carbohydrate metabolic process	GO_BiologicalProcess-EBI-UniProt-GOA-ACAP-ARAP_08.05.2020_00h00	0,006973192	0,015555582	1,11787E-11	6,6327E-10	[4, 5]	Group191	5,73	13,00
GO:0045913	positive regulation of carbohydrate metabolic process	GO_BiologicalProcess-EBI-UniProt-GOA-ACAP-ARAP_08.05.2020_00h00	0,002461149	0,007094337	1,11787E-11	6,6327E-10	[3, 4, 5, 6]	Group191	8,89	8,00
GO:0048660	regulation of smooth muscle cell proliferation	GO_BiologicalProcess-EBI-UniProt-GOA-ACAP-ARAP_08.05.2020_00h00	0,000114782	0,000695231	1,11787E-11	6,6327E-10	[4, 5]	Group191	8,97	13,00
GO:0050678	regulation of epithelial cell proliferation	GO_BiologicalProcess-EBI-UniProt-GOA-ACAP-ARAP_08.05.2020_00h00	4,34628E-05	0,000310403	1,11787E-11	6,6327E-10	[4, 5]	Group191	6,50	24,00

GO:1903035	negative regulation of response to wounding	GO_BiologicalProcess-EBI-UniProt-GOA-ACAP-ARAP_08.05.2020_00h00	0,005007775	0,011891912	1,11787E-11	6,6327E-10	[3, 4, 5, 6]	Group191	7,92	8,00
GO:0010631	epithelial cell migration	GO_BiologicalProcess-EBI-UniProt-GOA-ACAP-ARAP_08.05.2020_00h00	0,00018162	0,00096028	1,11787E-11	6,6327E-10	[4, 5, 6]	Group191	6,51	20,00
GO:0010632	regulation of epithelial cell migration	GO_BiologicalProcess-EBI-UniProt-GOA-ACAP-ARAP_08.05.2020_00h00	0,000186103	0,000978573	1,11787E-11	6,6327E-10	[3, 4, 5, 6, 7]	Group191	7,14	17,00
GO:0030193	regulation of blood coagulation	GO_BiologicalProcess-EBI-UniProt-GOA-ACAP-ARAP_08.05.2020_00h00	0,000625023	0,002481938	1,11787E-11	6,6327E-10	[3, 4, 5, 6, 7]	Group191	9,89	9,00
GO:0043255	regulation of carbohydrate biosynthetic process	GO_BiologicalProcess-EBI-UniProt-GOA-ACAP-ARAP_08.05.2020_00h00	0,024403341	0,039853238	1,11787E-11	6,6327E-10	[4, 5, 6]	Group191	6,42	7,00
GO:0044344	cellular response to fibroblast growth factor stimulus	GO_BiologicalProcess-EBI-UniProt-GOA-ACAP-ARAP_08.05.2020_00h00	0,033316683	0,049818851	1,11787E-11	6,6327E-10	[4, 6]	Group191	5,63	8,00
GO:0048661	positive regulation of smooth muscle cell proliferation	GO_BiologicalProcess-EBI-UniProt-GOA-ACAP-ARAP_08.05.2020_00h00	0,00013222	0,000762259	1,11787E-11	6,6327E-10	[4, 5, 6]	Group191	10,99	10,00
GO:0050679	positive regulation of epithelial cell proliferation	GO_BiologicalProcess-EBI-UniProt-GOA-ACAP-ARAP_08.05.2020_00h00	0,00018895	0,000982745	1,11787E-11	6,6327E-10	[4, 5, 6]	Group191	7,41	16,00
GO:0090288	negative regulation of cellular response to growth factor stimulus	GO_BiologicalProcess-EBI-UniProt-GOA-ACAP-ARAP_08.05.2020_00h00	0,012154936	0,023885572	1,11787E-11	6,6327E-10	[3, 4, 5, 6, 7]	Group191	6,02	10,00
GO:0101023	vascular endothelial cell proliferation	GO_BiologicalProcess-EBI-UniProt-GOA-ACAP-ARAP_08.05.2020_00h00	0,026696119	0,042651396	1,11787E-11	6,6327E-10	[5]	Group191	12,00	3,00
GO:1990874	vascular smooth muscle cell proliferation	GO_BiologicalProcess-EBI-UniProt-GOA-ACAP-ARAP_08.05.2020_00h00	0,003044682	0,008138997	1,11787E-11	6,6327E-10	[5]	Group191	10,91	6,00
GO:0001936	regulation of endothelial cell proliferation	GO_BiologicalProcess-EBI-UniProt-GOA-ACAP-ARAP_08.05.2020_00h00	0,014143549	0,026282284	1,11787E-11	6,6327E-10	[5, 6]	Group191	6,21	9,00
GO:0010634	positive regulation of epithelial cell migration	GO_BiologicalProcess-EBI-UniProt-GOA-ACAP-ARAP_08.05.2020_00h00	0,000839522	0,003066498	1,11787E-11	6,6327E-10	[3, 4, 5, 6, 7, 8]	Group191	7,69	12,00
GO:0014909	smooth muscle cell migration	GO_BiologicalProcess-EBI-UniProt-GOA-ACAP-ARAP_08.05.2020_00h00	0,000144714	0,000819473	1,11787E-11	6,6327E-10	[5, 6]	Group191	12,00	9,00
GO:1904705	regulation of vascular smooth muscle cell proliferation	GO_BiologicalProcess-EBI-UniProt-GOA-ACAP-ARAP_08.05.2020_00h00	0,003044682	0,008138997	1,11787E-11	6,6327E-10	[5, 6]	Group191	10,91	6,00
GO:0001938	positive regulation of endothelial cell proliferation	GO_BiologicalProcess-EBI-UniProt-GOA-ACAP-ARAP_08.05.2020_00h00	0,02131371	0,036101274	1,11787E-11	6,6327E-10	[5, 6, 7]	Group191	6,60	7,00
GO:0010594	regulation of endothelial cell migration	GO_BiologicalProcess-EBI-UniProt-GOA-ACAP-ARAP_08.05.2020_00h00	0,000681572	0,002640746	1,11787E-11	6,6327E-10	[4, 5, 6, 7, 8]	Group191	7,47	13,00
GO:0014910	regulation of smooth muscle cell migration	GO_BiologicalProcess-EBI-UniProt-GOA-ACAP-ARAP_08.05.2020_00h00	0,000348865	0,001605114	1,11787E-11	6,6327E-10	[5, 6, 7]	Group191	11,94	8,00
GO:0043542	endothelial cell migration	GO_BiologicalProcess-EBI-UniProt-GOA-ACAP-ARAP_08.05.2020_00h00	0,000331147	0,001553468	1,11787E-11	6,6327E-10	[5, 6, 7]	Group191	7,05	16,00
GO:1901343	negative regulation of vasculature development	GO_BiologicalProcess-EBI-UniProt-GOA-ACAP-ARAP_08.05.2020_00h00	0,028789286	0,045166142	1,11787E-11	6,6327E-10	[3, 4, 5, 6, 7, 8, 9]	Group191	5,80	8,00
GO:1904707	positive regulation of vascular smooth muscle cell proliferation	GO_BiologicalProcess-EBI-UniProt-GOA-ACAP-ARAP_08.05.2020_00h00	0,015443421	0,028044315	1,11787E-11	6,6327E-10	[5, 6, 7]	Group191	10,81	4,00
GO:0010595	positive regulation of endothelial cell migration	GO_BiologicalProcess-EBI-UniProt-GOA-ACAP-ARAP_08.05.2020_00h00	0,00920515	0,019361163	1,11787E-11	6,6327E-10	[4, 5, 6, 7, 8, 9]	Group191	7,14	8,00
GO:0014911	positive regulation of smooth muscle cell migration	GO_BiologicalProcess-EBI-UniProt-GOA-ACAP-ARAP_08.05.2020_00h00	0,000943803	0,003345259	1,11787E-11	6,6327E-10	[5, 6, 7, 8]	Group191	13,64	6,00

GO:0030212	hyaluronan metabolic process	GO_BiologicalProcess-EBI-UniProt-GOA-ACAP-ARAP_08.05.2020_00h00	0,005625159	0,013066206	1,11787E-11	6,6327E-10	[6, 7]	Group191	11,36	5,00
GO:0042730	fibrinolysis	GO_BiologicalProcess-EBI-UniProt-GOA-ACAP-ARAP_08.05.2020_00h00	0,007393209	0,016265061	1,11787E-11	6,6327E-10	[4, 5, 6, 7, 8, 9]	Group191	13,33	4,00
GO:0040037	negative regulation of fibroblast growth factor receptor signaling pathway	GO_BiologicalProcess-EBI-UniProt-GOA-ACAP-ARAP_08.05.2020_00h00	0,012620698	0,024499003	1,11787E-11	6,6327E-10	[4, 5, 6, 7, 8, 9, 10]	Group191	15,79	3,00
GO:0043535	regulation of blood vessel endothelial cell migration	GO_BiologicalProcess-EBI-UniProt-GOA-ACAP-ARAP_08.05.2020_00h00	0,004171744	0,010316174	1,11787E-11	6,6327E-10	[5, 6, 7, 8, 9]	Group191	8,16	8,00
GO:0043550	regulation of lipid kinase activity	GO_BiologicalProcess-EBI-UniProt-GOA-ACAP-ARAP_08.05.2020_00h00	0,030797191	0,047081329	1,11787E-11	6,6327E-10	[5, 6, 7, 8, 9]	Group191	7,46	5,00
GO:0055024	regulation of cardiac muscle tissue development	GO_BiologicalProcess-EBI-UniProt-GOA-ACAP-ARAP_08.05.2020_00h00	0,008760029	0,018588355	1,11787E-11	6,6327E-10	[5, 6, 7, 8, 9]	Group191	7,87	7,00
GO:1905564	positive regulation of vascular endothelial cell proliferation	GO_BiologicalProcess-EBI-UniProt-GOA-ACAP-ARAP_08.05.2020_00h00	0,014564467	0,026855865	1,11787E-11	6,6327E-10	[6, 7, 8]	Group191	15,00	3,00
GO:0002040	sprouting angiogenesis	GO_BiologicalProcess-EBI-UniProt-GOA-ACAP-ARAP_08.05.2020_00h00	0,00041584	0,001833911	1,11787E-11	6,6327E-10	[4, 5, 6, 7, 8, 9, 10, 11]	Group191	8,87	11,00
GO:0031639	plasminogen activation	GO_BiologicalProcess-EBI-UniProt-GOA-ACAP-ARAP_08.05.2020_00h00	0,003242962	0,008549627	1,11787E-11	6,6327E-10	[7, 8]	Group191	16,67	4,00
GO:0043536	positive regulation of blood vessel endothelial cell migration	GO_BiologicalProcess-EBI-UniProt-GOA-ACAP-ARAP_08.05.2020_00h00	0,02016817	0,034589496	1,11787E-11	6,6327E-10	[5, 6, 7, 8, 9, 10]	Group191	8,33	5,00
GO:0002043	blood vessel endothelial cell proliferation involved in sprouting angiogenesis	GO_BiologicalProcess-EBI-UniProt-GOA-ACAP-ARAP_08.05.2020_00h00	0,00033793	0,001562316	1,11787E-11	6,6327E-10	[5, 6, 7, 8, 9, 10, 11, 12]	Group191	20,83	5,00
GO:0048583	regulation of response to stimulus	GO_BiologicalProcess-EBI-UniProt-GOA-ACAP-ARAP_08.05.2020_00h00	6,27191E-11	2,00074E-08	3,1323E-11	1,1151E-09	[2, 3]	Group133	4,02	182,00
GO:0033554	cellular response to stress	GO_BiologicalProcess-EBI-UniProt-GOA-ACAP-ARAP_08.05.2020_00h00	1,66634E-08	3,98672E-07	3,1323E-11	1,1151E-09	[3]	Group133	4,55	100,00
GO:0048585	negative regulation of response to stimulus	GO_BiologicalProcess-EBI-UniProt-GOA-ACAP-ARAP_08.05.2020_00h00	1,4974E-08	3,67438E-07	3,1323E-11	1,1151E-09	[2, 3, 4]	Group133	4,85	85,00
GO:0080134	regulation of response to stress	GO_BiologicalProcess-EBI-UniProt-GOA-ACAP-ARAP_08.05.2020_00h00	1,20431E-08	3,20146E-07	3,1323E-11	1,1151E-09	[3, 4]	Group133	5,05	78,00
GO:0006974	cellular response to DNA damage stimulus	GO_BiologicalProcess-EBI-UniProt-GOA-ACAP-ARAP_08.05.2020_00h00	0,001050609	0,003642874	3,1323E-11	1,1151E-09	[4]	Group133	4,38	40,00
GO:0080135	regulation of cellular response to stress	GO_BiologicalProcess-EBI-UniProt-GOA-ACAP-ARAP_08.05.2020_00h00	5,18829E-08	9,54845E-07	3,1323E-11	1,1151E-09	[3, 4, 5]	Group133	6,08	48,00
GO:2001020	regulation of response to DNA damage stimulus	GO_BiologicalProcess-EBI-UniProt-GOA-ACAP-ARAP_08.05.2020_00h00	0,004286675	0,010465174	3,1323E-11	1,1151E-09	[4, 5, 6]	Group133	5,86	14,00
GO:0000187	activation of MAPK activity	GO_BiologicalProcess-EBI-UniProt-GOA-ACAP-ARAP_08.05.2020_00h00	0,001091557	0,003744156	4,56071E-11	1,353E-09	[7, 8, 9, 10, 11, 12, 13]	Group193	6,80	14,00
GO:0023051	regulation of signaling	GO_BiologicalProcess-EBI-UniProt-GOA-ACAP-ARAP_08.05.2020_00h00	4,33256E-10	3,76933E-08	4,56071E-11	1,353E-09	[2, 3]	Group193	4,12	158,00
GO:0048583	regulation of response to stimulus	GO_BiologicalProcess-EBI-UniProt-GOA-ACAP-ARAP_08.05.2020_00h00	6,27191E-11	2,00074E-08	4,56071E-11	1,353E-09	[2, 3]	Group193	4,02	182,00
GO:0051239	regulation of multicellular organismal process	GO_BiologicalProcess-EBI-UniProt-GOA-ACAP-ARAP_08.05.2020_00h00	8,97701E-09	2,60333E-07	4,56071E-11	1,353E-09	[2, 3]	Group193	4,10	140,00
GO:0006793	phosphorus metabolic process	GO_BiologicalProcess-EBI-UniProt-GOA-ACAP-ARAP_08.05.2020_00h00	6,13427E-09	2,09661E-07	4,56071E-11	1,353E-09	[3]	Group193	4,09	144,00

GO:0010033	response to organic substance	GO_BiologicalProcess-EBI-UniProt-GOA-ACAP-ARAP_08.05.2020_00h00	4,09467E-08	7,68353E-07	4,56071E-11	1,353E-09	[3]	Group193	4,00	141,00
GO:0016772	transferase activity, transferring phosphorus-containing groups	GO_MolecularFunction-EBI-UniProt-GOA-ACAP-ARAP_08.05.2020_00h00	2,30551E-06	2,53606E-05	4,56071E-11	1,353E-09	[3]	Group193	4,42	79,00
GO:0023056	positive regulation of signaling	GO_BiologicalProcess-EBI-UniProt-GOA-ACAP-ARAP_08.05.2020_00h00	6,42911E-11	1,53817E-08	4,56071E-11	1,353E-09	[2, 3, 4]	Group193	5,00	102,00
GO:0023057	negative regulation of signaling	GO_BiologicalProcess-EBI-UniProt-GOA-ACAP-ARAP_08.05.2020_00h00	8,40763E-06	7,73663E-05	4,56071E-11	1,353E-09	[2, 3, 4]	Group193	4,48	68,00
GO:0044092	negative regulation of molecular function	GO_BiologicalProcess-EBI-UniProt-GOA-ACAP-ARAP_08.05.2020_00h00	0,001134739	0,003864575	4,56071E-11	1,353E-09	[3]	Group193	4,07	51,00
GO:0044093	positive regulation of molecular function	GO_BiologicalProcess-EBI-UniProt-GOA-ACAP-ARAP_08.05.2020_00h00	6,68997E-11	1,28046E-08	4,56071E-11	1,353E-09	[3]	Group193	5,07	99,00
GO:0048584	positive regulation of response to stimulus	GO_BiologicalProcess-EBI-UniProt-GOA-ACAP-ARAP_08.05.2020_00h00	2,07976E-07	3,15925E-06	4,56071E-11	1,353E-09	[2, 3, 4]	Group193	4,21	109,00
GO:0048585	negative regulation of response to stimulus	GO_BiologicalProcess-EBI-UniProt-GOA-ACAP-ARAP_08.05.2020_00h00	1,4974E-08	3,67438E-07	4,56071E-11	1,353E-09	[2, 3, 4]	Group193	4,85	85,00
GO:0050790	regulation of catalytic activity	GO_BiologicalProcess-EBI-UniProt-GOA-ACAP-ARAP_08.05.2020_00h00	1,121E-08	3,06514E-07	4,56071E-11	1,353E-09	[3]	Group193	4,41	112,00
GO:0051240	positive regulation of multicellular organismal process	GO_BiologicalProcess-EBI-UniProt-GOA-ACAP-ARAP_08.05.2020_00h00	3,52846E-07	5,03991E-06	4,56071E-11	1,353E-09	[2, 3, 4]	Group193	4,50	86,00
GO:0051241	negative regulation of multicellular organismal process	GO_BiologicalProcess-EBI-UniProt-GOA-ACAP-ARAP_08.05.2020_00h00	0,001074145	0,003697685	4,56071E-11	1,353E-09	[2, 3, 4]	Group193	4,04	53,00
GO:0010646	regulation of cell communication	GO_BiologicalProcess-EBI-UniProt-GOA-ACAP-ARAP_08.05.2020_00h00	3,57848E-10	3,4246E-08	4,56071E-11	1,353E-09	[3, 4]	Group193	4,14	157,00
GO:0010941	regulation of cell death	GO_BiologicalProcess-EBI-UniProt-GOA-ACAP-ARAP_08.05.2020_00h00	3,69996E-07	5,20716E-06	4,56071E-11	1,353E-09	[3, 4]	Group193	4,57	82,00
GO:0019637	organophosphate metabolic process	GO_BiologicalProcess-EBI-UniProt-GOA-ACAP-ARAP_08.05.2020_00h00	0,000611052	0,002436569	4,56071E-11	1,353E-09	[3, 4]	Group193	4,26	48,00
GO:0042127	regulation of cell population proliferation	GO_BiologicalProcess-EBI-UniProt-GOA-ACAP-ARAP_08.05.2020_00h00	9,02399E-10	5,75731E-08	4,56071E-11	1,353E-09	[3, 4]	Group193	5,05	89,00
GO:0080134	regulation of response to stress	GO_BiologicalProcess-EBI-UniProt-GOA-ACAP-ARAP_08.05.2020_00h00	1,20431E-08	3,20146E-07	4,56071E-11	1,353E-09	[3, 4]	Group193	5,05	78,00
GO:2000026	regulation of multicellular organismal development	GO_BiologicalProcess-EBI-UniProt-GOA-ACAP-ARAP_08.05.2020_00h00	3,43602E-09	1,42968E-07	4,56071E-11	1,353E-09	[3, 4]	Group193	4,64	103,00
GO:0006796	phosphate-containing compound metabolic process	GO_BiologicalProcess-EBI-UniProt-GOA-ACAP-ARAP_08.05.2020_00h00	2,32844E-08	5,06435E-07	4,56071E-11	1,353E-09	[4]	Group193	4,04	141,00
GO:0007166	cell surface receptor signaling pathway	GO_BiologicalProcess-EBI-UniProt-GOA-ACAP-ARAP_08.05.2020_00h00	2,092E-08	4,76678E-07	4,56071E-11	1,353E-09	[3, 4, 5]	Group193	4,08	137,00
GO:0009966	regulation of signal transduction	GO_BiologicalProcess-EBI-UniProt-GOA-ACAP-ARAP_08.05.2020_00h00	2,92397E-09	1,27193E-07	4,56071E-11	1,353E-09	[3, 4, 5]	Group193	4,19	138,00
GO:0010647	positive regulation of cell communication	GO_BiologicalProcess-EBI-UniProt-GOA-ACAP-ARAP_08.05.2020_00h00	1,14659E-10	1,56756E-08	4,56071E-11	1,353E-09	[3, 4, 5]	Group193	4,98	101,00
GO:0010648	negative regulation of cell communication	GO_BiologicalProcess-EBI-UniProt-GOA-ACAP-ARAP_08.05.2020_00h00	7,87514E-06	7,317E-05	4,56071E-11	1,353E-09	[3, 4, 5]	Group193	4,49	68,00
GO:0016773	phosphotransferase activity, alcohol group as acceptor	GO_MolecularFunction-EBI-UniProt-GOA-ACAP-ARAP_08.05.2020_00h00	3,23306E-05	0,000239848	4,56071E-11	1,353E-09	[4]	Group193	4,40	63,00

GO:0030234	enzyme regulator activity	GO_BiologicalProcess-EBI-UniProt-GOA-ACAP-ARAP_08.05.2020_00h00	0,000976118	0,003447031	4,56071E-11	1,353E-09	[4]	Group193	4,17	48,00
GO:0035556	intracellular signal transduction	GO_BiologicalProcess-EBI-UniProt-GOA-ACAP-ARAP_08.05.2020_00h00	1,26934E-09	6,39346E-08	4,56071E-11	1,353E-09	[3, 4, 5]	Group193	4,34	130,00
GO:0043085	positive regulation of catalytic activity	GO_BiologicalProcess-EBI-UniProt-GOA-ACAP-ARAP_08.05.2020_00h00	1,27347E-09	6,09358E-08	4,56071E-11	1,353E-09	[4]	Group193	5,20	82,00
GO:0043086	negative regulation of catalytic activity	GO_BiologicalProcess-EBI-UniProt-GOA-ACAP-ARAP_08.05.2020_00h00	0,003219853	0,008512152	4,56071E-11	1,353E-09	[4]	Group193	4,20	37,00
GO:0051336	regulation of hydrolase activity	GO_BiologicalProcess-EBI-UniProt-GOA-ACAP-ARAP_08.05.2020_00h00	8,71715E-06	7,94506E-05	4,56071E-11	1,353E-09	[4]	Group193	4,59	63,00
GO:0051338	regulation of transferase activity	GO_BiologicalProcess-EBI-UniProt-GOA-ACAP-ARAP_08.05.2020_00h00	2,2116E-07	3,30703E-06	4,56071E-11	1,353E-09	[4]	Group193	5,31	58,00
GO:0071310	cellular response to organic substance	GO_BiologicalProcess-EBI-UniProt-GOA-ACAP-ARAP_08.05.2020_00h00	3,0656E-08	6,24208E-07	4,56071E-11	1,353E-09	[4]	Group193	4,21	122,00
GO:0009967	positive regulation of signal transduction	GO_BiologicalProcess-EBI-UniProt-GOA-ACAP-ARAP_08.05.2020_00h00	4,99385E-09	1,91165E-07	4,56071E-11	1,353E-09	[3, 4, 5, 6]	Group193	4,92	87,00
GO:0009968	negative regulation of signal transduction	GO_BiologicalProcess-EBI-UniProt-GOA-ACAP-ARAP_08.05.2020_00h00	6,11198E-07	8,01255E-06	4,56071E-11	1,353E-09	[3, 4, 5, 6]	Group193	4,89	66,00
GO:0051246	regulation of protein metabolic process	GO_BiologicalProcess-EBI-UniProt-GOA-ACAP-ARAP_08.05.2020_00h00	1,25694E-08	3,25106E-07	4,56071E-11	1,353E-09	[4, 5]	Group193	4,21	127,00
GO:0007167	enzyme linked receptor protein signaling pathway	GO_BiologicalProcess-EBI-UniProt-GOA-ACAP-ARAP_08.05.2020_00h00	3,81246E-06	3,96579E-05	4,56071E-11	1,353E-09	[4, 5, 6]	Group193	4,96	55,00
GO:0008047	enzyme activator activity	GO_BiologicalProcess-EBI-UniProt-GOA-ACAP-ARAP_08.05.2020_00h00	0,000987177	0,003460544	4,56071E-11	1,353E-09	[5]	Group193	4,96	28,00
GO:0016310	phosphorylation	GO_BiologicalProcess-EBI-UniProt-GOA-ACAP-ARAP_08.05.2020_00h00	8,61421E-08	1,42135E-06	4,56071E-11	1,353E-09	[5]	Group193	4,27	110,00
GO:0031098	stress-activated protein kinase signaling cascade	GO_BiologicalProcess-EBI-UniProt-GOA-ACAP-ARAP_08.05.2020_00h00	0,011678319	0,023091221	4,56071E-11	1,353E-09	[4, 5, 6]	Group193	4,94	16,00
GO:0032268	regulation of cellular protein metabolic process	GO_BiologicalProcess-EBI-UniProt-GOA-ACAP-ARAP_08.05.2020_00h00	6,44939E-09	2,1283E-07	4,56071E-11	1,353E-09	[4, 5, 6]	Group193	4,32	122,00
GO:0043087	regulation of GTPase activity	GO_BiologicalProcess-EBI-UniProt-GOA-ACAP-ARAP_08.05.2020_00h00	0,000172834	0,000924034	4,56071E-11	1,353E-09	[5]	Group193	5,53	28,00
GO:0043491	protein kinase B signaling	GO_BiologicalProcess-EBI-UniProt-GOA-ACAP-ARAP_08.05.2020_00h00	7,17147E-05	0,000466877	4,56071E-11	1,353E-09	[4, 5, 6]	Group193	7,20	19,00
GO:0051247	positive regulation of protein metabolic process	GO_BiologicalProcess-EBI-UniProt-GOA-ACAP-ARAP_08.05.2020_00h00	6,97002E-09	2,15171E-07	4,56071E-11	1,353E-09	[4, 5, 6]	Group193	4,84	89,00
GO:0051248	negative regulation of protein metabolic process	GO_BiologicalProcess-EBI-UniProt-GOA-ACAP-ARAP_08.05.2020_00h00	0,000265156	0,001301303	4,56071E-11	1,353E-09	[4, 5, 6]	Group193	4,34	51,00
GO:0051345	positive regulation of hydrolase activity	GO_BiologicalProcess-EBI-UniProt-GOA-ACAP-ARAP_08.05.2020_00h00	9,69574E-06	8,7536E-05	4,56071E-11	1,353E-09	[5]	Group193	5,30	43,00
GO:0051347	positive regulation of transferase activity	GO_BiologicalProcess-EBI-UniProt-GOA-ACAP-ARAP_08.05.2020_00h00	1,65631E-06	1,93304E-05	4,56071E-11	1,353E-09	[5]	Group193	5,68	43,00
GO:0051348	negative regulation of transferase activity	GO_BiologicalProcess-EBI-UniProt-GOA-ACAP-ARAP_08.05.2020_00h00	0,005998993	0,013800568	4,56071E-11	1,353E-09	[5]	Group193	5,32	16,00
GO:1902531	regulation of intracellular signal transduction	GO_BiologicalProcess-EBI-UniProt-GOA-ACAP-ARAP_08.05.2020_00h00	7,94893E-07	1,01428E-05	4,56071E-11	1,353E-09	[4, 5, 6]	Group193	4,43	85,00

GO:0023014	signal transduction by protein phosphorylation	GO_BiologicalProcess-EBI-UniProt-GOA-ACAP-ARAP_08.05.2020_00h00	6,64101E-07	8,58844E-06	4,56071E-11	1,353E-09	[3, 4, 5, 7, 8]	Group193	5,34	53,00
GO:0019220	regulation of phosphate metabolic process	GO_BiologicalProcess-EBI-UniProt-GOA-ACAP-ARAP_08.05.2020_00h00	1,73471E-08	4,04906E-07	4,56071E-11	1,353E-09	[5, 6]	Group193	4,71	91,00
GO:0032269	negative regulation of cellular protein metabolic process	GO_BiologicalProcess-EBI-UniProt-GOA-ACAP-ARAP_08.05.2020_00h00	0,00020134	0,001035927	4,56071E-11	1,353E-09	[4, 5, 6, 7]	Group193	4,45	49,00
GO:0032270	positive regulation of cellular protein metabolic process	GO_BiologicalProcess-EBI-UniProt-GOA-ACAP-ARAP_08.05.2020_00h00	5,27485E-09	1,94155E-07	4,56071E-11	1,353E-09	[4, 5, 6, 7]	Group193	4,96	85,00
GO:0042981	regulation of apoptotic process	GO_BiologicalProcess-EBI-UniProt-GOA-ACAP-ARAP_08.05.2020_00h00	1,11326E-06	1,33174E-05	4,56071E-11	1,353E-09	[5, 6]	Group193	4,59	75,00
GO:1902532	negative regulation of intracellular signal transduction	GO_BiologicalProcess-EBI-UniProt-GOA-ACAP-ARAP_08.05.2020_00h00	4,25172E-05	0,000305932	4,56071E-11	1,353E-09	[4, 5, 6, 7]	Group193	5,72	31,00
GO:1902533	positive regulation of intracellular signal transduction	GO_BiologicalProcess-EBI-UniProt-GOA-ACAP-ARAP_08.05.2020_00h00	3,51826E-06	3,69997E-05	4,56071E-11	1,353E-09	[4, 5, 6, 7]	Group193	4,97	55,00
GO:0016301	kinase activity	GO_BiologicalProcess-EBI-UniProt-GOA-ACAP-ARAP_08.05.2020_00h00	5,86806E-06	5,78942E-05	4,56071E-11	1,353E-09	[6]	Group193	4,48	70,00
GO:0031399	regulation of protein modification process	GO_BiologicalProcess-EBI-UniProt-GOA-ACAP-ARAP_08.05.2020_00h00	1,07623E-07	1,68845E-06	4,56071E-11	1,353E-09	[5, 6, 7]	Group193	4,55	90,00
GO:0043547	positive regulation of GTPase activity	GO_BiologicalProcess-EBI-UniProt-GOA-ACAP-ARAP_08.05.2020_00h00	0,000437988	0,001896626	4,56071E-11	1,353E-09	[6]	Group193	5,58	24,00
GO:0045936	negative regulation of phosphate metabolic process	GO_BiologicalProcess-EBI-UniProt-GOA-ACAP-ARAP_08.05.2020_00h00	0,000540563	0,002239477	4,56071E-11	1,353E-09	[5, 6, 7]	Group193	5,02	30,00
GO:0045937	positive regulation of phosphate metabolic process	GO_BiologicalProcess-EBI-UniProt-GOA-ACAP-ARAP_08.05.2020_00h00	1,12601E-09	6,73495E-08	4,56071E-11	1,353E-09	[5, 6, 7]	Group193	5,60	70,00
GO:0051896	regulation of protein kinase B signaling	GO_BiologicalProcess-EBI-UniProt-GOA-ACAP-ARAP_08.05.2020_00h00	0,000195626	0,001011969	4,56071E-11	1,353E-09	[5, 6, 7]	Group193	7,11	17,00
GO:0000165	MAPK cascade	GO_BiologicalProcess-EBI-UniProt-GOA-ACAP-ARAP_08.05.2020_00h00	1,91283E-06	2,15362E-05	4,56071E-11	1,353E-09	[4, 5, 6, 8, 9]	Group193	5,24	51,00
GO:0006468	protein phosphorylation	GO_BiologicalProcess-EBI-UniProt-GOA-ACAP-ARAP_08.05.2020_00h00	3,55363E-08	6,80165E-07	4,56071E-11	1,353E-09	[6, 7]	Group193	4,55	96,00
GO:0031401	positive regulation of protein modification process	GO_BiologicalProcess-EBI-UniProt-GOA-ACAP-ARAP_08.05.2020_00h00	7,84556E-08	1,34075E-06	4,56071E-11	1,353E-09	[5, 6, 7, 8]	Group193	5,11	68,00
GO:0042325	regulation of phosphorylation	GO_BiologicalProcess-EBI-UniProt-GOA-ACAP-ARAP_08.05.2020_00h00	6,23797E-08	1,10551E-06	4,56071E-11	1,353E-09	[6, 7]	Group193	4,77	82,00
GO:0051897	positive regulation of protein kinase B signaling	GO_BiologicalProcess-EBI-UniProt-GOA-ACAP-ARAP_08.05.2020_00h00	8,10058E-05	0,000506683	4,56071E-11	1,353E-09	[5, 6, 7, 8]	Group193	8,33	15,00
GO:0043549	regulation of kinase activity	GO_BiologicalProcess-EBI-UniProt-GOA-ACAP-ARAP_08.05.2020_00h00	1,8027E-06	2,05379E-05	4,56071E-11	1,353E-09	[5, 7, 8]	Group193	5,25	51,00
GO:0001932	regulation of protein phosphorylation	GO_BiologicalProcess-EBI-UniProt-GOA-ACAP-ARAP_08.05.2020_00h00	1,20031E-07	1,85273E-06	4,56071E-11	1,353E-09	[6, 7, 8]	Group193	4,86	75,00
GO:0042326	negative regulation of phosphorylation	GO_BiologicalProcess-EBI-UniProt-GOA-ACAP-ARAP_08.05.2020_00h00	0,001627748	0,005192518	4,56071E-11	1,353E-09	[6, 7, 8]	Group193	5,07	24,00
GO:0042327	positive regulation of phosphorylation	GO_BiologicalProcess-EBI-UniProt-GOA-ACAP-ARAP_08.05.2020_00h00	3,4551E-08	6,74801E-07	4,56071E-11	1,353E-09	[6, 7, 8]	Group193	5,40	63,00
GO:0004707	MAP kinase activity	GO_BiologicalProcess-EBI-UniProt-GOA-ACAP-ARAP_08.05.2020_00h00	0,004993279	0,011916628	4,56071E-11	1,353E-09	[5, 6, 7, 9, 10]	Group193	5,05	19,00

GO:0070371	ERK1 and ERK2 cascade	GO_BiologicalProcess-EBI-UniProt-GOA-ACAP-ARAP_08.05.2020_00h00	7,46413E-05	0,000479408	4,56071E-11	1,353E-09	[5, 6, 7, 9, 10]	Group193	6,59	22,00
GO:0001933	negative regulation of protein phosphorylation	GO_BiologicalProcess-EBI-UniProt-GOA-ACAP-ARAP_08.05.2020_00h00	0,004805825	0,011555714	4,56071E-11	1,353E-09	[6, 7, 8, 9]	Group193	4,88	21,00
GO:0001934	positive regulation of protein phosphorylation	GO_BiologicalProcess-EBI-UniProt-GOA-ACAP-ARAP_08.05.2020_00h00	4,44036E-07	6,07061E-06	4,56071E-11	1,353E-09	[6, 7, 8, 9]	Group193	5,24	57,00
GO:0004672	protein kinase activity	GO_BiologicalProcess-EBI-UniProt-GOA-ACAP-ARAP_08.05.2020_00h00	4,63941E-05	0,000324081	4,56071E-11	1,353E-09	[7, 8]	Group193	4,46	58,00
GO:0004693	cyclin-dependent protein serine/threonine kinase activity	GO_BiologicalProcess-EBI-UniProt-GOA-ACAP-ARAP_08.05.2020_00h00	0,000545112	0,002248586	4,56071E-11	1,353E-09	[5, 6, 9, 10]	Group193	8,59	11,00
GO:0018209	peptidyl-serine modification	GO_BiologicalProcess-EBI-UniProt-GOA-ACAP-ARAP_08.05.2020_00h00	0,000275328	0,001337508	4,56071E-11	1,353E-09	[7, 8]	Group193	6,01	22,00
GO:0018212	peptidyl-tyrosine modification	GO_BiologicalProcess-EBI-UniProt-GOA-ACAP-ARAP_08.05.2020_00h00	0,00348192	0,00900594	4,56071E-11	1,353E-09	[7, 8]	Group193	5,02	21,00
GO:0033674	positive regulation of kinase activity	GO_BiologicalProcess-EBI-UniProt-GOA-ACAP-ARAP_08.05.2020_00h00	1,62283E-05	0,000136232	4,56071E-11	1,353E-09	[6, 7, 8, 9]	Group193	5,53	37,00
GO:0043408	regulation of MAPK cascade	GO_BiologicalProcess-EBI-UniProt-GOA-ACAP-ARAP_08.05.2020_00h00	3,12878E-06	3,36432E-05	4,56071E-11	1,353E-09	[5, 6, 7, 8, 9, 10]	Group193	5,54	43,00
GO:0045859	regulation of protein kinase activity	GO_BiologicalProcess-EBI-UniProt-GOA-ACAP-ARAP_08.05.2020_00h00	1,77981E-05	0,000148111	4,56071E-11	1,353E-09	[6, 7, 8, 9]	Group193	5,12	44,00
GO:0000079	regulation of cyclin-dependent protein serine/threonine kinase activity	GO_BiologicalProcess-EBI-UniProt-GOA-ACAP-ARAP_08.05.2020_00h00	0,001520331	0,004948833	4,56071E-11	1,353E-09	[5, 6, 7, 8, 9, 10, 11]	Group193	8,74	9,00
GO:0018105	peptidyl-serine phosphorylation	GO_BiologicalProcess-EBI-UniProt-GOA-ACAP-ARAP_08.05.2020_00h00	0,000650843	0,00255269	4,56071E-11	1,353E-09	[7, 8, 9]	Group193	5,90	20,00
GO:0043410	positive regulation of MAPK cascade	GO_BiologicalProcess-EBI-UniProt-GOA-ACAP-ARAP_08.05.2020_00h00	2,28998E-07	3,37156E-06	4,56071E-11	1,353E-09	[5, 6, 7, 8, 9, 10, 11]	Group193	6,54	38,00
GO:0004674	protein serine/threonine kinase activity	GO_BiologicalProcess-EBI-UniProt-GOA-ACAP-ARAP_08.05.2020_00h00	7,87337E-05	0,000495711	4,56071E-11	1,353E-09	[8, 9]	Group193	4,80	44,00
GO:0006469	negative regulation of protein kinase activity	GO_BiologicalProcess-EBI-UniProt-GOA-ACAP-ARAP_08.05.2020_00h00	0,012703408	0,02460964	4,56071E-11	1,353E-09	[7, 8, 9, 10]	Group193	5,31	13,00
GO:0043405	regulation of MAP kinase activity	GO_BiologicalProcess-EBI-UniProt-GOA-ACAP-ARAP_08.05.2020_00h00	0,00374708	0,009486654	4,56071E-11	1,353E-09	[6, 7, 8, 9, 10, 11]	Group193	5,19	19,00
GO:0045860	positive regulation of protein kinase activity	GO_BiologicalProcess-EBI-UniProt-GOA-ACAP-ARAP_08.05.2020_00h00	9,04722E-05	0,00056222	4,56071E-11	1,353E-09	[7, 8, 9, 10]	Group193	5,41	32,00
GO:0070372	regulation of ERK1 and ERK2 cascade	GO_BiologicalProcess-EBI-UniProt-GOA-ACAP-ARAP_08.05.2020_00h00	0,001373574	0,004564272	4,56071E-11	1,353E-09	[6, 7, 8, 9, 10, 11]	Group193	5,83	18,00
GO:0071900	regulation of protein serine/threonine kinase activity	GO_BiologicalProcess-EBI-UniProt-GOA-ACAP-ARAP_08.05.2020_00h00	6,17963E-05	0,00041356	4,56071E-11	1,353E-09	[7, 8, 9, 10]	Group193	5,61	31,00
GO:0070374	positive regulation of ERK1 and ERK2 cascade	GO_BiologicalProcess-EBI-UniProt-GOA-ACAP-ARAP_08.05.2020_00h00	0,005401376	0,012638426	4,56071E-11	1,353E-09	[6, 7, 8, 9, 10, 11, 12]	Group193	5,91	13,00
GO:0032147	activation of protein kinase activity	GO_BiologicalProcess-EBI-UniProt-GOA-ACAP-ARAP_08.05.2020_00h00	2,21901E-05	0,000176966	4,56071E-11	1,353E-09	[8, 9, 10, 11]	Group193	6,48	26,00
GO:0071902	positive regulation of protein serine/threonine kinase activity	GO_BiologicalProcess-EBI-UniProt-GOA-ACAP-ARAP_08.05.2020_00h00	0,000370646	0,00168108	4,56071E-11	1,353E-09	[8, 9, 10, 11]	Group193	5,88	22,00
GO:0032879	regulation of localization	GO_BiologicalProcess-EBI-UniProt-GOA-ACAP-ARAP_08.05.2020_00h00	1,28192E-10	1,5335E-08	6,27813E-11	1,3969E-09	[2, 3]	Group148	4,45	133,00

GO:0040012	regulation of locomotion	GO_BiologicalProcess-EBI-UniProt-GOA-ACAP-ARAP_08.05.2020_00h00	1,89996E-12	1,81826E-09	6,27813E-11	1,3969E-09	[2, 3]	Group148	6,58	68,00
GO:0048870	cell motility	GO_BiologicalProcess-EBI-UniProt-GOA-ACAP-ARAP_08.05.2020_00h00	1,18486E-09	6,29948E-08	6,27813E-11	1,3969E-09	[2, 3]	Group148	5,00	90,00
GO:0051239	regulation of multicellular organismal process	GO_BiologicalProcess-EBI-UniProt-GOA-ACAP-ARAP_08.05.2020_00h00	8,97701E-09	2,60333E-07	6,27813E-11	1,3969E-09	[2, 3]	Group148	4,10	140,00
GO:0032880	regulation of protein localization	GO_BiologicalProcess-EBI-UniProt-GOA-ACAP-ARAP_08.05.2020_00h00	0,001417178	0,004660617	6,27813E-11	1,3969E-09	[3, 4]	Group148	4,12	47,00
GO:0051049	regulation of transport	GO_BiologicalProcess-EBI-UniProt-GOA-ACAP-ARAP_08.05.2020_00h00	6,30185E-05	0,00041881	6,27813E-11	1,3969E-09	[3, 4]	Group148	4,00	81,00
GO:0051050	positive regulation of transport	GO_BiologicalProcess-EBI-UniProt-GOA-ACAP-ARAP_08.05.2020_00h00	0,001033711	0,003597314	6,27813E-11	1,3969E-09	[2, 3, 4, 5]	Group148	4,20	46,00
GO:0051270	regulation of cellular component movement	GO_BiologicalProcess-EBI-UniProt-GOA-ACAP-ARAP_08.05.2020_00h00	4,42024E-11	2,11509E-08	6,27813E-11	1,3969E-09	[3, 4]	Group148	6,18	67,00
GO:0060341	regulation of cellular localization	GO_BiologicalProcess-EBI-UniProt-GOA-ACAP-ARAP_08.05.2020_00h00	0,000142641	0,000812543	6,27813E-11	1,3969E-09	[3, 4]	Group148	4,51	49,00
GO:0040013	negative regulation of locomotion	GO_BiologicalProcess-EBI-UniProt-GOA-ACAP-ARAP_08.05.2020_00h00	0,002997997	0,008104755	5,61565E-11	1,428E-09	[2, 3, 4]	Group082	5,42	18,00
GO:0051270	regulation of cellular component movement	GO_BiologicalProcess-EBI-UniProt-GOA-ACAP-ARAP_08.05.2020_00h00	4,42024E-11	2,11509E-08	5,61565E-11	1,428E-09	[3, 4]	Group082	6,18	67,00
GO:0051271	negative regulation of cellular component movement	GO_BiologicalProcess-EBI-UniProt-GOA-ACAP-ARAP_08.05.2020_00h00	0,001396025	0,004606881	5,61565E-11	1,428E-09	[3, 4, 5]	Group082	5,67	19,00
GO:0030336	negative regulation of cell migration	GO_BiologicalProcess-EBI-UniProt-GOA-ACAP-ARAP_08.05.2020_00h00	0,001381399	0,00457439	5,61565E-11	1,428E-09	[4, 5, 6, 7]	Group082	5,99	17,00
GO:0002294	CD4-positive, alpha-beta T cell differentiation involved in immune response	GO_BiologicalProcess-EBI-UniProt-GOA-ACAP-ARAP_08.05.2020_00h00	0,02291313	0,038135417	7,38011E-11	1,4596E-09	[8, 9, 10, 11, 12, 13]	Group190	8,06	5,00
GO:0040012	regulation of locomotion	GO_BiologicalProcess-EBI-UniProt-GOA-ACAP-ARAP_08.05.2020_00h00	1,89996E-12	1,81826E-09	7,38011E-11	1,4596E-09	[2, 3]	Group190	6,58	68,00
GO:0045321	leukocyte activation	GO_BiologicalProcess-EBI-UniProt-GOA-ACAP-ARAP_08.05.2020_00h00	7,48312E-05	0,000477423	7,38011E-11	1,4596E-09	[2, 3]	Group190	4,32	61,00
GO:0048870	cell motility	GO_BiologicalProcess-EBI-UniProt-GOA-ACAP-ARAP_08.05.2020_00h00	1,18486E-09	6,29948E-08	7,38011E-11	1,4596E-09	[2, 3]	Group190	5,00	90,00
GO:0031589	cell-substrate adhesion	GO_BiologicalProcess-EBI-UniProt-GOA-ACAP-ARAP_08.05.2020_00h00	3,26496E-10	3,47175E-08	7,38011E-11	1,4596E-09	[3]	Group190	9,07	34,00
GO:0051241	negative regulation of multicellular organismal process	GO_BiologicalProcess-EBI-UniProt-GOA-ACAP-ARAP_08.05.2020_00h00	0,001074145	0,003697685	7,38011E-11	1,4596E-09	[2, 3, 4]	Group190	4,04	53,00
GO:0098609	cell-cell adhesion	GO_BiologicalProcess-EBI-UniProt-GOA-ACAP-ARAP_08.05.2020_00h00	3,39098E-08	6,76077E-07	7,38011E-11	1,4596E-09	[3]	Group190	5,86	53,00
GO:0002262	myeloid cell homeostasis	GO_BiologicalProcess-EBI-UniProt-GOA-ACAP-ARAP_08.05.2020_00h00	0,000130379	0,000760808	7,38011E-11	1,4596E-09	[2, 5]	Group190	8,38	14,00
GO:0030155	regulation of cell adhesion	GO_BiologicalProcess-EBI-UniProt-GOA-ACAP-ARAP_08.05.2020_00h00	1,32928E-08	3,34768E-07	7,38011E-11	1,4596E-09	[3, 4]	Group190	6,36	48,00
GO:0045595	regulation of cell differentiation	GO_BiologicalProcess-EBI-UniProt-GOA-ACAP-ARAP_08.05.2020_00h00	5,09874E-05	0,000353586	7,38011E-11	1,4596E-09	[3, 4]	Group190	4,04	80,00
GO:0046649	lymphocyte activation	GO_BiologicalProcess-EBI-UniProt-GOA-ACAP-ARAP_08.05.2020_00h00	0,001648926	0,0052426	7,38011E-11	1,4596E-09	[3, 4]	Group190	4,41	36,00

GO:0051270	regulation of cellular component movement	GO_BiologicalProcess-EBI-UniProt-GOA-ACAP-ARAP_08.05.2020_00h00	4,42024E-11	2,11509E-08	7,38011E-11	1,4596E-09	[3, 4]	Group190	6,18	67,00
GO:0002520	immune system development	GO_BiologicalProcess-EBI-UniProt-GOA-ACAP-ARAP_08.05.2020_00h00	5,84203E-07	7,76503E-06	7,38011E-11	1,4596E-09	[2, 4, 5]	Group190	5,23	56,00
GO:0007159	leukocyte cell-cell adhesion	GO_BiologicalProcess-EBI-UniProt-GOA-ACAP-ARAP_08.05.2020_00h00	0,000792809	0,002952212	7,38011E-11	1,4596E-09	[4]	Group190	5,68	21,00
GO:0007160	cell-matrix adhesion	GO_BiologicalProcess-EBI-UniProt-GOA-ACAP-ARAP_08.05.2020_00h00	2,11574E-08	4,70876E-07	7,38011E-11	1,4596E-09	[4]	Group190	10,00	24,00
GO:0007162	negative regulation of cell adhesion	GO_BiologicalProcess-EBI-UniProt-GOA-ACAP-ARAP_08.05.2020_00h00	0,000116516	0,000701296	7,38011E-11	1,4596E-09	[3, 4, 5]	Group190	6,73	20,00
GO:0045785	positive regulation of cell adhesion	GO_BiologicalProcess-EBI-UniProt-GOA-ACAP-ARAP_08.05.2020_00h00	2,56997E-05	0,000201595	7,38011E-11	1,4596E-09	[3, 4, 5]	Group190	6,18	28,00
GO:0048872	homeostasis of number of cells	GO_BiologicalProcess-EBI-UniProt-GOA-ACAP-ARAP_08.05.2020_00h00	0,00023294	0,001161058	7,38011E-11	1,4596E-09	[4]	Group190	6,57	19,00
GO:0050866	negative regulation of cell activation	GO_BiologicalProcess-EBI-UniProt-GOA-ACAP-ARAP_08.05.2020_00h00	0,024882277	0,040497176	7,38011E-11	1,4596E-09	[3, 4, 5]	Group190	5,16	11,00
GO:0002285	lymphocyte activation involved in immune response	GO_BiologicalProcess-EBI-UniProt-GOA-ACAP-ARAP_08.05.2020_00h00	0,004762104	0,01147943	7,38011E-11	1,4596E-09	[4, 5]	Group190	6,25	12,00
GO:0010810	regulation of cell-substrate adhesion	GO_BiologicalProcess-EBI-UniProt-GOA-ACAP-ARAP_08.05.2020_00h00	3,73914E-05	0,000273157	7,38011E-11	1,4596E-09	[4, 5]	Group190	7,83	18,00
GO:0022407	regulation of cell-cell adhesion	GO_BiologicalProcess-EBI-UniProt-GOA-ACAP-ARAP_08.05.2020_00h00	9,88883E-08	1,57727E-06	7,38011E-11	1,4596E-09	[4, 5]	Group190	7,37	33,00
GO:0034101	erythrocyte homeostasis	GO_BiologicalProcess-EBI-UniProt-GOA-ACAP-ARAP_08.05.2020_00h00	0,000242113	0,00120053	7,38011E-11	1,4596E-09	[3, 6]	Group190	8,82	12,00
GO:0042110	T cell activation	GO_BiologicalProcess-EBI-UniProt-GOA-ACAP-ARAP_08.05.2020_00h00	0,002095667	0,006306772	7,38011E-11	1,4596E-09	[4, 5]	Group190	4,90	25,00
GO:0048534	hematopoietic or lymphoid organ development	GO_BiologicalProcess-EBI-UniProt-GOA-ACAP-ARAP_08.05.2020_00h00	2,0816E-05	0,000167403	7,38011E-11	1,4596E-09	[3, 4, 5, 6]	Group190	4,88	49,00
GO:0010811	positive regulation of cell-substrate adhesion	GO_BiologicalProcess-EBI-UniProt-GOA-ACAP-ARAP_08.05.2020_00h00	0,002776	0,007700383	7,38011E-11	1,4596E-09	[4, 5, 6]	Group190	7,46	10,00
GO:0022408	negative regulation of cell-cell adhesion	GO_BiologicalProcess-EBI-UniProt-GOA-ACAP-ARAP_08.05.2020_00h00	0,000468324	0,001983124	7,38011E-11	1,4596E-09	[4, 5, 6]	Group190	7,41	14,00
GO:0022409	positive regulation of cell-cell adhesion	GO_BiologicalProcess-EBI-UniProt-GOA-ACAP-ARAP_08.05.2020_00h00	0,000265458	0,001296139	7,38011E-11	1,4596E-09	[4, 5, 6]	Group190	6,51	19,00
GO:0051249	regulation of lymphocyte activation	GO_BiologicalProcess-EBI-UniProt-GOA-ACAP-ARAP_08.05.2020_00h00	0,021119829	0,035836305	7,38011E-11	1,4596E-09	[4, 5, 6]	Group190	4,14	23,00
GO:0030097	hemopoiesis	GO_BiologicalProcess-EBI-UniProt-GOA-ACAP-ARAP_08.05.2020_00h00	2,93906E-05	0,000225015	7,38011E-11	1,4596E-09	[4, 5, 6, 7]	Group190	4,89	47,00
GO:0051250	negative regulation of lymphocyte activation	GO_BiologicalProcess-EBI-UniProt-GOA-ACAP-ARAP_08.05.2020_00h00	0,02252461	0,037685405	7,38011E-11	1,4596E-09	[4, 5, 6, 7]	Group190	5,73	9,00
GO:1903037	regulation of leukocyte cell-cell adhesion	GO_BiologicalProcess-EBI-UniProt-GOA-ACAP-ARAP_08.05.2020_00h00	0,000205795	0,001042039	7,38011E-11	1,4596E-09	[5, 6]	Group190	6,29	21,00
GO:1903706	regulation of hemopoiesis	GO_BiologicalProcess-EBI-UniProt-GOA-ACAP-ARAP_08.05.2020_00h00	0,007959014	0,017271601	7,38011E-11	1,4596E-09	[3, 4, 5, 6, 7, 8]	Group190	4,53	23,00
GO:0002521	leukocyte differentiation	GO_BiologicalProcess-EBI-UniProt-GOA-ACAP-ARAP_08.05.2020_00h00	0,00341803	0,008912955	7,38011E-11	1,4596E-09	[4, 5, 6, 7, 8]	Group190	4,66	26,00

GO:0030099	myeloid cell differentiation	GO_BiologicalProcess-EBI-UniProt-GOA-ACAP-ARAP_08.05.2020_00h00	6,2456E-06	5,97704E-05	7,38011E-11	1,4596E-09	[4, 5, 6, 7, 8]	Group190	6,55	29,00
GO:0050863	regulation of T cell activation	GO_BiologicalProcess-EBI-UniProt-GOA-ACAP-ARAP_08.05.2020_00h00	0,011387548	0,022656724	7,38011E-11	1,4596E-09	[5, 6, 7]	Group190	4,84	17,00
GO:1903038	negative regulation of leukocyte cell-cell adhesion	GO_BiologicalProcess-EBI-UniProt-GOA-ACAP-ARAP_08.05.2020_00h00	0,023751667	0,039055575	7,38011E-11	1,4596E-09	[5, 6, 7]	Group190	6,02	8,00
GO:1903039	positive regulation of leukocyte cell-cell adhesion	GO_BiologicalProcess-EBI-UniProt-GOA-ACAP-ARAP_08.05.2020_00h00	0,005321251	0,012512132	7,38011E-11	1,4596E-09	[5, 6, 7]	Group190	5,71	14,00
GO:1903556	negative regulation of tumor necrosis factor superfamily cytokine production	GO_BiologicalProcess-EBI-UniProt-GOA-ACAP-ARAP_08.05.2020_00h00	0,029100992	0,045283981	7,38011E-11	1,4596E-09	[4, 5, 6, 7, 8]	Group190	7,58	5,00
GO:0030098	lymphocyte differentiation	GO_BiologicalProcess-EBI-UniProt-GOA-ACAP-ARAP_08.05.2020_00h00	0,005579029	0,013022271	7,38011E-11	1,4596E-09	[4, 5, 6, 7, 8, 9]	Group190	5,00	19,00
GO:0030218	erythrocyte differentiation	GO_BiologicalProcess-EBI-UniProt-GOA-ACAP-ARAP_08.05.2020_00h00	0,001763233	0,005550702	7,38011E-11	1,4596E-09	[4, 5, 6, 7, 8, 9]	Group190	7,94	10,00
GO:0045637	regulation of myeloid cell differentiation	GO_BiologicalProcess-EBI-UniProt-GOA-ACAP-ARAP_08.05.2020_00h00	0,023358641	0,038541757	7,38011E-11	1,4596E-09	[4, 5, 6, 7, 8, 9]	Group190	4,89	13,00
GO:0061515	myeloid cell development	GO_BiologicalProcess-EBI-UniProt-GOA-ACAP-ARAP_08.05.2020_00h00	0,000976749	0,003436575	7,38011E-11	1,4596E-09	[4, 5, 6, 7, 8, 9]	Group190	10,26	8,00
GO:0002573	myeloid leukocyte differentiation	GO_BiologicalProcess-EBI-UniProt-GOA-ACAP-ARAP_08.05.2020_00h00	0,002206373	0,00653715	7,38011E-11	1,4596E-09	[5, 6, 7, 8, 9]	Group190	6,31	14,00
GO:0030183	B cell differentiation	GO_BiologicalProcess-EBI-UniProt-GOA-ACAP-ARAP_08.05.2020_00h00	0,029878801	0,04604511	7,38011E-11	1,4596E-09	[5, 6, 7, 8, 9, 10]	Group190	5,76	8,00
GO:0030316	osteoclast differentiation	GO_BiologicalProcess-EBI-UniProt-GOA-ACAP-ARAP_08.05.2020_00h00	0,02034726	0,034772012	7,38011E-11	1,4596E-09	[6, 7, 8, 9, 10]	Group190	6,67	7,00
GO:0050860	negative regulation of T cell receptor signaling pathway	GO_BiologicalProcess-EBI-UniProt-GOA-ACAP-ARAP_08.05.2020_00h00	0,00033793	0,001562316	7,38011E-11	1,4596E-09	[4, 5, 6, 7, 8, 9, 10, 11, 12]	Group190	20,83	5,00
GO:0044092	negative regulation of molecular function	GO_BiologicalProcess-EBI-UniProt-GOA-ACAP-ARAP_08.05.2020_00h00	0,001134739	0,003864575	8,69098E-11	1,547E-09	[3]	Group188	4,07	51,00
GO:0050790	regulation of catalytic activity	GO_BiologicalProcess-EBI-UniProt-GOA-ACAP-ARAP_08.05.2020_00h00	1,121E-08	3,06514E-07	8,69098E-11	1,547E-09	[3]	Group188	4,41	112,00
GO:0016773	phosphotransferase activity, alcohol group as acceptor	GO_MolecularFunction-EBI-UniProt-GOA-ACAP-ARAP_08.05.2020_00h00	3,23306E-05	0,000239848	8,69098E-11	1,547E-09	[4]	Group188	4,40	63,00
GO:0019900	kinase binding	GO_MolecularFunction-EBI-UniProt-GOA-ACAP-ARAP_08.05.2020_00h00	0,00272406	0,007600365	8,69098E-11	1,547E-09	[4]	Group188	4,35	34,00
GO:0030234	enzyme regulator activity	GO_BiologicalProcess-EBI-UniProt-GOA-ACAP-ARAP_08.05.2020_00h00	0,000976118	0,003447031	8,69098E-11	1,547E-09	[4]	Group188	4,17	48,00
GO:0043086	negative regulation of catalytic activity	GO_BiologicalProcess-EBI-UniProt-GOA-ACAP-ARAP_08.05.2020_00h00	0,003219853	0,008512152	8,69098E-11	1,547E-09	[4]	Group188	4,20	37,00
GO:0051336	regulation of hydrolase activity	GO_BiologicalProcess-EBI-UniProt-GOA-ACAP-ARAP_08.05.2020_00h00	8,71715E-06	7,94506E-05	8,69098E-11	1,547E-09	[4]	Group188	4,59	63,00
GO:0051338	regulation of transferase activity	GO_BiologicalProcess-EBI-UniProt-GOA-ACAP-ARAP_08.05.2020_00h00	2,2116E-07	3,30703E-06	8,69098E-11	1,547E-09	[4]	Group188	5,31	58,00
GO:0051246	regulation of protein metabolic process	GO_BiologicalProcess-EBI-UniProt-GOA-ACAP-ARAP_08.05.2020_00h00	1,25694E-08	3,25106E-07	8,69098E-11	1,547E-09	[4, 5]	Group188	4,21	127,00
GO:0032268	regulation of cellular protein metabolic process	GO_BiologicalProcess-EBI-UniProt-GOA-ACAP-ARAP_08.05.2020_00h00	6,44939E-09	2,1283E-07	8,69098E-11	1,547E-09	[4, 5, 6]	Group188	4,32	122,00

GO:0051248	negative regulation of protein metabolic process	GO_BiologicalProcess-EBI-UniProt-GOA-ACAP-ARAP_08.05.2020_00h00	0,000265156	0,001301303	8,69098E-11	1,547E-09	[4, 5, 6]	Group188	4,34	51,00
GO:0051348	negative regulation of transferase activity	GO_BiologicalProcess-EBI-UniProt-GOA-ACAP-ARAP_08.05.2020_00h00	0,005998993	0,013800568	8,69098E-11	1,547E-09	[5]	Group188	5,32	16,00
GO:0019220	regulation of phosphate metabolic process	GO_BiologicalProcess-EBI-UniProt-GOA-ACAP-ARAP_08.05.2020_00h00	1,73471E-08	4,04906E-07	8,69098E-11	1,547E-09	[5, 6]	Group188	4,71	91,00
GO:0030162	regulation of proteolysis	GO_BiologicalProcess-EBI-UniProt-GOA-ACAP-ARAP_08.05.2020_00h00	0,004999209	0,011901102	8,69098E-11	1,547E-09	[5, 6]	Group188	4,21	33,00
GO:0032269	negative regulation of cellular protein metabolic process	GO_BiologicalProcess-EBI-UniProt-GOA-ACAP-ARAP_08.05.2020_00h00	0,00020134	0,001035927	8,69098E-11	1,547E-09	[4, 5, 6, 7]	Group188	4,45	49,00
GO:0031399	regulation of protein modification process	GO_BiologicalProcess-EBI-UniProt-GOA-ACAP-ARAP_08.05.2020_00h00	1,07623E-07	1,68845E-06	8,69098E-11	1,547E-09	[5, 6, 7]	Group188	4,55	90,00
GO:0045936	negative regulation of phosphate metabolic process	GO_BiologicalProcess-EBI-UniProt-GOA-ACAP-ARAP_08.05.2020_00h00	0,000540563	0,002239477	8,69098E-11	1,547E-09	[5, 6, 7]	Group188	5,02	30,00
GO:0042325	regulation of phosphorylation	GO_BiologicalProcess-EBI-UniProt-GOA-ACAP-ARAP_08.05.2020_00h00	6,23797E-08	1,10551E-06	8,69098E-11	1,547E-09	[6, 7]	Group188	4,77	82,00
GO:0045861	negative regulation of proteolysis	GO_BiologicalProcess-EBI-UniProt-GOA-ACAP-ARAP_08.05.2020_00h00	0,006054374	0,013861331	8,69098E-11	1,547E-09	[5, 6, 7, 8]	Group188	4,96	19,00
GO:0043549	regulation of kinase activity	GO_BiologicalProcess-EBI-UniProt-GOA-ACAP-ARAP_08.05.2020_00h00	1,8027E-06	2,05379E-05	8,69098E-11	1,547E-09	[5, 7, 8]	Group188	5,25	51,00
GO:0001932	regulation of protein phosphorylation	GO_BiologicalProcess-EBI-UniProt-GOA-ACAP-ARAP_08.05.2020_00h00	1,20031E-07	1,85273E-06	8,69098E-11	1,547E-09	[6, 7, 8]	Group188	4,86	75,00
GO:0042326	negative regulation of phosphorylation	GO_BiologicalProcess-EBI-UniProt-GOA-ACAP-ARAP_08.05.2020_00h00	0,001627748	0,005192518	8,69098E-11	1,547E-09	[6, 7, 8]	Group188	5,07	24,00
GO:0001933	negative regulation of protein phosphorylation	GO_BiologicalProcess-EBI-UniProt-GOA-ACAP-ARAP_08.05.2020_00h00	0,004805825	0,011555714	8,69098E-11	1,547E-09	[6, 7, 8, 9]	Group188	4,88	21,00
GO:0004672	protein kinase activity	GO_BiologicalProcess-EBI-UniProt-GOA-ACAP-ARAP_08.05.2020_00h00	4,63941E-05	0,000324081	8,69098E-11	1,547E-09	[7, 8]	Group188	4,46	58,00
GO:0004693	cyclin-dependent protein serine/threonine kinase activity	GO_BiologicalProcess-EBI-UniProt-GOA-ACAP-ARAP_08.05.2020_00h00	0,000545112	0,002248586	8,69098E-11	1,547E-09	[5, 6, 9, 10]	Group188	8,59	11,00
GO:0045859	regulation of protein kinase activity	GO_BiologicalProcess-EBI-UniProt-GOA-ACAP-ARAP_08.05.2020_00h00	1,77981E-05	0,000148111	8,69098E-11	1,547E-09	[6, 7, 8, 9]	Group188	5,12	44,00
GO:0052548	regulation of endopeptidase activity	GO_BiologicalProcess-EBI-UniProt-GOA-ACAP-ARAP_08.05.2020_00h00	0,000937128	0,003333945	8,69098E-11	1,547E-09	[6, 7, 8, 9]	Group188	5,29	24,00
GO:2000134	negative regulation of G1/S transition of mitotic cell cycle	GO_BiologicalProcess-EBI-UniProt-GOA-ACAP-ARAP_08.05.2020_00h00	0,022311579	0,037394363	8,69098E-11	1,547E-09	[6, 7, 8, 9]	Group188	6,54	7,00
GO:0000079	regulation of cyclin-dependent protein serine/threonine kinase activity	GO_BiologicalProcess-EBI-UniProt-GOA-ACAP-ARAP_08.05.2020_00h00	0,001520331	0,004948833	8,69098E-11	1,547E-09	[5, 6, 7, 8, 9, 10, 11]	Group188	8,74	9,00
GO:0045736	negative regulation of cyclin-dependent protein serine/threonine kinase activity	GO_BiologicalProcess-EBI-UniProt-GOA-ACAP-ARAP_08.05.2020_00h00	0,007393209	0,016265061	8,69098E-11	1,547E-09	[4, 5, 6, 7, 8, 9, 10, 11, 12]	Group188	13,33	4,00
GO:0006469	negative regulation of protein kinase activity	GO_BiologicalProcess-EBI-UniProt-GOA-ACAP-ARAP_08.05.2020_00h00	0,012703408	0,02460964	8,69098E-11	1,547E-09	[7, 8, 9, 10]	Group188	5,31	13,00
GO:0010951	negative regulation of endopeptidase activity	GO_BiologicalProcess-EBI-UniProt-GOA-ACAP-ARAP_08.05.2020_00h00	0,013479644	0,025748543	8,69098E-11	1,547E-09	[7, 8, 9, 10]	Group188	5,11	14,00
GO:0071900	regulation of protein serine/threonine kinase activity	GO_BiologicalProcess-EBI-UniProt-GOA-ACAP-ARAP_08.05.2020_00h00	6,17963E-05	0,00041356	8,69098E-11	1,547E-09	[7, 8, 9, 10]	Group188	5,61	31,00

GO:0012501	programmed cell death	GO_BiologicalProcess-EBI-UniProt-GOA-ACAP-ARAP_08.05.2020_00h00	9,88592E-07	1,19757E-05	1,12394E-10	1,6672E-09	[3]	Group180	4,27	94,00
GO:0048584	positive regulation of response to stimulus	GO_BiologicalProcess-EBI-UniProt-GOA-ACAP-ARAP_08.05.2020_00h00	2,07976E-07	3,15925E-06	1,12394E-10	1,6672E-09	[2, 3, 4]	Group180	4,21	109,00
GO:0051094	positive regulation of developmental process	GO_BiologicalProcess-EBI-UniProt-GOA-ACAP-ARAP_08.05.2020_00h00	8,57256E-07	1,07947E-05	1,12394E-10	1,6672E-09	[2, 3, 4]	Group180	4,74	70,00
GO:0010941	regulation of cell death	GO_BiologicalProcess-EBI-UniProt-GOA-ACAP-ARAP_08.05.2020_00h00	3,69996E-07	5,20716E-06	1,12394E-10	1,6672E-09	[3, 4]	Group180	4,57	82,00
GO:0042127	regulation of cell population proliferation	GO_BiologicalProcess-EBI-UniProt-GOA-ACAP-ARAP_08.05.2020_00h00	9,02399E-10	5,75731E-08	1,12394E-10	1,6672E-09	[3, 4]	Group180	5,05	89,00
GO:0008284	positive regulation of cell population proliferation	GO_BiologicalProcess-EBI-UniProt-GOA-ACAP-ARAP_08.05.2020_00h00	8,18119E-08	1,37358E-06	1,12394E-10	1,6672E-09	[3, 4, 5]	Group180	5,61	55,00
GO:0010942	positive regulation of cell death	GO_BiologicalProcess-EBI-UniProt-GOA-ACAP-ARAP_08.05.2020_00h00	5,54439E-06	5,52706E-05	1,12394E-10	1,6672E-09	[3, 4, 5]	Group180	5,53	41,00
GO:0045597	positive regulation of cell differentiation	GO_BiologicalProcess-EBI-UniProt-GOA-ACAP-ARAP_08.05.2020_00h00	0,00015702	0,000878762	1,12394E-10	1,6672E-09	[3, 4, 5]	Group180	4,52	48,00
GO:0060548	negative regulation of cell death	GO_BiologicalProcess-EBI-UniProt-GOA-ACAP-ARAP_08.05.2020_00h00	6,67908E-06	6,3286E-05	1,12394E-10	1,6672E-09	[3, 4, 5]	Group180	4,93	53,00
GO:0097190	apoptotic signaling pathway	GO_BiologicalProcess-EBI-UniProt-GOA-ACAP-ARAP_08.05.2020_00h00	1,38238E-06	1,63326E-05	1,12394E-10	1,6672E-09	[3, 4, 5]	Group180	6,00	39,00
GO:0097191	extrinsic apoptotic signaling pathway	GO_BiologicalProcess-EBI-UniProt-GOA-ACAP-ARAP_08.05.2020_00h00	0,000831256	0,00305966	1,12394E-10	1,6672E-09	[4, 5, 6]	Group180	6,48	16,00
GO:0097193	intrinsic apoptotic signaling pathway	GO_BiologicalProcess-EBI-UniProt-GOA-ACAP-ARAP_08.05.2020_00h00	0,000166499	0,000895165	1,12394E-10	1,6672E-09	[4, 5, 6]	Group180	6,56	20,00
GO:0042981	regulation of apoptotic process	GO_BiologicalProcess-EBI-UniProt-GOA-ACAP-ARAP_08.05.2020_00h00	1,11326E-06	1,33174E-05	1,12394E-10	1,6672E-09	[5, 6]	Group180	4,59	75,00
GO:2001233	regulation of apoptotic signaling pathway	GO_BiologicalProcess-EBI-UniProt-GOA-ACAP-ARAP_08.05.2020_00h00	0,003071183	0,008186971	1,12394E-10	1,6672E-09	[4, 5, 6, 7]	Group180	4,99	22,00
GO:0043065	positive regulation of apoptotic process	GO_BiologicalProcess-EBI-UniProt-GOA-ACAP-ARAP_08.05.2020_00h00	5,25908E-05	0,000362082	1,12394E-10	1,6672E-09	[5, 6, 7]	Group180	5,29	36,00
GO:0043066	negative regulation of apoptotic process	GO_BiologicalProcess-EBI-UniProt-GOA-ACAP-ARAP_08.05.2020_00h00	2,58132E-05	0,000200839	1,12394E-10	1,6672E-09	[5, 6, 7]	Group180	4,91	47,00
GO:2001234	negative regulation of apoptotic signaling pathway	GO_BiologicalProcess-EBI-UniProt-GOA-ACAP-ARAP_08.05.2020_00h00	0,001539791	0,004978311	1,12394E-10	1,6672E-09	[4, 5, 6, 7, 8]	Group180	6,11	16,00
GO:2001236	regulation of extrinsic apoptotic signaling pathway	GO_BiologicalProcess-EBI-UniProt-GOA-ACAP-ARAP_08.05.2020_00h00	0,01962244	0,034081079	1,12394E-10	1,6672E-09	[5, 6, 7, 8]	Group180	5,59	10,00
GO:2001242	regulation of intrinsic apoptotic signaling pathway	GO_BiologicalProcess-EBI-UniProt-GOA-ACAP-ARAP_08.05.2020_00h00	0,01703891	0,03036543	1,12394E-10	1,6672E-09	[5, 6, 7, 8]	Group180	5,71	10,00
GO:2001237	negative regulation of extrinsic apoptotic signaling pathway	GO_BiologicalProcess-EBI-UniProt-GOA-ACAP-ARAP_08.05.2020_00h00	0,012383579	0,024285011	1,12394E-10	1,6672E-09	[5, 6, 7, 8, 9]	Group180	6,78	8,00
GO:2001243	negative regulation of intrinsic apoptotic signaling pathway	GO_BiologicalProcess-EBI-UniProt-GOA-ACAP-ARAP_08.05.2020_00h00	0,00920515	0,019361163	1,12394E-10	1,6672E-09	[5, 6, 7, 8, 9]	Group180	7,14	8,00
GO:0050673	epithelial cell proliferation	GO_BiologicalProcess-EBI-UniProt-GOA-ACAP-ARAP_08.05.2020_00h00	3,83793E-06	3,94935E-05	1,07725E-10	1,7432E-09	[3]	Group163	6,71	29,00
GO:0048729	tissue morphogenesis	GO_BiologicalProcess-EBI-UniProt-GOA-ACAP-ARAP_08.05.2020_00h00	7,67653E-05	0,000486519	1,07725E-10	1,7432E-09	[3, 4]	Group163	5,14	37,00

GO:0035239	tube morphogenesis	GO_BiologicalProcess-EBI-UniProt-GOA-ACAP-ARAP_08.05.2020_00h00	6,64249E-09	2,11896E-07	1,07725E-10	1,7432E-09	[3, 4, 5]	Group163	6,04	55,00
GO:0001655	urogenital system development	GO_BiologicalProcess-EBI-UniProt-GOA-ACAP-ARAP_08.05.2020_00h00	0,00374708	0,009486654	1,07725E-10	1,7432E-09	[4, 5]	Group163	5,19	19,00
GO:0002009	morphogenesis of an epithelium	GO_BiologicalProcess-EBI-UniProt-GOA-ACAP-ARAP_08.05.2020_00h00	0,000210357	0,001059536	1,07725E-10	1,7432E-09	[4, 5]	Group163	5,24	31,00
GO:0050679	positive regulation of epithelial cell proliferation	GO_BiologicalProcess-EBI-UniProt-GOA-ACAP-ARAP_08.05.2020_00h00	0,00018895	0,000982745	1,07725E-10	1,7432E-09	[4, 5, 6]	Group163	7,41	16,00
GO:0060562	epithelial tube morphogenesis	GO_BiologicalProcess-EBI-UniProt-GOA-ACAP-ARAP_08.05.2020_00h00	0,025199339	0,040805023	1,07725E-10	1,7432E-09	[4, 5, 6]	Group163	4,51	16,00
GO:0072001	renal system development	GO_BiologicalProcess-EBI-UniProt-GOA-ACAP-ARAP_08.05.2020_00h00	0,010179704	0,02081619	1,07725E-10	1,7432E-09	[4, 5, 6]	Group163	5,02	16,00
GO:0001822	kidney development	GO_BiologicalProcess-EBI-UniProt-GOA-ACAP-ARAP_08.05.2020_00h00	0,032703687	0,049209792	1,07725E-10	1,7432E-09	[4, 5, 6, 7]	Group163	4,55	14,00
GO:0072073	kidney epithelium development	GO_BiologicalProcess-EBI-UniProt-GOA-ACAP-ARAP_08.05.2020_00h00	0,008775524	0,018580036	1,07725E-10	1,7432E-09	[5, 6, 7, 8]	Group163	6,33	10,00
GO:0035850	epithelial cell differentiation involved in kidney development	GO_BiologicalProcess-EBI-UniProt-GOA-ACAP-ARAP_08.05.2020_00h00	0,002283362	0,006702997	1,07725E-10	1,7432E-09	[5, 6, 7, 8, 9]	Group163	11,54	6,00
GO:0072015	glomerular visceral epithelial cell development	GO_BiologicalProcess-EBI-UniProt-GOA-ACAP-ARAP_08.05.2020_00h00	0,005222608	0,012340829	1,07725E-10	1,7432E-09	[6, 7, 8, 9, 10, 11, 12, 13]	Group163	21,43	3,00
GO:0007166	cell surface receptor signaling pathway	GO_BiologicalProcess-EBI-UniProt-GOA-ACAP-ARAP_08.05.2020_00h00	2,092E-08	4,76678E-07	1,63315E-10	1,938E-09	[3, 4, 5]	Group171	4,08	137,00
GO:0070848	response to growth factor	GO_BiologicalProcess-EBI-UniProt-GOA-ACAP-ARAP_08.05.2020_00h00	1,15067E-05	0,000101962	1,63315E-10	1,938E-09	[4]	Group171	5,37	41,00
GO:0090287	regulation of cellular response to growth factor stimulus	GO_BiologicalProcess-EBI-UniProt-GOA-ACAP-ARAP_08.05.2020_00h00	0,00110216	0,003767027	1,63315E-10	1,938E-09	[3, 4, 6]	Group171	5,94	18,00
GO:0009967	positive regulation of signal transduction	GO_BiologicalProcess-EBI-UniProt-GOA-ACAP-ARAP_08.05.2020_00h00	4,99385E-09	1,91165E-07	1,63315E-10	1,938E-09	[3, 4, 5, 6]	Group171	4,92	87,00
GO:0007167	enzyme linked receptor protein signaling pathway	GO_BiologicalProcess-EBI-UniProt-GOA-ACAP-ARAP_08.05.2020_00h00	3,81246E-06	3,96579E-05	1,63315E-10	1,938E-09	[4, 5, 6]	Group171	4,96	55,00
GO:0071363	cellular response to growth factor stimulus	GO_BiologicalProcess-EBI-UniProt-GOA-ACAP-ARAP_08.05.2020_00h00	2,22323E-05	0,000175837	1,63315E-10	1,938E-09	[5]	Group171	5,33	39,00
GO:0007169	transmembrane receptor protein tyrosine kinase signaling pathway	GO_BiologicalProcess-EBI-UniProt-GOA-ACAP-ARAP_08.05.2020_00h00	0,00012082	0,000718164	1,63315E-10	1,938E-09	[5, 6, 7]	Group171	4,97	38,00
GO:0045937	positive regulation of phosphate metabolic process	GO_BiologicalProcess-EBI-UniProt-GOA-ACAP-ARAP_08.05.2020_00h00	1,12601E-09	6,73495E-08	1,63315E-10	1,938E-09	[5, 6, 7]	Group171	5,60	70,00
GO:0031401	positive regulation of protein modification process	GO_BiologicalProcess-EBI-UniProt-GOA-ACAP-ARAP_08.05.2020_00h00	7,84556E-08	1,34075E-06	1,63315E-10	1,938E-09	[5, 6, 7, 8]	Group171	5,11	68,00
GO:0042325	regulation of phosphorylation	GO_BiologicalProcess-EBI-UniProt-GOA-ACAP-ARAP_08.05.2020_00h00	6,23797E-08	1,10551E-06	1,63315E-10	1,938E-09	[6, 7]	Group171	4,77	82,00
GO:0001932	regulation of protein phosphorylation	GO_BiologicalProcess-EBI-UniProt-GOA-ACAP-ARAP_08.05.2020_00h00	1,20031E-07	1,85273E-06	1,63315E-10	1,938E-09	[6, 7, 8]	Group171	4,86	75,00
GO:0042327	positive regulation of phosphorylation	GO_BiologicalProcess-EBI-UniProt-GOA-ACAP-ARAP_08.05.2020_00h00	3,4551E-08	6,74801E-07	1,63315E-10	1,938E-09	[6, 7, 8]	Group171	5,40	63,00
GO:0001934	positive regulation of protein phosphorylation	GO_BiologicalProcess-EBI-UniProt-GOA-ACAP-ARAP_08.05.2020_00h00	4,44036E-07	6,07061E-06	1,63315E-10	1,938E-09	[6, 7, 8, 9]	Group171	5,24	57,00

GO:0043408	regulation of MAPK cascade	GO_BiologicalProcess-EBI-UniProt-GOA-ACAP-ARAP_08.05.2020_00h00	3,12878E-06	3,36432E-05	1,63315E-10	1,938E-09	[5, 6, 7, 8, 9, 10]	Group171	5,54	43,00
GO:0043410	positive regulation of MAPK cascade	GO_BiologicalProcess-EBI-UniProt-GOA-ACAP-ARAP_08.05.2020_00h00	2,28998E-07	3,37156E-06	1,63315E-10	1,938E-09	[5, 6, 7, 8, 9, 10, 11]	Group171	6,54	38,00
GO:0051239	regulation of multicellular organismal process	GO_BiologicalProcess-EBI-UniProt-GOA-ACAP-ARAP_08.05.2020_00h00	8,97701E-09	2,60333E-07	1,42071E-10	1,9453E-09	[2, 3]	Group127	4,10	140,00
GO:0048585	negative regulation of response to stimulus	GO_BiologicalProcess-EBI-UniProt-GOA-ACAP-ARAP_08.05.2020_00h00	1,4974E-08	3,67438E-07	1,42071E-10	1,9453E-09	[2, 3, 4]	Group127	4,85	85,00
GO:0051093	negative regulation of developmental process	GO_BiologicalProcess-EBI-UniProt-GOA-ACAP-ARAP_08.05.2020_00h00	0,002781123	0,007692298	1,42071E-10	1,9453E-09	[2, 3, 4]	Group127	4,07	43,00
GO:0051241	negative regulation of multicellular organismal process	GO_BiologicalProcess-EBI-UniProt-GOA-ACAP-ARAP_08.05.2020_00h00	0,001074145	0,003697685	1,42071E-10	1,9453E-09	[2, 3, 4]	Group127	4,04	53,00
GO:2000026	regulation of multicellular organismal development	GO_BiologicalProcess-EBI-UniProt-GOA-ACAP-ARAP_08.05.2020_00h00	3,43602E-09	1,42968E-07	1,42071E-10	1,9453E-09	[3, 4]	Group127	4,64	103,00
GO:0002521	leukocyte differentiation	GO_BiologicalProcess-EBI-UniProt-GOA-ACAP-ARAP_08.05.2020_00h00	0,00341803	0,008912955	1,42071E-10	1,9453E-09	[4, 5, 6, 7, 8]	Group127	4,66	26,00
GO:0050793	regulation of developmental process	GO_BiologicalProcess-EBI-UniProt-GOA-ACAP-ARAP_08.05.2020_00h00	1,13592E-09	6,39455E-08	1,53186E-10	1,9476E-09	[2, 3]	Group126	4,42	124,00
GO:0051093	negative regulation of developmental process	GO_BiologicalProcess-EBI-UniProt-GOA-ACAP-ARAP_08.05.2020_00h00	0,002781123	0,007692298	1,53186E-10	1,9476E-09	[2, 3, 4]	Group126	4,07	43,00
GO:0051241	negative regulation of multicellular organismal process	GO_BiologicalProcess-EBI-UniProt-GOA-ACAP-ARAP_08.05.2020_00h00	0,001074145	0,003697685	1,53186E-10	1,9476E-09	[2, 3, 4]	Group126	4,04	53,00
GO:2000026	regulation of multicellular organismal development	GO_BiologicalProcess-EBI-UniProt-GOA-ACAP-ARAP_08.05.2020_00h00	3,43602E-09	1,42968E-07	1,53186E-10	1,9476E-09	[3, 4]	Group126	4,64	103,00
GO:0035239	tube morphogenesis	GO_BiologicalProcess-EBI-UniProt-GOA-ACAP-ARAP_08.05.2020_00h00	6,64249E-09	2,11896E-07	1,53186E-10	1,9476E-09	[3, 4, 5]	Group126	6,04	55,00
GO:0048514	blood vessel morphogenesis	GO_BiologicalProcess-EBI-UniProt-GOA-ACAP-ARAP_08.05.2020_00h00	9,25827E-09	2,60593E-07	1,53186E-10	1,9476E-09	[4, 5, 6, 7, 8, 9]	Group126	6,76	44,00
GO:0032879	regulation of localization	GO_BiologicalProcess-EBI-UniProt-GOA-ACAP-ARAP_08.05.2020_00h00	1,28192E-10	1,5335E-08	1,94136E-10	2,1598E-09	[2, 3]	Group179	4,45	133,00
GO:0140352	export from cell	GO_BiologicalProcess-EBI-UniProt-GOA-ACAP-ARAP_08.05.2020_00h00	9,53733E-05	0,000588853	1,94136E-10	2,1598E-09	[2, 4]	Group179	4,10	71,00
GO:0032880	regulation of protein localization	GO_BiologicalProcess-EBI-UniProt-GOA-ACAP-ARAP_08.05.2020_00h00	0,001417178	0,004660617	1,94136E-10	2,1598E-09	[3, 4]	Group179	4,12	47,00
GO:0051049	regulation of transport	GO_BiologicalProcess-EBI-UniProt-GOA-ACAP-ARAP_08.05.2020_00h00	6,30185E-05	0,00041881	1,94136E-10	2,1598E-09	[3, 4]	Group179	4,00	81,00
GO:0051050	positive regulation of transport	GO_BiologicalProcess-EBI-UniProt-GOA-ACAP-ARAP_08.05.2020_00h00	0,001033711	0,003597314	1,94136E-10	2,1598E-09	[2, 3, 4, 5]	Group179	4,20	46,00
GO:0060341	regulation of cellular localization	GO_BiologicalProcess-EBI-UniProt-GOA-ACAP-ARAP_08.05.2020_00h00	0,000142641	0,000812543	1,94136E-10	2,1598E-09	[3, 4]	Group179	4,51	49,00
GO:0034762	regulation of transmembrane transport	GO_BiologicalProcess-EBI-UniProt-GOA-ACAP-ARAP_08.05.2020_00h00	0,009670558	0,020031869	1,94136E-10	2,1598E-09	[3, 4, 5]	Group179	4,29	26,00
GO:1904951	positive regulation of establishment of protein localization	GO_BiologicalProcess-EBI-UniProt-GOA-ACAP-ARAP_08.05.2020_00h00	0,032269876	0,048864357	1,94136E-10	2,1598E-09	[2, 3, 4, 5, 6]	Group179	4,05	21,00
GO:0043269	regulation of ion transport	GO_BiologicalProcess-EBI-UniProt-GOA-ACAP-ARAP_08.05.2020_00h00	0,013630096	0,025880955	1,94136E-10	2,1598E-09	[4, 5]	Group179	4,02	30,00

GO:0043270	positive regulation of ion transport	GO_BiologicalProcess-EBI-UniProt-GOA-ACAP-ARAP_08.05.2020_00h00	0,029049521	0,045351375	1,94136E-10	2,1598E-09	[3, 4, 5, 6]	Group179	4,62	14,00
GO:0070201	regulation of establishment of protein localization	GO_BiologicalProcess-EBI-UniProt-GOA-ACAP-ARAP_08.05.2020_00h00	0,004486383	0,01086954	1,94136E-10	2,1598E-09	[4, 5]	Group179	4,18	35,00
GO:1903827	regulation of cellular protein localization	GO_BiologicalProcess-EBI-UniProt-GOA-ACAP-ARAP_08.05.2020_00h00	0,014565298	0,026805751	1,94136E-10	2,1598E-09	[4, 5]	Group179	4,19	25,00
GO:1903829	positive regulation of cellular protein localization	GO_BiologicalProcess-EBI-UniProt-GOA-ACAP-ARAP_08.05.2020_00h00	0,025775954	0,04145813	1,94136E-10	2,1598E-09	[3, 4, 5, 6]	Group179	4,49	16,00
GO:0072657	protein localization to membrane	GO_BiologicalProcess-EBI-UniProt-GOA-ACAP-ARAP_08.05.2020_00h00	0,00820952	0,017734786	1,94136E-10	2,1598E-09	[5]	Group179	4,22	29,00
GO:0090087	regulation of peptide transport	GO_BiologicalProcess-EBI-UniProt-GOA-ACAP-ARAP_08.05.2020_00h00	0,009193194	0,019378606	1,94136E-10	2,1598E-09	[4, 5, 6, 7]	Group179	4,01	34,00
GO:0009306	protein secretion	GO_BiologicalProcess-EBI-UniProt-GOA-ACAP-ARAP_08.05.2020_00h00	0,017033646	0,030412685	1,94136E-10	2,1598E-09	[4, 5, 6, 7, 8]	Group179	4,05	27,00
GO:0050663	cytokine secretion	GO_BiologicalProcess-EBI-UniProt-GOA-ACAP-ARAP_08.05.2020_00h00	0,028077139	0,044266593	1,94136E-10	2,1598E-09	[3, 4, 5, 6, 7, 8, 9]	Group179	4,76	13,00
GO:0072659	protein localization to plasma membrane	GO_BiologicalProcess-EBI-UniProt-GOA-ACAP-ARAP_08.05.2020_00h00	0,010597887	0,021351954	1,94136E-10	2,1598E-09	[6]	Group179	5,12	15,00
GO:1903747	regulation of establishment of protein localization to mitochondrion	GO_BiologicalProcess-EBI-UniProt-GOA-ACAP-ARAP_08.05.2020_00h00	0,016363574	0,029491413	1,94136E-10	2,1598E-09	[5, 6, 7, 8]	Group179	7,69	6,00
GO:0070838	divalent metal ion transport	GO_BiologicalProcess-EBI-UniProt-GOA-ACAP-ARAP_08.05.2020_00h00	0,001574539	0,005056488	1,94136E-10	2,1598E-09	[7]	Group179	4,93	26,00
GO:0060402	calcium ion transport into cytosol	GO_BiologicalProcess-EBI-UniProt-GOA-ACAP-ARAP_08.05.2020_00h00	0,000377397	0,001703626	1,94136E-10	2,1598E-09	[4, 10, 12]	Group179	7,57	14,00
GO:0030198	extracellular matrix organization	GO_BiologicalProcess-EBI-UniProt-GOA-ACAP-ARAP_08.05.2020_00h00	4,35711E-10	3,4748E-08	4,35711E-10	4,5622E-09	[5]	Group042	8,77	35,00
GO:0009653	anatomical structure morphogenesis	GO_BiologicalProcess-EBI-UniProt-GOA-ACAP-ARAP_08.05.2020_00h00	9,29458E-11	1,48249E-08	4,8189E-10	4,7654E-09	[2, 3]	Group184	4,51	130,00
GO:0009888	tissue development	GO_BiologicalProcess-EBI-UniProt-GOA-ACAP-ARAP_08.05.2020_00h00	5,58784E-08	1,00898E-06	4,8189E-10	4,7654E-09	[3]	Group184	4,48	98,00
GO:0048646	anatomical structure formation involved in morphogenesis	GO_BiologicalProcess-EBI-UniProt-GOA-ACAP-ARAP_08.05.2020_00h00	2,69379E-08	5,60425E-07	4,8189E-10	4,7654E-09	[2, 3, 4]	Group184	5,40	64,00
GO:0051093	negative regulation of developmental process	GO_BiologicalProcess-EBI-UniProt-GOA-ACAP-ARAP_08.05.2020_00h00	0,002781123	0,007692298	4,8189E-10	4,7654E-09	[2, 3, 4]	Group184	4,07	43,00
GO:0009790	embryo development	GO_BiologicalProcess-EBI-UniProt-GOA-ACAP-ARAP_08.05.2020_00h00	0,000211308	0,001058752	4,8189E-10	4,7654E-09	[3, 4]	Group184	4,41	50,00
GO:0022603	regulation of anatomical structure morphogenesis	GO_BiologicalProcess-EBI-UniProt-GOA-ACAP-ARAP_08.05.2020_00h00	5,07994E-07	6,84718E-06	4,8189E-10	4,7654E-09	[3, 4]	Group184	5,11	60,00
GO:0035295	tube development	GO_BiologicalProcess-EBI-UniProt-GOA-ACAP-ARAP_08.05.2020_00h00	2,65621E-08	5,64888E-07	4,8189E-10	4,7654E-09	[3, 4]	Group184	5,48	62,00
GO:0048729	tissue morphogenesis	GO_BiologicalProcess-EBI-UniProt-GOA-ACAP-ARAP_08.05.2020_00h00	7,67653E-05	0,000486519	4,8189E-10	4,7654E-09	[3, 4]	Group184	5,14	37,00
GO:2000026	regulation of multicellular organismal development	GO_BiologicalProcess-EBI-UniProt-GOA-ACAP-ARAP_08.05.2020_00h00	3,43602E-09	1,42968E-07	4,8189E-10	4,7654E-09	[3, 4]	Group184	4,64	103,00
GO:0035239	tube morphogenesis	GO_BiologicalProcess-EBI-UniProt-GOA-ACAP-ARAP_08.05.2020_00h00	6,64249E-09	2,11896E-07	4,8189E-10	4,7654E-09	[3, 4, 5]	Group184	6,04	55,00

GO:0048598	embryonic morphogenesis	GO_BiologicalProcess-EBI-UniProt-GOA-ACAP-ARAP_08.05.2020_00h00	0,000934627	0,003362548	4,8189E-10	4,7654E-09	[3, 4, 5]	Group184	4,79	31,00
GO:0060429	epithelium development	GO_BiologicalProcess-EBI-UniProt-GOA-ACAP-ARAP_08.05.2020_00h00	5,08926E-06	5,18131E-05	4,8189E-10	4,7654E-09	[4]	Group184	4,64	64,00
GO:0002009	morphogenesis of an epithelium	GO_BiologicalProcess-EBI-UniProt-GOA-ACAP-ARAP_08.05.2020_00h00	0,000210357	0,001059536	4,8189E-10	4,7654E-09	[4, 5]	Group184	5,24	31,00
GO:0009887	animal organ morphogenesis	GO_BiologicalProcess-EBI-UniProt-GOA-ACAP-ARAP_08.05.2020_00h00	0,000679847	0,002644772	4,8189E-10	4,7654E-09	[3, 4, 5, 6]	Group184	4,21	49,00
GO:0030855	epithelial cell differentiation	GO_BiologicalProcess-EBI-UniProt-GOA-ACAP-ARAP_08.05.2020_00h00	0,00043345	0,001885508	4,8189E-10	4,7654E-09	[4, 5]	Group184	4,66	38,00
GO:0072359	circulatory system development	GO_BiologicalProcess-EBI-UniProt-GOA-ACAP-ARAP_08.05.2020_00h00	3,81703E-07	5,29405E-06	4,8189E-10	4,7654E-09	[4, 5]	Group184	5,15	60,00
GO:0001704	formation of primary germ layer	GO_BiologicalProcess-EBI-UniProt-GOA-ACAP-ARAP_08.05.2020_00h00	0,008327947	0,017869608	4,8189E-10	4,7654E-09	[3, 4, 5, 6, 7]	Group184	6,77	9,00
GO:0002064	epithelial cell development	GO_BiologicalProcess-EBI-UniProt-GOA-ACAP-ARAP_08.05.2020_00h00	3,30027E-06	3,50928E-05	4,8189E-10	4,7654E-09	[4, 5, 6]	Group184	8,62	20,00
GO:0007369	gastrulation	GO_BiologicalProcess-EBI-UniProt-GOA-ACAP-ARAP_08.05.2020_00h00	0,00706902	0,015696175	4,8189E-10	4,7654E-09	[4, 5, 6]	Group184	5,94	12,00
GO:0060562	epithelial tube morphogenesis	GO_BiologicalProcess-EBI-UniProt-GOA-ACAP-ARAP_08.05.2020_00h00	0,025199339	0,040805023	4,8189E-10	4,7654E-09	[4, 5, 6]	Group184	4,51	16,00
GO:0001944	vasculature development	GO_BiologicalProcess-EBI-UniProt-GOA-ACAP-ARAP_08.05.2020_00h00	3,67366E-09	1,46487E-07	4,8189E-10	4,7654E-09	[4, 5, 6, 7]	Group184	6,47	50,00
GO:0001568	blood vessel development	GO_BiologicalProcess-EBI-UniProt-GOA-ACAP-ARAP_08.05.2020_00h00	7,05492E-09	2,10986E-07	4,8189E-10	4,7654E-09	[3, 5, 6, 7, 8]	Group184	6,49	48,00
GO:1901342	regulation of vasculature development	GO_BiologicalProcess-EBI-UniProt-GOA-ACAP-ARAP_08.05.2020_00h00	1,26962E-05	0,00011147	4,8189E-10	4,7654E-09	[4, 5, 6, 7, 8]	Group184	6,85	25,00
GO:0001525	angiogenesis	GO_BiologicalProcess-EBI-UniProt-GOA-ACAP-ARAP_08.05.2020_00h00	5,95789E-09	2,11174E-07	4,8189E-10	4,7654E-09	[3, 4, 5, 6, 7, 8, 9, 10]	Group184	7,27	40,00
GO:0048514	blood vessel morphogenesis	GO_BiologicalProcess-EBI-UniProt-GOA-ACAP-ARAP_08.05.2020_00h00	9,25827E-09	2,60593E-07	4,8189E-10	4,7654E-09	[4, 5, 6, 7, 8, 9]	Group184	6,76	44,00
GO:0045765	regulation of angiogenesis	GO_BiologicalProcess-EBI-UniProt-GOA-ACAP-ARAP_08.05.2020_00h00	5,97633E-05	0,000405628	4,8189E-10	4,7654E-09	[4, 5, 6, 7, 8, 9, 10, 11]	Group184	6,69	22,00
GO:0044092	negative regulation of molecular function	GO_BiologicalProcess-EBI-UniProt-GOA-ACAP-ARAP_08.05.2020_00h00	0,001134739	0,003864575	6,09514E-10	5,7102E-09	[3]	Group177	4,07	51,00
GO:0044093	positive regulation of molecular function	GO_BiologicalProcess-EBI-UniProt-GOA-ACAP-ARAP_08.05.2020_00h00	6,68997E-11	1,28046E-08	6,09514E-10	5,7102E-09	[3]	Group177	5,07	99,00
GO:0050790	regulation of catalytic activity	GO_BiologicalProcess-EBI-UniProt-GOA-ACAP-ARAP_08.05.2020_00h00	1,121E-08	3,06514E-07	6,09514E-10	5,7102E-09	[3]	Group177	4,41	112,00
GO:0030234	enzyme regulator activity	GO_BiologicalProcess-EBI-UniProt-GOA-ACAP-ARAP_08.05.2020_00h00	0,000976118	0,003447031	6,09514E-10	5,7102E-09	[4]	Group177	4,17	48,00
GO:0043085	positive regulation of catalytic activity	GO_BiologicalProcess-EBI-UniProt-GOA-ACAP-ARAP_08.05.2020_00h00	1,27347E-09	6,09358E-08	6,09514E-10	5,7102E-09	[4]	Group177	5,20	82,00
GO:0043086	negative regulation of catalytic activity	GO_BiologicalProcess-EBI-UniProt-GOA-ACAP-ARAP_08.05.2020_00h00	0,003219853	0,008512152	6,09514E-10	5,7102E-09	[4]	Group177	4,20	37,00
GO:0051020	GTPase binding	GO_MolecularFunction-EBI-UniProt-GOA-ACAP-ARAP_08.05.2020_00h00	0,012022416	0,023673771	6,09514E-10	5,7102E-09	[4]	Group177	4,26	25,00
GO:0051336	regulation of hydrolase activity	GO_BiologicalProcess-EBI-UniProt-GOA-ACAP-ARAP_08.05.2020_00h00	8,71715E-06	7,94506E-05	6,09514E-10	5,7102E-09	[4]	Group177	4,59	63,00

GO:0007264	small GTPase mediated signal transduction	GO_BiologicalProcess-EBI-UniProt-GOA-ACAP-ARAP_08.05.2020_00h00	0,002490827	0,007158323	6,09514E-10	5,7102E-09	[4, 5, 6]	Group177	4,65	28,00
GO:0008047	enzyme activator activity	GO_BiologicalProcess-EBI-UniProt-GOA-ACAP-ARAP_08.05.2020_00h00	0,000987177	0,003460544	6,09514E-10	5,7102E-09	[5]	Group177	4,96	28,00
GO:0043087	regulation of GTPase activity	GO_BiologicalProcess-EBI-UniProt-GOA-ACAP-ARAP_08.05.2020_00h00	0,000172834	0,000924034	6,09514E-10	5,7102E-09	[5]	Group177	5,53	28,00
GO:0051345	positive regulation of hydrolase activity	GO_BiologicalProcess-EBI-UniProt-GOA-ACAP-ARAP_08.05.2020_00h00	9,69574E-06	8,7536E-05	6,09514E-10	5,7102E-09	[5]	Group177	5,30	43,00
GO:0060589	nucleoside-triphosphatase regulator activity	GO_BiologicalProcess-EBI-UniProt-GOA-ACAP-ARAP_08.05.2020_00h00	0,003531843	0,009110441	6,09514E-10	5,7102E-09	[5]	Group177	5,22	19,00
GO:0030162	regulation of proteolysis	GO_BiologicalProcess-EBI-UniProt-GOA-ACAP-ARAP_08.05.2020_00h00	0,004999209	0,011901102	6,09514E-10	5,7102E-09	[5, 6]	Group177	4,21	33,00
GO:0043547	positive regulation of GTPase activity	GO_BiologicalProcess-EBI-UniProt-GOA-ACAP-ARAP_08.05.2020_00h00	0,000437988	0,001896626	6,09514E-10	5,7102E-09	[6]	Group177	5,58	24,00
GO:0005096	GTPase activator activity	GO_BiologicalProcess-EBI-UniProt-GOA-ACAP-ARAP_08.05.2020_00h00	0,004219768	0,010354661	6,09514E-10	5,7102E-09	[6, 7]	Group177	5,52	16,00
GO:0045862	positive regulation of proteolysis	GO_BiologicalProcess-EBI-UniProt-GOA-ACAP-ARAP_08.05.2020_00h00	0,023925476	0,039273895	6,09514E-10	5,7102E-09	[5, 6, 7, 8]	Group177	4,45	17,00
GO:0010952	positive regulation of peptidase activity	GO_BiologicalProcess-EBI-UniProt-GOA-ACAP-ARAP_08.05.2020_00h00	0,000395714	0,001777926	6,09514E-10	5,7102E-09	[6, 7, 8, 9]	Group177	7,21	15,00
GO:0052548	regulation of endopeptidase activity	GO_BiologicalProcess-EBI-UniProt-GOA-ACAP-ARAP_08.05.2020_00h00	0,000937128	0,003333945	6,09514E-10	5,7102E-09	[6, 7, 8, 9]	Group177	5,29	24,00
GO:0043280	positive regulation of cysteine-type endopeptidase activity involved in apoptotic process	GO_BiologicalProcess-EBI-UniProt-GOA-ACAP-ARAP_08.05.2020_00h00	0,000360827	0,001652209	6,09514E-10	5,7102E-09	[6, 7, 8, 9, 10, 11, 12]	Group177	8,45	12,00
GO:0023051	regulation of signaling	GO_BiologicalProcess-EBI-UniProt-GOA-ACAP-ARAP_08.05.2020_00h00	4,33256E-10	3,76933E-08	6,94518E-10	6,1812E-09	[2, 3]	Group154	4,12	158,00
GO:0010033	response to organic substance	GO_BiologicalProcess-EBI-UniProt-GOA-ACAP-ARAP_08.05.2020_00h00	4,09467E-08	7,68353E-07	6,94518E-10	6,1812E-09	[3]	Group154	4,00	141,00
GO:0010646	regulation of cell communication	GO_BiologicalProcess-EBI-UniProt-GOA-ACAP-ARAP_08.05.2020_00h00	3,57848E-10	3,4246E-08	6,94518E-10	6,1812E-09	[3, 4]	Group154	4,14	157,00
GO:0007166	cell surface receptor signaling pathway	GO_BiologicalProcess-EBI-UniProt-GOA-ACAP-ARAP_08.05.2020_00h00	2,092E-08	4,76678E-07	6,94518E-10	6,1812E-09	[3, 4, 5]	Group154	4,08	137,00
GO:0034097	response to cytokine	GO_BiologicalProcess-EBI-UniProt-GOA-ACAP-ARAP_08.05.2020_00h00	2,97291E-07	4,31072E-06	6,94518E-10	6,1812E-09	[4]	Group154	5,05	64,00
GO:0071310	cellular response to organic substance	GO_BiologicalProcess-EBI-UniProt-GOA-ACAP-ARAP_08.05.2020_00h00	3,0656E-08	6,24208E-07	6,94518E-10	6,1812E-09	[4]	Group154	4,21	122,00
GO:0019221	cytokine-mediated signaling pathway	GO_BiologicalProcess-EBI-UniProt-GOA-ACAP-ARAP_08.05.2020_00h00	0,000164126	0,000887395	6,94518E-10	6,1812E-09	[4, 5, 6]	Group154	4,81	40,00
GO:0070555	response to interleukin-1	GO_BiologicalProcess-EBI-UniProt-GOA-ACAP-ARAP_08.05.2020_00h00	1,33456E-05	0,000114034	6,94518E-10	6,1812E-09	[5]	Group154	8,45	18,00
GO:0071345	cellular response to cytokine stimulus	GO_BiologicalProcess-EBI-UniProt-GOA-ACAP-ARAP_08.05.2020_00h00	2,34572E-06	2,55097E-05	6,94518E-10	6,1812E-09	[5]	Group154	4,94	58,00
GO:0071363	cellular response to growth factor stimulus	GO_BiologicalProcess-EBI-UniProt-GOA-ACAP-ARAP_08.05.2020_00h00	2,22323E-05	0,000175837	6,94518E-10	6,1812E-09	[5]	Group154	5,33	39,00
GO:0048870	cell motility	GO_BiologicalProcess-EBI-UniProt-GOA-ACAP-ARAP_08.05.2020_00h00	1,18486E-09	6,29948E-08	7,52287E-10	6,3765E-09	[2, 3]	Group175	5,00	90,00

GO:0031589	cell-substrate adhesion	GO_BiologicalProcess-EBI-UniProt-GOA-ACAP-ARAP_08.05.2020_00h00	3,26496E-10	3,47175E-08	7,52287E-10	6,3765E-09	[3]	Group175	9,07	34,00
GO:0030155	regulation of cell adhesion	GO_BiologicalProcess-EBI-UniProt-GOA-ACAP-ARAP_08.05.2020_00h00	1,32928E-08	3,34768E-07	7,52287E-10	6,3765E-09	[3, 4]	Group175	6,36	48,00
GO:0007160	cell-matrix adhesion	GO_BiologicalProcess-EBI-UniProt-GOA-ACAP-ARAP_08.05.2020_00h00	2,11574E-08	4,70876E-07	7,52287E-10	6,3765E-09	[4]	Group175	10,00	24,00
GO:0034330	cell junction organization	GO_BiologicalProcess-EBI-UniProt-GOA-ACAP-ARAP_08.05.2020_00h00	6,16658E-05	0,000415593	7,52287E-10	6,3765E-09	[4]	Group175	5,08	39,00
GO:0045785	positive regulation of cell adhesion	GO_BiologicalProcess-EBI-UniProt-GOA-ACAP-ARAP_08.05.2020_00h00	2,56997E-05	0,000201595	7,52287E-10	6,3765E-09	[3, 4, 5]	Group175	6,18	28,00
GO:0010810	regulation of cell-substrate adhesion	GO_BiologicalProcess-EBI-UniProt-GOA-ACAP-ARAP_08.05.2020_00h00	3,73914E-05	0,000273157	7,52287E-10	6,3765E-09	[4, 5]	Group175	7,83	18,00
GO:0010811	positive regulation of cell-substrate adhesion	GO_BiologicalProcess-EBI-UniProt-GOA-ACAP-ARAP_08.05.2020_00h00	0,002776	0,007700383	7,52287E-10	6,3765E-09	[4, 5, 6]	Group175	7,46	10,00
GO:0010812	negative regulation of cell-substrate adhesion	GO_BiologicalProcess-EBI-UniProt-GOA-ACAP-ARAP_08.05.2020_00h00	0,032554209	0,04921703	7,52287E-10	6,3765E-09	[4, 5, 6]	Group175	7,35	5,00
GO:0034329	cell junction assembly	GO_BiologicalProcess-EBI-UniProt-GOA-ACAP-ARAP_08.05.2020_00h00	0,000284442	0,001361054	7,52287E-10	6,3765E-09	[5]	Group175	5,54	26,00
GO:1901888	regulation of cell junction assembly	GO_BiologicalProcess-EBI-UniProt-GOA-ACAP-ARAP_08.05.2020_00h00	0,029677309	0,045956612	7,52287E-10	6,3765E-09	[4, 5, 6]	Group175	5,02	11,00
GO:0001952	regulation of cell-matrix adhesion	GO_BiologicalProcess-EBI-UniProt-GOA-ACAP-ARAP_08.05.2020_00h00	4,35039E-05	0,000308394	7,52287E-10	6,3765E-09	[5, 6]	Group175	9,85	13,00
GO:1901890	positive regulation of cell junction assembly	GO_BiologicalProcess-EBI-UniProt-GOA-ACAP-ARAP_08.05.2020_00h00	0,023341313	0,038579684	7,52287E-10	6,3765E-09	[4, 5, 6, 7]	Group175	6,48	7,00
GO:0001954	positive regulation of cell-matrix adhesion	GO_BiologicalProcess-EBI-UniProt-GOA-ACAP-ARAP_08.05.2020_00h00	0,000936067	0,003342597	7,52287E-10	6,3765E-09	[5, 6, 7]	Group175	11,67	7,00
GO:0007044	cell-substrate junction assembly	GO_BiologicalProcess-EBI-UniProt-GOA-ACAP-ARAP_08.05.2020_00h00	6,03086E-06	5,82983E-05	7,52287E-10	6,3765E-09	[6]	Group175	11,82	13,00
GO:0048041	focal adhesion assembly	GO_BiologicalProcess-EBI-UniProt-GOA-ACAP-ARAP_08.05.2020_00h00	0,00302181	0,00814612	7,52287E-10	6,3765E-09	[5, 7]	Group175	8,60	8,00
GO:0031581	hemidesmosome assembly	GO_BiologicalProcess-EBI-UniProt-GOA-ACAP-ARAP_08.05.2020_00h00	1,29834E-05	0,000111938	7,52287E-10	6,3765E-09	[7]	Group175	38,46	5,00
GO:0050860	negative regulation of T cell receptor signaling pathway	GO_BiologicalProcess-EBI-UniProt-GOA-ACAP-ARAP_08.05.2020_00h00	0,00033793	0,001562316	7,52287E-10	6,3765E-09	[4, 5, 6, 7, 8, 9, 10, 11, 12]	Group175	20,83	5,00
GO:0004713	protein tyrosine kinase activity	GO_BiologicalProcess-EBI-UniProt-GOA-ACAP-ARAP_08.05.2020_00h00	0,017674305	0,031322796	7,52287E-10	6,3765E-09	[8, 9, 10]	Group175	5,08	13,00
GO:0033002	muscle cell proliferation	GO_BiologicalProcess-EBI-UniProt-GOA-ACAP-ARAP_08.05.2020_00h00	3,12928E-05	0,000233963	1,83274E-09	1,4829E-08	[3]	Group122	8,25	17,00
GO:0042127	regulation of cell population proliferation	GO_BiologicalProcess-EBI-UniProt-GOA-ACAP-ARAP_08.05.2020_00h00	9,02399E-10	5,75731E-08	1,83274E-09	1,4829E-08	[3, 4]	Group122	5,05	89,00
GO:0008284	positive regulation of cell population proliferation	GO_BiologicalProcess-EBI-UniProt-GOA-ACAP-ARAP_08.05.2020_00h00	8,18119E-08	1,37358E-06	1,83274E-09	1,4829E-08	[3, 4, 5]	Group122	5,61	55,00
GO:0060548	negative regulation of cell death	GO_BiologicalProcess-EBI-UniProt-GOA-ACAP-ARAP_08.05.2020_00h00	6,67908E-06	6,3286E-05	1,83274E-09	1,4829E-08	[3, 4, 5]	Group122	4,93	53,00
GO:0050678	regulation of epithelial cell proliferation	GO_BiologicalProcess-EBI-UniProt-GOA-ACAP-ARAP_08.05.2020_00h00	4,34628E-05	0,000310403	1,83274E-09	1,4829E-08	[4, 5]	Group122	6,50	24,00

GO:0050679	positive regulation of epithelial cell proliferation	GO_BiologicalProcess-EBI-UniProt-GOA-ACAP-ARAP_08.05.2020_00h00	0,00018895	0,000982745	1,83274E-09	1,4829E-08	[4, 5, 6]	Group122	7,41	16,00
GO:0070848	response to growth factor	GO_BiologicalProcess-EBI-UniProt-GOA-ACAP-ARAP_08.05.2020_00h00	1,15067E-05	0,000101962	2,70404E-09	2,0927E-08	[4]	Group160	5,37	41,00
GO:0071310	cellular response to organic substance	GO_BiologicalProcess-EBI-UniProt-GOA-ACAP-ARAP_08.05.2020_00h00	3,0656E-08	6,24208E-07	2,70404E-09	2,0927E-08	[4]	Group160	4,21	122,00
GO:0090287	regulation of cellular response to growth factor stimulus	GO_BiologicalProcess-EBI-UniProt-GOA-ACAP-ARAP_08.05.2020_00h00	0,00110216	0,003767027	2,70404E-09	2,0927E-08	[3, 4, 6]	Group160	5,94	18,00
GO:0007167	enzyme linked receptor protein signaling pathway	GO_BiologicalProcess-EBI-UniProt-GOA-ACAP-ARAP_08.05.2020_00h00	3,81246E-06	3,96579E-05	2,70404E-09	2,0927E-08	[4, 5, 6]	Group160	4,96	55,00
GO:0071363	cellular response to growth factor stimulus	GO_BiologicalProcess-EBI-UniProt-GOA-ACAP-ARAP_08.05.2020_00h00	2,22323E-05	0,000175837	2,70404E-09	2,0927E-08	[5]	Group160	5,33	39,00
GO:0071560	cellular response to transforming growth factor beta stimulus	GO_BiologicalProcess-EBI-UniProt-GOA-ACAP-ARAP_08.05.2020_00h00	0,006329319	0,014387551	2,70404E-09	2,0927E-08	[4, 6]	Group160	5,60	14,00
GO:0090288	negative regulation of cellular response to growth factor stimulus	GO_BiologicalProcess-EBI-UniProt-GOA-ACAP-ARAP_08.05.2020_00h00	0,012154936	0,023885572	2,70404E-09	2,0927E-08	[3, 4, 5, 6, 7]	Group160	6,02	10,00
GO:0007169	transmembrane receptor protein tyrosine kinase signaling pathway	GO_BiologicalProcess-EBI-UniProt-GOA-ACAP-ARAP_08.05.2020_00h00	0,00012082	0,000718164	2,70404E-09	2,0927E-08	[5, 6, 7]	Group160	4,97	38,00
GO:0090092	regulation of transmembrane receptor protein serine/threonine kinase signaling pathway	GO_BiologicalProcess-EBI-UniProt-GOA-ACAP-ARAP_08.05.2020_00h00	0,018717377	0,032806829	2,70404E-09	2,0927E-08	[4, 5, 6, 7, 8]	Group160	5,04	13,00
GO:0030510	regulation of BMP signaling pathway	GO_BiologicalProcess-EBI-UniProt-GOA-ACAP-ARAP_08.05.2020_00h00	0,013690378	0,025892671	2,70404E-09	2,0927E-08	[4, 5, 6, 7, 8, 9]	Group160	7,22	7,00
GO:0030513	positive regulation of BMP signaling pathway	GO_BiologicalProcess-EBI-UniProt-GOA-ACAP-ARAP_08.05.2020_00h00	0,016912684	0,030253155	2,70404E-09	2,0927E-08	[5, 6, 7, 8, 9, 10]	Group160	10,53	4,00
GO:0001935	endothelial cell proliferation	GO_BiologicalProcess-EBI-UniProt-GOA-ACAP-ARAP_08.05.2020_00h00	0,003633375	0,00934715	3,62156E-09	2,686E-08	[4]	Group161	6,79	11,00
GO:1901342	regulation of vasculature development	GO_BiologicalProcess-EBI-UniProt-GOA-ACAP-ARAP_08.05.2020_00h00	1,26962E-05	0,00011147	3,62156E-09	2,686E-08	[4, 5, 6, 7, 8]	Group161	6,85	25,00
GO:1901343	negative regulation of vasculature development	GO_BiologicalProcess-EBI-UniProt-GOA-ACAP-ARAP_08.05.2020_00h00	0,028789286	0,045166142	3,62156E-09	2,686E-08	[3, 4, 5, 6, 7, 8, 9]	Group161	5,80	8,00
GO:0001525	angiogenesis	GO_BiologicalProcess-EBI-UniProt-GOA-ACAP-ARAP_08.05.2020_00h00	5,95789E-09	2,11174E-07	3,62156E-09	2,686E-08	[3, 4, 5, 6, 7, 8, 9, 10]	Group161	7,27	40,00
GO:0043534	blood vessel endothelial cell migration	GO_BiologicalProcess-EBI-UniProt-GOA-ACAP-ARAP_08.05.2020_00h00	0,00532592	0,012492415	3,62156E-09	2,686E-08	[6, 7, 8]	Group161	7,26	9,00
GO:0043535	regulation of blood vessel endothelial cell migration	GO_BiologicalProcess-EBI-UniProt-GOA-ACAP-ARAP_08.05.2020_00h00	0,004171744	0,010316174	3,62156E-09	2,686E-08	[5, 6, 7, 8, 9]	Group161	8,16	8,00
GO:0002040	sprouting angiogenesis	GO_BiologicalProcess-EBI-UniProt-GOA-ACAP-ARAP_08.05.2020_00h00	0,00041584	0,001833911	3,62156E-09	2,686E-08	[4, 5, 6, 7, 8, 9, 10, 11]	Group161	8,87	11,00
GO:0045765	regulation of angiogenesis	GO_BiologicalProcess-EBI-UniProt-GOA-ACAP-ARAP_08.05.2020_00h00	5,97633E-05	0,000405628	3,62156E-09	2,686E-08	[4, 5, 6, 7, 8, 9, 10, 11]	Group161	6,69	22,00
GO:0002043	blood vessel endothelial cell proliferation involved in sprouting angiogenesis	GO_BiologicalProcess-EBI-UniProt-GOA-ACAP-ARAP_08.05.2020_00h00	0,00033793	0,001562316	3,62156E-09	2,686E-08	[5, 6, 7, 8, 9, 10, 11, 12]	Group161	20,83	5,00
GO:1903670	regulation of sprouting angiogenesis	GO_BiologicalProcess-EBI-UniProt-GOA-ACAP-ARAP_08.05.2020_00h00	0,003441989	0,008951043	3,62156E-09	2,686E-08	[5, 6, 7, 8, 9, 10, 11, 12]	Group161	9,33	7,00

GO:1903671	negative regulation of sprouting angiogenesis	GO_BiologicalProcess-EBI-UniProt-GOA-ACAP-ARAP_08.05.2020_00h00	0,032678593	0,04924947	3,62156E-09	2,686E-08	[5, 6, 7, 8, 9, 10, 11, 12, 13]	Group161	11,11	3,00
GO:0050839	cell adhesion molecule binding	GO_MolecularFunction-EBI-UniProt-GOA-ACAP-ARAP_08.05.2020_00h00	5,04854E-10	3,7165E-08	4,39417E-09	3,1286E-08	[3]	Group074	7,66	42,00
GO:0005178	integrin binding	GO_MolecularFunction-EBI-UniProt-GOA-ACAP-ARAP_08.05.2020_00h00	8,79082E-07	1,09257E-05	4,39417E-09	3,1286E-08	[3, 4]	Group074	10,76	17,00
GO:0045296	cadherin binding	GO_MolecularFunction-EBI-UniProt-GOA-ACAP-ARAP_08.05.2020_00h00	0,000159296	0,000886316	4,39417E-09	3,1286E-08	[4]	Group074	6,25	22,00
GO:0007229	integrin-mediated signaling pathway	GO_BiologicalProcess-EBI-UniProt-GOA-ACAP-ARAP_08.05.2020_00h00	0,001144356	0,003883505	4,39417E-09	3,1286E-08	[4, 5, 6]	Group074	8,40	10,00
GO:0040012	regulation of locomotion	GO_BiologicalProcess-EBI-UniProt-GOA-ACAP-ARAP_08.05.2020_00h00	1,89996E-12	1,81826E-09	9,46731E-09	6,4815E-08	[2, 3]	Group152	6,58	68,00
GO:0040017	positive regulation of locomotion	GO_BiologicalProcess-EBI-UniProt-GOA-ACAP-ARAP_08.05.2020_00h00	2,08823E-09	9,51635E-08	9,46731E-09	6,4815E-08	[2, 3, 4]	Group152	7,20	43,00
GO:0006935	chemotaxis	GO_BiologicalProcess-EBI-UniProt-GOA-ACAP-ARAP_08.05.2020_00h00	0,001700784	0,00538957	9,46731E-09	6,4815E-08	[3, 4]	Group152	4,56	32,00
GO:0051270	regulation of cellular component movement	GO_BiologicalProcess-EBI-UniProt-GOA-ACAP-ARAP_08.05.2020_00h00	4,42024E-11	2,11509E-08	9,46731E-09	6,4815E-08	[3, 4]	Group152	6,18	67,00
GO:0000904	cell morphogenesis involved in differentiation	GO_BiologicalProcess-EBI-UniProt-GOA-ACAP-ARAP_08.05.2020_00h00	0,000202059	0,001034068	9,46731E-09	6,4815E-08	[4, 5]	Group152	4,80	39,00
GO:0120039	plasma membrane bounded cell projection morphogenesis	GO_BiologicalProcess-EBI-UniProt-GOA-ACAP-ARAP_08.05.2020_00h00	0,009786652	0,020228567	9,46731E-09	6,4815E-08	[5, 6, 7]	Group152	4,12	30,00
GO:0048667	cell morphogenesis involved in neuron differentiation	GO_BiologicalProcess-EBI-UniProt-GOA-ACAP-ARAP_08.05.2020_00h00	0,006632533	0,014899846	9,46731E-09	6,4815E-08	[5, 6, 8, 9, 10]	Group152	4,33	28,00
GO:0007411	axon guidance	GO_BiologicalProcess-EBI-UniProt-GOA-ACAP-ARAP_08.05.2020_00h00	0,014432535	0,026663968	9,46731E-09	6,4815E-08	[4, 5, 6, 7, 8, 9, 10, 11, 12, 13]	Group152	4,93	15,00
GO:0061564	axon development	GO_BiologicalProcess-EBI-UniProt-GOA-ACAP-ARAP_08.05.2020_00h00	0,00784214	0,01709551	9,46731E-09	6,4815E-08	[6, 7, 9, 10, 11]	Group152	4,42	25,00
GO:0048584	positive regulation of response to stimulus	GO_BiologicalProcess-EBI-UniProt-GOA-ACAP-ARAP_08.05.2020_00h00	2,07976E-07	3,15925E-06	1,09679E-08	7,2307E-08	[2, 3, 4]	Group100	4,21	109,00
GO:0042127	regulation of cell population proliferation	GO_BiologicalProcess-EBI-UniProt-GOA-ACAP-ARAP_08.05.2020_00h00	9,02399E-10	5,75731E-08	1,09679E-08	7,2307E-08	[3, 4]	Group100	5,05	89,00
GO:0008284	positive regulation of cell population proliferation	GO_BiologicalProcess-EBI-UniProt-GOA-ACAP-ARAP_08.05.2020_00h00	8,18119E-08	1,37358E-06	1,09679E-08	7,2307E-08	[3, 4, 5]	Group100	5,61	55,00
GO:0008285	negative regulation of cell population proliferation	GO_BiologicalProcess-EBI-UniProt-GOA-ACAP-ARAP_08.05.2020_00h00	0,002655257	0,007430061	1,09679E-08	7,2307E-08	[3, 4, 5]	Group100	4,39	33,00
GO:0042981	regulation of apoptotic process	GO_BiologicalProcess-EBI-UniProt-GOA-ACAP-ARAP_08.05.2020_00h00	1,11326E-06	1,33174E-05	1,09679E-08	7,2307E-08	[5, 6]	Group100	4,59	75,00
GO:0006793	phosphorus metabolic process	GO_BiologicalProcess-EBI-UniProt-GOA-ACAP-ARAP_08.05.2020_00h00	6,13427E-09	2,09661E-07	1,17301E-08	7,457E-08	[3]	Group173	4,09	144,00
GO:0019637	organophosphate metabolic process	GO_BiologicalProcess-EBI-UniProt-GOA-ACAP-ARAP_08.05.2020_00h00	0,000611052	0,002436569	1,17301E-08	7,457E-08	[3, 4]	Group173	4,26	48,00
GO:0006796	phosphate-containing compound metabolic process	GO_BiologicalProcess-EBI-UniProt-GOA-ACAP-ARAP_08.05.2020_00h00	2,32844E-08	5,06435E-07	1,17301E-08	7,457E-08	[4]	Group173	4,04	141,00
GO:0055086	nucleobase-containing small molecule metabolic process	GO_BiologicalProcess-EBI-UniProt-GOA-ACAP-ARAP_08.05.2020_00h00	0,003933324	0,009853902	1,17301E-08	7,457E-08	[3, 4, 5]	Group173	4,41	30,00

GO:0006644	phospholipid metabolic process	GO_BiologicalProcess-EBI-UniProt-GOA-ACAP-ARAP_08.05.2020_00h00	0,002634456	0,007393474	1,17301E-08	7,457E-08	[4, 5]	Group173	4,97	23,00
GO:0030258	lipid modification	GO_BiologicalProcess-EBI-UniProt-GOA-ACAP-ARAP_08.05.2020_00h00	0,027085837	0,043130025	1,17301E-08	7,457E-08	[4, 5]	Group173	4,56	15,00
GO:0046486	glycerolipid metabolic process	GO_BiologicalProcess-EBI-UniProt-GOA-ACAP-ARAP_08.05.2020_00h00	0,004992535	0,01194464	1,17301E-08	7,457E-08	[4, 5]	Group173	4,71	23,00
GO:0090407	organophosphate biosynthetic process	GO_BiologicalProcess-EBI-UniProt-GOA-ACAP-ARAP_08.05.2020_00h00	0,000779392	0,002936529	1,17301E-08	7,457E-08	[4, 5]	Group173	4,79	32,00
GO:0008654	phospholipid biosynthetic process	GO_BiologicalProcess-EBI-UniProt-GOA-ACAP-ARAP_08.05.2020_00h00	0,002142059	0,006386139	1,17301E-08	7,457E-08	[4, 5, 6]	Group173	5,74	17,00
GO:0045017	glycerolipid biosynthetic process	GO_BiologicalProcess-EBI-UniProt-GOA-ACAP-ARAP_08.05.2020_00h00	0,010909148	0,021886906	1,17301E-08	7,457E-08	[4, 5, 6]	Group173	5,10	15,00
GO:0006650	glycerophospholipid metabolic process	GO_BiologicalProcess-EBI-UniProt-GOA-ACAP-ARAP_08.05.2020_00h00	0,003137021	0,008339248	1,17301E-08	7,457E-08	[5, 6]	Group173	5,17	20,00
GO:0046834	lipid phosphorylation	GO_BiologicalProcess-EBI-UniProt-GOA-ACAP-ARAP_08.05.2020_00h00	0,005052869	0,011969296	1,17301E-08	7,457E-08	[5, 6]	Group173	7,32	9,00
GO:0009117	nucleotide metabolic process	GO_BiologicalProcess-EBI-UniProt-GOA-ACAP-ARAP_08.05.2020_00h00	0,022016035	0,036963765	1,17301E-08	7,457E-08	[5, 6, 7]	Group173	4,07	24,00
GO:0046488	phosphatidylinositol metabolic process	GO_BiologicalProcess-EBI-UniProt-GOA-ACAP-ARAP_08.05.2020_00h00	0,017771911	0,031437557	1,17301E-08	7,457E-08	[6, 7]	Group173	5,24	12,00
GO:0043550	regulation of lipid kinase activity	GO_BiologicalProcess-EBI-UniProt-GOA-ACAP-ARAP_08.05.2020_00h00	0,030797191	0,047081329	1,17301E-08	7,457E-08	[5, 6, 7, 8, 9]	Group173	7,46	5,00
GO:0046856	phosphatidylinositol dephosphorylation	GO_BiologicalProcess-EBI-UniProt-GOA-ACAP-ARAP_08.05.2020_00h00	0,007393209	0,016265061	1,17301E-08	7,457E-08	[6, 7, 8]	Group173	13,33	4,00
GO:0006974	cellular response to DNA damage stimulus	GO_BiologicalProcess-EBI-UniProt-GOA-ACAP-ARAP_08.05.2020_00h00	0,001050609	0,003642874	1,34163E-08	8,2348E-08	[4]	Group140	4,38	40,00
GO:0080135	regulation of cellular response to stress	GO_BiologicalProcess-EBI-UniProt-GOA-ACAP-ARAP_08.05.2020_00h00	5,18829E-08	9,54845E-07	1,34163E-08	8,2348E-08	[3, 4, 5]	Group140	6,08	48,00
GO:2001020	regulation of response to DNA damage stimulus	GO_BiologicalProcess-EBI-UniProt-GOA-ACAP-ARAP_08.05.2020_00h00	0,004286675	0,010465174	1,34163E-08	8,2348E-08	[4, 5, 6]	Group140	5,86	14,00
GO:2001021	negative regulation of response to DNA damage stimulus	GO_BiologicalProcess-EBI-UniProt-GOA-ACAP-ARAP_08.05.2020_00h00	0,030644687	0,046923146	1,34163E-08	8,2348E-08	[3, 4, 5, 6, 7]	Group140	6,67	6,00
GO:0051052	regulation of DNA metabolic process	GO_BiologicalProcess-EBI-UniProt-GOA-ACAP-ARAP_08.05.2020_00h00	0,002958667	0,008043876	1,34163E-08	8,2348E-08	[4, 5, 6, 7]	Group140	5,19	20,00
GO:0051053	negative regulation of DNA metabolic process	GO_BiologicalProcess-EBI-UniProt-GOA-ACAP-ARAP_08.05.2020_00h00	0,02470557	0,040278073	1,34163E-08	8,2348E-08	[4, 5, 6, 7, 8]	Group140	5,97	8,00
GO:2000780	negative regulation of double-strand break repair	GO_BiologicalProcess-EBI-UniProt-GOA-ACAP-ARAP_08.05.2020_00h00	0,014057252	0,026326399	1,34163E-08	8,2348E-08	[5, 6, 7, 8, 9, 10]	Group140	11,11	4,00
GO:0032970	regulation of actin filament-based process	GO_BiologicalProcess-EBI-UniProt-GOA-ACAP-ARAP_08.05.2020_00h00	0,000145525	0,000819218	1,52233E-08	9,0325E-08	[3, 4]	Group182	5,90	25,00
GO:0044087	regulation of cellular component biogenesis	GO_BiologicalProcess-EBI-UniProt-GOA-ACAP-ARAP_08.05.2020_00h00	0,003717563	0,00943689	1,52233E-08	9,0325E-08	[3, 4]	Group182	4,03	42,00
GO:0051128	regulation of cellular component organization	GO_BiologicalProcess-EBI-UniProt-GOA-ACAP-ARAP_08.05.2020_00h00	9,5472E-08	1,54859E-06	1,52233E-08	9,0325E-08	[3, 4]	Group182	4,27	109,00
GO:0044089	positive regulation of cellular component biogenesis	GO_BiologicalProcess-EBI-UniProt-GOA-ACAP-ARAP_08.05.2020_00h00	0,006975319	0,01552414	1,52233E-08	9,0325E-08	[3, 4, 5]	Group182	4,41	26,00

GO:0051130	positive regulation of cellular component organization	GO_BiologicalProcess-EBI-UniProt-GOA-ACAP-ARAP_08.05.2020_00h00	7,13305E-08	1,24115E-06	1,52233E-08	9,0325E-08	[3, 4, 5]	Group182	5,15	67,00
GO:0097435	supramolecular fiber organization	GO_BiologicalProcess-EBI-UniProt-GOA-ACAP-ARAP_08.05.2020_00h00	0,002547205	0,007233459	1,52233E-08	9,0325E-08	[4]	Group182	4,41	33,00
GO:0030036	actin cytoskeleton organization	GO_BiologicalProcess-EBI-UniProt-GOA-ACAP-ARAP_08.05.2020_00h00	0,000141026	0,000808154	1,52233E-08	9,0325E-08	[3, 6]	Group182	4,99	37,00
GO:0033043	regulation of organelle organization	GO_BiologicalProcess-EBI-UniProt-GOA-ACAP-ARAP_08.05.2020_00h00	0,000797572	0,002958436	1,52233E-08	9,0325E-08	[4, 5]	Group182	4,03	56,00
GO:1902903	regulation of supramolecular fiber organization	GO_BiologicalProcess-EBI-UniProt-GOA-ACAP-ARAP_08.05.2020_00h00	0,000770037	0,002912747	1,52233E-08	9,0325E-08	[4, 5]	Group182	5,57	22,00
GO:0010638	positive regulation of organelle organization	GO_BiologicalProcess-EBI-UniProt-GOA-ACAP-ARAP_08.05.2020_00h00	0,002543105	0,007243308	1,52233E-08	9,0325E-08	[4, 5, 6]	Group182	4,49	31,00
GO:1902905	positive regulation of supramolecular fiber organization	GO_BiologicalProcess-EBI-UniProt-GOA-ACAP-ARAP_08.05.2020_00h00	0,002495378	0,007149931	1,52233E-08	9,0325E-08	[4, 5, 6]	Group182	6,22	14,00
GO:0007015	actin filament organization	GO_BiologicalProcess-EBI-UniProt-GOA-ACAP-ARAP_08.05.2020_00h00	0,002237182	0,006607973	1,52233E-08	9,0325E-08	[4, 5, 7]	Group182	5,03	23,00
GO:0031032	actomyosin structure organization	GO_BiologicalProcess-EBI-UniProt-GOA-ACAP-ARAP_08.05.2020_00h00	0,000129235	0,000758763	1,52233E-08	9,0325E-08	[4, 7]	Group182	7,66	16,00
GO:0032956	regulation of actin cytoskeleton organization	GO_BiologicalProcess-EBI-UniProt-GOA-ACAP-ARAP_08.05.2020_00h00	0,000413217	0,001830783	1,52233E-08	9,0325E-08	[4, 5, 6, 7]	Group182	5,84	22,00
GO:0051493	regulation of cytoskeleton organization	GO_BiologicalProcess-EBI-UniProt-GOA-ACAP-ARAP_08.05.2020_00h00	0,003203749	0,008493042	1,52233E-08	9,0325E-08	[5, 6]	Group182	4,62	27,00
GO:1901890	positive regulation of cell junction assembly	GO_BiologicalProcess-EBI-UniProt-GOA-ACAP-ARAP_08.05.2020_00h00	0,023341313	0,038579684	1,52233E-08	9,0325E-08	[4, 5, 6, 7]	Group182	6,48	7,00
GO:0051495	positive regulation of cytoskeleton organization	GO_BiologicalProcess-EBI-UniProt-GOA-ACAP-ARAP_08.05.2020_00h00	0,013936384	0,026202591	1,52233E-08	9,0325E-08	[5, 6, 7]	Group182	5,24	13,00
GO:0110053	regulation of actin filament organization	GO_BiologicalProcess-EBI-UniProt-GOA-ACAP-ARAP_08.05.2020_00h00	0,000781211	0,002931841	1,52233E-08	9,0325E-08	[5, 6, 7, 8]	Group182	6,12	18,00
GO:0051017	actin filament bundle assembly	GO_BiologicalProcess-EBI-UniProt-GOA-ACAP-ARAP_08.05.2020_00h00	2,96766E-05	0,000225401	1,52233E-08	9,0325E-08	[5, 6, 7, 9]	Group182	9,09	15,00
GO:0032231	regulation of actin filament bundle assembly	GO_BiologicalProcess-EBI-UniProt-GOA-ACAP-ARAP_08.05.2020_00h00	5,44583E-06	5,48596E-05	1,52233E-08	9,0325E-08	[4, 5, 6, 7, 8, 9, 10]	Group182	11,93	13,00
GO:0032232	negative regulation of actin filament bundle assembly	GO_BiologicalProcess-EBI-UniProt-GOA-ACAP-ARAP_08.05.2020_00h00	0,010381774	0,021094177	1,52233E-08	9,0325E-08	[5, 6, 7, 8, 9, 10, 11]	Group182	12,12	4,00
GO:0043149	stress fiber assembly	GO_BiologicalProcess-EBI-UniProt-GOA-ACAP-ARAP_08.05.2020_00h00	7,36972E-06	6,91454E-05	1,52233E-08	9,0325E-08	[5, 7, 8, 9, 11]	Group182	11,61	13,00
GO:0051496	positive regulation of stress fiber assembly	GO_BiologicalProcess-EBI-UniProt-GOA-ACAP-ARAP_08.05.2020_00h00	0,00011102	0,000676726	1,52233E-08	9,0325E-08	[5, 6, 7, 8, 9, 10, 11, 12, 13]	Group182	14,04	8,00
GO:0015278	calcium-release channel activity	GO_BiologicalProcess-EBI-UniProt-GOA-ACAP-ARAP_08.05.2020_00h00	0,010487842	0,021219587	1,8002E-08	1,0337E-07	[5, 6, 7, 8, 9, 10, 11, 12, 13, 14, 15]	Group189	9,80	5,00
GO:0051281	positive regulation of release of sequestered calcium ion into cytosol	GO_BiologicalProcess-EBI-UniProt-GOA-ACAP-ARAP_08.05.2020_00h00	0,025549145	0,041231927	1,8002E-08	1,0337E-07	[4, 5, 6, 7, 8, 9, 10, 11, 12, 13, 14, 15, 16]	Group189	9,30	4,00
GO:0051235	maintenance of location	GO_BiologicalProcess-EBI-UniProt-GOA-ACAP-ARAP_08.05.2020_00h00	0,002857794	0,007836415	1,8002E-08	1,0337E-07	[2, 3]	Group189	5,32	19,00
GO:0019725	cellular homeostasis	GO_BiologicalProcess-EBI-UniProt-GOA-ACAP-ARAP_08.05.2020_00h00	4,43303E-05	0,000311942	1,8002E-08	1,0337E-07	[2, 4]	Group189	4,70	50,00

GO:0042592	homeostatic process	GO_BiologicalProcess-EBI-UniProt-GOA-ACAP-ARAP_08.05.2020_00h00	9,1536E-07	1,12308E-05	1,8002E-08	1,0337E-07	[3]	Group189	4,32	91,00
GO:0051651	maintenance of location in cell	GO_BiologicalProcess-EBI-UniProt-GOA-ACAP-ARAP_08.05.2020_00h00	0,00192218	0,005914877	1,8002E-08	1,0337E-07	[2, 3, 4]	Group189	6,17	15,00
GO:0051049	regulation of transport	GO_BiologicalProcess-EBI-UniProt-GOA-ACAP-ARAP_08.05.2020_00h00	6,30185E-05	0,00041881	1,8002E-08	1,0337E-07	[3, 4]	Group189	4,00	81,00
GO:0051050	positive regulation of transport	GO_BiologicalProcess-EBI-UniProt-GOA-ACAP-ARAP_08.05.2020_00h00	0,001033711	0,003597314	1,8002E-08	1,0337E-07	[2, 3, 4, 5]	Group189	4,20	46,00
GO:0051051	negative regulation of transport	GO_BiologicalProcess-EBI-UniProt-GOA-ACAP-ARAP_08.05.2020_00h00	0,020912321	0,035610486	1,8002E-08	1,0337E-07	[2, 3, 4, 5]	Group189	4,19	22,00
GO:0060341	regulation of cellular localization	GO_BiologicalProcess-EBI-UniProt-GOA-ACAP-ARAP_08.05.2020_00h00	0,000142641	0,000812543	1,8002E-08	1,0337E-07	[3, 4]	Group189	4,51	49,00
GO:0034762	regulation of transmembrane transport	GO_BiologicalProcess-EBI-UniProt-GOA-ACAP-ARAP_08.05.2020_00h00	0,009670558	0,020031869	1,8002E-08	1,0337E-07	[3, 4, 5]	Group189	4,29	26,00
GO:0048878	chemical homeostasis	GO_BiologicalProcess-EBI-UniProt-GOA-ACAP-ARAP_08.05.2020_00h00	0,000636798	0,002507882	1,8002E-08	1,0337E-07	[4]	Group189	4,13	53,00
GO:0055082	cellular chemical homeostasis	GO_BiologicalProcess-EBI-UniProt-GOA-ACAP-ARAP_08.05.2020_00h00	2,01483E-05	0,000163406	1,8002E-08	1,0337E-07	[3, 5]	Group189	5,04	45,00
GO:0019751	polyol metabolic process	GO_BiologicalProcess-EBI-UniProt-GOA-ACAP-ARAP_08.05.2020_00h00	0,006168251	0,014088344	1,8002E-08	1,0337E-07	[4, 5]	Group189	6,67	10,00
GO:0034763	negative regulation of transmembrane transport	GO_BiologicalProcess-EBI-UniProt-GOA-ACAP-ARAP_08.05.2020_00h00	0,021045569	0,035773728	1,8002E-08	1,0337E-07	[3, 4, 5, 6]	Group189	6,15	8,00
GO:0034764	positive regulation of transmembrane transport	GO_BiologicalProcess-EBI-UniProt-GOA-ACAP-ARAP_08.05.2020_00h00	0,031413406	0,047870431	1,8002E-08	1,0337E-07	[3, 4, 5, 6]	Group189	4,98	11,00
GO:0043269	regulation of ion transport	GO_BiologicalProcess-EBI-UniProt-GOA-ACAP-ARAP_08.05.2020_00h00	0,013630096	0,025880955	1,8002E-08	1,0337E-07	[4, 5]	Group189	4,02	30,00
GO:0043270	positive regulation of ion transport	GO_BiologicalProcess-EBI-UniProt-GOA-ACAP-ARAP_08.05.2020_00h00	0,029049521	0,045351375	1,8002E-08	1,0337E-07	[3, 4, 5, 6]	Group189	4,62	14,00
GO:0043271	negative regulation of ion transport	GO_BiologicalProcess-EBI-UniProt-GOA-ACAP-ARAP_08.05.2020_00h00	0,011685916	0,023058603	1,8002E-08	1,0337E-07	[3, 4, 5, 6]	Group189	6,06	10,00
GO:0046165	alcohol biosynthetic process	GO_BiologicalProcess-EBI-UniProt-GOA-ACAP-ARAP_08.05.2020_00h00	0,029087059	0,045336019	1,8002E-08	1,0337E-07	[4, 5]	Group189	5,24	10,00
GO:0034765	regulation of ion transmembrane transport	GO_BiologicalProcess-EBI-UniProt-GOA-ACAP-ARAP_08.05.2020_00h00	0,028976446	0,045385364	1,8002E-08	1,0337E-07	[4, 5, 6]	Group189	4,10	21,00
GO:0044070	regulation of anion transport	GO_BiologicalProcess-EBI-UniProt-GOA-ACAP-ARAP_08.05.2020_00h00	0,032775592	0,049240567	1,8002E-08	1,0337E-07	[5, 6]	Group189	6,03	7,00
GO:0046173	polyol biosynthetic process	GO_BiologicalProcess-EBI-UniProt-GOA-ACAP-ARAP_08.05.2020_00h00	0,00929126	0,019456752	1,8002E-08	1,0337E-07	[5, 6]	Group189	7,78	7,00
GO:0010959	regulation of metal ion transport	GO_BiologicalProcess-EBI-UniProt-GOA-ACAP-ARAP_08.05.2020_00h00	0,008230304	0,017739642	1,8002E-08	1,0337E-07	[5, 6, 7]	Group189	4,73	20,00
GO:0030001	metal ion transport	GO_BiologicalProcess-EBI-UniProt-GOA-ACAP-ARAP_08.05.2020_00h00	0,00093562	0,003353513	1,8002E-08	1,0337E-07	[6]	Group189	4,31	43,00
GO:0030003	cellular cation homeostasis	GO_BiologicalProcess-EBI-UniProt-GOA-ACAP-ARAP_08.05.2020_00h00	2,90438E-05	0,000224153	1,8002E-08	1,0337E-07	[5, 7]	Group189	5,32	38,00
GO:0032412	regulation of ion transmembrane transporter activity	GO_BiologicalProcess-EBI-UniProt-GOA-ACAP-ARAP_08.05.2020_00h00	0,00381815	0,009641081	1,8002E-08	1,0337E-07	[5, 6, 7]	Group189	5,57	16,00

GO:0050806	positive regulation of synaptic transmission	GO_BiologicalProcess-EBI-UniProt-GOA-ACAP-ARAP_08.05.2020_00h00	0,010380722	0,021136915	1,8002E-08	1,0337E-07	[3, 4, 5, 6, 7, 8, 9]	Group189	5,88	11,00
GO:0051208	sequestering of calcium ion	GO_BiologicalProcess-EBI-UniProt-GOA-ACAP-ARAP_08.05.2020_00h00	0,000410002	0,001824987	1,8002E-08	1,0337E-07	[3, 4, 5, 8, 10]	Group189	8,33	12,00
GO:0098771	inorganic ion homeostasis	GO_BiologicalProcess-EBI-UniProt-GOA-ACAP-ARAP_08.05.2020_00h00	0,000333665	0,001557644	1,8002E-08	1,0337E-07	[6]	Group189	4,73	38,00
GO:1904062	regulation of cation transmembrane transport	GO_BiologicalProcess-EBI-UniProt-GOA-ACAP-ARAP_08.05.2020_00h00	0,004210313	0,010358021	1,8002E-08	1,0337E-07	[5, 6, 7]	Group189	5,14	19,00
GO:0006875	cellular metal ion homeostasis	GO_BiologicalProcess-EBI-UniProt-GOA-ACAP-ARAP_08.05.2020_00h00	0,000161235	0,000881727	1,8002E-08	1,0337E-07	[6, 8]	Group189	5,18	33,00
GO:0070838	divalent metal ion transport	GO_BiologicalProcess-EBI-UniProt-GOA-ACAP-ARAP_08.05.2020_00h00	0,001574539	0,005056488	1,8002E-08	1,0337E-07	[7]	Group189	4,93	26,00
GO:0070588	calcium ion transmembrane transport	GO_BiologicalProcess-EBI-UniProt-GOA-ACAP-ARAP_08.05.2020_00h00	0,003038352	0,008144826	1,8002E-08	1,0337E-07	[6, 8, 9]	Group189	5,29	19,00
GO:0010522	regulation of calcium ion transport into cytosol	GO_BiologicalProcess-EBI-UniProt-GOA-ACAP-ARAP_08.05.2020_00h00	0,007866058	0,017108677	1,8002E-08	1,0337E-07	[3, 4, 5, 7, 8, 9, 10, 11, 13]	Group189	7,34	8,00
GO:0006874	cellular calcium ion homeostasis	GO_BiologicalProcess-EBI-UniProt-GOA-ACAP-ARAP_08.05.2020_00h00	6,50747E-05	0,000426551	1,8002E-08	1,0337E-07	[7, 9]	Group189	5,77	29,00
GO:2001257	regulation of cation channel activity	GO_BiologicalProcess-EBI-UniProt-GOA-ACAP-ARAP_08.05.2020_00h00	0,002512984	0,007178881	1,8002E-08	1,0337E-07	[6, 7, 8, 9, 10]	Group189	6,47	13,00
GO:0060402	calcium ion transport into cytosol	GO_BiologicalProcess-EBI-UniProt-GOA-ACAP-ARAP_08.05.2020_00h00	0,000377397	0,001703626	1,8002E-08	1,0337E-07	[4, 10, 12]	Group189	7,57	14,00
GO:0051209	release of sequestered calcium ion into cytosol	GO_BiologicalProcess-EBI-UniProt-GOA-ACAP-ARAP_08.05.2020_00h00	0,0002773	0,001333548	1,8002E-08	1,0337E-07	[4, 5, 6, 7, 8, 10, 11, 12, 13, 14]	Group189	8,70	12,00
GO:0051480	regulation of cytosolic calcium ion concentration	GO_BiologicalProcess-EBI-UniProt-GOA-ACAP-ARAP_08.05.2020_00h00	0,002075759	0,006286397	1,8002E-08	1,0337E-07	[8, 10]	Group189	5,25	21,00
GO:0033002	muscle cell proliferation	GO_BiologicalProcess-EBI-UniProt-GOA-ACAP-ARAP_08.05.2020_00h00	3,12928E-05	0,000233963	2,73016E-08	1,5187E-07	[3]	Group176	8,25	17,00
GO:0050673	epithelial cell proliferation	GO_BiologicalProcess-EBI-UniProt-GOA-ACAP-ARAP_08.05.2020_00h00	3,83793E-06	3,94935E-05	2,73016E-08	1,5187E-07	[3]	Group176	6,71	29,00
GO:0035019	somatic stem cell population maintenance	GO_BiologicalProcess-EBI-UniProt-GOA-ACAP-ARAP_08.05.2020_00h00	0,020465165	0,034911167	2,73016E-08	1,5187E-07	[3, 4]	Group176	7,32	6,00
GO:0001935	endothelial cell proliferation	GO_BiologicalProcess-EBI-UniProt-GOA-ACAP-ARAP_08.05.2020_00h00	0,003633375	0,00934715	2,73016E-08	1,5187E-07	[4]	Group176	6,79	11,00
GO:0008284	positive regulation of cell population proliferation	GO_BiologicalProcess-EBI-UniProt-GOA-ACAP-ARAP_08.05.2020_00h00	8,18119E-08	1,37358E-06	2,73016E-08	1,5187E-07	[3, 4, 5]	Group176	5,61	55,00
GO:0046620	regulation of organ growth	GO_BiologicalProcess-EBI-UniProt-GOA-ACAP-ARAP_08.05.2020_00h00	0,024403341	0,039853238	2,73016E-08	1,5187E-07	[3, 4, 5]	Group176	6,42	7,00
GO:0048659	smooth muscle cell proliferation	GO_BiologicalProcess-EBI-UniProt-GOA-ACAP-ARAP_08.05.2020_00h00	0,000131848	0,000764721	2,73016E-08	1,5187E-07	[4]	Group176	8,84	13,00
GO:0001655	urogenital system development	GO_BiologicalProcess-EBI-UniProt-GOA-ACAP-ARAP_08.05.2020_00h00	0,00374708	0,009486654	2,73016E-08	1,5187E-07	[4, 5]	Group176	5,19	19,00
GO:0048660	regulation of smooth muscle cell proliferation	GO_BiologicalProcess-EBI-UniProt-GOA-ACAP-ARAP_08.05.2020_00h00	0,000114782	0,000695231	2,73016E-08	1,5187E-07	[4, 5]	Group176	8,97	13,00
GO:0050678	regulation of epithelial cell proliferation	GO_BiologicalProcess-EBI-UniProt-GOA-ACAP-ARAP_08.05.2020_00h00	4,34628E-05	0,000310403	2,73016E-08	1,5187E-07	[4, 5]	Group176	6,50	24,00

GO:0048661	positive regulation of smooth muscle cell proliferation	GO_BiologicalProcess-EBI-UniProt-GOA-ACAP-ARAP_08.05.2020_00h00	0,00013222	0,000762259	2,73016E-08	1,5187E-07	[4, 5, 6]	Group176	10,99	10,00
GO:0050679	positive regulation of epithelial cell proliferation	GO_BiologicalProcess-EBI-UniProt-GOA-ACAP-ARAP_08.05.2020_00h00	0,00018895	0,000982745	2,73016E-08	1,5187E-07	[4, 5, 6]	Group176	7,41	16,00
GO:0072001	renal system development	GO_BiologicalProcess-EBI-UniProt-GOA-ACAP-ARAP_08.05.2020_00h00	0,010179704	0,02081619	2,73016E-08	1,5187E-07	[4, 5, 6]	Group176	5,02	16,00
GO:1990874	vascular smooth muscle cell proliferation	GO_BiologicalProcess-EBI-UniProt-GOA-ACAP-ARAP_08.05.2020_00h00	0,003044682	0,008138997	2,73016E-08	1,5187E-07	[5]	Group176	10,91	6,00
GO:0001822	kidney development	GO_BiologicalProcess-EBI-UniProt-GOA-ACAP-ARAP_08.05.2020_00h00	0,032703687	0,049209792	2,73016E-08	1,5187E-07	[4, 5, 6, 7]	Group176	4,55	14,00
GO:0001936	regulation of endothelial cell proliferation	GO_BiologicalProcess-EBI-UniProt-GOA-ACAP-ARAP_08.05.2020_00h00	0,014143549	0,026282284	2,73016E-08	1,5187E-07	[5, 6]	Group176	6,21	9,00
GO:1904705	regulation of vascular smooth muscle cell proliferation	GO_BiologicalProcess-EBI-UniProt-GOA-ACAP-ARAP_08.05.2020_00h00	0,003044682	0,008138997	2,73016E-08	1,5187E-07	[5, 6]	Group176	10,91	6,00
GO:0001938	positive regulation of endothelial cell proliferation	GO_BiologicalProcess-EBI-UniProt-GOA-ACAP-ARAP_08.05.2020_00h00	0,02131371	0,036101274	2,73016E-08	1,5187E-07	[5, 6, 7]	Group176	6,60	7,00
GO:0055024	regulation of cardiac muscle tissue development	GO_BiologicalProcess-EBI-UniProt-GOA-ACAP-ARAP_08.05.2020_00h00	0,008760029	0,018588355	2,73016E-08	1,5187E-07	[5, 6, 7, 8, 9]	Group176	7,87	7,00
GO:0033554	cellular response to stress	GO_BiologicalProcess-EBI-UniProt-GOA-ACAP-ARAP_08.05.2020_00h00	1,66634E-08	3,98672E-07	3,63422E-08	1,9026E-07	[3]	Group141	4,55	100,00
GO:0006974	cellular response to DNA damage stimulus	GO_BiologicalProcess-EBI-UniProt-GOA-ACAP-ARAP_08.05.2020_00h00	0,001050609	0,003642874	3,63422E-08	1,9026E-07	[4]	Group141	4,38	40,00
GO:0000075	cell cycle checkpoint	GO_BiologicalProcess-EBI-UniProt-GOA-ACAP-ARAP_08.05.2020_00h00	0,003692508	0,00942328	3,63422E-08	1,9026E-07	[2, 4, 5, 6]	Group141	5,96	14,00
GO:0042770	signal transduction in response to DNA damage	GO_BiologicalProcess-EBI-UniProt-GOA-ACAP-ARAP_08.05.2020_00h00	0,001526183	0,004951043	3,63422E-08	1,9026E-07	[4, 5, 6]	Group141	7,59	11,00
GO:0072331	signal transduction by p53 class mediator	GO_BiologicalProcess-EBI-UniProt-GOA-ACAP-ARAP_08.05.2020_00h00	0,010597887	0,021351954	3,63422E-08	1,9026E-07	[4, 5, 6]	Group141	5,12	15,00
GO:2001020	regulation of response to DNA damage stimulus	GO_BiologicalProcess-EBI-UniProt-GOA-ACAP-ARAP_08.05.2020_00h00	0,004286675	0,010465174	3,63422E-08	1,9026E-07	[4, 5, 6]	Group141	5,86	14,00
GO:0000077	DNA damage checkpoint	GO_BiologicalProcess-EBI-UniProt-GOA-ACAP-ARAP_08.05.2020_00h00	0,003633375	0,00934715	3,63422E-08	1,9026E-07	[4, 5, 6, 7, 8]	Group141	6,79	11,00
GO:0009611	response to wounding	GO_BiologicalProcess-EBI-UniProt-GOA-ACAP-ARAP_08.05.2020_00h00	2,09631E-06	2,33275E-05	3,54375E-08	1,9115E-07	[3]	Group165	5,69	42,00
GO:0050878	regulation of body fluid levels	GO_BiologicalProcess-EBI-UniProt-GOA-ACAP-ARAP_08.05.2020_00h00	0,001702964	0,005378668	3,54375E-08	1,9115E-07	[3]	Group165	4,84	27,00
GO:0007599	hemostasis	GO_BiologicalProcess-EBI-UniProt-GOA-ACAP-ARAP_08.05.2020_00h00	0,001814493	0,00563789	3,54375E-08	1,9115E-07	[4]	Group165	5,42	20,00
GO:0042060	wound healing	GO_BiologicalProcess-EBI-UniProt-GOA-ACAP-ARAP_08.05.2020_00h00	7,38149E-05	0,000477303	3,54375E-08	1,9115E-07	[4]	Group165	5,40	33,00
GO:0033628	regulation of cell adhesion mediated by integrin	GO_BiologicalProcess-EBI-UniProt-GOA-ACAP-ARAP_08.05.2020_00h00	0,009662041	0,020057641	3,54375E-08	1,9115E-07	[4, 5]	Group165	10,00	5,00
GO:0051604	protein maturation	GO_BiologicalProcess-EBI-UniProt-GOA-ACAP-ARAP_08.05.2020_00h00	0,029049521	0,045351375	3,54375E-08	1,9115E-07	[4, 5]	Group165	4,62	14,00
GO:1903034	regulation of response to wounding	GO_BiologicalProcess-EBI-UniProt-GOA-ACAP-ARAP_08.05.2020_00h00	0,000707064	0,00271751	3,54375E-08	1,9115E-07	[4, 5]	Group165	7,11	14,00

GO:1903035	negative regulation of response to wounding	GO_BiologicalProcess-EBI-UniProt-GOA-ACAP-ARAP_08.05.2020_00h00	0,005007775	0,011891912	3,54375E-08	1,9115E-07	[3, 4, 5, 6]	Group165	7,92	8,00
GO:0030193	regulation of blood coagulation	GO_BiologicalProcess-EBI-UniProt-GOA-ACAP-ARAP_08.05.2020_00h00	0,000625023	0,002481938	3,54375E-08	1,9115E-07	[3, 4, 5, 6, 7]	Group165	9,89	9,00
GO:0014909	smooth muscle cell migration	GO_BiologicalProcess-EBI-UniProt-GOA-ACAP-ARAP_08.05.2020_00h00	0,000144714	0,000819473	3,54375E-08	1,9115E-07	[5, 6]	Group165	12,00	9,00
GO:0061041	regulation of wound healing	GO_BiologicalProcess-EBI-UniProt-GOA-ACAP-ARAP_08.05.2020_00h00	0,001230309	0,004145795	3,54375E-08	1,9115E-07	[5, 6]	Group165	7,36	12,00
GO:0042730	fibrinolysis	GO_BiologicalProcess-EBI-UniProt-GOA-ACAP-ARAP_08.05.2020_00h00	0,007393209	0,016265061	3,54375E-08	1,9115E-07	[4, 5, 6, 7, 8, 9]	Group165	13,33	4,00
GO:0031639	plasminogen activation	GO_BiologicalProcess-EBI-UniProt-GOA-ACAP-ARAP_08.05.2020_00h00	0,003242962	0,008549627	3,54375E-08	1,9115E-07	[7, 8]	Group165	16,67	4,00
GO:0044087	regulation of cellular component biogenesis	GO_BiologicalProcess-EBI-UniProt-GOA-ACAP-ARAP_08.05.2020_00h00	0,003717563	0,00943689	8,61421E-08	4,3809E-07	[3, 4]	Group150	4,03	42,00
GO:0051128	regulation of cellular component organization	GO_BiologicalProcess-EBI-UniProt-GOA-ACAP-ARAP_08.05.2020_00h00	9,5472E-08	1,54859E-06	8,61421E-08	4,3809E-07	[3, 4]	Group150	4,27	109,00
GO:0044089	positive regulation of cellular component biogenesis	GO_BiologicalProcess-EBI-UniProt-GOA-ACAP-ARAP_08.05.2020_00h00	0,006975319	0,01552414	8,61421E-08	4,3809E-07	[3, 4, 5]	Group150	4,41	26,00
GO:0051130	positive regulation of cellular component organization	GO_BiologicalProcess-EBI-UniProt-GOA-ACAP-ARAP_08.05.2020_00h00	7,13305E-08	1,24115E-06	8,61421E-08	4,3809E-07	[3, 4, 5]	Group150	5,15	67,00
GO:0031344	regulation of cell projection organization	GO_BiologicalProcess-EBI-UniProt-GOA-ACAP-ARAP_08.05.2020_00h00	0,004833171	0,011592343	8,61421E-08	4,3809E-07	[4, 5]	Group150	4,26	32,00
GO:0033043	regulation of organelle organization	GO_BiologicalProcess-EBI-UniProt-GOA-ACAP-ARAP_08.05.2020_00h00	0,000797572	0,002958436	8,61421E-08	4,3809E-07	[4, 5]	Group150	4,03	56,00
GO:0010638	positive regulation of organelle organization	GO_BiologicalProcess-EBI-UniProt-GOA-ACAP-ARAP_08.05.2020_00h00	0,002543105	0,007243308	8,61421E-08	4,3809E-07	[4, 5, 6]	Group150	4,49	31,00
GO:0031346	positive regulation of cell projection organization	GO_BiologicalProcess-EBI-UniProt-GOA-ACAP-ARAP_08.05.2020_00h00	0,009294322	0,019420668	8,61421E-08	4,3809E-07	[4, 5, 6]	Group150	4,67	20,00
GO:0120035	regulation of plasma membrane bounded cell projection organization	GO_BiologicalProcess-EBI-UniProt-GOA-ACAP-ARAP_08.05.2020_00h00	0,012516662	0,024396019	8,61421E-08	4,3809E-07	[5, 6]	Group150	4,04	30,00
GO:0090287	regulation of cellular response to growth factor stimulus	GO_BiologicalProcess-EBI-UniProt-GOA-ACAP-ARAP_08.05.2020_00h00	0,00110216	0,003767027	9,16346E-08	4,5308E-07	[3, 4, 6]	Group169	5,94	18,00
GO:0032633	interleukin-4 production	GO_BiologicalProcess-EBI-UniProt-GOA-ACAP-ARAP_08.05.2020_00h00	0,002621352	0,007400101	9,16346E-08	4,5308E-07	[3, 6]	Group169	13,51	5,00
GO:0003170	heart valve development	GO_BiologicalProcess-EBI-UniProt-GOA-ACAP-ARAP_08.05.2020_00h00	0,029100992	0,045283981	9,16346E-08	4,5308E-07	[3, 5, 6, 7]	Group169	7,58	5,00
GO:0001890	placenta development	GO_BiologicalProcess-EBI-UniProt-GOA-ACAP-ARAP_08.05.2020_00h00	0,00194322	0,005960454	9,16346E-08	4,5308E-07	[4, 5, 6, 7]	Group169	6,98	12,00
GO:0001892	embryonic placenta development	GO_BiologicalProcess-EBI-UniProt-GOA-ACAP-ARAP_08.05.2020_00h00	0,017631847	0,031363714	9,16346E-08	4,5308E-07	[3, 4, 5, 6, 7, 8]	Group169	6,86	7,00
GO:0002011	morphogenesis of an epithelial sheet	GO_BiologicalProcess-EBI-UniProt-GOA-ACAP-ARAP_08.05.2020_00h00	0,000160526	0,000882894	9,16346E-08	4,5308E-07	[5, 6]	Group169	13,33	8,00
GO:0060674	placenta blood vessel development	GO_BiologicalProcess-EBI-UniProt-GOA-ACAP-ARAP_08.05.2020_00h00	0,003317585	0,008674669	9,16346E-08	4,5308E-07	[3, 4, 5, 6, 7, 8, 9]	Group169	12,82	5,00
GO:0090092	regulation of transmembrane receptor protein serine/threonine kinase signaling pathway	GO_BiologicalProcess-EBI-UniProt-GOA-ACAP-ARAP_08.05.2020_00h00	0,018717377	0,032806829	9,16346E-08	4,5308E-07	[4, 5, 6, 7, 8]	Group169	5,04	13,00

GO:1903556	negative regulation of tumor necrosis factor superfamily cytokine production	GO_BiologicalProcess-EBI-UniProt-GOA-ACAP-ARAP_08.05.2020_00h00	0,029100992	0,045283981	9,16346E-08	4,5308E-07	[4, 5, 6, 7, 8]	Group169	7,58	5,00
GO:0030510	regulation of BMP signaling pathway	GO_BiologicalProcess-EBI-UniProt-GOA-ACAP-ARAP_08.05.2020_00h00	0,013690378	0,025892671	9,16346E-08	4,5308E-07	[4, 5, 6, 7, 8, 9]	Group169	7,22	7,00
GO:0072602	interleukin-4 secretion	GO_BiologicalProcess-EBI-UniProt-GOA-ACAP-ARAP_08.05.2020_00h00	0,000575675	0,002344345	9,16346E-08	4,5308E-07	[4, 5, 6, 7, 8, 9, 10]	Group169	42,86	3,00
GO:0097028	dendritic cell differentiation	GO_BiologicalProcess-EBI-UniProt-GOA-ACAP-ARAP_08.05.2020_00h00	0,029624119	0,045948595	9,16346E-08	4,5308E-07	[5, 6, 7, 8, 9]	Group169	8,89	4,00
GO:0030513	positive regulation of BMP signaling pathway	GO_BiologicalProcess-EBI-UniProt-GOA-ACAP-ARAP_08.05.2020_00h00	0,016912684	0,030253155	9,16346E-08	4,5308E-07	[5, 6, 7, 8, 9, 10]	Group169	10,53	4,00
GO:2000725	regulation of cardiac muscle cell differentiation	GO_BiologicalProcess-EBI-UniProt-GOA-ACAP-ARAP_08.05.2020_00h00	0,020105848	0,034731582	9,16346E-08	4,5308E-07	[5, 6, 7, 8, 9, 10]	Group169	10,00	4,00
GO:0034097	response to cytokine	GO_BiologicalProcess-EBI-UniProt-GOA-ACAP-ARAP_08.05.2020_00h00	2,97291E-07	4,31072E-06	1,8928E-07	9,1059E-07	[4]	Group115	5,05	64,00
GO:0070555	response to interleukin-1	GO_BiologicalProcess-EBI-UniProt-GOA-ACAP-ARAP_08.05.2020_00h00	1,33456E-05	0,000114034	1,8928E-07	9,1059E-07	[5]	Group115	8,45	18,00
GO:1904019	epithelial cell apoptotic process	GO_BiologicalProcess-EBI-UniProt-GOA-ACAP-ARAP_08.05.2020_00h00	3,38977E-05	0,000249539	1,8928E-07	9,1059E-07	[5]	Group115	10,81	12,00
GO:0071347	cellular response to interleukin-1	GO_BiologicalProcess-EBI-UniProt-GOA-ACAP-ARAP_08.05.2020_00h00	0,000117157	0,000700744	1,8928E-07	9,1059E-07	[6]	Group115	8,06	15,00
GO:0071356	cellular response to tumor necrosis factor	GO_BiologicalProcess-EBI-UniProt-GOA-ACAP-ARAP_08.05.2020_00h00	0,012930495	0,024948554	1,8928E-07	9,1059E-07	[6]	Group115	5,00	15,00
GO:0071222	cellular response to lipopolysaccharide	GO_BiologicalProcess-EBI-UniProt-GOA-ACAP-ARAP_08.05.2020_00h00	0,006491162	0,014651042	1,8928E-07	9,1059E-07	[5, 6, 8]	Group115	5,78	13,00
GO:0043491	protein kinase B signaling	GO_BiologicalProcess-EBI-UniProt-GOA-ACAP-ARAP_08.05.2020_00h00	7,17147E-05	0,000466877	4,96615E-07	2,3262E-06	[4, 5, 6]	Group172	7,20	19,00
GO:1901888	regulation of cell junction assembly	GO_BiologicalProcess-EBI-UniProt-GOA-ACAP-ARAP_08.05.2020_00h00	0,029677309	0,045956612	4,96615E-07	2,3262E-06	[4, 5, 6]	Group172	5,02	11,00
GO:0051896	regulation of protein kinase B signaling	GO_BiologicalProcess-EBI-UniProt-GOA-ACAP-ARAP_08.05.2020_00h00	0,000195626	0,001011969	4,96615E-07	2,3262E-06	[5, 6, 7]	Group172	7,11	17,00
GO:0051897	positive regulation of protein kinase B signaling	GO_BiologicalProcess-EBI-UniProt-GOA-ACAP-ARAP_08.05.2020_00h00	8,10058E-05	0,000506683	4,96615E-07	2,3262E-06	[5, 6, 7, 8]	Group172	8,33	15,00
GO:0043549	regulation of kinase activity	GO_BiologicalProcess-EBI-UniProt-GOA-ACAP-ARAP_08.05.2020_00h00	1,8027E-06	2,05379E-05	4,96615E-07	2,3262E-06	[5, 7, 8]	Group172	5,25	51,00
GO:0042327	positive regulation of phosphorylation	GO_BiologicalProcess-EBI-UniProt-GOA-ACAP-ARAP_08.05.2020_00h00	3,4551E-08	6,74801E-07	4,96615E-07	2,3262E-06	[6, 7, 8]	Group172	5,40	63,00
GO:0004707	MAP kinase activity	GO_BiologicalProcess-EBI-UniProt-GOA-ACAP-ARAP_08.05.2020_00h00	0,004993279	0,011916628	4,96615E-07	2,3262E-06	[5, 6, 7, 9, 10]	Group172	5,05	19,00
GO:0004672	protein kinase activity	GO_BiologicalProcess-EBI-UniProt-GOA-ACAP-ARAP_08.05.2020_00h00	4,63941E-05	0,000324081	4,96615E-07	2,3262E-06	[7, 8]	Group172	4,46	58,00
GO:0018212	peptidyl-tyrosine modification	GO_BiologicalProcess-EBI-UniProt-GOA-ACAP-ARAP_08.05.2020_00h00	0,00348192	0,00900594	4,96615E-07	2,3262E-06	[7, 8]	Group172	5,02	21,00
GO:0033674	positive regulation of kinase activity	GO_BiologicalProcess-EBI-UniProt-GOA-ACAP-ARAP_08.05.2020_00h00	1,62283E-05	0,000136232	4,96615E-07	2,3262E-06	[6, 7, 8, 9]	Group172	5,53	37,00
GO:0043408	regulation of MAPK cascade	GO_BiologicalProcess-EBI-UniProt-GOA-ACAP-ARAP_08.05.2020_00h00	3,12878E-06	3,36432E-05	4,96615E-07	2,3262E-06	[5, 6, 7, 8, 9, 10]	Group172	5,54	43,00

GO:0043410	positive regulation of MAPK cascade	GO_BiologicalProcess-EBI-UniProt-GOA-ACAP-ARAP_08.05.2020_00h00	2,28998E-07	3,37156E-06	4,96615E-07	2,3262E-06	[5, 6, 7, 8, 9, 10, 11]	Group172	6,54	38,00
GO:0019199	transmembrane receptor protein kinase activity	GO_BiologicalProcess-EBI-UniProt-GOA-ACAP-ARAP_08.05.2020_00h00	0,003846464	0,009687016	4,96615E-07	2,3262E-06	[8, 9]	Group172	7,63	9,00
GO:0043405	regulation of MAP kinase activity	GO_BiologicalProcess-EBI-UniProt-GOA-ACAP-ARAP_08.05.2020_00h00	0,00374708	0,009486654	4,96615E-07	2,3262E-06	[6, 7, 8, 9, 10, 11]	Group172	5,19	19,00
GO:0004713	protein tyrosine kinase activity	GO_BiologicalProcess-EBI-UniProt-GOA-ACAP-ARAP_08.05.2020_00h00	0,017674305	0,031322796	4,96615E-07	2,3262E-06	[8, 9, 10]	Group172	5,08	13,00
GO:0032147	activation of protein kinase activity	GO_BiologicalProcess-EBI-UniProt-GOA-ACAP-ARAP_08.05.2020_00h00	2,21901E-05	0,000176966	4,96615E-07	2,3262E-06	[8, 9, 10, 11]	Group172	6,48	26,00
GO:0031098	stress-activated protein kinase signaling cascade	GO_BiologicalProcess-EBI-UniProt-GOA-ACAP-ARAP_08.05.2020_00h00	0,011678319	0,023091221	7,73732E-07	3,5314E-06	[4, 5, 6]	Group132	4,94	16,00
GO:0023014	signal transduction by protein phosphorylation	GO_BiologicalProcess-EBI-UniProt-GOA-ACAP-ARAP_08.05.2020_00h00	6,64101E-07	8,58844E-06	7,73732E-07	3,5314E-06	[3, 4, 5, 7, 8]	Group132	5,34	53,00
GO:0000165	MAPK cascade	GO_BiologicalProcess-EBI-UniProt-GOA-ACAP-ARAP_08.05.2020_00h00	1,91283E-06	2,15362E-05	7,73732E-07	3,5314E-06	[4, 5, 6, 8, 9]	Group132	5,24	51,00
GO:0043408	regulation of MAPK cascade	GO_BiologicalProcess-EBI-UniProt-GOA-ACAP-ARAP_08.05.2020_00h00	3,12878E-06	3,36432E-05	7,73732E-07	3,5314E-06	[5, 6, 7, 8, 9, 10]	Group132	5,54	43,00
GO:0043410	positive regulation of MAPK cascade	GO_BiologicalProcess-EBI-UniProt-GOA-ACAP-ARAP_08.05.2020_00h00	2,28998E-07	3,37156E-06	7,73732E-07	3,5314E-06	[5, 6, 7, 8, 9, 10, 11]	Group132	6,54	38,00
GO:0007254	JNK cascade	GO_BiologicalProcess-EBI-UniProt-GOA-ACAP-ARAP_08.05.2020_00h00	0,030536547	0,046832494	7,73732E-07	3,5314E-06	[6, 7, 8, 10, 11]	Group132	5,00	11,00
GO:0001776	leukocyte homeostasis	GO_BiologicalProcess-EBI-UniProt-GOA-ACAP-ARAP_08.05.2020_00h00	0,015182317	0,02772801	8,12637E-07	3,6162E-06	[2, 5]	Group136	7,07	7,00
GO:0002262	myeloid cell homeostasis	GO_BiologicalProcess-EBI-UniProt-GOA-ACAP-ARAP_08.05.2020_00h00	0,000130379	0,000760808	8,12637E-07	3,6162E-06	[2, 5]	Group136	8,38	14,00
GO:0048872	homeostasis of number of cells	GO_BiologicalProcess-EBI-UniProt-GOA-ACAP-ARAP_08.05.2020_00h00	0,00023294	0,001161058	8,12637E-07	3,6162E-06	[4]	Group136	6,57	19,00
GO:0034101	erythrocyte homeostasis	GO_BiologicalProcess-EBI-UniProt-GOA-ACAP-ARAP_08.05.2020_00h00	0,000242113	0,00120053	8,12637E-07	3,6162E-06	[3, 6]	Group136	8,82	12,00
GO:0030099	myeloid cell differentiation	GO_BiologicalProcess-EBI-UniProt-GOA-ACAP-ARAP_08.05.2020_00h00	6,2456E-06	5,97704E-05	8,12637E-07	3,6162E-06	[4, 5, 6, 7, 8]	Group136	6,55	29,00
GO:0030218	erythrocyte differentiation	GO_BiologicalProcess-EBI-UniProt-GOA-ACAP-ARAP_08.05.2020_00h00	0,001763233	0,005550702	8,12637E-07	3,6162E-06	[4, 5, 6, 7, 8, 9]	Group136	7,94	10,00
GO:0043249	erythrocyte maturation	GO_BiologicalProcess-EBI-UniProt-GOA-ACAP-ARAP_08.05.2020_00h00	0,012620698	0,024499003	8,12637E-07	3,6162E-06	[4, 5, 6, 7, 8, 9, 10, 11]	Group136	15,79	3,00
GO:0042592	homeostatic process	GO_BiologicalProcess-EBI-UniProt-GOA-ACAP-ARAP_08.05.2020_00h00	9,1536E-07	1,12308E-05	9,1536E-07	3,974E-06	[3]	Group097	4,32	91,00
GO:0048871	multicellular organismal homeostasis	GO_BiologicalProcess-EBI-UniProt-GOA-ACAP-ARAP_08.05.2020_00h00	0,002410506	0,006969347	9,1536E-07	3,974E-06	[2, 4]	Group097	4,72	27,00
GO:0001894	tissue homeostasis	GO_BiologicalProcess-EBI-UniProt-GOA-ACAP-ARAP_08.05.2020_00h00	0,023241255	0,038480762	9,1536E-07	3,974E-06	[3, 5]	Group097	4,76	14,00
GO:0060249	anatomical structure homeostasis	GO_BiologicalProcess-EBI-UniProt-GOA-ACAP-ARAP_08.05.2020_00h00	0,020128882	0,03470872	9,1536E-07	3,974E-06	[4]	Group097	4,21	22,00
GO:0035633	maintenance of blood-brain barrier	GO_BiologicalProcess-EBI-UniProt-GOA-ACAP-ARAP_08.05.2020_00h00	0,000421301	0,001849472	9,1536E-07	3,974E-06	[4, 6]	Group097	15,79	6,00
GO:0019725	cellular homeostasis	GO_BiologicalProcess-EBI-UniProt-GOA-ACAP-ARAP_08.05.2020_00h00	4,43303E-05	0,000311942	9,1536E-07	3,974E-06	[2, 4]	Group149	4,70	50,00

GO:0042592	homeostatic process	GO_BiologicalProcess-EBI-UniProt-GOA-ACAP-ARAP_08.05.2020_00h00	9,1536E-07	1,12308E-05	9,1536E-07	3,974E-06	[3]	Group149	4,32	91,00
GO:0048871	multicellular organismal homeostasis	GO_BiologicalProcess-EBI-UniProt-GOA-ACAP-ARAP_08.05.2020_00h00	0,002410506	0,006969347	9,1536E-07	3,974E-06	[2, 4]	Group149	4,72	27,00
GO:0048878	chemical homeostasis	GO_BiologicalProcess-EBI-UniProt-GOA-ACAP-ARAP_08.05.2020_00h00	0,000636798	0,002507882	9,1536E-07	3,974E-06	[4]	Group149	4,13	53,00
GO:0055082	cellular chemical homeostasis	GO_BiologicalProcess-EBI-UniProt-GOA-ACAP-ARAP_08.05.2020_00h00	2,01483E-05	0,000163406	9,1536E-07	3,974E-06	[3, 5]	Group149	5,04	45,00
GO:0030003	cellular cation homeostasis	GO_BiologicalProcess-EBI-UniProt-GOA-ACAP-ARAP_08.05.2020_00h00	2,90438E-05	0,000224153	9,1536E-07	3,974E-06	[5, 7]	Group149	5,32	38,00
GO:0098771	inorganic ion homeostasis	GO_BiologicalProcess-EBI-UniProt-GOA-ACAP-ARAP_08.05.2020_00h00	0,000333665	0,001557644	9,1536E-07	3,974E-06	[6]	Group149	4,73	38,00
GO:0006875	cellular metal ion homeostasis	GO_BiologicalProcess-EBI-UniProt-GOA-ACAP-ARAP_08.05.2020_00h00	0,000161235	0,000881727	9,1536E-07	3,974E-06	[6, 8]	Group149	5,18	33,00
GO:0006874	cellular calcium ion homeostasis	GO_BiologicalProcess-EBI-UniProt-GOA-ACAP-ARAP_08.05.2020_00h00	6,50747E-05	0,000426551	9,1536E-07	3,974E-06	[7, 9]	Group149	5,77	29,00
GO:0045321	leukocyte activation	GO_BiologicalProcess-EBI-UniProt-GOA-ACAP-ARAP_08.05.2020_00h00	7,48312E-05	0,000477423	9,40576E-07	3,9863E-06	[2, 3]	Group178	4,32	61,00
GO:0002263	cell activation involved in immune response	GO_BiologicalProcess-EBI-UniProt-GOA-ACAP-ARAP_08.05.2020_00h00	1,98211E-05	0,000163524	9,40576E-07	3,9863E-06	[3]	Group178	5,24	41,00
GO:0140352	export from cell	GO_BiologicalProcess-EBI-UniProt-GOA-ACAP-ARAP_08.05.2020_00h00	9,53733E-05	0,000588853	9,40576E-07	3,9863E-06	[2, 4]	Group178	4,10	71,00
GO:0002274	myeloid leukocyte activation	GO_BiologicalProcess-EBI-UniProt-GOA-ACAP-ARAP_08.05.2020_00h00	0,001295747	0,004350983	9,40576E-07	3,9863E-06	[3, 4]	Group178	4,60	33,00
GO:0046649	lymphocyte activation	GO_BiologicalProcess-EBI-UniProt-GOA-ACAP-ARAP_08.05.2020_00h00	0,001648926	0,0052426	9,40576E-07	3,9863E-06	[3, 4]	Group178	4,41	36,00
GO:0051049	regulation of transport	GO_BiologicalProcess-EBI-UniProt-GOA-ACAP-ARAP_08.05.2020_00h00	6,30185E-05	0,00041881	9,40576E-07	3,9863E-06	[3, 4]	Group178	4,00	81,00
GO:0002520	immune system development	GO_BiologicalProcess-EBI-UniProt-GOA-ACAP-ARAP_08.05.2020_00h00	5,84203E-07	7,76503E-06	9,40576E-07	3,9863E-06	[2, 4, 5]	Group178	5,23	56,00
GO:0002444	myeloid leukocyte mediated immunity	GO_BiologicalProcess-EBI-UniProt-GOA-ACAP-ARAP_08.05.2020_00h00	0,000784727	0,00293353	9,40576E-07	3,9863E-06	[4]	Group178	4,90	30,00
GO:0002275	myeloid cell activation involved in immune response	GO_BiologicalProcess-EBI-UniProt-GOA-ACAP-ARAP_08.05.2020_00h00	0,001343737	0,004480686	9,40576E-07	3,9863E-06	[4, 5]	Group178	4,79	29,00
GO:0002285	lymphocyte activation involved in immune response	GO_BiologicalProcess-EBI-UniProt-GOA-ACAP-ARAP_08.05.2020_00h00	0,004762104	0,01147943	9,40576E-07	3,9863E-06	[4, 5]	Group178	6,25	12,00
GO:0006887	exocytosis	GO_BiologicalProcess-EBI-UniProt-GOA-ACAP-ARAP_08.05.2020_00h00	0,000864268	0,003132973	9,40576E-07	3,9863E-06	[3, 4, 5, 6]	Group178	4,33	43,00
GO:0042110	T cell activation	GO_BiologicalProcess-EBI-UniProt-GOA-ACAP-ARAP_08.05.2020_00h00	0,002095667	0,006306772	9,40576E-07	3,9863E-06	[4, 5]	Group178	4,90	25,00
GO:0051249	regulation of lymphocyte activation	GO_BiologicalProcess-EBI-UniProt-GOA-ACAP-ARAP_08.05.2020_00h00	0,021119829	0,035836305	9,40576E-07	3,9863E-06	[4, 5, 6]	Group178	4,14	23,00
GO:0030097	hemopoiesis	GO_BiologicalProcess-EBI-UniProt-GOA-ACAP-ARAP_08.05.2020_00h00	2,93906E-05	0,000225015	9,40576E-07	3,9863E-06	[4, 5, 6, 7]	Group178	4,89	47,00
GO:0043299	leukocyte degranulation	GO_BiologicalProcess-EBI-UniProt-GOA-ACAP-ARAP_08.05.2020_00h00	0,000458366	0,001958285	9,40576E-07	3,9863E-06	[3, 4, 5, 6, 7, 8]	Group178	5,07	30,00
GO:0045055	regulated exocytosis	GO_BiologicalProcess-EBI-UniProt-GOA-ACAP-ARAP_08.05.2020_00h00	0,000882685	0,00318766	9,40576E-07	3,9863E-06	[4, 5, 6, 7]	Group178	4,45	39,00

GO:0002521	leukocyte differentiation	GO_BiologicalProcess-EBI-UniProt-GOA-ACAP-ARAP_08.05.2020_00h00	0,00341803	0,008912955	9,40576E-07	3,9863E-06	[4, 5, 6, 7, 8]	Group178	4,66	26,00
GO:0009306	protein secretion	GO_BiologicalProcess-EBI-UniProt-GOA-ACAP-ARAP_08.05.2020_00h00	0,017033646	0,030412685	9,40576E-07	3,9863E-06	[4, 5, 6, 7, 8]	Group178	4,05	27,00
GO:0030098	lymphocyte differentiation	GO_BiologicalProcess-EBI-UniProt-GOA-ACAP-ARAP_08.05.2020_00h00	0,005579029	0,013022271	9,40576E-07	3,9863E-06	[4, 5, 6, 7, 8, 9]	Group178	5,00	19,00
GO:0043312	neutrophil degranulation	GO_BiologicalProcess-EBI-UniProt-GOA-ACAP-ARAP_08.05.2020_00h00	0,000478491	0,00201725	9,40576E-07	3,9863E-06	[4, 5, 6, 7, 8, 9]	Group178	5,19	28,00
GO:0097190	apoptotic signaling pathway	GO_BiologicalProcess-EBI-UniProt-GOA-ACAP-ARAP_08.05.2020_00h00	1,38238E-06	1,63326E-05	9,79782E-07	4,0558E-06	[3, 4, 5]	Group145	6,00	39,00
GO:0072331	signal transduction by p53 class mediator	GO_BiologicalProcess-EBI-UniProt-GOA-ACAP-ARAP_08.05.2020_00h00	0,010597887	0,021351954	9,79782E-07	4,0558E-06	[4, 5, 6]	Group145	5,12	15,00
GO:0097193	intrinsic apoptotic signaling pathway	GO_BiologicalProcess-EBI-UniProt-GOA-ACAP-ARAP_08.05.2020_00h00	0,000166499	0,000895165	9,79782E-07	4,0558E-06	[4, 5, 6]	Group145	6,56	20,00
GO:1905897	regulation of response to endoplasmic reticulum stress	GO_BiologicalProcess-EBI-UniProt-GOA-ACAP-ARAP_08.05.2020_00h00	0,009845688	0,020263061	9,79782E-07	4,0558E-06	[4, 5, 6]	Group145	7,69	7,00
GO:2001233	regulation of apoptotic signaling pathway	GO_BiologicalProcess-EBI-UniProt-GOA-ACAP-ARAP_08.05.2020_00h00	0,003071183	0,008186971	9,79782E-07	4,0558E-06	[4, 5, 6, 7]	Group145	4,99	22,00
GO:0008630	intrinsic apoptotic signaling pathway in response to DNA damage	GO_BiologicalProcess-EBI-UniProt-GOA-ACAP-ARAP_08.05.2020_00h00	0,002549824	0,007219472	9,79782E-07	4,0558E-06	[5, 6, 7]	Group145	8,11	9,00
GO:2001234	negative regulation of apoptotic signaling pathway	GO_BiologicalProcess-EBI-UniProt-GOA-ACAP-ARAP_08.05.2020_00h00	0,001539791	0,004978311	9,79782E-07	4,0558E-06	[4, 5, 6, 7, 8]	Group145	6,11	16,00
GO:2001242	regulation of intrinsic apoptotic signaling pathway	GO_BiologicalProcess-EBI-UniProt-GOA-ACAP-ARAP_08.05.2020_00h00	0,01703891	0,03036543	9,79782E-07	4,0558E-06	[5, 6, 7, 8]	Group145	5,71	10,00
GO:2001243	negative regulation of intrinsic apoptotic signaling pathway	GO_BiologicalProcess-EBI-UniProt-GOA-ACAP-ARAP_08.05.2020_00h00	0,00920515	0,019361163	9,79782E-07	4,0558E-06	[5, 6, 7, 8, 9]	Group145	7,14	8,00
GO:0070482	response to oxygen levels	GO_BiologicalProcess-EBI-UniProt-GOA-ACAP-ARAP_08.05.2020_00h00	1,73753E-06	2,00339E-05	1,0316E-06	4,1733E-06	[3]	Group099	6,99	29,00
GO:0001666	response to hypoxia	GO_BiologicalProcess-EBI-UniProt-GOA-ACAP-ARAP_08.05.2020_00h00	2,00466E-05	0,000163971	1,0316E-06	4,1733E-06	[3, 5]	Group099	6,67	25,00
GO:0036294	cellular response to decreased oxygen levels	GO_BiologicalProcess-EBI-UniProt-GOA-ACAP-ARAP_08.05.2020_00h00	6,02248E-06	5,88114E-05	1,0316E-06	4,1733E-06	[5]	Group099	8,60	19,00
GO:0071456	cellular response to hypoxia	GO_BiologicalProcess-EBI-UniProt-GOA-ACAP-ARAP_08.05.2020_00h00	4,2432E-05	0,000307632	1,0316E-06	4,1733E-06	[4, 6]	Group099	8,06	17,00
GO:0043618	regulation of transcription from RNA polymerase II promoter in response to stress	GO_BiologicalProcess-EBI-UniProt-GOA-ACAP-ARAP_08.05.2020_00h00	0,016302135	0,029436117	1,0316E-06	4,1733E-06	[5, 7, 8, 9, 10, 11, 12]	Group099	6,45	8,00
GO:0001894	tissue homeostasis	GO_BiologicalProcess-EBI-UniProt-GOA-ACAP-ARAP_08.05.2020_00h00	0,023241255	0,038480762	1,73712E-06	6,8713E-06	[3, 5]	Group159	4,76	14,00
GO:0043114	regulation of vascular permeability	GO_BiologicalProcess-EBI-UniProt-GOA-ACAP-ARAP_08.05.2020_00h00	0,008146646	0,017638776	1,73712E-06	6,8713E-06	[3, 5]	Group159	10,42	5,00
GO:0060249	anatomical structure homeostasis	GO_BiologicalProcess-EBI-UniProt-GOA-ACAP-ARAP_08.05.2020_00h00	0,020128882	0,03470872	1,73712E-06	6,8713E-06	[4]	Group159	4,21	22,00
GO:0002064	epithelial cell development	GO_BiologicalProcess-EBI-UniProt-GOA-ACAP-ARAP_08.05.2020_00h00	3,30027E-06	3,50928E-05	1,73712E-06	6,8713E-06	[4, 5, 6]	Group159	8,62	20,00
GO:0003158	endothelium development	GO_BiologicalProcess-EBI-UniProt-GOA-ACAP-ARAP_08.05.2020_00h00	0,000445457	0,001920282	1,73712E-06	6,8713E-06	[5]	Group159	8,80	11,00

GO:0035329	hippo signaling	GO_BiologicalProcess-EBI-UniProt-GOA-ACAP-ARAP_08.05.2020_00h00	0,000833247	0,003055238	1,73712E-06	6,8713E-06	[4, 5, 6]	Group159	13,95	6,00
GO:0035633	maintenance of blood-brain barrier	GO_BiologicalProcess-EBI-UniProt-GOA-ACAP-ARAP_08.05.2020_00h00	0,000421301	0,001849472	1,73712E-06	6,8713E-06	[4, 6]	Group159	15,79	6,00
GO:0043116	negative regulation of vascular permeability	GO_BiologicalProcess-EBI-UniProt-GOA-ACAP-ARAP_08.05.2020_00h00	0,009205425	0,019319279	1,73712E-06	6,8713E-06	[4, 6]	Group159	17,65	3,00
GO:0043117	positive regulation of vascular permeability	GO_BiologicalProcess-EBI-UniProt-GOA-ACAP-ARAP_08.05.2020_00h00	0,007729088	0,016887529	1,73712E-06	6,8713E-06	[4, 6]	Group159	18,75	3,00
GO:0001885	endothelial cell development	GO_BiologicalProcess-EBI-UniProt-GOA-ACAP-ARAP_08.05.2020_00h00	0,000314285	0,001481627	1,73712E-06	6,8713E-06	[5, 6, 7]	Group159	12,12	8,00
GO:0071320	cellular response to cAMP	GO_BiologicalProcess-EBI-UniProt-GOA-ACAP-ARAP_08.05.2020_00h00	0,003981134	0,009921734	1,73712E-06	6,8713E-06	[5, 6, 7]	Group159	10,34	6,00
GO:0051726	regulation of cell cycle	GO_BiologicalProcess-EBI-UniProt-GOA-ACAP-ARAP_08.05.2020_00h00	0,000162478	0,000883473	2,1903E-06	8,4755E-06	[3, 4]	Group183	4,30	56,00
GO:0006974	cellular response to DNA damage stimulus	GO_BiologicalProcess-EBI-UniProt-GOA-ACAP-ARAP_08.05.2020_00h00	0,001050609	0,003642874	2,1903E-06	8,4755E-06	[4]	Group183	4,38	40,00
GO:0045786	negative regulation of cell cycle	GO_BiologicalProcess-EBI-UniProt-GOA-ACAP-ARAP_08.05.2020_00h00	0,000479548	0,002012839	2,1903E-06	8,4755E-06	[3, 4, 5]	Group183	4,93	32,00
GO:0045787	positive regulation of cell cycle	GO_BiologicalProcess-EBI-UniProt-GOA-ACAP-ARAP_08.05.2020_00h00	0,018188933	0,032056738	2,1903E-06	8,4755E-06	[3, 4, 5]	Group183	4,51	18,00
GO:0000075	cell cycle checkpoint	GO_BiologicalProcess-EBI-UniProt-GOA-ACAP-ARAP_08.05.2020_00h00	0,003692508	0,00942328	2,1903E-06	8,4755E-06	[2, 4, 5, 6]	Group183	5,96	14,00
GO:0007050	cell cycle arrest	GO_BiologicalProcess-EBI-UniProt-GOA-ACAP-ARAP_08.05.2020_00h00	1,01457E-05	9,07426E-05	2,1903E-06	8,4755E-06	[3, 4, 5, 6]	Group183	8,00	20,00
GO:0007346	regulation of mitotic cell cycle	GO_BiologicalProcess-EBI-UniProt-GOA-ACAP-ARAP_08.05.2020_00h00	0,003878796	0,009742802	2,1903E-06	8,4755E-06	[4, 5]	Group183	4,37	31,00
GO:0090068	positive regulation of cell cycle process	GO_BiologicalProcess-EBI-UniProt-GOA-ACAP-ARAP_08.05.2020_00h00	0,028356241	0,044633094	2,1903E-06	8,4755E-06	[3, 4, 5, 6]	Group183	4,64	14,00
GO:0007093	mitotic cell cycle checkpoint	GO_BiologicalProcess-EBI-UniProt-GOA-ACAP-ARAP_08.05.2020_00h00	0,002140553	0,00640159	2,1903E-06	8,4755E-06	[3, 4, 5, 6, 7]	Group183	6,90	12,00
GO:0042770	signal transduction in response to DNA damage	GO_BiologicalProcess-EBI-UniProt-GOA-ACAP-ARAP_08.05.2020_00h00	0,001526183	0,004951043	2,1903E-06	8,4755E-06	[4, 5, 6]	Group183	7,59	11,00
GO:0045930	negative regulation of mitotic cell cycle	GO_BiologicalProcess-EBI-UniProt-GOA-ACAP-ARAP_08.05.2020_00h00	0,000464017	0,001973621	2,1903E-06	8,4755E-06	[4, 5, 6]	Group183	6,06	20,00
GO:0072331	signal transduction by p53 class mediator	GO_BiologicalProcess-EBI-UniProt-GOA-ACAP-ARAP_08.05.2020_00h00	0,010597887	0,021351954	2,1903E-06	8,4755E-06	[4, 5, 6]	Group183	5,12	15,00
GO:0097193	intrinsic apoptotic signaling pathway	GO_BiologicalProcess-EBI-UniProt-GOA-ACAP-ARAP_08.05.2020_00h00	0,000166499	0,000895165	2,1903E-06	8,4755E-06	[4, 5, 6]	Group183	6,56	20,00
GO:1901988	negative regulation of cell cycle phase transition	GO_BiologicalProcess-EBI-UniProt-GOA-ACAP-ARAP_08.05.2020_00h00	0,001211727	0,004097606	2,1903E-06	8,4755E-06	[4, 5, 6, 7]	Group183	6,25	16,00
GO:0000077	DNA damage checkpoint	GO_BiologicalProcess-EBI-UniProt-GOA-ACAP-ARAP_08.05.2020_00h00	0,003633375	0,00934715	2,1903E-06	8,4755E-06	[4, 5, 6, 7, 8]	Group183	6,79	11,00
GO:0008630	intrinsic apoptotic signaling pathway in response to DNA damage	GO_BiologicalProcess-EBI-UniProt-GOA-ACAP-ARAP_08.05.2020_00h00	0,002549824	0,007219472	2,1903E-06	8,4755E-06	[5, 6, 7]	Group183	8,11	9,00
GO:0071158	positive regulation of cell cycle arrest	GO_BiologicalProcess-EBI-UniProt-GOA-ACAP-ARAP_08.05.2020_00h00	0,002135142	0,006405427	2,1903E-06	8,4755E-06	[4, 5, 6, 7, 8]	Group183	9,09	8,00

GO:0031572	G2 DNA damage checkpoint	GO_BiologicalProcess-EBI-UniProt-GOA-ACAP-ARAP_08.05.2020_00h00	0,015443421	0,028044315	2,1903E-06	8,4755E-06	[5, 6, 7, 8, 9]	Group183	10,81	4,00
GO:0044773	mitotic DNA damage checkpoint	GO_BiologicalProcess-EBI-UniProt-GOA-ACAP-ARAP_08.05.2020_00h00	0,001520331	0,004948833	2,1903E-06	8,4755E-06	[5, 6, 7, 8, 9]	Group183	8,74	9,00
GO:0072422	signal transduction involved in DNA damage checkpoint	GO_BiologicalProcess-EBI-UniProt-GOA-ACAP-ARAP_08.05.2020_00h00	0,003707789	0,009437113	2,1903E-06	8,4755E-06	[5, 6, 7, 8, 9]	Group183	9,21	7,00
GO:0004693	cyclin-dependent protein serine/threonine kinase activity	GO_BiologicalProcess-EBI-UniProt-GOA-ACAP-ARAP_08.05.2020_00h00	0,000545112	0,002248586	2,1903E-06	8,4755E-06	[5, 6, 9, 10]	Group183	8,59	11,00
GO:2000134	negative regulation of G1/S transition of mitotic cell cycle	GO_BiologicalProcess-EBI-UniProt-GOA-ACAP-ARAP_08.05.2020_00h00	0,022311579	0,037394363	2,1903E-06	8,4755E-06	[6, 7, 8, 9]	Group183	6,54	7,00
GO:0006977	DNA damage response, signal transduction by p53 class mediator resulting in cell cycle arrest	GO_BiologicalProcess-EBI-UniProt-GOA-ACAP-ARAP_08.05.2020_00h00	0,003648136	0,009359963	2,1903E-06	8,4755E-06	[5, 6, 7, 8, 9, 10, 11, 12, 13]	Group183	10,53	6,00
GO:0001934	positive regulation of protein phosphorylation	GO_BiologicalProcess-EBI-UniProt-GOA-ACAP-ARAP_08.05.2020_00h00	4,44036E-07	6,07061E-06	2,47322E-06	9,1715E-06	[6, 7, 8, 9]	Group112	5,24	57,00
GO:0018209	peptidyl-serine modification	GO_BiologicalProcess-EBI-UniProt-GOA-ACAP-ARAP_08.05.2020_00h00	0,000275328	0,001337508	2,47322E-06	9,1715E-06	[7, 8]	Group112	6,01	22,00
GO:0018105	peptidyl-serine phosphorylation	GO_BiologicalProcess-EBI-UniProt-GOA-ACAP-ARAP_08.05.2020_00h00	0,000650843	0,00255269	2,47322E-06	9,1715E-06	[7, 8, 9]	Group112	5,90	20,00
GO:0033135	regulation of peptidyl-serine phosphorylation	GO_BiologicalProcess-EBI-UniProt-GOA-ACAP-ARAP_08.05.2020_00h00	0,011230711	0,022437977	2,47322E-06	9,1715E-06	[7, 8, 9, 10]	Group112	6,10	10,00
GO:0032147	activation of protein kinase activity	GO_BiologicalProcess-EBI-UniProt-GOA-ACAP-ARAP_08.05.2020_00h00	2,21901E-05	0,000176966	2,47322E-06	9,1715E-06	[8, 9, 10, 11]	Group112	6,48	26,00
GO:0070848	response to growth factor	GO_BiologicalProcess-EBI-UniProt-GOA-ACAP-ARAP_08.05.2020_00h00	1,15067E-05	0,000101962	2,4491E-06	9,2753E-06	[4]	Group079	5,37	41,00
GO:1901652	response to peptide	GO_BiologicalProcess-EBI-UniProt-GOA-ACAP-ARAP_08.05.2020_00h00	0,00784214	0,01709551	2,4491E-06	9,2753E-06	[4, 5]	Group079	4,42	25,00
GO:0007167	enzyme linked receptor protein signaling pathway	GO_BiologicalProcess-EBI-UniProt-GOA-ACAP-ARAP_08.05.2020_00h00	3,81246E-06	3,96579E-05	2,4491E-06	9,2753E-06	[4, 5, 6]	Group079	4,96	55,00
GO:0007169	transmembrane receptor protein tyrosine kinase signaling pathway	GO_BiologicalProcess-EBI-UniProt-GOA-ACAP-ARAP_08.05.2020_00h00	0,00012082	0,000718164	2,4491E-06	9,2753E-06	[5, 6, 7]	Group079	4,97	38,00
GO:0010810	regulation of cell-substrate adhesion	GO_BiologicalProcess-EBI-UniProt-GOA-ACAP-ARAP_08.05.2020_00h00	3,73914E-05	0,000273157	2,73209E-06	9,9247E-06	[4, 5]	Group109	7,83	18,00
GO:0010812	negative regulation of cell-substrate adhesion	GO_BiologicalProcess-EBI-UniProt-GOA-ACAP-ARAP_08.05.2020_00h00	0,032554209	0,04921703	2,73209E-06	9,9247E-06	[4, 5, 6]	Group109	7,35	5,00
GO:0001952	regulation of cell-matrix adhesion	GO_BiologicalProcess-EBI-UniProt-GOA-ACAP-ARAP_08.05.2020_00h00	4,35039E-05	0,000308394	2,73209E-06	9,9247E-06	[5, 6]	Group109	9,85	13,00
GO:0048041	focal adhesion assembly	GO_BiologicalProcess-EBI-UniProt-GOA-ACAP-ARAP_08.05.2020_00h00	0,00302181	0,00814612	2,73209E-06	9,9247E-06	[5, 7]	Group109	8,60	8,00
GO:0032232	negative regulation of actin filament bundle assembly	GO_BiologicalProcess-EBI-UniProt-GOA-ACAP-ARAP_08.05.2020_00h00	0,010381774	0,021094177	2,73209E-06	9,9247E-06	[5, 6, 7, 8, 9, 10, 11]	Group109	12,12	4,00
GO:0009057	macromolecule catabolic process	GO_BiologicalProcess-EBI-UniProt-GOA-ACAP-ARAP_08.05.2020_00h00	0,000587955	0,002364171	3,0958E-06	1,0805E-05	[4]	Group168	4,01	60,00
GO:0006508	proteolysis	GO_BiologicalProcess-EBI-UniProt-GOA-ACAP-ARAP_08.05.2020_00h00	5,7795E-05	0,00039507	3,0958E-06	1,0805E-05	[4, 5]	Group168	4,07	77,00
GO:0030163	protein catabolic process	GO_BiologicalProcess-EBI-UniProt-GOA-ACAP-ARAP_08.05.2020_00h00	0,003023085	0,008126664	3,0958E-06	1,0805E-05	[4, 5]	Group168	4,10	41,00

GO:0044265	cellular macromolecule catabolic process	GO_BiologicalProcess-EBI-UniProt-GOA-ACAP-ARAP_08.05.2020_00h00	0,000537689	0,002237255	3,0958E-06	1,0805E-05	[4, 5]		Group168	4,19	52,00
GO:0008233	peptidase activity	GO_BiologicalProcess-EBI-UniProt-GOA-ACAP-ARAP_08.05.2020_00h00	0,0002754	0,001331101	3,0958E-06	1,0805E-05	[5, 6]		Group168	4,39	49,00
GO:0030162	regulation of proteolysis	GO_BiologicalProcess-EBI-UniProt-GOA-ACAP-ARAP_08.05.2020_00h00	0,004999209	0,011901102	3,0958E-06	1,0805E-05	[5, 6]		Group168	4,21	33,00
GO:0044257	cellular protein catabolic process	GO_BiologicalProcess-EBI-UniProt-GOA-ACAP-ARAP_08.05.2020_00h00	0,003651367	0,009343204	3,0958E-06	1,0805E-05	[5, 6]		Group168	4,23	35,00
GO:0051603	proteolysis involved in cellular protein catabolic process	GO_BiologicalProcess-EBI-UniProt-GOA-ACAP-ARAP_08.05.2020_00h00	0,003256541	0,00856184	3,0958E-06	1,0805E-05	[5, 6, 7]		Group168	4,34	33,00
GO:0010498	proteasomal protein catabolic process	GO_BiologicalProcess-EBI-UniProt-GOA-ACAP-ARAP_08.05.2020_00h00	0,006044001	0,013870765	3,0958E-06	1,0805E-05	[5, 6, 7, 8]		Group168	4,64	23,00
GO:0070011	peptidase activity, acting on L-amino acid peptides	GO_BiologicalProcess-EBI-UniProt-GOA-ACAP-ARAP_08.05.2020_00h00	0,00039584	0,00177018	3,0958E-06	1,0805E-05	[6, 7]		Group168	4,37	47,00
GO:0004175	endopeptidase activity	GO_BiologicalProcess-EBI-UniProt-GOA-ACAP-ARAP_08.05.2020_00h00	0,000555813	0,002282888	3,0958E-06	1,0805E-05	[7, 8]		Group168	4,52	40,00
GO:0008234	cysteine-type peptidase activity	GO_BiologicalProcess-EBI-UniProt-GOA-ACAP-ARAP_08.05.2020_00h00	0,000334646	0,001554642	3,0958E-06	1,0805E-05	[7, 8]		Group168	5,49	26,00
GO:0052548	regulation of endopeptidase activity	GO_BiologicalProcess-EBI-UniProt-GOA-ACAP-ARAP_08.05.2020_00h00	0,000937128	0,003333945	3,0958E-06	1,0805E-05	[6, 7, 8, 9]		Group168	5,29	24,00
GO:0009894	regulation of catabolic process	GO_BiologicalProcess-EBI-UniProt-GOA-ACAP-ARAP_08.05.2020_00h00	0,002341402	0,006810703	3,09319E-06	1,1012E-05	[3, 4]		Group186	4,08	44,00
GO:0009057	macromolecule catabolic process	GO_BiologicalProcess-EBI-UniProt-GOA-ACAP-ARAP_08.05.2020_00h00	0,000587955	0,002364171	3,09319E-06	1,1012E-05	[4]		Group186	4,01	60,00
GO:0019439	aromatic compound catabolic process	GO_BiologicalProcess-EBI-UniProt-GOA-ACAP-ARAP_08.05.2020_00h00	0,012572658	0,024455354	3,09319E-06	1,1012E-05	[4]		Group186	4,15	27,00
GO:0044270	cellular nitrogen compound catabolic process	GO_BiologicalProcess-EBI-UniProt-GOA-ACAP-ARAP_08.05.2020_00h00	0,009469164	0,019699978	3,09319E-06	1,1012E-05	[4]		Group186	4,25	27,00
GO:0046700	heterocycle catabolic process	GO_BiologicalProcess-EBI-UniProt-GOA-ACAP-ARAP_08.05.2020_00h00	0,008757261	0,018623775	3,09319E-06	1,1012E-05	[4]		Group186	4,28	27,00
GO:1901361	organic cyclic compound catabolic process	GO_BiologicalProcess-EBI-UniProt-GOA-ACAP-ARAP_08.05.2020_00h00	0,013598622	0,025872527	3,09319E-06	1,1012E-05	[4]		Group186	4,09	28,00
GO:0006508	proteolysis	GO_BiologicalProcess-EBI-UniProt-GOA-ACAP-ARAP_08.05.2020_00h00	5,7795E-05	0,00039507	3,09319E-06	1,1012E-05	[4, 5]		Group186	4,07	77,00
GO:0006518	peptide metabolic process	GO_BiologicalProcess-EBI-UniProt-GOA-ACAP-ARAP_08.05.2020_00h00	0,002876424	0,007864966	3,09319E-06	1,1012E-05	[4, 5]		Group186	4,23	37,00
GO:0030163	protein catabolic process	GO_BiologicalProcess-EBI-UniProt-GOA-ACAP-ARAP_08.05.2020_00h00	0,003023085	0,008126664	3,09319E-06	1,1012E-05	[4, 5]		Group186	4,10	41,00
GO:0031329	regulation of cellular catabolic process	GO_BiologicalProcess-EBI-UniProt-GOA-ACAP-ARAP_08.05.2020_00h00	0,00275227	0,007656751	3,09319E-06	1,1012E-05	[4, 5]		Group186	4,21	38,00
GO:0034248	regulation of cellular amide metabolic process	GO_BiologicalProcess-EBI-UniProt-GOA-ACAP-ARAP_08.05.2020_00h00	0,000700788	0,00270425	3,09319E-06	1,1012E-05	[4, 5]		Group186	5,22	26,00
GO:0034655	nucleobase-containing compound catabolic process	GO_BiologicalProcess-EBI-UniProt-GOA-ACAP-ARAP_08.05.2020_00h00	0,009850574	0,020229612	3,09319E-06	1,1012E-05	[4, 5]		Group186	4,33	25,00
GO:0044265	cellular macromolecule catabolic process	GO_BiologicalProcess-EBI-UniProt-GOA-ACAP-ARAP_08.05.2020_00h00	0,000537689	0,002237255	3,09319E-06	1,1012E-05	[4, 5]		Group186	4,19	52,00

GO:0034250	positive regulation of cellular amide metabolic process	GO_BiologicalProcess-EBI-UniProt-GOA-ACAP-ARAP_08.05.2020_00h00	0,002245195	0,006611236	3,09319E-06	1,1012E-05	[4, 5, 6]	Group186	6,86	12,00
GO:0043604	amide biosynthetic process	GO_BiologicalProcess-EBI-UniProt-GOA-ACAP-ARAP_08.05.2020_00h00	0,00857806	0,018283304	3,09319E-06	1,1012E-05	[5]	Group186	4,00	35,00
GO:0010608	posttranscriptional regulation of gene expression	GO_BiologicalProcess-EBI-UniProt-GOA-ACAP-ARAP_08.05.2020_00h00	0,000370525	0,001688533	3,09319E-06	1,1012E-05	[5, 6]	Group186	4,89	34,00
GO:0043043	peptide biosynthetic process	GO_BiologicalProcess-EBI-UniProt-GOA-ACAP-ARAP_08.05.2020_00h00	0,013397908	0,025694987	3,09319E-06	1,1012E-05	[5, 6]	Group186	4,06	29,00
GO:0044257	cellular protein catabolic process	GO_BiologicalProcess-EBI-UniProt-GOA-ACAP-ARAP_08.05.2020_00h00	0,003651367	0,009343204	3,09319E-06	1,1012E-05	[5, 6]	Group186	4,23	35,00
GO:0043487	regulation of RNA stability	GO_BiologicalProcess-EBI-UniProt-GOA-ACAP-ARAP_08.05.2020_00h00	0,014361648	0,026584327	3,09319E-06	1,1012E-05	[3, 5, 6, 7, 8]	Group186	5,61	11,00
GO:0006401	RNA catabolic process	GO_BiologicalProcess-EBI-UniProt-GOA-ACAP-ARAP_08.05.2020_00h00	0,009984245	0,020460218	3,09319E-06	1,1012E-05	[5, 6, 7]	Group186	4,64	20,00
GO:0006413	translational initiation	GO_BiologicalProcess-EBI-UniProt-GOA-ACAP-ARAP_08.05.2020_00h00	0,002980064	0,008079099	3,09319E-06	1,1012E-05	[3, 6, 7, 8]	Group186	6,34	13,00
GO:0051603	proteolysis involved in cellular protein catabolic process	GO_BiologicalProcess-EBI-UniProt-GOA-ACAP-ARAP_08.05.2020_00h00	0,003256541	0,00856184	3,09319E-06	1,1012E-05	[5, 6, 7]	Group186	4,34	33,00
GO:0006402	mRNA catabolic process	GO_BiologicalProcess-EBI-UniProt-GOA-ACAP-ARAP_08.05.2020_00h00	0,014094888	0,026242817	3,09319E-06	1,1012E-05	[5, 6, 7, 8]	Group186	4,64	18,00
GO:0006417	regulation of translation	GO_BiologicalProcess-EBI-UniProt-GOA-ACAP-ARAP_08.05.2020_00h00	0,000423629	0,001851203	3,09319E-06	1,1012E-05	[5, 6, 7, 8]	Group186	5,59	24,00
GO:0010498	proteasomal protein catabolic process	GO_BiologicalProcess-EBI-UniProt-GOA-ACAP-ARAP_08.05.2020_00h00	0,006044001	0,013870765	3,09319E-06	1,1012E-05	[5, 6, 7, 8]	Group186	4,64	23,00
GO:0006446	regulation of translational initiation	GO_BiologicalProcess-EBI-UniProt-GOA-ACAP-ARAP_08.05.2020_00h00	0,019380657	0,033783767	3,09319E-06	1,1012E-05	[4, 6, 7, 8, 9]	Group186	7,41	6,00
GO:0017148	negative regulation of translation	GO_BiologicalProcess-EBI-UniProt-GOA-ACAP-ARAP_08.05.2020_00h00	0,012469945	0,024354564	3,09319E-06	1,1012E-05	[5, 6, 7, 8, 9]	Group186	6,34	9,00
GO:0045727	positive regulation of translation	GO_BiologicalProcess-EBI-UniProt-GOA-ACAP-ARAP_08.05.2020_00h00	0,011439243	0,022712355	3,09319E-06	1,1012E-05	[5, 6, 7, 8, 9]	Group186	6,43	9,00
GO:0000956	nuclear-transcribed mRNA catabolic process	GO_BiologicalProcess-EBI-UniProt-GOA-ACAP-ARAP_08.05.2020_00h00	0,029677309	0,045956612	3,09319E-06	1,1012E-05	[6, 7, 8, 9]	Group186	5,02	11,00
GO:0000288	nuclear-transcribed mRNA catabolic process, deadenylation-dependent decay	GO_BiologicalProcess-EBI-UniProt-GOA-ACAP-ARAP_08.05.2020_00h00	0,0039889	0,009915266	3,09319E-06	1,1012E-05	[7, 8, 9, 10]	Group186	9,09	7,00
GO:0043928	exonucleolytic catabolism of deadenylated mRNA	GO_BiologicalProcess-EBI-UniProt-GOA-ACAP-ARAP_08.05.2020_00h00	0,010381774	0,021094177	3,09319E-06	1,1012E-05	[8, 9, 10, 11]	Group186	12,12	4,00
GO:0007044	cell-substrate junction assembly	GO_BiologicalProcess-EBI-UniProt-GOA-ACAP-ARAP_08.05.2020_00h00	6,03086E-06	5,82983E-05	3,77865E-06	1,2935E-05	[6]	Group057	11,82	13,00
GO:0070831	basement membrane assembly	GO_BiologicalProcess-EBI-UniProt-GOA-ACAP-ARAP_08.05.2020_00h00	0,009205425	0,019319279	3,77865E-06	1,2935E-05	[6, 7]	Group057	17,65	3,00
GO:0031581	hemidesmosome assembly	GO_BiologicalProcess-EBI-UniProt-GOA-ACAP-ARAP_08.05.2020_00h00	1,29834E-05	0,000111938	3,77865E-06	1,2935E-05	[7]	Group057	38,46	5,00
GO:0036294	cellular response to decreased oxygen levels	GO_BiologicalProcess-EBI-UniProt-GOA-ACAP-ARAP_08.05.2020_00h00	6,02248E-06	5,88114E-05	4,39587E-06	1,4763E-05	[5]	Group060	8,60	19,00

GO:0043618	regulation of transcription from RNA polymerase II promoter in response to stress	GO_BiologicalProcess-EBI-UniProt-GOA-ACAP-ARAP_08.05.2020_00h00	0,016302135	0,029436117	4,39587E-06	1,4763E-05	[5, 7, 8, 9, 10, 11, 12]	Group060	6,45	8,00
GO:0036003	positive regulation of transcription from RNA polymerase II promoter in response to stress	GO_BiologicalProcess-EBI-UniProt-GOA-ACAP-ARAP_08.05.2020_00h00	0,008316356	0,017884837	4,39587E-06	1,4763E-05	[6, 7, 8, 9, 10, 11, 12, 13]	Group060	12,90	4,00
GO:0060429	epithelium development	GO_BiologicalProcess-EBI-UniProt-GOA-ACAP-ARAP_08.05.2020_00h00	5,08926E-06	5,18131E-05	5,08926E-06	1,6776E-05	[4]	Group102	4,64	64,00
GO:0030855	epithelial cell differentiation	GO_BiologicalProcess-EBI-UniProt-GOA-ACAP-ARAP_08.05.2020_00h00	0,00043345	0,001885508	5,08926E-06	1,6776E-05	[4, 5]	Group102	4,66	38,00
GO:0002064	epithelial cell development	GO_BiologicalProcess-EBI-UniProt-GOA-ACAP-ARAP_08.05.2020_00h00	3,30027E-06	3,50928E-05	5,08926E-06	1,6776E-05	[4, 5, 6]	Group102	8,62	20,00
GO:0003158	endothelium development	GO_BiologicalProcess-EBI-UniProt-GOA-ACAP-ARAP_08.05.2020_00h00	0,000445457	0,001920282	5,08926E-06	1,6776E-05	[5]	Group102	8,80	11,00
GO:0001885	endothelial cell development	GO_BiologicalProcess-EBI-UniProt-GOA-ACAP-ARAP_08.05.2020_00h00	0,000314285	0,001481627	5,08926E-06	1,6776E-05	[5, 6, 7]	Group102	12,12	8,00
GO:0098751	bone cell development	GO_BiologicalProcess-EBI-UniProt-GOA-ACAP-ARAP_08.05.2020_00h00	0,014057252	0,026326399	6,2456E-06	2,0213E-05	[4, 5, 6, 7]	Group084	11,11	4,00
GO:0030099	myeloid cell differentiation	GO_BiologicalProcess-EBI-UniProt-GOA-ACAP-ARAP_08.05.2020_00h00	6,2456E-06	5,97704E-05	6,2456E-06	2,0213E-05	[4, 5, 6, 7, 8]	Group084	6,55	29,00
GO:0061515	myeloid cell development	GO_BiologicalProcess-EBI-UniProt-GOA-ACAP-ARAP_08.05.2020_00h00	0,000976749	0,003436575	6,2456E-06	2,0213E-05	[4, 5, 6, 7, 8, 9]	Group084	10,26	8,00
GO:0043249	erythrocyte maturation	GO_BiologicalProcess-EBI-UniProt-GOA-ACAP-ARAP_08.05.2020_00h00	0,012620698	0,024499003	6,2456E-06	2,0213E-05	[4, 5, 6, 7, 8, 9, 10, 11]	Group084	15,79	3,00
GO:0044089	positive regulation of cellular component biogenesis	GO_BiologicalProcess-EBI-UniProt-GOA-ACAP-ARAP_08.05.2020_00h00	0,006975319	0,01552414	1,18322E-05	3,761E-05	[3, 4, 5]	Group130	4,41	26,00
GO:0034329	cell junction assembly	GO_BiologicalProcess-EBI-UniProt-GOA-ACAP-ARAP_08.05.2020_00h00	0,000284442	0,001361054	1,18322E-05	3,761E-05	[5]	Group130	5,54	26,00
GO:1901888	regulation of cell junction assembly	GO_BiologicalProcess-EBI-UniProt-GOA-ACAP-ARAP_08.05.2020_00h00	0,029677309	0,045956612	1,18322E-05	3,761E-05	[4, 5, 6]	Group130	5,02	11,00
GO:1901890	positive regulation of cell junction assembly	GO_BiologicalProcess-EBI-UniProt-GOA-ACAP-ARAP_08.05.2020_00h00	0,023341313	0,038579684	1,18322E-05	3,761E-05	[4, 5, 6, 7]	Group130	6,48	7,00
GO:0001954	positive regulation of cell-matrix adhesion	GO_BiologicalProcess-EBI-UniProt-GOA-ACAP-ARAP_08.05.2020_00h00	0,000936067	0,003342597	1,18322E-05	3,761E-05	[5, 6, 7]	Group130	11,67	7,00
GO:0099068	postsynapse assembly	GO_BiologicalProcess-EBI-UniProt-GOA-ACAP-ARAP_08.05.2020_00h00	0,009311849	0,0194149	1,18322E-05	3,761E-05	[5, 6, 7]	Group130	12,50	4,00
GO:0001816	cytokine production	GO_BiologicalProcess-EBI-UniProt-GOA-ACAP-ARAP_08.05.2020_00h00	0,000179184	0,00095266	1,64661E-05	5,142E-05	[2, 5]	Group072	4,75	41,00
GO:0006954	inflammatory response	GO_BiologicalProcess-EBI-UniProt-GOA-ACAP-ARAP_08.05.2020_00h00	0,000333665	0,001557644	1,64661E-05	5,142E-05	[4]	Group072	4,73	38,00
GO:0050727	regulation of inflammatory response	GO_BiologicalProcess-EBI-UniProt-GOA-ACAP-ARAP_08.05.2020_00h00	0,005733951	0,013254566	1,64661E-05	5,142E-05	[4, 5, 6]	Group072	4,99	19,00
GO:0001816	cytokine production	GO_BiologicalProcess-EBI-UniProt-GOA-ACAP-ARAP_08.05.2020_00h00	0,000179184	0,00095266	1,64661E-05	5,142E-05	[2, 5]	Group144	4,75	41,00
GO:0006954	inflammatory response	GO_BiologicalProcess-EBI-UniProt-GOA-ACAP-ARAP_08.05.2020_00h00	0,000333665	0,001557644	1,64661E-05	5,142E-05	[4]	Group144	4,73	38,00

GO:0001817	regulation of cytokine production	GO_BiologicalProcess-EBI-UniProt-GOA-ACAP-ARAP_08.05.2020_00h00	0,001565843	0,005045494	1,64661E-05	5,142E-05	[3, 4, 5, 6]	Group144	4,46	35,00
GO:0032635	interleukin-6 production	GO_BiologicalProcess-EBI-UniProt-GOA-ACAP-ARAP_08.05.2020_00h00	0,001438689	0,004715156	1,64661E-05	5,142E-05	[3, 6]	Group144	7,23	12,00
GO:0001819	positive regulation of cytokine production	GO_BiologicalProcess-EBI-UniProt-GOA-ACAP-ARAP_08.05.2020_00h00	0,013490905	0,025718717	1,64661E-05	5,142E-05	[3, 4, 5, 6, 7]	Group144	4,37	22,00
GO:0032652	regulation of interleukin-1 production	GO_BiologicalProcess-EBI-UniProt-GOA-ACAP-ARAP_08.05.2020_00h00	0,023341313	0,038579684	1,64661E-05	5,142E-05	[4, 5, 6, 7]	Group144	6,48	7,00
GO:0032675	regulation of interleukin-6 production	GO_BiologicalProcess-EBI-UniProt-GOA-ACAP-ARAP_08.05.2020_00h00	0,000707176	0,002707069	1,64661E-05	5,142E-05	[4, 5, 6, 7]	Group144	7,84	12,00
GO:0032715	negative regulation of interleukin-6 production	GO_BiologicalProcess-EBI-UniProt-GOA-ACAP-ARAP_08.05.2020_00h00	0,007454556	0,016362408	1,64661E-05	5,142E-05	[4, 5, 6, 7, 8]	Group144	10,64	5,00
GO:0050663	cytokine secretion	GO_BiologicalProcess-EBI-UniProt-GOA-ACAP-ARAP_08.05.2020_00h00	0,028077139	0,044266593	1,64661E-05	5,142E-05	[3, 4, 5, 6, 7, 8, 9]	Group144	4,76	13,00
GO:0030218	erythrocyte differentiation	GO_BiologicalProcess-EBI-UniProt-GOA-ACAP-ARAP_08.05.2020_00h00	0,001763233	0,005550702	2,06316E-05	6,3318E-05	[4, 5, 6, 7, 8, 9]	Group059	7,94	10,00
GO:0061515	myeloid cell development	GO_BiologicalProcess-EBI-UniProt-GOA-ACAP-ARAP_08.05.2020_00h00	0,000976749	0,003436575	2,06316E-05	6,3318E-05	[4, 5, 6, 7, 8, 9]	Group059	10,26	8,00
GO:0043249	erythrocyte maturation	GO_BiologicalProcess-EBI-UniProt-GOA-ACAP-ARAP_08.05.2020_00h00	0,012620698	0,024499003	2,06316E-05	6,3318E-05	[4, 5, 6, 7, 8, 9, 10, 11]	Group059	15,79	3,00
GO:0043616	keratinocyte proliferation	GO_BiologicalProcess-EBI-UniProt-GOA-ACAP-ARAP_08.05.2020_00h00	0,000302994	0,001435473	2,58225E-05	7,7905E-05	[4]	Group117	14,00	7,00
GO:0030856	regulation of epithelial cell differentiation	GO_BiologicalProcess-EBI-UniProt-GOA-ACAP-ARAP_08.05.2020_00h00	0,02252461	0,037685405	2,58225E-05	7,7905E-05	[4, 5, 6]	Group117	5,73	9,00
GO:0010837	regulation of keratinocyte proliferation	GO_BiologicalProcess-EBI-UniProt-GOA-ACAP-ARAP_08.05.2020_00h00	0,000487032	0,002035325	2,58225E-05	7,7905E-05	[5, 6]	Group117	15,38	6,00
GO:0030858	positive regulation of epithelial cell differentiation	GO_BiologicalProcess-EBI-UniProt-GOA-ACAP-ARAP_08.05.2020_00h00	0,025888894	0,041569918	2,58225E-05	7,7905E-05	[4, 5, 6, 7]	Group117	7,81	5,00
GO:1904035	regulation of epithelial cell apoptotic process	GO_BiologicalProcess-EBI-UniProt-GOA-ACAP-ARAP_08.05.2020_00h00	9,95666E-05	0,000610803	2,58225E-05	7,7905E-05	[6, 7]	Group117	11,36	10,00
GO:0001570	vasculogenesis	GO_BiologicalProcess-EBI-UniProt-GOA-ACAP-ARAP_08.05.2020_00h00	0,002293606	0,006712479	2,58225E-05	7,7905E-05	[4, 5, 6, 7, 8, 9, 10]	Group117	8,99	8,00
GO:0002294	CD4-positive, alpha-beta T cell differentiation involved in immune response	GO_BiologicalProcess-EBI-UniProt-GOA-ACAP-ARAP_08.05.2020_00h00	0,02291313	0,038135417	2,68601E-05	7,8379E-05	[8, 9, 10, 11, 12, 13]	Group125	8,06	5,00
GO:0002263	cell activation involved in immune response	GO_BiologicalProcess-EBI-UniProt-GOA-ACAP-ARAP_08.05.2020_00h00	1,98211E-05	0,000163524	2,68601E-05	7,8379E-05	[3]	Group125	5,24	41,00
GO:0046649	lymphocyte activation	GO_BiologicalProcess-EBI-UniProt-GOA-ACAP-ARAP_08.05.2020_00h00	0,001648926	0,0052426	2,68601E-05	7,8379E-05	[3, 4]	Group125	4,41	36,00
GO:0002285	lymphocyte activation involved in immune response	GO_BiologicalProcess-EBI-UniProt-GOA-ACAP-ARAP_08.05.2020_00h00	0,004762104	0,01147943	2,68601E-05	7,8379E-05	[4, 5]	Group125	6,25	12,00
GO:0042110	T cell activation	GO_BiologicalProcess-EBI-UniProt-GOA-ACAP-ARAP_08.05.2020_00h00	0,002095667	0,006306772	2,68601E-05	7,8379E-05	[4, 5]	Group125	4,90	25,00
GO:0030098	lymphocyte differentiation	GO_BiologicalProcess-EBI-UniProt-GOA-ACAP-ARAP_08.05.2020_00h00	0,005579029	0,013022271	2,68601E-05	7,8379E-05	[4, 5, 6, 7, 8, 9]	Group125	5,00	19,00

GO:0002294	CD4-positive, alpha-beta T cell differentiation involved in immune response	GO_BiologicalProcess-EBI-UniProt-GOA-ACAP-ARAP_08.05.2020_00h00	0,02291313	0,038135417	2,66407E-05	7,9034E-05	[8, 9, 10, 11, 12, 13]	Group174	8,06	5,00
GO:0072539	T-helper 17 cell differentiation	GO_BiologicalProcess-EBI-UniProt-GOA-ACAP-ARAP_08.05.2020_00h00	0,029607117	0,045996774	2,66407E-05	7,9034E-05	[6, 10, 11, 12, 13, 14, 15]	Group174	11,54	3,00
GO:0002285	lymphocyte activation involved in immune response	GO_BiologicalProcess-EBI-UniProt-GOA-ACAP-ARAP_08.05.2020_00h00	0,004762104	0,01147943	2,66407E-05	7,9034E-05	[4, 5]	Group174	6,25	12,00
GO:0032616	interleukin-13 production	GO_BiologicalProcess-EBI-UniProt-GOA-ACAP-ARAP_08.05.2020_00h00	0,032678593	0,04924947	2,66407E-05	7,9034E-05	[3, 6]	Group174	11,11	3,00
GO:0032633	interleukin-4 production	GO_BiologicalProcess-EBI-UniProt-GOA-ACAP-ARAP_08.05.2020_00h00	0,002621352	0,007400101	2,66407E-05	7,9034E-05	[3, 6]	Group174	13,51	5,00
GO:0032634	interleukin-5 production	GO_BiologicalProcess-EBI-UniProt-GOA-ACAP-ARAP_08.05.2020_00h00	0,021358484	0,036113199	2,66407E-05	7,9034E-05	[3, 6]	Group174	13,04	3,00
GO:0001819	positive regulation of cytokine production	GO_BiologicalProcess-EBI-UniProt-GOA-ACAP-ARAP_08.05.2020_00h00	0,013490905	0,025718717	2,66407E-05	7,9034E-05	[3, 4, 5, 6, 7]	Group174	4,37	22,00
GO:0070528	protein kinase C signaling	GO_BiologicalProcess-EBI-UniProt-GOA-ACAP-ARAP_08.05.2020_00h00	0,002037594	0,006210119	2,66407E-05	7,9034E-05	[4, 5, 6]	Group174	14,29	5,00
GO:0032736	positive regulation of interleukin-13 production	GO_BiologicalProcess-EBI-UniProt-GOA-ACAP-ARAP_08.05.2020_00h00	0,006402782	0,014520053	2,66407E-05	7,9034E-05	[4, 5, 6, 7, 8]	Group174	20,00	3,00
GO:0032754	positive regulation of interleukin-5 production	GO_BiologicalProcess-EBI-UniProt-GOA-ACAP-ARAP_08.05.2020_00h00	0,004184007	0,010319832	2,66407E-05	7,9034E-05	[4, 5, 6, 7, 8]	Group174	23,08	3,00
GO:0050663	cytokine secretion	GO_BiologicalProcess-EBI-UniProt-GOA-ACAP-ARAP_08.05.2020_00h00	0,028077139	0,044266593	2,66407E-05	7,9034E-05	[3, 4, 5, 6, 7, 8, 9]	Group174	4,76	13,00
GO:0030098	lymphocyte differentiation	GO_BiologicalProcess-EBI-UniProt-GOA-ACAP-ARAP_08.05.2020_00h00	0,005579029	0,013022271	2,66407E-05	7,9034E-05	[4, 5, 6, 7, 8, 9]	Group174	5,00	19,00
GO:0035745	T-helper 2 cell cytokine production	GO_BiologicalProcess-EBI-UniProt-GOA-ACAP-ARAP_08.05.2020_00h00	0,007729088	0,016887529	2,66407E-05	7,9034E-05	[4, 6, 8, 9]	Group174	18,75	3,00
GO:0072602	interleukin-4 secretion	GO_BiologicalProcess-EBI-UniProt-GOA-ACAP-ARAP_08.05.2020_00h00	0,000575675	0,002344345	2,66407E-05	7,9034E-05	[4, 5, 6, 7, 8, 9, 10]	Group174	42,86	3,00
GO:0097028	dendritic cell differentiation	GO_BiologicalProcess-EBI-UniProt-GOA-ACAP-ARAP_08.05.2020_00h00	0,029624119	0,045948595	2,66407E-05	7,9034E-05	[5, 6, 7, 8, 9]	Group174	8,89	4,00
GO:0030513	positive regulation of BMP signaling pathway	GO_BiologicalProcess-EBI-UniProt-GOA-ACAP-ARAP_08.05.2020_00h00	0,016912684	0,030253155	2,66407E-05	7,9034E-05	[5, 6, 7, 8, 9, 10]	Group174	10,53	4,00
GO:0051091	positive regulation of DNA-binding transcription factor activity	GO_BiologicalProcess-EBI-UniProt-GOA-ACAP-ARAP_08.05.2020_00h00	0,018849541	0,03297808	2,66407E-05	7,9034E-05	[2, 3, 4, 7, 8, 9, 10, 11, 12, 13]	Group174	4,90	14,00
GO:0051092	positive regulation of NF-kappaB transcription factor activity	GO_BiologicalProcess-EBI-UniProt-GOA-ACAP-ARAP_08.05.2020_00h00	0,012154936	0,023885572	2,66407E-05	7,9034E-05	[3, 4, 5, 8, 9, 10, 11, 12, 13, 14]	Group174	6,02	10,00
GO:0090559	regulation of membrane permeability	GO_BiologicalProcess-EBI-UniProt-GOA-ACAP-ARAP_08.05.2020_00h00	0,030644687	0,046923146	2,84964E-05	8,1812E-05	[3]	Group131	6,67	6,00
GO:0001836	release of cytochrome c from mitochondria	GO_BiologicalProcess-EBI-UniProt-GOA-ACAP-ARAP_08.05.2020_00h00	0,001802429	0,005618646	2,84964E-05	8,1812E-05	[4, 5, 6, 7]	Group131	10,45	7,00
GO:1903747	regulation of establishment of protein localization to mitochondrion	GO_BiologicalProcess-EBI-UniProt-GOA-ACAP-ARAP_08.05.2020_00h00	0,016363574	0,029491413	2,84964E-05	8,1812E-05	[5, 6, 7, 8]	Group131	7,69	6,00
GO:0097153	cysteine-type endopeptidase activity involved in apoptotic process	GO_BiologicalProcess-EBI-UniProt-GOA-ACAP-ARAP_08.05.2020_00h00	0,000249894	0,001232725	2,84964E-05	8,1812E-05	[5, 9, 10]	Group131	6,97	17,00

GO:0043281	regulation of cysteine-type endopeptidase activity involved in apoptotic process	GO_BiologicalProcess-EBI-UniProt-GOA-ACAP-ARAP_08.05.2020_00h00	0,000159916	0,000884625	2,84964E-05	8,1812E-05	[6, 7, 8, 9, 10, 11]	Group131	7,23	17,00
GO:0043154	negative regulation of cysteine-type endopeptidase activity involved in apoptotic process	GO_BiologicalProcess-EBI-UniProt-GOA-ACAP-ARAP_08.05.2020_00h00	0,000993196	0,003468936	2,84964E-05	8,1812E-05	[6, 7, 8, 9, 10, 11, 12]	Group131	9,28	9,00
GO:0006914	autophagy	GO_BiologicalProcess-EBI-UniProt-GOA-ACAP-ARAP_08.05.2020_00h00	0,030111073	0,046254087	3,29155E-05	9,2999E-05	[3, 4]	Group162	4,04	22,00
GO:0009894	regulation of catabolic process	GO_BiologicalProcess-EBI-UniProt-GOA-ACAP-ARAP_08.05.2020_00h00	0,002341402	0,006810703	3,29155E-05	9,2999E-05	[3, 4]	Group162	4,08	44,00
GO:0009057	macromolecule catabolic process	GO_BiologicalProcess-EBI-UniProt-GOA-ACAP-ARAP_08.05.2020_00h00	0,000587955	0,002364171	3,29155E-05	9,2999E-05	[4]	Group162	4,01	60,00
GO:0009895	negative regulation of catabolic process	GO_BiologicalProcess-EBI-UniProt-GOA-ACAP-ARAP_08.05.2020_00h00	0,004089869	0,010139908	3,29155E-05	9,2999E-05	[3, 4, 5]	Group162	5,26	18,00
GO:0030163	protein catabolic process	GO_BiologicalProcess-EBI-UniProt-GOA-ACAP-ARAP_08.05.2020_00h00	0,003023085	0,008126664	3,29155E-05	9,2999E-05	[4, 5]	Group162	4,10	41,00
GO:0031329	regulation of cellular catabolic process	GO_BiologicalProcess-EBI-UniProt-GOA-ACAP-ARAP_08.05.2020_00h00	0,00275227	0,007656751	3,29155E-05	9,2999E-05	[4, 5]	Group162	4,21	38,00
GO:0044265	cellular macromolecule catabolic process	GO_BiologicalProcess-EBI-UniProt-GOA-ACAP-ARAP_08.05.2020_00h00	0,000537689	0,002237255	3,29155E-05	9,2999E-05	[4, 5]	Group162	4,19	52,00
GO:0031330	negative regulation of cellular catabolic process	GO_BiologicalProcess-EBI-UniProt-GOA-ACAP-ARAP_08.05.2020_00h00	0,006713833	0,015047162	3,29155E-05	9,2999E-05	[4, 5, 6]	Group162	5,40	15,00
GO:0010608	posttranscriptional regulation of gene expression	GO_BiologicalProcess-EBI-UniProt-GOA-ACAP-ARAP_08.05.2020_00h00	0,000370525	0,001688533	3,29155E-05	9,2999E-05	[5, 6]	Group162	4,89	34,00
GO:0043487	regulation of RNA stability	GO_BiologicalProcess-EBI-UniProt-GOA-ACAP-ARAP_08.05.2020_00h00	0,014361648	0,026584327	3,29155E-05	9,2999E-05	[3, 5, 6, 7, 8]	Group162	5,61	11,00
GO:0006401	RNA catabolic process	GO_BiologicalProcess-EBI-UniProt-GOA-ACAP-ARAP_08.05.2020_00h00	0,009984245	0,020460218	3,29155E-05	9,2999E-05	[5, 6, 7]	Group162	4,64	20,00
GO:0007264	small GTPase mediated signal transduction	GO_BiologicalProcess-EBI-UniProt-GOA-ACAP-ARAP_08.05.2020_00h00	0,002490827	0,007158323	3,86397E-05	0,00010747	[4, 5, 6]	Group120	4,65	28,00
GO:0043087	regulation of GTPase activity	GO_BiologicalProcess-EBI-UniProt-GOA-ACAP-ARAP_08.05.2020_00h00	0,000172834	0,000924034	3,86397E-05	0,00010747	[5]	Group120	5,53	28,00
GO:0051345	positive regulation of hydrolase activity	GO_BiologicalProcess-EBI-UniProt-GOA-ACAP-ARAP_08.05.2020_00h00	9,69574E-06	8,7536E-05	3,86397E-05	0,00010747	[5]	Group120	5,30	43,00
GO:0007265	Ras protein signal transduction	GO_BiologicalProcess-EBI-UniProt-GOA-ACAP-ARAP_08.05.2020_00h00	0,009073091	0,019167656	3,86397E-05	0,00010747	[5, 6, 7]	Group120	4,68	20,00
GO:0043547	positive regulation of GTPase activity	GO_BiologicalProcess-EBI-UniProt-GOA-ACAP-ARAP_08.05.2020_00h00	0,000437988	0,001896626	3,86397E-05	0,00010747	[6]	Group120	5,58	24,00
GO:0005096	GTPase activator activity	GO_BiologicalProcess-EBI-UniProt-GOA-ACAP-ARAP_08.05.2020_00h00	0,004219768	0,010354661	3,86397E-05	0,00010747	[6, 7]	Group120	5,52	16,00
GO:0007492	endoderm development	GO_BiologicalProcess-EBI-UniProt-GOA-ACAP-ARAP_08.05.2020_00h00	0,000448379	0,001924211	4,22951E-05	0,00011582	[4]	Group153	10,34	9,00
GO:0048598	embryonic morphogenesis	GO_BiologicalProcess-EBI-UniProt-GOA-ACAP-ARAP_08.05.2020_00h00	0,000934627	0,003362548	4,22951E-05	0,00011582	[3, 4, 5]	Group153	4,79	31,00
GO:0001704	formation of primary germ layer	GO_BiologicalProcess-EBI-UniProt-GOA-ACAP-ARAP_08.05.2020_00h00	0,008327947	0,017869608	4,22951E-05	0,00011582	[3, 4, 5, 6, 7]	Group153	6,77	9,00

GO:0007369	gastrulation	GO_BiologicalProcess-EBI-UniProt-GOA-ACAP-ARAP_08.05.2020_00h00	0,00706902	0,015696175	4,22951E-05	0,00011582	[4, 5, 6]	Group153	5,94	12,00
GO:0034446	substrate adhesion-dependent cell spreading	GO_BiologicalProcess-EBI-UniProt-GOA-ACAP-ARAP_08.05.2020_00h00	0,007866058	0,017108677	4,22951E-05	0,00011582	[4, 5, 6]	Group153	7,34	8,00
GO:0085029	extracellular matrix assembly	GO_BiologicalProcess-EBI-UniProt-GOA-ACAP-ARAP_08.05.2020_00h00	0,001065304	0,003680489	4,22951E-05	0,00011582	[5, 6]	Group153	13,33	6,00
GO:0001706	endoderm formation	GO_BiologicalProcess-EBI-UniProt-GOA-ACAP-ARAP_08.05.2020_00h00	0,000936067	0,003342597	4,22951E-05	0,00011582	[4, 5, 6, 7, 8]	Group153	11,67	7,00
GO:0035987	endodermal cell differentiation	GO_BiologicalProcess-EBI-UniProt-GOA-ACAP-ARAP_08.05.2020_00h00	0,009662041	0,020057641	4,22951E-05	0,00011582	[4, 5, 6, 7, 8, 9]	Group153	10,00	5,00
GO:0070831	basement membrane assembly	GO_BiologicalProcess-EBI-UniProt-GOA-ACAP-ARAP_08.05.2020_00h00	0,009205425	0,019319279	4,22951E-05	0,00011582	[6, 7]	Group153	17,65	3,00
GO:0031581	hemidesmosome assembly	GO_BiologicalProcess-EBI-UniProt-GOA-ACAP-ARAP_08.05.2020_00h00	1,29834E-05	0,000111938	4,22951E-05	0,00011582	[7]	Group153	38,46	5,00
GO:0010812	negative regulation of cell-substrate adhesion	GO_BiologicalProcess-EBI-UniProt-GOA-ACAP-ARAP_08.05.2020_00h00	0,032554209	0,04921703	5,06855E-05	0,0001367	[4, 5, 6]	Group078	7,35	5,00
GO:0051017	actin filament bundle assembly	GO_BiologicalProcess-EBI-UniProt-GOA-ACAP-ARAP_08.05.2020_00h00	2,96766E-05	0,000225401	5,06855E-05	0,0001367	[5, 6, 7, 9]	Group078	9,09	15,00
GO:0032231	regulation of actin filament bundle assembly	GO_BiologicalProcess-EBI-UniProt-GOA-ACAP-ARAP_08.05.2020_00h00	5,44583E-06	5,48596E-05	5,06855E-05	0,0001367	[4, 5, 6, 7, 8, 9, 10]	Group078	11,93	13,00
GO:0032232	negative regulation of actin filament bundle assembly	GO_BiologicalProcess-EBI-UniProt-GOA-ACAP-ARAP_08.05.2020_00h00	0,010381774	0,021094177	5,06855E-05	0,0001367	[5, 6, 7, 8, 9, 10, 11]	Group078	12,12	4,00
GO:0031644	regulation of nervous system process	GO_BiologicalProcess-EBI-UniProt-GOA-ACAP-ARAP_08.05.2020_00h00	0,003465883	0,008988754	6,46096E-05	0,00017165	[4, 5]	Group158	6,83	11,00
GO:0031646	positive regulation of nervous system process	GO_BiologicalProcess-EBI-UniProt-GOA-ACAP-ARAP_08.05.2020_00h00	0,000627276	0,00248059	6,46096E-05	0,00017165	[3, 4, 5, 6]	Group158	10,96	8,00
GO:1901890	positive regulation of cell junction assembly	GO_BiologicalProcess-EBI-UniProt-GOA-ACAP-ARAP_08.05.2020_00h00	0,023341313	0,038579684	6,46096E-05	0,00017165	[4, 5, 6, 7]	Group158	6,48	7,00
GO:0050806	positive regulation of synaptic transmission	GO_BiologicalProcess-EBI-UniProt-GOA-ACAP-ARAP_08.05.2020_00h00	0,010380722	0,021136915	6,46096E-05	0,00017165	[3, 4, 5, 6, 7, 8, 9]	Group158	5,88	11,00
GO:0099068	postsynapse assembly	GO_BiologicalProcess-EBI-UniProt-GOA-ACAP-ARAP_08.05.2020_00h00	0,009311849	0,0194149	6,46096E-05	0,00017165	[5, 6, 7]	Group158	12,50	4,00
GO:0106027	neuron projection organization	GO_BiologicalProcess-EBI-UniProt-GOA-ACAP-ARAP_08.05.2020_00h00	0,015182317	0,02772801	6,46096E-05	0,00017165	[6]	Group158	7,07	7,00
GO:1904646	cellular response to amyloid-beta	GO_BiologicalProcess-EBI-UniProt-GOA-ACAP-ARAP_08.05.2020_00h00	0,018466409	0,032485943	6,46096E-05	0,00017165	[6, 7]	Group158	10,26	4,00
GO:0060291	long-term synaptic potentiation	GO_BiologicalProcess-EBI-UniProt-GOA-ACAP-ARAP_08.05.2020_00h00	0,012985187	0,02500367	6,46096E-05	0,00017165	[4, 5, 6, 7, 8, 9, 10]	Group158	7,29	7,00
GO:0098815	modulation of excitatory postsynaptic potential	GO_BiologicalProcess-EBI-UniProt-GOA-ACAP-ARAP_08.05.2020_00h00	0,008146646	0,017638776	6,46096E-05	0,00017165	[4, 5, 6, 7, 8, 9, 10]	Group158	10,42	5,00
GO:0060996	dendritic spine development	GO_BiologicalProcess-EBI-UniProt-GOA-ACAP-ARAP_08.05.2020_00h00	0,031476401	0,047890171	6,46096E-05	0,00017165	[3, 4, 7, 8, 10, 11, 12]	Group158	6,09	7,00
GO:2001257	regulation of cation channel activity	GO_BiologicalProcess-EBI-UniProt-GOA-ACAP-ARAP_08.05.2020_00h00	0,002512984	0,007178881	6,46096E-05	0,00017165	[6, 7, 8, 9, 10]	Group158	6,47	13,00
GO:0019637	organophosphate metabolic process	GO_BiologicalProcess-EBI-UniProt-GOA-ACAP-ARAP_08.05.2020_00h00	0,000611052	0,002436569	7,36423E-05	0,00019277	[3, 4]	Group124	4,26	48,00

GO:0055086	nucleobase-containing small molecule metabolic process	GO_BiologicalProcess-EBI-UniProt-GOA-ACAP-ARAP_08.05.2020_00h00	0,003933324	0,009853902	7,36423E-05	0,00019277	[3, 4, 5]	Group124	4,41	30,00
GO:0090407	organophosphate biosynthetic process	GO_BiologicalProcess-EBI-UniProt-GOA-ACAP-ARAP_08.05.2020_00h00	0,000779392	0,002936529	7,36423E-05	0,00019277	[4, 5]	Group124	4,79	32,00
GO:0006140	regulation of nucleotide metabolic process	GO_BiologicalProcess-EBI-UniProt-GOA-ACAP-ARAP_08.05.2020_00h00	0,023751667	0,039055575	7,36423E-05	0,00019277	[4, 5, 6, 7, 8]	Group124	6,02	8,00
GO:0009117	nucleotide metabolic process	GO_BiologicalProcess-EBI-UniProt-GOA-ACAP-ARAP_08.05.2020_00h00	0,022016035	0,036963765	7,36423E-05	0,00019277	[5, 6, 7]	Group124	4,07	24,00
GO:0009179	purine ribonucleoside diphosphate metabolic process	GO_BiologicalProcess-EBI-UniProt-GOA-ACAP-ARAP_08.05.2020_00h00	0,007714132	0,01689342	7,36423E-05	0,00019277	[7, 8, 9]	Group124	6,45	10,00
GO:0098801	regulation of renal system process	GO_BiologicalProcess-EBI-UniProt-GOA-ACAP-ARAP_08.05.2020_00h00	0,003317585	0,008674669	8,34554E-05	0,00021529	[4, 5]	Group101	12,82	5,00
GO:0032715	negative regulation of interleukin-6 production	GO_BiologicalProcess-EBI-UniProt-GOA-ACAP-ARAP_08.05.2020_00h00	0,007454556	0,016362408	8,34554E-05	0,00021529	[4, 5, 6, 7, 8]	Group101	10,64	5,00
GO:0033135	regulation of peptidyl-serine phosphorylation	GO_BiologicalProcess-EBI-UniProt-GOA-ACAP-ARAP_08.05.2020_00h00	0,011230711	0,022437977	8,34554E-05	0,00021529	[7, 8, 9, 10]	Group101	6,10	10,00
GO:0043027	cysteine-type endopeptidase inhibitor activity involved in apoptotic process	GO_BiologicalProcess-EBI-UniProt-GOA-ACAP-ARAP_08.05.2020_00h00	0,032678593	0,04924947	8,34554E-05	0,00021529	[5, 6, 7, 8, 9, 10, 11, 12, 13]	Group101	11,11	3,00
GO:0043154	negative regulation of cysteine-type endopeptidase activity involved in apoptotic process	GO_BiologicalProcess-EBI-UniProt-GOA-ACAP-ARAP_08.05.2020_00h00	0,000993196	0,003468936	8,34554E-05	0,00021529	[6, 7, 8, 9, 10, 11, 12]	Group101	9,28	9,00
GO:0001816	cytokine production	GO_BiologicalProcess-EBI-UniProt-GOA-ACAP-ARAP_08.05.2020_00h00	0,000179184	0,00095266	0,000101219	0,00025738	[2, 5]	Group123	4,75	41,00
GO:1904951	positive regulation of establishment of protein localization	GO_BiologicalProcess-EBI-UniProt-GOA-ACAP-ARAP_08.05.2020_00h00	0,032269876	0,048864357	0,000101219	0,00025738	[2, 3, 4, 5, 6]	Group123	4,05	21,00
GO:0001817	regulation of cytokine production	GO_BiologicalProcess-EBI-UniProt-GOA-ACAP-ARAP_08.05.2020_00h00	0,001565843	0,005045494	0,000101219	0,00025738	[3, 4, 5, 6]	Group123	4,46	35,00
GO:0032633	interleukin-4 production	GO_BiologicalProcess-EBI-UniProt-GOA-ACAP-ARAP_08.05.2020_00h00	0,002621352	0,007400101	0,000101219	0,00025738	[3, 6]	Group123	13,51	5,00
GO:0009306	protein secretion	GO_BiologicalProcess-EBI-UniProt-GOA-ACAP-ARAP_08.05.2020_00h00	0,017033646	0,030412685	0,000101219	0,00025738	[4, 5, 6, 7, 8]	Group123	4,05	27,00
GO:0050663	cytokine secretion	GO_BiologicalProcess-EBI-UniProt-GOA-ACAP-ARAP_08.05.2020_00h00	0,028077139	0,044266593	0,000101219	0,00025738	[3, 4, 5, 6, 7, 8, 9]	Group123	4,76	13,00
GO:0016032	viral process	GO_BiologicalProcess-EBI-UniProt-GOA-ACAP-ARAP_08.05.2020_00h00	6,47297E-05	0,000427216	0,00012331	0,00030914	[3]	Group091	4,77	46,00
GO:0051701	interaction with host	GO_BiologicalProcess-EBI-UniProt-GOA-ACAP-ARAP_08.05.2020_00h00	0,013796489	0,026041893	0,00012331	0,00030914	[3]	Group091	5,43	12,00
GO:0019058	viral life cycle	GO_BiologicalProcess-EBI-UniProt-GOA-ACAP-ARAP_08.05.2020_00h00	0,010250798	0,020916874	0,00012331	0,00030914	[4]	Group091	4,90	17,00
GO:0046718	viral entry into host cell	GO_BiologicalProcess-EBI-UniProt-GOA-ACAP-ARAP_08.05.2020_00h00	0,023751667	0,039055575	0,00012331	0,00030914	[5, 6]	Group091	6,02	8,00
GO:0001885	endothelial cell development	GO_BiologicalProcess-EBI-UniProt-GOA-ACAP-ARAP_08.05.2020_00h00	0,000314285	0,001481627	0,000126811	0,00031351	[5, 6, 7]	Group064	12,12	8,00
GO:0071287	cellular response to manganese ion	GO_BiologicalProcess-EBI-UniProt-GOA-ACAP-ARAP_08.05.2020_00h00	0,004184007	0,010319832	0,000126811	0,00031351	[6]	Group064	23,08	3,00
GO:0071320	cellular response to cAMP	GO_BiologicalProcess-EBI-UniProt-GOA-ACAP-ARAP_08.05.2020_00h00	0,003981134	0,009921734	0,000126811	0,00031351	[5, 6, 7]	Group064	10,34	6,00

GO:0001935	endothelial cell proliferation	GO_BiologicalProcess-EBI-UniProt-GOA-ACAP-ARAP_08.05.2020_00h00	0,003633375	0,00934715	0,000148303	0,00036162	[4]	Group137	6,79	11,00
GO:0001936	regulation of endothelial cell proliferation	GO_BiologicalProcess-EBI-UniProt-GOA-ACAP-ARAP_08.05.2020_00h00	0,014143549	0,026282284	0,000148303	0,00036162	[5, 6]	Group137	6,21	9,00
GO:0040037	negative regulation of fibroblast growth factor receptor signaling pathway	GO_BiologicalProcess-EBI-UniProt-GOA-ACAP-ARAP_08.05.2020_00h00	0,012620698	0,024499003	0,000148303	0,00036162	[4, 5, 6, 7, 8, 9, 10]	Group137	15,79	3,00
GO:0070306	lens fiber cell differentiation	GO_BiologicalProcess-EBI-UniProt-GOA-ACAP-ARAP_08.05.2020_00h00	0,015443421	0,028044315	0,000148303	0,00036162	[4, 5, 6, 8, 9, 10]	Group137	10,81	4,00
GO:0002040	sprouting angiogenesis	GO_BiologicalProcess-EBI-UniProt-GOA-ACAP-ARAP_08.05.2020_00h00	0,00041584	0,001833911	0,000148303	0,00036162	[4, 5, 6, 7, 8, 9, 10, 11]	Group137	8,87	11,00
GO:0002043	blood vessel endothelial cell proliferation involved in sprouting angiogenesis	GO_BiologicalProcess-EBI-UniProt-GOA-ACAP-ARAP_08.05.2020_00h00	0,00033793	0,001562316	0,000148303	0,00036162	[5, 6, 7, 8, 9, 10, 11, 12]	Group137	20,83	5,00
GO:1903670	regulation of sprouting angiogenesis	GO_BiologicalProcess-EBI-UniProt-GOA-ACAP-ARAP_08.05.2020_00h00	0,003441989	0,008951043	0,000148303	0,00036162	[5, 6, 7, 8, 9, 10, 11, 12]	Group137	9,33	7,00
GO:0015278	calcium-release channel activity	GO_BiologicalProcess-EBI-UniProt-GOA-ACAP-ARAP_08.05.2020_00h00	0,010487842	0,021219587	0,000158836	0,00038206	[5, 6, 7, 8, 9, 10, 11, 12, 13, 14, 15]	Group151	9,80	5,00
GO:0051281	positive regulation of release of sequestered calcium ion into cytosol	GO_BiologicalProcess-EBI-UniProt-GOA-ACAP-ARAP_08.05.2020_00h00	0,025549145	0,041231927	0,000158836	0,00038206	[4, 5, 6, 7, 8, 9, 10, 11, 12, 13, 14, 15, 16]	Group151	9,30	4,00
GO:0051651	maintenance of location in cell	GO_BiologicalProcess-EBI-UniProt-GOA-ACAP-ARAP_08.05.2020_00h00	0,00192218	0,005914877	0,000158836	0,00038206	[2, 3, 4]	Group151	6,17	15,00
GO:0034764	positive regulation of transmembrane transport	GO_BiologicalProcess-EBI-UniProt-GOA-ACAP-ARAP_08.05.2020_00h00	0,031413406	0,047870431	0,000158836	0,00038206	[3, 4, 5, 6]	Group151	4,98	11,00
GO:0043270	positive regulation of ion transport	GO_BiologicalProcess-EBI-UniProt-GOA-ACAP-ARAP_08.05.2020_00h00	0,029049521	0,045351375	0,000158836	0,00038206	[3, 4, 5, 6]	Group151	4,62	14,00
GO:0051208	sequestering of calcium ion	GO_BiologicalProcess-EBI-UniProt-GOA-ACAP-ARAP_08.05.2020_00h00	0,000410002	0,001824987	0,000158836	0,00038206	[3, 4, 5, 8, 10]	Group151	8,33	12,00
GO:0010522	regulation of calcium ion transport into cytosol	GO_BiologicalProcess-EBI-UniProt-GOA-ACAP-ARAP_08.05.2020_00h00	0,007866058	0,017108677	0,000158836	0,00038206	[3, 4, 5, 7, 8, 9, 10, 11, 13]	Group151	7,34	8,00
GO:0060402	calcium ion transport into cytosol	GO_BiologicalProcess-EBI-UniProt-GOA-ACAP-ARAP_08.05.2020_00h00	0,000377397	0,001703626	0,000158836	0,00038206	[4, 10, 12]	Group151	7,57	14,00
GO:0051209	release of sequestered calcium ion into cytosol	GO_BiologicalProcess-EBI-UniProt-GOA-ACAP-ARAP_08.05.2020_00h00	0,0002773	0,001333548	0,000158836	0,00038206	[4, 5, 6, 7, 8, 10, 11, 12, 13, 14]	Group151	8,70	12,00
GO:1903053	regulation of extracellular matrix organization	GO_BiologicalProcess-EBI-UniProt-GOA-ACAP-ARAP_08.05.2020_00h00	0,006195141	0,01411607	0,000184808	0,00043861	[4, 5, 6]	Group067	11,11	5,00
GO:0022617	extracellular matrix disassembly	GO_BiologicalProcess-EBI-UniProt-GOA-ACAP-ARAP_08.05.2020_00h00	0,000448379	0,001924211	0,000184808	0,00043861	[5, 6]	Group067	10,34	9,00
GO:0010715	regulation of extracellular matrix disassembly	GO_BiologicalProcess-EBI-UniProt-GOA-ACAP-ARAP_08.05.2020_00h00	0,012620698	0,024499003	0,000184808	0,00043861	[5, 6, 7]	Group067	15,79	3,00
GO:0040008	regulation of growth	GO_BiologicalProcess-EBI-UniProt-GOA-ACAP-ARAP_08.05.2020_00h00	0,002803932	0,007710812	0,000192469	0,00045078	[2, 3]	Group166	4,42	32,00
GO:0045927	positive regulation of growth	GO_BiologicalProcess-EBI-UniProt-GOA-ACAP-ARAP_08.05.2020_00h00	0,01690525	0,030296486	0,000192469	0,00045078	[2, 3, 4]	Group166	4,96	14,00
GO:0048638	regulation of developmental growth	GO_BiologicalProcess-EBI-UniProt-GOA-ACAP-ARAP_08.05.2020_00h00	0,014668097	0,026943127	0,000192469	0,00045078	[3, 4]	Group166	4,71	17,00

GO:0001558	regulation of cell growth	GO_BiologicalProcess-EBI-UniProt-GOA-ACAP-ARAP_08.05.2020_00h00	0,028421564	0,044662461	0,000192469	0,00045078	[3, 4, 5]	Group166	4,22	19,00
GO:0046620	regulation of organ growth	GO_BiologicalProcess-EBI-UniProt-GOA-ACAP-ARAP_08.05.2020_00h00	0,024403341	0,039853238	0,000192469	0,00045078	[3, 4, 5]	Group166	6,42	7,00
GO:0048588	developmental cell growth	GO_BiologicalProcess-EBI-UniProt-GOA-ACAP-ARAP_08.05.2020_00h00	0,01525921	0,027815361	0,000192469	0,00045078	[3, 4, 5]	Group166	5,18	13,00
GO:0030307	positive regulation of cell growth	GO_BiologicalProcess-EBI-UniProt-GOA-ACAP-ARAP_08.05.2020_00h00	0,020310845	0,034771878	0,000192469	0,00045078	[3, 4, 5, 6]	Group166	5,56	10,00
GO:0120039	plasma membrane bounded cell projection morphogenesis	GO_BiologicalProcess-EBI-UniProt-GOA-ACAP-ARAP_08.05.2020_00h00	0,009786652	0,020228567	0,000192469	0,00045078	[5, 6, 7]	Group166	4,12	30,00
GO:0055024	regulation of cardiac muscle tissue development	GO_BiologicalProcess-EBI-UniProt-GOA-ACAP-ARAP_08.05.2020_00h00	0,008760029	0,018588355	0,000192469	0,00045078	[5, 6, 7, 8, 9]	Group166	7,87	7,00
GO:0048667	cell morphogenesis involved in neuron differentiation	GO_BiologicalProcess-EBI-UniProt-GOA-ACAP-ARAP_08.05.2020_00h00	0,006632533	0,014899846	0,000192469	0,00045078	[5, 6, 8, 9, 10]	Group166	4,33	28,00
GO:0060043	regulation of cardiac muscle cell proliferation	GO_BiologicalProcess-EBI-UniProt-GOA-ACAP-ARAP_08.05.2020_00h00	0,031797417	0,04830179	0,000192469	0,00045078	[4, 5, 6, 7, 8, 9, 10, 11]	Group166	8,70	4,00
GO:0061564	axon development	GO_BiologicalProcess-EBI-UniProt-GOA-ACAP-ARAP_08.05.2020_00h00	0,00784214	0,01709551	0,000192469	0,00045078	[6, 7, 9, 10, 11]	Group166	4,42	25,00
GO:0043154	negative regulation of cysteine-type endopeptidase activity involved in apoptotic process	GO_BiologicalProcess-EBI-UniProt-GOA-ACAP-ARAP_08.05.2020_00h00	0,000993196	0,003468936	0,000192469	0,00045078	[6, 7, 8, 9, 10, 11, 12]	Group166	9,28	9,00
GO:0001816	cytokine production	GO_BiologicalProcess-EBI-UniProt-GOA-ACAP-ARAP_08.05.2020_00h00	0,000179184	0,00095266	0,000205909	0,000476	[2, 5]	Group113	4,75	41,00
GO:0001817	regulation of cytokine production	GO_BiologicalProcess-EBI-UniProt-GOA-ACAP-ARAP_08.05.2020_00h00	0,001565843	0,005045494	0,000205909	0,000476	[3, 4, 5, 6]	Group113	4,46	35,00
GO:0001819	positive regulation of cytokine production	GO_BiologicalProcess-EBI-UniProt-GOA-ACAP-ARAP_08.05.2020_00h00	0,013490905	0,025718717	0,000205909	0,000476	[3, 4, 5, 6, 7]	Group113	4,37	22,00
GO:0051091	positive regulation of DNA-binding transcription factor activity	GO_BiologicalProcess-EBI-UniProt-GOA-ACAP-ARAP_08.05.2020_00h00	0,018849541	0,03297808	0,000205909	0,000476	[2, 3, 4, 7, 8, 9, 10, 11, 12, 13]	Group113	4,90	14,00
GO:0051092	positive regulation of NF-kappaB transcription factor activity	GO_BiologicalProcess-EBI-UniProt-GOA-ACAP-ARAP_08.05.2020_00h00	0,012154936	0,023885572	0,000205909	0,000476	[3, 4, 5, 8, 9, 10, 11, 12, 13, 14]	Group113	6,02	10,00
GO:0033002	muscle cell proliferation	GO_BiologicalProcess-EBI-UniProt-GOA-ACAP-ARAP_08.05.2020_00h00	3,12928E-05	0,000233963	0,000281373	0,00064211	[3]	Group156	8,25	17,00
GO:0035019	somatic stem cell population maintenance	GO_BiologicalProcess-EBI-UniProt-GOA-ACAP-ARAP_08.05.2020_00h00	0,020465165	0,034911167	0,000281373	0,00064211	[3, 4]	Group156	7,32	6,00
GO:0046620	regulation of organ growth	GO_BiologicalProcess-EBI-UniProt-GOA-ACAP-ARAP_08.05.2020_00h00	0,024403341	0,039853238	0,000281373	0,00064211	[3, 4, 5]	Group156	6,42	7,00
GO:0050679	positive regulation of epithelial cell proliferation	GO_BiologicalProcess-EBI-UniProt-GOA-ACAP-ARAP_08.05.2020_00h00	0,00018895	0,000982745	0,000281373	0,00064211	[4, 5, 6]	Group156	7,41	16,00
GO:0101023	vascular endothelial cell proliferation	GO_BiologicalProcess-EBI-UniProt-GOA-ACAP-ARAP_08.05.2020_00h00	0,026696119	0,042651396	0,000281373	0,00064211	[5]	Group156	12,00	3,00
GO:0055024	regulation of cardiac muscle tissue development	GO_BiologicalProcess-EBI-UniProt-GOA-ACAP-ARAP_08.05.2020_00h00	0,008760029	0,018588355	0,000281373	0,00064211	[5, 6, 7, 8, 9]	Group156	7,87	7,00
GO:0060487	lung epithelial cell differentiation	GO_BiologicalProcess-EBI-UniProt-GOA-ACAP-ARAP_08.05.2020_00h00	0,018932438	0,03306267	0,000281373	0,00064211	[5, 6, 7, 8, 9]	Group156	13,64	3,00
GO:1905564	positive regulation of vascular endothelial cell proliferation	GO_BiologicalProcess-EBI-UniProt-GOA-ACAP-ARAP_08.05.2020_00h00	0,014564467	0,026855865	0,000281373	0,00064211	[6, 7, 8]	Group156	15,00	3,00

GO:0060043	regulation of cardiac muscle cell proliferation	GO_BiologicalProcess-EBI-UniProt-GOA-ACAP-ARAP_08.05.2020_00h00	0,031797417	0,04830179	0,000281373	0,00064211	[4, 5, 6, 7, 8, 9, 10, 11]	Group156	8,70	4,00
GO:2001169	regulation of ATP biosynthetic process	GO_BiologicalProcess-EBI-UniProt-GOA-ACAP-ARAP_08.05.2020_00h00	0,014564467	0,026855865	0,000281373	0,00064211	[4, 5, 7, 8, 9, 10, 11, 12]	Group156	15,00	3,00
GO:1903320	regulation of protein modification by small protein conjugation or removal	GO_BiologicalProcess-EBI-UniProt-GOA-ACAP-ARAP_08.05.2020_00h00	0,000302301	0,001439316	0,000302301	0,00068113	[6, 7, 8]	Group052	6,85	17,00
GO:0031398	positive regulation of protein ubiquitination	GO_BiologicalProcess-EBI-UniProt-GOA-ACAP-ARAP_08.05.2020_00h00	0,001763233	0,005550702	0,000302301	0,00068113	[7, 8, 9, 10, 11]	Group052	7,94	10,00
GO:0051020	GTPase binding	GO_MolecularFunction-EBI-UniProt-GOA-ACAP-ARAP_08.05.2020_00h00	0,012022416	0,023673771	0,000323321	0,00071939	[4]	Group106	4,26	25,00
GO:0005088	Ras guanyl-nucleotide exchange factor activity	GO_BiologicalProcess-EBI-UniProt-GOA-ACAP-ARAP_08.05.2020_00h00	0,018565632	0,03260057	0,000323321	0,00071939	[5]	Group106	6,30	8,00
GO:0031267	small GTPase binding	GO_MolecularFunction-EBI-UniProt-GOA-ACAP-ARAP_08.05.2020_00h00	0,001787578	0,005590562	0,000323321	0,00071939	[5]	Group106	5,12	23,00
GO:0043087	regulation of GTPase activity	GO_BiologicalProcess-EBI-UniProt-GOA-ACAP-ARAP_08.05.2020_00h00	0,000172834	0,000924034	0,000323321	0,00071939	[5]	Group106	5,53	28,00
GO:0043547	positive regulation of GTPase activity	GO_BiologicalProcess-EBI-UniProt-GOA-ACAP-ARAP_08.05.2020_00h00	0,000437988	0,001896626	0,000323321	0,00071939	[6]	Group106	5,58	24,00
GO:0009894	regulation of catabolic process	GO_BiologicalProcess-EBI-UniProt-GOA-ACAP-ARAP_08.05.2020_00h00	0,002341402	0,006810703	0,000329505	0,0007241	[3, 4]	Group114	4,08	44,00
GO:0009895	negative regulation of catabolic process	GO_BiologicalProcess-EBI-UniProt-GOA-ACAP-ARAP_08.05.2020_00h00	0,004089869	0,010139908	0,000329505	0,0007241	[3, 4, 5]	Group114	5,26	18,00
GO:0031329	regulation of cellular catabolic process	GO_BiologicalProcess-EBI-UniProt-GOA-ACAP-ARAP_08.05.2020_00h00	0,00275227	0,007656751	0,000329505	0,0007241	[4, 5]	Group114	4,21	38,00
GO:0031330	negative regulation of cellular catabolic process	GO_BiologicalProcess-EBI-UniProt-GOA-ACAP-ARAP_08.05.2020_00h00	0,006713833	0,015047162	0,000329505	0,0007241	[4, 5, 6]	Group114	5,40	15,00
GO:0042177	negative regulation of protein catabolic process	GO_BiologicalProcess-EBI-UniProt-GOA-ACAP-ARAP_08.05.2020_00h00	0,032142218	0,048748184	0,000329505	0,0007241	[4, 5, 6, 7]	Group114	5,67	8,00
GO:0045861	negative regulation of proteolysis	GO_BiologicalProcess-EBI-UniProt-GOA-ACAP-ARAP_08.05.2020_00h00	0,006054374	0,013861331	0,000329505	0,0007241	[5, 6, 7, 8]	Group114	4,96	19,00
GO:0010035	response to inorganic substance	GO_BiologicalProcess-EBI-UniProt-GOA-ACAP-ARAP_08.05.2020_00h00	0,01391736	0,026218332	0,000336764	0,00073102	[3]	Group170	4,16	26,00
GO:0042493	response to drug	GO_BiologicalProcess-EBI-UniProt-GOA-ACAP-ARAP_08.05.2020_00h00	0,000187942	0,000982845	0,000336764	0,00073102	[3]	Group170	4,38	52,00
GO:0046677	response to antibiotic	GO_BiologicalProcess-EBI-UniProt-GOA-ACAP-ARAP_08.05.2020_00h00	0,003973101	0,009927566	0,000336764	0,00073102	[3]	Group170	5,16	19,00
GO:0071216	cellular response to biotic stimulus	GO_BiologicalProcess-EBI-UniProt-GOA-ACAP-ARAP_08.05.2020_00h00	0,008525003	0,018251516	0,000336764	0,00073102	[3]	Group170	5,41	14,00
GO:0000302	response to reactive oxygen species	GO_BiologicalProcess-EBI-UniProt-GOA-ACAP-ARAP_08.05.2020_00h00	0,033104222	0,049656335	0,000336764	0,00073102	[4]	Group170	4,78	12,00
GO:0062197	cellular response to chemical stress	GO_BiologicalProcess-EBI-UniProt-GOA-ACAP-ARAP_08.05.2020_00h00	0,013435385	0,025715327	0,000336764	0,00073102	[4]	Group170	4,66	18,00
GO:0071236	cellular response to antibiotic	GO_BiologicalProcess-EBI-UniProt-GOA-ACAP-ARAP_08.05.2020_00h00	0,011685916	0,023058603	0,000336764	0,00073102	[4]	Group170	6,06	10,00
GO:0097237	cellular response to toxic substance	GO_BiologicalProcess-EBI-UniProt-GOA-ACAP-ARAP_08.05.2020_00h00	0,015992491	0,028931595	0,000336764	0,00073102	[4]	Group170	5,00	14,00

GO:0042542	response to hydrogen peroxide	GO_BiologicalProcess-EBI-UniProt-GOA-ACAP-ARAP_08.05.2020_00h00	0,02252461	0,037685405	0,000336764	0,00073102	[4, 5]	Group170	5,73	9,00
GO:0034614	cellular response to reactive oxygen species	GO_BiologicalProcess-EBI-UniProt-GOA-ACAP-ARAP_08.05.2020_00h00	0,01962244	0,034081079	0,000336764	0,00073102	[5, 6]	Group170	5,59	10,00
GO:0032715	negative regulation of interleukin-6 production	GO_BiologicalProcess-EBI-UniProt-GOA-ACAP-ARAP_08.05.2020_00h00	0,007454556	0,016362408	0,000336764	0,00073102	[4, 5, 6, 7, 8]	Group170	10,64	5,00
GO:0070301	cellular response to hydrogen peroxide	GO_BiologicalProcess-EBI-UniProt-GOA-ACAP-ARAP_08.05.2020_00h00	0,02034726	0,034772012	0,000336764	0,00073102	[5, 6, 7]	Group170	6,67	7,00
GO:1903556	negative regulation of tumor necrosis factor superfamily cytokine production	GO_BiologicalProcess-EBI-UniProt-GOA-ACAP-ARAP_08.05.2020_00h00	0,029100992	0,045283981	0,000336764	0,00073102	[4, 5, 6, 7, 8]	Group170	7,58	5,00
GO:0071222	cellular response to lipopolysaccharide	GO_BiologicalProcess-EBI-UniProt-GOA-ACAP-ARAP_08.05.2020_00h00	0,006491162	0,014651042	0,000336764	0,00073102	[5, 6, 8]	Group170	5,78	13,00
GO:0033002	muscle cell proliferation	GO_BiologicalProcess-EBI-UniProt-GOA-ACAP-ARAP_08.05.2020_00h00	3,12928E-05	0,000233963	0,000403892	0,00086618	[3]	Group157	8,25	17,00
GO:0045927	positive regulation of growth	GO_BiologicalProcess-EBI-UniProt-GOA-ACAP-ARAP_08.05.2020_00h00	0,01690525	0,030296486	0,000403892	0,00086618	[2, 3, 4]	Group157	4,96	14,00
GO:0035019	somatic stem cell population maintenance	GO_BiologicalProcess-EBI-UniProt-GOA-ACAP-ARAP_08.05.2020_00h00	0,020465165	0,034911167	0,000403892	0,00086618	[3, 4]	Group157	7,32	6,00
GO:0048638	regulation of developmental growth	GO_BiologicalProcess-EBI-UniProt-GOA-ACAP-ARAP_08.05.2020_00h00	0,014668097	0,026943127	0,000403892	0,00086618	[3, 4]	Group157	4,71	17,00
GO:0046620	regulation of organ growth	GO_BiologicalProcess-EBI-UniProt-GOA-ACAP-ARAP_08.05.2020_00h00	0,024403341	0,039853238	0,000403892	0,00086618	[3, 4, 5]	Group157	6,42	7,00
GO:0001938	positive regulation of endothelial cell proliferation	GO_BiologicalProcess-EBI-UniProt-GOA-ACAP-ARAP_08.05.2020_00h00	0,02131371	0,036101274	0,000403892	0,00086618	[5, 6, 7]	Group157	6,60	7,00
GO:0002088	lens development in camera-type eye	GO_BiologicalProcess-EBI-UniProt-GOA-ACAP-ARAP_08.05.2020_00h00	0,009845688	0,020263061	0,000403892	0,00086618	[3, 7, 8, 9]	Group157	7,69	7,00
GO:0055024	regulation of cardiac muscle tissue development	GO_BiologicalProcess-EBI-UniProt-GOA-ACAP-ARAP_08.05.2020_00h00	0,008760029	0,018588355	0,000403892	0,00086618	[5, 6, 7, 8, 9]	Group157	7,87	7,00
GO:0060043	regulation of cardiac muscle cell proliferation	GO_BiologicalProcess-EBI-UniProt-GOA-ACAP-ARAP_08.05.2020_00h00	0,031797417	0,04830179	0,000403892	0,00086618	[4, 5, 6, 7, 8, 9, 10, 11]	Group157	8,70	4,00
GO:2000725	regulation of cardiac muscle cell differentiation	GO_BiologicalProcess-EBI-UniProt-GOA-ACAP-ARAP_08.05.2020_00h00	0,020105848	0,034731582	0,000403892	0,00086618	[5, 6, 7, 8, 9, 10]	Group157	10,00	4,00
GO:0030856	regulation of epithelial cell differentiation	GO_BiologicalProcess-EBI-UniProt-GOA-ACAP-ARAP_08.05.2020_00h00	0,02252461	0,037685405	0,000427715	0,00090635	[4, 5, 6]	Group164	5,73	9,00
GO:1904019	epithelial cell apoptotic process	GO_BiologicalProcess-EBI-UniProt-GOA-ACAP-ARAP_08.05.2020_00h00	3,38977E-05	0,000249539	0,000427715	0,00090635	[5]	Group164	10,81	12,00
GO:0030858	positive regulation of epithelial cell differentiation	GO_BiologicalProcess-EBI-UniProt-GOA-ACAP-ARAP_08.05.2020_00h00	0,025888894	0,041569918	0,000427715	0,00090635	[4, 5, 6, 7]	Group164	7,81	5,00
GO:0032715	negative regulation of interleukin-6 production	GO_BiologicalProcess-EBI-UniProt-GOA-ACAP-ARAP_08.05.2020_00h00	0,007454556	0,016362408	0,000427715	0,00090635	[4, 5, 6, 7, 8]	Group164	10,64	5,00
GO:0071356	cellular response to tumor necrosis factor	GO_BiologicalProcess-EBI-UniProt-GOA-ACAP-ARAP_08.05.2020_00h00	0,012930495	0,024948554	0,000427715	0,00090635	[6]	Group164	5,00	15,00
GO:0072577	endothelial cell apoptotic process	GO_BiologicalProcess-EBI-UniProt-GOA-ACAP-ARAP_08.05.2020_00h00	0,003648136	0,009359963	0,000427715	0,00090635	[6]	Group164	10,53	6,00
GO:1903556	negative regulation of tumor necrosis factor superfamily cytokine production	GO_BiologicalProcess-EBI-UniProt-GOA-ACAP-ARAP_08.05.2020_00h00	0,029100992	0,045283981	0,000427715	0,00090635	[4, 5, 6, 7, 8]	Group164	7,58	5,00

GO:1904035	regulation of epithelial cell apoptotic process	GO_BiologicalProcess-EBI-UniProt-GOA-ACAP-ARAP_08.05.2020_00h00	9,95666E-05	0,000610803	0,000427715	0,00090635	[6, 7]	Group164	11,36	10,00
GO:1904036	negative regulation of epithelial cell apoptotic process	GO_BiologicalProcess-EBI-UniProt-GOA-ACAP-ARAP_08.05.2020_00h00	0,000302994	0,001435473	0,000427715	0,00090635	[6, 7, 8]	Group164	14,00	7,00
GO:0060055	angiogenesis involved in wound healing	GO_BiologicalProcess-EBI-UniProt-GOA-ACAP-ARAP_08.05.2020_00h00	0,032678593	0,04924947	0,000427715	0,00090635	[4, 5, 6, 7, 8, 9, 10, 11]	Group164	11,11	3,00
GO:2000351	regulation of endothelial cell apoptotic process	GO_BiologicalProcess-EBI-UniProt-GOA-ACAP-ARAP_08.05.2020_00h00	0,002064665	0,006272649	0,000427715	0,00090635	[7, 8]	Group164	11,76	6,00
GO:2000352	negative regulation of endothelial cell apoptotic process	GO_BiologicalProcess-EBI-UniProt-GOA-ACAP-ARAP_08.05.2020_00h00	0,008316356	0,017884837	0,000427715	0,00090635	[7, 8, 9]	Group164	12,90	4,00
GO:0019838	growth factor binding	GO_MolecularFunction-EBI-UniProt-GOA-ACAP-ARAP_08.05.2020_00h00	0,000592761	0,002373526	0,000592761	0,00124131	[3]	Group071	8,00	12,00
GO:0005520	insulin-like growth factor binding	GO_MolecularFunction-EBI-UniProt-GOA-ACAP-ARAP_08.05.2020_00h00	1,53255E-05	0,000129792	0,000592761	0,00124131	[4]	Group071	21,88	7,00
GO:0031994	insulin-like growth factor I binding	GO_MolecularFunction-EBI-UniProt-GOA-ACAP-ARAP_08.05.2020_00h00	0,004184007	0,010319832	0,000592761	0,00124131	[5]	Group071	23,08	3,00
GO:0005165	neurotrophin receptor binding	GO_MolecularFunction-EBI-UniProt-GOA-ACAP-ARAP_08.05.2020_00h00	0,004184007	0,010319832	0,000625023	0,00129365	[5]	Group070	23,08	3,00
GO:0030159	signaling receptor complex adaptor activity	GO_MolecularFunction-EBI-UniProt-GOA-ACAP-ARAP_08.05.2020_00h00	0,031797417	0,04830179	0,000625023	0,00129365	[5]	Group070	8,70	4,00
GO:0038179	neurotrophin signaling pathway	GO_BiologicalProcess-EBI-UniProt-GOA-ACAP-ARAP_08.05.2020_00h00	0,001065304	0,003680489	0,000625023	0,00129365	[4, 5, 6]	Group070	13,33	6,00
GO:0018209	peptidyl-serine modification	GO_BiologicalProcess-EBI-UniProt-GOA-ACAP-ARAP_08.05.2020_00h00	0,000275328	0,001337508	0,000670247	0,00135573	[7, 8]	Group119	6,01	22,00
GO:0018105	peptidyl-serine phosphorylation	GO_BiologicalProcess-EBI-UniProt-GOA-ACAP-ARAP_08.05.2020_00h00	0,000650843	0,00255269	0,000670247	0,00135573	[7, 8, 9]	Group119	5,90	20,00
GO:0010951	negative regulation of endopeptidase activity	GO_BiologicalProcess-EBI-UniProt-GOA-ACAP-ARAP_08.05.2020_00h00	0,013479644	0,025748543	0,000670247	0,00135573	[7, 8, 9, 10]	Group119	5,11	14,00
GO:0033135	regulation of peptidyl-serine phosphorylation	GO_BiologicalProcess-EBI-UniProt-GOA-ACAP-ARAP_08.05.2020_00h00	0,011230711	0,022437977	0,000670247	0,00135573	[7, 8, 9, 10]	Group119	6,10	10,00
GO:0043027	cysteine-type endopeptidase inhibitor activity involved in apoptotic process	GO_BiologicalProcess-EBI-UniProt-GOA-ACAP-ARAP_08.05.2020_00h00	0,032678593	0,04924947	0,000670247	0,00135573	[5, 6, 7, 8, 9, 10, 11, 12, 13]	Group119	11,11	3,00
GO:0043154	negative regulation of cysteine-type endopeptidase activity involved in apoptotic process	GO_BiologicalProcess-EBI-UniProt-GOA-ACAP-ARAP_08.05.2020_00h00	0,000993196	0,003468936	0,000670247	0,00135573	[6, 7, 8, 9, 10, 11, 12]	Group119	9,28	9,00
GO:0002088	lens development in camera-type eye	GO_BiologicalProcess-EBI-UniProt-GOA-ACAP-ARAP_08.05.2020_00h00	0,009845688	0,020263061	0,000666914	0,00136449	[3, 7, 8, 9]	Group062	7,69	7,00
GO:0070306	lens fiber cell differentiation	GO_BiologicalProcess-EBI-UniProt-GOA-ACAP-ARAP_08.05.2020_00h00	0,015443421	0,028044315	0,000666914	0,00136449	[4, 5, 6, 8, 9, 10]	Group062	10,81	4,00
GO:0002043	blood vessel endothelial cell proliferation involved in sprouting angiogenesis	GO_BiologicalProcess-EBI-UniProt-GOA-ACAP-ARAP_08.05.2020_00h00	0,00033793	0,001562316	0,000666914	0,00136449	[5, 6, 7, 8, 9, 10, 11, 12]	Group062	20,83	5,00
GO:0071216	cellular response to biotic stimulus	GO_BiologicalProcess-EBI-UniProt-GOA-ACAP-ARAP_08.05.2020_00h00	0,008525003	0,018251516	0,000731504	0,00146301	[3]	Group167	5,41	14,00
GO:0032635	interleukin-6 production	GO_BiologicalProcess-EBI-UniProt-GOA-ACAP-ARAP_08.05.2020_00h00	0,001438689	0,004715156	0,000731504	0,00146301	[3, 6]	Group167	7,23	12,00

GO:0042542	response to hydrogen peroxide	GO_BiologicalProcess-EBI-UniProt-GOA-ACAP-ARAP_08.05.2020_00h00	0,02252461	0,037685405	0,000731504	0,00146301	[4, 5]	Group167	5,73	9,00
GO:0032652	regulation of interleukin-1 production	GO_BiologicalProcess-EBI-UniProt-GOA-ACAP-ARAP_08.05.2020_00h00	0,023341313	0,038579684	0,000731504	0,00146301	[4, 5, 6, 7]	Group167	6,48	7,00
GO:0032675	regulation of interleukin-6 production	GO_BiologicalProcess-EBI-UniProt-GOA-ACAP-ARAP_08.05.2020_00h00	0,000707176	0,002707069	0,000731504	0,00146301	[4, 5, 6, 7]	Group167	7,84	12,00
GO:0051250	negative regulation of lymphocyte activation	GO_BiologicalProcess-EBI-UniProt-GOA-ACAP-ARAP_08.05.2020_00h00	0,02252461	0,037685405	0,000731504	0,00146301	[4, 5, 6, 7]	Group167	5,73	9,00
GO:0032715	negative regulation of interleukin-6 production	GO_BiologicalProcess-EBI-UniProt-GOA-ACAP-ARAP_08.05.2020_00h00	0,007454556	0,016362408	0,000731504	0,00146301	[4, 5, 6, 7, 8]	Group167	10,64	5,00
GO:0070301	cellular response to hydrogen peroxide	GO_BiologicalProcess-EBI-UniProt-GOA-ACAP-ARAP_08.05.2020_00h00	0,02034726	0,034772012	0,000731504	0,00146301	[5, 6, 7]	Group167	6,67	7,00
GO:1903556	negative regulation of tumor necrosis factor superfamily cytokine production	GO_BiologicalProcess-EBI-UniProt-GOA-ACAP-ARAP_08.05.2020_00h00	0,029100992	0,045283981	0,000731504	0,00146301	[4, 5, 6, 7, 8]	Group167	7,58	5,00
GO:0071222	cellular response to lipopolysaccharide	GO_BiologicalProcess-EBI-UniProt-GOA-ACAP-ARAP_08.05.2020_00h00	0,006491162	0,014651042	0,000731504	0,00146301	[5, 6, 8]	Group167	5,78	13,00
GO:0097028	dendritic cell differentiation	GO_BiologicalProcess-EBI-UniProt-GOA-ACAP-ARAP_08.05.2020_00h00	0,029624119	0,045948595	0,000731504	0,00146301	[5, 6, 7, 8, 9]	Group167	8,89	4,00
GO:2000352	negative regulation of endothelial cell apoptotic process	GO_BiologicalProcess-EBI-UniProt-GOA-ACAP-ARAP_08.05.2020_00h00	0,008316356	0,017884837	0,000731504	0,00146301	[7, 8, 9]	Group167	12,90	4,00
GO:0043027	cysteine-type endopeptidase inhibitor activity involved in apoptotic process	GO_BiologicalProcess-EBI-UniProt-GOA-ACAP-ARAP_08.05.2020_00h00	0,032678593	0,04924947	0,000731504	0,00146301	[5, 6, 7, 8, 9, 10, 11, 12, 13]	Group167	11,11	3,00
GO:0051098	regulation of binding	GO_BiologicalProcess-EBI-UniProt-GOA-ACAP-ARAP_08.05.2020_00h00	0,000744828	0,002828573	0,000744828	0,0014731	[3]	Group107	5,58	22,00
GO:0043393	regulation of protein binding	GO_BiologicalProcess-EBI-UniProt-GOA-ACAP-ARAP_08.05.2020_00h00	0,000816717	0,003017755	0,000744828	0,0014731	[4]	Group107	6,73	15,00
GO:0051099	positive regulation of binding	GO_BiologicalProcess-EBI-UniProt-GOA-ACAP-ARAP_08.05.2020_00h00	0,000577287	0,002340946	0,000744828	0,0014731	[4]	Group107	7,25	14,00
GO:0051101	regulation of DNA binding	GO_BiologicalProcess-EBI-UniProt-GOA-ACAP-ARAP_08.05.2020_00h00	0,02470557	0,040278073	0,000744828	0,0014731	[4]	Group107	5,97	8,00
GO:0032092	positive regulation of protein binding	GO_BiologicalProcess-EBI-UniProt-GOA-ACAP-ARAP_08.05.2020_00h00	0,000677081	0,00264476	0,000744828	0,0014731	[5]	Group107	9,78	9,00
GO:0051219	phosphoprotein binding	GO_MolecularFunction-EBI-UniProt-GOA-ACAP-ARAP_08.05.2020_00h00	0,011026364	0,022075796	0,000753237	0,00147337	[3]	Group118	7,53	7,00
GO:0090559	regulation of membrane permeability	GO_BiologicalProcess-EBI-UniProt-GOA-ACAP-ARAP_08.05.2020_00h00	0,030644687	0,046923146	0,000753237	0,00147337	[3]	Group118	6,67	6,00
GO:0006839	mitochondrial transport	GO_BiologicalProcess-EBI-UniProt-GOA-ACAP-ARAP_08.05.2020_00h00	0,033457808	0,049951829	0,000753237	0,00147337	[4]	Group118	4,64	13,00
GO:1903829	positive regulation of cellular protein localization	GO_BiologicalProcess-EBI-UniProt-GOA-ACAP-ARAP_08.05.2020_00h00	0,025775954	0,04145813	0,000753237	0,00147337	[3, 4, 5, 6]	Group118	4,49	16,00
GO:0001836	release of cytochrome c from mitochondria	GO_BiologicalProcess-EBI-UniProt-GOA-ACAP-ARAP_08.05.2020_00h00	0,001802429	0,005618646	0,000753237	0,00147337	[4, 5, 6, 7]	Group118	10,45	7,00
GO:1903747	regulation of establishment of protein localization to mitochondrion	GO_BiologicalProcess-EBI-UniProt-GOA-ACAP-ARAP_08.05.2020_00h00	0,016363574	0,029491413	0,000753237	0,00147337	[5, 6, 7, 8]	Group118	7,69	6,00
GO:0042149	cellular response to glucose starvation	GO_BiologicalProcess-EBI-UniProt-GOA-ACAP-ARAP_08.05.2020_00h00	0,025549145	0,041231927	0,00079609	0,00150749	[5, 6, 7]	Group116	9,30	4,00

GO:0071287	cellular response to manganese ion	GO_BiologicalProcess-EBI-UniProt-GOA-ACAP-ARAP_08.05.2020_00h00	0,004184007	0,010319832	0,00079609	0,00150749	[6]	Group116	23,08	3,00
GO:0071320	cellular response to cAMP	GO_BiologicalProcess-EBI-UniProt-GOA-ACAP-ARAP_08.05.2020_00h00	0,003981134	0,009921734	0,00079609	0,00150749	[5, 6, 7]	Group116	10,34	6,00
GO:0060487	lung epithelial cell differentiation	GO_BiologicalProcess-EBI-UniProt-GOA-ACAP-ARAP_08.05.2020_00h00	0,018932438	0,03306267	0,00079609	0,00150749	[5, 6, 7, 8, 9]	Group116	13,64	3,00
GO:0043618	regulation of transcription from RNA polymerase II promoter in response to stress	GO_BiologicalProcess-EBI-UniProt-GOA-ACAP-ARAP_08.05.2020_00h00	0,016302135	0,029436117	0,00079609	0,00150749	[5, 7, 8, 9, 10, 11, 12]	Group116	6,45	8,00
GO:0036003	positive regulation of transcription from RNA polymerase II promoter in response to stress	GO_BiologicalProcess-EBI-UniProt-GOA-ACAP-ARAP_08.05.2020_00h00	0,008316356	0,017884837	0,00079609	0,00150749	[6, 7, 8, 9, 10, 11, 12, 13]	Group116	12,90	4,00
GO:2001020	regulation of response to DNA damage stimulus	GO_BiologicalProcess-EBI-UniProt-GOA-ACAP-ARAP_08.05.2020_00h00	0,004286675	0,010465174	0,000795422	0,00152242	[4, 5, 6]	Group138	5,86	14,00
GO:0051052	regulation of DNA metabolic process	GO_BiologicalProcess-EBI-UniProt-GOA-ACAP-ARAP_08.05.2020_00h00	0,002958667	0,008043876	0,000795422	0,00152242	[4, 5, 6, 7]	Group138	5,19	20,00
GO:0051053	negative regulation of DNA metabolic process	GO_BiologicalProcess-EBI-UniProt-GOA-ACAP-ARAP_08.05.2020_00h00	0,02470557	0,040278073	0,000795422	0,00152242	[4, 5, 6, 7, 8]	Group138	5,97	8,00
GO:0051054	positive regulation of DNA metabolic process	GO_BiologicalProcess-EBI-UniProt-GOA-ACAP-ARAP_08.05.2020_00h00	0,009825587	0,020265274	0,000795422	0,00152242	[4, 5, 6, 7, 8]	Group138	5,69	12,00
GO:0071897	DNA biosynthetic process	GO_BiologicalProcess-EBI-UniProt-GOA-ACAP-ARAP_08.05.2020_00h00	0,006491162	0,014651042	0,000795422	0,00152242	[5, 6, 7]	Group138	5,78	13,00
GO:2000278	regulation of DNA biosynthetic process	GO_BiologicalProcess-EBI-UniProt-GOA-ACAP-ARAP_08.05.2020_00h00	0,005610256	0,013063299	0,000795422	0,00152242	[5, 6, 7, 8]	Group138	7,20	9,00
GO:2000573	positive regulation of DNA biosynthetic process	GO_BiologicalProcess-EBI-UniProt-GOA-ACAP-ARAP_08.05.2020_00h00	0,004285873	0,010489975	0,000795422	0,00152242	[5, 6, 7, 8, 9]	Group138	8,97	7,00
GO:1904645	response to amyloid-beta	GO_BiologicalProcess-EBI-UniProt-GOA-ACAP-ARAP_08.05.2020_00h00	0,001862162	0,005748675	0,000791828	0,00153201	[5, 6]	Group068	12,00	6,00
GO:1904646	cellular response to amyloid-beta	GO_BiologicalProcess-EBI-UniProt-GOA-ACAP-ARAP_08.05.2020_00h00	0,018466409	0,032485943	0,000791828	0,00153201	[6, 7]	Group068	10,26	4,00
GO:0098815	modulation of excitatory postsynaptic potential	GO_BiologicalProcess-EBI-UniProt-GOA-ACAP-ARAP_08.05.2020_00h00	0,008146646	0,017638776	0,000791828	0,00153201	[4, 5, 6, 7, 8, 9, 10]	Group068	10,42	5,00
GO:0034976	response to endoplasmic reticulum stress	GO_BiologicalProcess-EBI-UniProt-GOA-ACAP-ARAP_08.05.2020_00h00	0,014044944	0,026354926	0,000886187	0,00166043	[4]	Group142	4,95	15,00
GO:0035967	cellular response to topologically incorrect protein	GO_BiologicalProcess-EBI-UniProt-GOA-ACAP-ARAP_08.05.2020_00h00	0,000578597	0,00233636	0,000886187	0,00166043	[4, 5]	Group142	7,60	13,00
GO:0030968	endoplasmic reticulum unfolded protein response	GO_BiologicalProcess-EBI-UniProt-GOA-ACAP-ARAP_08.05.2020_00h00	0,021922005	0,03687058	0,000886187	0,00166043	[3, 4, 5, 6, 7]	Group142	6,11	8,00
GO:1905897	regulation of response to endoplasmic reticulum stress	GO_BiologicalProcess-EBI-UniProt-GOA-ACAP-ARAP_08.05.2020_00h00	0,009845688	0,020263061	0,000886187	0,00166043	[4, 5, 6]	Group142	7,69	7,00
GO:0034620	cellular response to unfolded protein	GO_BiologicalProcess-EBI-UniProt-GOA-ACAP-ARAP_08.05.2020_00h00	0,006754607	0,015103176	0,000886187	0,00166043	[5, 6]	Group142	6,58	10,00
GO:0042149	cellular response to glucose starvation	GO_BiologicalProcess-EBI-UniProt-GOA-ACAP-ARAP_08.05.2020_00h00	0,025549145	0,041231927	0,000886187	0,00166043	[5, 6, 7]	Group142	9,30	4,00
GO:0070059	intrinsic apoptotic signaling pathway in response to endoplasmic reticulum stress	GO_BiologicalProcess-EBI-UniProt-GOA-ACAP-ARAP_08.05.2020_00h00	0,032554209	0,04921703	0,000886187	0,00166043	[5, 6, 7]	Group142	7,35	5,00

GO:2001242	regulation of intrinsic apoptotic signaling pathway	GO_BiologicalProcess-EBI-UniProt-GOA-ACAP-ARAP_08.05.2020_00h00	0,01703891	0,03036543	0,000886187	0,00166043	[5, 6, 7, 8]	Group142	5,71	10,00
GO:0001935	endothelial cell proliferation	GO_BiologicalProcess-EBI-UniProt-GOA-ACAP-ARAP_08.05.2020_00h00	0,003633375	0,00934715	0,000912621	0,00169215	[4]	Group129	6,79	11,00
GO:0090287	regulation of cellular response to growth factor stimulus	GO_BiologicalProcess-EBI-UniProt-GOA-ACAP-ARAP_08.05.2020_00h00	0,00110216	0,003767027	0,000912621	0,00169215	[3, 4, 6]	Group129	5,94	18,00
GO:0090288	negative regulation of cellular response to growth factor stimulus	GO_BiologicalProcess-EBI-UniProt-GOA-ACAP-ARAP_08.05.2020_00h00	0,012154936	0,023885572	0,000912621	0,00169215	[3, 4, 5, 6, 7]	Group129	6,02	10,00
GO:0001936	regulation of endothelial cell proliferation	GO_BiologicalProcess-EBI-UniProt-GOA-ACAP-ARAP_08.05.2020_00h00	0,014143549	0,026282284	0,000912621	0,00169215	[5, 6]	Group129	6,21	9,00
GO:0090092	regulation of transmembrane receptor protein serine/threonine kinase signaling pathway	GO_BiologicalProcess-EBI-UniProt-GOA-ACAP-ARAP_08.05.2020_00h00	0,018717377	0,032806829	0,000912621	0,00169215	[4, 5, 6, 7, 8]	Group129	5,04	13,00
GO:0040037	negative regulation of fibroblast growth factor receptor signaling pathway	GO_BiologicalProcess-EBI-UniProt-GOA-ACAP-ARAP_08.05.2020_00h00	0,012620698	0,024499003	0,000912621	0,00169215	[4, 5, 6, 7, 8, 9, 10]	Group129	15,79	3,00
GO:0003170	heart valve development	GO_BiologicalProcess-EBI-UniProt-GOA-ACAP-ARAP_08.05.2020_00h00	0,029100992	0,045283981	0,001004703	0,00184368	[3, 5, 6, 7]	Group080	7,58	5,00
GO:0002011	morphogenesis of an epithelial sheet	GO_BiologicalProcess-EBI-UniProt-GOA-ACAP-ARAP_08.05.2020_00h00	0,000160526	0,000882894	0,001004703	0,00184368	[5, 6]	Group080	13,33	8,00
GO:0044319	wound healing, spreading of cells	GO_BiologicalProcess-EBI-UniProt-GOA-ACAP-ARAP_08.05.2020_00h00	0,001784321	0,005598672	0,001004703	0,00184368	[4, 5, 6, 8, 9]	Group080	14,71	5,00
GO:0035313	wound healing, spreading of epidermal cells	GO_BiologicalProcess-EBI-UniProt-GOA-ACAP-ARAP_08.05.2020_00h00	0,009205425	0,019319279	0,001004703	0,00184368	[5, 6, 7, 9, 10]	Group080	17,65	3,00
GO:0005975	carbohydrate metabolic process	GO_BiologicalProcess-EBI-UniProt-GOA-ACAP-ARAP_08.05.2020_00h00	0,014086824	0,02627893	0,001067563	0,00193904	[3]	Group181	4,08	28,00
GO:0072593	reactive oxygen species metabolic process	GO_BiologicalProcess-EBI-UniProt-GOA-ACAP-ARAP_08.05.2020_00h00	0,031204019	0,047627185	0,001067563	0,00193904	[3]	Group181	4,58	14,00
GO:0044262	cellular carbohydrate metabolic process	GO_BiologicalProcess-EBI-UniProt-GOA-ACAP-ARAP_08.05.2020_00h00	0,000464017	0,001973621	0,001067563	0,00193904	[3, 4]	Group181	6,06	20,00
GO:0062012	regulation of small molecule metabolic process	GO_BiologicalProcess-EBI-UniProt-GOA-ACAP-ARAP_08.05.2020_00h00	0,027698154	0,043958761	0,001067563	0,00193904	[3, 4]	Group181	4,18	20,00
GO:0005976	polysaccharide metabolic process	GO_BiologicalProcess-EBI-UniProt-GOA-ACAP-ARAP_08.05.2020_00h00	0,00532592	0,012492415	0,001067563	0,00193904	[4]	Group181	7,26	9,00
GO:0006022	aminoglycan metabolic process	GO_BiologicalProcess-EBI-UniProt-GOA-ACAP-ARAP_08.05.2020_00h00	0,01962244	0,034081079	0,001067563	0,00193904	[4]	Group181	5,59	10,00
GO:0016051	carbohydrate biosynthetic process	GO_BiologicalProcess-EBI-UniProt-GOA-ACAP-ARAP_08.05.2020_00h00	0,029786352	0,046050951	0,001067563	0,00193904	[4]	Group181	4,86	12,00
GO:0062013	positive regulation of small molecule metabolic process	GO_BiologicalProcess-EBI-UniProt-GOA-ACAP-ARAP_08.05.2020_00h00	0,008775524	0,018580036	0,001067563	0,00193904	[3, 4, 5]	Group181	6,33	10,00
GO:0006109	regulation of carbohydrate metabolic process	GO_BiologicalProcess-EBI-UniProt-GOA-ACAP-ARAP_08.05.2020_00h00	0,006973192	0,015555582	0,001067563	0,00193904	[4, 5]	Group181	5,73	13,00
GO:0045913	positive regulation of carbohydrate metabolic process	GO_BiologicalProcess-EBI-UniProt-GOA-ACAP-ARAP_08.05.2020_00h00	0,002461149	0,007094337	0,001067563	0,00193904	[3, 4, 5, 6]	Group181	8,89	8,00
GO:0000271	polysaccharide biosynthetic process	GO_BiologicalProcess-EBI-UniProt-GOA-ACAP-ARAP_08.05.2020_00h00	0,007299894	0,016133945	0,001067563	0,00193904	[5]	Group181	8,14	7,00
GO:0043255	regulation of carbohydrate biosynthetic process	GO_BiologicalProcess-EBI-UniProt-GOA-ACAP-ARAP_08.05.2020_00h00	0,024403341	0,039853238	0,001067563	0,00193904	[4, 5, 6]	Group181	6,42	7,00

GO:0014909	smooth muscle cell migration	GO_BiologicalProcess-EBI-UniProt-GOA-ACAP-ARAP_08.05.2020_00h00	0,000144714	0,000819473	0,001067563	0,00193904	[5, 6]	Group181	12,00	9,00
GO:0044070	regulation of anion transport	GO_BiologicalProcess-EBI-UniProt-GOA-ACAP-ARAP_08.05.2020_00h00	0,032775592	0,049240567	0,001067563	0,00193904	[5, 6]	Group181	6,03	7,00
GO:0006140	regulation of nucleotide metabolic process	GO_BiologicalProcess-EBI-UniProt-GOA-ACAP-ARAP_08.05.2020_00h00	0,023751667	0,039055575	0,001067563	0,00193904	[4, 5, 6, 7, 8]	Group181	6,02	8,00
GO:0014910	regulation of smooth muscle cell migration	GO_BiologicalProcess-EBI-UniProt-GOA-ACAP-ARAP_08.05.2020_00h00	0,000348865	0,001605114	0,001067563	0,00193904	[5, 6, 7]	Group181	11,94	8,00
GO:0032885	regulation of polysaccharide biosynthetic process	GO_BiologicalProcess-EBI-UniProt-GOA-ACAP-ARAP_08.05.2020_00h00	0,023646265	0,038949184	0,001067563	0,00193904	[5, 6, 7]	Group181	9,52	4,00
GO:0051546	keratinocyte migration	GO_BiologicalProcess-EBI-UniProt-GOA-ACAP-ARAP_08.05.2020_00h00	0,012620698	0,024499003	0,001067563	0,00193904	[5, 6, 7]	Group181	15,79	3,00
GO:0014911	positive regulation of smooth muscle cell migration	GO_BiologicalProcess-EBI-UniProt-GOA-ACAP-ARAP_08.05.2020_00h00	0,000943803	0,003345259	0,001067563	0,00193904	[5, 6, 7, 8]	Group181	13,64	6,00
GO:0030212	hyaluronan metabolic process	GO_BiologicalProcess-EBI-UniProt-GOA-ACAP-ARAP_08.05.2020_00h00	0,005625159	0,013066206	0,001067563	0,00193904	[6, 7]	Group181	11,36	5,00
GO:0051549	positive regulation of keratinocyte migration	GO_BiologicalProcess-EBI-UniProt-GOA-ACAP-ARAP_08.05.2020_00h00	0,003281716	0,008604389	0,001067563	0,00193904	[4, 5, 6, 7, 8, 9]	Group181	25,00	3,00
GO:0009179	purine ribonucleoside diphosphate metabolic process	GO_BiologicalProcess-EBI-UniProt-GOA-ACAP-ARAP_08.05.2020_00h00	0,007714132	0,01689342	0,001067563	0,00193904	[7, 8, 9]	Group181	6,45	10,00
GO:0019751	polyol metabolic process	GO_BiologicalProcess-EBI-UniProt-GOA-ACAP-ARAP_08.05.2020_00h00	0,006168251	0,014088344	0,001366218	0,00245643	[4, 5]	Group065	6,67	10,00
GO:0071545	inositol phosphate catabolic process	GO_BiologicalProcess-EBI-UniProt-GOA-ACAP-ARAP_08.05.2020_00h00	0,001606011	0,005140308	0,001366218	0,00245643	[5, 6, 7]	Group065	20,00	4,00
GO:0046856	phosphatidylinositol dephosphorylation	GO_BiologicalProcess-EBI-UniProt-GOA-ACAP-ARAP_08.05.2020_00h00	0,007393209	0,016265061	0,001366218	0,00245643	[6, 7, 8]	Group065	13,33	4,00
GO:0098751	bone cell development	GO_BiologicalProcess-EBI-UniProt-GOA-ACAP-ARAP_08.05.2020_00h00	0,014057252	0,026326399	0,001366218	0,00245643	[4, 5, 6, 7]	Group066	11,11	4,00
GO:0061515	myeloid cell development	GO_BiologicalProcess-EBI-UniProt-GOA-ACAP-ARAP_08.05.2020_00h00	0,000976749	0,003436575	0,001366218	0,00245643	[4, 5, 6, 7, 8, 9]	Group066	10,26	8,00
GO:0030316	osteoclast differentiation	GO_BiologicalProcess-EBI-UniProt-GOA-ACAP-ARAP_08.05.2020_00h00	0,02034726	0,034772012	0,001366218	0,00245643	[6, 7, 8, 9, 10]	Group066	6,67	7,00
GO:0006914	autophagy	GO_BiologicalProcess-EBI-UniProt-GOA-ACAP-ARAP_08.05.2020_00h00	0,030111073	0,046254087	0,001519263	0,00270429	[3, 4]	Group090	4,04	22,00
GO:0009894	regulation of catabolic process	GO_BiologicalProcess-EBI-UniProt-GOA-ACAP-ARAP_08.05.2020_00h00	0,002341402	0,006810703	0,001519263	0,00270429	[3, 4]	Group090	4,08	44,00
GO:0016236	macroautophagy	GO_BiologicalProcess-EBI-UniProt-GOA-ACAP-ARAP_08.05.2020_00h00	0,033472851	0,049896449	0,001519263	0,00270429	[4, 5]	Group090	4,53	14,00
GO:0031329	regulation of cellular catabolic process	GO_BiologicalProcess-EBI-UniProt-GOA-ACAP-ARAP_08.05.2020_00h00	0,00275227	0,007656751	0,001519263	0,00270429	[4, 5]	Group090	4,21	38,00
GO:0006458	'de novo' protein folding	GO_BiologicalProcess-EBI-UniProt-GOA-ACAP-ARAP_08.05.2020_00h00	0,004597845	0,011111458	0,001563882	0,00275615	[3]	Group134	11,90	5,00
GO:0061077	chaperone-mediated protein folding	GO_BiologicalProcess-EBI-UniProt-GOA-ACAP-ARAP_08.05.2020_00h00	0,027465085	0,04366127	0,001563882	0,00275615	[3]	Group134	7,69	5,00
GO:0003756	protein disulfide isomerase activity	GO_MolecularFunction-EBI-UniProt-GOA-ACAP-ARAP_08.05.2020_00h00	0,012620698	0,024499003	0,001563882	0,00275615	[3, 5]	Group134	15,79	3,00

GO:0022417	protein maturation by protein folding	GO_BiologicalProcess-EBI-UniProt-GOA-ACAP-ARAP_08.05.2020_00h00	0,00186122	0,005764362	0,001563882	0,00275615	[3, 5, 6]	Group134	30,00	3,00
GO:0015036	disulfide oxidoreductase activity	GO_BiologicalProcess-EBI-UniProt-GOA-ACAP-ARAP_08.05.2020_00h00	0,000560477	0,002292205	0,001563882	0,00275615	[5]	Group134	15,00	6,00
GO:0015035	protein disulfide oxidoreductase activity	GO_BiologicalProcess-EBI-UniProt-GOA-ACAP-ARAP_08.05.2020_00h00	0,002326542	0,006788111	0,001563882	0,00275615	[6]	Group134	18,18	4,00
GO:0015037	peptide disulfide oxidoreductase activity	GO_BiologicalProcess-EBI-UniProt-GOA-ACAP-ARAP_08.05.2020_00h00	0,005222608	0,012340829	0,001563882	0,00275615	[6]	Group134	21,43	3,00
GO:0007219	Notch signaling pathway	GO_BiologicalProcess-EBI-UniProt-GOA-ACAP-ARAP_08.05.2020_00h00	0,002203569	0,006549115	0,001884013	0,00325587	[4, 5, 6]	Group135	6,57	13,00
GO:0008366	axon ensheathment	GO_BiologicalProcess-EBI-UniProt-GOA-ACAP-ARAP_08.05.2020_00h00	0,020160235	0,034637965	0,001884013	0,00325587	[3, 6, 7]	Group135	5,84	9,00
GO:0042063	gliogenesis	GO_BiologicalProcess-EBI-UniProt-GOA-ACAP-ARAP_08.05.2020_00h00	0,010466523	0,021221317	0,001884013	0,00325587	[5, 6, 7]	Group135	5,00	16,00
GO:0010001	glial cell differentiation	GO_BiologicalProcess-EBI-UniProt-GOA-ACAP-ARAP_08.05.2020_00h00	0,023210056	0,038495708	0,001884013	0,00325587	[4, 6, 7, 8]	Group135	5,04	12,00
GO:0021782	glial cell development	GO_BiologicalProcess-EBI-UniProt-GOA-ACAP-ARAP_08.05.2020_00h00	0,015593992	0,028264109	0,001884013	0,00325587	[4, 5, 7, 8, 9]	Group135	6,50	8,00
GO:0048708	astrocyte differentiation	GO_BiologicalProcess-EBI-UniProt-GOA-ACAP-ARAP_08.05.2020_00h00	0,027841724	0,043967873	0,001884013	0,00325587	[5, 6, 7, 8, 9]	Group135	6,82	6,00
GO:0014002	astrocyte development	GO_BiologicalProcess-EBI-UniProt-GOA-ACAP-ARAP_08.05.2020_00h00	0,031797417	0,04830179	0,001884013	0,00325587	[5, 6, 7, 8, 9, 10]	Group135	8,70	4,00
GO:0031668	cellular response to extracellular stimulus	GO_BiologicalProcess-EBI-UniProt-GOA-ACAP-ARAP_08.05.2020_00h00	0,019883081	0,034471214	0,001872875	0,00326835	[3, 4]	Group121	4,86	14,00
GO:0009267	cellular response to starvation	GO_BiologicalProcess-EBI-UniProt-GOA-ACAP-ARAP_08.05.2020_00h00	0,026896063	0,042899221	0,001872875	0,00326835	[4, 5, 6]	Group121	5,56	9,00
GO:0042149	cellular response to glucose starvation	GO_BiologicalProcess-EBI-UniProt-GOA-ACAP-ARAP_08.05.2020_00h00	0,025549145	0,041231927	0,001872875	0,00326835	[5, 6, 7]	Group121	9,30	4,00
GO:0070059	intrinsic apoptotic signaling pathway in response to endoplasmic reticulum stress	GO_BiologicalProcess-EBI-UniProt-GOA-ACAP-ARAP_08.05.2020_00h00	0,032554209	0,04921703	0,001872875	0,00326835	[5, 6, 7]	Group121	7,35	5,00
GO:0071287	cellular response to manganese ion	GO_BiologicalProcess-EBI-UniProt-GOA-ACAP-ARAP_08.05.2020_00h00	0,004184007	0,010319832	0,001872875	0,00326835	[6]	Group121	23,08	3,00
GO:0036003	positive regulation of transcription from RNA polymerase II promoter in response to stress	GO_BiologicalProcess-EBI-UniProt-GOA-ACAP-ARAP_08.05.2020_00h00	0,008316356	0,017884837	0,001872875	0,00326835	[6, 7, 8, 9, 10, 11, 12, 13]	Group121	12,90	4,00
GO:0019900	kinase binding	GO_MolecularFunction-EBI-UniProt-GOA-ACAP-ARAP_08.05.2020_00h00	0,00272406	0,007600365	0,001970044	0,00337181	[4]	Group075	4,35	34,00
GO:0019901	protein kinase binding	GO_MolecularFunction-EBI-UniProt-GOA-ACAP-ARAP_08.05.2020_00h00	0,00820952	0,017734786	0,001970044	0,00337181	[5]	Group075	4,22	29,00
GO:1990782	protein tyrosine kinase binding	GO_MolecularFunction-EBI-UniProt-GOA-ACAP-ARAP_08.05.2020_00h00	0,004437468	0,010778317	0,001970044	0,00337181	[6]	Group075	8,08	8,00
GO:0042326	negative regulation of phosphorylation	GO_BiologicalProcess-EBI-UniProt-GOA-ACAP-ARAP_08.05.2020_00h00	0,001627748	0,005192518	0,001970044	0,00337181	[6, 7, 8]	Group075	5,07	24,00
GO:0051651	maintenance of location in cell	GO_BiologicalProcess-EBI-UniProt-GOA-ACAP-ARAP_08.05.2020_00h00	0,00192218	0,005914877	0,002095667	0,00351914	[2, 3, 4]	Group147	6,17	15,00

GO:0019751	polyol metabolic process	GO_BiologicalProcess-EBI-UniProt-GOA-ACAP-ARAP_08.05.2020_00h00	0,006168251	0,014088344	0,002095667	0,00351914	[4, 5]	Group147	6,67	10,00
GO:0046165	alcohol biosynthetic process	GO_BiologicalProcess-EBI-UniProt-GOA-ACAP-ARAP_08.05.2020_00h00	0,029087059	0,045336019	0,002095667	0,00351914	[4, 5]	Group147	5,24	10,00
GO:0046173	polyol biosynthetic process	GO_BiologicalProcess-EBI-UniProt-GOA-ACAP-ARAP_08.05.2020_00h00	0,00929126	0,019456752	0,002095667	0,00351914	[5, 6]	Group147	7,78	7,00
GO:0051208	sequestering of calcium ion	GO_BiologicalProcess-EBI-UniProt-GOA-ACAP-ARAP_08.05.2020_00h00	0,000410002	0,001824987	0,002095667	0,00351914	[3, 4, 5, 8, 10]	Group147	8,33	12,00
GO:0071545	inositol phosphate catabolic process	GO_BiologicalProcess-EBI-UniProt-GOA-ACAP-ARAP_08.05.2020_00h00	0,001606011	0,005140308	0,002095667	0,00351914	[5, 6, 7]	Group147	20,00	4,00
GO:0032959	inositol trisphosphate biosynthetic process	GO_BiologicalProcess-EBI-UniProt-GOA-ACAP-ARAP_08.05.2020_00h00	0,026696119	0,042651396	0,002095667	0,00351914	[6, 7, 8]	Group147	12,00	3,00
GO:0060402	calcium ion transport into cytosol	GO_BiologicalProcess-EBI-UniProt-GOA-ACAP-ARAP_08.05.2020_00h00	0,000377397	0,001703626	0,002095667	0,00351914	[4, 10, 12]	Group147	7,57	14,00
GO:0051209	release of sequestered calcium ion into cytosol	GO_BiologicalProcess-EBI-UniProt-GOA-ACAP-ARAP_08.05.2020_00h00	0,0002773	0,001333548	0,002095667	0,00351914	[4, 5, 6, 7, 8, 10, 11, 12, 13, 14]	Group147	8,70	12,00
GO:0048608	reproductive structure development	GO_BiologicalProcess-EBI-UniProt-GOA-ACAP-ARAP_08.05.2020_00h00	0,00208441	0,006292682	0,00208441	0,00353357	[3, 4, 5, 6]	Group076	4,98	24,00
GO:0001890	placenta development	GO_BiologicalProcess-EBI-UniProt-GOA-ACAP-ARAP_08.05.2020_00h00	0,00194322	0,005960454	0,00208441	0,00353357	[4, 5, 6, 7]	Group076	6,98	12,00
GO:0001892	embryonic placenta development	GO_BiologicalProcess-EBI-UniProt-GOA-ACAP-ARAP_08.05.2020_00h00	0,017631847	0,031363714	0,00208441	0,00353357	[3, 4, 5, 6, 7, 8]	Group076	6,86	7,00
GO:0060674	placenta blood vessel development	GO_BiologicalProcess-EBI-UniProt-GOA-ACAP-ARAP_08.05.2020_00h00	0,003317585	0,008674669	0,00208441	0,00353357	[3, 4, 5, 6, 7, 8, 9]	Group076	12,82	5,00
GO:0098751	bone cell development	GO_BiologicalProcess-EBI-UniProt-GOA-ACAP-ARAP_08.05.2020_00h00	0,014057252	0,026326399	0,002186506	0,00363737	[4, 5, 6, 7]	Group094	11,11	4,00
GO:0002521	leukocyte differentiation	GO_BiologicalProcess-EBI-UniProt-GOA-ACAP-ARAP_08.05.2020_00h00	0,00341803	0,008912955	0,002186506	0,00363737	[4, 5, 6, 7, 8]	Group094	4,66	26,00
GO:0002573	myeloid leukocyte differentiation	GO_BiologicalProcess-EBI-UniProt-GOA-ACAP-ARAP_08.05.2020_00h00	0,002206373	0,00653715	0,002186506	0,00363737	[5, 6, 7, 8, 9]	Group094	6,31	14,00
GO:0030316	osteoclast differentiation	GO_BiologicalProcess-EBI-UniProt-GOA-ACAP-ARAP_08.05.2020_00h00	0,02034726	0,034772012	0,002186506	0,00363737	[6, 7, 8, 9, 10]	Group094	6,67	7,00
GO:0051250	negative regulation of lymphocyte activation	GO_BiologicalProcess-EBI-UniProt-GOA-ACAP-ARAP_08.05.2020_00h00	0,02252461	0,037685405	0,002327856	0,00380145	[4, 5, 6, 7]	Group105	5,73	9,00
GO:0048041	focal adhesion assembly	GO_BiologicalProcess-EBI-UniProt-GOA-ACAP-ARAP_08.05.2020_00h00	0,00302181	0,00814612	0,002327856	0,00380145	[5, 7]	Group105	8,60	8,00
GO:1903038	negative regulation of leukocyte cell-cell adhesion	GO_BiologicalProcess-EBI-UniProt-GOA-ACAP-ARAP_08.05.2020_00h00	0,023751667	0,039055575	0,002327856	0,00380145	[5, 6, 7]	Group105	6,02	8,00
GO:0050860	negative regulation of T cell receptor signaling pathway	GO_BiologicalProcess-EBI-UniProt-GOA-ACAP-ARAP_08.05.2020_00h00	0,00033793	0,001562316	0,002327856	0,00380145	[4, 5, 6, 7, 8, 9, 10, 11, 12]	Group105	20,83	5,00
GO:0004725	protein tyrosine phosphatase activity	GO_BiologicalProcess-EBI-UniProt-GOA-ACAP-ARAP_08.05.2020_00h00	0,032775592	0,049240567	0,002327856	0,00380145	[8, 9]	Group105	6,03	7,00
GO:0003013	circulatory system process	GO_BiologicalProcess-EBI-UniProt-GOA-ACAP-ARAP_08.05.2020_00h00	0,002791696	0,007699287	0,002315196	0,00381579	[3]	Group146	4,51	30,00
GO:0008015	blood circulation	GO_BiologicalProcess-EBI-UniProt-GOA-ACAP-ARAP_08.05.2020_00h00	0,00145709	0,004759166	0,002315196	0,00381579	[4]	Group146	4,83	28,00

GO:0008217	regulation of blood pressure	GO_BiologicalProcess-EBI-UniProt-GOA-ACAP-ARAP_08.05.2020_00h00	0,019399151	0,033754524	0,002315196	0,00381579	[3, 5]	Group146	5,37	11,00
GO:0098801	regulation of renal system process	GO_BiologicalProcess-EBI-UniProt-GOA-ACAP-ARAP_08.05.2020_00h00	0,003317585	0,008674669	0,002315196	0,00381579	[4, 5]	Group146	12,82	5,00
GO:0003073	regulation of systemic arterial blood pressure	GO_BiologicalProcess-EBI-UniProt-GOA-ACAP-ARAP_08.05.2020_00h00	0,002397156	0,006951752	0,002315196	0,00381579	[4, 6]	Group146	8,18	9,00
GO:0003094	glomerular filtration	GO_BiologicalProcess-EBI-UniProt-GOA-ACAP-ARAP_08.05.2020_00h00	0,026696119	0,042651396	0,002315196	0,00381579	[5]	Group146	12,00	3,00
GO:0003093	regulation of glomerular filtration	GO_BiologicalProcess-EBI-UniProt-GOA-ACAP-ARAP_08.05.2020_00h00	0,003281716	0,008604389	0,002315196	0,00381579	[5, 6, 7, 9]	Group146	25,00	3,00
GO:0090280	positive regulation of calcium ion import	GO_BiologicalProcess-EBI-UniProt-GOA-ACAP-ARAP_08.05.2020_00h00	0,016667997	0,029927343	0,002315196	0,00381579	[5, 6, 7, 8, 9, 10, 11]	Group146	14,29	3,00
GO:0043027	cysteine-type endopeptidase inhibitor activity involved in apoptotic process	GO_BiologicalProcess-EBI-UniProt-GOA-ACAP-ARAP_08.05.2020_00h00	0,032678593	0,04924947	0,002315196	0,00381579	[5, 6, 7, 8, 9, 10, 11, 12, 13]	Group146	11,11	3,00
GO:0046677	response to antibiotic	GO_BiologicalProcess-EBI-UniProt-GOA-ACAP-ARAP_08.05.2020_00h00	0,003973101	0,009927566	0,002769187	0,00448105	[3]	Group143	5,16	19,00
GO:0071216	cellular response to biotic stimulus	GO_BiologicalProcess-EBI-UniProt-GOA-ACAP-ARAP_08.05.2020_00h00	0,008525003	0,018251516	0,002769187	0,00448105	[3]	Group143	5,41	14,00
GO:0071236	cellular response to antibiotic	GO_BiologicalProcess-EBI-UniProt-GOA-ACAP-ARAP_08.05.2020_00h00	0,011685916	0,023058603	0,002769187	0,00448105	[4]	Group143	6,06	10,00
GO:0042542	response to hydrogen peroxide	GO_BiologicalProcess-EBI-UniProt-GOA-ACAP-ARAP_08.05.2020_00h00	0,02252461	0,037685405	0,002769187	0,00448105	[4, 5]	Group143	5,73	9,00
GO:0002237	response to molecule of bacterial origin	GO_BiologicalProcess-EBI-UniProt-GOA-ACAP-ARAP_08.05.2020_00h00	0,027832035	0,044098109	0,002769187	0,00448105	[4, 6]	Group143	4,37	17,00
GO:0071347	cellular response to interleukin-1	GO_BiologicalProcess-EBI-UniProt-GOA-ACAP-ARAP_08.05.2020_00h00	0,000117157	0,000700744	0,002769187	0,00448105	[6]	Group143	8,06	15,00
GO:1903556	negative regulation of tumor necrosis factor superfamily cytokine production	GO_BiologicalProcess-EBI-UniProt-GOA-ACAP-ARAP_08.05.2020_00h00	0,029100992	0,045283981	0,002769187	0,00448105	[4, 5, 6, 7, 8]	Group143	7,58	5,00
GO:0071222	cellular response to lipopolysaccharide	GO_BiologicalProcess-EBI-UniProt-GOA-ACAP-ARAP_08.05.2020_00h00	0,006491162	0,014651042	0,002769187	0,00448105	[5, 6, 8]	Group143	5,78	13,00
GO:0070997	neuron death	GO_BiologicalProcess-EBI-UniProt-GOA-ACAP-ARAP_08.05.2020_00h00	0,002958667	0,008043876	0,002958667	0,00470217	[3]	Group050	5,19	20,00
GO:1901214	regulation of neuron death	GO_BiologicalProcess-EBI-UniProt-GOA-ACAP-ARAP_08.05.2020_00h00	0,01810614	0,031969696	0,002958667	0,00470217	[4, 5]	Group050	4,69	16,00
GO:0008536	Ran GTPase binding	GO_MolecularFunction-EBI-UniProt-GOA-ACAP-ARAP_08.05.2020_00h00	0,002954694	0,008055959	0,002954694	0,00473816	[7]	Group018	13,16	5,00
GO:0007565	female pregnancy	GO_BiologicalProcess-EBI-UniProt-GOA-ACAP-ARAP_08.05.2020_00h00	0,002029531	0,006205307	0,003226102	0,00508182	[3, 4]	Group058	6,36	14,00
GO:0007566	embryo implantation	GO_BiologicalProcess-EBI-UniProt-GOA-ACAP-ARAP_08.05.2020_00h00	0,000936067	0,003342597	0,003226102	0,00508182	[2, 3, 4, 5]	Group058	11,67	7,00
GO:0033273	response to vitamin	GO_BiologicalProcess-EBI-UniProt-GOA-ACAP-ARAP_08.05.2020_00h00	0,023341313	0,038579684	0,003226102	0,00508182	[4, 6]	Group058	6,48	7,00
GO:0002521	leukocyte differentiation	GO_BiologicalProcess-EBI-UniProt-GOA-ACAP-ARAP_08.05.2020_00h00	0,00341803	0,008912955	0,00341803	0,00529052	[4, 5, 6, 7, 8]	Group092	4,66	26,00
GO:0030098	lymphocyte differentiation	GO_BiologicalProcess-EBI-UniProt-GOA-ACAP-ARAP_08.05.2020_00h00	0,005579029	0,013022271	0,00341803	0,00529052	[4, 5, 6, 7, 8, 9]	Group092	5,00	19,00
GO:0030183	B cell differentiation	GO_BiologicalProcess-EBI-UniProt-GOA-ACAP-ARAP_08.05.2020_00h00	0,029878801	0,04604511	0,00341803	0,00529052	[5, 6, 7, 8, 9, 10]	Group092	5,76	8,00

GO:0002335	mature B cell differentiation	GO_BiologicalProcess-EBI-UniProt-GOA-ACAP-ARAP_08.05.2020_00h00	0,029607117	0,045996774	0,00341803	0,00529052	[6, 7, 8, 9, 10, 11]	Group092	11,54	3,00
GO:0042578	phosphoric ester hydrolase activity	GO_MolecularFunction-EBI-UniProt-GOA-ACAP-ARAP_08.05.2020_00h00	0,01470738	0,026911974	0,003392755	0,00529746	[4]	Group095	4,14	26,00
GO:0035303	regulation of dephosphorylation	GO_BiologicalProcess-EBI-UniProt-GOA-ACAP-ARAP_08.05.2020_00h00	0,033220664	0,04975301	0,003392755	0,00529746	[6, 7]	Group095	4,93	11,00
GO:0035306	positive regulation of dephosphorylation	GO_BiologicalProcess-EBI-UniProt-GOA-ACAP-ARAP_08.05.2020_00h00	0,006470072	0,014637965	0,003392755	0,00529746	[6, 7, 8]	Group095	9,38	6,00
GO:0050860	negative regulation of T cell receptor signaling pathway	GO_BiologicalProcess-EBI-UniProt-GOA-ACAP-ARAP_08.05.2020_00h00	0,00033793	0,001562316	0,003392755	0,00529746	[4, 5, 6, 7, 8, 9, 10, 11, 12]	Group095	20,83	5,00
GO:0004725	protein tyrosine phosphatase activity	GO_BiologicalProcess-EBI-UniProt-GOA-ACAP-ARAP_08.05.2020_00h00	0,032775592	0,049240567	0,003392755	0,00529746	[8, 9]	Group095	6,03	7,00
GO:0015931	nucleobase-containing compound transport	GO_BiologicalProcess-EBI-UniProt-GOA-ACAP-ARAP_08.05.2020_00h00	0,032646921	0,04927934	0,004215118	0,00646803	[5]	Group110	4,66	13,00
GO:0015932	nucleobase-containing compound transmembrane transporter activity	GO_BiologicalProcess-EBI-UniProt-GOA-ACAP-ARAP_08.05.2020_00h00	0,017647753	0,031333763	0,004215118	0,00646803	[4, 6]	Group110	8,62	5,00
GO:1901505	carbohydrate derivative transmembrane transporter activity	GO_BiologicalProcess-EBI-UniProt-GOA-ACAP-ARAP_08.05.2020_00h00	0,021512173	0,036308907	0,004215118	0,00646803	[4, 6]	Group110	8,20	5,00
GO:0006862	nucleotide transport	GO_BiologicalProcess-EBI-UniProt-GOA-ACAP-ARAP_08.05.2020_00h00	0,023646265	0,038949184	0,004215118	0,00646803	[6]	Group110	9,52	4,00
GO:0006611	protein export from nucleus	GO_BiologicalProcess-EBI-UniProt-GOA-ACAP-ARAP_08.05.2020_00h00	0,030000042	0,046157621	0,004215118	0,00646803	[5, 6, 7, 8, 9]	Group110	5,21	10,00
GO:0006493	protein O-linked glycosylation	GO_BiologicalProcess-EBI-UniProt-GOA-ACAP-ARAP_08.05.2020_00h00	0,004298435	0,010467182	0,004298435	0,0065395	[5, 7, 8]	Group055	7,50	9,00
GO:0016266	O-glycan processing	GO_BiologicalProcess-EBI-UniProt-GOA-ACAP-ARAP_08.05.2020_00h00	0,029100992	0,045283981	0,004298435	0,0065395	[6, 8, 9]	Group055	7,58	5,00
GO:0005976	polysaccharide metabolic process	GO_BiologicalProcess-EBI-UniProt-GOA-ACAP-ARAP_08.05.2020_00h00	0,00532592	0,012492415	0,004358792	0,00657513	[4]	Group089	7,26	9,00
GO:0006022	aminoglycan metabolic process	GO_BiologicalProcess-EBI-UniProt-GOA-ACAP-ARAP_08.05.2020_00h00	0,01962244	0,034081079	0,004358792	0,00657513	[4]	Group089	5,59	10,00
GO:0000271	polysaccharide biosynthetic process	GO_BiologicalProcess-EBI-UniProt-GOA-ACAP-ARAP_08.05.2020_00h00	0,007299894	0,016133945	0,004358792	0,00657513	[5]	Group089	8,14	7,00
GO:0030212	hyaluronan metabolic process	GO_BiologicalProcess-EBI-UniProt-GOA-ACAP-ARAP_08.05.2020_00h00	0,005625159	0,013066206	0,004358792	0,00657513	[6, 7]	Group089	11,36	5,00
GO:0062012	regulation of small molecule metabolic process	GO_BiologicalProcess-EBI-UniProt-GOA-ACAP-ARAP_08.05.2020_00h00	0,027698154	0,043958761	0,004643952	0,00694642	[3, 4]	Group155	4,18	20,00
GO:0055086	nucleobase-containing small molecule metabolic process	GO_BiologicalProcess-EBI-UniProt-GOA-ACAP-ARAP_08.05.2020_00h00	0,003933324	0,009853902	0,004643952	0,00694642	[3, 4, 5]	Group155	4,41	30,00
GO:0062013	positive regulation of small molecule metabolic process	GO_BiologicalProcess-EBI-UniProt-GOA-ACAP-ARAP_08.05.2020_00h00	0,008775524	0,018580036	0,004643952	0,00694642	[3, 4, 5]	Group155	6,33	10,00
GO:0006109	regulation of carbohydrate metabolic process	GO_BiologicalProcess-EBI-UniProt-GOA-ACAP-ARAP_08.05.2020_00h00	0,006973192	0,015555582	0,004643952	0,00694642	[4, 5]	Group155	5,73	13,00
GO:0006140	regulation of nucleotide metabolic process	GO_BiologicalProcess-EBI-UniProt-GOA-ACAP-ARAP_08.05.2020_00h00	0,023751667	0,039055575	0,004643952	0,00694642	[4, 5, 6, 7, 8]	Group155	6,02	8,00
GO:0009117	nucleotide metabolic process	GO_BiologicalProcess-EBI-UniProt-GOA-ACAP-ARAP_08.05.2020_00h00	0,022016035	0,036963765	0,004643952	0,00694642	[5, 6, 7]	Group155	4,07	24,00

GO:0045980	negative regulation of nucleotide metabolic process	GO_BiologicalProcess-EBI-UniProt-GOA-ACAP-ARAP_08.05.2020_00h00	0,021358484	0,036113199	0,004643952	0,00694642	[4, 5, 6, 7, 8, 9]	Group155	13,04	3,00
GO:0009167	purine ribonucleoside monophosphate metabolic process	GO_BiologicalProcess-EBI-UniProt-GOA-ACAP-ARAP_08.05.2020_00h00	0,020105848	0,034731582	0,004643952	0,00694642	[7, 8, 9]	Group155	10,00	4,00
GO:0009179	purine ribonucleoside diphosphate metabolic process	GO_BiologicalProcess-EBI-UniProt-GOA-ACAP-ARAP_08.05.2020_00h00	0,007714132	0,01689342	0,004643952	0,00694642	[7, 8, 9]	Group155	6,45	10,00
GO:2001169	regulation of ATP biosynthetic process	GO_BiologicalProcess-EBI-UniProt-GOA-ACAP-ARAP_08.05.2020_00h00	0,014564467	0,026855865	0,004643952	0,00694642	[4, 5, 7, 8, 9, 10, 11, 12]	Group155	15,00	3,00
GO:0045992	negative regulation of embryonic development	GO_BiologicalProcess-EBI-UniProt-GOA-ACAP-ARAP_08.05.2020_00h00	0,032678593	0,04924947	0,004808458	0,00713255	[3, 4, 5, 6]	Group096	11,11	3,00
GO:0007219	Notch signaling pathway	GO_BiologicalProcess-EBI-UniProt-GOA-ACAP-ARAP_08.05.2020_00h00	0,002203569	0,006549115	0,004808458	0,00713255	[4, 5, 6]	Group096	6,57	13,00
GO:0008593	regulation of Notch signaling pathway	GO_BiologicalProcess-EBI-UniProt-GOA-ACAP-ARAP_08.05.2020_00h00	0,007866058	0,017108677	0,004808458	0,00713255	[4, 5, 6, 7]	Group096	7,34	8,00
GO:0045746	negative regulation of Notch signaling pathway	GO_BiologicalProcess-EBI-UniProt-GOA-ACAP-ARAP_08.05.2020_00h00	0,004597845	0,011111458	0,004808458	0,00713255	[4, 5, 6, 7, 8]	Group096	11,90	5,00
GO:0048708	astrocyte differentiation	GO_BiologicalProcess-EBI-UniProt-GOA-ACAP-ARAP_08.05.2020_00h00	0,027841724	0,043967873	0,004808458	0,00713255	[5, 6, 7, 8, 9]	Group096	6,82	6,00
GO:0006022	aminoglycan metabolic process	GO_BiologicalProcess-EBI-UniProt-GOA-ACAP-ARAP_08.05.2020_00h00	0,01962244	0,034081079	0,005321251	0,00776379	[4]	Group093	5,59	10,00
GO:0032885	regulation of polysaccharide biosynthetic process	GO_BiologicalProcess-EBI-UniProt-GOA-ACAP-ARAP_08.05.2020_00h00	0,023646265	0,038949184	0,005321251	0,00776379	[5, 6, 7]	Group093	9,52	4,00
GO:1904707	positive regulation of vascular smooth muscle cell proliferation	GO_BiologicalProcess-EBI-UniProt-GOA-ACAP-ARAP_08.05.2020_00h00	0,015443421	0,028044315	0,005321251	0,00776379	[5, 6, 7]	Group093	10,81	4,00
GO:0030212	hyaluronan metabolic process	GO_BiologicalProcess-EBI-UniProt-GOA-ACAP-ARAP_08.05.2020_00h00	0,005625159	0,013066206	0,005321251	0,00776379	[6, 7]	Group093	11,36	5,00
GO:0050764	regulation of phagocytosis	GO_BiologicalProcess-EBI-UniProt-GOA-ACAP-ARAP_08.05.2020_00h00	0,005313152	0,012523859	0,005313152	0,00781604	[4, 5, 6]	Group021	7,84	8,00
GO:0050918	positive chemotaxis	GO_BiologicalProcess-EBI-UniProt-GOA-ACAP-ARAP_08.05.2020_00h00	0,005643533	0,013077145	0,005643533	0,0081012	[4, 5]	Group023	8,54	7,00
GO:0048592	eye morphogenesis	GO_BiologicalProcess-EBI-UniProt-GOA-ACAP-ARAP_08.05.2020_00h00	0,027837381	0,044033676	0,005636813	0,00815734	[5, 6, 7, 8]	Group103	5,52	9,00
GO:0002088	lens development in camera-type eye	GO_BiologicalProcess-EBI-UniProt-GOA-ACAP-ARAP_08.05.2020_00h00	0,009845688	0,020263061	0,005636813	0,00815734	[3, 7, 8, 9]	Group103	7,69	7,00
GO:0002089	lens morphogenesis in camera-type eye	GO_BiologicalProcess-EBI-UniProt-GOA-ACAP-ARAP_08.05.2020_00h00	0,018932438	0,03306267	0,005636813	0,00815734	[3, 4, 7, 8, 9, 10]	Group103	13,64	3,00
GO:0055024	regulation of cardiac muscle tissue development	GO_BiologicalProcess-EBI-UniProt-GOA-ACAP-ARAP_08.05.2020_00h00	0,008760029	0,018588355	0,005636813	0,00815734	[5, 6, 7, 8, 9]	Group103	7,87	7,00
GO:0070306	lens fiber cell differentiation	GO_BiologicalProcess-EBI-UniProt-GOA-ACAP-ARAP_08.05.2020_00h00	0,015443421	0,028044315	0,005636813	0,00815734	[4, 5, 6, 8, 9, 10]	Group103	10,81	4,00
GO:2000249	regulation of actin cytoskeleton reorganization	GO_BiologicalProcess-EBI-UniProt-GOA-ACAP-ARAP_08.05.2020_00h00	0,006195141	0,01411607	0,006195141	0,00882188	[5, 6, 7, 8]	Group035	11,11	5,00
GO:0010661	positive regulation of muscle cell apoptotic process	GO_BiologicalProcess-EBI-UniProt-GOA-ACAP-ARAP_08.05.2020_00h00	0,00654021	0,014727013	0,00654021	0,00923934	[6, 7, 8]	Group038	13,79	4,00
GO:0035019	somatic stem cell population maintenance	GO_BiologicalProcess-EBI-UniProt-GOA-ACAP-ARAP_08.05.2020_00h00	0,020465165	0,034911167	0,006930633	0,0097138	[3, 4]	Group088	7,32	6,00

GO:0006140	regulation of nucleotide metabolic process	GO_BiologicalProcess-EBI-UniProt-GOA-ACAP-ARAP_08.05.2020_00h00	0,023751667	0,039055575	0,006930633	0,0097138	[4, 5, 6, 7, 8]	Group088	6,02	8,00
GO:0009179	purine ribonucleoside diphosphate metabolic process	GO_BiologicalProcess-EBI-UniProt-GOA-ACAP-ARAP_08.05.2020_00h00	0,007714132	0,01689342	0,006930633	0,0097138	[7, 8, 9]	Group088	6,45	10,00
GO:2001169	regulation of ATP biosynthetic process	GO_BiologicalProcess-EBI-UniProt-GOA-ACAP-ARAP_08.05.2020_00h00	0,014564467	0,026855865	0,006930633	0,0097138	[4, 5, 7, 8, 9, 10, 11, 12]	Group088	15,00	3,00
GO:0001501	skeletal system development	GO_BiologicalProcess-EBI-UniProt-GOA-ACAP-ARAP_08.05.2020_00h00	0,007357109	0,016222933	0,007357109	0,01023098	[4, 5]	Group037	4,44	25,00
GO:0051817	modulation of process of other organism involved in symbiotic interaction	GO_BiologicalProcess-EBI-UniProt-GOA-ACAP-ARAP_08.05.2020_00h00	0,007866058	0,017108677	0,007866058	0,01085394	[3]	Group044	7,34	8,00
GO:0044794	positive regulation by host of viral process	GO_BiologicalProcess-EBI-UniProt-GOA-ACAP-ARAP_08.05.2020_00h00	0,010835065	0,021783942	0,007866058	0,01085394	[3, 4, 5, 6]	Group044	16,67	3,00
GO:0048863	stem cell differentiation	GO_BiologicalProcess-EBI-UniProt-GOA-ACAP-ARAP_08.05.2020_00h00	0,014694748	0,02694037	0,008115702	0,01111227	[4]	Group045	5,05	14,00
GO:0060485	mesenchyme development	GO_BiologicalProcess-EBI-UniProt-GOA-ACAP-ARAP_08.05.2020_00h00	0,022654012	0,037835758	0,008115702	0,01111227	[4, 5, 6]	Group045	4,78	14,00
GO:0007569	cell aging	GO_BiologicalProcess-EBI-UniProt-GOA-ACAP-ARAP_08.05.2020_00h00	0,032775592	0,049240567	0,008240161	0,01119655	[3]	Group087	6,03	7,00
GO:2000772	regulation of cellular senescence	GO_BiologicalProcess-EBI-UniProt-GOA-ACAP-ARAP_08.05.2020_00h00	0,005625159	0,013066206	0,008240161	0,01119655	[4, 5, 6]	Group087	11,36	5,00
GO:2000773	negative regulation of cellular senescence	GO_BiologicalProcess-EBI-UniProt-GOA-ACAP-ARAP_08.05.2020_00h00	0,012620698	0,024499003	0,008240161	0,01119655	[3, 4, 5, 6, 7]	Group087	15,79	3,00
GO:0071479	cellular response to ionizing radiation	GO_BiologicalProcess-EBI-UniProt-GOA-ACAP-ARAP_08.05.2020_00h00	0,020465165	0,034911167	0,008240161	0,01119655	[5, 6]	Group087	7,32	6,00
GO:0071218	cellular response to misfolded protein	GO_BiologicalProcess-EBI-UniProt-GOA-ACAP-ARAP_08.05.2020_00h00	0,018932438	0,03306267	0,008316356	0,01121448	[5, 6]	Group047	13,64	3,00
GO:0006515	protein quality control for misfolded or incompletely synthesized proteins	GO_BiologicalProcess-EBI-UniProt-GOA-ACAP-ARAP_08.05.2020_00h00	0,005755048	0,013271279	0,008316356	0,01121448	[6, 7, 8]	Group047	14,29	4,00
GO:0046620	regulation of organ growth	GO_BiologicalProcess-EBI-UniProt-GOA-ACAP-ARAP_08.05.2020_00h00	0,024403341	0,039853238	0,008570778	0,01138506	[3, 4, 5]	Group086	6,42	7,00
GO:0003170	heart valve development	GO_BiologicalProcess-EBI-UniProt-GOA-ACAP-ARAP_08.05.2020_00h00	0,029100992	0,045283981	0,008570778	0,01138506	[3, 5, 6, 7]	Group086	7,58	5,00
GO:0055024	regulation of cardiac muscle tissue development	GO_BiologicalProcess-EBI-UniProt-GOA-ACAP-ARAP_08.05.2020_00h00	0,008760029	0,018588355	0,008570778	0,01138506	[5, 6, 7, 8, 9]	Group086	7,87	7,00
GO:2000725	regulation of cardiac muscle cell differentiation	GO_BiologicalProcess-EBI-UniProt-GOA-ACAP-ARAP_08.05.2020_00h00	0,020105848	0,034731582	0,008570778	0,01138506	[5, 6, 7, 8, 9, 10]	Group086	10,00	4,00
GO:0071496	cellular response to external stimulus	GO_BiologicalProcess-EBI-UniProt-GOA-ACAP-ARAP_08.05.2020_00h00	0,008531612	0,018224895	0,008531612	0,01141825	[3]	Group077	4,89	18,00
GO:0031668	cellular response to extracellular stimulus	GO_BiologicalProcess-EBI-UniProt-GOA-ACAP-ARAP_08.05.2020_00h00	0,019883081	0,034471214	0,008531612	0,01141825	[3, 4]	Group077	4,86	14,00
GO:0009267	cellular response to starvation	GO_BiologicalProcess-EBI-UniProt-GOA-ACAP-ARAP_08.05.2020_00h00	0,026896063	0,042899221	0,008531612	0,01141825	[4, 5, 6]	Group077	5,56	9,00
GO:0042149	cellular response to glucose starvation	GO_BiologicalProcess-EBI-UniProt-GOA-ACAP-ARAP_08.05.2020_00h00	0,025549145	0,041231927	0,008531612	0,01141825	[5, 6, 7]	Group077	9,30	4,00
GO:0048145	regulation of fibroblast proliferation	GO_BiologicalProcess-EBI-UniProt-GOA-ACAP-ARAP_08.05.2020_00h00	0,008760029	0,018588355	0,008760029	0,01155026	[4, 5]	Group017	7,87	7,00

GO:0120193	tight junction organization	GO_BiologicalProcess-EBI-UniProt-GOA-ACAP-ARAP_08.05.2020_00h00	0,00929126	0,019456752	0,00929126	0,01198438	[6]	Group049	7,78	7,00
GO:0070830	bicellular tight junction assembly	GO_BiologicalProcess-EBI-UniProt-GOA-ACAP-ARAP_08.05.2020_00h00	0,022756126	0,037940092	0,00929126	0,01198438	[8]	Group049	7,14	6,00
GO:0030856	regulation of epithelial cell differentiation	GO_BiologicalProcess-EBI-UniProt-GOA-ACAP-ARAP_08.05.2020_00h00	0,02252461	0,037685405	0,009262776	0,01203485	[4, 5, 6]	Group073	5,73	9,00
GO:0030858	positive regulation of epithelial cell differentiation	GO_BiologicalProcess-EBI-UniProt-GOA-ACAP-ARAP_08.05.2020_00h00	0,025888894	0,041569918	0,009262776	0,01203485	[4, 5, 6, 7]	Group073	7,81	5,00
GO:2000352	negative regulation of endothelial cell apoptotic process	GO_BiologicalProcess-EBI-UniProt-GOA-ACAP-ARAP_08.05.2020_00h00	0,008316356	0,017884837	0,009262776	0,01203485	[7, 8, 9]	Group073	12,90	4,00
GO:0051782	negative regulation of cell division	GO_BiologicalProcess-EBI-UniProt-GOA-ACAP-ARAP_08.05.2020_00h00	0,009205425	0,019319279	0,009205425	0,01204828	[3, 4, 5]	Group027	17,65	3,00
GO:0019211	phosphatase activator activity	GO_BiologicalProcess-EBI-UniProt-GOA-ACAP-ARAP_08.05.2020_00h00	0,009205425	0,019319279	0,009205425	0,01204828	[6, 8]	Group040	17,65	3,00
GO:0019751	polyol metabolic process	GO_BiologicalProcess-EBI-UniProt-GOA-ACAP-ARAP_08.05.2020_00h00	0,006168251	0,014088344	0,009676366	0,01239132	[4, 5]	Group098	6,67	10,00
GO:0046165	alcohol biosynthetic process	GO_BiologicalProcess-EBI-UniProt-GOA-ACAP-ARAP_08.05.2020_00h00	0,029087059	0,045336019	0,009676366	0,01239132	[4, 5]	Group098	5,24	10,00
GO:0046173	polyol biosynthetic process	GO_BiologicalProcess-EBI-UniProt-GOA-ACAP-ARAP_08.05.2020_00h00	0,00929126	0,019456752	0,009676366	0,01239132	[5, 6]	Group098	7,78	7,00
GO:0004435	phosphatidylinositol phospholipase C activity	GO_MolecularFunction-EBI-UniProt-GOA-ACAP-ARAP_08.05.2020_00h00	0,032678593	0,04924947	0,009676366	0,01239132	[7]	Group098	11,11	3,00
GO:0032959	inositol trisphosphate biosynthetic process	GO_BiologicalProcess-EBI-UniProt-GOA-ACAP-ARAP_08.05.2020_00h00	0,026696119	0,042651396	0,009676366	0,01239132	[6, 7, 8]	Group098	12,00	3,00
GO:1905114	cell surface receptor signaling pathway involved in cell-cell signaling	GO_BiologicalProcess-EBI-UniProt-GOA-ACAP-ARAP_08.05.2020_00h00	0,010511293	0,021222169	0,010511293	0,01336436	[3, 4, 5, 6]	Group139	4,10	30,00
GO:0016055	Wnt signaling pathway	GO_BiologicalProcess-EBI-UniProt-GOA-ACAP-ARAP_08.05.2020_00h00	0,002622226	0,007380795	0,010511293	0,01336436	[4, 5, 6, 7]	Group139	4,75	26,00
GO:0030111	regulation of Wnt signaling pathway	GO_BiologicalProcess-EBI-UniProt-GOA-ACAP-ARAP_08.05.2020_00h00	0,007104552	0,015738556	0,010511293	0,01336436	[4, 5, 6, 7, 8]	Group139	4,88	19,00
GO:0030177	positive regulation of Wnt signaling pathway	GO_BiologicalProcess-EBI-UniProt-GOA-ACAP-ARAP_08.05.2020_00h00	0,000124381	0,000734769	0,010511293	0,01336436	[4, 5, 6, 7, 8, 9]	Group139	8,02	15,00
GO:0060070	canonical Wnt signaling pathway	GO_BiologicalProcess-EBI-UniProt-GOA-ACAP-ARAP_08.05.2020_00h00	0,022989346	0,038195841	0,010511293	0,01336436	[5, 6, 7, 8]	Group139	4,56	16,00
GO:0060828	regulation of canonical Wnt signaling pathway	GO_BiologicalProcess-EBI-UniProt-GOA-ACAP-ARAP_08.05.2020_00h00	0,013665481	0,025896763	0,010511293	0,01336436	[5, 6, 7, 8, 9]	Group139	4,97	15,00
GO:0090263	positive regulation of canonical Wnt signaling pathway	GO_BiologicalProcess-EBI-UniProt-GOA-ACAP-ARAP_08.05.2020_00h00	0,000592761	0,002373526	0,010511293	0,01336436	[5, 6, 7, 8, 9, 10]	Group139	8,00	12,00
GO:1903209	positive regulation of oxidative stress-induced cell death	GO_BiologicalProcess-EBI-UniProt-GOA-ACAP-ARAP_08.05.2020_00h00	0,010835065	0,021783942	0,010835065	0,01367831	[4, 5, 6, 7, 8]	Group034	16,67	3,00
GO:0071850	mitotic cell cycle arrest	GO_BiologicalProcess-EBI-UniProt-GOA-ACAP-ARAP_08.05.2020_00h00	0,016667997	0,029927343	0,011528101	0,01445072	[4, 5, 6, 7]	Group048	14,29	3,00
GO:0007096	regulation of exit from mitosis	GO_BiologicalProcess-EBI-UniProt-GOA-ACAP-ARAP_08.05.2020_00h00	0,012620698	0,024499003	0,011528101	0,01445072	[5, 6, 7, 8, 9]	Group048	15,79	3,00
GO:0045927	positive regulation of growth	GO_BiologicalProcess-EBI-UniProt-GOA-ACAP-ARAP_08.05.2020_00h00	0,01690525	0,030296486	0,011754811	0,01463186	[2, 3, 4]	Group128	4,96	14,00

GO:0035019	somatic stem cell population maintenance	GO_BiologicalProcess-EBI-UniProt-GOA-ACAP-ARAP_08.05.2020_00h00	0,020465165	0,034911167	0,011754811	0,01463186	[3, 4]	Group128	7,32	6,00
GO:0046620	regulation of organ growth	GO_BiologicalProcess-EBI-UniProt-GOA-ACAP-ARAP_08.05.2020_00h00	0,024403341	0,039853238	0,011754811	0,01463186	[3, 4, 5]	Group128	6,42	7,00
GO:0055024	regulation of cardiac muscle tissue development	GO_BiologicalProcess-EBI-UniProt-GOA-ACAP-ARAP_08.05.2020_00h00	0,008760029	0,018588355	0,011754811	0,01463186	[5, 6, 7, 8, 9]	Group128	7,87	7,00
GO:0060487	lung epithelial cell differentiation	GO_BiologicalProcess-EBI-UniProt-GOA-ACAP-ARAP_08.05.2020_00h00	0,018932438	0,03306267	0,011754811	0,01463186	[5, 6, 7, 8, 9]	Group128	13,64	3,00
GO:0060043	regulation of cardiac muscle cell proliferation	GO_BiologicalProcess-EBI-UniProt-GOA-ACAP-ARAP_08.05.2020_00h00	0,031797417	0,04830179	0,011754811	0,01463186	[4, 5, 6, 7, 8, 9, 10, 11]	Group128	8,70	4,00
GO:0035019	somatic stem cell population maintenance	GO_BiologicalProcess-EBI-UniProt-GOA-ACAP-ARAP_08.05.2020_00h00	0,020465165	0,034911167	0,012154936	0,01502485	[3, 4]	Group083	7,32	6,00
GO:0060487	lung epithelial cell differentiation	GO_BiologicalProcess-EBI-UniProt-GOA-ACAP-ARAP_08.05.2020_00h00	0,018932438	0,03306267	0,012154936	0,01502485	[5, 6, 7, 8, 9]	Group083	13,64	3,00
GO:0060043	regulation of cardiac muscle cell proliferation	GO_BiologicalProcess-EBI-UniProt-GOA-ACAP-ARAP_08.05.2020_00h00	0,031797417	0,04830179	0,012154936	0,01502485	[4, 5, 6, 7, 8, 9, 10, 11]	Group083	8,70	4,00
GO:0036003	positive regulation of transcription from RNA polymerase II promoter in response to stress	GO_BiologicalProcess-EBI-UniProt-GOA-ACAP-ARAP_08.05.2020_00h00	0,008316356	0,017884837	0,012154936	0,01502485	[6, 7, 8, 9, 10, 11, 12, 13]	Group083	12,90	4,00
GO:0071214	cellular response to abiotic stimulus	GO_BiologicalProcess-EBI-UniProt-GOA-ACAP-ARAP_08.05.2020_00h00	0,011601744	0,022987306	0,012251197	0,0150394	[3, 4]	Group085	4,74	18,00
GO:0010212	response to ionizing radiation	GO_BiologicalProcess-EBI-UniProt-GOA-ACAP-ARAP_08.05.2020_00h00	0,029789085	0,045980893	0,012251197	0,0150394	[4]	Group085	5,45	9,00
GO:2000773	negative regulation of cellular senescence	GO_BiologicalProcess-EBI-UniProt-GOA-ACAP-ARAP_08.05.2020_00h00	0,012620698	0,024499003	0,012251197	0,0150394	[3, 4, 5, 6, 7]	Group085	15,79	3,00
GO:0071479	cellular response to ionizing radiation	GO_BiologicalProcess-EBI-UniProt-GOA-ACAP-ARAP_08.05.2020_00h00	0,020465165	0,034911167	0,012251197	0,0150394	[5, 6]	Group085	7,32	6,00
GO:0008544	epidermis development	GO_BiologicalProcess-EBI-UniProt-GOA-ACAP-ARAP_08.05.2020_00h00	0,012401821	0,024271049	0,012401821	0,01512003	[4]	Group020	4,41	22,00
GO:0034501	protein localization to kinetochore	GO_BiologicalProcess-EBI-UniProt-GOA-ACAP-ARAP_08.05.2020_00h00	0,012620698	0,024499003	0,012620698	0,01528221	[8]	Group004	15,79	3,00
GO:0009415	response to water	GO_BiologicalProcess-EBI-UniProt-GOA-ACAP-ARAP_08.05.2020_00h00	0,012620698	0,024499003	0,012620698	0,01528221	[3, 4]	Group028	15,79	3,00
GO:0050873	brown fat cell differentiation	GO_BiologicalProcess-EBI-UniProt-GOA-ACAP-ARAP_08.05.2020_00h00	0,013251976	0,025466148	0,013251976	0,01593819	[5]	Group022	9,26	5,00
GO:0019956	chemokine binding	GO_MolecularFunction-EBI-UniProt-GOA-ACAP-ARAP_08.05.2020_00h00	0,014057252	0,026326399	0,014057252	0,01646178	[4]	Group041	11,11	4,00
GO:0043276	anoikis	GO_BiologicalProcess-EBI-UniProt-GOA-ACAP-ARAP_08.05.2020_00h00	0,014057252	0,026326399	0,014057252	0,01646178	[5]	Group054	11,11	4,00
GO:2000209	regulation of anoikis	GO_BiologicalProcess-EBI-UniProt-GOA-ACAP-ARAP_08.05.2020_00h00	0,029607117	0,045996774	0,014057252	0,01646178	[6, 7]	Group054	11,54	3,00
GO:0008191	metalloendopeptidase inhibitor activity	GO_BiologicalProcess-EBI-UniProt-GOA-ACAP-ARAP_08.05.2020_00h00	0,009205425	0,019319279	0,013796489	0,01648171	[8, 9, 10, 11, 12]	Group069	17,65	3,00
GO:0008237	metallopeptidase activity	GO_BiologicalProcess-EBI-UniProt-GOA-ACAP-ARAP_08.05.2020_00h00	0,013796489	0,026041893	0,013796489	0,01648171	[7, 8]	Group069	5,43	12,00
GO:1905048	regulation of metallopeptidase activity	GO_BiologicalProcess-EBI-UniProt-GOA-ACAP-ARAP_08.05.2020_00h00	0,001606011	0,005140308	0,013796489	0,01648171	[6, 7, 8, 9]	Group069	20,00	4,00

GO:0030258	lipid modification	GO_BiologicalProcess-EBI-UniProt-GOA-ACAP-ARAP_08.05.2020_00h00	0,027085837	0,043130025	0,014310171	0,01654033	[4, 5]	Group081	4,56	15,00
GO:0046834	lipid phosphorylation	GO_BiologicalProcess-EBI-UniProt-GOA-ACAP-ARAP_08.05.2020_00h00	0,005052869	0,011969296	0,014310171	0,01654033	[5, 6]	Group081	7,32	9,00
GO:1904707	positive regulation of vascular smooth muscle cell proliferation	GO_BiologicalProcess-EBI-UniProt-GOA-ACAP-ARAP_08.05.2020_00h00	0,015443421	0,028044315	0,014310171	0,01654033	[5, 6, 7]	Group081	10,81	4,00
GO:0043550	regulation of lipid kinase activity	GO_BiologicalProcess-EBI-UniProt-GOA-ACAP-ARAP_08.05.2020_00h00	0,030797191	0,047081329	0,014310171	0,01654033	[5, 6, 7, 8, 9]	Group081	7,46	5,00
GO:0016051	carbohydrate biosynthetic process	GO_BiologicalProcess-EBI-UniProt-GOA-ACAP-ARAP_08.05.2020_00h00	0,029786352	0,046050951	0,013954064	0,01655882	[4]	Group061	4,86	12,00
GO:0043271	negative regulation of ion transport	GO_BiologicalProcess-EBI-UniProt-GOA-ACAP-ARAP_08.05.2020_00h00	0,011685916	0,023058603	0,013954064	0,01655882	[3, 4, 5, 6]	Group061	6,06	10,00
GO:0044070	regulation of anion transport	GO_BiologicalProcess-EBI-UniProt-GOA-ACAP-ARAP_08.05.2020_00h00	0,032775592	0,049240567	0,013954064	0,01655882	[5, 6]	Group061	6,03	7,00
GO:0034248	regulation of cellular amide metabolic process	GO_BiologicalProcess-EBI-UniProt-GOA-ACAP-ARAP_08.05.2020_00h00	0,000700788	0,00270425	0,014053418	0,01656628	[4, 5]	Group111	5,22	26,00
GO:0043043	peptide biosynthetic process	GO_BiologicalProcess-EBI-UniProt-GOA-ACAP-ARAP_08.05.2020_00h00	0,013397908	0,025694987	0,014053418	0,01656628	[5, 6]	Group111	4,06	29,00
GO:0006417	regulation of translation	GO_BiologicalProcess-EBI-UniProt-GOA-ACAP-ARAP_08.05.2020_00h00	0,000423629	0,001851203	0,014053418	0,01656628	[5, 6, 7, 8]	Group111	5,59	24,00
GO:0017148	negative regulation of translation	GO_BiologicalProcess-EBI-UniProt-GOA-ACAP-ARAP_08.05.2020_00h00	0,012469945	0,024354564	0,014053418	0,01656628	[5, 6, 7, 8, 9]	Group111	6,34	9,00
GO:0035278	miRNA mediated inhibition of translation	GO_BiologicalProcess-EBI-UniProt-GOA-ACAP-ARAP_08.05.2020_00h00	0,005222608	0,012340829	0,014053418	0,01656628	[6, 7, 8, 9, 10, 11]	Group111	21,43	3,00
GO:0031099	regeneration	GO_BiologicalProcess-EBI-UniProt-GOA-ACAP-ARAP_08.05.2020_00h00	0,014252392	0,026433215	0,014252392	0,01658121	[3]	Group000	5,41	12,00
GO:0044346	fibroblast apoptotic process	GO_BiologicalProcess-EBI-UniProt-GOA-ACAP-ARAP_08.05.2020_00h00	0,014564467	0,026855865	0,014564467	0,01672565	[5]	Group010	15,00	3,00
GO:0045655	regulation of monocyte differentiation	GO_BiologicalProcess-EBI-UniProt-GOA-ACAP-ARAP_08.05.2020_00h00	0,014564467	0,026855865	0,014564467	0,01672565	[6, 7, 8, 9, 10, 11]	Group011	15,00	3,00
GO:1904872	regulation of telomerase RNA localization to Cajal body	GO_BiologicalProcess-EBI-UniProt-GOA-ACAP-ARAP_08.05.2020_00h00	0,012620698	0,024499003	0,014564467	0,01672565	[3, 4, 6, 7]	Group053	15,79	3,00
GO:0090671	telomerase RNA localization to Cajal body	GO_BiologicalProcess-EBI-UniProt-GOA-ACAP-ARAP_08.05.2020_00h00	0,014564467	0,026855865	0,014564467	0,01672565	[5, 6]	Group053	15,00	3,00
GO:0038061	NIK/NF-kappaB signaling	GO_BiologicalProcess-EBI-UniProt-GOA-ACAP-ARAP_08.05.2020_00h00	0,015273142	0,027787827	0,015273142	0,01742705	[4, 5, 6]	Group051	5,81	10,00
GO:1901222	regulation of NIK/NF-kappaB signaling	GO_BiologicalProcess-EBI-UniProt-GOA-ACAP-ARAP_08.05.2020_00h00	0,025498083	0,04121903	0,015273142	0,01742705	[5, 6, 7]	Group051	6,36	7,00
GO:0042698	ovulation cycle	GO_BiologicalProcess-EBI-UniProt-GOA-ACAP-ARAP_08.05.2020_00h00	0,016363574	0,029491413	0,016363574	0,01855233	[2, 3, 4]	Group006	7,69	6,00
GO:0005080	protein kinase C binding	GO_MolecularFunction-EBI-UniProt-GOA-ACAP-ARAP_08.05.2020_00h00	0,016469596	0,029626697	0,016469596	0,01855435	[6]	Group036	8,77	5,00
GO:0034162	toll-like receptor 9 signaling pathway	GO_BiologicalProcess-EBI-UniProt-GOA-ACAP-ARAP_08.05.2020_00h00	0,026696119	0,042651396	0,016469596	0,01855435	[5, 6, 7]	Group046	12,00	3,00
GO:0070423	nucleotide-binding oligomerization domain containing signaling pathway	GO_BiologicalProcess-EBI-UniProt-GOA-ACAP-ARAP_08.05.2020_00h00	0,014057252	0,026326399	0,016469596	0,01855435	[5, 6, 7]	Group046	11,11	4,00

GO:0071243	cellular response to arsenic-containing substance	GO_BiologicalProcess-EBI-UniProt-GOA-ACAP-ARAP_08.05.2020_00h00	0,016667997	0,029927343	0,016667997	0,01865977	[4]	Group029	14,29	3,00
GO:0071349	cellular response to interleukin-12	GO_BiologicalProcess-EBI-UniProt-GOA-ACAP-ARAP_08.05.2020_00h00	0,017647753	0,031333763	0,017647753	0,01963312	[6]	Group030	8,62	5,00
GO:0043243	positive regulation of protein-containing complex disassembly	GO_BiologicalProcess-EBI-UniProt-GOA-ACAP-ARAP_08.05.2020_00h00	0,018466409	0,032485943	0,018466409	0,02041628	[4, 5, 6, 7]	Group007	10,26	4,00
GO:0030258	lipid modification	GO_BiologicalProcess-EBI-UniProt-GOA-ACAP-ARAP_08.05.2020_00h00	0,027085837	0,043130025	0,018857107	0,02071954	[4, 5]	Group104	4,56	15,00
GO:0071545	inositol phosphate catabolic process	GO_BiologicalProcess-EBI-UniProt-GOA-ACAP-ARAP_08.05.2020_00h00	0,001606011	0,005140308	0,018857107	0,02071954	[5, 6, 7]	Group104	20,00	4,00
GO:0046488	phosphatidylinositol metabolic process	GO_BiologicalProcess-EBI-UniProt-GOA-ACAP-ARAP_08.05.2020_00h00	0,017771911	0,031437557	0,018857107	0,02071954	[6, 7]	Group104	5,24	12,00
GO:0046856	phosphatidylinositol dephosphorylation	GO_BiologicalProcess-EBI-UniProt-GOA-ACAP-ARAP_08.05.2020_00h00	0,007393209	0,016265061	0,018857107	0,02071954	[6, 7, 8]	Group104	13,33	4,00
GO:0052866	phosphatidylinositol phosphate phosphatase activity	GO_BiologicalProcess-EBI-UniProt-GOA-ACAP-ARAP_08.05.2020_00h00	0,012752683	0,024655186	0,018857107	0,02071954	[7]	Group104	11,43	4,00
GO:0030545	receptor regulator activity	GO_BiologicalProcess-EBI-UniProt-GOA-ACAP-ARAP_08.05.2020_00h00	0,020130254	0,034648661	0,020130254	0,02184869	[4]	Group056	4,11	24,00
GO:0030546	signaling receptor activator activity	GO_BiologicalProcess-EBI-UniProt-GOA-ACAP-ARAP_08.05.2020_00h00	0,026597006	0,042564105	0,020130254	0,02184869	[5]	Group056	4,09	22,00
GO:0048015	phosphatidylinositol-mediated signaling	GO_BiologicalProcess-EBI-UniProt-GOA-ACAP-ARAP_08.05.2020_00h00	0,020030504	0,034664001	0,020030504	0,0218738	[5, 6, 7]	Group016	5,34	11,00
GO:0048012	hepatocyte growth factor receptor signaling pathway	GO_BiologicalProcess-EBI-UniProt-GOA-ACAP-ARAP_08.05.2020_00h00	0,021358484	0,036113199	0,021358484	0,02304127	[6, 7, 8]	Group015	13,04	3,00
GO:0046777	protein autophosphorylation	GO_BiologicalProcess-EBI-UniProt-GOA-ACAP-ARAP_08.05.2020_00h00	0,021527739	0,036271207	0,021527739	0,02308396	[7, 8]	Group014	4,94	13,00
GO:0032799	low-density lipoprotein receptor particle metabolic process	GO_BiologicalProcess-EBI-UniProt-GOA-ACAP-ARAP_08.05.2020_00h00	0,023946417	0,03924096	0,023946417	0,02552373	[5, 6]	Group002	12,50	3,00
GO:0009226	nucleotide-sugar biosynthetic process	GO_BiologicalProcess-EBI-UniProt-GOA-ACAP-ARAP_08.05.2020_00h00	0,023946417	0,03924096	0,023946417	0,02552373	[5, 6, 7]	Group025	12,50	3,00
GO:0072574	hepatocyte proliferation	GO_BiologicalProcess-EBI-UniProt-GOA-ACAP-ARAP_08.05.2020_00h00	0,023946417	0,03924096	0,023946417	0,02552373	[5, 7, 8, 9, 10]	Group031	12,50	3,00
GO:0046324	regulation of glucose import	GO_BiologicalProcess-EBI-UniProt-GOA-ACAP-ARAP_08.05.2020_00h00	0,024371797	0,039869763	0,024371797	0,0258225	[5, 6, 7, 8, 10, 11]	Group013	7,94	5,00
GO:0051058	negative regulation of small GTPase mediated signal transduction	GO_BiologicalProcess-EBI-UniProt-GOA-ACAP-ARAP_08.05.2020_00h00	0,024371797	0,039869763	0,024371797	0,0258225	[5, 6, 7, 8]	Group024	7,94	5,00
GO:0016579	protein deubiquitination	GO_BiologicalProcess-EBI-UniProt-GOA-ACAP-ARAP_08.05.2020_00h00	0,025704479	0,041412771	0,025704479	0,02707336	[6, 7, 8, 9]	Group063	4,70	14,00
GO:0071108	protein K48-linked deubiquitination	GO_BiologicalProcess-EBI-UniProt-GOA-ACAP-ARAP_08.05.2020_00h00	0,003317585	0,008674669	0,025704479	0,02707336	[7, 8, 9, 10]	Group063	12,82	5,00
GO:0061578	Lys63-specific deubiquitinase activity	GO_BiologicalProcess-EBI-UniProt-GOA-ACAP-ARAP_08.05.2020_00h00	0,006402782	0,014520053	0,025704479	0,02707336	[8, 9, 10, 11]	Group063	20,00	3,00
GO:0098631	cell adhesion mediator activity	GO_BiologicalProcess-EBI-UniProt-GOA-ACAP-ARAP_08.05.2020_00h00	0,025888894	0,041569918	0,025888894	0,02710719	[3]	Group032	7,81	5,00
GO:1900034	regulation of cellular response to heat	GO_BiologicalProcess-EBI-UniProt-GOA-ACAP-ARAP_08.05.2020_00h00	0,026506023	0,042489555	0,026506023	0,02759106	[4, 5, 6]	Group033	6,90	6,00

GO:0032469	endoplasmic reticulum calcium ion homeostasis	GO_BiologicalProcess-EBI-UniProt-GOA-ACAP-ARAP_08.05.2020_00h00	0,026696119	0,042651396	0,026696119	0,02762738	[8, 10]	Group001	12,00	3,00
GO:0032967	positive regulation of collagen biosynthetic process	GO_BiologicalProcess-EBI-UniProt-GOA-ACAP-ARAP_08.05.2020_00h00	0,026696119	0,042651396	0,026696119	0,02762738	[4, 5, 6]	Group003	12,00	3,00
GO:0048305	immunoglobulin secretion	GO_BiologicalProcess-EBI-UniProt-GOA-ACAP-ARAP_08.05.2020_00h00	0,026696119	0,042651396	0,026696119	0,02762738	[4, 5, 6, 7, 8, 9]	Group019	12,00	3,00
GO:0016780	phosphotransferase activity, for other substituted phosphate groups	GO_MolecularFunction-EBI-UniProt-GOA-ACAP-ARAP_08.05.2020_00h00	0,026696119	0,042651396	0,026696119	0,02762738	[4]	Group039	12,00	3,00
GO:0045807	positive regulation of endocytosis	GO_BiologicalProcess-EBI-UniProt-GOA-ACAP-ARAP_08.05.2020_00h00	0,028982626	0,045320872	0,028982626	0,02982027	[3, 4, 5, 6, 7]	Group012	6,19	7,00
GO:0002029	desensitization of G protein-coupled receptor signaling pathway	GO_BiologicalProcess-EBI-UniProt-GOA-ACAP-ARAP_08.05.2020_00h00	0,029607117	0,045996774	0,029607117	0,03028774	[5, 6, 7, 8]	Group008	11,54	3,00
GO:0007617	mating behavior	GO_BiologicalProcess-EBI-UniProt-GOA-ACAP-ARAP_08.05.2020_00h00	0,029607117	0,045996774	0,029607117	0,03028774	[3, 4, 5, 6]	Group009	11,54	3,00
GO:0007040	lysosome organization	GO_BiologicalProcess-EBI-UniProt-GOA-ACAP-ARAP_08.05.2020_00h00	0,030797191	0,047081329	0,030797191	0,03114716	[7]	Group043	7,46	5,00
GO:0051453	regulation of intracellular pH	GO_BiologicalProcess-EBI-UniProt-GOA-ACAP-ARAP_08.05.2020_00h00	0,030644687	0,046923146	0,030644687	0,03117002	[8, 10]	Group026	6,67	6,00
GO:0035270	endocrine system development	GO_BiologicalProcess-EBI-UniProt-GOA-ACAP-ARAP_08.05.2020_00h00	0,032142218	0,048748184	0,032142218	0,03232381	[4, 5]	Group005	5,67	8,00
GO:0072527	pyrimidine-containing compound metabolic process	GO_BiologicalProcess-EBI-UniProt-GOA-ACAP-ARAP_08.05.2020_00h00	0,023341313	0,038579684	0,034110095	0,0341101	[4]	Group108	6,48	7,00
GO:0072528	pyrimidine-containing compound biosynthetic process	GO_BiologicalProcess-EBI-UniProt-GOA-ACAP-ARAP_08.05.2020_00h00	0,02291313	0,038135417	0,034110095	0,0341101	[5]	Group108	8,06	5,00
GO:0006220	pyrimidine nucleotide metabolic process	GO_BiologicalProcess-EBI-UniProt-GOA-ACAP-ARAP_08.05.2020_00h00	0,030797191	0,047081329	0,034110095	0,0341101	[5, 6, 7, 8]	Group108	7,46	5,00
GO:0003841	1-acylglycerol-3-phosphate O-acyltransferase activity	GO_MolecularFunction-EBI-UniProt-GOA-ACAP-ARAP_08.05.2020_00h00	0,007729088	0,016887529	0,034110095	0,0341101	[7]	Group108	18,75	3,00
GO:0016024	CDP-diacylglycerol biosynthetic process	GO_BiologicalProcess-EBI-UniProt-GOA-ACAP-ARAP_08.05.2020_00h00	0,005222608	0,012340829	0,034110095	0,0341101	[6, 7, 8, 9, 10]	Group108	21,43	3,00
