# Supplementary material for: Analysis of the Role of FRMD5 in the Biology of Papillary Thyroid Carcinoma
Source: Int J Mol Sci. 2021 Jun 23;22(13):6726. doi: 10.3390/ijms22136726 (PMC8268710; doi:10.3390/ijms22136726)
Supplement: Supplementary file 1 [file ijms-22-06726-s001.zip › Supplementary Table S8.pdf]

Supplementary Table S8. Gene ontology (GO) enrichment analysis of the downregulated genes in BCPAP siFRMD5 (in comparison with BCPAP siNEG).

GOID	GOTerm	Ontology Source	Term PValue	Term PValue Corrected with Benjamini-Hochberg	Group PValue	Group PValue Corrected with Benjamini-Hochberg	GOLevels	GOGroups	% Associated Genes	Nr. Genes
GO:0007018	microtubule-based movement	GO_BiologicalProcess-EBI-UniProt-GOA-ACAP-ARAP_08.05.2020_00h00	3,81545E-06	0,00051509	7,44462E-07	2,1589E-05	[3]	Group26	7,47	23,00
GO:0051656	establishment of organelle localization	GO_BiologicalProcess-EBI-UniProt-GOA-ACAP-ARAP_08.05.2020_00h00	0,003018338	0,03595373	7,44462E-07	2,1589E-05	[3, 4]	Group26	4,81	22,00
GO:0003341	cilium movement	GO_BiologicalProcess-EBI-UniProt-GOA-ACAP-ARAP_08.05.2020_00h00	0,004320167	0,0364514	7,44462E-07	2,1589E-05	[4]	Group26	8,64	7,00
GO:0099111	microtubule-based transport	GO_BiologicalProcess-EBI-UniProt-GOA-ACAP-ARAP_08.05.2020_00h00	5,07271E-06	0,00051361	7,44462E-07	2,1589E-05	[4]	Group26	8,74	18,00
GO:0030705	cytoskeleton-dependent intracellular transport	GO_BiologicalProcess-EBI-UniProt-GOA-ACAP-ARAP_08.05.2020_00h00	8,62051E-06	0,00069826	7,44462E-07	2,1589E-05	[4, 5]	Group26	8,41	18,00
GO:0010970	transport along microtubule	GO_BiologicalProcess-EBI-UniProt-GOA-ACAP-ARAP_08.05.2020_00h00	0,000145428	0,00654426	7,44462E-07	2,1589E-05	[5, 6]	Group26	8,00	14,00
GO:0098840	protein transport along microtubule	GO_BiologicalProcess-EBI-UniProt-GOA-ACAP-ARAP_08.05.2020_00h00	0,000491532	0,01327137	7,44462E-07	2,1589E-05	[5, 6, 7, 8, 9]	Group26	10,96	8,00
GO:0042073	intraciliary transport	GO_BiologicalProcess-EBI-UniProt-GOA-ACAP-ARAP_08.05.2020_00h00	0,002533206	0,03419828	7,44462E-07	2,1589E-05	[5, 6, 7, 8, 9, 10]	Group26	10,91	6,00
GO:0007018	microtubule-based movement	GO_BiologicalProcess-EBI-UniProt-GOA-ACAP-ARAP_08.05.2020_00h00	3,81545E-06	0,00051509	4,25032E-06	6,163E-05	[3]	Group23	7,47	23,00
GO:0003341	cilium movement	GO_BiologicalProcess-EBI-UniProt-GOA-ACAP-ARAP_08.05.2020_00h00	0,004320167	0,0364514	4,25032E-06	6,163E-05	[4]	Group23	8,64	7,00
GO:0003351	epithelial cilium movement involved in extracellular fluid movement	GO_BiologicalProcess-EBI-UniProt-GOA-ACAP-ARAP_08.05.2020_00h00	0,007314167	0,04356232	4,25032E-06	6,163E-05	[5]	Group23	12,90	4,00
GO:0036158	outer dynein arm assembly	GO_BiologicalProcess-EBI-UniProt-GOA-ACAP-ARAP_08.05.2020_00h00	0,009795591	0,04779777	4,25032E-06	6,163E-05	[7, 8, 9, 10]	Group23	16,67	3,00
GO:0036159	inner dynein arm assembly	GO_BiologicalProcess-EBI-UniProt-GOA-ACAP-ARAP_08.05.2020_00h00	0,005776326	0,04332244	4,25032E-06	6,163E-05	[7, 8, 9, 10]	Group23	20,00	3,00
GO:0032886	regulation of microtubule-based process	GO_BiologicalProcess-EBI-UniProt-GOA-ACAP-ARAP_08.05.2020_00h00	0,004141333	0,03727199	1,10085E-05	7,9812E-05	[3, 4]	Group29	5,67	14,00
GO:0051129	negative regulation of cellular component organization	GO_BiologicalProcess-EBI-UniProt-GOA-ACAP-ARAP_08.05.2020_00h00	0,000690086	0,01330881	1,10085E-05	7,9812E-05	[3, 4, 5]	Group29	4,50	35,00
GO:0000226	microtubule cytoskeleton organization	GO_BiologicalProcess-EBI-UniProt-GOA-ACAP-ARAP_08.05.2020_00h00	0,002389811	0,03456692	1,10085E-05	7,9812E-05	[3, 6]	Group29	4,49	28,00
GO:0033043	regulation of organelle organization	GO_BiologicalProcess-EBI-UniProt-GOA-ACAP-ARAP_08.05.2020_00h00	0,000180345	0,00730399	1,10085E-05	7,9812E-05	[4, 5]	Group29	4,11	57,00
GO:0007098	centrosome cycle	GO_BiologicalProcess-EBI-UniProt-GOA-ACAP-ARAP_08.05.2020_00h00	0,006605746	0,04385782	1,10085E-05	7,9812E-05	[3, 4, 5, 8]	Group29	6,77	9,00
GO:0010639	negative regulation of organelle organization	GO_BiologicalProcess-EBI-UniProt-GOA-ACAP-ARAP_08.05.2020_00h00	0,000843036	0,01551953	1,10085E-05	7,9812E-05	[4, 5, 6]	Group29	5,23	23,00
GO:0070925	organelle assembly	GO_BiologicalProcess-EBI-UniProt-GOA-ACAP-ARAP_08.05.2020_00h00	5,21096E-05	0,00301491	1,10085E-05	7,9812E-05	[5]	Group29	4,75	43,00

GO:0120036	plasma membrane bounded cell projection organization	GO_BiologicalProcess-EBI-UniProt-GOA-ACAP-ARAP_08.05.2020_00h00	3,95132E-05	0,00266714	1,10085E-05	7,9812E-05	[5]	Group29	4,13	67,00
GO:1902115	regulation of organelle assembly	GO_BiologicalProcess-EBI-UniProt-GOA-ACAP-ARAP_08.05.2020_00h00	0,000240963	0,00887181	1,10085E-05	7,9812E-05	[4, 5, 6]	Group29	7,28	15,00
GO:0051298	centrosome duplication	GO_BiologicalProcess-EBI-UniProt-GOA-ACAP-ARAP_08.05.2020_00h00	0,009555393	0,04777696	1,10085E-05	7,9812E-05	[3, 4, 5, 6, 9]	Group29	8,33	6,00
GO:0044782	cilium organization	GO_BiologicalProcess-EBI-UniProt-GOA-ACAP-ARAP_08.05.2020_00h00	1,73402E-06	0,00070228	1,10085E-05	7,9812E-05	[5, 6]	Group29	6,88	28,00
GO:0051493	regulation of cytoskeleton organization	GO_BiologicalProcess-EBI-UniProt-GOA-ACAP-ARAP_08.05.2020_00h00	0,007308025	0,04417538	1,10085E-05	7,9812E-05	[5, 6]	Group29	4,28	25,00
GO:0070507	regulation of microtubule cytoskeleton organization	GO_BiologicalProcess-EBI-UniProt-GOA-ACAP-ARAP_08.05.2020_00h00	0,00642897	0,04339555	1,10085E-05	7,9812E-05	[4, 5, 6, 7]	Group29	5,80	12,00
GO:0120035	regulation of plasma membrane bounded cell projection organization	GO_BiologicalProcess-EBI-UniProt-GOA-ACAP-ARAP_08.05.2020_00h00	0,007689316	0,04386159	1,10085E-05	7,9812E-05	[5, 6]	Group29	4,04	30,00
GO:1902117	positive regulation of organelle assembly	GO_BiologicalProcess-EBI-UniProt-GOA-ACAP-ARAP_08.05.2020_00h00	0,003758924	0,03903498	1,10085E-05	7,9812E-05	[4, 5, 6, 7]	Group29	8,86	7,00
GO:0051494	negative regulation of cytoskeleton organization	GO_BiologicalProcess-EBI-UniProt-GOA-ACAP-ARAP_08.05.2020_00h00	0,003792188	0,03745941	1,10085E-05	7,9812E-05	[5, 6, 7]	Group29	6,51	11,00
GO:0097711	ciliary basal body-plasma membrane docking	GO_BiologicalProcess-EBI-UniProt-GOA-ACAP-ARAP_08.05.2020_00h00	0,003552196	0,03785893	1,10085E-05	7,9812E-05	[4, 5, 7, 8]	Group29	8,08	8,00
GO:0120031	plasma membrane bounded cell projection assembly	GO_BiologicalProcess-EBI-UniProt-GOA-ACAP-ARAP_08.05.2020_00h00	3,70553E-06	0,00075037	1,10085E-05	7,9812E-05	[6]	Group29	5,85	35,00
GO:0120032	regulation of plasma membrane bounded cell projection assembly	GO_BiologicalProcess-EBI-UniProt-GOA-ACAP-ARAP_08.05.2020_00h00	0,000491566	0,01244277	1,10085E-05	7,9812E-05	[5, 6, 7]	Group29	7,11	14,00
GO:0120034	positive regulation of plasma membrane bounded cell projection assembly	GO_BiologicalProcess-EBI-UniProt-GOA-ACAP-ARAP_08.05.2020_00h00	0,008254359	0,04517588	1,10085E-05	7,9812E-05	[4, 5, 6, 7, 8]	Group29	7,02	8,00
GO:0046605	regulation of centrosome cycle	GO_BiologicalProcess-EBI-UniProt-GOA-ACAP-ARAP_08.05.2020_00h00	0,005017432	0,0406412	1,10085E-05	7,9812E-05	[4, 5, 6, 7, 8, 9]	Group29	9,52	6,00
GO:1902017	regulation of cilium assembly	GO_BiologicalProcess-EBI-UniProt-GOA-ACAP-ARAP_08.05.2020_00h00	0,0001401	0,00709256	1,10085E-05	7,9812E-05	[5, 6, 7, 8]	Group29	13,11	8,00
GO:0051640	organelle localization	GO_BiologicalProcess-EBI-UniProt-GOA-ACAP-ARAP_08.05.2020_00h00	0,000314103	0,00978551	1,06653E-05	0,0001031	[3]	Group21	4,81	33,00
GO:0051656	establishment of organelle localization	GO_BiologicalProcess-EBI-UniProt-GOA-ACAP-ARAP_08.05.2020_00h00	0,003018338	0,03595373	1,06653E-05	0,0001031	[3, 4]	Group21	4,81	22,00
GO:0051648	vesicle localization	GO_BiologicalProcess-EBI-UniProt-GOA-ACAP-ARAP_08.05.2020_00h00	0,000468468	0,0135521	1,06653E-05	0,0001031	[4]	Group21	6,58	16,00
GO:0030705	cytoskeleton-dependent intracellular transport	GO_BiologicalProcess-EBI-UniProt-GOA-ACAP-ARAP_08.05.2020_00h00	8,62051E-06	0,00069826	1,06653E-05	0,0001031	[4, 5]	Group21	8,41	18,00
GO:0043970	histone H3-K9 acetylation	GO_BiologicalProcess-EBI-UniProt-GOA-ACAP-ARAP_08.05.2020_00h00	0,003769295	0,03816412	0,000226447	0,00131339	[8, 9, 10, 11, 12, 13]	Group27	23,08	3,00
GO:0003007	heart morphogenesis	GO_BiologicalProcess-EBI-UniProt-GOA-ACAP-ARAP_08.05.2020_00h00	0,010306201	0,04797714	0,000226447	0,00131339	[4, 5, 6, 7]	Group27	5,09	14,00
GO:1902275	regulation of chromatin organization	GO_BiologicalProcess-EBI-UniProt-GOA-ACAP-ARAP_08.05.2020_00h00	0,006192945	0,04400251	0,000226447	0,00131339	[5, 6, 7]	Group27	5,83	12,00
GO:0003208	cardiac ventricle morphogenesis	GO_BiologicalProcess-EBI-UniProt-GOA-ACAP-ARAP_08.05.2020_00h00	0,010866376	0,04889869	0,000226447	0,00131339	[4, 5, 6, 7, 8, 9]	Group27	8,11	6,00

GO:0010717	regulation of epithelial to mesenchymal transition	GO_BiologicalProcess-EBI-UniProt-GOA-ACAP-ARAP_08.05.2020_00h00	0,008607737	0,04527446	0,000226447	0,00131339	[4, 5, 6, 7, 8, 9]	Group27	7,61	7,00
GO:0043543	protein acylation	GO_BiologicalProcess-EBI-UniProt-GOA-ACAP-ARAP_08.05.2020_00h00	0,006248162	0,0436294	0,000226447	0,00131339	[6, 7]	Group27	5,41	14,00
GO:2000136	regulation of cell proliferation involved in heart morphogenesis	GO_BiologicalProcess-EBI-UniProt-GOA-ACAP-ARAP_08.05.2020_00h00	0,011417896	0,0497231	0,000226447	0,00131339	[4, 5, 6, 7, 8, 9]	Group27	15,79	3,00
GO:2000826	regulation of heart morphogenesis	GO_BiologicalProcess-EBI-UniProt-GOA-ACAP-ARAP_08.05.2020_00h00	0,004815062	0,03979796	0,000226447	0,00131339	[5, 6, 7, 8]	Group27	11,36	5,00
GO:0060956	endocardial cell differentiation	GO_BiologicalProcess-EBI-UniProt-GOA-ACAP-ARAP_08.05.2020_00h00	0,000300709	0,01014893	0,000226447	0,00131339	[4, 6, 7, 8, 9]	Group27	50,00	3,00
GO:1901983	regulation of protein acetylation	GO_BiologicalProcess-EBI-UniProt-GOA-ACAP-ARAP_08.05.2020_00h00	0,010866376	0,04889869	0,000226447	0,00131339	[6, 7, 8, 9]	Group27	8,11	6,00
GO:0001657	ureteric bud development	GO_BiologicalProcess-EBI-UniProt-GOA-ACAP-ARAP_08.05.2020_00h00	0,006699307	0,0437616	0,000226447	0,00131339	[5, 6, 8, 9, 10, 11]	Group27	7,27	8,00
GO:0060675	ureteric bud morphogenesis	GO_BiologicalProcess-EBI-UniProt-GOA-ACAP-ARAP_08.05.2020_00h00	0,008944069	0,0458525	0,000226447	0,00131339	[6, 7, 8, 9, 10, 11, 12, 13]	Group27	8,45	6,00
GO:0032550	purine ribonucleoside binding	GO_MolecularFunction-EBI-UniProt-GOA-ACAP-ARAP_08.05.2020_00h00	0,000678931	0,01374836	0,000493656	0,002386	[5]	Group19	5,31	23,00
GO:0032561	guanyl ribonucleotide binding	GO_MolecularFunction-EBI-UniProt-GOA-ACAP-ARAP_08.05.2020_00h00	0,000493656	0,01176063	0,000493656	0,002386	[5, 6, 7]	Group19	5,33	24,00
GO:0000910	cytokinesis	GO_BiologicalProcess-EBI-UniProt-GOA-ACAP-ARAP_08.05.2020_00h00	0,005824229	0,04288751	0,000878179	0,00363817	[3, 4]	Group14	6,15	11,00
GO:0051781	positive regulation of cell division	GO_BiologicalProcess-EBI-UniProt-GOA-ACAP-ARAP_08.05.2020_00h00	0,002936431	0,03603801	0,000878179	0,00363817	[3, 4, 5]	Group14	8,33	8,00
GO:0048755	branching morphogenesis of a nerve	GO_BiologicalProcess-EBI-UniProt-GOA-ACAP-ARAP_08.05.2020_00h00	0,001673155	0,02823449	0,001319666	0,00478379	[3, 4, 5, 6]	Group13	30,00	3,00
GO:0021544	subpallium development	GO_BiologicalProcess-EBI-UniProt-GOA-ACAP-ARAP_08.05.2020_00h00	0,003836539	0,03699519	0,001319666	0,00478379	[3, 4, 5, 7, 8, 9, 10]	Group13	15,38	4,00
GO:0008015	blood circulation	GO_BiologicalProcess-EBI-UniProt-GOA-ACAP-ARAP_08.05.2020_00h00	0,001739129	0,02817389	0,001918974	0,00556502	[4]	Group25	4,66	27,00
GO:0090257	regulation of muscle system process	GO_BiologicalProcess-EBI-UniProt-GOA-ACAP-ARAP_08.05.2020_00h00	0,009854881	0,04751461	0,001918974	0,00556502	[4, 5]	Group25	5,28	13,00
GO:1903522	regulation of blood circulation	GO_BiologicalProcess-EBI-UniProt-GOA-ACAP-ARAP_08.05.2020_00h00	0,005426986	0,04226787	0,001918974	0,00556502	[4, 5]	Group25	5,18	16,00
GO:0045932	negative regulation of muscle contraction	GO_BiologicalProcess-EBI-UniProt-GOA-ACAP-ARAP_08.05.2020_00h00	0,003311349	0,03725268	0,001918974	0,00556502	[3, 4, 5, 6, 7]	Group25	16,00	4,00
GO:0060047	heart contraction	GO_BiologicalProcess-EBI-UniProt-GOA-ACAP-ARAP_08.05.2020_00h00	0,007254847	0,04451838	0,001918974	0,00556502	[5]	Group25	5,15	15,00
GO:0055117	regulation of cardiac muscle contraction	GO_BiologicalProcess-EBI-UniProt-GOA-ACAP-ARAP_08.05.2020_00h00	0,006792301	0,04366479	0,001918974	0,00556502	[6, 7, 8]	Group25	7,95	7,00
GO:0055118	negative regulation of cardiac muscle contraction	GO_BiologicalProcess-EBI-UniProt-GOA-ACAP-ARAP_08.05.2020_00h00	0,000300709	0,01014893	0,001918974	0,00556502	[5, 6, 7, 8, 9]	Group25	50,00	3,00
GO:0060322	head development	GO_BiologicalProcess-EBI-UniProt-GOA-ACAP-ARAP_08.05.2020_00h00	0,001750239	0,02726334	0,001750239	0,00563966	[3]	Group15	4,20	37,00
GO:0007420	brain development	GO_BiologicalProcess-EBI-UniProt-GOA-ACAP-ARAP_08.05.2020_00h00	0,004225209	0,03640872	0,001750239	0,00563966	[4, 5, 6, 7]	Group15	4,07	34,00
GO:0065004	protein-DNA complex assembly	GO_BiologicalProcess-EBI-UniProt-GOA-ACAP-ARAP_08.05.2020_00h00	0,002584411	0,03376408	0,002584411	0,00576522	[6, 7]	Group05	5,77	15,00

GO:0043462	regulation of ATPase activity	GO_BiologicalProcess-EBI-UniProt-GOA-ACAP-ARAP_08.05.2020_00h00	0,001253543	0,02207326	0,0021924	0,00577996	[5]	Group20	9,52	8,00
GO:0060589	nucleoside-triphosphatase regulator activity	GO_BiologicalProcess-EBI-UniProt-GOA-ACAP-ARAP_08.05.2020_00h00	0,011320079	0,04983296	0,0021924	0,00577996	[5]	Group20	4,67	17,00
GO:0060590	ATPase regulator activity	GO_BiologicalProcess-EBI-UniProt-GOA-ACAP-ARAP_08.05.2020_00h00	0,003167177	0,03664877	0,0021924	0,00577996	[6]	Group20	12,50	5,00
GO:0051097	negative regulation of helicase activity	GO_BiologicalProcess-EBI-UniProt-GOA-ACAP-ARAP_08.05.2020_00h00	0,000300709	0,01014893	0,0021924	0,00577996	[7]	Group20	50,00	3,00
GO:0035809	regulation of urine volume	GO_BiologicalProcess-EBI-UniProt-GOA-ACAP-ARAP_08.05.2020_00h00	0,002411896	0,03368338	0,002411896	0,00582875	[4]	Group12	17,39	4,00
GO:0060337	type I interferon signaling pathway	GO_BiologicalProcess-EBI-UniProt-GOA-ACAP-ARAP_08.05.2020_00h00	0,002936431	0,03603801	0,002936431	0,00608261	[5, 6, 7, 8, 9]	Group04	8,33	8,00
GO:0001504	neurotransmitter uptake	GO_BiologicalProcess-EBI-UniProt-GOA-ACAP-ARAP_08.05.2020_00h00	0,009784135	0,04832408	0,004099257	0,00699285	[4, 5]	Group28	9,62	5,00
GO:0051580	regulation of neurotransmitter uptake	GO_BiologicalProcess-EBI-UniProt-GOA-ACAP-ARAP_08.05.2020_00h00	0,002837319	0,03590982	0,004099257	0,00699285	[4, 5, 6]	Group28	16,67	4,00
GO:0051952	regulation of amine transport	GO_BiologicalProcess-EBI-UniProt-GOA-ACAP-ARAP_08.05.2020_00h00	0,00507975	0,04033919	0,004099257	0,00699285	[4, 5, 6]	Group28	7,62	8,00
GO:0051953	negative regulation of amine transport	GO_BiologicalProcess-EBI-UniProt-GOA-ACAP-ARAP_08.05.2020_00h00	0,005743539	0,04388931	0,004099257	0,00699285	[3, 4, 5, 6, 7]	Group28	13,79	4,00
GO:0014059	regulation of dopamine secretion	GO_BiologicalProcess-EBI-UniProt-GOA-ACAP-ARAP_08.05.2020_00h00	0,003929641	0,03701174	0,004099257	0,00699285	[3, 4, 5, 6, 7, 8, 9]	Group28	11,90	5,00
GO:0015872	dopamine transport	GO_BiologicalProcess-EBI-UniProt-GOA-ACAP-ARAP_08.05.2020_00h00	0,002304964	0,03457446	0,004099257	0,00699285	[5, 6, 7]	Group28	11,11	6,00
GO:0045921	positive regulation of exocytosis	GO_BiologicalProcess-EBI-UniProt-GOA-ACAP-ARAP_08.05.2020_00h00	0,01075657	0,04894843	0,004099257	0,00699285	[4, 5, 6, 7, 8]	Group28	7,29	7,00
GO:0032225	regulation of synaptic transmission, dopaminergic	GO_BiologicalProcess-EBI-UniProt-GOA-ACAP-ARAP_08.05.2020_00h00	0,000570006	0,01282513	0,004099257	0,00699285	[5, 6, 7, 8, 9]	Group28	25,00	4,00
GO:0014047	glutamate secretion	GO_BiologicalProcess-EBI-UniProt-GOA-ACAP-ARAP_08.05.2020_00h00	0,008308607	0,04486648	0,004099257	0,00699285	[4, 6, 7, 8, 9, 10]	Group28	10,00	5,00
GO:0001963	synaptic transmission, dopaminergic	GO_BiologicalProcess-EBI-UniProt-GOA-ACAP-ARAP_08.05.2020_00h00	0,008195748	0,04546956	0,004099257	0,00699285	[7, 8]	Group28	12,50	4,00
GO:0006835	dicarboxylic acid transport	GO_BiologicalProcess-EBI-UniProt-GOA-ACAP-ARAP_08.05.2020_00h00	0,007065354	0,04402259	0,004099257	0,00699285	[7, 8]	Group28	7,21	8,00
GO:0015800	acidic amino acid transport	GO_BiologicalProcess-EBI-UniProt-GOA-ACAP-ARAP_08.05.2020_00h00	0,001253543	0,02207326	0,004099257	0,00699285	[6, 8, 9]	Group28	9,52	8,00
GO:1901216	positive regulation of neuron death	GO_BiologicalProcess-EBI-UniProt-GOA-ACAP-ARAP_08.05.2020_00h00	0,004015227	0,03695834	0,004015227	0,0072776	[4, 5, 6]	Group09	7,92	8,00
GO:0000002	mitochondrial genome maintenance	GO_BiologicalProcess-EBI-UniProt-GOA-ACAP-ARAP_08.05.2020_00h00	0,003836539	0,03699519	0,003836539	0,00741731	[6]	Group00	15,38	4,00
GO:0045444	fat cell differentiation	GO_BiologicalProcess-EBI-UniProt-GOA-ACAP-ARAP_08.05.2020_00h00	0,008382128	0,04466792	0,008382128	0,00972327	[4]	Group02	5,39	13,00
GO:0006914	autophagy	GO_BiologicalProcess-EBI-UniProt-GOA-ACAP-ARAP_08.05.2020_00h00	0,006077953	0,04395663	0,006077953	0,00979226	[3, 4]	Group22	4,40	24,00
GO:0010506	regulation of autophagy	GO_BiologicalProcess-EBI-UniProt-GOA-ACAP-ARAP_08.05.2020_00h00	0,011320079	0,04983296	0,006077953	0,00979226	[4, 5, 6]	Group22	4,67	17,00
GO:0000422	autophagy of mitochondrion	GO_BiologicalProcess-EBI-UniProt-GOA-ACAP-ARAP_08.05.2020_00h00	0,001253543	0,02207326	0,006077953	0,00979226	[4, 5, 7]	Group22	9,52	8,00

GO:0000045	autophagosome assembly	GO_BiologicalProcess-EBI-UniProt-GOA-ACAP-ARAP_08.05.2020_00h00	0,003552196	0,03785893	0,006077953	0,00979226	[6, 7]	Group22	8,08	8,00
GO:0060325	face morphogenesis	GO_BiologicalProcess-EBI-UniProt-GOA-ACAP-ARAP_08.05.2020_00h00	0,008195748	0,04546956	0,008195748	0,0099032	[3, 4, 5, 6]	Group03	12,50	4,00
GO:0045017	glycerolipid biosynthetic process	GO_BiologicalProcess-EBI-UniProt-GOA-ACAP-ARAP_08.05.2020_00h00	0,007937216	0,04464684	0,007937216	0,01000779	[4, 5, 6]	Group01	5,10	15,00
GO:0010631	epithelial cell migration	GO_BiologicalProcess-EBI-UniProt-GOA-ACAP-ARAP_08.05.2020_00h00	0,011507019	0,04957812	0,007653754	0,01008904	[4, 5, 6]	Group24	4,89	15,00
GO:0036303	lymph vessel morphogenesis	GO_BiologicalProcess-EBI-UniProt-GOA-ACAP-ARAP_08.05.2020_00h00	0,011417896	0,0497231	0,007653754	0,01008904	[3, 4, 6, 7, 8, 9]	Group24	15,79	3,00
GO:0038084	vascular endothelial growth factor signaling pathway	GO_BiologicalProcess-EBI-UniProt-GOA-ACAP-ARAP_08.05.2020_00h00	0,006395692	0,04390263	0,007653754	0,01008904	[6, 7, 8]	Group24	10,64	5,00
GO:0043534	blood vessel endothelial cell migration	GO_BiologicalProcess-EBI-UniProt-GOA-ACAP-ARAP_08.05.2020_00h00	0,004193723	0,036923	0,007653754	0,01008904	[6, 7, 8]	Group24	7,26	9,00
GO:0043535	regulation of blood vessel endothelial cell migration	GO_BiologicalProcess-EBI-UniProt-GOA-ACAP-ARAP_08.05.2020_00h00	0,003336776	0,03652417	0,007653754	0,01008904	[5, 6, 7, 8, 9]	Group24	8,16	8,00
GO:0061042	vascular wound healing	GO_BiologicalProcess-EBI-UniProt-GOA-ACAP-ARAP_08.05.2020_00h00	0,009795591	0,04779777	0,007653754	0,01008904	[5, 6, 7, 8, 9, 10, 11, 12]	Group24	16,67	3,00
GO:0090311	regulation of protein deacetylation	GO_BiologicalProcess-EBI-UniProt-GOA-ACAP-ARAP_08.05.2020_00h00	0,007632066	0,04415695	0,007632066	0,01053952	[6, 7, 8, 9]	Group07	10,20	5,00
GO:0120163	negative regulation of cold-induced thermogenesis	GO_BiologicalProcess-EBI-UniProt-GOA-ACAP-ARAP_08.05.2020_00h00	0,007632066	0,04415695	0,007632066	0,01053952	[3, 4, 5, 6, 7, 8]	Group08	10,20	5,00
GO:0062197	cellular response to chemical stress	GO_BiologicalProcess-EBI-UniProt-GOA-ACAP-ARAP_08.05.2020_00h00	0,009463804	0,04791051	0,009463804	0,01055578	[4]	Group16	4,66	18,00
GO:0034599	cellular response to oxidative stress	GO_BiologicalProcess-EBI-UniProt-GOA-ACAP-ARAP_08.05.2020_00h00	0,008637466	0,04484838	0,009463804	0,01055578	[4, 5]	Group16	4,92	16,00
GO:0034332	adherens junction organization	GO_BiologicalProcess-EBI-UniProt-GOA-ACAP-ARAP_08.05.2020_00h00	0,010195925	0,04801569	0,010195925	0,01056007	[6]	Group11	8,22	6,00
GO:0070231	T cell apoptotic process	GO_BiologicalProcess-EBI-UniProt-GOA-ACAP-ARAP_08.05.2020_00h00	0,010585342	0,04871663	0,010585342	0,01058534	[7]	Group06	9,43	5,00
GO:0030548	acetylcholine receptor regulator activity	GO_BiologicalProcess-EBI-UniProt-GOA-ACAP-ARAP_08.05.2020_00h00	0,006977784	0,04415629	0,006977784	0,0106503	[6, 7, 8, 9, 10, 11]	Group10	18,75	3,00
GO:0008134	transcription factor binding	GO_MolecularFunction-EBI-UniProt-GOA-ACAP-ARAP_08.05.2020_00h00	0,009982729	0,04756477	0,009982729	0,01072219	[3]	Group17	4,00	29,00
GO:0070491	repressing transcription factor binding	GO_MolecularFunction-EBI-UniProt-GOA-ACAP-ARAP_08.05.2020_00h00	0,000645692	0,01376343	0,009982729	0,01072219	[5]	Group17	10,53	8,00
GO:0140030	modification-dependent protein binding	GO_MolecularFunction-EBI-UniProt-GOA-ACAP-ARAP_08.05.2020_00h00	0,007459866	0,04378617	0,007459866	0,01081681	[3]	Group18	6,25	10,00
GO:0031593	polyubiquitin modification-dependent protein binding	GO_MolecularFunction-EBI-UniProt-GOA-ACAP-ARAP_08.05.2020_00h00	0,009784135	0,04832408	0,007459866	0,01081681	[4]	Group18	9,62	5,00
